# Supplementary material for: Changes in addressing inequalities in access to hospital care in Andhra Pradesh and Maharashtra states of India: a difference-in-differences study using repeated cross-sectional surveys
Source: BMJ Open. 2014 Jun 4;4(6):e004471. doi: 10.1136/bmjopen-2013-004471 (PMC4054646; doi:10.1136/bmjopen-2013-004471)
Supplement: Author's manuscript [file bmjopen-2013-004471.draft_revisions.pdf]

Trends in addressing inequalities in access to hospital care: A tale of two Indian states

Journal:	*BMJ Open*
Manuscript ID:	bmjopen-2013-004471
Article Type:	Research
Date Submitted by the Author:	19-Nov-2013
Complete List of Authors:	Rao, Mala; University of East London, Institute for Health and Human Development<br>Katyal, Anuradha; ACCESSHealth International,<br>Singh, Prabal; ACCESSHealth International,<br>Samarth, Amit; Sughavazhvu Healthcare,<br>Bergkvist, Sofi; ACCESSHealth International,<br>Kancharla, Manjusha; Indian School of Business,<br>Wagstaff, Adam; World Bank, Development Research Group<br>Netuveli, Gopalakrishnan; University of East London, Institute for Health and Human Development<br>Renton, Adrian; University of East London, Institute for Health and Human Development
**Primary Subject Heading**:	Public health
Secondary Subject Heading:	Health services research
Keywords:	Health economics < HEALTH SERVICES ADMINISTRATION & MANAGEMENT, Health policy < HEALTH SERVICES ADMINISTRATION & MANAGEMENT, PUBLIC HEALTH

SCHOLARONE™ Manuscripts

TITLE PAGE

TITLE:

Trends in addressing inequalities in access to hospital care: A tale of two Indian states

STUDY DESIGN: A cross-sectional difference in difference study with parallel control.

AUTHOR NAMES, ADDRESSES AND POSITIONS:

1.Mala Rao. Professor of International Health, Institute for Health and Human Development, University of East London, UK & Honorary Adviser, Administrative Staff College of India, Hyderabad

2. Anuradha Katyal, Consultant, ACCESS Health International, Hyderabad, India

3.Prabal V Singh, Director Research, ACCESS Health International, Hyderabad, India

4.Amit Samarth, Head, Strategy and Planning, SughaVazhvu Healthcare, Thanjavur, India

5.Sofi Bergkvist, Managing Director, ACCESS Health International, Hyderabad, India

6.Manjusha Kancharla, Research Associate, Indian School of Business, Hyderabad India

7.Adam Wagstaff, Research Manager, Human Development & Public Services team, Development Research Group (DECRG), The World Bank, Washington DC, USA

8.Gopalakrishnan Netuveli. Professor of Public Health, Institute for Health and Human Development, University of East London & ESRC International Centre for Life Course Studies in Society and Health, University College London, UK

9.Adrian Renton. Director, Institute for Health and Human Development, University of East London, UK

Corresponding author and guarantor of the data and information: Mala Rao (best email address: m.rao@uel.ac.uk).

For peer review only

ABSTRACT. WORD COUNT: 294

OBJECTIVES

To compare the effects of health innovations over time on access to and out of pocket expenditure (OOPE) on hospital inpatient care in Andhra Pradesh (AP) and Maharashtra (MH) states of India

STUDY DESIGN

A cross-sectional difference in difference (DID) study with parallel control.

SETTING

National Sample Survey Organisation of India urban and rural 'first stratum units', 863 in AP and 1008 in MH.

PARTICIPANTS

8623 households in AP and 10073 in MH

MAIN OUTCOME MEASURES

Average OOPE, large OOPE and large borrowing per household per year for inpatient care, hospitalisation rate per 1000 population per year.

RESULTS

Average expenditure, large expenditures and large borrowings on inpatient care had increased in both MH and AP, but the increase was smaller in AP across these 3 outcome measures. DIDs for average expenditure and large borrowings were significant and in favour of AP for the rural and the poorest households. Hospitalisation rates also increased in both states but more so in AP (5.6 per 1000 population vs 2.2), although the DID was not significant and the sub group analysis presented a mixed picture.

CONCLUSIONS

Health innovations in Andhra Pradesh had a greater beneficial effect on hospital inpatient care-related expenditures than innovations in Maharashtra. The Rajiv Aarogyasri Community Health Insurance scheme is likely to have contributed to these impacts in Andhra Pradesh at least in part. However, out of pocket expenditure increased in both states over time. Schemes such as the Aarogyasri and RSBY may result in some positive outcomes but, equity of access to health care and the reduction of the overall burden of out of pocket expenditure especially in the most vulnerable sections of the population are likely to require additional interventions that address gaps in the availability of and access to care.

For peer review only

For peer review only - http://bmjopen.bmj.com/site/about/guidelines.xhtml

ARTICLE SUMMARY

STRENGTHS AND LIMITATIONS OF THE STUDY

- This study uses a quasi-experimental design to compare changes between the Indian states of Andhra Pradesh and Maharashtra, in hospital inpatient care related expenditures and behaviours before and after the rollout of the Aarogyasri and RSBY schemes
- The study based on a survey of 18696 households has shown that health innovations in Andhra Pradesh had a greater beneficial effect on hospital inpatient care-related expenditures and access than innovations in Maharashtra. The Aarogyasri scheme is likely to have contributed to these impacts in Andhra Pradesh.
- The study also highlights the implications of the findings for policy and practice and additional interventions necessary to address gaps in the availability of and access to care.
- The study is only able to compare the effects of health innovations over time across the 2 states but does not allow the drawing of inferences on the impacts of individual initiatives.
- The study uses the difference in difference methodology and its findings may have been affected by unobservable differential changes between the 2 states.

For peer review only - http://bmjopen.bmj.com/site/about/guidelines.xhtml

For peer review only

ACKNOWLEDGEMENTS:

The study was funded by International Development and Research Centre, Canada, Wellcome Trust, the UK Department for International Development, and Rockefeller Foundation. The World Bank supported Adam Wagstaff's contribution to the study. We gratefully acknowledge the financial support of these organisations, as well as the enthusiastic encouragement we have received from them throughout the study. Our thanks also go to Bhimasankaram Pochiraju, Sundaresh Peri, C Ravi, and Rahul Ahluwalia for their advice and guidance at various stages of the study design and data analysis, and to colleagues at the University of East London and Administrative Staff College of India Hyderabad for their administrative support. The authors also thank P Suryanarayana, CM Reddy, D Chakrapani and K Pandu Ranga Reddy and AY Jadhav and his colleagues for their contribution to the training of the survey teams, verification of the survey and data collection and acknowledge the IMRB International Social Research Institute team's support in carrying out the survey.

We are grateful to Somil Nagpal and Joseph Kutzin for commenting on the report and helping us to improve it, to Sujatha Rao for her encouragement and valuable suggestions and ideas throughout the course of the study and to Jay Bagaria for her help during the early stage of the development of the proposal. Finally we thank PV Ramesh without whose constant support, guidance and encouragement, this study would not have been possible.

For peer review only

INTRODUCTION

In 2005, member states of the World Health Organization (WHO) committed to develop their health financing systems to deliver universal coverage (UC); that is that all people have access to health services and do not suffer financial hardship paying for them[1] . WHO's 2010 World Health Report[2] recommended *inter alia* that countries reduce reliance on direct payments, and improve equity of access, including through the introduction of prepayment schemes. However, one recent systematic review of the impact of national health insurance schemes in low and middle income countries[3] found only weak evidence of increased use of healthcare and reduced out-of-pocket expenses, with the poorer benefitting less, whilst others[4,5] concluded that health insurance improved health care access and use, as well as financial protection in most cases but had no conclusive impact on health status. Both highlighted the need for more rigorous assessments of such schemes.

India has one of the highest levels of out-of-pocket health expenditure (more than 80% of private health expenditure)[6]. The aim of our study was to explore how recently introduced health financing initiatives have affected access to and out-of-pocket expenditure (OOPE) on inpatient hospital care in the Indian states of Andhra Pradesh (AP) and Maharashtra (MH).

BACKGROUND

In its *Eleventh Five Year Plan (2007-12)*[7], Government of India sought to increase Public Expenditure on Health and to strengthen investment in rural health infrastructure through the National Rural Health Mission. Its *Twelfth Five Year Plan (2012-17)*[8], reflected the recommendation of the Planning Commission's High Level Expert Group for general taxation to be the principal source of health care financing. It proposed the development of government-funded health insurance schemes, building on the evidence from experimental schemes being introduced across many States. In India, health is primarily a state rather than national responsibility[6]. Whilst it is recognised that political will and good governance are essential, both the political mobilization of funds for health schemes and the effectiveness and efficiency of funded schemes will be enhanced by robust evidence which documents as to whether schemes achieve objectives, what works well and the main challenges they face.

In India, evaluation is not routine, even of large costly public health care programmes. Nevertheless, some assessments have been carried out, for example of the Yeshasvini Co-operative Farmers Health Care Scheme of Karnataka, the longest running state-supported health insurance scheme for the informal sector in India[9], with promising results in terms of increased utilization of and reduced borrowing for health care services[10]. However, these early models covered small populations and offered limited benefits, so that the policy implications of conclusions drawn from even the best evaluations were unclear.

This scenario has changed during the past 5 years, with the launch of 2 schemes, the Rajiv Aarogyasri[11] Community Health Insurance Scheme (Aarogyasri) of Andhra Pradesh (AP) and Rashtriya Swasthya Bima Yojana (RSBY) currently offered in 30 states and union territories of India[12] including AP's neighbouring state of Maharashtra (MH). Their scale in terms of population coverage and range of treatments offered, significantly enhances their potential to inform India's road map towards universal health coverage. Both schemes belong to the new generation of publicly funded government-sponsored health insurance schemes, principally aimed at providing financial protection to the poor against catastrophic health shocks, which, for these schemes, the Government has defined as inpatient hospital care[9]. Both schemes have been subject to some assessment in their early phases, but a recent editorial[13] highlighted the necessity of further and repeated evaluations to enhance the credibility and accountability of existing schemes and to identify those which deserve scale-up. The Aarogyasri and RSBY schemes and the other recent health sector innovations in AP and

For peer review only - http://bmjopen.bmj.com/site/about/guidelines.xhtml

Maharashtra which form the major backdrop and complex health care architecture against which these have been launched, and which may have contributed to the changes in outcomes we have explored in our study, are described below.

The Rajiv Aarogyasri Health Insurance Scheme of AP

In 2007 AP launched a pioneering new state-wide fully state-funded health insurance scheme, the Rajiv Aarogyasri Community Health Insurance Scheme (Aarogyasri)[11] to provide treatment for serious and life-threatening illnesses. The specific objectives include: to improve access of poor families to quality 'tertiary' medical care (meaning low-frequency, high cost specialist care) and treatment of identified diseases requiring hospitalisation through an identified network of health care providers, to provide financial cover for catastrophic illnesses which have the potential to wipe out life time savings of poor families and to provide 'universal coverage to the urban and the rural poor in the state[14] albeit for the conditions covered in the benefits package. All families with a 'below poverty line' (BPL) ration card, i.e. those on an annual income below USD 1384 (INR 75,000) in urban areas and USD 1107 (INR 60,000) in rural areas, and including individuals with pre-existing medical conditions are *automatically* enrolled and the scheme was estimated to cover approximately 20.4 million poor and lower middle class families, comprising about 85 percent of the state's population in 2009[9]. Enrollees make no contribution, the annual benefit is a maximum of USD 4,500 (INR 200,000) per family per year and there is no limit on the size of the family[14]. A total of 942 medical and surgical procedures across 31 clinical specialties [14] are provided and the benefits include all inpatient costs - associated investigations, food, transport and medicines for 10 days following discharge. One year follow-up packages including consultation, medicines, and diagnostics are also available for 125 procedures requiring longer periods of follow up[9] . Aarogyasri has unique features including *Aarogyamithras* (health system navigators), outreach *health camps* delivered by participating hospitals to educate, screen and case-find and a state-of-the-art information technology-based management system. At the time of this study, 353 public and private sector hospitals were 'empanelled' to provide services to Aarogyasri beneficiaries.

In 2009, a descriptive study of *Aarogyasri*, based on an analysis of claims data and a survey of beneficiaries[15], concluded that while the scheme was beginning to reach its intended beneficiaries uptake was lower among scheduled castes and tribes. This was confirmed by Fan and colleagues[16], who used variations in programme roll-out over time and districts to evaluate the scheme using National Sample Survey data collected before and after its launch. They reported reduced out-of-pocket expenditure in this initial phase but no major impact on catastrophic healthcare expenditure. Inspired by Aarogyasri and mindful of the political benefits of introducing popular health reforms, other states have launched health financing innovations similar to this model.

RSBY in Maharashtra

RSBY was launched across a number of states by the Ministry of Labour, Government of India (GOI) in 2008[17] and provides access to free inpatient hospital care up to USD 550 (INR 30,000) per family per year[18]. Households which meet the criteria based on the much more limiting definition of poverty and numbers of poor families provided for each State by the GOI Planning Commission are eligible to enrol, and pay a contribution of USD 0.55 (INR 30) at registration and at each annual renewal[9]. Up to 5 family members, including those with pre-existing conditions can be covered, and personal information including biometric data are collected prior to the issue of a smart card with encoded details of the family. 700 procedures covering 18 broad categories of interventions which would generally be included under the umbrella of 'secondary' care, are provided and the benefit packages include the intervention, public transport costs limited to 1.8 USD (INR 100) per visit and 18.2 USD (INR 1000) per year and post-hospitalization drugs for 5 days. Networked hospitals are required to provide free outpatient consultations (which have only recently been introduced. Personal communication, Kurian OC) but other costs such as ambulatory diagnostics and medicines

For peer review only - http://bmjopen.bmj.com/site/about/guidelines.xhtml

have to be borne by the beneficiaries, except if investigations lead to inpatient admissions within a day[9]. A pilot of the RSBY scheme was launched in 1 district of AP, but only after the start of our household survey.

In Maharashtra, enrolment began in Aug 2009, and by mid 2013, approximately 2 million of the eligible 4 million families were enrolled in the scheme[12] which is being implemented in 31 of 35 districts in the state. Enrolment had extended to 26 districts prior to June 2012, when our household survey began. Notably, only 15 out of 1215 hospitals contracted for RSBY funded services are from the public sector [12]. Early assessments of the scheme's impact nationally, suggest that although the rate of hospitalisations has increased, awareness of the scheme was poor and remains a barrier to uptake. Notable variations in enrolment and scheme awareness were also observed by a descriptive study of RSBY conducted in the Amravati district of Maharashtra which has a large tribal population[19]. In Maharashtra[17], utilisation rates have been reported to be lower than in other states and the male:female enrolment ratio is 6.5:3.5.

Other major health sector initiatives in AP and Maharashtra

Both states have a complex health care landscape with numerous programmes in place. There are several initiatives launched during the past decade, some of which are common to both states and driven by national strategies, and others owe their existence to state-level enterprise, innovation and political support. The most notable programmes with the potential to impact on in patient care are described below.

The National Rural Health Mission (NRHM) was launched in 2005 nationwide, with a key aim of reducing maternal and infant mortality[20]. Government reports suggest that its notable achievements include an increase in institutional deliveries; in AP from 1.25 million in 2005-06 to 1.46 million by 2011-12[21] and in MH from 1.1 million to 1.63 million[22], achieving an institutional delivery rate of approximately 92 percent in both states. Also common to both states is the '104 health information help line' launched in AP in 2008 and MH in 2011[23], to provide medical advice and information based on validated algorithms and disease summaries, direct callers to appropriate health facilities or to receive a complaint against a public sector health facility. In AP the help line and call centre were subsumed within the Aarogyasri infrastructure by 2011.

In MH, the RSBY was preceded by the Jeevandayee scheme launched in 1997 with the objective of reducing catastrophic OOPE on inpatient care in the BPL population[24]. Potential beneficiaries were required to apply for funding after a diagnosis was confirmed and the scheme covered serious illness such as cardiac and renal disease and cancer. However, the scheme uptake has been low, and while it has continued to run in parallel to the RSBY, only 66,853 procedures (4456 procedures per year in a state with 112.37 million people) have been approved during the scheme's lifetime[25]. Since 2006, MH has also had a scheme in place which mandated 20% of the beds in private hospitals to be made available for free or at subsidized rates to poor patients (personal communication, Kurian OC). It has been estimated that around 10,000 private beds are available for the poor across MH, equivalent to approximately 20% of the total bed capacity of the public sector. Although the implementation of the scheme is reported to be erratic (Kurian OC), it may have had some positive impact on access to hospital inpatient care for serious illness.

Launched in 1995-96, the Navasanjeevani Yojana scheme is exclusive to the 15 tribal districts of MH and was to improve maternal and infant mortality in these vulnerable populations[26]. It has focused on strengthening primary health and nutrition services and access to safe drinking water.

A service available in AP but not in MH is the '108' scheme, launched in 2005 to provide a state-of-the-art medical emergency response service[27]. At the time of our study, 802 ambulances catered to approximately 3,500 emergencies per day[28].

For peer review only - http://bmjopen.bmj.com/site/about/guidelines.xhtml

OBJECTIVES OF THE STUDY

Our objective was to compare the effects of health innovations over time on access to and OOPE on inpatient care in AP and MH and to assess whether the AP initiatives had larger or smaller beneficial effects than those found in MH. These differential effects are likely to be substantially due to the Aarogyasri scheme in AP and the RSBY in MH. In this paper, we report findings from a study which compared these trends. The findings do not allow us to draw inferences on the impacts of individual initiatives, but nevertheless contribute new knowledge on the impact and role of the innovations, provide lessons for other programmes, and strengthen the evidence base for policy on UC in India.

METHODS

Overview

None of the aforementioned initiatives – including the Aarogyasri and RSBY schemes in which we are especially interested – was piloted in a systematic way, let alone via a carefully designed randomized control trial. Following MRC Guidance[29,30] and best practice we therefore opted for a quasi experimental design in which we seek to minimise selection bias, to control for confounding variables and to reduce the effects of chance. Specifically, we compare changes in hospital inpatient care related expenditures and behaviours (HREB) in AP and MH before and after the rollout of the Aarogyasri scheme in AP and the RSBY scheme in MH. The difference in changes between AP and MH is not an estimate of a specific initiative. Rather it tells us whether, on balance, the AP initiatives have had larger (or smaller) beneficial effects than the MH initiatives, and if so how much more (or less) beneficial they have been. Since the NRHM was common to both states and the MH-specific initiatives were quite small in scale or unlikely to affect HREBs, any difference in change between the two states is quite likely to be mainly due to differential effects of the Aarogyasri and RSBY programmes.

AP and MH have a similar development profile (Figure 1). AP's other socio-economically similar neighbouring states of Karnataka and Tamil Nadu had already introduced Aarogyasri-like schemes, and Odisha and Chattisgarh the only other neighbours, had comparatively higher levels of socio-economic deprivation. HREBs were measured in both AP and MH by two waves of household survey before (2004) and after (2012) the introduction of Aarogyasri and RSBY. The study protocol and questionnaire for the 2012 survey were reviewed and agreed by the Research Ethics Committee of the Administrative Staff College of India, Hyderabad.

Figure 1. Location of and selected indicators for Andhra Pradesh and Maharashtra

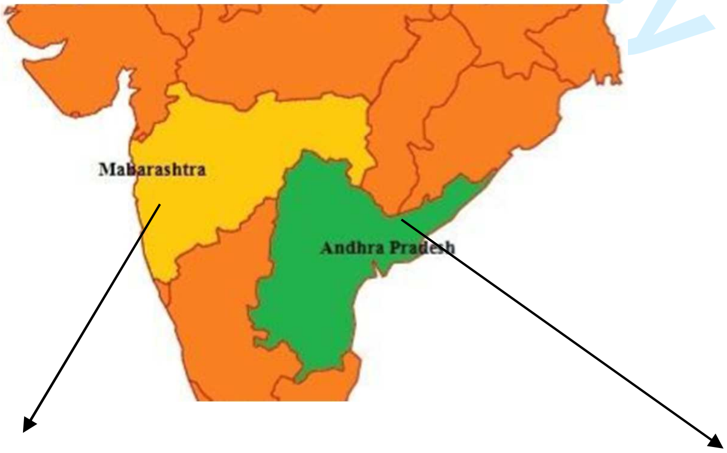

For peer review only

For peer review only - http://bmjopen.bmj.com/site/about/guidelines.xhtml

Maharashtra	Indicator	Andhra Pradesh
112.37	Population (2011 census, in millions)	84.66
10.20	% Schedule Caste (2001 Census)*	16.60
8.90	% Schedule tribe (2001 census)*	6.20
101,314	Per capita income 2011-12 (in INR)**	71,540
35	Number of districts	23
5,314	Households covered in NSSO 60th round (2004-05)	5,059
10,073	Households covered in the study 2012	8,623

*Note that 2011 census data for social groups are not yet published

**Source: Presentation on Annual Plan 2012-13 and Five Year Plan 2012-17[31]

Survey design

Baseline Survey : 2004

We used the original data from the National Sample Survey Organization (NSSO) 60th decennial round household survey undertaken in 2004[32] to estimate baseline HREB estimates for AP and MH (Table 1). This was the most recent round measuring morbidity profiles, use of health care services including hospitalised and non-hospitalised treatments and expenditures incurred. The household survey used a multi-stage stratified sampling methodology to identify a representative random population sample and an interviewer completed questionnaire to obtain measures of HREB along with socio-demographic, household expenditure and other information. (see Online Appendix for full technical details of sampling method and full questionnaire).

Follow up survey: 2012

We used the same household survey design and methods to collect post-intervention data in AP and MH as those used by NSSO. Briefly, the household survey used a multi-stage stratified sampling methodology with the 'First Stage Units' (FSUs) identical to those used by NSSO in their 66th round (2008-09)[33], the latest round for which FSUs had been mapped. However, the FSUs were not the same as those in NSSO 2004, our baseline survey, rapid urbanisation having changed substantially, the urban-rural landscape of both states and thus the geographical basis for sampling units.

Table 1: Urban and rural populations and households surveyed in 2004 and 2012 in Andhra Pradesh and Maharashtra

	Andhra Pradesh		Maharashtra	
	NSSO 60th round 2004-05	Our survey 2012	NSSO 60th round 2004-05	Our survey 2012
Population	76,210,007*	84,665,533**	96,878,627*	112,372,972**
Urban population	20,808,940*	28,353,745**	41,100,980*	50,827,531**
Rural population	55,401,067*	56,311,788 **	55,777,647*	61,545,441**
Total households (urban)	4,397,138*	6,778,225**	8,403,224*	10,813,928**
Total households (rural)	12,607,167*	14,246,309**	11,173,512*	13,016,652**
Total households	17,004,305*	21,024,534**	19,576,736*	23,830,580**
FSUs (urban)	183	372	267	504
FSU (rural)	325	491	265	504
Total households	1824	3715	2664	5038

For peer review only - http://bmjopen.bmj.com/site/about/guidelines.xhtml

surveyed (urban)				
Total households covered (rural)	3235	4908	2650	5035

*2001 census ** 2011 census
FSU - First Stratum Unit
The NSSO 66th round had 492 rural FSUs in AP, but 1 FSU was found to be uninhabited.

The interviewer completed household survey questionnaire was pre-tested and then piloted in both states prior to the survey. All respondents provided written informed consent for participation. Questions addressed the following: household composition and socio-demographic characteristics of members, household expenditure, and health expenditure (outpatient and inpatient) and means of its financing, healthcare seeking behaviour, factors affecting access to healthcare and awareness and perceptions of the quality of the *Aarogyasri* scheme (AP only). The survey questions in 2012 were identical to those from the NSSO 2004[34]. Additional questions specific to the Aarogyasri and other relevant schemes were also added.

A survey of 18696 households across 2 states and 1871 locations within the states is a challenging undertaking. The survey design had several features intended to assure the quality of data collected. Few academic institutions have internal capacity to carry out such large surveys, and consequently, the Social and Research Institute of IMRB International, a leading market research agency was selected to carry out the survey. The Institute has field survey teams based in every Indian state, conversant in local languages and dialects and trained to carry out surveys in the socio-economic development sector. Its clients include Government of India (for whom the national Family Health Survey data are collected), World Bank and other UN organisations. A group of NSSO consultants in AP and the Indian Socioeconomic Research Unit, Pune were recruited to support the training of the field survey teams and data verification.

We planned three levels of verification of the study data; the first to be undertaken by the survey agency, the second to be carried out by the study team and the third, by the agencies mentioned above. Survey teams for each district were accountable to a field supervisor who was responsible for checking both the household listing and data entry on a daily basis. The study team also accompanied the field staff to survey sites on a regular basis. Data collected from 250 households in each state (approximately 2.5% of the surveyed households) and 186 of the FSU listings (approximately 10%) were independently verified by the agencies in both the villages and urban blocks in order to ensure that the sampling method and administration of the questionnaire survey were being correctly applied. The data entry was carried out by the Institute using a double entry method and any questionnaires reported incorrect were sent back to the field for re-survey. The research team carried out a final validation and review of the data.

Outcome measures

Average inpatient (IP) expenditure per household per year. Average out-of-pocket expenditure for inpatient care during 1 year prior to the survey was estimated from questionnaire responses for AP

For peer review only - http://bmjopen.bmj.com/site/about/guidelines.xhtml

and MH from both baseline and follow-up data. Reimbursements for inpatient expenditure were deducted from the total where households had received them.

Large out-of-pocket inpatient expenditure. Because of the limited data on household consumption in the 2004 NSSO health survey we did not estimate 'catastrophic health expenditure'. Instead, we constructed a measure of 'large' out-of-pocket expenditure. The Aarogyasri Health Care Trust data on expenditure incurred by the Government of AP per case in 2012 were examined[14] and the mean was estimated as USD 419 (INR 23,000). A household was deemed to have incurred 'large' expenditure if OOPE for inpatient care was equal to or greater than this threshold.

Large borrowing. We estimated the total amount borrowed by a household to meet the expenditure of all the inpatient episodes of that family during the previous year. A household was considered to have incurred 'large borrowing' if the borrowing was equal to or exceeded the BPL threshold set by Government of AP: INR 70000 for urban families and INR 65000 for rural households. These prices have been deflated to 2004 levels.

Hospitalisation rate. This was estimated as the number of individuals hospitalised during the previous year, per 1000 population.

Variations in outcomes were examined between male and female-headed households, and rural and urban populations, as well as across social groups and economic groups represented by asset quintiles.

Because of the limited data on household consumption in the 2004 NSSO health survey which made the estimation of wealth difficult, we have opted instead to measure household living standards using an asset or wealth index based on information on ownership of household durables, dwelling type, etc., using principal component analysis to estimate weights[35, 36,37] for each indicator. The indicators used were limited to those collected in the 2004 NSSO: type of structure of the dwelling unit, type of toilet, type of fuel used for cooking and source of drinking. Data from the 2004 and 2012 surveys were pooled so that the index captures changes in living standards between the two years; there are therefore more households in the top quintile in 2012 than in 2004. The statistical software Stata 11 was used to generate the index.

Deflation of follow-up expenditure estimates.
The 2012 expenditure data including the threshold for large expenditures were deflated using the consumer price index of the Government of India[38] to reflect 2004 prices.

Analysis
Our repeat cross-sectional surveys do not allow estimation of within-individual household changes in outcomes over time. Our analysis therefore focused on estimating outcomes averaged across states, and in comparing changes in these over time between AP and MH. If we assume that outcome determinants other than Aarogyasri and RSBY remained stable in the two states over time or followed a parallel trend, then a difference-in-difference (DID) analysis will uncover the net effect of Aarogyasri over and above RSBY.

The difference in differences of outcome (Y_{DD}) is

$$(Y_{2012}^{AP} - Y_{2004}^{AP}) - (Y_{2012}^{MH} - Y_{2004}^{MH})$$

where the subscripts and superscripts for Y refer to the respective states and the years when the surveys were done. Confidence intervals were calculated from the standard error Y_{DD} of and the p-

For peer review only - http://bmjopen.bmj.com/site/about/guidelines.xhtml

value for the null-hypothesis ($Y_{DD} = 0$) was tested using the Wald test as $t = \frac{Y_{DD}}{SE_{Y_{DD}}}$ with one degree of freedom. Y_{DD} was estimated using ordinary least square regression:

$$y_{it} = \beta_0 + \beta_1 state_i + \beta_2 survey_t + \beta_3 (state * survey)_{it} + \sum_{k=1}^{m} \beta_{3+k} covariate_k + \varepsilon$$

The basic DID results are obtained using the above regression with covariates excluded. The adjusted DID results are obtained using the above regression with m=9 covariates, namely the gender of head of household, a dummy variable capturing whether the household lives in a rural or urban location, three dummy variables capturing the household's social group (the lowest is the excluded category), and four asset quintile dummies (the bottom is the excluded category). In the regression y_{it} is the outcome, *state* is a dummy variable with 0 for MH and 1 for AP, and *survey* is a dummy variable with 0 for 2004 survey and 1 for 2012 survey. The coefficient for the interaction term, β_3, gives the differences in differences estimate, Y_{DD}. Robust standard errors of Y_{DD} were calculated to account for clustering of households within FSUs using Stata survey commands. A positive value for Y_{DD} suggested that the change in the outcome in AP was more than the change in MH and the negative value would suggest the reverse.

Sub groups were not mutually adjusted for the analysis due to sample size restrictions in relation to some of them.

Role of the funding sources

The external funding sources had no role in study design, data collection, analysis, interpretation or reporting, or in submission decision.

RESULTS

A total of 5314 and 5059 households from Maharashtra and AP were surveyed by the NSSO in 2004 (Table 2). Our survey in 2012 included 10073 (MH) and 8623 (AP) households.

For peer review only

Table 2. Socio-demographic characteristics of baseline and follow-up samples

Subgroups	Number (%) of Households 2004		Number (%) of Households 2012	
	Maharashtra	Andhra Pradesh	Maharashtra	Andhra Pradesh
**All**	5314	5059	10073	8623
**Head of Household**				
Male	4785 (90.0)	4433(87.6)	8543(84.8)	7418(86.0)

Female	529 (10.0)	626 (12.4)	1530 (15.2)	1205 (14.0)
**Social Group**				
Scheduled Tribes	413 (7.8)	296 (5.9)	1364 (13.5)	883 (10.2)
Scheduled Castes	809 (15.2)	974 (19.3)	2235 (22.2)	1797 (20.8)
Other Excluded	1644 (30.9)	2317 (45.8)	1899 (18.9)	3419 (39.7)
All other Groups	2448 (46.1)	1472 (29.1)	4571 (45.4)	2524(29.3)
**Location**				
Rural	2,650 (49.9)	3235 (63.9)	5035(50.0)	4908 (57.0)
Urban	2664 (50.1)	1824 (36.1)	5038 (50.0)	3715(43.0)
**Asset Quintile**				
Lowest	1,260 (23.7)	1,594(31.5)	996(9.9)	826(9.6)
Second	1,016 (19.1)	1,237 (24.5)	1,841(18.2)	1,286(14.9)
Third	772 (14.5)	753(14.9)	2,228(22.1)	2,121(24.60)
Fourth	857 (16.1)	744(14.7)	2,373 (23.6)	3,072(35.6)
Fifth	1,408(26.5)	730(14.4)	2,633(26.1)	1,318(15.3)

Changes in average in-patient expenditure

Table 3 (top panel) shows average baseline levels of inpatient expenditure. The table also shows the real terms change (deflated to 2004 prices) in these outcomes at follow up and the difference in difference (DID) estimate comparing AP with MH. DIDs for overall results are shown unadjusted, as well as adjusted for the effects of the covariates. Breakdowns by sex of head of household, social group, urban/rural location and asset quintiles are also shown.

Overall, average inpatient expenditure increased in real terms in both the states between 2004 and 2012, but the increase was significantly greater in MH (unadjusted DID= -498.2 INR, 95% CI= -792.9: -203.5, p=0.0009). The trend of a greater increase in MH was evident across all sub groups of analysis except the richest asset quintile. However, the DIDs reached significance in male headed households (DID=-513.7INR, 95% CI= -843.9: -183.4, p=0.0023), scheduled castes (DID= -708.7INR , 95% CI=-1234.3: -183.2, p=0.0082), all 'other' social groups (DID=-1110.46INR, 95% CI=-1868 : -352.9, p=0.0041), rural households (DID= -504 INR, 95% CI= -801.9: -206.0, p=0.0009) and the poorest (DID= -1001.3 INR, 95% CI= -1751: -251.7, p= 0.0089) and middle asset quintiles (DID= -798.1 INR, 95% CI= -1362.9:-233.3, p= 0.0056).

Table 3: Change in average inpatient expenditure (in INR) in Maharashtra and Andhra Pradesh between 2004 and 2012

Subgroups	Baseline Mean (95% CI)		Change 2004:2012 Mean (95% CI)		DID	
	Maharashtra	Andhra Pradesh	Maharashtra	Andhra Pradesh	Mean (95%CI)	p
Household inpatient expenditure						

For peer review only - http://bmjopen.bmj.com/site/about/guidelines.xhtml

**All**	1091.6(978.4:1204.8)	723.5(527.5:919.5)	942.8(749.9:1135.6)	444.55(221.5: 667.6)	-498.2(-792.9:-203.5)	0.0009
					**DID (adjusted for covariates)**	
					-565.8(862.9: -268.6)	0.0002
**Head of Household**						
Male	1,132.9(1015:1,251)	758(419.7:1,096.3)	935(727:1,143.01)	1074.9(555.9:1593.8)	-513.7(- 843.9: - 183.4)	0.0023
Female	757.1(555.5:1014.6)	341.1(222.3:460.01)	421.3(164.7:678.0)	589.9(307.22:872.8)	-484.9(- 1075.6:105.9)	0.1076
**Social Group**						
Scheduled Tribes	376.6(231.7:521.6)	432.7(212.52:652.9)	1153.1(803.3:1502.9)	675.2(163.2:1187.2)	-477.9(-1097.7:142)	0.1307
Scheduled Castes	696.7(500.2:893.2)	432.6(305.6:559.4)	1464.1(1039.9:1888.4)	755.4(444.9:1065.9)	-708.7(-1234.3: - 183.2)	0.0082
Other Excluded	1,028.6(838.4:1218.8	562.4(463.7:662)	928.9(532.9:1324.9)	767.9(569.6:966.2)	-161(-603.7: 281.7)	0.4758
All other Groups	1,424.5(1,222.3:1626.7)	1306.2(627.8:1984.6)	734.9(427.9:1041.7)	-375.6(-1,068.5:317.4)	-1110.46(-1868 : -352.9)	0.0041
**Location**						
Rural	897.8(768.1:1027.5)	571.4(496.2:646.6)	1084.7(826.3:1343.1)	580.7(432.2:729.2)	-504(-801.9: -206.0)	0.0009
Urban	1343.5(1146.1:1540.9)	1113.5(466.2:1760.8)	753.6(458.7:1048.6)	92.3(-586.92:771.5)	-661.3(-1401.5: 78.864)	0.0799
**Quintile**						
poorest	656.3(498.0:814.6)	391.5(319:464.1)	1692.5(1053.3:2331.7)	691.2(298.9:1083.5)	-1001.3(-1751: -251.7)	0.0089
2nd	786.5(583.5:989.5)	443.3(356.5:530.2)	979.3(599.4:1359.2)	839.5(465.7:1213.3)	-139.8(-672.5:393)	0.607
middle	1062.7(738.8:1386.1)	862.1(577.8:1146.5)	1011.8(550.2:1473.4)	213.7(-112.1:539.6)	-798.1(- 1362.9: - 233.3)	0.0056
4th	1241.7(894.4:1589.1)	1819(337.5:3302.5)	803.6(328.7:1278.5)	-644.3(-2128.3: 839.7)	-1447.9(-3005.2:109.5)	0.0684
richest	1818.6(1505.5:2131.8)	908.3(682.1:1133.4)	252.3(-193.4:698.1)	362.1(15.3:708.9)	109.7(- 454.80:674.3)	0.7031

Large expenditures for inpatient care

Proportions of households incurring large expenditures showed an increase in both states (Table 4), but the increase was smaller in AP for the sample as a whole as well as for all the groups except for the second asset quintile. The DID was strongly significant for the households overall (adjusted DID=-1.8, 95% CI: -3:-0.7, p= 0.0009), but this was not observed for any of the sub groups of analysis.

For peer review only - http://bmjopen.bmj.com/site/about/guidelines.xhtml

For peer review only

Table 4: Change in the proportion (%) of households incurring large health expenditures for in-patient care in Maharashtra and AP between 2004 and 2012

Subgroups	Baseline Mean (95% CI)		Change 2004:2012 Mean (95% CI)		DID	
	Maharashtra	Andhra Pradesh	Maharashtra	Andhra Pradesh	Mean (95%CI)	p
Large IP expenditure (INR 23000 deflated to 2004 figures)						
**All**	6.7(6:7.3)	3.4(2.9:3.9)	3.1(2.1:4.1)	2.2(1.5:2.8)	-0.91(-2.1:0.27)	0.1302
					**DID (adjusted for covariates)**	
					-1.8(-3:-0.7)	0.0009
**Head of Household**						
Male	6.8(6.2:7.6)	3.5(3.1:4)	3.1(2.0:4.1)	2.1(1.5:2.9)	-0.8(-2.1:-0.4)	0.1928
Female	5.0(3.7:6.4)	2.8(1.9:3.7)	3.9(2:5.8)	2(6.6:3.4)	-1.8(-4.2:0.50)	0.1222
**Social Group**						
Scheduled Tribes	2.2(1.3:3.1)	1.4(0.5:2.2)	5.3(3.5:7)	3.5(1.9:5.1)	-1.7(-4.1:0.61)	0.1478
Scheduled Castes	6.1(4.6:7.7)	2.1(1.5:2.6)	4.3(2.3:6.3)	3(1.9:4.1)	-1.2(-3.5 :1.01)	0.2785
Other Excluded	5.9(4.7:7.0)	3.3(2.7:3.8)	2.9(1.1:4.6)	2.7(1.7:3.6)	-2.1(-2.2:1.8)	0.8389
All other Groups	8.3(7.5:7.9)	5.4(4.4:6.3)	2.2(0.9:3.6)	0.51(-0.7:1.7)	-1.7(-3.5:0.04)	0.0628
**Location**						
Rural	1.9(1.5:2.2)	0.9(0.79:1.1)	1.7(1.1:2.3)	1.3(0.09:1.6)	-0.45(-1.1:0.25)	0.2098
Urban	12.9(11.9:14)	9.7(8.9:10.7)	4.4(3.0:5.7)	3.9(2.6:5.3)	-0.7(-2.4: 1.5)	0.6350
**Quintile**						
poorest	1.8(1.3:2.4)	1.1(0.8:1.4)	3.7(2.2:5.2)	1.7(0.7:2.7)	-0.2(-3.8:-0.19)	0.0307
2nd	2.7(1.9:3.5)	1.2(0.9:1.5)	2.1(0.93:3.3)	2.2(1.4:3.1)	0.9(-1.4:1.6)	0.9079
middle	6.9(4.9:8.9)	4.2(3.1:5.3)	1.3(-1:3.6)	0.9(-1.2:1.4)	-1.2(-3.9:1.4)	0.3596
4th	10.7(8.7:12.6)	7.6(5.9: 9.2)	1.8(-0.57:4.3)	-0.036(-1.9:1.80)	-1.9(-4.9:1.2)	0.2268
richest	13.5(12:14.9)	9.6(8.1:11.2)	0.3(-1.6:2.2)	-0.6(-2.8:1.6)	-0.9(-3.7:2)	0.5601

Changes in large borrowing for inpatient care

In both states proportions of households incurring large borrowings to meet inpatient expenses increased from 2004 to 2012 (Table 5). However, there was a consistent pattern of smaller increases in AP for the overall population, as well as all subgroups (except the richest asset quintile) with DIDs strongly significant for the overall population (adjusted DID=-4, 95% CI: -6.6:-1.4, p= 0.0032), scheduled tribes (DID= -5.5, 95% CIs:-9.3:-1, p= 0.0048), rural households (DID= -4.7, 95% CIs: -7.3 : -2.1, p= 0.0007) and all asset quintiles except the richest (the poorest asset quintile DID= -9.0, 95% CI: -14.0: -4.4, p=0.0002).

Table 5: Change in the proportion (%) of households large borrowings for in-patient care in Maharashtra and AP between 2004 and 2012

For peer review only - http://bmjopen.bmj.com/site/about/guidelines.xhtml

Subgroups	Baseline Mean (95% CI)		Change 2004:2012 Mean (95% CI)		DID	
	Maharashtra	Andhra Pradesh	Maharashtra	Andhra Pradesh	Mean (95%CI)	p
**Proportion of households having large borrowings**						
**All**	7.5(6.7:8.2)	3.8(3:4.5)	8.9(6.8:11)	5.3(3.4:7.2)	-3.7(-6.4:-0.908)	0.0100
					**DID (adjusted for covariates)**	
					-4(-6.6:-1.4)	0.0032
**Head of Household**						
Male	7.8(7.0:8.5)	3.9(3.1:4.7)	9.8(6.6:1.3)	5.3(3.3:7.3)	-3.6(-6.6: -0.62)	0.0187
Female	5(3:7)	2.9(1.7:4.1)	8.(6.6:11.2)	5(2.9:7.23)	-4.7(-8.3:-1)	0.0137
**Social Group**						
Scheduled Tribes	3.6(2.3:4.9)	2.4(0.93:3.9)	11(8.9:14)	5.8(2.8:8.8)	-5.5(-9.3: -1.8)	0.0048
Scheduled Castes	7.2(5.5:8.8)	3(1.7:4.2)	9.6(6.6:13)	5.8(3.4:8.3)	-3.8(-7.5:0.03)	0.0518
Other Excluded	8.0(6.8:9.2)	3.5(2.7:4.4)	8(5.8:10.3)	5.3(3.2:7.4)	-2.8(-5.7:0.19)	0.0661
All other Groups	8(7.15:8.8)	0.052(.040:0.064)	8.8(5.9:12)	4.7(2.2:7.4)	-4.1(-7.9:-.4.0)	0.0302
**Location**						
Rural	6.5(5.6:7.5)	0.03(0.024:0038)	10(8.5:12)	5·8(3.9:7.6)	-4.7(-7.3:-2.1)	0.0007
Urban	8.7(7.3:10)	0.056(0.048:0.064)	7.0(4.5:9.5)	4(1.1:6.9)	-3.0(-6.7:0.68)	0.1081
**Quintile**						
poorest	5.2(3.9:6.5)	0.025(0.016:0.033)	12.1(7.8:16)	3.1(1.3:0.049)	-9(-14:-4.4)	0.0002
2nd	0.064(0.048:0.08)	0.027(0.021:0.032)	0.095(0.070:0.12)	0.052(0.034:0.070)	-0.043(-.073: -.013)	0.0062
middle	0.074(0.050:0.098)	0.048(0.031:0.065)	0.10(.073:0.133)	0.044(0.013:0.076)	-0.059(-.100:-.017)	0.0069
4th	0 .087(0.063:0.110)	0.06(.041:0.078)	0.083(0.061:0.104)	0.039(0.014:0.064)	-0.044(-.075:-.012)	0.0076
richest	0.10(0.090:0.12)	0.064(0.049:0.079)	0.045(0.0035:0.086)	0.049(-.0068:0.105)	0.0045(-.062:0.071)	0.8937

Hospital utilisation for in-patient care

Overall, hospitalisation rates have increased in both AP and Maharashtra (Table 6) but more so in AP (5.6 per 1000 population vs 2.2), although the DID was not statistically significant. The sub group analysis presented a mixed picture. For both male and female headed households there was a greater increase in hospitalisation in AP, but this reached moderate statistical significance only for female headed households (DID=27.6, 95% CI =1.1:54.1, p-value = 0.0415). There is an increase in hospitalisations among scheduled tribes in Maharashtra and a reduction in AP (DID = -19.8, 95% CI = -37.3: -2.3, p-value = 0.0272) but the opposite trend was seen among 'other excluded' groups with an increase in AP and a reduction in MH (DID = 12.5, 95% CI= 1.2:23.9 p-value = 0.0309). In scheduled castes hospitalisations had increased in both states but more so in AP, while in the 'other' group there was a small increase in MH and a small reduction in AP. In the poorest quintile, the increase in hospitalisation was significantly greater in MH (DID= -14.4, 95%CI=-28:-0.31, p= 0.0451).

Table 6: Changes in hospitalisation (per 1000 population) in Maharashtra and AP between 2004 and 2012

For peer review only - http://bmjopen.bmj.com/site/about/guidelines.xhtml

Subgroups	Baseline Mean (95% CI)		Change 2004:2012 Mean (95% CI)		DID	
	Maharashtra	Andhra Pradesh	Maharashtra	Andhra Pradesh	Mean (95%CI)	p
Hospitalizations per 1000 population						
**All**	41.3(37.3:45.2)	31.5(27.8:35.3)	2.2 (-4.7:9.1)	5.6(-1.1:12.3)	3.4(-5.9 :12.7)	0.4636
					**DID (adjusted)**	
					0.7(-8.6:10.2)	0.8685
**Head of Household**						
Male	41.0(37.1:44.9)	31.7 (16.9:34.0)	1.9(-5.1:8.8)	4.4(-2.4:11.1)	2.5(-6.9:11.9)	0.5966
Female	51.6 (30.6:72.5)	25.5(27.7:35.7)	13.9(-7.53:35.4)	41.5 (24.6:58.4)	27.6(1.1:54.1)	0.0415
**Social Group**						
Scheduled Tribes	23.7(14.2 :33.1615)	35.5(21.9:49.1)	17.1(5.8:28.5)	-2.7(-16.95:11.5)	-19.8(-37.3: -2.3)	0.0272
Scheduled Castes	42.4(36.3:48.4)	29.5(22.6:36.5)	3.9(-7.9:15.7)	7.6(-2.6:17.7)	3.7(-11.4:18.7)	0.6268
Other Excluded	44.2(38.2:50.2)	29.9(25.5:34.3)	-1.9(-11.2 :7.3109)	10.6(3.4:17.8)	12.5(1.2:23.9)	0.0309
All other Groups	42.5(37.1:47.9)	34.9(29.0:40.9)	0.93(-7.3:9.1)	-1.1(-9.4: 7.4)	-2.0(-13.5 :9.4)	0.7235
**Location**						
Rural	36.6(32.2:41)	28.9(26.4:31.5)	9.5(3.5:15.4)	8.8(2.2:15.3)	-0.69(-9.3:7.9)	0.8725
Urban	48.2(43.7:53)	38.2(35.5:41.2)	-7.9(-14.5: -1.3)	-2.5(-12.1:7.1)	5.4(-5.8:16.6)	0.3358
**Quintile**						
poorest	31.4(25.9:36.9)	27.5(22.8:32.1)	20.7(9:32.8)	6.4(-1.7:14.4)	-14.4(-28:-0.31)	0.0451
2nd	36.5(28.3:44.7)	27.1(21.9:32.4)	8(0.5:15.4)	8.9(-.56:18.4)	0.9(-10 : 12.5)	0.8746
middle	47.8(33.6:62.1)	35.3(29.0:41.6)	-1.5(-17.9:14.8)	4.1(-.56:13.7)	5.6(-12.8:24.0)	0.5457
4th	46.9(39.9:53.9)	41.7(32.7:50.8)	-4.0(-12:3.4)	-5.1(-15.7:5.4)	-0.75(-13.3:11.9)	0.9056
richest	51.0(46:56.1)	36.5(25.8:47.2)	-13.1(-18.9: -7.1)	1.5(-19.5:22.6)	15.0(-5.8: 3.6)	0.1665

LIMITATIONS OF THE STUDY

Difference in difference estimations aimed at assessing the impacts of interventions, assume that both populations demonstrate similar characteristics prior to the start of the intervention, and that 'unobservables' follow a common trend; under such circumstances, any differences in changes observed over time between the 2 populations are attributable to the interventions[39, 40]. Despite AP and Maharashtra having broadly similar socio-economic profiles, there may have been factors resulting in unobserved differential changes between the 2 populations and to which the results of the DID analysis may be at least partially attributable.

A second limitation could arise from the impact of other public health programmes implemented during the period 2004 to 2012. The most significant of these is the National Rural Health Mission launched in 2005, mainly to improve maternal and child health through the revitalization of rural primary care and child and maternal health services. A key assumption of our study is that the impacts of the NRHM in terms of healthcare expenditure for maternal and child health care would have been similar in both states, as this was a nationwide development. Despite the improvements in the public sector maternal and child health services sought by the NRHM, it is widely recognised

For peer review only

For peer review only - http://bmjopen.bmj.com/site/about/guidelines.xhtml

that the public, including BPL families, continues to pay OOP for private health care. We have assumed that this behaviour is likely to be similar across the 2 states. Other health initiatives such as the Navsanjeevani Yojana and HMRI were unlikely to have had an impact on inpatient care or expenditure. The 108 scheme had the potential, in AP, to influence hospitalisation rates, by helping more households to visit hospitals when seriously ill. But the effect is likely to be small as the majority of even serious illnesses do not result in a 108 call for transport. The implementation of the RSBY and the scheme to make private hospital beds available for the poor in MH may have diluted the DID, although both schemes are known to have been only partially implemented.

Lastly, the 2004 NSSO survey which served as our baseline, was carried out between January and June 2004. Our end-line 2012 survey was carried out over a period of 3 months from June to September. The morbidity and mortality patterns recorded in different time periods may vary, and could have influenced the data.

DISCUSSION

We found that average expenditure, large expenditures and large borrowings on inpatient care had increased in both MH and AP, but the increase was consistently smaller in AP across these 3 outcome measures, and may be suggestive of Aarogyasri having a somewhat larger effect than RSBY. Similar increases in institutional deliveries across the 2 states, and low levels of utilisation of RSBY and Jeevandayee schemes in MH may further strengthen this explanation.

The increase in average OOPE on inpatient care in AP and MH reflects a trend observed nationwide[16, 41]. The Aarogyasri scheme may have contributed to the more favourable trajectory in AP both directly and indirectly, in that the scheme may have contributed to a reduction in the prices of interventions and an increase in competition among health care providers. The evaluation of the Yeshasvini scheme also found a significant reduction in the price of surgical interventions[10]. Our findings may suggest that the positive effects of Aarogyasri detected by other studies[15,16] at an early stage of the roll out of the scheme have been sustained. Automatic enrolment into the scheme, near universality of coverage and no requirement for enrollee contributions may have contributed to the significant DIDs in male headed households, scheduled castes, rural households and the poorest and middle asset quintiles. But these benefits were not demonstrated in some of the most vulnerable groups - female headed households and scheduled tribes. This is consistent with the findings of other studies[5,16] which reported that the slightly less vulnerable may benefit more from such schemes, with non-financial barriers undermining access to services for the most vulnerable socio-economic groups. The case summaries below illustrate the obstacles they face. A similar observation was reported by the evaluation of the Mexican Seguro Popular health scheme which showed that a third of the treatment-cluster households who were automatically affiliated were unaware of this fact[42].

The likely explanation of an Aarogyasri effect may be strengthened by the trend for large borrowings which has also increased over time in both states but less so in AP across all groups of analysis except the richest asset quintile. A multi-country analysis of household catastrophic health expenditure highlighted that increasing the availability of health services is critical to improving health in poor countries, but it could also raise the proportion of households facing catastrophic expenditure unless financial risk protection policies are given a high priority[43]. Borrowing of comparatively small amounts is less impactful and may be a result of improved access to financial markets and also supports consumption smoothening. During recent decades, AP has in particular witnessed a significant rise in microfinance institutions and debt due to high levels of interest levied by the more recent entrants to this market[44, 45]. Nevertheless our results suggest that increases in large borrowings associated with inpatient health care were smaller in AP. Strongly significant DIDs in scheduled tribes, rural households and the poorest and second asset quintiles and moderately

For peer review only - http://bmjopen.bmj.com/site/about/guidelines.xhtml

significant DIDs in female headed households, scheduled castes, and other excluded social groups may also point to Aarogyasri beginning to offer greater equity of access when families are faced with serious illness.

In terms of large OOPE, a significant DID was found only for the total households, but the direction of all DIDs except for the second asset quintile was the same as for average expenditure on inpatient care and large borrowings, i.e., in favour of AP. It is perfectly possible that the non-significant differences for the majority of sub groups are due at least in part to the RSBY, the availability of private sector hospital beds for the poor and the Jeevandayee schemes reducing large OOPE in MH, and that without these schemes the expenditure would have been greater. In AP, 70 percent of survey households reported that they were covered by the Aarogyasri scheme, but only 25 percent of these 'covered' households were aware that the benefit package was limited. It is also possible therefore that in AP some families seek hospital care assuming that the Aarogyasri scheme provides comprehensive cover, but are faced with large expenditures when their treatments fall outside the limits.

The mixed picture in relation to the increasing rate of hospitalisations in both states may suggest that the Aarogyasri and in particular, the RSBY scheme which covers common hospital procedures are addressing a large hitherto unmet need for inpatient care. The 108 scheme in AP may be an additional albeit smaller contributor. It may also be explained by a supplier-induced demand. An assessment of health provider behaviour and governance is an important strand of the evaluation of health financing schemes[46], but was outside the remit of our study. We would however strongly recommend an impact evaluation focusing on health care supply to complement our evidence. The reduction in hospitalisations among scheduled tribes and the poorest asset quintile in AP are of concern and suggest that if the poor are to secure the benefits appropriated by the near poor or more often by the rich[47], the provision of more comprehensive health schemes is essential, which combines the tertiary care focus of Aarogyasri and the secondary care benefits of RSBY with attention paid to minimise barriers such as the widespread influence of illiteracy and lack of awareness, which limit access to even schemes such as Aarogyasri that are apparently highly inclusive and non-discriminatory, as well as distance to facilities. Our case summaries illustrate this. Furthermore, health financing reforms such as the Jamkesmas[48] in Indonesia and the Seguro Popular in Mexico[42], both countries similar to India in terms of population and growing economies, include outpatient and inpatient care, and curative as well as preventive services, suggesting that a more comprehensive service is possible to implement and worthy of consideration.

In summary, health innovations in AP had a greater beneficial effect on hospital inpatient care-related expenditures than innovations in MH. The Aarogyasri scheme is likely to have contributed to these impacts in AP, at least in part. But in both states, OOPE increased over time. Hospitalisations also increased, and while it is generally assumed that in developing countries this is likely to address genuine need, its impact on health status is not known.

IMPLICATIONS FOR POLICY AND PRACTICE

Despite these uncertainties, Aarogyasri is perceived, with some justification across India as a successful scheme, and is being rapidly replicated across the states. Since July 2012, MH too has joined the list of states offering an Aarogyasri-like scheme, the Rajiv Gandhi Jeevandayee Arogya Yojana[49]. This study has highlighted that such schemes may result in some positive outcomes. Although the study was not designed to elicit the specific features of the scheme to which any comparatively greater benefits may be attributable, and which deserve to be replicated in other states, evidence from systematic reviews[3] points to the need for schemes to be more 'comprehensively' designed to maximise their positive impact. Aarogyasri's design in terms of its aims to address both financial and non-financial barriers - being fully state funded, the systematic

For peer review only - http://bmjopen.bmj.com/site/about/guidelines.xhtml

administrative implementation of Aarogyasri across the state so that it is now almost universal, automatic enrolment, allowing access to the scheme via a ration card which most poor families own, the wide spectrum of treatments offered, the large number of health care providers empanelled - is perhaps responsible as a 'comprehensive' package, for the greater impact.

But the study also suggests that equity of access to health care and the reduction of the overall burden of OOPE especially in the most vulnerable sections of the population[50] are likely to require additional interventions that address gaps in the availability of care and provide patients appropriate pathways that support their journey from a strong and comprehensive primary care service where they may be informed of their entitlements and investigated for their initial symptoms to appropriate hospitals for the treatment of serious illness. Besides, inpatient care is only consumed by a small proportion of households in a year[51], and this is especially true of tertiary care, while many more will seek outpatient services and referral to inpatient care, should this be required. Others have strongly recommended the strengthening of the primary care base as an essential means to universal health coverage and this study confirms their view[41]. Key implications for AP are to explore how best the most advantageous features of Aarogyasri can be extended to include both secondary and primary care, while those for MH may be to build on and unify its menu of currently available schemes to create an evidence-based comprehensive health delivery system. These conclusions may be applicable to other states with similar health financing schemes.

The design of health financing systems as well as their evaluations is complex and challenging, as the mountain of available evidence suggests[41, 42, 51, 52]. Even the ground-breaking Seguro Popular health insurance programme of Mexico which used a cluster-randomised trial design with strong government support demonstrated some but not all anticipated outcomes, contrary to expectations[42], and a key recommendation was that continued assessment of the programme was needed. Furthermore, differences in data and methodology may result in even very well designed evaluations of the same programme producing contrasting findings[52]. Our evaluation is the first to use a quasi-experimental methodology - the best possible in the hierarchy of evaluation methodologies, when a randomised control trial is not achievable - to evaluate the health financing reforms in 2 large states of India. Despite that, the study has limitations which we have acknowledged. For example, the evaluation was not designed to assess provider behaviours. Many other pertinent questions such as the impact of the schemes on the overall economy of health care cannot be answered by a single evaluation. But these are recognised problems which can only be addressed through continuous assessments, as other evaluations have shown. Our study has nevertheless produced sufficient insights to enable policy leaders to improve programme effectiveness and, importantly, to undertake further assessment. Our household survey data provide a valuable baseline for future monitoring in both states.

This study needs to be followed up with further and repeated evaluations as AP's and MH's schemes evolve; to assess the impacts of re-design and to help health policy leaders achieve their aspiration of universal access to good quality health care.

Three faces of the Aarogyasri Scheme

The beneficiary:

Lakshmamma, a 65 year old widow, is from a tribal background. She is an unskilled labourer, supporting a family of 5, with a monthly income of INR 4000 (USD 74). She was referred to a municipal hospital with chest pain and underwent heart surgery. The hospital which is a part of the

For peer review only - http://bmjopen.bmj.com/site/about/guidelines.xhtml

Aarogyasri network provided free care and her total out of pocket expenses amounted to INR 850 (16 USD) for initial transport. In addition to the surgery, she received free food, money for transport home and follow up medicines. She is very satisfied with the service she received.

The excluded:

Ramulamma, a mother has a BPL card, but her daughter Lakshmi does not, as she was abandoned by her husband who is a government employee and entitled to free health care. The mother was unable to secure free care using her BPL card for her seriously ill daughter who paid out-of-pocket at a private facility where she was offered a hysterectomy for the relief of her gynaecological symptoms. Lakshmi's health care costs were met by the family selling a number of household assets and Lakshmi's daughter discontinuing her education to take up paid work. Lakshmi is severely depressed and does not speak to anyone.

The uninformed:

Krishnamma 43 years old, had severe stomach pains one night. Although the family had a BPL card, Ramulu her husband and Srinivas her son rushed her to a private hospital nearby, which was not part of the Aarogyasri network. They were unaware of how to access the Aarogyasri scheme hospitals which were further away from home, and the local primary health services being inadequate, Krishnamma had not had her initial symptoms investigated. The treatment was funded through a loan from a private moneylender. On discharge, she has not attended follow up, as the family cannot afford transport or medicines.

For peer review only

For peer review only - http://bmjopen.bmj.com/site/about/guidelines.xhtml

All authors had full access to all the data (including statistical reports and tables) and can take responsibility for the integrity of the data and the accuracy of the data analysis

M Rao affirms that the manuscript is an honest, accurate and transparent account of the study being reported and no important aspects of the study have been omitted.

FUNDED BY : Wellcome Trust, IDRC, Rockefeller Foundation, DFID, World Bank.

ROLE OF THE FUNDING SOURCES: The external funding sources had no role in study design, data collection, analysis, interpretation or reporting, or in submission decision.

ROLE OF THE STUDY SPONSOR: The study was carried out in response to the formal invitation of the Government of Andhra Pradesh which supported our applications for funding (by providing formal letters of support). The Government did not provide any funding, and had no role in study design, data collection, analysis, interpretation or reporting, or in submission decision.

STATEMENT OF INDEPENDENCE OF RESEARCHERS: The authors can confirm that they worked independently from the Funders and Government of Andhra Pradesh who had no role in any aspect of the research

CONFLICT OF INTEREST

All authors have completed the ICMJE forms for disclosure of potential conflicts of interest (available on request from the corresponding author) and declare that 1) MR, AK, PS, AS, and SB have support from the Rockefeller Foundation, Wellcome Trust, International Development Research Centre, Canada and Department for International Development, UK, 2) the authors have no relationships with the funders that might have an interest in the submitted work in the previous 3 years, 3) the authors' spouses, partners, or children have no financial relationships that may be relevant to the submitted work, and 4) the authors have no non-financial interests that may be relevant to the submitted work.

ETHICAL APPROVAL: given by the research ethics committee of the Administrative Staff College of India, Hyderabad, which hosted the study. Household survey questionnaires include signed consent by the head of the household or another adult representative of the household.

DATA SHARING STATEMENT: The 2004 National Sample Survey Organisation of India household survey questionnaire and data are available to the public. The 2012 household survey questionnaire, and the full anonymised household survey dataset will be available with open access as soon as the data analyses and submissions for publication are completed.

COPYRIGHT STATEMENT: Mala Rao, the Corresponding Author has the right to grant on behalf of all authors and does grant on behalf of all authors, an exclusive licence on a worldwide basis to the BMJ Publishing Group Ltd to permit this article (if accepted) to be published in BMJ editions and any other BMJPGL products and sublicences such use and exploit all subsidiary rights, as set out in your licence.

STROBE CHECKLIST FOR CROSS SECTIONAL STUDIES: We can confirm that all 22 items in the STROBE checklist are included in the paper.

For peer review only - http://bmjopen.bmj.com/site/about/guidelines.xhtml

For peer review only

CONTRIBUTORS' STATEMENT

MR conceived and designed the study, applied for funding, and was responsible for the supervision and management of the study as well as the dissemination of its results. She is also the guarantor of the study. SB shared responsibility for the conception of the study, applications for funding, study design and data collation and analysis, contributed to the questionnaire design and commented on drafts of the report. PS contributed to the conception of the study and study design, led the questionnaire design and survey implementation, including training of survey staff, monitoring survey progress and data collation and verification, commented on drafts of the report and helped prepare the references. AK undertook the data collation, verification and analysis, assisted with the survey and questionnaire design and survey implementation and prepared the tables for the report. AS led the literature review, assisted with the study and questionnaire design, survey implementation and preparation and analysis of baseline data, and commented on drafts of the report. MK helped with the data analysis. AW devised the methodology for the estimation of the programme impacts, advised during the data-collection and data-preparation stages, wrote and implemented the computer code for the model estimation, helped to oversee the production of the results, and contributed text to the report. GN provided technical advice on accounting for the complex survey structure in the analysis, developed a STATA equation, helped to compute an asset index, advised on the output tables, verified the analysis and commented on drafts of the report. AR helped develop a conceptual framework for the evaluation, advised on funding proposals, the study design, analytical methodology and presentation of results and contributed text to the report. MR wrote the first draft of the paper and its redrafts in accordance with the comments of all other authors and reviewers.

For peer review only

References

[1] Resolution WHA58.33. Sustainable health financing, universal coverage and social health insurance. In: *Fifty-eighth World Health Assembly, Geneva, 16–25 May 2005.* Geneva, World Health Organization, 2005 (http://apps.who.int/gb/ebwha/pdf_files/WHA58/WHA58_33-en.pdf, accessed 26th March 2013).

[2] World Health Organization. Health Systems Financing Path to Universal Coverage. Geneva: World Health Organization. 2010. Available from: World Health Organization Library Information Services.

[3] Acharya A, Vellakkal S, Taylor F, Masset E, Satija A, Burke M, Ebrahim S. Impact of national health insurance for the poor and the informal sector in low and middle income countries : a systematic review. London: EPPICentre, Social Science Research Unit, Institute of Education, University of London. 2012. Available from: http://www.dfid.gov.uk/r4d/PDF/Outputs/SystematicReviews/Health-insurance-2012Acharya-report.pdf

[4] Giedion, U., B. Y. Diaz . A Review of the evidence. In: The Impact of Health Insurance in Low-and Middle-Income Countries. M. L. Escobar, C. Griffin and R. P. Shaw (ed). Washington DC, Brookings Inst Press 2011.

[5] Giedion, U, Alfonso, E , Diaz. *The impact of universal coverage schemes in the developing world : a review of the existing evidence*. Universal Health Coverage (UNICO) studies series ; no. 25. Washington D.C. : The World Bank. 2013

[6] National Commission on Macroeconomics and Health. Report of the National Commission on Macroeconomics and Health. New Delhi: Ministry of Health and Family Welfare of India. 2005. Available from: Ministry of Health and Family Welfare

[7] Eleventh Five Year Plan (2007–2012). Vol 2: Social Sector. Government of India Planning Commission. October, 2008. http://planningcommission.nic.in/plans/planrel/fiveyr/11th/11_v2/11th_vol2.pdf, (accessed Mar 26 2013).

[8] Faster, Sustainable and More Inclusive Growth. An Approach to the Twelfth Five Year Plan (2012-17). Government of India Planning Commission. October, 2011. http://planningcommission.nic.in/plans/planrel/12appdrft/appraoch_12plan.pdf, accessed 26th March 2013

[9] La Forgia G, Nagpal S. Government-Sponsored Health Insurance in India. *Are You Covered?* World Bank Washington DC 2012. doi:10.1596/978-0-8213-9618-6.

[10] Agarwal A. "Impact evaluation of India's 'Yeshasvini' community based health insurance programme. *Health Economics* 2010; 19: 5-35

[11] See The scheme website at https://www.aarogyasri.org/ASRI/index.jsp, accessed 26th March 2013.

[12] Scheme status. http://www.rsby.gov.in/statewise.aspx?state=35 (accessed Apr 30 2013)

[13] Mahal A. Learning and getting better: Rigorous evaluation of health policy in India. National Medical Journal of India. 2011. 325-27.

[14] Aarogyasri Health Care Trust Annual Report 2011-2012. https://www.aarogyasri.org/ASRI/EXT_IMAGES/documents/Annual_Report_201011.pdf (accessed Mar 26 2013)

[15] Rao M, Ramachandra S, Bandyopadhyay S, et al. Addressing healthcare needs of people living below the poverty line: a rapid assessment of the Andhra Pradesh Health Insurance Scheme. National Medical Journal of India. 2011. 24(6):335-41.

[16] Fan V, Karan A, Mahal A. "State Health Insurance and Out of-Pocket Health Expenditures in Andhra Pradesh, India." CGD Working Paper 298.Washington, D.C.: Center for Global Development. 2012 http://www.cgdev.org/content/publications/detail/1426275 (accessed Mar 15 2013)

[17] Palacios R, Das J, Sun C eds. India's health insurance scheme for the poor: Evidence from the early experience of Rashtriya Swasthya Bima Yojana. New Delhi: Center for Policy Research; 2011

[18] Rashtriya Swasthya Bima Yojana overview and scheme details. http://www.rsby.gov.in (accessed Apr 30 2013)

[19] Rathi P. http://www.priorities2012.com/documents/3d-4-patreek.pdf

[20] NRHM Mission Document.http://www.nird.org.in/brgf/doc/Rural%20HealthMission_Document.pdf (accessed Apr 30 2013)

[21] State HMIS data analysis, April'10-March'11 Andhra Pradesh, prepared by NHSRC.http://cfw.ap.nic.in/nrhm/pdf/AP_Analysis_Apr2010-Mar2011.pdf (accessed May 2 2013)

[22] NRHM achievements in Maharashtra. http://www.nrhm.maharashtra.gov.in/achievements.htm (accessed May 2 2013)

[23] Health information helpline.http://www.hmri.in/oursolutions-healthinformation.html (accessed Apr 30 2013)

For peer review only - http://bmjopen.bmj.com/site/about/guidelines.xhtml

[24] About the project/scheme. http://www.maha-arogya.gov.in/projectandschemes/Jeevandaiaarogya/default.htm (accessed Apr 30 2013)
[25] Jeevandayee scheme performance: Information received from Rajiv Gandhi Jeevandayee Arogya Yojana Society Govt of Maharashtra on May 17 2013
[26] Projects and schemes. http://maha-arogya.gov.in/projectandschemes/itdpnavsanjivani%5Cdefault.htm (accessed Apr 30 2013)
[27] Mallepeddi R , Pernefeldt H, Bergkvist S . Andhra Pradesh health sector reform, A narrative case study, Technical paper No.7 for The Rockefeller Foundation–Sponsored Initiative on the Role of the Private Sector in Health Systems in Developing Countries. 2009 ; pp 42-48
[28]EMRI. http://www.emri.in/states.html (accessed Apr 30 2013)
[29] Developing and evaluating complex interventions: new guidance. Medical Research Council. London 2008.
[30] Cesar G V, Jean-Pierre H, Jennifer B. Evidence-Based Public Health: Moving Beyond Randomized Trials. Am J Public Health. 2004 March; 94(3): 400–405.
[31] Presentation on Annual Plan 2012-13 and Five Year Plan 2012-17. http://planningcommission.nic.in/plans/stateplan/Presentations12_13/maharashtra1213.pdf (accessed on mar 25 2013)
[32] National Sample Survey Organization. Morbidity, Healthcare and condition of the Aged. New Delhi: Ministry of Statistics and Programme Implementation. 2004.
[33] National Sample Survey Organization. Household Consumer Expenditure 66th round . New Delhi: Ministry of Statistics and Programme Implementation; 2009-10
[34] National Sample Survey Organization. Household Consumer Expenditure in India. New Delhi: Ministry of Statistics and Programme Implementation; 2005.
[35] Booysen F, Van der Berg S & Burger R. Using an Asset Index to Assess Trends in Poverty in Seven Sub-Saharan African Countries. World Development. 2008; 36(6): 1113–1130
36 Vyas S. & Kumarnayake L. Constructing socio-economic status indices: How to use principal components analysis. Health Policy and planning. 2006; 459-468
[37] O'Donnell O, Van Doorslaer E, Wagstaff A & Lindelow M, 2008. Analyzing Health Equity Using Household Survey Data: A Guide to Techniques and Their Implementation. Washington DC: World Bank Institute. Chapter 6, Measurement of Living Standards. http://siteresources.worldbank.org/INTPAH/Resources/Publications/459843-1195594469249/HealthEquityFINAL.pdf (accessed May 9 2013)
[38] Ministry of Finance. Economic Survey 2011-12. New Delhi: Government of India. 2012. Available from: http://indiabudget.nic.in/
[39] Poverty Reduction and Social Development Department. A User's Guide to Poverty and Social Impact Analysis. Washington: World Bank;2003
[40] Gertler P, Martinez S, Premand P, Rawlings L, Vermeersch C. Impact Evaluation in Practice. Washington: World Bank; 2011.
[41] Selvaraj S, Karan A. Why Publicly-Financed Health Insurance Schemes Are Ineffective in Providing Financial Risk Protection. Economic and Political Weekly 2012 March 17; XLVII(11):60-68
[42] King G, Gakidou E, Imai K, Lakin J, Moore RT, Nall C. Public policy for the poor? A randomised assessment of the Mexican universal health programme. Lancet. 2009 Apr 8; DOI:10.1016/S0140-6736(09)60239-7.
[43] Xu K, Evans DB, Kawabata K, Zeramdini R, Klavus J, Murray CJ. Household catastrophic health expenditure: a multicountry analysis. Lancet. 2003 Jul 12;362(9378):111-7.
[44] Naga Sridhar G. Microfinance institutions: Moving beyond AP crisis. The Business Line [Newspaper Online]. 2012 Dec 29. Available from: http://www.thehindubusinessline.com/companies/microfinance-institutions-moving-beyond-ap-crisis/article4253239.ece
[45] Naga Sridhar G. Life after microfinance in AP. The Business Line [Newspaper Online]. 2013 Jan 4. Available from: http://www.thehindubusinessline.com/opinion/life-after-microfinance-in-ap/article4273183.ece
[46] Prasad N P, Raghavendra P. Healthcare models in the era of medical neoliberalism. A study of Aarogyasri in Andhra Pradesh. Economic and Political Weekly 2012 Oct 27;XLVII(43):118-126
[47] Health Systems 20-20. An Evaluation of the Effects of the National Health Insurance Scheme in Ghana. Bethesda: Health Systems 20-20. 2009.
[48] http://www.jointlearningnetwork.org/programs/compare/benefits/142%2C16
[49] Rajiv Jeevandayi scheme details. http://www.jeevandayee.gov.in/RGJAY/FrontServlet?requestType=CommonRH&actionVal=RightFrame&page=

For peer review only - http://bmjopen.bmj.com/site/about/guidelines.xhtml

undefined%3E%3E%3Cb%3ERGJAY%3C/b%3E&pageName=RGJAY&mainMenu=About&subMenu=RGJAY (accessed on Apr 30 2013)

[50] Tangcharoensathien V, Swasdiworn W, Jongudomsuk P et al . Universal Coverage Scheme in Thailand: Equity Outcomes and Future Agendas to Meet Challenges. Geneva: World Health Organization. 2010.

[51] Dilip TR. On Publicy-Financed Health Insurance Schemes. Is the analysis premature? Economic and Political Weekly. 2012 May 5; XLVII(1)8:79-80.

[52] Vallakal S, Ebrahim S. Publicly-Financed Health Insurance Schemes. Concerns about Impact Assessment. Economic and Political Weekly. 2013 Jan 5; XLVIII(1);24-27.

For peer review only

For peer review only - http://bmjopen.bmj.com/site/about/guidelines.xhtml

Report No. 507 (60/25.0/1)

Morbidity, Health Care and the Condition of the Aged

For peer review only

NSS 60th round (January – June 2004)

National Sample Survey Organisation
Ministry of Statistics and Programme Implementation
Government of India

March 2006

Preface

The National Sample Survey Organisation (NSSO) has been collecting information on various facets of the Indian economy through nationwide sample surveys to assist in socio-economic planning and policy-making. The National Sample Survey (NSS) made its first attempt to collect information on morbidity in the 7th round (October 1953 - March 1954). This maiden attempt and the subsequent three surveys on the subject from the NSS 11th to 13th rounds (1956-58) were exploratory in nature and aimed to evolve an appropriate data collection method for studying morbidity profile in India. However, a full-scale survey on morbidity was conducted for the first time during the NSS 28th round (October 1973 - June 1974). Since then, the NSSO had not undertaken any separate survey on morbidity and the collection of data on morbidity became a part of the decennial surveys on social consumption carried out in the NSS 35th round (July 1980 - June 1981), 42nd round (July 1986 - June 1987) and 52nd round (July 1995 - June 1996).

A survey on 'Morbidity and Health care' was undertaken by the NSSO during the NSS 60th round (January - June 2004) at the request of Ministry of Health and Family Welfare. This subject was taken up along with the subjects of household consumer expenditure and employment-unemployment. The present report is based on the enquiry on 'Morbidity and Health Care' conducted during the NSS 60th round. The enquiry covered the curative aspects of the general health care system in India, utilization of health care services provided by the public and private sector and the expenditure incurred by the households for availing these services. In addition, information on the condition and problems of the aged persons was also collected.

The report consists of five chapters and three appendices. Chapter One deals with the introduction and Chapter Two with concepts and definitions that have been used in the survey. Chapters Three, Four and Five discuss the summary results of the survey and their comparison with the results of the previous surveys. While Chapter Three discusses the survey estimates relating to Morbidity and Hospitalisation, Chapter Four deals with estimates on Immunisation and Maternal Health Care. In Chapter Five, the survey results on Conditions and Health Care of the Aged have been discussed**.**

The Survey Design and Research Division of NSSO was responsible for development of survey methodology as well as drafting of the report. The fieldwork for the survey was handled by the Field Operations Division of NSSO. While the data processing and tabulation work was handled by the Data Processing Division of NSSO, the Coordination and Publication Division of NSSO coordinated various activities pertaining to the survey.

I am thankful to the Chairman and the Members of the Working Group for the NSS 60th round as well as to the Chairman and the Members of the Governing Council of NSSO for their overall guidance at various stages of survey work. The report, I hope, will be useful to the planners and policy makers.

Comments and suggestions from readers will be most welcome.

New Delhi
March 2006

Dr. K.V. Rao
Director General and Chief Executive Officer
National Sample Survey Organisation
Ministry of Statistics and Programme Implementation

For peer review only

Highlights

A. Morbidity and health care

		Rural	Urban
**Proportion of Ailing Persons by Residence Status & Sex**			
**Number (per 1000) of persons reporting ailment (PAP) during a period of 15 days**	Male	83	91
	Female	93	108
	Person	88	99
**Level of Morbidity in broad age-groups**			
**PAP during a period of 15 days in broad age-groups**			
	0-14	72	79
	15-29	49	50
	30-44	78	79
	45-59	119	149
	60 or more	283	368
**Inter-state Variation in Level of Morbidity**			
**States with relatively high PAP during a period of 15 days**			
	Kerala	255	240
	Punjab	136	107
	West Bengal	114	157
	Maharashtra	93	118
**States with relatively low PAP during a period of 15 days**			
	Jharkhand	33	50
	Bihar	53	63
	Uttaranchal	52	65
	Rajasthan	57	72
**Commencement of Ailment**			
**Number (per 1000) of persons reporting commencement of any ailment (PPC) during last 15 days in broad age-groups**			
	0-14	54	59
	15-29	29	32
	30-44	37	38
	45-59	46	40
	60 or more	68	55
	all ages	45	44
**Treatment of Ailments**			
**Percentage of spells of ailments treated during 15 days**			
	Male	82	90
	Female	82	89
	Person	82	89
**Reason for No Treatment**			
**Percentage distribution of untreated spells of ailments by reason for no treatment**			
	No medical facility	12	1
	Lack of faith	3	2
	Long waiting	1	2
	Financial problem	28	20
	Ailment not considered serious	32	50
	Others	24	25
	Total	100	100

Continued

For peer review only - http://bmjopen.bmj.com/site/about/guidelines.xhtml

Highlights

	Rural	Urban
**Sources of Non-hospitalised Treatment**		
**Percentage distribution of non-hospitalised treatments by source of treatment**		
Govt. sources	22	19
Private sources	78	81
Total	100	100
**Hospitalised Treatment**		
**Number (per 1000) of persons hospitalised any time during a period of 365 days**		
Male	23	31
Female	22	31
Person	23	31
**Level of Hospitalisation in broad age-groups**		
**Number (per 1000) of persons hospitalised any time during a period of 365 days in broad age-groups**		
0-14	12	20
15-29	19	21
30-44	25	29
45-59	39	48
60 or more	56	91
**Percentage distribution of hospitalised treatments by type of hospital**		
Govt. hospitals	42	38
Private hospitals	58	62
all hospitals	100	100
**Average duration of stay (0.0 days) by type of hospital**		
Govt. hospitals	10.9	10.8
Private hospitals	8.3	7.3
**Cost of Treatment**		
**Average medical expenditure (Rs.) for non-hospitalised treatment per ailing person during a period of 15 days**		
Male	275	322
Female	240	291
Person	257	306
**Average total expenditure (Rs.) for non-hospitalised treatment per ailing person during a period of 15 days**		
Medical expenditure:		
Govt. sources	11	7
Private sources	246	299
Other expenditure	27	20
Total	285	326
**Average medical expenditure (Rs.) per hospitalisation during a period of 365 days**		
Male	5,946	9,535
Female	5,406	8,112
Person	5,695	8,851

Continued

For peer review only - http://bmjopen.bmj.com/site/about/guidelines.xhtml

Highlights

	Rural	Urban
**Average total expenditure (Rs.) for hospitalised treatment per hospitalisation case during a period of 365 days**		
Medical expenditure:		
Govt. sources	3,238	3,877
Private sources	7,408	11,553
Other expenditure	530	516
Total	6,225	9,367

B. Immunisation of children aged 0 –4 years

	Rural	Urban
**Number (per 1000) of children of age 0 – 4 years receiving any immunisation during a period of 365 days**		
Boys	893	934
Girls	892	937
Children	892	936
**Average expenditure incurred (Rs. 0.0) on immunisation during a period of 365 days**		
Boys	22.3	109.2
Girls	17.4	118.3
Children	19.9	113.4

C. Pregnancy, Childbirth & Maternity Care

	Rural	Urban
**Number (per 1000) of women of age 15 – 49 years pregnant any time during a period of 365 days**	127	107
**Number (per 1000) of pregnant women of age 15 – 49 years who delivered a child**	716	694
**Percentage distribution of women who delivered a child during a period of 365 days by place of delivery**		
Government hospital	183	310
Private hospital	166	429
Home	651	261
**Average expenditure per childbirth during a period of 365 days by place of delivery**		
Government hospital	1,165	994
Private hospital	4,137	5,480
Home	414	552
all	1,169	2,806
**Number (per 1000) of pregnant women of age 15 – 49 years who availed of maternal care service**		
Antenatal care services	698	836
Post-natal care services	626	729
**Average expenditure (Rs.) on maternal care service**		
Antenatal care services	499	905
Post-natal care services	402	595

Continued

For peer review only

For peer review only - http://bmjopen.bmj.com/site/about/guidelines.xhtml

Highlights

D. Condition of the Aged (60 yrs. or more)

	Rural	Urban
**Number (per 1000) of aged in the population**		
Male	70	62
Female	71	71
Person	70	66
**Old-age dependency ratio (aged per 1000 of persons of age 15-59 yrs.)**	125	103
**Sex-ratio among the aged**	985	1046
**Percentage distribution of the aged by their living arrangement**		
Living alone	5	4
With spouse	58	58
Children	33	33
Others	4	5
Total	100	100
**Percentage of the aged depending on others for day-to-day maintenance**	67	64
**Category of persons supporting the dependent aged**		
Spouse	13	15
Own Children	78	76
Grand children	3	3
Others	6	6
Total	100	100
**Number (per 1000) of aged persons who cannot move and are confined to bed or home**		
Male	67	68
Female	88	100
Person	77	84

For peer review only

For peer review only - http://bmjopen.bmj.com/site/about/guidelines.xhtml

Contents

		Page No.
**Chapter One**	**Introduction**	**1 – 4**
**Chapter Two**	**Concepts and Definitions**	**5 – 8**
**Chapter Three**	**Morbidity and Hospitalisation**	**9 – 43**
**Chapter Four**	**Immunisation and Maternity Health Care**	**44 – 53**
**Chapter Five**	**Condition and Health Care of the Aged**	**54 – 65**
**Appendix A**	**Detailed tables**	**A-1 – A-380**
**Appendix B**	**Coverage, Sample Design and Estimation Procedure**	**B-1 – B-11**
**Appendix C**	**Schedule 25.0: Morbidity and Health Care**	**C-1 – C-10**

Detailed tables

table no.	title	page no.
	**Part A1: Morbidity and Hospitalisation**	
1	Number of villages/blocks, households, persons, aged (60 years and above) persons surveyed, number of hospitalised and ailing persons surveyed and average household size, separately for each State/UT	A-1
2	Per 1000 distribution of households by type of structure for (i) each monthly per capita expenditure (mpce) class, (ii) each social-group and (iii) each household type	A-7
3	Per 1000 distribution of households by major source of drinking water for (i) each mpce class, (ii) each social group, and (iii) each household type	A-10
4	Proportion of households treating water before drinking and per 1000 distribution of such households by type of water treatment for each major source of drinking water	A-13
5	Number per 1000 households treating water before drinking and per 1000 distribution of such households by type of water treatment for each mpce class	A-15
6	Per 1000 distribution of households by primary source of energy for cooking for each MPCE class	A-17
7	Number per 1000 of households having pet animals and per 1000 distribution of such households by mpce class for each State/UT	A-20

Continued

For peer review only - http://bmjopen.bmj.com/site/about/guidelines.xhtml

table no.	title	page no.
8	Per 1000 distribution of persons by age-group for each sex	A-26
9	Per 1000 distribution of persons of age 5 years and above by general educational level for each age-group and sex	A-31
19	Number of hospitalisation cases during last 365 days and number of cases among these where any medical services were provided by the employer by source of treatment for each age-group	A-40
20	Rate of hospitalisation (number per 1,00,000) during last 365 days by sex and broad age-group	A-43
21	Rate of hospitalised persons (number per 1,00,000) during last 365 days by sex and broad age-group	A-45
22	Per 1000 distribution of hospitalised cases during last 365 days by nature of ailment for different age-groups and sex	A-47
23	Per 1000 distribution of hospitalisation cases by type of hospital and type of ward for each mpce class and sex	A-65
24	Per 1000 distribution of hospitalised cases receiving treatment during last 365 days by time when admitted into hospital and by time when discharged from hospital for each type of hospital and sex of patient	A-74
25	Per 1000 distribution of hospitalisation cases by duration of stay in hospital and average duration of stay in hospital (in 0.0 days) for each type of hospital	A-77
26	Per 1000 distribution of hospitalisation cases by duration of stay in hospital and average duration of stay in hospital separately for government and private hospitals and for each mpce class	A-80
27	Number per 1000 cases of hospitalisation receiving specific types of medical service and their break-up by payment category for different types of hospital	A-87
28	Number per 1000 of hospitalisation cases receiving treatment before hospitalisation and per 1000 distribution of such cases by source of treatment availed of for each type of hospital where admitted and sex	A-92
29	Number per 1000 cases of hospitalisation that received treatment after discharge from hospital and per 1000 distribution of such cases by source of treatment availed of for each type of hospital where admitted and sex	A-95
30	Average total medical expenditure (Rs.) for treatment per hospitalisation case during the stay at hospital (as inpatient) for last 365 days by type of hospital (Govt./Pvt), mpce class and sex	A-98
31	Average expenditure (Rs.) per hospitalisation case on account of hospitalisation for each mpce class and sex	A-101
32	Average total expenditure on account of hospitalisation per household and per 1000 distribution of total household expenditure on account of hospitalisation by source of finance for each mpce class	A-106
33	Average medical expenditure (Rs.) for treatment under different heads of treatment during stay at hospitals as inpatient during last 365 days per hospitalisation case receiving medical treatment for each type of hospital, mpce class of household	A-109
34	Number per 1000 of hospitalised cases getting reimbursement, average amount of reimbursement and per 1000 distribution of such cases by source for mpce class of households	A-124

Continued

For peer review only - http://bmjopen.bmj.com/site/about/guidelines.xhtml

table no.	title	page no.
35	Number per 1000 of persons reporting ailment during the last 15 days by sex and age-group for each State/UT	A-127
36	Number per 1000 of persons reporting ailment during the last 15 days by mpce class and social group for each sex and State/UT	A-133
37	Number per 1000 of persons suffering from ailment on the day before the date of survey by sex and age-group	A-151
38	Number of persons per 1,00,000 reporting onset of ailments during last 15 days and average duration of ailment by sex and age-group	A-157
39	Number per 1,00,000 of persons reporting onset of specific ailments during last 15 days and average duration of ailment by broad ailment type	A-160
40	Average number of spells of ailment during last 15 days, average time of indisposition during last 15 days, average total duration of ailment and average loss of household income due to ailment during last 15 days for each age-group	A-166
41	Number per 1000 of spells of ailment during last 15 days treated on medical advice and per 1000 distribution of untreated spells of ailments not given medical treatment by reason for not taking treatment for each mpce class and social-group	A-169
42	Number of spells of ailments receiving treatment from non- government sources per 1000 of treated spells during last 15 days and their distribution by reason for not taking treatment from govt. sources for each mpce class	A-178
43	Number per 1000 untreated spells of ailment reporting measures taken for recovery/relief (other than medical treatment), per 1000 distribution of such spells of ailments by measure taken for recovery/relief and average expenditure incurred for such measures for recovery/relief separately for each mpce class	A-187
44	Average medical expenditure (Rs.) for treatment (other than as inpatient) under different heads per ailing person medically treated during last 15 days by mpce class	A-196
45	Average total expenditure incurred during the last 15 days on account of treatment per ailing person (but not as inpatient of a hospital) for each sex and mpce class	A-205
46	Per 1000 distribution of ailing persons among those who were medically treated but not as inpatient of hospital during last 15 days by type of payment made for availing some services for each category of specific medical service for each State/UT	A-210
47	Per 1000 distribution of total household expenditure incurred during the last 15 days in connection with treatment (other than as inpatient of hospital) by source of finance for each mpce class and social group	A-222
48	Number per 1000 of treated persons getting reimbursement for treatment and per 1000 distribution of total amount of reimbursement by source of reimbursement for each sex, mpce class and social-group	A-225

Continued

For peer review only - http://bmjopen.bmj.com/site/about/guidelines.xhtml

table no.	title	page no.
	**Part A2: Immunisation and Maternal Health**	
49	Number per 1000 of children (0 – 4 years) receiving any type of immunisation and average expenditure incurred by sex for each mpce class and social group	A-231
50	Per 1000 number of ever married women aged 15 - 49 yrs. who were pregnant any time during last 365 days, their distribution by status of pregnancy and place of childbirth, and average expenditure incurred per case of childbirth, separately for each age-group, mpce class and social group	A-234
51	(i) Per 1000 distribution of ever married pregnant women (15 - 49 yrs.) during last 365 days, by source of receiving pre-natal care, (ii) per 1000 distribution of ever married pregnant women (15 - 49 yrs.) who gave child birth by source of receiving post-natal care, and (iii) average expenditure on pre-natal care and post-natal care by type of institution for each age-group	A-240
	**Part A3: Condition and Health Care of the Aged**	
10	Per 1000 distribution of aged persons by number of living children for each sex and State/UT	A-243
11	Per 1000 distribution of aged persons by state of economic independence for each sex	A-261
12	Per 1000 distribution of fully economically independent aged persons by number of dependants for each sex and State/UT	A-270
13	Per 1000 distribution of economically dependent aged persons by category of persons financially supporting the aged person for each sex	A-288
14	Per 1000 distribution of aged persons by type of living arrangement for each sex and State/UT	A-297
15	Per 1000 distribution of aged persons living alone or living with spouse only by location of residence of any child/ grandchild or sibling for each sex	A-315
16	Per 1000 distribution of aged persons by state of physical mobility for each age–group and sex	A-324
17.1	Per 1000 distribution of aged persons with illness or otherwise by their perception about current state of health for each mpce class and sex	A-327
17.2	Per 1000 distribution of aged persons with illness or otherwise by their perception about current state of health for each sex	A-336
18.1	Per 1000 distribution of aged persons with illness or otherwise by their perception about change in state of health for each quintile class and sex	A-354
18.2	Per 1000 distribution of aged persons with illness or otherwise by their perception about change in state of health for each sex	A-363

For peer review only - http://bmjopen.bmj.com/site/about/guidelines.xhtml

Chapter One

Introduction

1.1.1 The National Sample Survey Organisation (NSSO) was set up in 1950 as a permanent survey organisation to collect data on various facets of the Indian economy through nation-wide sample surveys in order to assist in socio-economic planning and policy-making. The National Sample Survey made its first attempt to collect information on morbidity in the seventh round (Oct. 1953 - March 1954). This survey and the morbidity surveys conducted in the three subsequent rounds (the eleventh to the thirteenth, 1956-58) were all exploratory in nature. The aim of these surveys was to evolve an appropriate data collection method for studying morbidity profile in India. These surveys were followed up by a pilot survey in the seventeenth round (Sept. 1961 - July 1962) to examine alternative approaches of morbidity reporting. With the aid of the findings of these exploratory surveys, a full-scale survey on morbidity was conducted in the twenty-eighth round (Oct. 1973 - June 1974). Since then, the NSSO had not undertaken any separate morbidity survey and data on morbidity became a part of the decennial surveys on social consumption.

1.1.2 The NSSO carried out the first all-India Survey on Social Consumption in its 35th round (July 1980 - June 1981). The items covered were the public distribution system, health services including mass immunisation and family welfare programmes, and educational services. The results of the survey could not be brought out owing to some unavoidable reasons. The second survey on Social Consumption was carried out in the 42nd round (July 1986 - June 1987) with some modifications in the coverage of subjects. Topics like Problems of Aged Persons were included in this round. The third Survey on Social Consumption was carried out in the 52nd round (July 1995 - June 1996). Two topics, viz. utilisation of the public distribution system and utilisation of family planning services, were dropped, as these were covered in the NSS 50th round and in a nationwide survey by the Ministry of Health and Family Welfare, respectively.

1.1.3 After a gap of about nine years, the Governing Council decided to take up a survey on 'Morbidity and Health care' at the request of Ministry of Health and Family Welfare, during the period January to June, 2004.

1.2 The Present Report

1.2.1 The present report is based on the enquiry on morbidity and health care conducted in the 60th round (January - June 2004) of the NSSO. The enquiry covered the curative aspects of the general health care system in India and also the utilization of health care services provided by the public and private sector, together with the expenditure incurred by the households for availing these services.

This report presents the survey results relating to all these aspects viz., the utilisation of the curative health care services, morbidity profile of the population, hospitalised and non-hospitalised treatment of ailments together with the estimates of expenditure incurred for treatment of ailments. In addition, results on problems of the aged persons are also provided separately in the report.

For peer review only

1.2.2 Layout of the report**:** The report contains five chapters and three appendices. Apart from this chapter, Chapter Two provides the concepts and definitions used for the purpose of survey on morbidity and health care. Chapters three, four and five discuss the summary results of the survey and attempts to provide a perspective for proper interpretation of the results. While Chapter Three discusses the survey estimates relating to Morbidity and Hospitalisation, Chapter Four deals with estimates on Immunisation and Maternal Health Care. In Chapter Five, the survey results on Conditions and Health Care of the Aged have been discussed. The detailed survey results in the form of tables are given for rural and urban India in Appendix A, which is sub-divided into three parts, viz. Part A1, Part A2 and Part A3, giving, respectively, the tables relating to and discussed in Chapters Three, Four and Five. While the sample design and estimation procedure have been outlined in Appendix B, the facsimile of the schedule is given in Appendix C.

1.2.3 The estimates presented in the report are based on the data from the *Central sample only* (pooled data of the two *sub-samples*). The survey results presented in the report are mostly in the form of ratios. The basic aggregate estimates of population and number of households, given in the detailed tables of the Appendix, generally as marginal column totals, may be used for working out rates and ratios for domains not presented in the report.

1.3 Sixtieth Round

1.3.1 In the Sixtieth round of NSS, data was collected through a survey on the subject of 'Morbidity and Health Care'. Apart from this subject, surveys were also undertaken separately on 'Household Consumer Expenditure' and 'Employment and Unemployment'. In the survey on Morbidity and Health Care, the following main aspects were covered:

(i) Morbidity and utilisation of health care services including immunisation and maternity care,
(ii) Problems of aged persons, and
(iii) Expenditure of the households for availing the health care services.

1.3.2 The object of the present survey was essentially to study the benefits derived by various sections of the population from investments and outlays made by the Government, as well as by the private sector in the fields of health and get an estimate of expenditure incurred by households to avail health care services including immunization and maternity care.

1.4 Geographical coverage

1.4.1 The entire area of the country was covered with the exception of some interior areas of Nagaland and Andaman & Nicobar Islands, and Leh (Ladakh) and Kargil districts of Jammu & Kashmir.

1.5 Method of Data Collection

1.5.1 The present report is based on the information on morbidity and health care services collected in Schedule 25.0. The data were collected from a sample of households by the interview method. A set of probing questions was put to as many individual members of a selected household as possible to ascertain whether they had suffered from any ailment during the reference period and whether they had taken any medical treatment for it. As far as possible, efforts were made to collect information relating to ailments of each household member from the member themselves. But in spite of the best efforts, some other person of the household might have provided this information, especially for the children and the aged

For peer review only - http://bmjopen.bmj.com/site/about/guidelines.xhtml

persons in the household. Efforts were made to interview all the adult male members of each sample household, personally. For the children, particularly the young, attempts were made to get the required information from their mothers.

1.6 **Reference period**

1.6.1 The enquiry on morbidity was conducted with a reference period of 15 days. All spells of ailment suffered by each member, both present as well as the deceased, of the sample household, during the 15 days preceding the date of enquiry, whether or not the patient was hospitalised for treatment, were covered in the survey. For hospitalised treatment, however, information was collected for every event of hospitalisation of a member, whether living or deceased at the time of survey, during the 365 days preceding the date of enquiry.

1.7 **Sample design**

1.7.1 The sample design adopted for the survey was essentially a two-stage stratified design, with census villages and urban blocks as the first-stage units (FSUs) for the rural and urban areas respectively, and households as the second-stage units (SSUs). The survey period, January - June 2004, was split up into two sub-rounds of three months each. The rural and urban samples of FSUs were drawn independently in the form of two sub-samples and equal numbers of FSUs of each sub-sample were allocated for the two sub-rounds. (For a detailed discussion on sample design, see Appendix B.)

1.7.2 *Sample size -- first-stage units*: In all, 10,072 villages were planned to be surveyed in this round. Of these, 4,908 were allocated to the central sample -- the part surveyed mainly by the NSSO field staff -- and the rest to the State sample -- the part surveyed by the State agencies. In the urban sector, the allocations for the Central and State samples were, respectively, 2,708 and 3,096 blocks. This report is based on the estimates obtained from the central sample alone. The number of villages and that of urban blocks actually surveyed as the central sample were 4,755 and 2,668 respectively.

1.7.3 *Sample size -- second-stage units*: For Schedule 25.0, 10 households were planned to be surveyed in each selected FSU. In the Central sample, the actual numbers of households surveyed in the rural and urban areas were 47,302 and 26,566, respectively. The number of villages and blocks allotted and surveyed , and the number of households surveyed in the rural and urban sectors of each State/UT are given in Statement 1.

For peer review only

For peer review only - http://bmjopen.bmj.com/site/about/guidelines.xhtml

Statement 1: Number of sample villages/blocks allotted and surveyed, and the households surveyed in the central sample for NSS 60th round survey

State /UT	villages / blocks				households surveyed	
	allotted		surveyed			
	rural	urban	rural	urban	rural	urban
(1)	(2)	(3)	(4)	(5)	(6)	(7)
Andhra Pradesh	328	184	325	183	3235	1824
Arunachal Pradesh	76	32	75	32	732	320
Assam	220	48	215	48	2150	480
Bihar	360	64	354	64	3536	638
Chhattisgarh	112	40	107	40	1070	400
Delhi	8	100	8	100	78	961
Goa	8	12	8	12	79	120
Gujarat	152	132	150	131	1497	1309
Haryana	88	56	86	55	851	549
Himachal Pradesh	132	20	126	20	1239	200
Jammu & Kashmir	136	68	83	38	821	380
Jharkhand	144	60	141	60	1398	598
Karnataka	192	152	185	152	1847	1518
Kerala	200	100	184	100	1839	990
Madhya Pradesh	232	128	229	128	2281	1280
Maharashtra	272	272	265	267	2650	2664
Manipur	108	52	107	52	1070	520
Meghalaya	56	24	56	24	536	239
Mizoram	44	64	41	64	394	631
Nagaland	24	12	24	12	240	120
Orissa	212	56	211	56	2094	560
Punjab	84	68	82	68	816	676
Rajasthan	236	108	234	108	2311	1072
Sikkim	44	8	44	8	440	80
Tamil Nadu	260	260	254	260	2540	2599
Tripura	84	24	82	24	820	240
Uttaranchal	36	20	36	20	346	200
Uttar Pradesh	680	264	671	264	6682	2627
West Bengal	324	188	317	188	3170	1879
Andaman & Nicobar Islands	16	12	15	12	140	120
Chandigarh	8	36	8	34	80	333
Dadra & Nagar Haveli	8	8	8	8	80	80
Daman & Diu	8	8	8	8	80	79
Lakshadweep	8	8	8	8	80	80
Pondicherry	8	20	8	20	80	200
**All India**	4,908	2,708	4,755	2,668	47,302	26,566

For peer review only - http://bmjopen.bmj.com/site/about/guidelines.xhtml

Chapter Two

Concepts and Definitions

2.1.1 The concepts and definitions of the terms used in this report are briefly discussed in this chapter. For better morbidity reporting, some probing questions were put to the informants during data collection. Such special attempts to elicit information on morbidity and treatment of ailments are also indicated in this chapter.

2.2 **Household**: A group of persons normally living together and taking food from a common kitchen constitutes a household. The word "normally" means that temporary visitors are excluded but temporary stay-aways are included. Thus a son or daughter residing in a hostel for studies is excluded from the household of his/her parents, but a resident employee or resident domestic servant or paying guest (but not just a tenant in the house) is included in the employer/host's household. "Living together" is usually given more importance than "sharing food from a common kitchen" in drawing the boundaries of a household in case the two criteria are in conflict; however, in the special case of a person taking food with his family but sleeping elsewhere (say, in a shop or a different house) due to space shortage, the household formed by such a person's family members is taken to include the person also. Each inmate of a mess, hotel, boarding lodging house, hostel, etc., is considered as a single-member household but a family living in a hotel is considered to be one household only; the same applies to residential staff of such establishments.

For peer review only

2.3.1 **Ailment - illness or injury**: Ailment, i.e. illness or injury, mean any deviation from the state of physical and mental well-being. An ailment may not cause any necessity of hospitalisation, confinement to bed or restricted activity. An ailing member is a normal member of the household who was suffering from any ailment during the reference period. For the purpose of survey, one will be treated as sick if one feels sick**.** This will also include among other things:

- Cases of visual, hearing, speech, locomotor and mental disabilities;
- Injuries will cover all types of damages, such as cuts, wounds, haemorrhage, fractures and burns caused by an accident, including bites to any part of the body;
- Cases of spontaneous abortion - natural or accidental;

This will not include:

- Cases of sterilisation, insertion of IUD, getting MTP, etc.,
- Cases of pregnancy and childbirth.

Cases of sterilisation, insertion of IUD, getting MTP, etc., under family planning programme, pregnancy and child birth are not treated as ailment. But a spontaneous abortion, is treated as a deviation from the state of normal health and thus considered to be illness.

2.3.2 For ascertaining whether an individual had suffered from any ailment during the reference period and whether she/he had received any medical treatment on that account, the following set of probing questions was put, in the survey, to the informant:

For peer review only - http://bmjopen.bmj.com/site/about/guidelines.xhtml

- During the reference period, did the member feel anything wrong relating to skin, head, eyes, ears, nose, throat, arms, hands, chest, heart, stomach, liver, kidney, legs, feet or any other organ of the body?
- Does the member suffer from any disease of a chronic nature relating to stomach, lungs, nervous system, circulation system, bones and joints, eye, ear, mouth or any other organ of the body?
- Does the member have any kind of hearing, visual, speech or locomotor disability?
- Did the member take, during the reference period, any medicine or medical advice for his/her own ailment or injury?

2.4 **Hospitalisation:** One was considered hospitalised if one had availed of medical services as an indoor patient in any hospital. Hospital, for the purpose of survey, referred to any medical institution having provision for admission of sick persons as indoor patients (inpatients) for treatment. Hospitals covered public hospitals, community health centres and primary health centres (if provided with beds), ESI hospitals, private hospitals, nursing homes, etc. In this context it may be noted that admission for treatment of ailment and discharge thereof from the hospital was considered as case of hospitalisation irrespective of the duration of stay in the hospital. It may also be noted that hospitalisation in the cases of normal pregnancy and childbirth were treated as hospitalisation cases.

2. 5 **Confinement to bed**: It referred to a state of health where the ailing person is required or compelled to mostly stay in bed at his/her residence/home.

2.6 **Restriction of activity:** By disability or restricted activity it was meant the state of health which prevents the ailing person from doing any of his/her normal avocation. For economically employed persons, restricted activity meant abstention from the economic activity. In the case of a housewife, this meant cutting down of the day's chores. In the case of retired persons, this referred to the pruning of his/her normal activity. In the case of students attending educational institution, this referred to abstention from attending classes. For infants below school going age and for the very old, restricted activity was not to be considered in view of the fact that their usual activities are of restricted nature.

2.7 **Spell of ailment**: A continuous period of sickness owing to a specific ailment will be treated as a spell of ailment.

2.8 **Duration of ailment**: Duration of ailment is the period between the commencement of the ailment and termination of it by recovery. For ascertaining the period of ailment during the reference period, commencement was taken as on the first day of the reference period if it was on a day beyond the reference period. Similarly, if the ailment was found to be continuing on the date of enquiry, the day of termination of the ailment was taken as the last day of the reference period.

2.9 **Medical treatment:** A person was considered to have received medical treatment if he/she had consulted a doctor anywhere (in OPD of a hospital, community health centre, primary health centre/sub-centre, dispensary, doctor's chamber, private residence, etc.) and obtained medical advice on his/her ailment. The doctor consulted may follow any system of medicine, viz. allopathic, homeopathic, ayurvedic, unani, hakimi or some other recognised system. Treatment taken on the basis of medical advice/prescription of a doctor obtained earlier for similar ailment(s) was also considered as medical treatment. Self-doctoring or

For peer review only - http://bmjopen.bmj.com/site/about/guidelines.xhtml

acting on the advice of non-medical persons such as friends, relatives, pharmacists, etc., was not considered as treatment.

2.10 **Expenditure for medical treatment**: Total expenditure incurred for medical treatment received during the reference period (15 days for non-hospitalised treatment and 365 days for hospitalised treatment) includedexpenditure on items like

bed charges (with charges for food included in it)
medicines (including drips)
materials for bandage, plaster, etc.
fees for the services of medical and para-medical personnel
charges - for diagnostic tests
operations and therapies
charges of ambulance
costs of oxygen, blood, etc.

All other types of expenditure incurred for treatment, such as lodging charges of escort, attendant charges, cost of transport other than ambulance, and cost of personal medical appliances, were excluded from medical expenditure.

2.11 **Disability**: A person with restrictions or lack of abilities to perform an activity in the manner or within the range considered normal for a human being was treated as having disability. It excluded illness/injury of recent origin (morbidity) resulting into temporary loss of ability to see, hear, speak or move.

2.11.1 **Mental disability:** Persons who had difficulty in understanding routine instructions, who do not carry out their activities like others of similar age or exhibit behaviours like talking to self, laughing / crying, staring, violence, fear and suspicion without reason were considered as mentally disabled for the purpose of the survey. The “activities like others of similar age” included activities of communication (speech), self-care (cleaning of teeth, wearing clothes, taking bath, taking food, personal hygiene, etc.), home living (doing some household chores) and social skills.

2.11.2 **Visual disability:** By visual disability, it was meant, loss or lack of ability to execute tasks requiring adequate visual acuity. For the survey, visually disabled included (a) those who did not have any light perception - both eyes taken together and (b) those who had light perception but could not correctly count fingers of hand (with spectacles/contact lenses if he/she used spectacles/contact lenses) from a distance of 3 metres (or 10 feet) in good day light with both eyes open. Night blindness was not considered as visual disability.

2.11.3 **Hearing disability:** This refers to persons’ inability to hear properly. Hearing disability was to be judged taking into consideration the disability of the better ear. In other words, if one ear of a person was normal and the other ear has total hearing loss, then the person was to be judged as normal in hearing for the purpose of the survey. Hearing disability was judged without taking into consideration the use of hearing aids (i.e., the position for the person when hearing aid was not used). Persons with hearing disability might have different degrees of disability, such as profound, severe or moderate. A person was treated as having ‘profound’ hearing disability if he/she could not hear at all or could only hear loud sounds, such as, thunder or understands only gestures. A person was treated as having ‘severe’ hearing disability if he/she could hear only shouted words or can hear only if

For peer review only - http://bmjopen.bmj.com/site/about/guidelines.xhtml

the speaker is sitting in the front. A person was treated as having 'moderate' hearing disability if his/her disability was neither profound nor severe. Such a person would usually ask to repeat the words spoken by the speaker or would like to see the face of the speaker while he/she spoke or felt difficulty in conducting conversations.

2.11.4 **Speech disability:** This referred to persons' inability to speak properly. Speech of a person was judged to be disordered if the person's speech was not understood by the listener. Persons with speech disability included those who cannot speak, can speak only with limited words or those with loss of voice. It also included those whose speech was not understood due to defects in speech, such as stammering, nasal voice, hoarse voice and discordant voice and articulation defects, etc.

2.11.5 **Locomotor disability:** A person with - (a) loss or lack of normal ability to execute distinctive activities associated with the movement of himself/herself and objects from place to place and (b) physical deformities, other than those involving the hand or leg or both, regardless of whether the same caused loss or lack of normal movement of body – was considered as disabled with locomotor disability. Thus, persons having locomotor disability included those with (a) loss or absence or inactivity of whole or part of hand or leg or both due to amputation, paralysis, deformity or dysfunction of joints which affects his/her "normal ability to move self or objects" and (b) those with physical deformities in the body (other than limbs), such as, hunch back, deformed spine, etc. Dwarfs and persons with stiff neck of permanent nature who generally did not have difficulty in the normal movement of body and limbs were also treated as disabled.

2.12 **Abortion:** Abortion is the case of foetus born before the completion of 28 weeks since conception and showing no sign of life at birth.

2.13 **Live-birth:** When a child shows any evidence of life at birth, irrespective of the interval since conception, it is the case of a live-birth. The child may, however, expire within a very short time after birth.

2.14 **Still-birth:** It is the case of a baby born after completion of 28 weeks and showing no sign of life. The birth of a foetus caused by abortion is not considered a 'still-birth'.

For peer review only

For peer review only - http://bmjopen.bmj.com/site/about/guidelines.xhtml

Chapter Three
Morbidity and Hospitalisation

3.1 General

3.1.1 It may be mentioned at the outset that the National Health Policy was formulated and enacted in 1983. Since then, NSSO has conducted three nationwide surveys on utilisation of medical services by the various sections of the population. The present enquiry on morbidity and utilisation of medical services, the third in the series, has been carried out in the 60th round of the NSS during January to June, 2004. Although the objective of these surveys was essentially to study the utilisation of public and private health services by various sections of the population, the present survey specially attempts to measure the role of the private institutions vis-à-vis government institutions in providing health care services. It is important to note that of the first two enquiries, one was carried out during 1986-87 (NSS 42nd round) and the other during the post 'economic' liberalisation period 1995-96 (NSS 52nd round). The first enquiry on the subject, conducted only after three years of the enactment of the Health Policy, was, therefore, too early to register the effects of the policy. The results of the second survey are expected to reveal to some extent the effects of the National Health Policy jointly with the effect of economic liberalisation – although the period ever since the formulation and enactment of policy is assumed to be small. This present survey, therefore, assumes special significance for a comprehensive study on the general health care system in India and for measuring the role of private institutions in the health sector.

3.1.2 This chapter summarises the important findings of the survey and discusses the salient features pertaining to the curative aspects of the general health care system in India. Wherever possible, the results of the earlier rounds have been placed side by side so that trends and changes over the periods of survey may be examined. The focus is principally on the all-India estimates; as a secondary task, one proceeds to examine the disparities in the utilisation of the health services across the major states and across their rural and urban sectors. The estimates for the smaller States and Union Territories (UTs) have been neither presented nor considered for discussion in this chapter. In fact, the estimates for the 20 major states in the rural sector of the country and 21 major states in the urban sector have been presented in this chapter. The estimates for all the remaining states, group of UTs and for the group of North-eastern states (excluding Assam) appear (along with the estimates for the major states) only in the appendix to this report. The reason is that the sample sizes for the smaller states and UTs may not be adequate for getting sufficiently reliable estimates – at least for measuring change or inter-state comparison. A major state, for the rural sector, is one whose rural population exceeds one crore; a major state for the urban sector is defined similarly. Unless otherwise mentioned, state (major state) level estimates of the parameters discussed in this chapter are presented at the end of the chapter.

3.1.3 The discussion starts with observations on the distribution of households over some important characteristics that have relevance, directly or indirectly, with the conditions of living and health of the individuals. This occupies Section 2 of this chapter. Following this, Sections 3 to 6 deal with the morbidity rates, in general, and treatment of ailments, particulars of hospitalisation and cost of treatment jointly with related characteristics to reveal the multi-

For peer review only - http://bmjopen.bmj.com/site/about/guidelines.xhtml

dimensional aspects of the health care system of the country. The results on immunisation services received by children of age less than five years have been presented and discussed in Section 7. This section also investigates on the maternity care received by pregnant mothers. Finally, the chapter concludes with the results and discussion on the condition of the aged – those with age 60 years or more, in Section 8. In fact, it highlights the structure and composition of the aged in respect of age, sex, dependency ratio, etc. and the conditions of the aged in respect of their economic dependency, number of dependants, living arrangements, persons supporting the aged, physical immobility, etc. Note that the all-India level estimates given in this report relate to the whole of the India Union, including all the states and UTs.

3.1.4 *Use of Estimated Aggregates*: A few points on some general aspects of the survey estimates need to be stated in this paragraph. Compared to the census population or the projections thereof, population estimates from the NSSO surveys are, in general, on the lower side. This difference arises mainly due to the differences in methods and coverage adopted by the NSSO in comparison with the census operation. However, the ratios obtained from the surveys are much closer to the ratios obtained using census figures. Usually, the estimates on morbidity rates are presented as ratios, except for the estimates on expenditure on treatment, etc. To estimate an absolute number in any category, it is advisable to apply the survey estimates of ratios to the census population or projections thereof, for that category. The estimates of aggregates given in the detailed tables may help to combine the ratios but may not be used for deriving estimates of absolute numbers for a characteristic. It may also be noted that as the tables are generally presented as per thousand distributions, the figures are rounded off. Thus, while using the ratios from the survey results, it is to be noted that the accuracy of these derived aggregates will be limited to the number of significant digits available in the ratio or percentage estimates presented in the report. The estimated aggregates, wherever possible, may be used as weights for combining the ratios.

3.2 Household Profile

3.2.1 As per the survey estimates, about 959 million people lived in 199 million households in India. About 72 per cent of the households belonged to rural India and accounted for nearly 75 per cent of total population (see Table 1 in Appendix A). A household consisted, on an average, of 5.0 persons in rural areas and 4.4 persons in the urban areas. The rural population had 964 females per 1000 males while the urban had 917.

3.2.2 Monthly per capita expenditure (MPCE)

3.2.2.1 Household income, or for that matter 'level of living', is highly related with the 'general health' of the household members as well as to the extent of 'medical care' received by them. Thus, as the background information, the distribution of households and population by income level is useful information for a correlative study on morbidity and health care. As it is difficult to collect reliable income data, the NSSO collects data on consumption expenditure in its surveys. Since MPCE is a supplementary classificatory variable for correlative study of the main theme of the round, data was collected through a short set of 5 questions (see block [3] of Schedule 25.0 in Appendix C) rather than a detailed listing of consumption items that is used

For peer review only - http://bmjopen.bmj.com/site/about/guidelines.xhtml

when household consumption expenditure is the main theme of the survey. This procedure is known to underestimate the level of MPCE in comparison with the detailed schedule but expected to provide a reasonable proxy for relative ranking of the households according to level of living. The distributions of households by MPCE class for rural and urban India are presented in Statement 2.

Statement 2
Per 1000 distribution of households by MPCE class during January – June 2004

India

rural		urban	
MPCE class (Rs.)	house-holds	MPCE class (Rs.)	house-holds
less than 225	46	*less than 300*	27
225 – 255	38	*300 – 350*	19
255 – 300	88	*350 – 425*	51
300 – 340	78	*425 – 500*	99
340 – 380	93	*500 – 575*	45
380 – 420	99	*575 – 665*	81
420 – 470	102	*665 – 775*	110
470 – 525	110	*775 – 915*	101
525 – 615	109	*915 – 1120*	149
615 – 775	113	*1120 – 1500*	158
775 – 950	58	*1500 – 1925*	48
950 or more	66	*1925 or more*	110
all	1000	*all*	1000

3.2.2.2 In urban India, about 32 per cent of the households spent less than Rs. 665 per month per person. On the other hand, in rural India, about 44 per cent of the households spent less than Rs.420. Over 56 per cent of the urban households spent Rs.775 or more per person per month compared to only 12 per cent in rural areas.

3.2.2.3 *Type of Structure of Dwelling*: Structure of the dwelling not only reflects the living condition of the household and its members but also has a bearing on the health conditions of the members of the household. The distribution of households by type of structure of their dwelling units is, therefore, given in Statement 3 for each household social group for the rural and urban areas. It can be seen that more than half of the households in the rural areas resided in *semi-pucca* or *kutcha* structures. The distribution in respect of the structure of dwellings is found to deteriorate for the weaker sections of the population and is worst for the Scheduled Tribes (STs). Merely 20 per cent of the households among the *STs* resided in the dwellings made of *pucca* materials, the rest living either in *semi-pucca* or in *kutcha* dwellings. Pucca structure is much more common in the urban areas with 84 per cent of the households reporting it. The proportion of population living in *pucca* structures was highest among the *others* category of households (90 per cent) and lowest, as expected, among the *ST* households (65 per cent). The corresponding distributions for the states and the group of UTs is given in Statement 3.1 (p. 36). It can be seen that about 75 per cent or more households, in general, in the rural areas of Chhattishgarh, Assam, Orissa, Jharkhand and West Bengal lived either in *kutcha* or in *semi-pucca* structures. Though the corresponding proportions are lower in urban areas, wide inter-

Statement 3
Per 1000 distribution of households by structure of dwelling for each household social group

India

structure of dwelling	household social group				total
	ST	SC	OBC	others	
**rural**					
pucca	195	410	520	582	480
semi- pucca	570	335	306	280	332
kutcha	235	255	173	138	188
all	1000	1000	1000	1000	1000
**urban**					
pucca	653	765	813	902	842
semi- pucca	259	150	136	79	115
kutcha	88	85	50	19	43
all	1000	1000	1000	1000	1000

For peer review only - http://bmjopen.bmj.com/site/about/guidelines.xhtml

Statement 4
Per 1000 distribution of households by structure of dwelling for each household monthly per capita expenditure class

India

mpce classes	distribution by type of structure: pucca	semi-pucca	kutcha	total
		**rural**		
less than 225	259	441	299	1000
225 – 255	285	411	304	1000
255 – 300	319	421	261	1000
300 – 340	381	374	245	1000
340 – 380	400	385	215	1000
380 – 420	427	373	199	1000
420 – 470	477	335	188	1000
470 – 525	535	309	155	1000
525 – 615	575	286	139	1000
615 – 775	647	243	110	1000
775 – 950	740	190	70	1000
950 & above	839	115	47	1000
all	480	332	188	1000
		**urban**		
less than 300	494	326	179	1000
300 – 350	561	274	165	1000
350 – 425	654	244	101	1000
425 – 500	746	187	68	1000
500 – 575	738	192	69	1000
575 – 665	810	145	45	1000
665 – 775	857	111	32	1000
775 – 915	897	82	20	1000
915 – 1120	938	50	12	1000
1120 – 1500	961	31	7	1000
1500 – 1925	976	18	3	1000
1925 & above	993	4	3	1000
all	842	115	43	1000

State variations exist. One observes that *kutcha* structures housed 19 per cent of households in urban Orissa and 10-11 per cent of households in urban areas of Tamil Nadu and Bihar.

3.2.2.4 *MPCE Class and Type of Structure of Dwelling*: Generally, the quality of dwelling structure is known to be associated with the level of living of a household. From this angle, it is useful to examine the dwelling type in relation to the size of MPCE. It is seen from Statement 4 that in rural areas, while only 26 per cent of the poorest class (MPCE Rs. 225 or less) reported living in *pucca* dwellings, as many as 84 per cent of the richest class of households (MPCE Rs. 950 or above) lived in the *pucca* dwellings. The disparity between the poor and the rich is found to be less in the urban areas as compared to the rural areas. In fact, the proportion of households living in *pucca* dwellings ranged from 49 per cent (for MPCE Rs. 300 or less) to 99 per cent (for MPCE Rs. 1925 or above) in the urban areas.

3.2.2.5 *Major Source of Drinking Water*: The quality of water used for drinking is a very important determinant of health condition. The source from where drinking water is collected by the household roughly indicates its quality and, thus the awareness of the households of the need for drinking water of proper quality. Information collected on the major source of drinking water used by the household during the major part of a year has been presented in Statement 5 separately for the rural and urban areas. The most prevalent source, in the rural areas, is found to be 'tube-well/ hand pump'. Next in importance, as reported, were 'tap' and 'pucca well'. The proportions of households reporting the use of drinking water for the major part of the year from these three sources were 56 per cent, 25 per

Statement 5
Per 1000 distribution of households by major source of drinking water

India

major source of drinking water	**rural**	**urban**	**rural+urban**
bottled water	10	28	15
tap	248	676	357
tube-well/ hand pump	564	224	478
tankers	7	23	11
pucca well	139	44	115
tank/ pond reserved for drinking	13	2	10
river/ canal	8	0	6
others	10	3	8
total	1000	1000	1000

For peer review only - http://bmjopen.bmj.com/site/about/guidelines.xhtml

cent and 13 per cent, respectively, in the rural areas. The same three sources were the most important in urban areas, but in a different order. 'Tap' was the most important (68 per cent), followed by 'tube well/ hand pump' (22 per cent), and 'pucca well' (4 per cent). Thus these three sources together provided drinking water to 94 per cent of households in both rural and urban areas. A small but significant proportion of households, even in 2004, collected their drinking water either from a 'tank /pond reserved for drinking', or from a 'river/ canal': they formed 2 per cent of rural households in India. The estimates for the states are given in Statements 5.1R and 5.1U (pp 37-38).

3.2.2.6 *Treatment of Water before Drinking*: The water collected by a household for drinking is sometimes not consumed directly but only after some cleaning/treatment. Prior cleaning/treatment of water before drinking is good indicator of health awareness. It would, therefore, be of interest to examine the proportion of households resorting to cleaning of collected water, tabulated by source of drinking water. The relevant results are presented in Statement 6 separately for rural and urban areas of India. As a whole, a higher proportion (38 per cent) of households in the urban areas reported cleaning of water before drinking than in the rural (20 per cent). The proportions are appreciably higher among the households that collected their drinking water from 'tank/pond reserved for drinking', from 'tankers', from a 'pucca well' or from a 'river/canal'. Among all the sources, water collected from 'tube-well/hand pump' was perhaps felt to be safest for drinking as only 10 per cent of the households in the rural areas and 16 per cent of the households in the urban areas purified such water before drinking. State-level estimates on number per 1000 of households treating water before drinking are also given in Statement 5.1.

Statement 6
Number per 1000 of households treating water before drinking for each major source of drinking water

India

major source of drinking water	**rural**	**urban**
tap	291	436
tube-well/ hand pump	98	164
tankers	459	546
pucca well	366	570
tank/ pond reserved for drinking	551	731
river/ canal	327	584
others	276	351
all	199	375

3.2.2.7 *Methods Used for Treating Water before Drinking:* For those households which treated water before drinking, information on the method usually adopted by households for treating water was collected. The specified list of methods used to classify the method of treatment reported by household for which the data was collected in the survey were 'ultra-violet/resin', 'filter', 'boiling', 'cloth screen' and 'any disinfectant'. The results are given in Statement 7 in the form of distribution of households that reported treatment of water before drinking by type of treatment done, separately for rural and urban areas at all India level. Most of the rural as well as urban households used 'cloth screen' for purification of drinking water. Among the households reporting purification of water before drinking, about 63 per cent in the rural areas and nearly 40 per cent in the urban areas used the traditional method of 'cloth screen'. The most scientific method among the specified methods, 'ultra-violet/resin', was adopted by only 5 per cent in the urban areas and less than 1 per cent in the rural areas.

For peer review only - http://bmjopen.bmj.com/site/about/guidelines.xhtml

3.2.2.8 *Age Distribution of Population*: Any study relating to a human population remains incomplete unless its size and age-sex composition is known before its specific characteristics are examined. Thus, the distribution of population by age-group and sex, as background information, will certainly help in easy understanding of the results, particularly the study of various indicators of morbidity. In Statement 8, the distribution of population over broad age groups is presented separately for males and females in rural and urban areas. Comparable results based on the 58th round (July – December 2002) survey are also presented in this statement. It can be seen that while the proportion of boys and girls (age 0-14 years) was around 37 per cent in the rural areas, it was around 30 per cent in the urban areas. On the other hand, the aged (60 years or more) constituted a little over 7 per cent of the rural male, rural female and urban female and a little over 6 per cent of the urban male population. No marked differences can be observed between the estimates obtained from the two surveys.

Statement 7
Per 1000 distribution of households that treated water before drinking by type of treatment

India

treatment of water before drinking	**rural**	**urban**
ultra-violet/resin	8	53
filter	248	254
boiling	89	275
cloth screen	626	395
any disinfectant	11	10
others	18	13
total	1000	1000

3.3 Morbidity and Health Care

Statement 8
Per 1000 distribution of population by broad age group in NSS 60th and 58th rounds

India

age group (years)	**rural male**		**rural female**	
	60th rd.	58th rd.	60th rd.	58th rd.
0-14	376	368	357	351
15-29	249	257	256	255
30-44	189	185	196	201
45-59	115	117	119	117
60 & above	70	72	71	76
all ages	1000	1000	1000	1000
	**urban male**		**urban female**	
age group (years)	60th rd.	58th rd.	60th rd.	58th rd.
0-14	299	302	290	297
15-29	296	305	288	294
30-44	213	205	222	214
45-59	131	124	129	120
60 & above	62	63	71	75
all ages	1000	1000	1000	1000

Note: Survey period, NSS 58th round: July to December, 2002

3.3.1 *Level of Morbidity*: Statement 9 gives the survey estimates on prevalence of morbidity. For the purpose of the survey, it is termed Proportion of Ailing Persons (PAP), measured as the number of persons reporting ailment during a 15-day period per 1000 persons for some broad age-groups. It shows a difference of 1 percentage point in the PAP between the rural and urban areas. The rate differed between the male and female population by 1 percentage point in rural India and 2 percentage points in urban India. As expected, the PAPs are found to be higher for children and much higher for the higher age groups – the lowest being the PAPs for the youth (age 15-29 years). While 12 to 15 per cent of persons in the age-bracket 45-59 reported ailments, the proportion was as high as 28 and 37 per cent for the aged persons (age 60 years or more) in rural and urban areas, respectively. The rural–urban differentials are also significant among the aged. The estimates for different broad age groups are also shown in Figure 1.

For peer review only - http://bmjopen.bmj.com/site/about/guidelines.xhtml

Statement 9
Number (per 1000) of persons reporting ailment (PAP) during last 15 days

India

broad age-groups	rural			urban			rural + urban		
	male	female	all	male	female	all	male	female	all
0-14	76	68	72	84	74	79	78	69	74
15-29	41	57	49	44	56	50	42	56	49
30-44	64	93	78	64	95	79	64	93	78
45-59	107	132	119	127	173	149	113	143	128
60 & above	285	282	283	352	383	368	301	307	304
**all**	**83** (54)	**93** (57)	**88** (55)	**91** (51)	**108** (58)	**99** (54)	**85** (53)	**97** (57)	**91** (55)

Figures in brackets give the corresponding estimates from NSS 52nd Round (1995-96)

Figure 1: Age-specific morbidity prevalence (PAP)

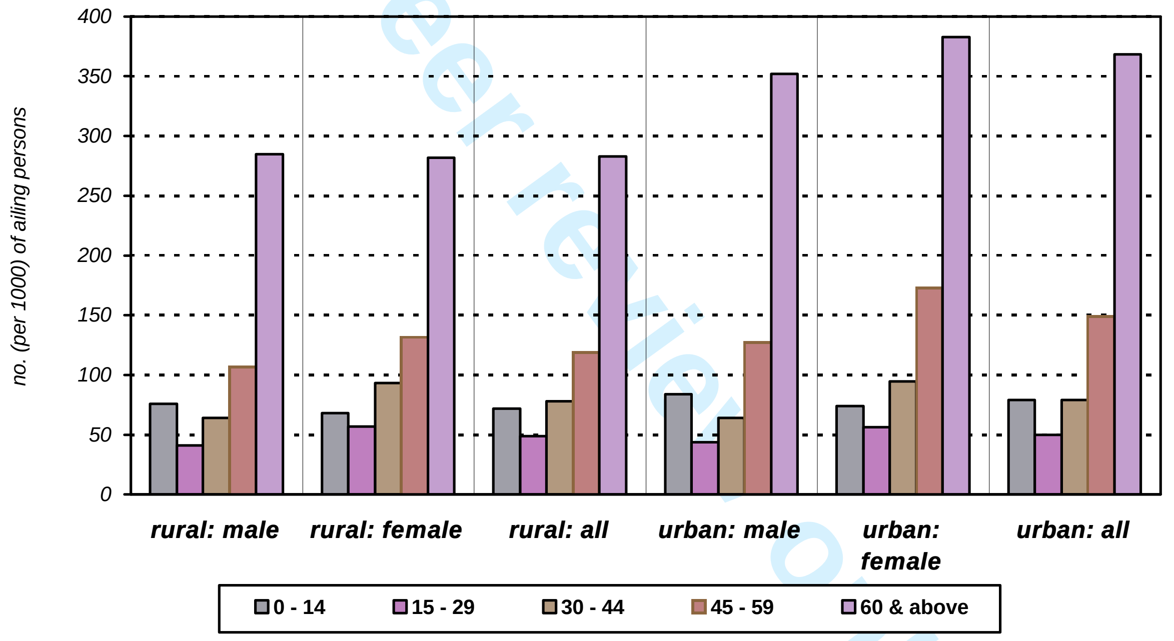

3.3.1.1 The morbidity rate (PAP) presented in Statement 9 gives the estimated proportion of persons reporting ailment suffered at any time during the reference period and are not strictly the *prevalence rates* as recommended by the Expert Committee on Health Statistics of the WHO. The WHO defines *prevalence rate* as the ratio between the number of spells of ailment suffered at any time during the reference period and the population exposed to the risk. It measures the *frequency of illnesses* prevailing during the reference period, whereas Statement 9 gives the *number of persons reporting ailments* during a 15-day period per 1000 persons.

3.3.1.2 As the estimates are based on self-reported morbidity data, rather than on medical examination, the information on number spells of different ailments suffered during the reference period is not likely to reflect the objective illness-status of the patients, particularly the number of

For peer review only - http://bmjopen.bmj.com/site/about/guidelines.xhtml

diseases a patient is afflicted with. Thus, only the estimated proportion (number per 1000) of ailing persons is used as a measure of morbidity rates in this report. The comparison of the survey estimates of morbidity rates, with those of the previous NSS round (52nd round: July 1995 – June 1996) shows that the PAP has increased by 3 and 4 percentage points in the rural and urban areas, respectively. The increase in PAP over time is probably due to increased health consciousness over time and consequently, improvement in the self-reporting of ailments by the informants. The PAPs for the major states are given in Figure 2.

Figure 2: Number (per 1000) of ailing persons (PAP)

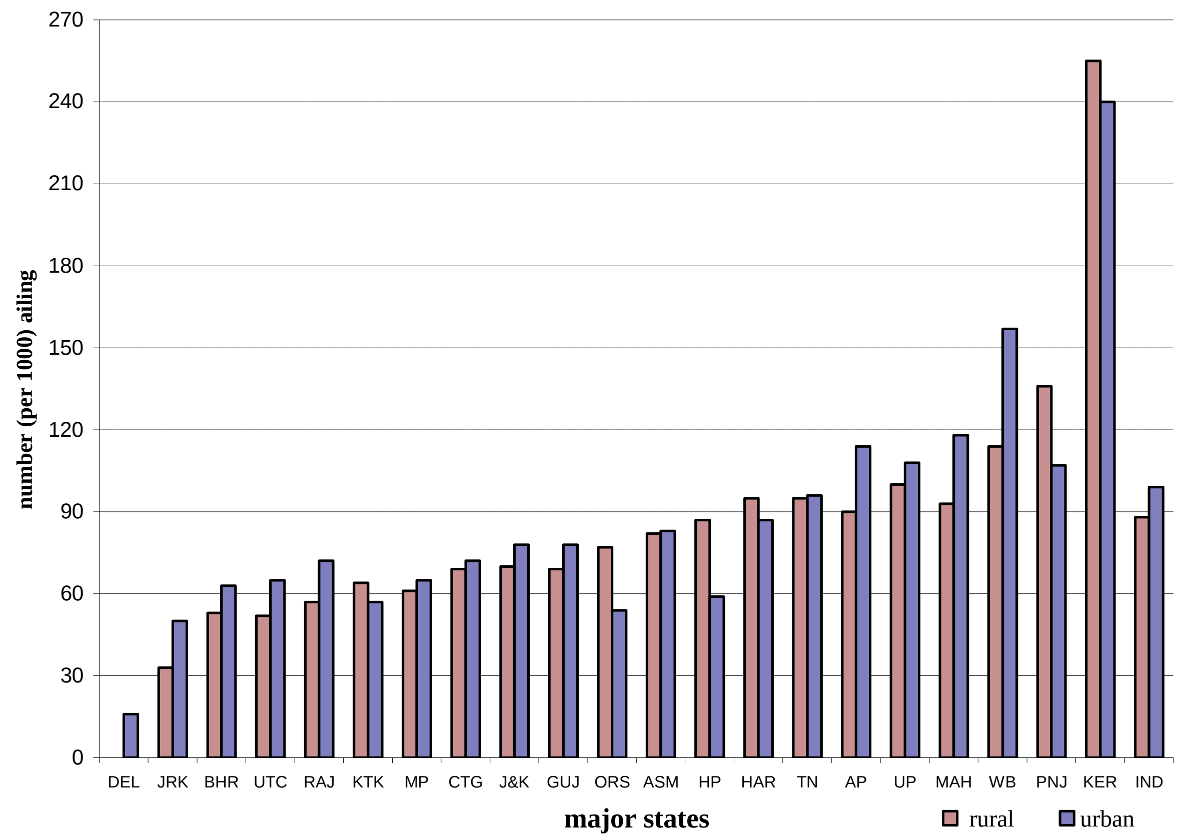

3.3.1.3 *PAP and Level of Living*: Statement 10 shows the relationship between morbidity reporting and level of living, measured by per capita monthly consumption expenditure (MPCE). The data on household consumption expenditure were collected through a much smaller set of questions in comparison with the elaborate schedule of enquiry generally used in NSSO for collecting consumption data (see para 3.2.2.1). It reveals a broad positive association between MPCE and PAP, in both rural and urban areas. The range of variation in PAP was larger in the rural areas than in the urban areas. If MPCE is considered to be a proxy for level of living of the households, the data appear to show that the level of morbidity tends to rise with the level of living. This may mean either that the poor are less prone to sickness than the rich, or that the reporting of morbidity improves with improvement in the level of living. Of the two hypotheses, the second seems to be the more plausible.

For peer review only - http://bmjopen.bmj.com/site/about/guidelines.xhtml

3.3.1.4 *PAP and Social Groups of Households*: The PAPs in households belonging to different social groups can be an interesting object of study. The estimates are given in Statement 11. It may be seen from the statement that the estimates of PAP are found to be lowest among the *STs* followed by *SCs*, and is highest among the *others* category of persons in both rural and urban areas. It may be mentioned that in the 52nd round survey, the ailments were classified by their duration viz., short-duration or long-duration, determined on the basis of the type of ailment (disease) as reported by the ailing member. In the 60th round, this distinction among the ailments (diseases) based on the report of the ailing member was not adopted since the duration of an ailment may not be reported correctly by the respondent and any classification based on this reporting may not be adequate and proper.

Statement 10
PAP* during last 15 days for each household monthly per capita expenditure class (Rs.)

India

rural		urban	
MPCE class	PAP	MPCE class	PAP
less than 225	64	*less than 300*	65
225 – 255	56	*300 – 350*	89
255 – 300	64	*350 – 425*	83
300 – 340	66	*425 – 500*	83
340 – 380	77	*500 – 575*	85
380 – 420	80	*575 – 665*	85
420 – 470	86	*665 – 775*	101
470 – 525	93	*775 – 915*	93
525 – 615	94	*915 – 1120*	100
615 – 775	112	*1120 – 1500*	118
775 – 950	137	*1500 – 1925*	115
950 & above	165	*1925 & above*	149
all	88	*all*	99

*number per 1000 of ailing persons

Statement 11
PAP during last 15 days for each household social group

India

household social groups	**rural**	**urban**	**rural + urban**
ST	58	61	58
SC	88	86	88
OBC	87	91	88
Others	102	113	106
all	88	99	91

3.3.2 *Commencement of Ailment*: Statement 12 gives the age and gender-specific **p**ropor-tions (number per 1000) of **p**ersons reporting **c**ommencement (PPC) of any ailment during the reference period of 15 days preceding the date of survey, separately for rural and urban areas. It may be noted that the PPC is also different from the incidence rate, as defined in the recommendations of the Expert Commission on Health Statistics of the WHO (see para 3.3.1.1). While the incidence rate measures the frequency

Statement 12
Number (per 1000) of persons reporting commencement of any ailment (PPC) during last 15 days

India

broad age-group	**rural**			**urban**			**rural+urban**		
	male	female	all	male	female	all	male	female	all
0-14	57	51	54	63	54	59	58	52	55
15-29	25	33	29	29	36	32	26	34	30
30-44	31	44	37	35	41	38	32	43	38
45-59	40	52	46	30	52	40	38	52	45
60 & above	65	70	68	48	61	55	61	68	64
all	43 (30)	46 (32)	45 (31)	41 (30)	46 (31)	44 (30)	42 (30)	46 (32)	44 (31)

Note: PPCs for 52nd round are in parentheses;

For peer review only - http://bmjopen.bmj.com/site/about/guidelines.xhtml

of illnesses commencing during a reference period, the PPC gives the estimated proportion of persons reporting commencement of any ailment. Like the PAPs, the PPCs do not reflect any perceptible rural-urban difference. The observed almost equal levels of morbidity reporting in the rural and urban areas is, apparently, contrary to the poorer health conditions in the rural areas reflected by health indicators like infant mortality rate and mortality rate at other ages, expectation of life at birth, etc.

3.3.2.1 There is a well-established direct association between morbidity reporting and level of health consciousness. In an attempt to understand how health consciousness influences morbidity reporting, Infant Mortality Rates (IMRs) estimated through the Sample Registration Scheme of the Office of the Registrar-General of India are also provided alongside PAPs and PPCs for the

Statement 13

Number (per 1000) of persons reporting ailment (PAP) and number reporting commencement of any ailment (PPC) during last 15 days, along with mortality rate (IMR) for major states

major state	**rural**			**urban**		
	PAP	PPC	IMR	PAP	PPC	IMR
Andhra Pradesh	90	36	71	114	47	35
Assam	82	58	73	83	48	38
Bihar	53	32	62	63	30	50
Chhattisgarh	69	38	-	72	31	-
Delhi	@	@	@	16	7	-
Gujarat	69	29	68	78	29	37
Haryana	95	48	65	87	43	51
Himachal Pradesh	87	26	-	59	19	-
Jammu & Kashmir	70	30	-	78	34	-
Jharkhand	33	21	-	50	21	-
Karnataka	64	32	65	57	20	25
Kerala	255	103	11	240	100	8
Madhya Pradesh	61	32	90	65	36	56
Maharashtra	93	44	52	118	50	34
Orissa	77	49	91	54	30	56
Punjab	136	61	55	107	44	35
Rajasthan	57	23	81	72	27	55
Tamil Nadu	95	54	50	96	49	32
Uttaranchal	52	31	-	65	25	-
Uttar Pradesh	100	55	83	108	55	58
West Bengal	114	56	52	157	62	36
India	88	45	69*	99	44	40*
	(55)	(31)	(79)	(54)	(30)	(47)

@ 'Not presented'. Note: '-' indicates that figures are not available.

* Excludes Nagaland

1. The data on Infant Mortality Rate (IMR) are estimates for 2002 obtained by the Sample Registration Scheme of the Registrar-General of India. India.
2. IMR values in brackets are averages of IMRs estimated for 1995 and 1996 by the Sample Registration Scheme of the Registrar-General of India. Other figures in brackets are NSS (52nd round) estimates for 1995-96.

For peer review only - http://bmjopen.bmj.com/site/about/guidelines.xhtml

major states in Statement 13. IMR is a widely accepted indicator of general health of a population and health care services available to it. Moreover, the better the health care services,the higher is the level of health consciousness expected to be. Thus, IMR has been taken as a broad indicator of health consciousness. Interestingly, while the IMRs and PAPs for Kerala, Punjab, West Bengal and many other states suggest an expected negative relationship between infant mortality and morbidity reporting, the data on rural areas of Orissa, Rajasthan and Madhya Pradesh seem to point to the contrary. The statement also reveals similar features for urban PAP as well as PPC of the rural and urban areas.

3.3.2.2 Statement 13 also reveals wide inter-state variations in PAP as well as PPC in both rural and urban areas. The level of morbidity in the rural areas of the major states (other than Delhi for which the sample size is too small), measured in terms of PAP, varied from 33 in Jharkhand to 255 in Kerala. The variation in PPC, on the other hand, ranged from 21 in Jharkhand to 103 in Kerala. Comparatively, inter-state variation in morbidity reporting in the urban areas was less pronounced, with PAP ranging from 50 in Jharkhand to 240 in Kerala. It is, however, seen that the states with relatively high morbidity reporting in the rural areas also reported high morbidity in the urban areas. In fact, in both rural and urban areas, the states of Kerala, Punjab, West Bengal and Andhra Pradesh were at one extreme reporting high levels of morbidity, while at the other extreme were Jharkhand, Uttaranchal, Bihar and Rajasthan with low levels of morbidity reporting. It may be noted that Kerala besides having the lowest infant mortality rate, was also far ahead of other states in achievements in the field of health care, as reflected by other indicators like birth rate, proportion of institutional births, life expectancy, etc. But since the state had a high proportion of aged people (60 years and above) with, as one might expect, high levels of morbidity, the overall morbidity levels of this state tend to be higher than those of other states. The proportion of the aged in Kerala (11.2 per cent) is found by the current survey to be much higher than that of the country as a whole (6.9 per cent).

3.4 Treatment of Ailments

3.4.1 *Proportion of Ailing Persons Treated*: Persons who are ailing do not always get their ailments medically treated and sometimes resort to self-medication, home remedies or no medical care. Statement 14 gives the percentage of ailments treated. While for the present round (Jan-June 2004), the estimates relate to the spells of ailments treated, the estimates obtained from the 52nd (1995-96) and the 42nd (1986-87) round surveys relate to the proportion of ailing persons medically treated. These two sets of estimates are almost comparable since the average number of spells per ailing person, for all the categories of persons considered in the Statement, has been found to be around 1. In all the surveys, the percentage of ailing persons who got their ailments treated is found to be higher

Statement 14
Percentage spells of ailments treated during last 15 days

India

gender	2004 *60th rd.*	1995-96 *52nd rd**.	1986-87 *42nd rd**.
**rural**			
male	82 (1.0)	84	83
female	82 (1.0)	82	80
person	82 (1.0)	83	82
**urban**			
male	90 (1.1)	91	90
female	89 (1.1)	90	88
person	89 (1.1)	91	89

Figures in brackets give average number of spells of ailment per ailing persons
* % of ailing persons treated for 52nd and 42nd rounds.

For peer review only - http://bmjopen.bmj.com/site/about/guidelines.xhtml

Statement 15
Number per 1000 of spells of ailments treated during 15 days by MPCE class

India

rural		urban	
MPCE class	treated spells	MPCE class	treated spells
less than 225	759	*less than 300*	782
225 – 255	779	*300 – 350*	875
255 – 300	777	*350 – 425*	847
300 – 340	756	*425 – 500*	853
340 – 380	796	*500 – 575*	896
380 – 420	806	*575 – 665*	863
420 – 470	820	*665 – 775*	905
470 – 525	815	*775 – 915*	911
525 – 615	829	*915 – 1120*	901
615 – 775	869	*1120 – 1500*	896
775 – 950	878	*1500 – 1925*	888
950 & above	894	*1925 & above*	945
all	823	all	892

in the urban areas than in the rural areas. The reported rates of treatment of the sick do not indicate any perceptible gender bias in either of the surveys. Moreover, the results of the three rounds do not reveal any detectable change over time in the percentage of ailing persons treated.

3.4.2 *Spells of Ailments Treated and MPCE*: Statement 15 reveals the relationship between the percentage of treated spells of ailments and monthly per capita consumption expenditure (MPCE) separately for the rural and urban areas. The proportion varied between 76 to 89 per cent in the rural areas and 78 to 95 per cent in the urban areas over the different expenditure classes, the proportion increasing gradually with the level of expenditure or levels of living. The overall difference between the rural and urban areas was about 7 percentage points – more spells of ailments being treated in the urban areas.

3.4.3 *Untreated Spells of Ailment*: Untreated spells of ailments, whose proportion can be derived from Statement 13, are further distributed in Statement 16 by reason for not taking treatment. The corresponding estimates for NSS 52nd round are also presented in the statement. It is seen that, in the current round of survey as well as in the previous survey, the reason most often cited for no treatment was that the ailment was ‘not serious’. This reason was reported by 32 per cent and 50 per cent of the cases of untreated ailments in the rural and urban areas, respectively. In the 52nd round, this reason accounted for about 50 to 60 per cent of the cases of untreated ailment. The proportion is found to have come down considerably in the rural areas since 1995-96, pointing to an increase in health consciousness among rural households. The ‘financial problem’ was next in importance as a reason for no treatment, accounting for 28 per cent and 20 per cent of the untreated ailments in the rural and urban areas, respectively.

Statement 16
Percentage distribution of untreated spells of ailments by reason for no treatment

India

reason for no treatment	rural		Urban	
	2004 *60th rd*	‘95-96 *52nd rd*	2004 *60th rd*	‘95-96 *52nd rd*
no medical facility	12	9	1	1
facility available but:				
lack of faith	3	4	2	5
long waiting	1	1	2	1
financial problem	28	24	20	21
ailment not considered serious	32	52	50	60
Others (incl. n. r.)	24	10	25	12
all (incl. n.r. cases)	100	100	100	100

Note: For the 52nd round, estimates relate to untreated persons

For peer review only - http://bmjopen.bmj.com/site/about/guidelines.xhtml

Statement 17
Per 1000 distribution of treated spells of ailments during 15 days by source of treatment (institution) for each MPCE class

India

rural			urban		
MPCE class (Rs.)	per 1000 no. of ailments treated		MPCE class (Rs.)	per 1000 no. of ailments treated	
	Govt. insti.	Pvt. insti.		Govt. insti.	Pvt. insti.
less than 225	296	704	*less than 300*	260	740
225 – 255	262	738	*300 – 350*	214	786
255 – 300	257	743	*350 – 425*	255	745
300 – 340	262	738	*425 – 500*	253	747
340 – 380	214	786	*500 – 575*	230	770
380 – 420	220	780	*575 – 665*	261	739
420 – 470	222	778	*665 – 775*	171	829
470 – 525	202	798	*775 – 915*	215	785
525 – 615	227	773	*915 – 1120*	190	810
615 – 775	214	786	*1120 – 1500*	156	844
775 – 950	230	770	*1500 – 1925*	171	829
950 & above	179	821	*1925 & above*	109	891
all	224	776	all	192	808

3.4.4 *Share of Public Provider in Treatment of Illnesses*: The public providers for health care include government hospitals, government clinics, government dispensaries, Primary Health Centres (PHCs) and the Community Health Centres (CHCs), and the state and central government assisted ESI hospitals and dispensaries. The rest of the providers fall in the category of 'private' sources. The 'private' sources include private doctors, nursing homes, private hospitals, charitable institutions, etc. Statement 17 shows how the share of public provider in treatment of ailments varies with expenditure class. It reveals that a large proportion of total ailments were treated from the private sources - 78 per cent in the rural areas and 81 per cent in the urban areas, while the overall proportion of treated (spells of) ailments to all ailments was 82 per cent in the rural and 89 per cent in the urban areas (see Statement 15). By and large, the statement also shows a progressive, if gradual, decline with rise in level of living in the reliance on public sector institutions as measured by proportion of ailments treated in such institutions. For the people in the lowest MPCE class (less than Rs. 225) in the rural areas, treatment was received from the government institutions in about 30 per cent of the treated cases, whereas the proportion was 18 per cent for highest MPCE class (Rs. 950 & above). In the urban areas, the corresponding proportions were 26 per cent and 11 per cent. The share of the public provider in treatment of ailments is given in Statement 18 for the different household social groups. The statement shows more reliance on the public provider among the households belonging to the scheduled categories, both in the rural and in the urban areas. While for the people belonging to the *ST* and *SC* categories, treatment was from the Government institutions in about 24 to 33 per cent of the treated cases of ailments, the proportion was about 17 to 20 per

Statement 18
Proportion (per 1000) of spells of ailments treated during 15 days and per 1000 distribution of treated spells of ailments by source of treatment (institution) for each household social group

India

hhd. social group	rural			urban		
	number (per 1000) treated spells	source of treatment		number (per 1000) treated spells	source of treatment	
		Govt. instn.	Pvt. instn.		Govt. instn.	Pvt. instn.
ST	767	325	675	878	301	699
SC	798	262	738	867	240	760
OBC	830	205	795	890	204	796
Others	842	204	796	902	170	830
all	823	224	776	892	192	808

For peer review only - http://bmjopen.bmj.com/site/about/guidelines.xhtml

cent for the people belonging to the *others* categories. The share of public provider in non-hospitalised treatment of ailments is also shown in Figure 3.

Figure 3: Share of public provider in non-hospitalised medical treatment of ailments– all-India

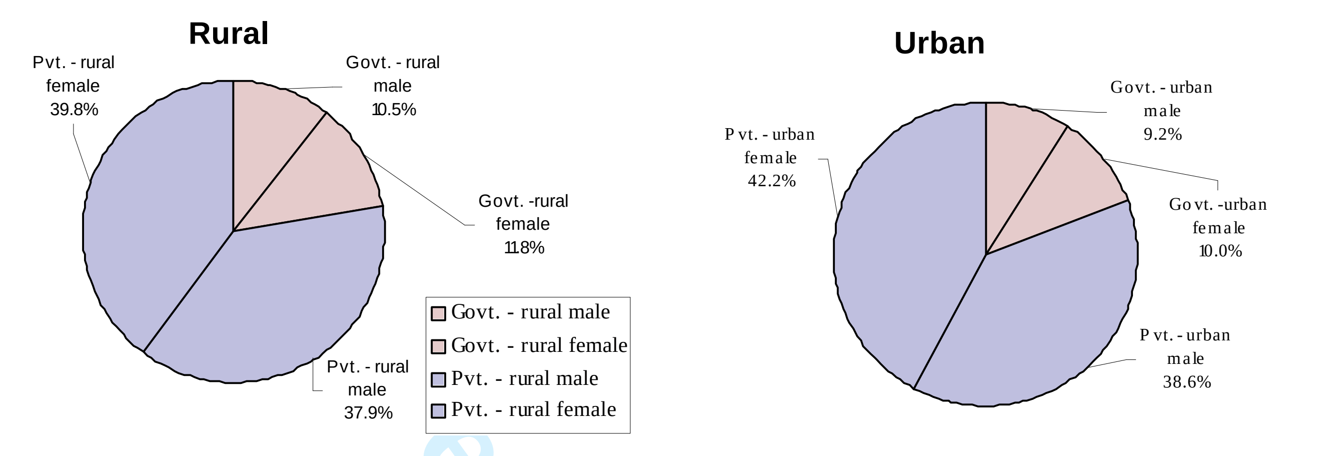

Statement 19
Percentage of spells of ailments treated (non-institutional) during 15 days and percentage distribution of treated spells of ailments by source of treatment in major states

major state	rural			urban		
	% spells of ailments treated	source of treatment (%)		% spells of ailments treated	source of treatment (%)	
		Govt.	Pvt.		Govt.	Pvt.
Andhra Pradesh	77	21	79	88	20	80
Assam	79	27	73	94	24	76
Bihar	81	5	95	88	11	89
Chattisgarh	89	15	85	90	20	80
Delhi	@	@	@	95	23	77
Gujarat	84	21	79	93	18	82
Haryana	94	12	88	95	20	80
Himachal Pradesh	94	68	32	92	86	14
Jammu & Kashmir	82	52	48	94	51	49
Jharkhand	92	13	87	96	24	76
Karnataka	78	34	66	87	16	84
Kerala	87	37	63	90	22	78
Madhya Pradesh	87	23	77	95	23	77
Maharashtra	88	16	84	92	11	89
Orissa	76	51	49	86	54	46
Punjab	94	16	84	96	18	82
Rajasthan	90	44	56	90	53	47
Tamil Nadu	78	29	71	87	22	78
Uttaranchal	89	18	82	98	35	65
Uttar Pradesh	77	10	90	88	13	87
West Bengal	80	19	81	83	20	80
India	82	22	78	89	19	81

@ not presented

3.4.5 *Inter-state Variation in Proportion of Ailments Treated by Government Sources*: Statement 19 reveals wide inter-state variation in the percentage of ailments treated as well as in the percentage of ailments treated from government sources. Relatively high percentage of treated ailments was reported in the rural areas of Punjab, Haryana, Himachal Pradesh and Jharkhand, and in the urban areas of Uttaranchal, Jharkhand, Punjab, Madhya Pradesh, Haryana, Delhi, Assam and Jammu & Kashmir. But, even among these states, the utilisation of government sources for treatment of ailments varied considerably. While use of public health care facilities for treatment of ailment was lowest in the rural areas of Bihar (5 per cent), preceded by Uttar Pradesh,

For peer review only - http://bmjopen.bmj.com/site/about/guidelines.xhtml

Statement 20
Percentage of treated ailments receiving non-hospitalised treatment from government sources

major state	rural			urban		
	2004 *60th Rd*	1995-96 *52nd Rd*	1986-87 *42ndRd*	2004 *60th Rd*	1995-96 *52nd Rd*	1986-87 *42ndRd*
Andhra Pradesh	21	22	12	20	19	16
Assam	27	29	40	24	22	26
Bihar	5	13	14	11	33	17
Chattisgarh	15	*	*	20	*	*
Delhi	@	*	*	23	*	*
Gujarat	21	25	28	18	22	18
Haryana	12	13	15	20	11	19
Himachal Pradesh	68	*	*	86	*	*
Jammu & Kashmir	52	*	*	51	*	*
Jharkhand	13	*	*	24	*	*
Karnataka	34	26	32	16	17	30
Kerala	37	28	32	22	28	33
Madhya Pradesh	23	23	24	23	19	28
Maharashtra	16	16	21	11	17	15
Orissa	51	38	37	54	34	43
Punjab	16	7	12	18	6	11
Rajasthan	44	36	46	53	41	52
Tamil Nadu	29	25	28	22	28	31
Uttaranchal	18	*	*	35	*	*
Uttar Pradesh	10	8	*	13	9	14
West Bengal	19	15	16	20	19	20
India	22	19	21	19	20	24

Note: 1. The estimates of the 52nd round are based only on the treatments with reported source of treatment.
2. * denotes estimate not available and @ denotes estimate not presented.

Jharkhand, Maharashtra, Punjab, Uttaranchal and West Bengal, it was highest in Himachal Pradesh (68 per cent), followed by Jammu & Kashmir, Orissa and Rajasthan. In the urban areas, although the proportion of treated cases of ailments was, in general, higher than in the rural, the reliance on the government health institutions was less than in the rural areas. This is perhaps, to some extent, due to easy availability of and accessibility to the private health institutions in urban. The percentage of ailments treated by government sources was reported to be as low as 11 per cent in the urban areas of Bihar and Maharashtra, preceded by Uttar Pradesh (13 per cent), Karnataka (16 per cent), Gujarat, Punjab (18 per cent each) and. West Bengal (20 per cent).

3.4.6 *Changing Role of Public Institutions*: Statement 20 shows the changes in the share of government institutions in the case of non-hospitalised treatment of ailments for different states over the periods. It can be seen that there has been a marginal change in the share at the all-India level in both rural and urban areas. While some increase is seen in the utilisation of government institutions in the rural areas, the reverse is the case in the urban areas. States showing an increase in this share were Orissa, Kerala, Karnataka and Rajasthan in respect of rural areas, and Orissa, Punjab and Rajasthan in respect of urban areas. While Bihar and Gujarat showed a decline in the share of public institutions in treatment of non-hospitalised ailments in both rural and urban areas, a significant decline in the share of public institutions in treating ailments can be seen in the urban areas of Kerala, Maharashtra and Tamil Nadu.

3.5 Hospitalised Treatment of Ailments

3.5.1 *Proportion of Persons Hospitalised*: Medical treatment of an ailing person as an inpatient in any medical institution having provision for treating the sick as inpatients is considered as hospitalised treatment. Statement 21 gives the estimates of number (per 1000) of persons hospitalised during a reference period of 365 days. It may be noted that the average number of

For peer review only - http://bmjopen.bmj.com/site/about/guidelines.xhtml

cases of hospitalisation per hospitalised person was around 1.1 in both the rural and the urban areas. It is seen that the estimated proportion of hospitalised persons differed substantially between the rural and the urban areas. About 3.1 per cent of the urban population were hospitalised at some time during a reference period of 365 days. The proportion of persons hospitalised in the rural areas was much lower (2.3 per cent). The survey results, however, do not reflect any systematic gender differential in this respect, either in the rural or in the urban areas. The rate increases with the age of a person and is the highest for the aged (60+) persons, both in rural and urban areas. Among the aged, the gender differential is more pronounced in the urban than the rural areas. The corresponding estimates for the states are given in Statement 21.1 (p. 39).

Statement 21
Number (per 1000) of persons hospitalised during a reference period of 365 days

India

age-group	male	female	person
**rural**			
0-14	15	10	12
15-29	17	21	19
30-44	23	27	25
45-59	41	37	39
60 & above	63	49	56
all	23	22	23 (1.1)
NSS 52nd Rd.	14	13	13 (1.1)
**urban**			
0-14	22	17	20
15-29	18	26	21
30-44	28	31	29
45-59	51	45	48
60 & above	102	81	91
all ages	31	31	31 (1.1)
NSS 52nd Rd.	20	20	20 (1.1)
**rural +urban**			
0-14	16	11	14
15-29	17	22	20
30-44	25	28	26
45-59	44	39	41
60 & above	72	57	64
all ages	25	24	25 (1.1)
NSS 52nd Rd.	16	15	15 (1.1)

Note: Figures in parentheses give the average number of cases of hospitalisation per hospitalised person

3.5.2 *Hospitalised Cases and Level of Living*: Statement 22 reveals the relationship between the number (per 1000) of persons hospitalised during the 365 days preceding the date of survey and average monthly per capita consumption expenditure (MPCE), separately for male and female populations of the rural and urban areas of the country as a whole. Considering MPCE as a proxy for level of living, the estimates suggest a positive association between level of living and the rate of hospitalisation in both rural and urban areas, except for the highest two MPCE classes. The rise in the rate is steeper for the rural than for the urban areas. Though the behaviour for the last two MPCE classes cannot be explained fully, it seems that cases of hospitalisation were more common - 9 to 16 per cent - among the middle and upper middle classes of levels of living.

Statement 22
Number per 1000 of hospitalised cases of treatment by household MPCE class

India

rural				urban			
MPCE class	male	female	person	MPCE class	male	female	person
less than 225	31	33	32	*less than 300*	28	22	25
225 – 255	21	29	25	*300 – 350*	17	19	18
255 – 300	57	54	56	*350 – 425*	50	51	50
300 – 340	64	59	62	*425 – 500*	101	95	98
340 – 380	74	71	72	*500 – 575*	49	52	50
380 – 420	97	87	92	*575 – 665*	88	104	95
420 – 470	86	94	90	*665 – 775*	131	109	121
470 – 525	106	117	111	*775 – 915*	111	117	114
525 – 615	124	117	121	*915 – 1120*	121	139	130
615 – 775	151	145	148	*1120 – 1500*	168	143	156
775 – 950	72	80	76	*1500 – 1925*	53	51	52
950 & above	116	113	115	*1925 & above*	84	99	91
all	1000	1000	1000	*all*	1000	1000	1000

3.5.3 *Proportion of Persons Hospitalised -- Inter-state Variation*: Statement 21.2 gives the estimated proportion (per 1000)

For peer review only - http://bmjopen.bmj.com/site/about/guidelines.xhtml

Statement 21.2
Proportion (per 1000) of persons hospitalised in rural and urban areas and population per bed in the state

major state	no. per 1000 hospitalised		popn. per hospital bed*
	**rural**	**urban**	
AP	22	28	1057
Assam	11	16	1782
Bihar	10	10	3029
Chhattisgarh	12	27	-
Delhi	-	11	493
Gujarat	29	36	709
Haryana	32	31	3026
HP	32	31	
J & K	18	20	4790
Jharkhand	9	22	-
Karnataka	23	26	1319
Kerala	101	90	325
MP	18	29	5582
Maharashtra	30	36	920
Orissa	23	30	3064
Punjab	30	30	1623
Rajasthan	18	25	3175
Tamil Nadu	37	37	1135
Uttaranchal	17	19	-
UP	13	20	2647
West Bengal	23	35	1464
India	23	31	1503

*Relate to 1.1.2002, except for Andhra Pradesh(1998), Assam(1991), Bihar(1992), Gujarat(1995), Karnataka(1998), Madhya Pradesh, Kerala and Orissa (2001), Maharashtra(2000), Tamil Nadu(1990) and Uttar Pradesh(1986) and are taken from 'Health Information of India' (2003), Central Bureau of Health Intelligence, Directorate General of Health Services, Ministry of Health and Family Welfare, Govt. of India.

of persons hospitalised during a reference period of 365 days in the rural and urban areas of major states. The estimates reveal wide inter-state variation in the proportion. In Kerala, the proportion, in both rural and urban areas, was much higher than in the rest of the major states. While for the country as a whole, out of every 1000 persons, 23 in the rural areas and 31 in the urban areas were hospitalised during a period of 365 days, the corresponding estimates for Kerala were as high as 101 and 90. The other states, reporting relatively high proportions of persons hospitalised though much less than Kerala were Tamil Nadu, Haryana, Maharashtra and Himachal Pradesh, rural Punjab and urban areas of West Bengal and Gujarat.

3.5.3.1 The data on health care services available from official sources is regularly compiled by the Directorate General of Health Services, Ministry of Health and Family Welfare, of the Government of India. The information relating to availability of beds for hospitalised treatment according to this Directorate is also included in Statement 21.2. Notice that population per hospital bed is a crude measure that does not distinguish between rural and urban location nor take account of the nature and perceived quality of hospitalisation services that indirectly get reflected in the survey-based estimates being compared. Consequently, one-to-one relationship between the two are not to be expected. Nevertheless the survey results on hospitalisation seem to be broadly consistent with the data from the official source. The association is relatively closer with the survey-based results for the urban areas possibly because of the predominantly urban location of the hospital. Kerala, with an exceptionally advanced health care system in the country, had, according to the official source, a hospital bed for every 325 persons and the results of the present survey indicate an exceptionally high proportion of persons receiving hospitalised treatment in the state. Among the other states reporting a high proportion of persons receiving hospitalised treatment, Gujarat, Maharashtra, and Tamil Nadu had a hospital bed, on an average, for a relatively small number of persons. However, in Haryana, where a high proportion of persons reporting hospitalised treatment was reported, the ratio of population to number of hospital beds was much higher than the national average. The estimates for proportion of hospitalised cases are also shown in Figure 4.

3.5.4 *Hospitalisation by Type of Ailment*: Number (per 1000) of persons hospitalised with certain specific ailments, or ailment types, is presented in Statement 23. It may be of interest to note that

For peer review only - http://bmjopen.bmj.com/site/about/guidelines.xhtml

Figure 4: Number (per 1000) of persons hospitalised

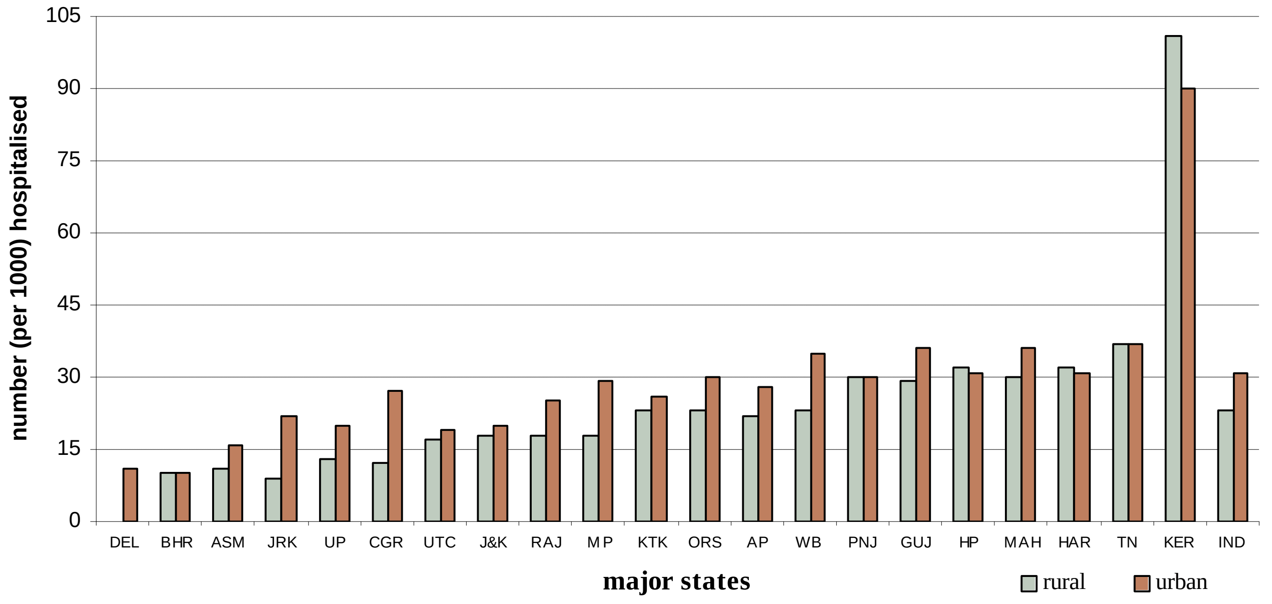

Statement 23
Per 1000 distribution of persons hospitalised by type of ailment

India

type of ailment*	rural	urban
Diarrhoea/ dysentery	76	62
Gastritis/ gastric or peptic ulcer	48	39
Hepatitis/Jaundice	15	22
Heart disease	43	80
Hypertension	18	32
Respiratory incl. ear/nose/throat ailmts	35	30
Tuberculosis	30	17
Bronchial asthma	34	30
Disorders of joints and bones	25	26
Diseases of kidney/urinary system	37	49
Gynaecological disorders	52	50
Neurological disorders	32	32
Psychiatric disorders	10	6
Cataract	29	24
Diabetes mellitus	18	24
Malaria	32	36
Fever of unknown origin	79	67
Locomotor disability	13	9
Accidents/injuries/Burns/etc.	101	88
Cancer and other tumors	28	32
Other diagnosed ailments	164	166
Other undiagnosed ailments	19	15
any ailment	1000	1000

* ailments with at least 1 % share are only listed separately.

apart from the 'other diagnosed ailments' that account for nearly 17 per cent of the hospitalisation cases, the proportions of cases of hospitalisation due to accidents, injuries or burns were the highest among the 'ailment types' considered. They formed about one-tenth of the total cases of hospitalisation. Other ailments with relatively high proportion of cases of hospitalisation were 'fevers of unknown origin' (8 per cent), 'diarrhoea/ dysentery' (7 per cent), 'heart disease' and 'gynaecological disorders' (5 per cent each). There is not much of rural-urban difference in the proportion of hospitalisation cases within each ailment, except for the heart diseases, where the proportion of hospitalisation cases in the urban areas was almost double that in the rural areas.

3.5.6 *Hospitalised Treatment and Type of Hospital*: Statement 24 gives the share of government and private institutions in treating the hospitalised cases of ailments in the rural and urban areas of the country. As in case of non-hospitalised treatment of ailments, here too it was the private institutions that were the main provider of inpatient health care both in the rural

For peer review only - http://bmjopen.bmj.com/site/about/guidelines.xhtml

and urban areas. It is seen that the private institutions dominate the field in treating the inpatients and in 2004, about 58 and 62 per cent of the hospitalised cases, in the rural and urban areas, respectively, were treated by the non-government institutions. Note that the role of government and non-government institutions has reversed between the periods 1986-87 and 2004; about 60 per cent of the hospitalised cases were treated by the government institutions in 1986-87. A steady decline in the use of Government sources and a corresponding increase in the use of private sources over the last three NSS rounds are also evident. The changes were, however, sharper during the period between 1986-87 and 1995-96 rather than between 1995-96 and 2004. The share of the public provider and govt. provider in hospitalised treatments is also shown in Figure 5, separately for rural and urban areas.

Statement 24
Per 1000 distribution of cases of hospitalised treatment by type of hospital during 2004, 1995-96 and 1986-87

India

type of hospital	**rural**			**urban**		
	2004 (*60th*)	1995-96 (*52nd*)	1986-87 (*42nd*)	2004 (*60th*)	1995-96 (*52nd*)	1986-87 (*42nd*)
government	417	438	597	382	431	603
non-government	583	562	403	618	569	397
all hospitals	1000	1000	1000	1000	1000	1000

3.5.7 *Share of Public Provider in Hospitalised Treatment --Inter-state Variation*: Statement 24.1 gives the proportion (per 1000) of cases of hospitalised treatment received from public sector and private sector hospitals for rural and urban areas of major states. In most of the states, the proportion for the rural and urban populations differed little. On the whole, in both the sectors, the reliance on the private sector seems to be greater than that on the public sector. But the rural people had to rely more on public sector hospitals compared to the urban people.

Figure 5: Share of public provider in hospitalised treatment – all-India

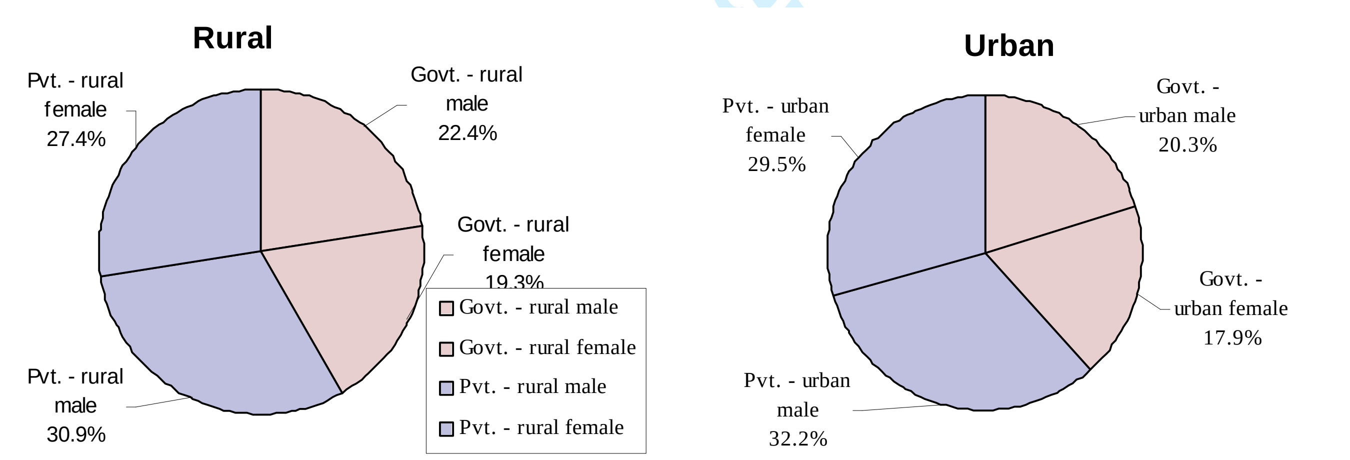

3.5.7.1 The statement indicates that reliance on the public sector for hospitalised treatment varied a great deal from state to state. The proportion (per 1000) of hospitalised treatments received from public sector hospitals varied from 143 (Bihar) to 913 (Jammu & Kashmir) in the rural areas. Apart from Jammu & Kashmir, three other states, viz., Orissa, West Bengal and Himachal Pradesh, reported relatively high proportions of cases of hospitalised treatment from public

For peer review only - http://bmjopen.bmj.com/site/about/guidelines.xhtml

institutions. Bihar, Haryana and Maharashtra showed a high degree of reliance on private sector hospitals.

3.5.7.2 The information on percentage of beds in public and private hospitals in different states, obtained from the Directorate of Health Services, Ministry of Health and Family Welfare of Government of India is also presented in the statement. Ideally, the parity between the share of beds in public institutions and the proportion of hospitalised cases treated in the public institutions should exist if the performance in respect of quality of service and rate of servicing of the two types institutions (public and private) is more or less equal. The deviation, on the other hand, is expected to indicate the differences in the performance of the institutions. Although, the survey results on utilisation of public sector hospitals in different states are seen to be broadly consistent with the data available from the official source, the difference between the two proportions is found be quite pronounced in some states; the proportion of hospitalised cases treated in the public institutions falls short of the proportion of beds in public institutions except three states, viz., Jammu & Kashmir and Kerala where the performance of the public institutions had been better than the private institutions. The states where the performance of public hospitals was very poor were Bihar, Haryana, Karnataka, Maharashtra, Punjab, Tamil Nadu and Uttar Pradesh. However, according to the Directorate, as on 1.1.202, there were beds only in the public sector hospitals in Madhya Pradesh and Rajasthan, but the results of the present survey reveal that a sizable proportion of the hospitalised treatment in these two states were provided by the private sector hospitals.

Statement 24.1
Number (per 1000) of hospitalised cases treated in public hospital and private hospital

major state	treated in hospital #				% of beds in govt. hospitals*
	**rural**		**urban**		
	govt. hosp.	pvt. hosp.	govt. hosp.	pvt. hosp.	
AP	272	727	358	642	40
Assam	742	258	554	446	84
Bihar	144	856	215	785	71
Chhattisgarh	535	465	493	507	-
Delhi	@	@	373	627	64
Gujarat	313	687	261	739	42
Haryana	206	794	290	710	75
HP	781	219	895	105	91
J & K	913	87	865	135	75
Jharkhand	466	534	312	688	-
Karnataka	400	600	289	711	74
Kerala	356	644	346	654	31
MP	585	415	485	515	-
Maharashtra	287	713	280	720	57
Orissa	791	209	731	269	98
Punjab	294	706	264	736	75
Rajasthan	521	479	637	363	-
TN	408	592	372	628	78
Uttaranchal	431	569	342	658	-
UP	269	730	314	686	72
WB	786	213	654	346	86
India	417	583	382	618	62

Based on 'type of hospital' reported cases only.
*: The data relate to 1.1.2002 and are taken from 'Health Information of India' (2003), Central Bureau of Health Intelligence, Directorate General of Health Services, Ministry of Health and Family Welfare, Govt. of India. @ 'not presented"

Statement 25
Average duration of stay (in days) in hospital separately in public and private hospital

India

sector	average no. of days stayed in a hospital	
	type of hospital	
	govt.	private
rural	10.9	8.3
urban	10.8	7.3
rural + urban	10.9	8.0

3.5.8 *Duration of Stay in Hospital*: Statement 25 shows the distribution of hospitalised cases by duration of stay in the

For peer review only - http://bmjopen.bmj.com/site/about/guidelines.xhtml

hospital, separately for the government and private hospitals. The average duration of stay for inpatient care in a hospital during a period of 365 days was shorter in the private hospitals than in the govt. hospitals in both the rural and urban sectors. The average duration of stay in govt. hospital was 11 days and that in a private hospital was only 8 days.

3.5.9 *Treatment Before and After Hospitalisation*: In most of the hospitalisation cases, the ailing person generally undergoes treatment before getting admitted in the hospital and also, when they are discharged from the hospital as a follow-up of the ongoing treatment. The proportion of hospitalisation cases that were treated before hospitalisation and after hospitalisation and the distribution of such cases by the source of their treatment are given in Statement 26. About 55 per cent of hospitalisation cases got treated before hospitalisation, and 76 per cent continued treatment after hospitalisation. However, the average duration of treatment before hospitalisation (98 days) is almost double that of the duration of treatment after hospitalisation (46 days). Among the cases where treatment was taken, the most important source of treatment before hospitalisation was 'private doctors' (48 per cent) while it was 'private hospital' (44 per cent) in the case of treatment after hospitalisation.

Statement 26
Number per 1000 of hospitalisation cases receiving treatment before and after hospitalisation by source of treatment

India

source of treatment	treatment before hospitalisation			treatment after hospitalization		
	**rural**	**urban**	**rural + urban**	**rural**	**urban**	**rural + urban**
public hospital	253	211	239	333	285	317
public dispensary	25	35	28	16	36	23
private hospital	244	263	250	427	465	440
private doctor	477	490	481	222	213	219
all	1000	1000	1000	1000	1000	1000
no. per 1000. of cases receiving treatment	542	569	551	749	782	759
average duration of treatment (0.0 days)	95.7	102.0	97.8	46.1	46.2	46.2

3.6 Cost of Treatment

3.6.1 Cost of Non-hospitalised Treatment

3.6.1.1 In the present survey, data on expenses incurred for medical treatment was collected separately for each case of hospitalisation for hospitalised treatment, but in the case of non-hospitalised treatment, for the ailing person as a whole irrespective of the number of spells and type of ailment or hospitalisation. The 'other expenses' was also recorded separately along with the *medical expenses*. *Medical expenses* included expenditure on items like medicines, bandages, plaster etc., fees paid for medical and para-medical services, charges for diagnostic tests, charges for operation and therapies, charges for ambulance, costs of oxygen and blood, etc. The 'other expenses' constituted all expenses relating to treatment of an ailment incurred by the household in connection with treatment of an ailing member of the household, but other than the medical expenditure proper. This category of expenditure included all transport charges (except ambulance charges) paid by the household members in connection with the treatment, lodging

For peer review only - http://bmjopen.bmj.com/site/about/guidelines.xhtml

charges of the patient and her or his escort(s), attendant charges paid, and personal medical appliances purchased during the reference period. The estimates of 'total expenditure' were arrived at as the sum of 'medical expenditure' and 'other expenditure'.

3.6.1.2 *Average Expenditure for Non-hospitalised Treatment per Ailing Person*: Statement 27 gives the estimates of *medical expenditure* incurred per treated person for non-hospitalised treatment during a period of 15 days. The statement provides separate estimates for treatment of male and female patients in rural and urban areas of the country. It is seen that, on an average, a higher amount was spent for non-hospitalised treatment for an ailing person in the urban areas than that for a ailing person in the rural areas. Secondly, while Rs. 275 to Rs. 322 was spent in a period of 15 days for treatment of an ailing male, a little lesser amount was spent for treatment of a ailing female – cost of treatment being Rs. 240 to Rs. 291.

Statement 27
Average total medical expenditure (Rs.) for non-hospitalised treatment per ailing person during last 15 days

India

gender	**rural**	**urban**
male	275	322
female	240	291
person	257	306

3.6.1.3 *Expenditure for Non-hospitalised Treatment per Treated Person and Level of Living*: Statement 28 gives the *total expenditure* incurred on non-hospitalised treatment per treated person for different monthly per capita consumer expenditure classes. The total *medical* expenditure has been divided into two parts – the part paid to the govt sources and the other to the private sources for availing the total service for treatment of the ailment. As stated before, the *other expenditure* relates to the treatment but not on medical purposes. It is seen that the *total expenditure* incurred on non-hospitalised treatment is broadly positively correlated with levels of living measured in terms of MPCE. The average *total expenditure* for treatment, in urban areas, was Rs. 326 -- much higher than that in the rural areas (Rs. 285). Of these, about 90 per cent were spent on account of *medical treatment* and the rest for other related non-medical expenditure. It may be noted in this context that the data on expenditure were neither collected separately for each spell of ailment nor by source of treatment, but for a treated ailing person as a whole. Thus,

Statement 28
Average total expenditure (Rs.) for non-hospitalised treatment per treated person during the last 15 days for each MPCE class (Rs.)

India

MPCE class	medical expn.: goods & services from		other expen-diture	total expen-diture
	govt. instn.	private instn.		
				**rural**
less than 225	9	163	19	191
225 – 255	8	212	18	238
255 – 300	15	174	21	210
300 – 340	19	190	27	237
340 – 380	12	194	24	229
380 – 420	19	202	28	250
420 – 470	9	211	22	243
470 – 525	13	316	29	358
525 – 615	7	241	28	276
615 – 775	4	263	30	297
775 – 950	11	280	32	323
950 & above	12	377	37	426
all	11	246	27	285
				**urban**
less than 300	4	203	25	231
300 – 350	2	246	10	258
350 – 425	5	185	9	199
425 – 500	5	238	15	259
500 – 575	9	213	14	236
575 – 665	8	244	20	272
665 – 775	8	225	20	253
775 – 915	5	280	18	304
915 – 1120	3	270	17	290
1120 – 1500	4	389	26	418
1500 – 1925	4	352	15	371
1925 & above	17	496	35	548
all	7	299	20	326

For peer review only - http://bmjopen.bmj.com/site/about/guidelines.xhtml

the expenditure shown under 'govt. institution' or 'private institution' gives only the break-up of the average *medical expenditure* incurred for treating a person and not the entire amount of expenditure required to be made for treating a spell of ailment of an ailing person in case the entire treatment had been taken from a single source, i.e., the 'government institution' or 'private institution'. The share of *medical expenditure* of govt. institutions for providing goods and services for treatment was far less than that of the private institutions. This phenomenon is more prominent in the urban areas. One of the reasons may be that the government institutions render a large amount of free or highly subsidised services to the people compared to the private institutions. The other reason is that the role of government institutions is very limited in respect of number of persons treated (only 22–23 per cent of treated spells of ailments get treated from government sources). Note that the share of expenditure on private sources in treatment expenses is seen to be more for households with a higher level of living.

3.6.1.4 *Inter-state Variation in Total Expenditure per Non-hospitalised Ailing Person*: Statements 29R and 29U (pp 40-41) gives the average *medical expenditure* by source of treatment and other related non-medical expenditure per treated person during a period of 15 days for the rural and urban areas of different states. It may be noted that the estimates given in the statement pertain only to the non-hospitalised treatment of ailments. It is seen that the average total expenditure per treated ailment was Rs.285 and Rs.326 in rural and urban areas respectively at the all India level. The medical expenditure per treated ailment varied widely across the states. In the rural areas, it varied from Rs.182 in Kerala to Rs.390 in Rajasthan, and in the urban areas, from Rs.193 in Kerala to Rs. 443 in Jammu & Kashmir. Note that in Kerala, the proportion of cases treated through govt. institutions was also very high. The other states reporting relatively low medical expenditure per treated ailment in the rural areas were Tamil Nadu (Rs.184), Gujarat (Rs.206) and West Bengal (Rs.207). In the urban areas, besides Kerala, relatively low expenditure per treated ailment was reported in Uttaranchal (Rs. 250), Chhattisgarh (Rs.276) and Haryana as well as in Tamil Nadu (Rs.277). Interestingly, contrary to what is observed for most of the states as well as for the country as a whole, Uttar Pradesh, Uttaranchal and Haryana reported a higher medical expenditure per treated ailment in the rural areas than in the urban areas.

3.6.1.5 *Loss of Household Income*: Often ailment of a working member of the household causes loss of household income. Ailment of a non-working member too causes disruption of usual activity of the working member of the household, which in turn results in loss of household income. While for persons getting pay, either as regular salaried employee or casual labour, the amount of loss in income during the period of treatment was derived on the basis of pay that he/she was drawing before the hospitalisation/ailment; for the self-employed persons, it was imputed based on the proportionate average income (lost) during those days. For non-ailing members of the household who could not carry out their 'work' (economic activity) in order to attend to the ailing member, the loss of income for them, if any, was derived in the same manner and was also included in the loss of income of the household. Amount of such a loss incurred by the household during the reference period was collected in the survey. Estimates of loss of household income per treated person as obtained from the survey have also been placed in Statements 29R and 29U (pp 40-41) to get an idea of the total burden on the household due to treatment of ailment. The loss of household income was the highest in Bihar (Rs. 585) followed

For peer review only - http://bmjopen.bmj.com/site/about/guidelines.xhtml

Statement 30
Per 1000 distribution of household total expenditure on treatment during the last 15 days by source of finance for each MPCE class

India

MPCE classes (Rs.)	source of finance			
	hhd income/ savings	borro-wings	others*	total
				**rural**
less than 225	785	134	81	1000
225 – 255	688	214	98	1000
255 – 300	785	176	39	1000
300 – 340	689	262	50	1000
340 – 380	722	164	114	1000
380 – 420	725	196	80	1000
420 – 470	740	177	83	1000
470 – 525	684	279	38	1000
525 – 615	806	147	47	1000
615 – 775	865	106	30	1000
775 – 950	869	116	15	1000
950 or more	794	136	70	1000
all	772	172	56	1000
				**urban**
less than 300	717	244	39	1000
300 – 350	778	127	96	1000
350 – 425	820	147	33	1000
425 – 500	732	201	68	1000
500 – 575	851	116	33	1000
575 – 665	856	125	19	1000
665 – 775	843	99	58	1000
775 – 915	932	38	29	1000
915 – 1120	911	47	42	1000
1120 – 1500	871	60	69	1000
1500 – 1925	964	12	24	1000
1925 or more	862	11	127	1000
all	865	71	64	1000

*include contribution from friends & relatives and sale of ornaments and other physical assets, draught animals, etc.

by Himachal Pradesh (Rs. 200) and Andhra Pradesh (Rs. 175) in the treatment of rural population. This was much higher than the all-India estimate of Rs. 135 as the loss of household income. It was Orissa which had the highest loss of household income (Rs. 887) in treatment of urban population, followed by Jharkhand (Rs. 220), Bihar (Rs. 150) and Andhra Pradesh (Rs. 124), the loss being of Rs. 96 at the all-India level.

3.6.1.6 *Source of Finance for Non-hospitalised Treatment during the last 15 days*: The results relating to the sources from which the household met their expenditure on treatment during the last 15 days are given in Statement 30 in the form of distribution for each household monthly per capita expenditure class for both the rural and urban areas at the all-India level. It can be seen that 77 and 88 per cent of the *total expenditure* for treatment of rural and urban population, respectively was financed by households' own 'income and savings'. This was 17 and 7 per cent in the case of financing by 'borrowing' by the rural and urban households, respectively. As expected, the dependency on own 'income/savings' for financing of expenditure on treatment was more in the case of households with higher levels of living as measured by monthly consumption expenditure.

3.6.2 Cost of Hospitalised Treatment

3.6.2.1 For the hospitalised treatments, information on expenses was collected separately for each event of hospitalisation during the reference period. The expenditure for hospitalised treatment on items such as doctor's fees, bed charges, and cost of medicines and other materials and services supplied by the hospital, as well as charges for diagnostic tests done at the hospital, were included in *medical expenditure*. The 'other expenses' relating to hospitalised treatment is the same as that for non-medical treatments. The estimates of 'total expenditure' for hospitalised treatment were arrived at as the sum of 'medical expenditure' and 'other expenditure'.

Statement 31
Average medical expenditure (Rs.) per hospitalisation

India

gender	rural		urban	
	2004	1995-96	2004	1995-96
male	5,946	3,778	9,535	4,185
female	5,406	2,510	8,112	3,625
person	5,695	3,202	8,851	3,921

Note: The survey period of NSS 60th round was Jan. – June 2004 while that for 52nd was July 1995 – June 1996.

For peer review only - http://bmjopen.bmj.com/site/about/guidelines.xhtml

3.6.2.2 *Average Expenditure for Medical Treatment per Hospitalisation*: Statement 31 gives the estimates of average *medical expenditure* incurred per hospitalised case of treatment during the reference period of 365 days. The statement provides separate estimates for treatment of male and female patients in the rural and urban areas of the country as a whole. It is seen that, on an average, a much higher amount was spent for treatment per hospitalised case by people in the urban (Rs. 8,851) than in the rural (Rs. 5,695). The statement also indicates the presence of a distinct gender bias in the urban areas in respect of expenditure incurred per hospitalisation, though the estimates of proportion of persons hospitalised (given in Statement 21) do not reflect any perceptible gender difference either in rural or urban areas. The average amount spent for treating a female as inpatient in a hospital was less (Rs. 5,406 in the rural and Rs. 8,112 in the urban) than for a male (Rs. 5,946 in the rural and Rs. 9,535 in the urban). Another point to be noted is that the expenditure for inpatient treatment in the hospitals has increased substantially since 1996-97 – from Rs. 3,202 to Rs. 5,695 in the rural areas and from Rs. 3,921 to Rs. 8,851 in the urban areas.

Statement 32
Average medical expenditure (Rs.) per hospitalisation case

India

type of hospital	rural		urban	
	2004	1995-96	2004	1995-96
government hospitals	3,238	2,080	3,877	2,195
private hospitals	7,408	4,300	11,553	5,344
any hospital	5,695	3,202	8,851	3,921

3.6.2.2 *Cost of Treatment per Hospitalisation and Type of Hospital*: Statement 32 gives estimates of average *medical expenditure* incurred for an event of hospitalisation in different types of establishments separately for the rural and urban population of the country. It is seen that the average *medical expenditure* for hospitalised treatment from a public sector hospital was much lower than that from a private sector hospital in both rural and urban areas - less than half in the rural areas and about one-third in urban areas. The rural population spent, on an average, Rs.3,238 for a hospitalised treatment in a public sector hospital and Rs.7,408 for one in a private sector hospital. The average *medical expenditure* of the urban population for a hospitalised treatment in a public and private hospital was, respectively, Rs.3,877 and Rs.11,553. Moreover, compared to the estimate of *medical expenditure* obtained from the 1995-96 (NSS 52nd round) survey, the expenditure showed a steep rise in 2004, for government and the private hospitals. A maximum rise seems to have taken place for the private hospitals in the urban areas, a category that accounts for 62 per cent of hospitalisation cases.

3.6.2.3 *Expenditure on Hospitalisation and Level of Living*: Statement 33 gives the average *medical expenditure* incurred on a case of hospitalisation by households belonging to different classes of monthly per capita consumer expenditure, widely considered to reflect the level of living of a household, separately in government and private hospitals. It is seen that the expenditure incurred on hospitalisation is broadly correlated with levels of living irrespective of type of hospital and sector (rural/urban) and the relationship between them is positive. The relationship seems to be stronger in the rural areas than in the urban areas. A sudden drop in *medical expenditure* on hospitalisation as one moves from the first (lowest) expenditure class to

For peer review only - http://bmjopen.bmj.com/site/about/guidelines.xhtml

Statement 33
Average medical expenditure per hospitalisation by type of hospital for each household MPCE class

India

rural			urban		
MPCE class	av. med. expn. in hosp.		MPCE class	av. med. expn. in hosp.	
	Govt.	Pvt.		Govt.	Pvt.
less than 225	2,530	5,431	*less than 300*	1,746	9,576
225 – 255	3,173	4,886	*300 – 350*	1,640	3,357
255 – 300	2,087	5,543	*350 – 425*	2,594	4,852
300 – 340	2,950	5,777	*425 – 500*	2,690	8,258
340 – 380	2,586	5,245	*500 – 575*	2,365	6,158
380 – 420	2,094	6,895	*575 – 665*	2,984	5,442
420 – 470	2,884	6,028	*665 – 775*	3,011	10,823
470 – 525	3,017	6,781	*775 – 915*	4,304	8,074
525 – 615	3,509	6,811	*915 – 1120*	5,226	10,149
615 – 775	4,049	7,327	*1120 – 1500*	5,402	13,758
775 – 950	4,192	9,843	*1500 – 1925*	8,231	16,433
950 & above	6,374	10,749	*1925 & above*	15,132	22,918
all	3,238	7,408	all	3,877	11,553

the second can be seen in the case of private hospitals in both the sectors. This drop, which is difficult to explain, is more pronounced in the urban sector than in the rural sector. One reason could be that the poorest segment of the population, lacking the right connections, are forced to seek inpatient treatment from the private hospitals in emergencies.

3.6.2.4 *Total Expenditure per Hospitalised Case and Inter-state Variation*: Statement 34 gives the average total expenditure and *medical expenditure* per event of hospitalisation during 365 days by source of treatment for the rural and urban areas. It can be seen that both the average total expenditure and the *medical expenditure* proper per hospitalisation case were almost 50 per cent higher in the urban areas than in the rural areas. The differentials in expenditure between private sector hospitals and public hospitals have already been discussed (in para 3.6.2.2). As expected, the loss in household income due to hospitalisation was more when a male member of the household was hospitalised and was very high in the urban areas. The inter-state variation in the total expenditure is shown in Statements 34.1R and 34.1U (pp 42-43) for rural and urban areas, respectively. Like the non-hospitalised treatment, the cost per hospitalisation in a government establishment was lowest in Tamil Nadu (Rs. 637 in the rural areas and Rs. 1,666 in the urban). In fact, it was relatively low in all the southern states, as well as in Gujarat, Maharashtra and West Bengal. Moreover, on an average, the public hospitals were less expensive than the private sector hospitals in the rural and urban areas of all the states, except urban Haryana and Bihar. The difference between public and private hospitals in average *medical expenditure* for hospitalised treatment received was more pronounced in the urban areas than in the rural areas. The difference in costs of hospitalised treatment from public and private sector hospitals was more

Statement 34
Average medical and other related non-medical expenditure (Rs,) per hospitalisation case during 365 days by source of treatment

India

gender and sector	medical expenditure by source of treatment			other expen-diture	total expen-diture	loss of household income
	Govt.	Pvt.	all			
rural male	3,550	7,640	5,946	550	6,496	760
rural female	2,874	7,145	5,406	508	5,914	493
rural person	3,238	7,408	5,695	530	6,225	636
urban male	4,135	12,448	9,535	545	10,080	1,073
urban female	3,600	10,580	8,112	485	8,596	391
urban person	3,877	11,553	8,851	516	9,367	745

For peer review only - http://bmjopen.bmj.com/site/about/guidelines.xhtml

pronounced in Tamil Nadu and other southern states, as well as in Himachal Pradesh, West Bengal, Maharashtra and Jammu & Kashmir. The highest expenditure for public sector hospitals was reported from rural Haryana and urban Bihar. In the case of private sector hospitals, the highest expenditure was reported in Himachal Pradesh.

3.6.2.5 *Loss of Household Income*: Estimate of loss of household income per treated person as obtained from the survey have also been placed in Statements 34.1R and 34.1U (pp 42-43) to get an idea of the total burden on the households due to hospitalisation. The loss of household income was highest in Himachal Pradesh (Rs. 1,893), followed by Jammu & Kashmir (Rs. 1,377) and Jharkhand (Rs. 1,357) in the rural areas. This was much higher than the all-India estimate of Rs. 636. It was Jharkhand which revealed the highest loss of household income (Rs. 3,971) in the urban areas, followed by Assam (Rs. 1,714) and Andhra Pradesh (Rs. 1,650) - the extent of loss being Rs. 745 at the all-India level.

3.6.2.6 *Source of Finance for Hospitalised Treatment during the last 365 days*: The contributions of different sources towards financing the total expenditure on hospitalisation are

For peer review only

Statement 35
Per 1000 distribution of household total expenditure on treatment on account of hospitalisation during the last 365 days by source of finance for each MPCE class

India

rural					urban				
MPCE classes (Rs.)	source of finance				MPCE classes (Rs.)	source of finance			
	house-hold income/ savings	borro-wings	oth-ers*	total		house-hold income/ savings	borro-wings	oth-ers*	total
less than 225	332	435	233	1000	*less than 300*	541	308	151	1000
225 – 255	265	556	178	1000	*300 – 350*	338	401	262	1000
255 – 300	314	488	198	1000	*350 – 425*	322	334	343	1000
300 – 340	349	462	189	1000	*425 – 500*	345	486	169	1000
340 – 380	379	461	161	1000	*500 – 575*	472	359	169	1000
380 – 420	347	402	251	1000	*575 – 665*	419	407	174	1000
420 – 470	391	451	158	1000	*665 – 775*	573	295	132	1000
470 – 525	399	435	166	1000	*775 – 915*	526	283	191	1000
525 – 615	420	411	169	1000	*915 – 1120*	625	191	184	1000
615 – 775	418	406	176	1000	*1120 – 1500*	585	203	212	1000
775 – 950	419	411	170	1000	*1500 – 1925*	707	116	177	1000
950 or more	485	331	184	1000	*1925 or more*	665	107	229	1000
all	409	411	181	1000	*all*	578	227	195	1000

*include contribution from friends & relatives and sale of ornaments and other physical assets, draught animals, etc.

tabulated in Statement 35 for household at different levels of living as measured by monthly per capita expenditure. A perceptible rural-urban difference is noted in the relative importance of different source categories. While the rural households depended in equal measure on their 'income/saving' and on 'borrowing' – 41 per cent each -- the urban households relied much more on their 'income/saving' (58 per cent) for financing expenditure on hospitalisation, than on 'borrowings' (23 per cent). The households in the lower and middle expenditure classes in the rural areas, however, depended more on 'borrowings', as their 'income/savings', perhaps, were not adequate to meet this expenditure.

For peer review only - http://bmjopen.bmj.com/site/about/guidelines.xhtml

Statement 3.1
Per 1000 distribution of households by structure of the dwelling for each major states

major state	type of structure of the dwelling							
	**rural**				**urban**			
	pucca	semi-pucca	kutcha	total	pucca	semi-pucca	kutcha	total
Andhra Pradesh	616	181	203	1000	864	70	66	1000
Assam	165	433	402	1000	622	311	64	1000
Bihar	431	255	313	1000	825	60	114	1000
Chhattisgarh	91	894	15	1000	706	281	8	1000
Delhi	-	-	-	-	949	24	27	1000
Gujarat	594	349	57	1000	926	57	16	1000
Haryana	940	34	26	1000	975	18	7	1000
Himachal Pradesh	723	251	26	1000	843	89	68	1000
Jammu & Kashmir	352	444	204	1000	714	227	59	1000
Jharkhand	220	564	214	1000	809	170	21	1000
Karnataka	466	473	60	1000	746	224	29	1000
Kerala	730	198	72	1000	847	129	24	1000
Madhya Pradesh	313	588	99	1000	788	196	15	1000
Maharashtra	532	418	50	1000	864	117	19	1000
Orissa	209	289	502	1000	651	153	189	1000
Punjab	840	135	26	1000	913	63	24	1000
Rajasthan	596	214	189	1000	961	29	9	1000
Tamil Nadu	535	217	248	1000	779	119	102	1000
Uttaranchal	837	14	149	1000	968	29	3	1000
Uttar Pradesh	561	244	195	1000	868	88	44	1000
West Bengal	262	469	269	1000	789	166	45	1000
India	480	332	188	1000	842	115	43	1000

For peer review only

For peer review only - http://bmjopen.bmj.com/site/about/guidelines.xhtml

Statement 5.1R

Per 1000 distribution of households by major source of drinking water and number (per 1000) of households treating water before drinking for each major state in rural areas

Rural

major state	major source of drinking water									no. per 1000 treating water before drinking
	bottled water	tap	tube-well/ hand pump	tan-kers	pucca well	tank/ pond reserved for drinking	river/ canal	others	total	
Andhra Pradesh	25	572	279	2	99	9	7	8	1000	243
Assam	0	63	609	3	201	53	33	38	1000	273
Bihar	0	23	925	1	46	1	3	1	1000	226
Chhattisgarh	1	48	763	0	164	6	3	13	1000	138
Gujarat	17	470	386	2	88	33	3	1	1000	727
Haryana	2	445	446	5	94	8	0	0	1000	9
Himachal Pradesh	45	758	56	2	25	15	3	96	1000	35
Jammu & Kashmir	114	493	157	0	12	140	56	27	1000	337
Jharkhand	1	19	473	7	458	1	31	10	1000	387
Karnataka	11	563	307	0	104	8	5	3	1000	982
Kerala	4	107	62	0	766	15	8	37	1000	39
Madhya Pradesh	4	116	625	0	237	4	10	4	1000	8
Maharashtra	37	415	259	48	213	5	14	8	1000	403
Orissa	0	64	718	3	183	5	10	17	1000	497
Punjab	3	302	687	0	4	0	0	5	1000	1
Rajasthan	9	236	493	29	133	76	18	5	1000	7
Tamil Nadu	14	815	100	6	48	8	3	5	1000	35
Uttaranchal	22	614	269	0	1	4	0	91	1000	581
Uttar Pradesh	1	39	883	0	75	1	1	0	1000	563
West Bengal	1	67	854	5	65	0	1	6	1000	184
India	10	248	564	7	139	13	8	10	1000	199

For peer review only - http://bmjopen.bmj.com/site/about/guidelines.xhtml

Statement 5.1U

Per 1000 distribution of households by major source of drinking water and number (per 1000) of households treating water before drinking for each major state in urban areas

Urban

major state	major source of drinking water									no. per 1000 treating water before drinking
	bottled water	tap	tube-well/ hand pump	tan-kers	pucca well	tank/ pond reserved for drinking	river/ canal	others	total	
Andhra Pradesh	17	783	127	48	25	0	0	0	1000	475
Assam	2	321	495	0	164	18	0	0	1000	515
Bihar	0	310	680	0	9	0	0	0	1000	495
Chhattisgarh	4	688	230	7	68	0	3	0	1000	351
Delhi	78	792	80	49	0	0	0	1	1000	142
Gujarat	26	859	91	14	2	7	1	0	1000	820
Haryana	6	752	229	3	11	0	0	0	1000	93
Himachal Pradesh	119	819	53	0	4	1	0	3	1000	178
Jammu & Kashmir	93	859	48	0	0	0	0	0	1000	706
Jharkhand	35	435	331	5	185	2	2	5	1000	555
Karnataka	18	815	117	26	20	0	0	4	1000	969
Kerala	6	347	49	0	591	2	0	5	1000	329
Madhya Pradesh	11	648	278	15	48	0	0	0	1000	61
Maharashtra	38	879	55	0	27	0	0	0	1000	450
Orissa	0	492	278	0	221	0	0	10	1000	898
Punjab	80	682	239	0	0	0	0	0	1000	305
Rajasthan	29	828	99	19	23	0	0	2	1000	47
Tamil Nadu	83	691	58	141	13	3	0	11	1000	188
Uttaranchal	48	894	41	0	0	0	0	18	1000	571
Uttar Pradesh	3	407	585	0	6	0	0	0	1000	412
West Bengal	9	575	398	3	12	0	0	3	1000	621
India	28	676	224	23	44	2	0	3	1000	375

For peer review only

For peer review only - http://bmjopen.bmj.com/site/about/guidelines.xhtml

Statement 21.1
Number (per 1000) of persons hospitalised for each broad age-groups in major states

major state	rural						urban					
	age group (in years)											
	0-14	15-29	30-44	45-59	60 & above	all	0-14	15-29	30-44	45-59	60 & above	all
Andhra Pradesh	12	19	23	39	46	22	17	20	26	51	94	28
Assam	18	24	29	39	108	29	25	12	36	32	66	26
Bihar	7	8	15	16	30	11	10	10	15	27	73	16
Chhattisgarh	8	11	12	21	16	12	19	20	29	47	44	27
Delhi	-	-	-	-	-	-	5	12	6	14	55	11
Gujarat	14	24	27	54	78	29	29	25	34	47	104	36
Haryana	23	25	30	57	70	32	18	30	30	34	98	31
HP	13	25	44	50	66	32	9	38	21	69	136	31
Jammu & Kashmir	9	4	10	4	4	7	20	16	26	27	48	21
Jharkhand	19	20	11	19	9	17	29	25	25	27	64	28
Karnataka	19	5	7	15	29	13	15	12	12	29	0	15
Kerala	14	21	27	37	36	23	23	20	34	47	58	30
Madhya Pradesh	17	27	31	48	63	30	20	16	40	45	81	30
Maharashtra	9	16	22	33	40	18	18	16	25	35	85	25
Orissa	9	13	16	25	64	16	10	14	19	23	41	16
Punjab	6	20	20	26	42	17	12	16	13	43	41	19
Rajasthan	7	11	20	22	27	13	12	16	22	33	70	20
Tamil Nadu	17	22	22	28	46	23	24	21	30	51	92	35
Uttaranchal	44	62	48	105	177	65	30	29	34	157	131	46
Uttar Pradesh	16	110	15	26	57	48	16	17	40	61	112	28
West Bengal	12	23	29	71	22	24	45	37	7	29	73	34
**India**	12	19	25	39	56	23	20	21	29	48	91	31

'-' indicates, not presented cases.

For peer review only

For peer review only - http://bmjopen.bmj.com/site/about/guidelines.xhtml

Statement 29R

Average medical and other related non-medical expenditure per treated person during 15 days by source of treatment

(in Rs.) Rural

major state	medical expenditure by source of treatment: Govt.	Pvt.	all	other expen-diture	total expen-diture	loss of household income per treated person
Andhra Pradesh	6	232	238	24	261	175
Assam	14	212	226	35	261	69
Bihar	42	263	305	47	351	585
Chhattisgarh	37	194	231	32	263	144
Gujarat	1	205	206	36	242	85
Haryana	1	295	296	21	317	77
Himachal Pradesh	4	366	371	46	417	200
Jammu & Kashmir	13	340	352	42	394	151
Jharkhand	36	319	356	14	370	121
Karnataka	4	271	275	38	313	83
Kerala	3	179	182	16	198	72
Madhya Pradesh	4	220	225	30	255	96
Maharashtra	12	223	235	31	266	112
Orissa	25	192	217	21	238	133
Punjab	3	223	227	14	240	45
Rajasthan	4	386	390	46	436	99
Tamil Nadu	0	183	184	24	208	77
Uttaranchal	0	452	453	98	551	51
Uttar Pradesh	10	326	336	27	363	152
West Bengal	20	187	207	17	225	98
India	11	246	257	27	285	135

For peer review only - http://bmjopen.bmj.com/site/about/guidelines.xhtml

Statement 29U

Average medical and other related non-medical expenditure per treated person during 15 days by source of treatment

(in Rs.) Urban

major state	medical expenditure by source of treatment			other expen-diture	total expen-diture	loss of household income per treated person
	Govt.	Pvt.	all			
Andhra Pradesh	2	345	347	22	368	124
Assam	43	308	351	34	385	116
Bihar	14	303	317	55	372	150
Chhattisgarh	4	272	276	15	291	66
Delhi	11	381	392	11	403	78
Gujarat	1	398	399	29	428	69
Haryana	1	276	277	20	298	96
Himachal Pradesh	1	339	340	14	354	37
Jammu & Kashmir	4	438	443	35	478	157
Jharkhand	8	377	385	25	410	220
Karnataka	14	301	315	20	336	83
Kerala	5	189	193	13	206	83
Madhya Pradesh	3	320	323	33	356	121
Maharashtra	2	300	302	14	316	61
Orissa	38	242	280	34	313	887
Punjab	1	347	348	17	366	125
Rajasthan	4	310	314	15	328	61
Tamil Nadu	17	260	277	24	301	48
Uttaranchal	0	250	250	15	266	16
Uttar Pradesh	9	303	312	22	334	117
West Bengal	5	301	306	19	325	77
India	7	299	306	20	326	96

For peer review only

For peer review only - http://bmjopen.bmj.com/site/about/guidelines.xhtml

Statement 34.1R
Average medical and total expenditure per hospitalisation case by type of hospital and loss of household income due to hospitalisation in major states
(in Rs.) Rural

major state	medical expenditure by source of treatment			other expen-diture	total expen-diture	loss of household income
	Govt.	Pvt.	all			
Andhra Pradesh	2,176	6,794	5,574	663	6,237	805
Assam	3,157	8,179	4,195	502	4,697	1,025
Bihar	4,998	6,949	6,655	758	7,413	1,008
Chhattisgarh	4,038	6,086	5,003	430	5,433	711
Gujarat	2,253	6,789	5,408	449	5,857	442
Haryana	11,665	7,147	8,006	541	8,548	654
Himachal Pradesh	6,035	14,652	7,984	883	8,867	1,893
Jammu & Kashmir	4,463	10,145	4,967	698	5,666	1,377
Jharkhand	2,961	6,214	4,799	539	5,338	1,357
Karnataka	2,610	7,918	5,800	471	6,271	530
Kerala	2,174	4,565	3,717	342	4,059	431
Madhya Pradesh	3,238	6,185	4,486	522	5,008	836
Maharashtra	2,243	7,094	5,709	451	6,160	535
Orissa	3,096	7,713	4,089	537	4,625	582
Punjab	9,774	13,044	12,132	623	12,755	589
Rajasthan	5,464	9,540	7,453	840	8,294	846
Tamil Nadu	637	8,360	5,238	537	5,775	369
Uttaranchal	5,166	12,544	9,486	1,245	10,731	1,224
Uttar Pradesh	7,648	9,169	8,765	652	9,417	920
West Bengal	2,464	10,339	4,149	433	4,582	386
India	3,238	7,408	5,695	530	6,225	636

For peer review only - http://bmjopen.bmj.com/site/about/guidelines.xhtml

Statement 34.1U
Average medical and total expenditure per hospitalisation case by type of hospital and loss of household income due to hospitalisation in major states
(in Rs.) Urban

major state	medical expenditure by source of treatment			other expen-diture	total expen-diture	loss of household income
	Govt.	Pvt.	all			
Andhra Pradesh	1,450	13,036	9,197	887	10,085	1,650
Assam	2,696	20,048	10,467	991	11,459	1,714
Bihar	30,822	11,807	14,674	1,033	15,708	1,566
Chhattisgarh	4,244	4,359	4,317	337	4,655	227
Delhi	3,847	14,065	10,568	338	10,906	504
Gujarat	4,358	9,448	8,303	485	8,788	649
Haryana	20,372	11,148	13,626	618	14,244	828
Himachal Pradesh	5,590	23,447	7,649	651	8,300	1,607
Jammu & Kashmir	4,383	17,822	6,122	810	6,931	1,574
Jharkhand	3,716	8,434	7,375	602	7,977	3,971
Karnataka	1,660	9,837	7,552	438	7,990	790
Kerala	2,600	6,179	4,954	247	5,201	578
Madhya Pradesh	2,602	8,661	5,772	1,004	6,775	968
Maharashtra	3,297	11,618	9,776	338	10,114	668
Orissa	4,906	11,020	6,660	634	7,294	713
Punjab	10,323	19,035	16,728	807	17,535	728
Rajasthan	5,590	10,559	7,483	528	8,012	692
Tamil Nadu	1,666	15,680	10,747	559	11,306	367
Uttaranchal	4,083	19,861	14,925	513	15,438	450
Uttar Pradesh	5,144	10,351	8,907	342	9,250	536
West Bengal	4,312	16,025	8,715	510	9,224	529
India	3,877	11,553	8,851	516	9,367	745

For peer review only - http://bmjopen.bmj.com/site/about/guidelines.xhtml

Chapter Four

Immunisation and Maternity Health Care

4.1 General

4.1.1 As already discussed in Chapter One, the survey results on immunisation and maternity health care services received and expenditure incurred to avail these services have been discussed in this chapter. Information on immunisation was collected through a set of questions for children belonging to the age-group 0-4 years (see block [11] of Schedule 25.0 in Appendix C). The information relating to maternity health care that included pregnancy status, childbirth, ante-natal and post-natal care were collected from the ever married women below 50 years of age.

4.2 Immunisation of Children of Age 0 – 4

4.2.1 *Immunisation Rate*: A child was considered to have received immunisation if he/she had received any of the vaccinations viz. BCG, Measles, DPT (any of the 3 doses), Polio (excluding Polio 0 which is given at the time of birth), Hepatitis vaccine (A or B), MMR, Pneumovax (for Pneumonia) and Oral Typhoid. Statement 36 gives the number per 1000 children of age 0 – 4 yrs. who received any immunisation during the last 365 days. It may be noted that a child who received only a part of an immunisation package was also considered to have received immunisation for the purpose of the survey. It can be seen that 90 per cent of the children had received some immunisation during this period. There is a rural-urban differential of about 5 percentage points in these rates; about 94 per cent of children, in the urban areas, received immunisation as against 89 per cent in the rural areas. This difference is in all probability due to lower availability of adequate health facilities and awareness of the people in rural areas compared to the urban. The estimates, however, do not show any gender differential in the rates. The corresponding estimates of immunisation rate (per 1000) by State are given in Statement 36.1 (p. 49).

Statement 36
Number (per 1000) children of age 0–4 years receiving any immunisation

India

gender	number (per 1000) receiving any immunisation		
	rural	urban	rural + urban
boys	893	934	902
girls	892	937	900
children	892	936	901

4.2.2 *Expenditure Incurred on Immunisation and Levels of Living*: Average expen-diture incurred by the households for any immunisation of children in the age-group 0–4 years is given for different levels of living in terms of household monthly capita expenditure class in Statement 37. A wide gap in the average expenditure for immunisation of children is noticed between the rural and urban areas. On an average, around Rs. 20 was spent for immunisation of a rural child and Rs. 113, nearly 6 times as much for an urban child. The reason could be that although the government provides free immunisation services in both rural and urban areas, there is a preference among a sizeable section of the urban population to rely on private doctors, known to them recommended by others, for immunisation of their children. This phenomenon may also be there in the case of relatively richer households in the rural areas. It is interesting to note the gender differential in the expenditure on immunisation in both the rural and urban areas. While in

For peer review only - http://bmjopen.bmj.com/site/about/guidelines.xhtml

the rural areas, the people spent more money on immunisation of a male child than a girl child, it was the reverse in the urban areas, with the exception in the lowest expenditure class. The state-level estimates on average expenditure are given in Statement 37.1 (p.50). The rate of immunisation is also found to be very high in both the sectors of all the states.

Statement 37
Average expenditure per child of age 0 – 4 years on immunisation in each household MPCE class

India

Rural				Urban			
MPCE classes (Rs.)	average expenditure on immunisation (Rs.)			MPCE classes (Rs.)	average expenditure on immunisation (Rs.)		
	boys	girls	children		boys	girls	children
less than 225	4.1	5.2	4.7	*less than 300*	36.9	6.2	21.4
225 – 255	12.1	11.5	11.8	*300 – 350*	18.2	19.5	18.8
255 – 300	12.0	12.9	12.4	*350 – 425*	26.8	12.4	20.2
300 – 340	8.8	9.5	9.1	*425 – 500*	18.0	19.5	18.8
340 – 380	9.5	13.3	11.3	*500 – 575*	42.6	26.0	34.5
380 – 420	17.6	12.2	14.9	*575 – 665*	41.1	65.0	52.2
420 – 470	15.0	17.8	16.4	*665 – 775*	67.0	65.5	66.3
470 – 525	25.9	17.7	21.8	*775 – 915*	85.3	104.5	94.0
525 – 615	31.0	20.5	26.0	*915 – 1120*	178.6	148.2	165.2
615 – 775	34.0	28.5	31.4	*1120 – 1500*	207.5	359.6	275.6
775 – 950	79.9	53.1	68.7	*1500 – 1925*	389.8	447.7	414.6
950 or more	142.0	93.7	120.6	*1925 or more*	615.8	796.2	686.6
all	22.3	17.4	19.9	*all*	109.2	118.3	113.4

4.3 Incidence of Pregnancy, Childbirth and Maternity Care

4.3.1 *Incidence of pregnancy of women of age 15-49 years and loss of pregnancy*: In order to provide estimates of expenditure for antenatal and post-natal care of women of age 15-49 years and expenditure on childbirth, information on the relevant aspects was collected for latest case of pregnancy of women any time during a period of 365 days preceding the date of survey and importantly, from those among them who had given birth. Estimates on proportion of **w**omen who were **p**regnant **a**ny **t**ime (WPAT) during the 365 days prior to the date of survey and 'wastage of pregnancy' (per 1000) are given in Statement 38 for five-year age groups of women. Wastage of pregnancy (WOP) is defined as the ratio of 'total number of pregnancies which did not result into childbirth during the reference period' and 'total number of pregnancies during the same period'. In deriving this indicator, one needs to know - if a woman was pregnant anytime during a reference period, and if so, number of times pregnant during that period and the number of chilbirths given during that period. In this survey, information in respect of 'number of times pregnant during that period' was not collected. If we assume that women who were pregnant during anytime during the reference period (365 days preceding the date of interview) were pregnant only once, we can approximate WOP from the survey data. The statement also gives the distribution of childbirth by place of birth (home/govt. hospital/private hospital). It may be noted that for calculation of 'wastage of pregnancy', it is assumed that the women who were pregnant during the reference period were pregnant once only as the number of pregnancies was not recorded for them by the survey. In that sense, the estimate of 'wastage of pregnancy' is assumed to be the crude one. It can be seen that among women in the age group 15–49 years, about 13 per cent of in the rural areas and 11 per cent in the urban areas were pregnant during the 365 days preceding the date of

For peer review only - http://bmjopen.bmj.com/site/about/guidelines.xhtml

survey. It is important to note that these rates are much lower than the birth rates in the rural and urban areas of the country.

This suggests that there was, perhaps, a gross under-reporting of the events of pregnancy in both the sectors. However, the incidence of pregnancy is found to be highest, as expected, in the age group 20-24 years – the rate being 29 (rural) to 32 (urban) per cent. The incidence of pregnancy diminishes as the age of women advances gradually to 49 years, and is found to reach 1 per cent only in the terminating age group. Secondly, about 72 per cent of the pregnant women in the rural areas delivered a child, while about 69 per cent did so in the urban areas. In other words, about 28 per cent of the pregnancies were wasted in the rural areas, and the percentage was a little higher in the urban areas (31 per cent). Wastage of pregnancy is highest in the age group 15-19 years, and it gradually decreases with the advance of age. Another point may be noted that the rate is, in general, higher for all the age groups in urban areas than that in rural areas. It may be noted that the number of sample count reporting pregnancy in the last two age groups was very small and hence the estimates may not be reliable.

Statement 38
Number (per 1000) of **w**omen aged 15 – 49 years who were **p**regnant **a**ny **t**ime during last 365 days (WPAT), per 1000 **w**astage **o**f **p**regnancy (WOP) and per 1000 distribution of childbirth by place of delivery for each broad age group

India

age group	WPAT	WOP	per 1000 distri. of child-birth by place of delivery		
			govt. hosp.	pvt. hosp.	home
	**rural**				
15-19	250	377	220	175	605
20-24	289	279	196	197	607
25-29	174	288	194	162	645
30-34	99	236	156	143	702
35-39	45	270	85	62	853
40-44	19	257	135	90	775
45-49	14	239	57	83	861
**all**	**127**	**284**	**182**	**166**	**652**
	**urban**				
15-19	293	445	332	382	286
20-24	323	310	328	404	268
25-29	177	288	309	485	206
30-34	69	267	262	462	276
35-39	23	290	262	332	406
40-44	6	304	75	164	761
45-49	6	244	639	303	58
**all**	**107**	**306**	**311**	**429**	**259**
	**rural+urban**				
15-19	256	387	237	204	560
20-24	296	286	225	245	529
25-29	175	288	222	240	538
30-34	91	242	175	202	623
35-39	39	272	114	106	780
40-44	15	263	128	99	773
45-49	11	240	138	113	749
**all**	**122**	**289**	**210**	**222**	**568**

4.3.2 *Institutional Childbirth*: Statement 39 also gives the distribution of childbirth by place of delivery separately for the rural and urban areas at the all India level. In the rural areas, about 65 per cent of the childbirths were non-institutional, that is, at home or any other place other than the hospitals. At the other end, the proportion of non-institutional child-births was 26 per cent in

Statement 39
Per 1000 distribution of childbirth by place of delivery during 2004 and 1995-96

India

place of delivery	rural			urban		
	2004 (*60th*)	1995-96 (*52nd*)	1986-87 (*42nd*)	2004 (*60th*)	1995-96 (*52nd*)	1986-87 (*42nd*)
Govt. hospital	183	-	89	310	-	290
private hospital	166	-	46	429	-	192
hospital	359	179	135	739	594	482
home*	651	787	805	261	384	468
all#	1000	1000	1000	1000	1000	1000

* include 'other places', other than 'hospital' for NSS 52nd Round.

# include 'not recorded' cases in NSS 52nd and 42nd rounds.

For peer review only - http://bmjopen.bmj.com/site/about/guidelines.xhtml

the urban areas. The share of govt. hospitals in the case of institutional births was 31 per cent in the urban areas and only 18 per cent in the rural areas. This share, for the country as whole was 21 per cent (see Statement 38). The increase in the share of the institutional childbirth had been phenomenal. In the rural areas, it increased from 14 per cent in 1986-87 to 18 per cent in 1995-96 and subsequently to 36 per cent in 2004; while the increase, in the urban areas, was from 48 per cent to 59 per cent during the period from 1986-87 to 1995-96, and from 59 per cent to 74 per cent during the period from 1995-96 to 2004. A sharp rise in the share of private institutions in childbirth can also be seen during 1986-87 and 2004 – increased from 5 to 17 per cent in the rural areas and 19 to 43 per cent in the urban areas.

4.3.3 *Expenditure on Childbirth during the last 365 days*: Statement 40 gives the expenditure incurred per childbirth separately for the rural and urban areas. An average of Rs. 1,521 was spent per childbirth during January-June, 2004. There was a perceptible difference in the expenditure incurred for childbirth between the rural and urban areas. For childbirth, this amount was Rs. 1,169 and Rs. 2,806 in the rural and urban, respectively. Again, the cost of a child delivery in private hospital was as high as Rs. 4,692 as compared to Rs. 1,111 in a government hospital. On the other hand, the average cost for delivery of a child at home was only Rs. 428 – the expenditure being Rs. 414 in the rural areas and Rs. 552 in the urban areas. It may be noted that the average expenditure per childbirth was higher in the rural govt. hospital (Rs. 1,165) than their urban counterpart (Rs. 994). Although the instructions were to record the total expenditure on childbirth, including expenditure incurred on treatment of any complication arising at the time of childbirth, it is possible that all other expenditures made in connection with the childbirth, such as, those on travelling, lodging, etc., had also been included while reporting this item, and the rural mothers might have incurred more of such expenditure than the urban mothers due to non-availability of adequate health infrastructure in the rural areas. The corresponding estimate for the major states is given in Statement 40.1 (p. 51).

Statement 40
Average expenditure (Rs.) per childbirth by place of delivery

India

sector	average expenditure on childbirth (Rs.)			
	govt. hosp.	pvt. hosp.	home	all
rural	1,165	4,137	414	1,169
urban	994	5,480	552	2,806
rural+ urban	1,111	4,692	428	1,519

Statement 41
Number (per 1000) of women* who availed antenatal care services (PWANC), post-natal care services (PWPNC) and proportion of these services availed from government and private sources

India

sector	PWANC	source		PWANC	source	
		govt.	pvt.		govt.	pvt.
	**antenatal care**			**post-natal care**		
rural	698	609	391	626	449	551
urban	836	462	538	729	422	578

* aged 15 – 49 years & pregnant anytime during the last 365 days

4.3.4 *Antenatal and Post-natal care – by Source*: Information on maternal care taken by women who were pregnant anytime during the last 365 days was collected in the survey together with the

For peer review only - http://bmjopen.bmj.com/site/about/guidelines.xhtml

expenditure incurred for availing ante-natal and post-natal services. Statement 41 gives the proportion of such women availing these services by the source of availing such services. It is seen that about 70 per cent of pregnant women had taken some antenatal care in the rural areas, and the proportion was much higher -- 84 per cent -- in the urban areas. Compared to the antenatal care, on the other hand, the incidence of availing services for post-natal care was not very common among the women giving childbirth, both in the rural as well as in the urban areas. About 63 per cent in the rural areas and 73 per cent in the urban areas availed of some post-natal care services. Important to note that the private institutions played a major role in providing the maternal care services except in the case of antenatal care services in the rural areas, where the govt. institutions played a dominant role.

4.3.5 *Expenditure incurred for availing Maternity Care*: The expenditure incurred for availing maternity care services from different sources is given in Statement 42. Expenditure involved in the case of availing of antenatal care services was more than that of the post–natal care services, irrespective of whether the women resided in the rural or urban area. For a woman, average expenditure on antenatal and post-natal care was, respectively, Rs. 499 and Rs. 404 in the rural areas. The corresponding values in the urban areas were Rs. 906 and Rs. 596. As expected, the antenatal and post-natal care services were found to be much more expensive in the private sources as compared to the government sources. This differential is sharper in the case of antenatal care in the urban areas.

Statement 42
Average expenditure (Rs.) on antenatal care services (ANC), post-natal care services (PNC) by women* by source of service for each broad age group

India

sector	average expenditure on ANC from sources			average expenditure on PNC from sources		
	govt.	pvt.	all	govt.	pvt.	all
**rural**	230	918	499	232	541	402
**urban**	356	1377	905	367	762	595

* aged 15 – 49 years & pregnant anytime during the last 365 days

4.3.6 *State level Estimates of PWANC and PWPNC*: The estimates of PWANC, PWPNC and share of public sources and average expenditure for availing these services are given in Statements 42.1R and 42.1U (pp 52-53) for the rural and urban areas. The proportion of women availing antenatal and post-natal care services were the highest in Kerala, followed by Tamil Nadu and Karnataka in the rural areas. states where proportionately more women availed both the antenatal and post-natal care services from government sources were Himachal Pradesh, Jammu & Kashmir and Chhattisgarh.

For peer review only - http://bmjopen.bmj.com/site/about/guidelines.xhtml

Statement 36.1

Percentage of children of age 0 – 4 years receiving immunisation in each major state

major state	percentage receiving any immunisation								
	**rural**			**urban**			**rural + urban**		
	boys	girls	children	boys	girls	children	boys	girls	children
Andhra Pradesh	97	95	96	96	96	96	97	95	96
Assam	84	86	85	88	93	90	85	86	85
Bihar	80	78	79	87	92	89	81	79	80
Chhattisgarh	91	87	89	97	98	98	91	88	90
Delhi	-	-	-	94	93	94	-	-	-
Gujarat	90	92	91	95	98	96	92	94	93
Haryana	84	86	85	96	98	96	87	89	87
Himachal Pradesh	94	93	94	89	83	86	94	92	93
Jammu & Kashmir	97	95	96	94	95	95	97	95	96
Jharkhand	88	86	87	87	87	87	88	87	87
Karnataka	98	98	98	98	97	97	98	98	98
Kerala	93	96	95	95	97	96	94	96	95
Madhya Pradesh	88	89	88	89	92	90	88	90	89
Maharashtra	96	95	96	98	97	97	97	96	96
Orissa	94	94	94	100	98	99	95	95	95
Punjab	98	91	94	90	86	88	95	90	92
Rajasthan	93	91	92	96	95	95	94	91	93
Tamil Nadu	97	98	98	96	96	96	97	97	97
Uttaranchal	92	99	94	99	100	100	93	99	95
Uttar Pradesh	84	86	85	88	87	88	85	86	86
West Bengal	93	92	93	93	95	94	93	92	93
India	89	89	89	93	94	94	90	90	90

'-' indicates, not presented cases.

For peer review only

For peer review only - http://bmjopen.bmj.com/site/about/guidelines.xhtml

Statement 37.1
Average expenditure on immunisation per child of age 0 – 4 years receiving immunisation on immunisation in each major state

(in Rs.)

major state	average expenditure on immunisation per child (Rs.)								
	**rural**			**urban**			**rural + urban**		
	boys	girls	children	boys	girls	children	boys	girls	children
Andhra Pradesh	35	37	36	80	102	90	46	53	50
Assam	16	33	25	116	229	172	24	45	35
Bihar	30	34	32	136	96	119	40	39	39
Chhattisgarh	8	13	10	57	52	54	14	18	16
Delhi	-	-	-	359	340	351	-	-	-
Gujarat	12	7	9	148	175	160	56	53	55
Haryana	35	12	24	86	57	74	48	22	36
Himachal Pradesh	4	22	13	1	44	20	4	24	14
Jammu & Kashmir	47	26	36	147	121	135	63	38	51
Jharkhand	24	23	23	129	130	129	37	32	35
Karnataka	20	21	21	64	65	64	33	32	33
Kerala	53	47	50	119	137	128	67	68	68
Madhya Pradesh	9	7	8	58	40	49	19	13	16
Maharashtra	45	9	28	149	214	179	82	81	81
Orissa	7	5	6	68	22	46	14	7	10
Punjab	44	25	34	97	80	90	62	39	51
Rajasthan	3	5	4	16	21	18	5	8	7
Tamil Nadu	47	20	34	121	127	124	71	56	64
Uttaranchal	12	7	10	95	287	172	23	47	33
Uttar Pradesh	12	10	11	56	69	62	19	20	19
West Bengal	6	16	11	162	128	147	37	34	35
India	22	17	20	109	118	113	41	37	39

’-‘ indicates, not presented cases.

For peer review only
For peer review only - http://bmjopen.bmj.com/site/about/guidelines.xhtml

Statement 40.1
Average expenditure per childbirth (Rs.) by place of delivery in major states

(in Rs.)

major state	rural				urban			
	place of delivery				place of delivery			
	Govt. hospital	Pvt. hospital	home	all	Govt. hospital	Pvt. hospital	home	all
Andhra Pradesh	885	3,082	354	1,519	744	5,142	197	2,781
Assam	1,252	1,906	348	754	1,003	6,651	100	3,063
Bihar	2,327	2,187	407	677	1,443	1,813	418	1,144
Chhattisgarh	678	3,342	320	431	1,213	3,784	550	2,049
Delhi	-	-	-	-	889	5,484	790	1,389
Gujarat	1,415	3,221	385	1,329	499	4,803	389	3,067
Haryana	2,786	5,240	400	1,671	1,096	4,520	507	2,351
Himachal Pradesh	3,437	7,833	737	2,752	430	8,226	650	1,691
Jammu & Kashmir	1,401	*	775	1,059	919	7,345	478	1,663
Jharkhand	660	1,446	333	515	2,007	5,208	992	4,096
Karnataka	340	4,141	195	1,305	483	6,937	319	3,152
Kerala	2,088	6,391	*	4,983	1,686	6,172	*	4,906
Madhya Pradesh	1,626	7,186	422	1,285	905	5,624	534	2,583
Maharashtra	633	2,756	320	1,028	828	5,995	364	3,930
Orissa	1,603	2,266	165	609	1,046	6,931	368	1,475
Punjab	3,342	5,770	862	3,269	2,352	5,806	459	2,308
Rajasthan	1,714	3,448	444	901	2,870	6,345	899	3,668
Tamil Nadu	482	5,199	202	1,992	374	6,744	543	3,434
Uttaranchal	1,192	2,000	322	391	1,393	6,638	274	1,234
Uttar Pradesh	1,725	4,008	505	856	1,688	4,361	643	1,851
West Bengal	827	4,370	342	971	1,060	6,551	436	2,386
India	1,165	4,137	414	1,169	994	5,480	552	2,806

’-‘ indicates, not presented cases.; * no sample

For peer review only

For peer review only - http://bmjopen.bmj.com/site/about/guidelines.xhtml

Statement 42.1R

Number (per 1000) of women* who availed (i) antenatal care services (PWANC), (ii) post-natal care services (PWPNC), (iii) proportion of these services availed from government (per 1000) and (iv) average expenditure (Rs. 0.0) on antenatal care services (ANC), post-natal care services (PNC) by women* by source of service for each major state in rural areas

Rural

major state	number per 1000 of women* availing									
	antenatal care services					post-natal care services				
	PWANC	services from govt. sources	average exp. (Rs.)			PWPNC	services from govt. sources	average exp. (Rs.)		
			govt.	pvt.	all			govt.	pvt.	all
Andhra Pradesh	906	518	407	1446	907	719	455	220	476	360
Assam	753	753	167	624	280	703	576	306	401	347
Bihar	530	174	408	473	462	524	198	238	339	319
Chhattisgarh	695	822	94	341	138	574	662	132	151	139
Gujarat	763	674	61	1872	651	503	528	139	1781	915
Haryana	801	754	186	876	356	453	327	244	1245	919
Himachal Pradesh	922	920	437	1912	557	739	881	531	742	556
Jammu & Kashmir	772	830	811	878	822	583	731	535	553	540
Jharkhand	523	412	258	293	278	586	376	104	179	151
Karnataka	876	697	113	948	366	796	647	178	447	273
Kerala	958	294	1337	1492	1446	873	297	784	1388	1209
Madhya Pradesh	574	758	214	1189	450	580	656	308	589	405
Maharashtra	809	587	142	1246	598	609	482	115	485	307
Orissa	775	826	186	752	284	738	670	246	421	304
Punjab	711	520	930	1631	1267	567	443	658	587	618
Rajasthan	706	807	292	883	406	570	479	548	864	713
Tamil Nadu	954	711	116	1730	583	775	697	63	596	224
Uttaranchal	557	801	47	1258	289	231	188	661	98	204
Uttar Pradesh	544	537	109	556	316	642	277	175	395	334
West Bengal	890	670	262	622	381	661	551	130	341	225
India	698	609	230	918	499	626	449	232	541	402

* women aged 15 – 59 years who were pregnant anytime during the last 365 days

For peer review only - http://bmjopen.bmj.com/site/about/guidelines.xhtml

Statement 42.1U

Number (per 1000) of women* who availed (i) antenatal care services (PWANC), (ii) post-natal care services (PWPNC), (iii) proportion of these services availed from government (per 1000) and (iv) average expenditure (Rs. 0.0) on antenatal care services (ANC), post-natal care services (PNC) by women* by source of service for each major state in urban areas

Urban

major state	number per 1000 of women* availing									
	antenatal services					post-natal services				
	PWANC	services from govt. sources	average exp. (Rs.)			PWPNC	services from govt. sources	services from govt. sources		
			govt.	pvt.	all			govt.	pvt.	all
Andhra Pradesh	925	347	630	1614	1272	820	377	341	533	460
Assam	1000	447	412	687	564	953	666	503	1663	889
Bihar	753	80	266	603	576	724	78	522	344	358
Chhattisgarh	872	596	122	734	369	580	487	100	643	379
Delhi	801	605	412	1605	883	809	677	379	1066	600
Gujarat	882	426	337	1568	1043	693	344	243	955	710
Haryana	825	464	306	1141	754	547	417	263	1303	869
Himachal Pradesh	1000	885	127	859	211	877	642	130	291	188
Jammu & Kashmir	911	629	627	2332	1259	821	504	402	1893	1142
Jharkhand	911	168	419	841	770	903	200	500	421	436
Karnataka	920	634	293	1271	651	766	625	236	867	473
Kerala	976	290	1163	1905	1690	830	336	1614	1331	1426
Madhya Pradesh	790	597	334	1180	675	757	658	411	980	608
Maharashtra	863	286	341	1636	1266	763	304	314	779	638
Orissa	889	893	617	916	649	601	831	137	1540	373
Punjab	555	686	384	2282	979	574	729	682	786	710
Rajasthan	874	577	586	1687	1052	698	383	911	1805	1463
Tamil Nadu	986	566	126	1739	826	744	542	130	732	406
Uttaranchal	908	911	204	1594	329	278	495	119	337	229
Uttar Pradesh	640	448	225	665	468	710	321	282	464	406
West Bengal	943	573	379	1332	786	690	493	423	589	507
India	836	462	356	1377	905	729	422	367	762	595

* women aged 15 – 59 years who were pregnant anytime during the last 365 days

For peer review only

For peer review only - http://bmjopen.bmj.com/site/about/guidelines.xhtml

Chapter Five

Condition and Health Care of the Aged

5.1 General

5.1.1 The results relating to the condition and health care of persons aged 60 and above, to be referred to as the aged persons, have been placed and discussed in this Chapter. The results are based on the information collected in the field through a particular module of the survey schedule (see block [6] of Schedule 25.0 given in Appendix C).

5.1.2 As mentioned earlier, one of the objectives of collecting information through this survey was to assess the structure and composition of the aged in respect of age, sex, dependency ratio, etc. and the conditions of the aged in respect of their economic dependency, number of dependants, living arrangements, persons supporting the aged, physical immobility, etc. In the survey, those who were of age 60 years and above were considered aged. Information on number of surviving children, living arrangement, economic independence, number of dependants, persons supporting the aged, etc., was collected for the aged. The results obtained from the survey are presented in this section. The main findings of the survey are discussed at the all-India level. Similar estimates obtained from the earlier survey carried out during 1995-96 (NSS 52nd round), wherever possible, are also placed side by side to give an idea about the changes over this period.

Statement 43
Share (per 1000) of the aged to total population obtained from NSS surveys and population censuses for each sex

India

source	rural			urban		
	male	female	person	male	female	person
Census 1981	68	68	68	51	58	54
NSS 43rd round (1987-88)	65	66	65	54	61	57
Census 1991	78	74	76	62	66	63
NSS 50th round (1993-94)	68	69	69	55	64	60
NSS 52nd round (1995-96)	55	59	57	47	53	50
Census 2001	74	81	77	62	72	67
NSS 60th round (Jan-Jun, 2004)	70	71	70	62	71	66

5.1.3 *Demographic Burden:* It is found from the survey results that of the estimated 66.4 million aged persons in the country, about 75 per cent were residing in the rural areas and remaining 25 per cent in the urban areas. Their magnitude, either in terms of number or share to total population is found to rise gradually The proportion of aged persons, which was 6.5 per cent (43.5 million) in 1981 (as per Population Census), grew to 6.8 per cent (61.4 million) in 1991 and 7.4 per cent (76.6 million) in 2001. Although the number of aged persons obtained from the present survey, was lower than even that of Census 2001, its magnitude in terms of proportion in the total population was fairly close to that of Census. More importantly, the two sets of

For peer review only - http://bmjopen.bmj.com/site/about/guidelines.xhtml

estimates of proportions of the aged, obtained from the surveys and census, reveal similar trend over the time points, except that from NSS 52nd round. Perhaps, there has possibly been some problem of bias in age reporting in that round. This apart, the survey has also revealed that the concentration of aged in terms of its share to total population tends to be higher in the rural areas than in the urban areas - the share being 7.0 and 6.6 per cent, respectively, which is again supported by the Census estimates, as given in Statement 43. Male-female and rural-urban differences in the proportion of aged persons are found to exist and the differences are significant. The share of the aged females was higher than that of the aged males in the urban areas and almost the same in the rural areas. This fact reflects the higher expectancy of life for females and probably the out-migration of the males in the working age groups from the rural areas. The corresponding estimates for major states are given in Statement 43.1 (p 61).

5.1.4 *Old-age Dependency Ratio:* It measures the responsibilities of the aged to the working-age population. In our country, generally, persons aged 15 to 59 years are supposed to form the population of working ages and at age 60, people generally retire or withdraw themselves from work. Thus, the population aged 60 or more divided by the number aged 15 to 59years gives the old-age dependency ratio. The old-age dependency ratios (per 1000) are given in Statement 44. It may be seen that the old-age dependency ratio is higher in the rural than in the urban areas. In the rural areas, every 1000 persons in the working age had to provide support, physically or otherwise, to 125 aged persons, to maintain their daily life. The number was 103, a little less, in the urban areas.

Statement 44
Old-age dependency ratio (per 1000) obtained from NSS surveys and census

India

source	**rural**	**urban**	**rural +urban**
Census 1981	94	71	89
NSS 43rd round (1987-88)	111	88	103
Census 1991	123	96	118
NSS 50th round (1993-94)	108	90	104
NSS 52nd round (1995-96)	92	74	87
Census 2001	141	107	131
NSS 60th round (Jan-Jun, 2004)	125	103	119

According to both the census and NSS survey estimates, the ratio has increased over time except for NSS 52nd round, where it shows a decline. The dip in the proportion of the aged in the 52nd round can most likely be attributed to age-reporting bias. It also shows that the 'burden' of the aged on the working population has increased, substantially, during the last decade. The estimates for the major states, given in Statement 43.1 (p.61) (as also in Figure 5) show that the old-age dependency ratio is the highest in Kerala (with the highest proportion of aged persons) in both the rural and urban areas, followed by Jammu &

Statement 45
Sex ratio (number of females per 1000 males) among aged persons obtained from census and NSS surveys

India

source	**rural**	**urban**	**rural + urban**
Census 1981	954	986	960
NSS 43rd round (1987-88)	971	1032	983
Census 1991	922	960	930
NSS 50th round (1993-94)	963	1060	984
NSS 52nd round (1995-96)	1017	1043	1023
Census 2001	1026	1038	1029
NSS 60th round (Jan-Jun, 2004)	985	1046	999

For peer review only - http://bmjopen.bmj.com/site/about/guidelines.xhtml

Kashmir and Uttaranchal in the rural, and Maharashtra and Uttaranchal in the urban areas.

5.1.5 *Sex Ratio*: According to Population Census, the sex ratio among the aged that was 960 females per 1000 males during 1981, dropped to 930 during 1991 and thereafter increased to 1029 in 2001. The NSS estimates, on the other hand, showed a rising trend between the period 1993-94 and 1995-96, but declined between 1995-96 and 2004. The trend is also somewhat different in the rural and urban areas. In the rural areas, the sex ratio among the aged declined during the period 1987-88 to 1993-94 and rose in 1995-96, has again declined in 2004 (see Statement 46). In urban areas, the sex ratio increased during 1987-88 to 1993-94 and then dropped in 1995-96 and 2004.

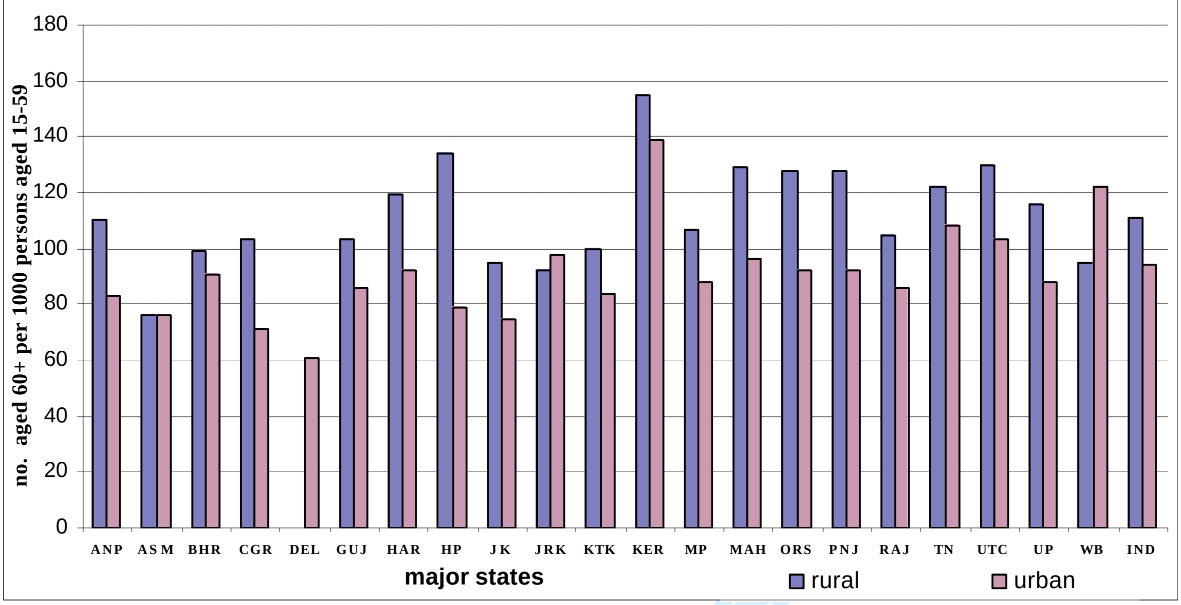

Figure 5: Old-age dependency ratio

5.1.6 *Aged Persons and their Surviving Sons and Daughters:* The extended family system is the dominant form of family in India. In such a system, many of the aged, particularly those who have lost their spouses, depend on their children for maintenance. In Statement 46, the proportion of aged persons by number of their surviving children is given separately for each sex and sector at the all-India level. In India, during January-June 2004, about 94 per cent of the aged had at least one surviving child. In other words, about 6 per cent of the aged had no surviving children on the date of survey. The rural-urban differences appeared to be nil with respect to the proportion of the elderly who had surviving children. However, it was marginally higher for males than for females. The results also indicate that since 1995-96, there has been a little improvement in regard to the proportion of aged persons having their children alive.

For peer review only - http://bmjopen.bmj.com/site/about/guidelines.xhtml

5.1.7 *Living Arrangement:* Besides the number of surviving children, information on living arrangement of the elderly was collected in the survey. The results show that about 57 per cent of the aged were living with their spouses and another 32 per cent were living without their spouses but with their children, while about 4 to 5 per cent were living with other relations and non-relations (see Statement 47). Nevertheless, 4 to 5 per cent were still living alone. An interesting gender-differential is observed in the living arrangement among the elderly and the pattern is similar in both rural and urban areas. In terms of proportions, more males than females lived with their spouses. On the other hand, compared to the males, proportionately more females lived either alone or with their surviving children or lived with other relations and non-relations. Probably, this pattern is the impact of the higher incidence of widowhood among the elderly females than among the elderly males. The incidence of widowhood is higher among women because they live longer, and because in our society, men generally marry women younger than themselves. However, the living arrangement of the aged has not changed much over time since 1995-96, the proportion of the aged living alone remaining almost the same. Between 1995-96 and 2004, the proportion of the aged who lived with their spouses had however,

Statement 46
Proportion (per 1000) of aged persons by number of their surviving children for each sex

India

gender & sector	no. of surviving children: 0	1	2	1 or more
**rural**				
male	53	66	124	947
	(55)	(61)	(105)	(945)
female	56	95	129	944
	(62)	(81)	(120)	(938)
person	55	81	126	945
	(58)	(71)	(113)	(942)
**urban**				
male	49	77	170	951
	(53)	(71)	(112)	(947)
female	66	94	141	934
	(65)	(87)	(111)	(935)
person	58	85	155	942
	(59)	(79)	(112)	(941)
all	55	82	133	945
	(58)	(73)	(112)	(942)

Note: Figures in parenthesis give the corresponding estimates obtained from NSS 52nd Round (1995-96).

Statement 47
Per 1000 distribution of aged persons by type of living arrangement for each sex

India

living arrangement	rural			urban		
	male	female	person	male	female	person
1. alone	28	76	53	21	65	43
	(25)	(61)	(43)	(30)	(60)	(45)
2. with spouse only	162	87	125	133	75	104
	(137)	(77)	(107)	(103)	(57)	(80)
3. with spouse & other members	597	284	442	649	294	468
	(613)	(313)	(462)	(648)	(297)	(469)
4. with spouse (2+3)	759	371	567	782	369	572
	(750)	(390)	(569)	(751)	(354)	(549)
5. with children	168	475	320	154	482	322
	(179)	(481)	(331)	(178)	(512)	(349)
6. with other relations & non-relations	27	56	42	29	67	49
	(38)	(59)	(48)	(35)	(65)	(51)
total (include. n.r.)	1000	1000	1000	1000	1000	1000

Note: Figures in parentheses give the corresponding estimates obtained from NSS 52nd round (1995-96).

For peer review only - http://bmjopen.bmj.com/site/about/guidelines.xhtml

gone up significantly from 55 to 57 per cent in urban areas and remained the same in rural areas. The fall between 1995-96 and 2004 in the proportion of aged persons living with their children reflects the gradual break-down of the extended family system in India in both its rural and urban areas. The estimates for major states are given in Statement 47.1 (pp 62 – 65). It can be seen that more than 15 per cent of females in rural areas of Tamil Nadu and Uttaranchal and urban areas of Chhattisgarh were 'living alone' during the survey period.

Statement 48
Per 1000 distribution of aged persons by state of economic independence for each sex

India

gender	state of economic independence			
	not dependent on others	partially dependent on others	fully dependent on others	total (incl. n.r.)
**rural**				
male	513 (485)	152 (180)	320 (313)	1000
female	139 (121)	124 (146)	720 (706)	1000
person	327 (301)	138 (163)	519 (511)	1000
**urban**				
male	555 (515)	134 (169)	301 (297)	1000
female	170 (115)	95 (110)	721 (757)	1000
person	359 (311)	114 (139)	516 (532)	1000

Note: Figures in parentheses give the corresponding estimates obtained from NSS 52nd Round (1995-96)

5.1.8 *Economic Independence:* The living arrangement describes how the physical well-being of the aged is taken care of in the family in our society. Similarly, the economic independence reveals the associated problem of day-to-day maintenance of livelihood of the elderly. The per 1000 distribution of aged persons by state of economic independence is given in Statement 48 for each sex, separately for rural and urban sectors of India. As many as 65 per cent of the aged had to depend on others for their day-to-day maintenance. The situation was worse for elderly females. Among them, about 85 per cent were economically dependent either partially or fully. In this respect, males were much better off -- 46 to 49 per cent among them did not fully depend on others for their livelihood. Compared to 1995-96, the results of the present survey indicate that the economic condition of the elderly has improved in general and more in the urban sector, both among men and among women.

5.1.9 *Economic Support Providers:* As has been observed, a large proportion of the elderly are economically dependent on others for their livelihood. It is, therefore, pertinent to know who are the persons providing economic support to these elderly. Such information was collected in the survey and the results are presented in Statement 49 separately for each sex and sector at the all-India level. It is seen that of the economically dependent aged, a majority (about 76 to 78 per cent) had to depend on their children and a sizable proportion (13 to 15 per cent) on their spouses for their economic support. Only 3 per cent were supported by their grandchildren and the rest (6 per cent) had to depend on 'others', including non-relations. Between the years 1995-96 and 2004, the distribution of the aged who were economically dependent changed in respect of the category of persons supporting them for their livelihood. The patterns of change are not similar for males and females, but are so for the elderly living in the rural and urban areas. In the inter-survey periods, the proportion of the aged males and females depending on their children for

For peer review only - http://bmjopen.bmj.com/site/about/guidelines.xhtml

economic support has increased in both rural and urban areas and more so in the rural areas. On the other hand, the proportion of those depending on their spouse decreased, in general, among the males but marginally increased among the aged females in the urban areas. The proportion in this category, however, did not show any change between the periods in the rural areas.

Statement 49
Per 1000 distribution of economically dependent aged persons by category of persons supporting the aged for each sex

India

gender	category of persons supporting: spouse	own children	grand-children	others	total
**rural**					
male	70 (113)	850 (766)	22 (50)	57 (71)	1000
female	159 (159)	746 (717)	31 (52)	63 (72)	1000
person	127 (142)	784 (735)	28 (52)	61 (71)	1000
**urban**					
male	60 (105)	865 (792)	18 (54)	57 (49)	1000
female	192 (182)	710 (695)	30 (56)	68 (67)	1000
person	148 (156)	762 (728)	26 (55)	64 (61)	1000

Note: Figures in parentheses give the corresponding estimates obtained from NSS 52nd Round (1996-96).

5.1.10 *Number of Dependants:* While for the economically dependent aged, information on category of persons supporting the aged was collected, information on the number of dependants was collected for the aged who were economically independent. The results have been presented in Statement 50 in the form of distribution of economically independent aged persons by number of dependants. The results are given for each sex and sector at the all-India level. It has been observed earlier in this section that about 33 to 36 per cent of the aged were economically independent. Of them, about 85 to 88 per cent were reported to be living with one or more dependants. In other words, about 12 to 15 per cent had no dependants. The gender differences are quite pronounced in this distribution. The distribution for males appears to have shifted to the right of that for females. This implies that on an average an aged male had more dependants than an aged female during 2004. The pattern of the distribution, however, appears to be the same for both rural and urban areas.

Statement 50
Per 1000 distribution of economically independent aged persons by number of dependants for each sex

India

gender	number of dependants: nil	1	2	3-5	6 or more	total
**rural**						
male	69	321	234	247	130	1000
female	320	175	370	95	40	1000
person	122	290	262	215	111	1000
**urban**						
male	83	396	256	206	59	1000
female	341	146	417	79	17	1000
person	146	335	295	176	49	1000

5.1.11 *Physical Mobility:* For the aged persons the ability to move is an important indicator of their physical condition of health and also indicates the degree of their dependence on others for movement and performing their daily routine. The proportion (number per 1000) of the aged persons who cannot move around and are confined to their home or who cannot move at all and are confined to bed is given in Statement 51. The results are given for each sex and sector at all-India level. About 8 per cent of the aged

For peer review only - http://bmjopen.bmj.com/site/about/guidelines.xhtml

persons were either confined to their home or bed. The proportion of aged persons reporting confinement to their home or bed was found to increase with the age for all categories, being as high as 27 for persons aged 80 or more. The incidence of confinement is seen to be higher among women than among men in both rural and urban areas.

Statement 51

Proportion (number per 1000) of aged persons who cannot move and are confined to bed or home

India

age-group (years)	rural			urban		
	male	female	person	male	female	person
60-64	27	34	31	33	34	33
64-69	51	50	51	34	63	50
70-74	79	132	105	77	116	97
75-79	117	163	139	113	185	147
80 & above	220	326	269	239	323	283
all aged	67	88	77	68	100	84

5.1.12 *Own Perception about Health:* The perception about one's health is an important factor in getting an idea about a person's actual health condition. A person may be considered as being in good health if he feels so. This is the criterion generally used in NSS surveys to classify an individual as sick or otherwise. Moreover, it reflects the mental health of that person. With this idea, information about the perception of aged persons about their current health was collected in the survey and is presented in Statement 52 separately for those with sickness and without it[1]. It can be seen that as high as 55 to 63 per cent of the aged with sickness felt that they were in a good or fair condition of health. The proportion among the aged without sickness was 77 to 78 per cent. Possibly they considered their sickness as a problem of ageing. Among the aged, the men seemed to be feeling that they had a better health condition even with sickness compared to the aged women. As against this, about 13 to 17 per cent of the aged who were not even sick considered themselves as having a 'poor' state of health.

Statement 52

Per 1000 distribution of aged persons by own perception about their health

India

own perception about current state of health	aged persons with sickness			aged persons without sickness		
	male	female	person	male	female	person
**rural**						
excellent / very good	19	14	17	81	43	62
good / fair	580	525	553	772	770	771
poor	401	460	429	147	187	167
all aged	1000	1000	1000	1000	1000	1000
**urban**						
excellent / very good	31	19	24	114	72	92
good / fair	641	620	631	775	780	778
poor	327	360	345	111	148	130
all aged	1000	1000	1000	1000	1000	1000

Note: The proportions have been adjusted excluding the 'not reported cases'.

[1] As part of the survey on morbidity it was ascertained for all the persons in the surveyed households, including the aged, whether they had suffered from any ailment during the last 15 days. This enabled the aged to be classified as 'with sickness' or 'without sickness'.

For peer review only - http://bmjopen.bmj.com/site/about/guidelines.xhtml

Statement 43.1
Number of aged per 1000 persons for each sex and major state

major state	rural				urban			
	no. of aged per 1000 persons			old age dependency ratio	no. of aged per 1000 persons			old age dependency ratio
	male	female	person		male	female	person	
Andhra Pradesh	73	77	75	110	53	64	58	83
Assam	53	43	48	76	54	50	52	76
Bihar	59	52	55	99	62	55	59	91
Chhattisgarh	53	77	65	103	43	57	50	71
Delhi	-	-	-	-	40	49	44	61
Gujarat	67	68	68	128	54	69	61	86
Haryana	72	84	78	103	51	77	63	92
Himachal Pradesh	92	95	94	119	50	52	51	79
Jammu & Kashmir	73	54	64	134	60	50	55	75
Jharkhand	63	48	56	95	68	69	68	98
				92				
Karnataka	71	66	69	100	58	61	59	84
Kerala	106	123	115	155	103	109	106	139
Madhya Pradesh	63	68	65	107	54	64	59	88
Maharashtra	83	88	86	129	65	76	70	96
Orissa	90	80	85	128	73	61	68	92
Punjab	85	88	86	128	62	67	64	92
Rajasthan	59	67	63	105	56	61	58	86
Tamil Nadu	87	85	86	122	73	85	79	108
Uttaranchal	80	73	77	130	61	88	74	103
Uttar Pradesh	64	67	66	116	51	60	56	88
West Bengal	62	64	63	95	90	95	92	122
India	70	71	70	111	62	71	66	94

For peer review only

For peer review only - http://bmjopen.bmj.com/site/about/guidelines.xhtml

Statement 47.1
Per 1000 distribution of aged persons by type of living arrangement for each sex and major state

major state	living arrangement					
	alone	with spouse only	with spouse and other members	with children	with other relations & non-relations	total (include n.r.)
						**rural male**
Andhra Pradesh	37	324	479	138	12	1000
Assam	3	42	733	165	10	1000
Bihar	31	149	599	193	10	1000
Chhattisgarh	37	181	510	204	35	1000
Gujarat	40	182	551	207	14	1000
Haryana	0	70	696	209	23	1000
Himachal Pradesh	42	132	596	181	29	1000
Jammu & Kashmir	0	35	699	208	35	1000
Jharkhand	28	146	537	238	34	1000
Karnataka	21	138	685	129	26	1000
Kerala	13	127	698	137	23	1000
Madhya Pradesh	33	190	545	161	47	1000
Maharashtra	23	184	644	124	21	1000
Orissa	25	157	667	117	9	1000
Punjab	26	105	606	211	39	1000
Rajasthan	19	122	629	174	50	1000
Tamil Nadu	52	302	516	104	27	1000
Uttaranchal	44	123	543	204	11	1000
Uttar Pradesh	34	115	549	224	48	1000
West Bengal	12	108	676	158	17	1000
India	28	162	597	168	27	1000

For peer review only

For peer review only - http://bmjopen.bmj.com/site/about/guidelines.xhtml

Statement 47.1
Per 1000 distribution of aged persons by type of living arrangement for each sex and major state

major state	living arrangement					
	alone	with spouse only	with spouse and other members	with children	with other relations & non-relations	total (include n.r.)
						**rural female**
Andhra Pradesh	144	134	151	478	82	1000
Assam	64	32	212	626	14	1000
Bihar	53	90	390	416	21	1000
Chhattisgarh	118	87	212	498	63	1000
Gujarat	88	113	282	494	21	1000
Haryana	8	56	519	383	34	1000
Himachal Pradesh	40	72	240	532	84	1000
Jammu & Kashmir	9	36	435	486	27	1000
Jharkhand	48	60	360	496	16	1000
Karnataka	97	65	195	552	81	1000
Kerala	42	56	281	539	67	1000
Madhya Pradesh	83	129	294	408	58	1000
Maharashtra	105	97	301	415	73	1000
Orissa	44	77	300	504	43	1000
Punjab	37	93	406	443	19	1000
Rajasthan	45	78	312	523	42	1000
Tamil Nadu	194	110	204	391	100	1000
Uttaranchal	188	86	286	399	30	1000
Uttar Pradesh	51	85	343	445	49	1000
West Bengal	57	43	174	624	61	1000
India	80	87	284	475	56	1000

For peer review only - http://bmjopen.bmj.com/site/about/guidelines.xhtml

Statement 47.1
Per 1000 distribution of aged persons by type of living arrangement for each sex and major state

major state	living arrangement					
	alone	with spouse only	with spouse and other members	with children	with other relations & non-relations	total (include n.r.)
						**urban male**
Andhra Pradesh	27	215	579	150	25	1000
Assam	10	25	800	141	21	1000
Bihar	7	55	610	248	11	1000
Chhattisgarh	12	37	711	240	0	1000
Delhi	18	125	646	188	10	1000
Gujarat	9	139	710	136	7	1000
Haryana	0	137	663	180	13	1000
Himachal Pradesh	0	149	634	168	49	1000
Jammu & Kashmir	4	133	655	183	19	1000
Jharkhand	43	44	712	159	5	1000
Karnataka	8	96	729	141	16	1000
Kerala	7	168	695	93	36	1000
Madhya Pradesh	20	103	649	169	38	1000
Maharashtra	17	130	653	176	15	1000
Orissa	12	166	650	133	12	1000
Punjab	21	96	732	109	17	1000
Rajasthan	24	112	643	190	29	1000
Tamil Nadu	35	221	587	121	36	1000
Uttaranchal	0	200	586	202	13	1000
Uttar Pradesh	33	112	601	203	27	1000
West Bengal	27	106	664	102	80	1000
India	21	133	649	154	29	1000

For peer review only - http://bmjopen.bmj.com/site/about/guidelines.xhtml

Statement 47.1
Per 1000 distribution of aged persons by type of living arrangement for each sex and major state

major state	living arrangement					
	alone	with spouse only	with spouse and other members	with children	with other relations & non-relations	total (include n.r.)
						**urban female**
Andhra Pradesh	91	95	194	537	67	1000
Assam	0	0	328	544	30	1000
Bihar	47	87	431	359	31	1000
Chhattisgarh	168	11	247	469	105	1000
Delhi	59	83	362	463	21	1000
Gujarat	70	89	301	487	44	1000
Haryana	61	87	343	459	17	1000
Himachal Pradesh	0	61	236	680	14	1000
Jammu & Kashmir	24	117	325	522	0	1000
Jharkhand	31	39	365	482	29	1000
Karnataka	72	31	209	612	71	1000
Kerala	59	64	201	539	121	1000
Madhya Pradesh	65	52	313	462	76	1000
Maharashtra	67	83	303	482	53	1000
Orissa	38	59	388	434	40	1000
Punjab	25	68	497	387	13	1000
Rajasthan	61	81	353	468	36	1000
Tamil Nadu	120	99	199	480	100	1000
Uttaranchal	43	98	236	582	10	1000
Uttar Pradesh	34	79	363	440	63	1000
West Bengal	37	61	308	462	113	1000
India	65	75	294	482	67	1000

For peer review only - http://bmjopen.bmj.com/site/about/guidelines.xhtml

Appendix A

Detailed Tables

For peer review only

Description of terms used in detailed tables

**ONES:**	**Other North-Eastern States (except Assam)**
**GUTs:**	**Group of Union Territories**
**SC:**	**Scheduled Caste**
**ST:**	**Scheduled Tribe**
**OBC:**	**Other Backward Classes**
**hosp:**	**hospitalisation**
**n.r.:**	**not recorded (cases)**
**estd. :**	**estimated**

Appendix A

Part A1

Morbidity and Hospitalisation

Detailed Tables

For peer review only

Appendix A

Table (1): Number of villages/blocks, households, persons, aged (60 years and above) persons surveyed, number of hospitalised and ailing persons surveyed and average household size, separately for each State/UT

Rural

state/ut	number of surveyed				during last 365 days		sample persons reporting ailment during last 15 days	estimated (00)				
	villages / blocks	house-holds	persons	aged persons	sample persons hospita-lised	sample cases of hospi-talisa-tion		house-holds	persons	cases of hospita-lisation during last 365 days	aged persons	persons reportting ailment during last 15 days
(1)	(2)	(3)	(4)	(5)	(6)	(7)	(8)	(9)	(10)	(11)	(12)	(13)
Andhra Pradesh	325	3235	14220	1385	1302	1424	1616	127106	518887	12166	38765	46666
Arunachal Pradesh	75	732	4025	258	239	241	242	1607	7825	234	442	480
Assam	215	2150	11777	794	706	713	884	40438	214785	2353	10376	17667
Bihar	354	3536	20059	1590	1242	1298	1101	110195	615580	6455	34119	32720
Chhattisgarh	107	1070	5831	494	344	383	445	34768	173362	2570	11289	12059
Delhi	8	78	449	33	12	12	3	3402	18367	88	661	69
Goa	8	79	370	51	35	42	39	2886	11202	414	1121	1544
Gujarat	150	1497	8007	721	616	701	632	61384	307814	9762	20859	21330
Haryana	86	851	4881	484	372	437	507	26260	143202	5550	11118	13695
Himachal Pradesh	126	1239	6248	693	501	568	654	11323	52711	1905	4946	4600
Jammu & Kashmir	83	821	4772	403	323	334	350	10923	59602	1030	3801	4155
Jharkhand	141	1398	7554	561	366	381	248	36589	195002	1858	10841	6396
Karnataka	185	1847	9393	831	761	817	707	69596	327112	7813	22520	20918
Kerala	184	1839	8636	1154	963	1211	2148	52360	232988	29660	26687	59480
Madhya Pradesh	229	2281	12879	1093	864	943	842	84510	450315	8418	29367	27603
Maharashtra	265	2650	13577	1433	1158	1306	1442	117727	557585	18615	47679	51983
Manipur	107	1070	5776	374	369	382	158	2769	14425	278	673	411
Meghalaya	56	536	2861	191	118	123	154	3724	18864	133	898	970
Mizoram	41	394	2138	137	125	127	34	769	3859	67	196	80
Nagaland	24	240	1219	80	96	96	74	819	4113	53	148	257
Orissa	211	2094	10292	1008	802	868	761	64256	301133	7222	25527	23398
Punjab	82	816	4577	497	344	389	646	29462	154633	4948	13361	21156
Rajasthan	234	2311	13364	1135	857	948	919	74322	402102	7301	25196	22748
Sikkim	44	440	2167	204	156	168	124	884	3855	64	224	212

Continued

For peer review only

For peer review only - http://bmjopen.bmj.com/site/about/guidelines.xhtml

Appendix A

Table (1): Number of villages/blocks, households, persons, aged (60 years and above) persons surveyed, number of hospitalised and ailing persons surveyed and average household size, separately for each State/UT

Rural

state/ut	number of surveyed				during last 365 days		sample persons reporting ailment during last 15 days	estimated (00)				
	villages / blocks	house-holds	persons	aged persons	sample persons hospita-lised	sample cases of hospi-talisa-tion		house-holds	persons	cases of hospita-lisation during last 365 days	aged persons	persons reportting ailment during last 15 days
(1)	(2)	(3)	(4)	(5)	(6)	(7)	(8)	(9)	(10)	(11)	(12)	(13)
Tamil Nadu	254	2540	10348	1120	1090	1194	1100	102672	395508	15805	34107	37573
Tripura	82	820	3798	306	347	358	501	5867	26112	1055	1602	3407
Uttaranchal	36	346	1703	192	128	138	120	12664	63267	1111	4865	3293
Uttar Pradesh	671	6682	41137	3474	2589	2798	4365	218347	1283403	17077	84086	128631
West Bengal	317	3170	16111	1366	1298	1425	1836	122084	583207	14285	36602	66736
A & N Islands	15	140	702	49	59	63	36	493	2239	145	120	115
Chandigarh	8	80	367	28	30	33	40	230	898	15	16	47
Dadra & N. Haveli	8	80	428	30	36	42	13	455	2276	212	117	35
Daman & Diu	8	80	341	20	32	32	14	284	992	24	17	18
Lakshadweep	8	80	446	38	32	34	58	49	240	14	16	27
Pondicherry	8	80	322	38	34	37	58	842	3299	223	309	490
ONES	429	4232	21984	1550	1450	1495	1287	16440	79053	1883	4184	5817
GUTs	55	540	2606	203	223	241	219	2353	9945	633	594	733
all-India	4755	47302	250775	22265	18346	20066	22871	1432066	7150765	178921	502670	630970

For peer review only

For peer review only - http://bmjopen.bmj.com/site/about/guidelines.xhtml

Appendix A

Table (1): Number of villages/blocks, households, persons, aged (60 years and above) persons surveyed, number of hospitalised and ailing persons surveyed and average household size, separately for each State/UT

Urban

state/ut	number of surveyed				during last 365 days		sample persons reporting ailment during last 15 days	estimated (00)				
	villages / blocks	house-holds	persons	aged persons	sample persons hospita-lised	sample cases of hospi-talisa-tion		house-holds	persons	cases of hospita-lisation during last 365 days	aged persons	persons reportting ailment during last 15 days
(1)	(2)	(3)	(4)	(5)	(6)	(7)	(8)	(9)	(10)	(11)	(12)	(13)
Andhra Pradesh	183	1824	8167	798	782	881	1150	49582	197123	6207	11470	22567
Arunachal Pradesh	32	320	1571	72	95	98	73	235	1015	27	17	52
Assam	48	480	2314	194	167	174	185	5327	22588	382	1180	1868
Bihar	64	638	3792	314	209	220	243	13707	72955	788	4283	4631
Chhattisgarh	40	400	2069	151	154	173	170	6245	28167	832	1401	2035
Delhi	100	961	4757	418	214	218	111	23077	99257	1088	4342	1575
Goa	12	120	533	72	48	55	51	1343	5554	214	485	532
Gujarat	131	1309	6569	616	561	626	605	36737	158240	6208	9693	12335
Haryana	55	549	2891	275	214	240	277	10125	47999	1589	3040	4202
Himachal Pradesh	20	200	903	99	94	99	88	1528	5751	184	294	341
Jammu & Kashmir	38	380	2005	193	147	161	166	2691	13556	283	744	1062
Jharkhand	60	598	3400	305	206	216	152	7584	38998	841	2664	1968
Karnataka	152	1518	7593	698	609	638	594	30672	136290	3306	8098	7751
Kerala	100	990	4697	612	515	623	1061	19615	81492	8874	8672	19610
Madhya Pradesh	128	1280	7116	646	531	590	561	27843	146425	4509	8580	9589
Maharashtra	267	2664	13001	1268	1167	1339	1665	90560	377719	15209	26513	44847
Manipur	52	520	2833	231	188	192	79	1069	5586	97	369	151
Meghalaya	24	239	1196	80	94	105	60	671	2797	69	90	140
Mizoram	64	631	3236	246	257	266	90	576	2841	83	145	50
Nagaland	12	120	594	29	49	49	34	520	2400	35	76	125
Orissa	56	560	2711	261	205	229	177	9739	40397	1346	2729	2195
Punjab	68	676	3446	329	253	271	408	17902	76184	2375	4912	8130
Rajasthan	108	1072	5891	537	450	509	520	21730	104402	2897	6088	7589
Sikkim	8	80	380	29	32	34	13	168	580	9	18	7

Continued

For peer review only - http://bmjopen.bmj.com/site/about/guidelines.xhtml

Appendix A

Table (1): Number of villages/blocks, households, persons, aged (60 years and above) persons surveyed, number of hospitalised and ailing persons surveyed and average household size, separately for each State/UT

Urban

state/ut	number of surveyed				during last 365 days		sample persons reporting ailment during last 15 days	estimated (00)				
	villages / blocks	house-holds	persons	aged persons	sample persons hospita-lised	sample cases of hospi-talisa-tion		house-holds	persons	cases of hospita-lisation during last 365 days	aged persons	persons reportting ailment during last 15 days
(1)	(2)	(3)	(4)	(5)	(6)	(7)	(8)	(9)	(10)	(11)	(12)	(13)
Tamil Nadu	260	2599	10946	1237	1104	1228	1255	57196	214404	8355	16939	20654
Tripura	24	240	1017	99	106	107	93	1157	4220	194	267	305
Uttaranchal	20	200	943	98	79	85	104	4314	17011	343	1252	1103
Uttar Pradesh	264	2627	15097	1241	1001	1115	1822	64161	335456	7401	18694	36403
West Bengal	188	1879	8793	1028	781	872	1335	45779	184397	6974	17040	28996
A & N Islands	12	120	543	40	60	69	39	251	1032	54	33	61
Chandigarh	34	333	1438	137	128	138	145	1777	6378	165	344	452
Dadra & N. Haveli	8	80	355	27	32	34	7	59	269	8	7	7
Daman & Diu	8	79	387	38	28	37	15	91	383	16	26	13
Lakshadweep	8	80	516	45	40	48	74	47	284	26	19	40
Pondicherry	20	200	863	103	90	110	169	1561	6299	376	586	1175
ONES	216	2150	10827	786	821	851	442	4395	19439	514	983	830
GUTs	90	892	4102	390	378	436	449	3787	14646	645	1015	1748
all-India	2668	26566	132563	12566	10690	11849	13591	555641	2438451	81363	161110	242560

For peer review only

For peer review only - http://bmjopen.bmj.com/site/about/guidelines.xhtml

Appendix A

Table (1): Number of villages/blocks, households, persons, aged (60 years and above) persons surveyed, number of hospitalised and ailing persons surveyed and average household size, separately for each State/UT

Rural+Urban

state/ut	number of surveyed				during last 365 days		sample persons reporting ailment during last 15 days	estimated (00)				
	villages / blocks	house-holds	persons	aged persons	sample persons hospita-lised	sample cases of hospi-talisa-tion		house-holds	persons	cases of hospita-lisation during last 365 days	aged persons	persons reportting ailment during last 15 days
(1)	(2)	(3)	(4)	(5)	(6)	(7)	(8)	(9)	(10)	(11)	(12)	(13)
Andhra Pradesh	508	5059	22387	2183	2084	2305	2766	176688	716010	18373	50236	69233
Arunachal Pradesh	107	1052	5596	330	334	339	315	1842	8841	261	460	532
Assam	263	2630	14091	988	873	887	1069	45765	237373	2735	11556	19535
Bihar	418	4174	23851	1904	1451	1518	1344	123902	688535	7243	38403	37351
Chhattisgarh	147	1470	7900	645	498	556	615	41013	201529	3402	12690	14094
Delhi	108	1039	5206	451	226	230	114	26479	117625	1176	5002	1644
Goa	20	199	903	123	83	97	90	4229	16756	628	1606	2076
Gujarat	281	2806	14576	1337	1177	1327	1237	98121	466054	15970	30551	33665
Haryana	141	1400	7772	759	586	677	784	36386	191201	7139	14158	17897
Himachal Pradesh	146	1439	7151	792	595	667	742	12851	58463	2089	5240	4941
Jammu & Kashmir	121	1201	6777	596	470	495	516	13615	73158	1313	4545	5217
Jharkhand	201	1996	10954	866	572	597	400	44174	234001	2699	13505	8364
Karnataka	337	3365	16986	1529	1370	1455	1301	100268	463403	11120	30618	28669
Kerala	284	2829	13333	1766	1478	1834	3209	71975	314480	38534	35359	79090
Madhya Pradesh	357	3561	19995	1739	1395	1533	1403	112353	596739	12926	37947	37192
Maharashtra	532	5314	26578	2701	2325	2645	3107	208288	935303	33825	74193	96830
Manipur	159	1590	8609	605	557	574	237	3837	20011	375	1043	562
Meghalaya	80	775	4057	271	212	228	214	4395	21661	201	988	1111
Mizoram	105	1025	5374	383	382	393	124	1345	6701	150	341	129
Nagaland	36	360	1813	109	145	145	108	1339	6513	88	224	382
Orissa	267	2654	13003	1269	1007	1097	938	73995	341530	8568	28256	25592
Punjab	150	1492	8023	826	597	660	1054	47364	230817	7323	18274	29286
Rajasthan	342	3383	19255	1672	1307	1457	1439	96052	506504	10197	31284	30336
Sikkim	52	520	2547	233	188	202	137	1052	4435	74	242	220

Continued

For peer review only

For peer review only - http://bmjopen.bmj.com/site/about/guidelines.xhtml

Appendix A

Table (1): Number of villages/blocks, households, persons, aged (60 years and above) persons surveyed, number of hospitalised and ailing persons surveyed and average household size, separately for each State/UT

Rural+Urban

state/ut	number of surveyed				during last 365 days		sample persons reporting ailment during last 15 days	estimated (00)				
	villages / blocks	house-holds	persons	aged persons	sample persons hospita-lised	sample cases of hospi-talisa-tion		house-holds	persons	cases of hospita-lisation during last 365 days	aged persons	persons reportting ailment during last 15 days
(1)	(2)	(3)	(4)	(5)	(6)	(7)	(8)	(9)	(10)	(11)	(12)	(13)
Tamil Nadu	514	5139	21294	2357	2194	2422	2355	159868	609912	24161	51046	58227
Tripura	106	1060	4815	405	453	465	594	7024	30332	1249	1869	3712
Uttaranchal	56	546	2646	290	207	223	224	16978	80278	1454	6116	4397
Uttar Pradesh	935	9309	56234	4715	3590	3913	6187	282507	1618859	24478	102779	165033
West Bengal	505	5049	24904	2394	2079	2297	3171	167863	767604	21259	53642	95732
A & N Islands	27	260	1245	89	119	132	75	744	3270	199	152	177
Chandigarh	42	413	1805	165	158	171	185	2007	7276	179	360	499
Dadra & N. Haveli	16	160	783	57	68	76	20	514	2545	220	124	41
Daman & Diu	16	159	728	58	60	69	29	375	1376	40	43	32
Lakshadweep	16	160	962	83	72	82	132	96	524	40	35	67
Pondicherry	28	280	1185	141	124	147	227	2403	9599	599	895	1665
ONES	645	6382	32811	2336	2271	2346	1729	20834	98492	2397	5167	6647
GUTs	145	1432	6708	593	601	677	668	6140	24590	1277	1609	2481
all-India	7423	73868	383338	34831	29036	31915	36462	1987707	9589216	260284	663779	873530

For peer review only

For peer review only - http://bmjopen.bmj.com/site/about/guidelines.xhtml

Appendix A

Table (2): Per 1000 distribution of households by type of structure for (i) each monthly per capita expenditure (mpce) class, (ii) each social-group and (iii) each household type

All-India Rural

monthly per capita expenditure class (Rs.)/ social groupp./ hh. type	type of structure							no. of house-holds per 1000 house-holds	no. of households	
	house-hold without structure	household with structure				n.r.	total		estd. (00)	sample
		pucca	semi-pucca	servi-ceable kutcha	unservi-ceable kutcha					
(1)	(2)	(3)	(4)	(5)	(6)	(7)	(8)	(9)	(10)	(11)
0 – 225	0	259	441	257	42	0	1000	46	65977	1944
225 – 255	0	285	411	269	35	0	1000	38	53995	1537
255 – 300	0	319	421	230	31	0	1000	88	125961	3734
300 – 340	0	381	374	215	30	0	1000	78	112145	3410
340 – 380	0	400	385	194	21	0	1000	93	132620	3961
380 – 420	0	427	373	183	16	1	1000	99	141982	4447
420 – 470	0	477	335	174	14	0	1000	102	146498	4777
470 – 525	0	535	309	143	12	0	1000	110	157063	5354
525 – 615	0	575	286	126	13	0	1000	109	156235	5627
615 – 775	0	647	243	102	8	0	1000	113	162457	5943
775 – 950	0	740	190	58	12	0	1000	58	82383	3077
950 +	0	839	115	44	3	0	1000	66	94751	3491
all classes	0	480	332	169	19	0	1000	1000	1432066	47302
ST	0	195	570	213	22	0	1000	105	150047	6906
SC	0	410	335	225	30	0	1000	219	314076	9154
OBC	0	520	306	157	16	0	1000	413	591725	18502
others	0	582	280	124	14	0	1000	263	376218	12740
all groups	0	480	332	169	19	0	1000	1000	1432066	47302
self-employed in non-agriculture	0	551	294	139	16	0	1000	152	218051	7082
agricultural labour	0	295	408	262	36	0	1000	275	393716	10427
other labour	0	466	343	170	21	0	1000	111	158569	4849
self-employed in agriculture	0	530	321	138	12	0	1000	346	495746	19025
others	0	684	218	87	10	1	1000	116	165984	5919
all types	0	480	332	169	19	0	1000	1000	1432066	47302
estd. no. of hhs. (00)	212	670900	476432	254266	29964	293	1432066	X	X	X
sample hhs.	5	21735	15913	8633	1006	10	47302	X	X	X

For peer review only

For peer review only - http://bmjopen.bmj.com/site/about/guidelines.xhtml

Appendix A

Table (2): Per 1000 distribution of households by type of structure for (i) each monthly per capita expenditure (mpce) class, (ii) each social-group and (iii) each household type

All-India Urban

monthly per capita expenditure class (Rs.)/ social groupp./ hh. type	type of structure							no. of house-holds per 1000 house-holds	no. of households	
	household without structure	household with structure				n.r.	total		estd. (00)	sample
		pucca	semi-pucca	servi-ceable kutcha	unservi-ceable kutcha					
(1)	(2)	(3)	(4)	(5)	(6)	(7)	(8)	(9)	(10)	(11)
0 –300	1	494	326	129	50	0	1000	27	15106	684
300 – 350	0	561	274	130	35	0	1000	19	10600	476
350 – 425	0	654	244	81	20	0	1000	51	28410	1396
425 – 500	0	746	187	55	13	0	1000	99	55107	2735
500 – 575	0	738	192	61	8	0	1000	45	25162	1225
575 – 665	0	810	145	41	4	0	1000	81	45164	2259
665 – 775	0	857	111	26	6	0	1000	110	60948	3090
775 – 915	1	897	82	16	4	0	1000	101	56174	3036
915 – 1120	0	938	50	11	1	0	1000	149	82902	3829
1120 – 1500	1	961	31	7	0	0	1000	158	87926	4158
1500 – 1925	0	976	18	3	0	3	1000	48	26825	1311
1925 +	0	993	4	3	0	0	1000	110	61317	2367
all classes	0	842	115	35	8	0	1000	1000	555641	26566
ST	0	653	259	69	19	0	1000	26	14552	1803
SC	1	765	150	62	23	0	1000	156	86575	3666
OBC	0	813	136	43	7	0	1000	351	195136	8976
others	0	902	79	17	2	0	1000	467	259378	12121
all groups	0	842	115	35	8	0	1000	1000	555641	26566
self-employed	0	861	101	31	7	0	1000	364	202439	10632
regular wage salary earning	0	907	72	18	2	0	1000	401	222650	10198
casual labour	0	564	312	101	23	0	1000	128	70945	3053
others	0	895	63	28	14	0	1000	107	59606	2683
all types	0	842	115	35	8	0	1000	1000	555641	26566
estd. no. of hhs. (00)	270	471283	60496	19460	4053	79	555641	X	X	X
sample hhs.	9	21718	3559	1096	180	4	26566	X	X	X

For peer review only

For peer review only - http://bmjopen.bmj.com/site/about/guidelines.xhtml

Appendix A

Table (2): Per 1000 distribution of households by type of structure for (i) each monthly per capita expenditure (mpce) class, (ii) each social-group and (iii) each household type

All-India | Rural + Urban

monthly per capita expenditure class (Rs.)/ social groupp./ hh. type	type of structure							no. of house-holds per 1000 house-holds	no. of households	
	house-hold without structure	household with structure				n.r.	total		estd. (00)	sample
		pucca	semi-pucca	servi-ceable kutcha	unservi-ceable kutcha					
(1)	(2)	(3)	(4)	(5)	(6)	(7)	(8)	(9)	(10)	(11)
0 – 225	0	299	422	235	44	0	1000	41	81083	2628
225 – 255	0	331	388	246	35	0	1000	32	64595	2013
255 – 300	0	381	388	202	29	0	1000	78	154371	5130
300 – 340	0	497	315	164	25	0	1000	84	167251	6145
340 – 380	0	456	353	172	19	0	1000	79	157781	5186
380 – 420	0	515	321	150	14	0	1000	94	187146	6706
420 – 470	0	580	274	134	12	0	1000	104	207446	7867
470 – 525	1	627	251	111	10	0	1000	107	213237	8390
525 – 615	0	686	214	91	9	0	1000	120	239136	9456
615 – 775	0	746	176	72	6	0	1000	126	250383	10101
775 – 950	0	794	151	45	10	1	1000	55	109208	4388
950 +	0	894	75	29	2	0	1000	79	156068	5858
all classes	0	572	277	135	16	0	1000	1000	1987707	73868
ST	0	230	546	202	22	0	1000	83	164600	8709
SC	0	482	297	192	29	0	1000	202	400650	12820
OBC	0	586	268	131	14	0	1000	396	786861	27478
others	0	700	205	84	10	0	1000	320	635596	24861
all groups	0	572	277	135	16	0	1000	1000	1987707	73868
self-employed in non-agriculture	0	700	202	87	11	0	1000	212	420491	17714
agricultural labour	0	501	295	180	24	0	1000	310	616366	20625
other labour	0	495	333	149	22	0	1000	115	229514	7902
self-employed in agriculture	0	530	321	138	12	0	1000	249	495746	19025
others	0	723	190	76	11	1	1000	113	225590	8602
all types	0	572	277	135	16	0	1000	1000	1987707	73868
estd. no. of hhs. (00)	482	1142183	536927	273726	34016	372	1987707	X	X	X
sample hhs.	14	43453	19472	9729	1186	14	73868	X	X	X

For peer review only - http://bmjopen.bmj.com/site/about/guidelines.xhtml

Appendix A

Table (3): Per 1000 distribution of households by major source of drinking water for (i) each mpce class, (ii) each socia[l] group, and (iii) each household type

All-India | Rural

monthly per capita expendi-ture class (Rs.)/social gp./ hh. type	major source of drinking water										no. of households	
	bottled water	tap	tube-well/ hand pump	tan-kers	pucca well	tank/ pond reser-ved for drin-king	river/ canal	others	n.r.	total	estd. (00)	sampl[e]
(1)	(2)	(3)	(4)	(5)	(6)	(7)	(8)	(9)	(10)	(11)	(12)	(13
0 – 225	4	146	670	5	147	4	7	16	0	1000	65977	194
225 – 255	6	169	673	1	120	7	13	12	0	1000	53995	153
255 – 300	6	184	662	3	120	10	8	8	0	1000	125961	373
300 – 340	5	201	626	4	140	9	7	8	0	1000	112145	341
340 – 380	6	218	627	8	114	10	10	7	0	1000	132620	396
380 – 420	8	234	575	8	137	17	9	12	0	1000	141982	444
420 – 470	12	250	547	5	158	13	7	9	0	1000	146498	477
470 – 525	11	268	538	7	140	15	10	11	0	1000	157063	535
525 – 615	13	303	490	8	141	23	10	12	0	1000	156235	562
615 – 775	15	311	476	17	147	17	8	11	0	1000	162457	594
775 – 950	19	336	466	10	140	14	6	10	0	1000	82383	307
950 +	17	391	383	12	167	16	5	9	0	1000	94751	349
all classes	10	248	564	7	139	13	8	10	0	1000	1432066	4730
ST	4	153	553	3	200	22	30	34	0	1000	150047	690
SC	9	263	594	4	107	11	5	6	0	1000	314076	915
OBC	9	258	546	9	153	13	6	5	0	1000	591725	1850
others	14	256	573	9	117	12	6	12	0	1000	376218	1274
all groups	10	248	564	7	139	13	8	10	0	1000	1432066	4730
self-employed in non-agriculture	13	257	564	6	136	9	7	9	0	1000	218051	708
agricultural labour	10	277	567	6	117	9	8	7	0	1000	393716	1042
other labour	7	303	460	13	180	19	6	13	0	1000	158569	484
self-employed in agriculture	8	192	607	7	143	18	11	13	0	1000	495746	1902
others	15	323	495	9	136	7	6	8	0	1000	165984	591
all types	10	248	564	7	139	13	8	10	0	1000	1432066	4730
estd. no. of hhs. (00)	4974	389225	770736	10219	200542	19018	11796	15461	94	1432066	X	[illegible]
sample hhs.	572	12697	23792	303	6577	1083	773	1500	5	47302	X	[illegible]

For peer review only

For peer review only - http://bmjopen.bmj.com/site/about/guidelines.xhtml

Appendix A

Table (3): Per 1000 distribution of households by major source of drinking water for (i) each mpce class, (ii) each socia[illegible] group, and (iii) each household type

All-India Urba[illegible]

monthly per capita expendi-ture class (Rs.)/social gp./ hh. type	major source of drinking water										no. of household[illegible]	
	bottled water	tap	tube-well/ hand pump	tan-kers	pucca well	tank/ pond reser-ved for drin-king	river/ canal	others	n.r.	total	estd. (00)	sampl[illegible]
(1)	(2)	(3)	(4)	(5)	(6)	(7)	(8)	(9)	(10)	(11)	(12)	(13[illegible]
0 –300	6	466	461	9	56	0	1	1	0	1000	15106	68
300 – 350	19	504	399	12	61	1	0	5	0	1000	10600	47
350 – 425	14	566	355	21	40	0	0	4	0	1000	28410	139
425 – 500	16	601	303	24	53	1	0	2	0	1000	55107	273
500 – 575	8	569	336	18	63	2	2	2	0	1000	25162	122
575 – 665	12	649	256	22	55	2	1	3	0	1000	45164	225
665 – 775	32	677	210	18	59	2	1	2	0	1000	60948	309
775 – 915	22	733	174	24	38	5	0	3	0	1000	56174	303
915 – 1120	29	724	179	27	36	2	0	3	0	1000	82902	382
1120 – 1500	36	772	122	30	34	2	0	4	0	1000	87926	415
1500 – 1925	34	769	117	37	38	1	0	4	0	1000	26825	131
1925 +	94	776	95	17	16	0	0	2	0	1000	61317	236
all classes	28	676	224	23	44	2	0	3	0	1000	555641	2656
ST	29	589	190	18	128	21	3	23	0	1000	14552	180
SC	21	631	287	27	31	1	0	2	0	1000	86575	366
OBC	23	634	242	33	63	2	1	3	0	1000	195136	897
others	35	728	190	14	30	1	0	2	0	1000	259378	1212
all groups	28	676	224	23	44	2	0	3	0	1000	555641	2656
self-employed in non-agriculture	26	654	254	20	42	2	0	2	0	1000	202439	1063
agricultural labour	33	743	163	28	28	2	0	3	0	1000	222650	1019
other labour	21	588	279	20	86	1	0	3	0	1000	70945	305
others	31	593	284	17	70	1	0	4	0	1000	59606	268
all types	28	676	224	23	44	2	0	3	0	1000	555641	2656
estd. no. of hhs. (00)	7305	383355	115097	13578	23566	1049	176	1515	0	555641	X	[illegible]
sample hhs.	761	18203	5134	587	1294	242	72	272	1	26566	X	[illegible]

For peer review only - http://bmjopen.bmj.com/site/about/guidelines.xhtml

Appendix A

Table (3): Per 1000 distribution of households by major source of drinking water for (i) each mpce class, (ii) each socia group, and (iii) each household type

All-India — Rural + Urbar

monthly per capita expendi-ture class (Rs.)/social gp./ hh. type	major source of drinking water										no. of households	
	bottled water	tap	tube-well/ hand pump	tan-kers	pucca well	tank/ pond reser-ved for drin-king	river/ canal	others	n.r.	total	estd. (00)	sampl
(1)	(2)	(3)	(4)	(5)	(6)	(7)	(8)	(9)	(10)	(11)	(12)	(13
0 – 235	5	200	634	6	132	4	6	13	0	1000	81083	262
235 – 265	8	224	628	3	110	6	11	11	0	1000	64595	201
265 – 320	7	254	605	6	106	8	7	7	0	1000	154371	513
320 – 365	8	329	523	11	112	6	5	6	0	1000	167251	614
365 – 410	7	276	579	9	106	8	8	6	0	1000	157781	518
410 – 460	9	329	502	12	118	13	7	10	0	1000	187146	670
460 – 520	18	366	455	8	131	10	5	7	0	1000	207446	786
520 – 605	14	386	446	11	114	12	7	9	0	1000	213237	839
605 – 730	18	432	395	14	109	17	7	9	0	1000	239136	945
730 – 980	21	456	364	21	112	12	5	9	0	1000	250383	1010
980 – 1285	23	434	386	16	116	11	5	8	0	1000	109208	438
1285 +	45	530	279	14	112	10	3	7	0	1000	156068	585
all classes	15	357	478	11	115	10	6	8	0	1000	1987707	7386
ST	6	187	525	4	194	22	28	33	0	1000	164600	870
SC	11	338	532	8	92	9	4	5	0	1000	400650	1282
OBC	12	343	477	14	133	11	5	5	0	1000	786861	2747
others	22	431	431	11	85	8	4	8	0	1000	635596	2486
all groups	15	357	478	11	115	10	6	8	0	1000	1987707	7386
self-employed in non-agriculture	19	446	416	13	91	6	4	5	0	1000	420491	1771
agricultural labour	18	434	431	13	87	7	5	6	0	1000	616366	2062
other labour	11	387	407	15	152	14	4	10	0	1000	229514	790
self-employed in agriculture	8	192	607	7	143	18	11	13	0	1000	495746	1902
others	18	373	456	10	124	6	5	7	0	1000	225590	860
all types	15	357	478	11	115	10	6	8	0	1000	1987707	7386
estd. no. of hhs. (00)	2278	772580	885833	23797	224108	20067	11972	16977	95	1987707	X	[illegible]
sample hhs.	1333	30900	28926	890	7871	1325	845	1772	6	73868	X	[illegible]

For peer review only - http://bmjopen.bmj.com/site/about/guidelines.xhtml

Appendix A

Table (4): Proportion of households treating water before drinking and per 1000 distribution of such households by type of water treatment for each major source of drinking water

All-India Rural

major source of drinking water	no. per 1000 hhs. treating water before drinking	type of treatment done to water before drinking							households treating water	
		ultra-violet /resin	filter	boiling	cloth screen	any disin-fectant	others	total	estd. no. (00)	sample
(1)	(2)	(3)	(4)	(5)	(6)	(7)	(8)	(9)	(10)	(11)
tap	291	8	210	87	669	8	18	1000	113397	3690
tube well/ hand pump	98	11	123	128	718	5	15	1000	75207	2379
tankers	459	1	148	98	741	11	0	1000	4690	143
pucca well	366	5	417	58	474	22	23	1000	73306	2360
tank/pond reserved for drinking	551	3	239	35	709	11	3	1000	10480	644
river/canal	327	19	339	81	512	20	29	1000	3861	281
others	276	14	621	124	230	0	11	1000	4292	581
total	199	8	248	89	626	11	18	1000	285234	10078
estd. no. of hhs. treating water (00)	285234	2234	70865	25433	178562	3120	5021	285234	X	X
sample hhs. treating water	10078	61	3793	1216	4716	125	167	10078	X	X

Table (4): Proportion of households treating water before drinking and per 1000 distribution of such households by type of water treatment for each major source of drinking water

All-India

Urban

major source of drinking water	no. per 1000 hhs. treating water before drinking	type of treatment done to water before drinking							households treating water	
		ultra-violet /resin	filter	boiling	cloth screen	any disin-fectant	others	total	estd. no. (00)	sample
(1)	(2)	(3)	(4)	(5)	(6)	(7)	(8)	(9)	(10)	(11)
tap	436	56	222	285	415	11	11	1000	167042	8226
tube well/ hand pump	164	57	145	372	384	15	27	1000	18912	1055
tankers	546	30	576	141	253	0	0	1000	7409	322
pucca well	570	31	589	108	258	0	14	1000	13439	708
tank/pond reserved for drinking	731	18	419	78	419	0	66	1000	767	127
river/canal	584	0	123	287	590	0	0	1000	103	22
others	351	0	766	213	0	21	0	1000	531	126
total	375	53	254	275	395	10	13	1000	208203	10586
estd. no. of hhs. treating water (00)	208203	11087	52828	57321	82266	2067	2636	208203	X	X
sample hhs. treating water	10586	448	3385	3155	3302	142	154	10586	X	X

For peer review only

For peer review only - http://bmjopen.bmj.com/site/about/guidelines.xhtml

Appendix A

Table (4): Proportion of households treating water before drinking and per 1000 distribution of such households by type of water treatment for each major source of drinking water

All-India Rural + Urban

major source of drinking water	no. per 1000 hhs. treating water before drinking	type of treatment done to water before drinking							households treating water	
		ultra-violet /resin	filter	boiling	cloth screen	any disin-fectant	others	total	estd. no. (00)	sample
(1)	(2)	(3)	(4)	(5)	(6)	(7)	(8)	(9)	(10)	(11)
tap	363	36	218	205	518	10	14	1000	280439	11916
tube well/ hand pump	106	20	127	177	651	7	18	1000	94119	3434
tankers	508	19	410	125	442	4	0	1000	12099	465
pucca well	387	9	444	66	441	19	21	1000	86746	3068
tank/pond reserved for drinking	560	4	252	38	689	10	7	1000	11247	771
river/canal	331	18	334	86	514	20	29	1000	3963	303
others	283	12	637	134	205	2	10	1000	4823	707
total	248	27	251	168	529	11	16	1000	493437	20664
estd. no. of hhs. treating water (00)	493437	13320	123692	82754	260828	5187	7656	493437	X	X
sample hhs. treating water	20664	509	7178	4371	8018	267	321	20664	X	X

For peer review only - http://bmjopen.bmj.com/site/about/guidelines.xhtml

Appendix A

Table (5): Number per 1000 households treating water before drinking and per 1000 distribution of such households by type of water treatment for each mpce class

All-India Rural

monthly per capita expenditure class (Rs.)	no. per 1000 hhs. treating water before drinking	type of treatment done to water before drinking							households treating water	
		ultra-violet /resin	filter	boiling	cloth screen	any disin-fectant	others	total	estd. no. (00)	sample
(1)	(2)	(3)	(4)	(5)	(6)	(7)	(8)	(9)	(10)	(11)
0 – 225	107	35	84	67	776	10	28	1000	7071	221
225 – 255	122	6	70	67	842	5	9	1000	6568	167
255 – 300	132	2	120	65	774	12	26	1000	16645	486
300 – 340	140	0	135	84	742	11	28	1000	15734	482
340 – 380	153	0	132	56	779	23	9	1000	20338	638
380 – 420	160	8	199	73	679	13	27	1000	22661	753
420 – 470	186	3	193	70	705	5	24	1000	27181	903
470 – 525	202	2	226	86	657	10	19	1000	31711	1193
525 – 615	222	3	252	75	651	8	12	1000	34728	1333
615 – 775	264	14	323	89	542	14	18	1000	42893	1605
775 – 950	285	10	299	125	546	5	16	1000	23487	989
950 +	382	17	454	148	361	12	8	1000	36217	1308
all classes	199	8	248	89	626	11	18	1000	285234	10078
estd. no. of hhs. treating water (00)	285234	2234	70865	25433	178562	3120	5021	285234	X	X
sample hhs. treating water	10078	61	3793	1216	4716	125	167	10078	X	X

Table (5): Number per 1000 households treating water before drinking and per 1000 distribution of such households by type of water treatment for each mpce class

All-India Urban

monthly per capita expenditure class (Rs.)	no. per 1000 hhs. treating water before drinking	type of treatment done to water before drinking							households treating water	
		ultra-violet /resin	filter	boiling	cloth screen	any disin-fectant	others	total	estd. no. (00)	sample
(1)	(2)	(3)	(4)	(5)	(6)	(7)	(8)	(9)	(10)	(11)
0 –300	123	7	357	50	576	9	2	1000	1859	112
300 – 350	162	0	187	152	657	4	0	1000	1722	74
350 – 425	200	5	169	73	713	17	23	1000	5676	291
425 – 500	229	2	248	142	574	19	16	1000	12599	673
500 – 575	242	0	295	147	531	16	11	1000	6086	369
575 – 665	292	11	259	183	524	11	12	1000	13191	676
665 – 775	323	8	276	141	545	8	21	1000	19664	1109
775 – 915	355	4	237	205	518	20	16	1000	19963	1171
915 – 1120	397	14	247	297	420	8	14	1000	32943	1684
1120 – 1500	473	41	264	308	373	6	9	1000	41567	2144
1500 – 1925	572	113	297	358	216	9	7	1000	15344	786
1925 +	613	179	232	439	132	7	11	1000	37588	1497
all classes	375	53	254	275	395	10	13	1000	208203	10586
estd. no. of hhs. treating water (00)	208203	11087	52828	57321	82266	2067	2636	208203	X	X
sample hhs. treating water	10586	448	3385	3155	3302	142	154	10586	X	X

For peer review only - http://bmjopen.bmj.com/site/about/guidelines.xhtml

Appendix A

Table (5): Number per 1000 households treating water before drinking and per 1000 distribution of such households by type of water treatment for each mpce class

All-India Rural + Urban

monthly per capita expenditure class (Rs.)	no. per 1000 hhs. treating water before drinking	type of treatment done to water before drinking							households treating water	
		ultra-violet /resin	filter	boiling	cloth screen	any disin-fectant	others	total	estd. no. (00)	sample
(1)	(2)	(3)	(4)	(5)	(6)	(7)	(8)	(9)	(10)	(11)
0 – 235	110	29	141	63	734	10	22	1000	8930	333
235 – 265	128	5	95	85	804	5	8	1000	8290	241
265 – 320	145	3	132	67	758	14	25	1000	22321	777
320 – 365	169	1	185	110	667	14	23	1000	28333	1155
365 – 410	167	0	170	77	722	22	10	1000	26425	1007
410 – 460	192	9	221	114	622	12	22	1000	35852	1429
460 – 520	226	5	228	100	638	6	22	1000	46844	2012
520 – 605	242	3	230	132	603	14	18	1000	51674	2364
605 – 730	283	9	250	183	539	8	13	1000	67671	3017
730 – 980	337	27	294	197	459	10	14	1000	84460	3749
980 – 1285	356	51	298	217	416	6	12	1000	38831	1775
1285 +	473	100	341	296	244	10	9	1000	73805	2805
all classes	248	27	251	168	529	11	16	1000	493437	20664
estd. no. of hhs. treating water (00)	493437	13320	123692	82754	260828	5187	7656	493437	X	X
sample hhs. treating water	20664	509	7178	4371	8018	267	321	20664	X	X

For peer review only - http://bmjopen.bmj.com/site/about/guidelines.xhtml

Table (6): Per 1000 distribution of households by primary source of energy for cooking for each MPCE class

All-India Rural

monthly per capita expenditure class (Rs.)	no cook-ing arra-nge-ment	primary source of energy for cooking											households	
		coke/ coal	fire-wood and chips	LPG	gob ar gas	dung cake	char-coal	kero-sene	elec-tri-city	others	n.r.	total	estd. no. (00)	sam-ple
(1)	(2)	(3)	(4)	(5)	(6)	(7)	(8)	(9)	(10)	(11)	(12)	(13)	(14)	(15)
0 – 225	8	4	855	9	0	68	0	0	0	56	0	1000	65977	1944
225 – 255	0	11	833	12	1	86	1	0	0	56	0	1000	53995	1537
255 – 300	4	6	832	10	0	101	2	2	0	42	1	1000	125961	3734
300 – 340	10	5	808	11	0	105	1	3	0	56	0	1000	112145	3410
340 – 380	6	10	818	19	3	98	0	3	0	41	0	1000	132620	3961
380 – 420	1	8	801	30	3	110	0	3	0	44	0	1000	141982	4447
420 – 470	5	11	834	30	3	81	0	4	0	32	0	1000	146498	4777
470 – 525	3	9	799	57	3	95	0	8	0	26	1	1000	157063	5354
525 – 615	4	8	796	74	3	79	0	9	1	25	0	1000	156235	5627
615 – 775	7	6	734	131	4	87	0	12	0	18	0	1000	162457	5943
775 – 950	8	7	642	227	5	71	0	20	1	19	0	1000	82383	3077
950 +	25	8	423	439	4	60	0	27	3	10	0	1000	94751	3491
all classes	6	8	770	82	3	89	0	8	0	34	0	1000	1432066	47302
estd. hhs. (00)	277	11257	1103263	117093	3847	126857	484	10763	713	48130	383	1432066	X	X
sample hhs.	108	374	36672	4271	122	4050	29	348	29	1282	17	47302	X	X

For peer review only - http://bmjopen.bmj.com/site/about/guidelines.xhtml

Appendix A

Table (6): Per 1000 distribution of households by primary source of energy for cooking for each MPCE class

All-India Urban

monthly per capita expenditure class (Rs.)	no cook-ing arra-nge-ment	primary source of energy for cooking											households	
		coke/ coal	fire-wood and chips	LPG	gob ar gas	dung cake	char-coal	kero-sene	elec-tri-city	others	n.r.	total	estd. no. (00)	sam-ple
(1)	(2)	(3)	(4)	(5)	(6)	(7)	(8)	(9)	(10)	(11)	(12)	(13)	(14)	(15)
0 –300	48	92	634	128	0	32	5	20	0	40	0	1000	15106	684
300 – 350	35	65	606	159	0	51	11	44	1	29	0	1000	10600	476
350 – 425	32	35	585	202	1	37	1	92	0	16	0	1000	28410	1396
425 – 500	9	52	478	287	0	34	4	115	3	18	0	1000	55107	2735
500 – 575	26	46	429	333	5	29	1	96	4	30	0	1000	25162	1225
575 – 665	42	31	331	424	0	16	3	144	1	7	0	1000	45164	2259
665 – 775	20	40	253	494	1	11	3	172	3	5	0	1000	60948	3090
775 – 915	31	22	170	623	0	6	0	135	4	8	0	1000	56174	3036
915 – 1120	40	25	97	657	1	8	0	163	5	5	0	1000	82902	3829
1120 – 1500	66	7	52	757	0	3	1	101	3	10	0	1000	87926	4158
1500 – 1925	39	9	22	861	0	1	0	49	7	12	0	1000	26825	1311
1925 +	107	0	15	853	0	1	0	13	0	10	0	1000	61317	2367
all classes	44	27	223	566	1	13	1	110	3	12	0	1000	555641	26566
estd. hhs. (00)	24724	15051	123747	314275	310	7431	828	61240	1585	6400	50	555641	X	X
sample hhs.	339	816	6196	16357	21	339	69	2106	89	225	9	26566	X	X

For peer review only

For peer review only - http://bmjopen.bmj.com/site/about/guidelines.xhtml

Appendix A

Table (6): Per 1000 distribution of households by primary source of energy for cooking for each MPCE class

All-India Rural + Urban

monthly per capita expenditure class (Rs.)	no cook-ing arra-nge-ment	primary source of energy for cooking											households	
		coke/ coal	fire-wood and chips	LPG	gob ar gas	dung cake	char-coal	kero-sene	elec-tri-city	others	n.r.	total	estd. no. (00)	sam-ple
(1)	(2)	(3)	(4)	(5)	(6)	(7)	(8)	(9)	(10)	(11)	(12)	(13)	(14)	(15)
0 – 235	15	21	814	31	0	61	1	4	0	53	0	1000	81083	2628
235 – 265	6	20	796	36	1	80	2	7	0	52	0	1000	64595	2013
265 – 320	9	11	787	45	0	89	1	18	0	37	1	1000	154371	5130
320 – 365	10	20	699	102	0	82	2	40	1	43	0	1000	167251	6145
365 – 410	9	16	756	69	3	87	0	18	1	40	0	1000	157781	5186
410 – 460	11	14	687	125	3	87	1	37	0	35	0	1000	187146	6706
460 – 520	10	20	663	166	2	60	1	53	1	24	0	1000	207446	7867
520 – 605	11	12	633	206	2	72	0	41	1	21	1	1000	213237	8390
605 – 730	17	14	554	276	2	54	0	62	2	18	0	1000	239136	9456
730 – 980	28	6	494	351	3	57	0	43	1	15	0	1000	250383	10101
980 – 1285	16	7	490	383	4	54	0	27	2	17	0	1000	109208	4388
1285 +	57	5	263	602	2	37	0	22	2	10	0	1000	156068	5858
all classes	17	13	617	217	2	68	1	36	1	27	0	1000	1987707	73868
estd. hhs. (00)	34001	26308	1227010	431368	4157	134288	1312	72003	2298	54529	432	1987707	X	X
sample hhs.	447	1190	42868	20628	143	4389	98	2454	118	1507	26	73868	X	X

For peer review only

For peer review only - http://bmjopen.bmj.com/site/about/guidelines.xhtml

Appendix A

Table (7): Number per 1000 of households having pet animals and per 1000 distribution of such households by mpce class for each State/UT

Rural

state/ut	number per 1000 of hhs. having a pet animal	households with pet animals in mpce class													households having a pet animal	
		0-225	225-255	255-300	300-340	340-380	380-420	420-470	470-525	525-615	615-775	775-950	950+	total	estd. no. (00)	sample
(1)	(2)	(3)	(4)	(5)	(6)	(7)	(8)	(9)	(10)	(11)	(12)	(13)	(14)	(15)	(16)	(17)
Andhra Pradesh	36	48	39	115	62	55	124	146	181	88	89	31	21	1000	4526	136
Arunachal Pradesh	561	100	26	26	28	42	83	83	100	118	122	136	136	1000	903	456
Assam	524	12	10	50	48	62	92	121	142	201	179	63	22	1000	21203	1145
Bihar	175	61	44	103	69	134	170	105	116	110	55	26	7	1000	19246	651
Chhattisgarh	53	120	111	184	61	134	102	83	81	6	5	113	0	1000	1831	60
Delhi	4	0	0	0	0	0	0	0	0	0	1000	0	0	1000	15	1
Goa	186	0	0	0	0	0	0	34	112	53	205	57	539	1000	536	22
Gujarat	58	41	5	42	14	113	47	34	82	157	171	94	201	1000	3558	103
Haryana	108	0	0	7	2	0	4	12	93	174	179	263	265	1000	2830	94
Himachal Pradesh	139	10	14	17	69	19	52	40	101	168	158	93	260	1000	1571	192
Jammu & Kashmir	159	10	0	24	20	52	49	104	141	144	193	116	146	1000	1742	112
Jharkhand	373	41	68	117	123	108	133	128	66	115	74	17	11	1000	13635	559
Karnataka	160	20	27	68	68	106	93	147	94	93	158	38	88	1000	11106	339
Kerala	198	2	0	22	14	12	43	65	68	116	217	137	302	1000	10350	356
Madhya Pradesh	172	154	42	90	114	137	87	101	89	86	61	39	0	1000	14535	404
Maharashtra	131	24	31	69	48	71	122	99	144	103	124	85	79	1000	15398	395
Manipur	501	4	0	0	6	26	32	153	166	270	202	87	53	1000	1387	545
Meghalaya	553	3	0	8	35	55	71	125	192	207	187	71	47	1000	2058	319
Mizoram	419	0	0	1	9	1	30	41	52	220	236	201	209	1000	322	162
Nagaland	650	0	0	0	0	0	6	0	47	42	134	420	350	1000	533	163

Continued

For peer review only - http://bmjopen.bmj.com/site/about/guidelines.xhtml

Appendix A

Table (7): Number per 1000 of households having pet animals and per 1000 distribution of such households by mpce class for each State/UT

Rural

state/ut	number per 1000 of hhs. having a pet animal	households with pet animals in mpce class													households having a pet animal	
		0-225	225-255	255-300	300-340	340-380	380-420	420-470	470-525	525-615	615-775	775-950	950+	total	estd. no. (00)	sample
(1)	(2)	(3)	(4)	(5)	(6)	(7)	(8)	(9)	(10)	(11)	(12)	(13)	(14)	(15)	(16)	(17)
Orissa	66	224	158	112	121	88	53	63	73	25	38	33	11	1000	4254	140
Punjab	129	0	0	0	21	2	77	15	103	156	142	163	322	1000	3790	107
Rajasthan	81	2	25	51	39	80	88	87	162	128	159	54	126	1000	6031	204
Sikkim	249	0	3	45	72	64	33	147	182	186	123	45	100	1000	220	112
Tamil Nadu	111	56	18	16	28	94	106	110	68	110	245	92	57	1000	11443	235
Tripura	352	22	60	105	71	116	133	140	114	99	86	31	23	1000	2066	297
Uttaranchal	215	0	66	90	43	117	158	193	83	101	60	47	42	1000	2728	102
Uttar Pradesh	89	24	55	111	76	130	106	132	112	82	86	51	35	1000	19426	633
West Bengal	110	30	33	78	77	86	152	161	101	108	94	42	37	1000	13390	354
A & N Islands	403	0	0	0	0	72	44	7	73	89	120	306	289	1000	198	84
Chandigarh	58	0	0	0	0	0	0	0	0	0	0	0	1000	1000	13	6
Dadra & N. Haveli	270	77	0	0	0	0	0	96	105	164	328	50	179	1000	123	26
Daman & Diu	43	0	0	0	0	0	0	0	106	202	0	485	208	1000	12	5
Lakshadweep	69	0	0	0	0	0	0	0	0	0	164	607	229	1000	3	7
Pondicherry	141	0	0	0	0	158	0	138	189	19	0	0	497	1000	119	7
ONES	456	20	20	36	36	59	75	117	138	166	150	100	82	1000	7489	2054
GUTs	199	20	0	0	0	70	18	63	109	91	138	159	331	1000	469	135
All-India	133	42	35	72	63	90	105	111	108	117	124	64	70	1000	191102	8533

For peer review only

For peer review only - http://bmjopen.bmj.com/site/about/guidelines.xhtml

Appendix A

For peer review only

Table (7): Number per 1000 of households having pet animals and per 1000 distribution of such households by mpce class for each State/UT

Urban

state/ut	number per 1000 of hhs. having a pet animal	households with pet animals in mpce class													households having a pet animal	
		0-300	300-350	350-425	425-500	500-575	575-665	665-775	775-915	915-1120	1120-1500	1500-1925	1925+	total	estd. no. (00)	sample
(1)	(2)	(3)	(4)	(5)	(6)	(7)	(8)	(9)	(10)	(11)	(12)	(13)	(14)	(15)	(16)	(17)
Andhra Pradesh	47	28	0	16	58	1	50	26	77	126	174	49	396	1000	2314	74
Arunachal Pradesh	121	3	0	0	75	0	43	168	503	164	14	31	0	1000	28	39
Assam	140	0	0	15	19	77	145	208	166	234	72	23	42	1000	745	90
Bihar	71	105	31	161	107	36	56	62	125	220	6	0	92	1000	980	50
Chhattisgarh	42	108	0	33	114	0	20	191	172	262	100	0	0	1000	265	14
Delhi	18	0	0	0	22	0	14	297	27	46	73	0	520	1000	407	16
Goa	238	0	0	4	77	0	52	139	157	78	249	136	108	1000	320	33
Gujarat	15	0	0	0	126	16	8	46	72	213	425	14	79	1000	551	27
Haryana	45	72	0	90	0	6	35	119	147	347	145	31	5	1000	456	22
Himachal Pradesh	76	0	0	0	277	0	0	28	187	74	313	0	119	1000	116	20
Jammu & Kashmir	17	0	0	0	0	0	0	164	507	135	89	0	106	1000	45	11
Jharkhand	45	0	16	74	255	91	16	36	30	210	146	18	110	1000	340	30
Karnataka	67	17	7	49	139	105	114	56	161	50	159	78	64	1000	2054	120
Kerala	158	2	11	41	66	50	70	111	57	107	172	140	172	1000	3105	182
Madhya Pradesh	59	5	4	237	164	81	152	96	91	39	77	1	52	1000	1635	94
Maharashtra	60	11	130	66	67	23	42	78	112	184	111	38	140	1000	5447	144
Manipur	188	0	0	9	139	71	175	315	118	123	51	0	0	1000	201	108
Meghalaya	159	0	7	3	0	0	92	12	70	180	202	366	67	1000	107	46
Mizoram	328	6	0	0	31	0	45	79	205	233	250	90	62	1000	189	208
Nagaland	349	0	0	0	0	0	0	45	80	241	421	97	115	1000	181	38

Continued

Appendix A

Table (7): Number per 1000 of households having pet animals and per 1000 distribution of such households by mpce class for each State/UT

Urban

state/ut	number per 1000 of hhs. having a pet animal	households with pet animals in mpce class												households having a pet animal	
		0-300	300-350	350-425	425-500	500-575	575-665	665-775	775-915	915-1120	1120-1500	1500-1925	1925+	total	estd. no. (00)	sample
(1)	(2)	(3)	(4)	(5)	(6)	(7)	(8)	(9)	(10)	(11)	(12)	(13)	(14)	(15)	(16)	(17)
Orissa	34	109	71	0	94	146	64	169	20	89	40	47	151	1000	334	30
Punjab	33	0	16	11	210	0	3	241	251	29	135	15	89	1000	589	35
Rajasthan	28	93	0	58	21	39	55	58	58	354	204	32	28	1000	616	39
Sikkim	140	0	0	0	0	0	0	158	59	296	65	422	0	1000	23	14
Tamil Nadu	48	1	0	62	109	32	81	85	68	135	164	43	220	1000	2746	153
Tripura	56	0	0	0	312	184	247	133	0	124	0	0	0	1000	65	15
Uttaranchal	84	0	0	12	143	0	209	96	43	112	170	203	10	1000	362	25
Uttar Pradesh	45	39	24	127	79	93	49	89	93	158	87	88	76	1000	2857	150
West Bengal	46	37	48	94	76	9	47	75	43	136	142	125	168	1000	2084	104
A & N Islands	96	0	0	0	0	28	28	28	582	265	25	31	13	1000	24	16
Chandigarh	11	0	0	0	39	0	0	0	0	169	143	0	650	1000	20	10
Dadra & N. Haveli	11	0	0	0	0	0	462	192	0	0	0	346	0	1000	1	3
Daman & Diu	0	0	0	0	0	0	0	0	0	0	0	0	0	0	0	0
Lakshadweep	212	0	0	0	29	19	0	50	514	326	35	26	0	1000	10	23
Pondicherry	33	192	0	0	77	138	69	103	73	0	0	217	131	1000	52	13
ONES	181	1	1	3	71	33	89	132	126	190	198	106	50	1000	795	468
GUTs	28	93	0	0	47	75	43	62	215	122	36	117	189	1000	107	65
All-India	53	22	34	70	88	42	66	91	97	145	137	63	145	1000	29268	1996

For peer review only - http://bmjopen.bmj.com/site/about/guidelines.xhtml

Appendix A

Table (7): Number per 1000 of households having pet animals and per 1000 distribution of such households by mpce class for each State/UT

Rural+Urban

state/ut	number per 1000 of hhs. having a pet animal	households with pet animals in mpce class													households having a pet animal	
		0-235	235-265	265-320	320-365	365-410	410-460	460-520	520-605	605-730	730-980	980-1285	1285+	total	estd. no. (00)	sample
(1)	(2)	(3)	(4)	(5)	(6)	(7)	(8)	(9)	(10)	(11)	(12)	(13)	(14)	(15)	(16)	(17)
Andhra Pradesh	39	41	26	82	61	37	99	106	146	101	117	37	148	1000	6840	210
Arunachal Pradesh	505	97	25	25	29	41	82	86	112	119	119	133	132	1000	931	495
Assam	480	11	9	49	47	62	94	124	142	202	175	61	22	1000	21948	1235
Bihar	163	63	43	106	71	129	164	103	117	115	53	25	11	1000	20226	701
Chhattisgarh	51	118	97	165	68	117	91	97	93	39	17	98	0	1000	2096	74
Delhi	16	0	0	0	21	0	14	287	26	44	106	0	502	1000	423	17
Goa	202	0	0	2	29	0	20	73	128	62	221	86	378	1000	856	55
Gujarat	42	35	5	37	29	100	42	36	80	164	205	83	184	1000	4109	130
Haryana	90	10	0	19	2	1	9	27	101	198	174	231	229	1000	3286	116
Himachal Pradesh	131	9	13	16	83	18	48	39	107	162	169	86	250	1000	1686	212
Jammu & Kashmir	131	10	0	24	20	51	48	105	150	144	190	113	145	1000	1787	123
Jharkhand	316	40	66	116	127	108	130	126	65	117	75	17	14	1000	13975	589
Karnataka	131	19	24	65	79	106	96	133	104	86	158	45	84	1000	13160	459
Kerala	187	2	3	27	26	21	49	76	66	114	207	138	272	1000	13455	538
Madhya Pradesh	144	139	38	105	119	132	94	101	89	81	62	35	5	1000	16169	498
Maharashtra	100	21	57	68	53	58	101	93	136	124	121	73	95	1000	20845	539
Manipur	414	4	0	1	23	32	50	173	160	251	183	76	46	1000	1588	653
Meghalaya	493	3	0	8	33	53	72	119	186	205	187	85	48	1000	2165	365
Mizoram	380	2	0	0	17	1	35	55	108	225	241	160	155	1000	511	370
Nagaland	533	0	0	0	0	0	4	12	56	92	207	338	291	1000	714	201

Continued

For peer review only

For peer review only - http://bmjopen.bmj.com/site/about/guidelines.xhtml

Appendix A

Table (7): Number per 1000 of households having pet animals and per 1000 distribution of such households by mpce class for each State/UT

Rural+Urban

state/ut	number per 1000 of hhs. having a pet animal	households with pet animals in mpce class													households having a pet animal	
		0-235	235-265	265-320	320-365	365-410	410-460	460-520	520-605	605-730	730-980	980-1285	1285+	total	estd. no. (00)	sample
(1)	(2)	(3)	(4)	(5)	(6)	(7)	(8)	(9)	(10)	(11)	(12)	(13)	(14)	(15)	(16)	(17)
Orissa	62	216	152	104	119	92	54	71	69	29	38	34	21	1000	4588	170
Punjab	92	0	2	1	46	2	67	45	123	139	141	143	291	1000	4379	142
Rajasthan	69	10	23	52	37	77	85	84	152	149	163	52	117	1000	6647	243
Sikkim	232	0	3	41	65	58	30	148	171	196	117	81	90	1000	244	126
Tamil Nadu	89	45	14	25	44	82	101	106	68	115	229	83	89	1000	14189	388
Tripura	303	21	58	102	78	119	136	140	111	99	83	31	23	1000	2131	312
Uttaranchal	182	0	59	81	55	103	164	181	78	102	73	66	38	1000	3089	127
Uttar Pradesh	79	26	51	113	77	125	98	126	110	92	86	56	40	1000	22283	783
West Bengal	92	31	35	80	77	76	138	150	93	112	100	53	55	1000	15473	458
A & N Islands	299	0	0	0	0	67	42	9	129	109	110	276	259	1000	223	100
Chandigarh	17	0	0	0	23	0	0	0	0	102	86	0	789	1000	33	16
Dadra & N. Haveli	240	77	0	0	0	0	2	97	105	163	326	51	178	1000	124	29
Daman & Diu	33	0	0	0	0	0	0	0	106	202	0	485	208	1000	12	5
Lakshadweep	139	0	0	0	22	14	0	37	384	243	68	173	58	1000	13	30
Pondicherry	71	58	0	0	23	152	21	127	154	13	0	66	385	1000	171	20
ONES	398	18	18	32	39	57	76	119	137	168	155	101	79	1000	8284	2522
GUTs	94	34	0	0	9	71	23	63	129	97	119	152	304	1000	576	200
All-India	111	40	35	72	66	84	100	108	107	121	125	64	80	1000	220370	10529

For peer review only

For peer review only - http://bmjopen.bmj.com/site/about/guidelines.xhtml

Appendix A

Table (8): Per 1000 distribution of persons by age-group for each sex

All-India Sub-sample: 1 Rural

age-group	sex			estimated no. (00)			sample		
	males	females	persons	males	females	persons	males	females	persons
(1)	(2)	(3)	(4)	(5)	(6)	(7)	(8)	(9)	(10)
0 - 4	120	120	120	439531	424973	864504	7294	6682	13976
5 - 9	131	125	128	482247	441594	923841	7940	7266	15206
10 -14	125	112	118	459092	394877	853969	7645	6579	14224
15 - 19	94	84	89	345187	297557	642744	6255	5437	11692
20 - 24	78	89	83	285621	313488	599110	5274	5511	10785
25 - 29	77	84	81	283350	297059	580409	4940	5127	10067
30 - 34	68	77	72	250082	271437	521518	4311	4527	8838
35 - 39	66	67	67	243960	238383	482343	4106	3877	7983
40 - 44	57	51	54	209488	181802	391290	3311	2974	6285
45 - 49	46	43	45	169779	153577	323357	2801	2615	5416
50 - 54	33	36	34	120445	127346	247791	2064	2373	4437
55 - 59	36	39	37	132106	137353	269458	2244	2714	4958
60 - 64	24	26	25	88210	93488	181698	2063	1989	4052
65 - 69	20	22	21	73999	78022	152021	1662	1637	3299
70 & above	24	24	24	88987	83968	172955	2030	1803	3833
60 & above	68	72	70	251197	255477	506675	5755	5429	11184
total	1000	1000	1000	3672085	3534923	7207009	63940	61111	125051

Table (8): Per 1000 distribution of persons by age-group for each sex

All-India Sub-sample: 1 Urban

age-group	sex			estimated no. (00)			sample		
	males	females	persons	males	females	persons	males	females	persons
(1)	(2)	(3)	(4)	(5)	(6)	(7)	(8)	(9)	(10)
0 - 4	90	85	87	111386	96132	207518	3451	3043	6494
5 - 9	94	95	94	116102	107435	223537	3243	2954	6197
10 -14	112	111	112	139209	125322	264531	3331	3220	6551
15 - 19	109	99	105	135313	112581	247894	3260	2978	6238
20 - 24	103	100	102	128041	113027	241068	3171	3229	6400
25 - 29	88	87	88	109526	98847	208373	3072	3027	6099
30 - 34	79	81	80	97891	91890	189781	2777	2555	5332
35 - 39	69	79	74	85143	89689	174831	2421	2272	4693
40 - 44	64	64	64	79151	72469	151620	2013	1745	3758
45 - 49	55	49	52	68406	56058	124464	1637	1521	3158
50 - 54	40	43	42	50018	49059	99077	1222	1356	2578
55 - 59	36	38	37	44314	42763	87077	1141	1402	2543
60 - 64	22	23	22	26814	26477	53291	1104	1068	2172
65 - 69	17	19	18	21098	21801	42899	890	901	1791
70 & above	21	26	24	26249	29806	56055	1081	1183	2264
60 & above	60	69	64	74161	78084	152245	3075	3152	6227
total	1000	1000	1000	1238660	1133354	2372014	33814	32454	66268

For peer review only - http://bmjopen.bmj.com/site/about/guidelines.xhtml

Appendix A

Table (8): Per 1000 distribution of persons by age-group for each sex

All-India   Sub-sample: 1   Rural + Urban

age-group	sex			estimated no. (00)			sample		
	males	females	persons	males	females	persons	males	females	persons
(1)	(2)	(3)	(4)	(5)	(6)	(7)	(8)	(9)	(10)
0 - 4	112	112	112	550916	521105	1072021	10745	9725	20470
5 - 9	122	118	120	598349	549029	1147378	11183	10220	21403
10 -14	122	111	117	598301	520199	1118500	10976	9799	20775
15 - 19	98	88	93	480500	410138	890638	9515	8415	17930
20 - 24	84	91	88	413663	426515	840177	8445	8740	17185
25 - 29	80	85	82	392876	395906	788782	8012	8154	16166
30 - 34	71	78	74	347973	363326	711299	7088	7082	14170
35 - 39	67	70	69	329103	328072	657174	6527	6149	12676
40 - 44	59	54	57	288639	254271	542910	5324	4719	10043
45 - 49	49	45	47	238185	209636	447821	4438	4136	8574
50 - 54	35	38	36	170463	176405	346868	3286	3729	7015
55 - 59	36	39	37	176419	180116	356535	3385	4116	7501
60 - 64	23	26	25	115024	119965	234989	3167	3057	6224
65 - 69	19	21	20	95098	99822	194920	2552	2538	5090
70 & above	23	24	24	115236	113774	229010	3111	2986	6097
60 & above	66	71	69	325358	333562	658920	8830	8581	17411
total	1000	1000	1000	4910745	4668277	9579022	97754	93565	191319

Table (8): Per 1000 distribution of persons by age-group for each sex

All-India   Sub-sample: 2   Rural

age-group	sex			estimated no. (00)			sample		
	males	females	persons	males	females	persons	males	females	persons
(1)	(2)	(3)	(4)	(5)	(6)	(7)	(8)	(9)	(10)
0 - 4	118	119	118	427298	413377	840675	7183	6814	13997
5 - 9	132	125	129	477580	435852	913432	8049	7430	15479
10 -14	126	114	120	453180	397420	850600	7687	6781	14468
15 - 19	98	86	92	352467	300992	653459	6336	5531	11867
20 - 24	77	86	81	276053	300635	576688	5194	5526	10720
25 - 29	76	83	79	272739	289370	562109	4979	5171	10150
30 - 34	66	79	72	238709	275255	513965	4269	4547	8816
35 - 39	69	65	67	250141	228068	478209	4186	3851	8037
40 - 44	51	52	52	185572	181834	367406	3204	3068	6272
45 - 49	46	43	45	166336	149799	316135	2776	2627	5403
50 - 54	36	38	37	128734	134017	262751	2152	2415	4567
55 - 59	34	39	37	124401	136028	260428	2210	2657	4867
60 - 64	27	26	26	97145	89899	187043	2137	1982	4119
65 - 69	19	21	20	69521	71750	141271	1600	1564	3164
70 & above	25	23	24	88645	81705	170351	2036	1762	3798
60 & above	71	70	70	255311	243354	498665	5773	5308	11081
total	1000	1000	1000	3608520	3486002	7094522	63998	61726	125724

For peer review only - http://bmjopen.bmj.com/site/about/guidelines.xhtml

Appendix A

Table (8): Per 1000 distribution of persons by age-group for each sex

All-India Sub-sample: 2 Urban

age-group	sex			estimated no. (00)			sample		
	males	females	persons	males	females	persons	males	females	persons
(1)	(2)	(3)	(4)	(5)	(6)	(7)	(8)	(9)	(10)
0 - 4	91	84	88	119253	100925	220178	3524	3058	6582
5 - 9	98	98	98	128446	117550	245996	3308	2967	6275
10 -14	112	109	110	145752	130573	276326	3311	3059	6370
15 - 19	110	100	105	143172	120048	263220	3359	2996	6355
20 - 24	96	104	100	125917	124934	250851	3167	3315	6482
25 - 29	85	85	85	110759	101530	212289	2946	3066	6012
30 - 34	79	82	80	102972	98517	201490	2847	2464	5311
35 - 39	74	77	75	96091	92401	188492	2352	2173	4525
40 - 44	61	61	61	80218	72683	152901	1916	1740	3656
45 - 49	54	51	53	71076	60778	131854	1634	1525	3159
50 - 54	40	40	40	52052	47370	99423	1275	1307	2582
55 - 59	36	38	37	46800	45094	91893	1174	1473	2647
60 - 64	21	26	23	27997	30779	58777	1095	1088	2183
65 - 69	17	22	20	22756	26263	49019	829	937	1766
70 & above	25	25	25	32588	29591	62179	1223	1167	2390
60 & above	64	72	68	83341	86634	169975	3147	3192	6339
total	1000	1000	1000	1305850	1199038	2504888	33960	32335	66295

Table (8): Per 1000 distribution of persons by age-group for each sex

All-India Sub-sample: 2 Rural + Urban

age-group	sex			estimated no. (00)			sample		
	males	females	persons	males	females	persons	males	females	persons
(1)	(2)	(3)	(4)	(5)	(6)	(7)	(8)	(9)	(10)
0 - 4	111	110	111	546551	514302	1060853	10707	9872	20579
5 - 9	123	118	121	606026	553402	1159428	11357	10397	21754
10 -14	122	113	117	598932	527993	1126925	10998	9840	20838
15 - 19	101	90	95	495639	421041	916680	9695	8527	18222
20 - 24	82	91	86	401970	425569	827539	8361	8841	17202
25 - 29	78	83	81	383497	390900	774398	7925	8237	16162
30 - 34	70	80	75	341682	373773	715454	7116	7011	14127
35 - 39	70	68	69	346232	320469	666702	6538	6024	12562
40 - 44	54	54	54	265790	254517	520307	5120	4808	9928
45 - 49	48	45	47	237413	210577	447990	4410	4152	8562
50 - 54	37	39	38	180786	181388	362173	3427	3722	7149
55 - 59	35	39	37	171200	181121	352322	3384	4130	7514
60 - 64	25	26	26	125142	120678	245820	3232	3070	6302
65 - 69	19	21	20	92277	98013	190290	2429	2501	4930
70 & above	25	24	24	121233	111296	232529	3259	2929	6188
60 & above	69	70	70	338652	329988	668639	8920	8500	17420
total	1000	1000	1000	4914370	4685040	9599410	97958	94061	192019

For peer review only - http://bmjopen.bmj.com/site/about/guidelines.xhtml

Appendix A

Table (8): Per 1000 distribution of persons by age-group for each sex

All-India Sub-sample: All Rural

age-group	sex			estimated no. (00)			sample		
	males	females	persons	males	females	persons	males	females	persons
(1)	(2)	(3)	(4)	(5)	(6)	(7)	(8)	(9)	(10)
0 - 4	119	119	119	433414	419175	852589	14477	13496	27973
5 - 9	132	125	128	479914	438723	918637	15989	14696	30685
10 -14	125	113	119	456136	396149	852284	15332	13360	28692
15 - 19	96	85	91	348827	299275	648102	12591	10968	23559
20 - 24	77	87	82	280837	307061	587899	10468	11037	21505
25 - 29	76	84	80	278044	293214	571259	9919	10298	20217
30 - 34	67	78	72	244395	273346	517741	8580	9074	17654
35 - 39	68	66	67	247051	233226	480276	8292	7728	16020
40 - 44	54	52	53	197530	181818	379348	6515	6042	12557
45 - 49	46	43	45	168058	151688	319746	5577	5242	10819
50 - 54	34	37	36	124589	130681	255271	4216	4788	9004
55 - 59	35	39	37	128253	136690	264943	4454	5371	9825
60 - 64	25	26	26	92678	91693	184371	4200	3971	8171
65 - 69	20	21	21	71760	74886	146646	3262	3201	6463
70 & above	24	24	24	88816	82836	171653	4066	3565	7631
60 & above	70	71	70	253254	249416	502670	11528	10737	22265
total	1000	1000	1000	3640303	3510463	7150765	127938	122837	250775

Table (8): Per 1000 distribution of persons by age-group for each sex

All-India Sub-sample: All Urban

age-group	sex			estimated no. (00)			sample		
	males	females	persons	males	females	persons	males	females	persons
(1)	(2)	(3)	(4)	(5)	(6)	(7)	(8)	(9)	(10)
0 - 4	91	84	88	115319	98529	213848	6975	6101	13076
5 - 9	96	96	96	122274	112492	234766	6551	5921	12472
10 -14	112	110	111	142481	127947	270428	6642	6279	12921
15 - 19	109	100	105	139243	116314	255557	6619	5974	12593
20 - 24	100	102	101	126979	118980	245959	6338	6544	12882
25 - 29	87	86	86	110142	100189	210331	6018	6093	12111
30 - 34	79	82	80	100432	95203	195635	5624	5019	10643
35 - 39	71	78	74	90617	91045	181662	4773	4445	9218
40 - 44	63	62	62	79685	72576	152260	3929	3485	7414
45 - 49	55	50	53	69741	58418	128159	3271	3046	6317
50 - 54	40	41	41	51035	48215	99250	2497	2663	5160
55 - 59	36	38	37	45557	43928	89485	2315	2875	5190
60 - 64	22	25	23	27406	28628	56034	2199	2156	4355
65 - 69	17	21	19	21927	24032	45959	1719	1838	3557
70 & above	23	25	24	29418	29699	59117	2304	2350	4654
60 & above	62	71	66	78751	82359	161110	6222	6344	12566
total	1000	1000	1000	1272255	1166196	2438451	67774	64789	132563

For peer review only - http://bmjopen.bmj.com/site/about/guidelines.xhtml

Appendix A

Table (8): Per 1000 distribution of persons by age-group for each sex

All-India Sub-sample: All Rural + Urban

age-group	sex			estimated no. (00)			sample		
	males	females	persons	males	females	persons	males	females	persons
(1)	(2)	(3)	(4)	(5)	(6)	(7)	(8)	(9)	(10)
0 - 4	112	111	111	548734	517704	1066437	21452	19597	41049
5 - 9	123	118	120	602188	551215	1153403	22540	20617	43157
10 -14	122	112	117	598616	524096	1122712	21974	19639	41613
15 - 19	99	89	94	488070	415589	903659	19210	16942	36152
20 - 24	83	91	87	407816	426042	833858	16806	17581	34387
25 - 29	79	84	82	388187	393403	781590	15937	16391	32328
30 - 34	70	79	74	344827	368549	713377	14204	14093	28297
35 - 39	69	69	69	337667	324271	661938	13065	12173	25238
40 - 44	56	54	55	277215	254394	531609	10444	9527	19971
45 - 49	48	45	47	237799	210106	447905	8848	8288	17136
50 - 54	36	38	37	175624	178896	354521	6713	7451	14164
55 - 59	35	39	37	173810	180618	354428	6769	8246	15015
60 - 64	24	26	25	120083	120321	240405	6399	6127	12526
65 - 69	19	21	20	93687	98918	192605	4981	5039	10020
70 & above	24	24	24	118235	112535	230770	6370	5915	12285
60 & above	68	71	69	332005	331775	663779	17750	17081	34831
total	1000	1000	1000	4912558	4676658	9589216	195712	187626	383338

For peer review only

For peer review only - http://bmjopen.bmj.com/site/about/guidelines.xhtml

Table (9): Per 1000 distribution of persons of age 5 years and above by general educational level for each age-group and sex

All-India — Rural — Male

age-group	general educational level												per 1000 no. of persons in the age-group	number of persons (5+)	
	not literate	literate									n.r.	total		estd. (00)	sample
		without formal school-ing	below primary	pri-mary	middle	second-dary	higher second-dary	diploma / certifi-cate course	gra-duate	post-gra-duate & above					
(1)	(2)	(3)	(4)	(5)	(6)	(7)	(8)	(9)	(10)	(11)	(12)	(13)	(14)	(15)	(16)
5-9	394	23	554	27	0	0	0	0	0	0	2	1000	150	479914	15989
10-14	111	5	304	426	153	1	0	0	0	0	0	1000	142	456136	15332
15-19	143	4	78	203	371	151	48	2	1	0	0	1000	109	348827	12591
20-24	188	5	74	172	281	127	105	8	35	6	0	1000	88	280837	10468
25-29	235	8	82	159	245	136	70	7	46	11	0	1000	87	278044	9919
30-34	299	11	95	153	206	105	65	8	47	12	0	1000	76	244395	8580
35-39	360	14	103	157	187	86	41	5	36	10	0	1000	77	247051	8292
40-44	392	16	110	162	165	78	36	3	32	7	0	1000	62	197530	6515
45-49	419	15	106	157	154	82	28	4	30	6	0	1000	52	168058	5577
50-54	456	16	121	139	133	82	30	4	16	3	0	1000	39	124589	4216
55-59	498	17	115	149	103	67	19	5	21	6	1	1000	40	128253	4454
60 & above	588	23	124	122	74	44	11	2	9	2	0	1000	79	253254	11528
15 & above	329	12	97	161	211	102	49	5	27	6	0	1000	708	2270839	82140
7 & above	285	12	186	190	183	77	37	4	20	5	0	1000	937	3003821	106655
5 & above	308	13	195	178	171	72	35	4	19	4	0	1000	1000	3206889	113461
estd. no. of persons (age 5+) (00)	986519	40156	624826	572135	549809	232453	112364	11376	61548	14134	1568	3206889	X	X	X
sample persons (age 5+)	31748	1671	21822	20569	20319	9178	4544	436	2504	596	74	113461	X	X	X

For peer review only

For peer review only - http://bmjopen.bmj.com/site/about/guidelines.xhtml

Appendix A

Table (9): Per 1000 distribution of persons of age 5 years and above by general educational level for each age-group and sex

All-India Rural Female

age-group	general educational level												per 1000 no. of persons in the age-group	number of persons (5+)	
	not literate	literate									n.r.	total			
		without formal school-ing	below primary	pri-mary	middle	second-dary	higher second-dary	diploma / certifi-cate course	gra-duate	post-gra-duate & above				estd. (00)	sample
(1)	(2)	(3)	(4)	(5)	(6)	(7)	(8)	(9)	(10)	(11)	(12)	(13)	(14)	(15)	(16)
5-9	435	20	514	29	0	0	0	0	0	0	2	1000	142	438723	14696
10-14	182	7	281	387	142	1	0	0	0	0	0	1000	128	396149	13360
15-19	266	7	66	183	302	127	48	2	0	0	0	1000	97	299275	10968
20-24	442	7	67	132	189	76	57	4	22	4	0	1000	99	307061	11037
25-29	514	10	73	126	147	72	31	2	20	5	1	1000	95	293214	10298
30-34	605	11	71	113	114	47	20	2	14	3	0	1000	88	273346	9074
35-39	659	8	67	107	96	36	12	1	10	3	0	1000	75	233226	7728
40-44	693	13	67	103	77	29	10	0	5	1	1	1000	59	181818	6042
45-49	723	9	69	108	60	19	4	1	5	1	0	1000	49	151688	5242
50-54	776	9	69	78	43	16	3	0	4	1	0	1000	42	130681	4788
55-59	846	9	52	50	32	6	1	1	2	0	0	1000	44	136690	5371
60 & above	888	9	42	39	13	5	1	1	1	0	0	1000	81	249416	10737
15 & above	602	9	65	111	125	51	23	2	9	2	0	1000	730	2256416	81285
7 & above	516	10	146	143	116	40	18	1	7	2	1	1000	939	2904146	103124
5 & above	525	10	156	134	109	38	17	1	7	2	1	1000	1000	3091287	109341
estd. no. of persons (age 5+) (00)	1622316	32042	482949	415604	337544	116571	52533	3882	21398	4791	1657	3091287	X	X	X
sample persons (age 5+)	53751	1444	17578	15441	12932	4786	2120	159	853	208	69	109341	X	X	X

For peer review only - http://bmjopen.bmj.com/site/about/guidelines.xhtml

For peer review only

Appendix A

Table (9): Per 1000 distribution of persons of age 5 years and above by general educational level for each age-group and sex

All-India | Rural | Person

age-group	general educational level												per 1000 no. of persons in the age-group	number of persons (5+)	
	not literate	literate									n.r.	total		estd. (00)	sample
		without formal school-ing	below primary	pri-mary	middle	second-dary	higher second-dary	diploma / certifi-cate course	gra-duate	post-gra-duate & above					
(1)	(2)	(3)	(4)	(5)	(6)	(7)	(8)	(9)	(10)	(11)	(12)	(13)	(14)	(15)	(16)
5-9	414	22	535	28	0	0	0	0	0	0	2	1000	146	918637	30685
10-14	144	6	294	407	148	1	0	0	0	0	0	1000	135	852284	28692
15-19	200	6	72	194	339	140	48	2	0	0	0	1000	103	648102	23559
20-24	321	6	70	151	233	100	80	6	28	5	0	1000	93	587899	21505
25-29	378	9	78	142	195	103	50	5	32	8	0	1000	91	571259	20217
30-34	460	11	82	132	158	74	41	5	29	7	0	1000	82	517741	17654
35-39	505	11	86	133	143	62	27	3	23	7	0	1000	76	480276	16020
40-44	536	15	89	134	122	55	23	2	19	4	0	1000	60	379348	12557
45-49	563	12	88	134	110	52	16	3	18	3	0	1000	51	319746	10819
50-54	620	13	94	108	87	48	16	2	10	2	0	1000	41	255271	9004
55-59	678	13	82	98	66	36	10	3	11	3	0	1000	42	264943	9825
60 & above	737	16	84	81	44	25	6	2	5	1	0	1000	80	502670	22265
15 & above	465	10	81	136	168	77	36	3	18	4	0	1000	719	4527255	163425
7 & above	399	11	166	167	150	59	28	3	14	3	0	1000	938	5907967	209779
5 & above	414	11	176	157	141	55	26	2	13	3	1	1000	1000	6298176	222802
estd. no. of persons (age 5+) (00)	2608835	72199	1107775	987739	887353	349024	164898	15258	82947	18925	3225	6298176	X	X	X
sample persons (age 5+)	85499	3115	39400	36010	33251	13964	6664	595	3357	804	143	222802	X	X	X

For peer review only - http://bmjopen.bmj.com/site/about/guidelines.xhtml

For peer review only

Appendix A

Table (9): Per 1000 distribution of persons of age 5 years and above by general educational level for each age-group and sex

All-India Urban Male

age-group	general educational level												per 1000 no. of persons in the age-group	number of persons (5+)	
	not literate	literate									n.r.	total			
		without formal school-ing	below primary	pri-mary	middle	second-dary	higher second-dary	diploma / certifi-cate course	gra-duate	post-gra-duate & above				estd. (00)	sample
(1)	(2)	(3)	(4)	(5)	(6)	(7)	(8)	(9)	(10)	(11)	(12)	(13)	(14)	(15)	(16)
5-9	251	22	676	49	0	0	0	0	0	0	2	1000	106	122274	6551
10-14	63	5	224	453	253	1	0	0	0	0	1	1000	123	142481	6642
15-19	76	3	50	155	332	248	131	4	1	0	0	1000	120	139243	6619
20-24	79	6	38	132	238	149	207	12	128	12	0	1000	110	126979	6338
25-29	96	4	56	112	217	154	118	27	169	48	0	1000	95	110142	6018
30-34	108	7	68	118	217	144	100	22	166	51	0	1000	87	100432	5624
35-39	153	5	60	126	180	141	86	26	174	48	0	1000	78	90617	4773
40-44	167	9	63	139	172	146	95	15	138	55	0	1000	69	79685	3929
45-49	148	4	87	108	168	164	72	17	163	68	0	1000	60	69741	3271
50-54	137	12	61	138	138	156	74	25	176	81	0	1000	44	51035	2497
55-59	186	10	81	145	144	148	74	18	145	50	0	1000	39	45557	2315
60 & above	232	22	105	138	127	160	50	22	104	41	0	1000	68	78751	6222
15 & above	127	7	63	131	210	166	111	18	127	39	0	1000	771	892181	47606
7 & above	118	8	131	169	202	134	89	14	103	32	0	1000	957	1106870	58031
5 & above	132	9	148	162	193	128	86	14	98	30	0	1000	1000	1156936	60799
estd. no. of persons (age 5+) (00)	152741	9994	170775	187733	223605	148105	99000	15985	113548	34989	460	1156936	X	X	X
sample persons (age 5+)	7670	599	8821	9355	11949	8303	5287	821	6158	1804	32	60799	X	X	X

For peer review only - http://bmjopen.bmj.com/site/about/guidelines.xhtml

Appendix A

Table (9): Per 1000 distribution of persons of age 5 years and above by general educational level for each age-group and sex

All-India | Urban | Female

age-group	general educational level												per 1000 no. of persons in the age-group	number of persons (5+)	
	not literate	literate									n.r.	total		estd. (00)	sample
		without formal school-ing	below primary	pri-mary	middle	second-dary	higher second-dary	diploma / certifi-cate course	gra-duate	post-gra-duate & above					
(1)	(2)	(3)	(4)	(5)	(6)	(7)	(8)	(9)	(10)	(11)	(12)	(13)	(14)	(15)	(16)
5-9	277	21	651	50	0	0	0	0	0	0	2	1000	105	112492	5921
10-14	72	6	211	467	238	5	0	0	0	0	0	1000	120	127947	6279
15-19	98	1	35	120	323	254	163	3	2	0	0	1000	109	116314	5974
20-24	158	6	47	113	189	138	166	10	147	24	0	1000	111	118980	6544
25-29	198	4	44	124	200	134	93	6	142	54	1	1000	94	100189	6093
30-34	270	7	49	104	180	126	79	15	133	37	0	1000	89	95203	5019
35-39	318	11	67	131	169	117	60	5	89	34	0	1000	85	91045	4445
40-44	326	9	61	117	139	147	61	9	86	44	0	1000	68	72576	3485
45-49	349	13	67	126	143	117	62	4	95	25	0	1000	55	58418	3046
50-54	393	8	74	118	127	120	48	7	68	37	0	1000	45	48215	2663
55-59	492	11	79	125	117	79	31	3	48	15	1	1000	41	43928	2875
60 & above	584	21	97	104	81	55	19	3	28	7	0	1000	77	82359	6344
15 & above	287	8	58	118	180	137	90	7	87	27	0	1000	775	827228	46488
7 & above	251	9	123	159	176	112	73	6	71	22	0	1000	956	1020906	56215
5 & above	260	9	139	152	168	107	70	5	68	21	0	1000	1000	1067667	58688
estd. no. of persons (age 5+) (00)	277707	10115	148459	162557	179484	113917	74383	5724	72377	22440	504	1067667	X	X	X
sample persons (age 5+)	14885	625	8174	8914	9996	6387	4139	329	3942	1256	41	58688	X	X	X

For peer review only

For peer review only - http://bmjopen.bmj.com/site/about/guidelines.xhtml

Appendix A

Table (9): Per 1000 distribution of persons of age 5 years and above by general educational level for each age-group and sex

All-India Urban Person

age-group	general educational level												per 1000 no. of persons in the age-group	number of persons (5+)	
	not literate	literate									n.r.	total			
		without formal school-ing	below primary	pri-mary	middle	second-dary	higher second-dary	diploma / certifi-cate course	gra-duate	post-gra-duate & above				estd. (00)	sample
(1)	(2)	(3)	(4)	(5)	(6)	(7)	(8)	(9)	(10)	(11)	(12)	(13)	(14)	(15)	(16)
5-9	263	21	664	50	0	0	0	0	0	0	2	1000	106	234766	12472
10-14	67	6	218	460	246	3	0	0	0	0	0	1000	122	270428	12921
15-19	86	2	43	139	328	251	146	4	1	0	0	1000	115	255557	12593
20-24	117	6	42	123	214	143	187	11	137	18	0	1000	111	245959	12882
25-29	144	4	50	118	209	144	106	17	156	50	1	1000	95	210331	12111
30-34	187	7	58	111	199	135	89	19	150	44	0	1000	88	195635	10643
35-39	236	8	63	129	175	129	73	16	131	41	0	1000	82	181662	9218
40-44	243	9	62	129	156	147	79	12	114	49	0	1000	68	152260	7414
45-49	240	8	78	116	156	143	68	11	132	48	0	1000	58	128159	6317
50-54	261	10	68	128	133	139	62	16	124	60	0	1000	45	99250	5160
55-59	336	10	80	135	131	114	53	11	98	33	1	1000	40	89485	5190
60 & above	412	22	101	120	103	107	34	12	65	23	0	1000	72	161110	12566
15 & above	204	8	61	125	196	152	101	13	108	33	0	1000	773	1719409	94094
7 & above	182	8	127	164	189	123	81	10	87	27	0	1000	956	2127777	114246
5 & above	193	9	144	157	181	118	78	10	84	26	0	1000	1000	2224603	119487
estd. no. of persons (age 5+) (00)	430448	20109	319234	350291	403089	262022	173384	21709	185925	57429	964	2224603	X	X	X
sample persons (age 5+)	22555	1224	16995	18269	21945	14690	9426	1150	10100	3060	73	119487	X	X	X

For peer review only - http://bmjopen.bmj.com/site/about/guidelines.xhtml

Appendix A

Table (9): Per 1000 distribution of persons of age 5 years and above by general educational level for each age-group and sex

All-India | Rural + Urban | Male

age-group	general educational level												per 1000 no. of persons in the age-group	number of persons (5+)	
	not literate	literate									n.r.	total		estd. (00)	sample
		without formal schooling	below primary	primary	middle	secondary	higher secondary	diploma / certificate course	graduate	post-graduate & above					
(1)	(2)	(3)	(4)	(5)	(6)	(7)	(8)	(9)	(10)	(11)	(12)	(13)	(14)	(15)	(16)
5-9	365	23	579	31	0	0	0	0	0	0	2	1000	138	602188	22540
10-14	99	5	285	432	177	1	0	0	0	0	0	1000	137	598616	21974
15-19	124	4	70	189	359	179	72	3	1	0	0	1000	112	488070	19210
20-24	154	5	63	159	267	134	136	10	64	7	0	1000	93	407816	16806
25-29	196	7	75	146	237	141	84	13	81	22	0	1000	89	388187	15937
30-34	243	10	87	143	210	117	75	12	82	23	0	1000	79	344827	14204
35-39	304	12	92	149	185	101	53	11	73	20	0	1000	77	337667	13065
40-44	327	14	96	156	167	98	53	6	62	20	0	1000	64	277215	10444
45-49	339	12	100	143	158	106	41	8	69	24	0	1000	54	237799	8848
50-54	363	15	104	138	134	104	43	10	63	26	0	1000	40	175624	6713
55-59	416	15	106	148	114	88	33	9	54	18	0	1000	40	173810	6769
60 & above	503	22	120	126	86	71	20	7	32	11	0	1000	76	332005	17750
15 & above	272	11	87	153	211	120	67	9	55	16	0	1000	725	3163020	129746
7 & above	240	11	171	185	188	93	51	7	43	12	0	1000	942	4110691	164686
5 & above	261	11	182	174	177	87	48	6	40	11	0	1000	1000	4363824	174260
estd. no. of persons (age 5+) (00)	1139260	50150	795601	759868	773414	380558	211365	27361	175096	49123	2029	4363824	X	X	X
sample persons (age 5+)	39418	2270	30643	29924	32268	17481	9831	1257	8662	2400	106	174260	X	X	X

For peer review only - http://bmjopen.bmj.com/site/about/guidelines.xhtml

For peer review only

Appendix A

Table (9): Per 1000 distribution of persons of age 5 years and above by general educational level for each age-group and sex

All-India | Rural + Urban | Female

age-group	general educational level												per 1000 no. of persons in the age-group	number of persons (5+)	
	not literate	literate									n.r.	total		estd. (00)	sample
		without formal school-ing	below primary	pri-mary	middle	second-dary	higher second-dary	diploma / certifi-cate course	gra-duate	post-gra-duate & above					
(1)	(2)	(3)	(4)	(5)	(6)	(7)	(8)	(9)	(10)	(11)	(12)	(13)	(14)	(15)	(16)
5-9	403	20	542	33	0	0	0	0	0	0	2	1000	133	551215	20617
10-14	155	7	264	406	166	2	0	0	0	0	0	1000	126	524096	19639
15-19	219	6	57	165	308	162	80	2	1	0	0	1000	100	415589	16942
20-24	363	7	61	127	189	93	88	6	57	10	0	1000	102	426042	17581
25-29	433	8	66	125	160	88	47	3	51	17	1	1000	95	393403	16391
30-34	518	10	65	111	131	67	35	6	45	12	0	1000	89	368549	14093
35-39	563	9	67	113	116	59	26	2	32	12	0	1000	78	324271	12173
40-44	589	12	65	107	94	63	25	3	28	13	1	1000	61	254394	9527
45-49	619	10	68	113	83	46	20	2	30	7	0	1000	51	210106	8288
50-54	673	9	70	89	66	44	15	2	22	10	0	1000	43	178896	7451
55-59	760	9	58	69	53	24	8	2	13	4	0	1000	43	180618	8246
60 & above	813	12	56	55	30	17	6	2	7	2	0	1000	80	331775	17081
15 & above	518	9	63	113	140	74	41	3	30	9	0	1000	741	3083643	127773
7 & above	447	9	140	147	132	59	32	2	24	7	0	1000	944	3925053	159339
5 & above	457	10	152	139	124	55	31	2	23	7	1	1000	1000	4158955	168029
estd. no. of persons (age 5+) (00)	1900023	42157	631408	578161	517028	230488	126917	9606	93775	27230	2161	4158955	X	X	X
sample persons (age 5+)	68636	2069	25752	24355	22928	11173	6259	488	4795	1464	110	168029	X	X	X

For peer review only - http://bmjopen.bmj.com/site/about/guidelines.xhtml

For peer review only

Appendix A

Table (9): Per 1000 distribution of persons of age 5 years and above by general educational level for each age-group and sex

All-India Rural + Urban Person

age-group	general educational level												per 1000 no. of persons in the age-group	number of persons (5+)	
	not literate	literate									n.r.	total		estd. (00)	sample
		without formal school-ing	below primary	pri-mary	middle	second-dary	higher second-dary	diploma / certifi-cate course	gra-duate	post-gra-duate & above					
(1)	(2)	(3)	(4)	(5)	(6)	(7)	(8)	(9)	(10)	(11)	(12)	(13)	(14)	(15)	(16)
5-9	383	22	561	32	0	0	0	0	0	0	2	1000	135	1153403	43157
10-14	125	6	276	420	172	1	0	0	0	0	0	1000	132	1122712	41613
15-19	167	5	64	178	336	171	76	2	1	0	0	1000	106	903659	36152
20-24	261	6	62	143	227	113	111	8	60	9	0	1000	98	833858	34387
25-29	315	7	70	136	198	114	65	8	66	19	0	1000	92	781590	32328
30-34	385	10	76	126	169	91	54	9	63	17	0	1000	84	713377	28297
35-39	431	10	80	132	151	80	40	6	53	16	0	1000	78	661938	25238
40-44	452	13	81	132	132	81	39	5	46	17	0	1000	62	531609	19971
45-49	471	11	85	129	123	78	31	5	51	16	0	1000	53	447905	17136
50-54	519	12	87	113	100	73	29	6	42	18	0	1000	42	354521	14164
55-59	591	12	81	108	83	55	20	5	33	11	0	1000	42	354428	15015
60 & above	658	17	88	91	58	44	13	4	20	6	0	1000	78	663779	34831
15 & above	393	10	75	133	176	98	54	6	43	12	0	1000	733	6246664	257519
7 & above	341	10	156	166	161	76	42	5	33	10	0	1000	943	8035744	324025
5 & above	357	11	167	157	151	72	40	4	32	9	0	1000	1000	8522779	342289
estd. no. of persons (age 5+) (00)	3039283	92308	1427008	1338029	1290442	611046	338282	36967	268871	76353	4189	8522779	X	X	X
sample persons (age 5+)	108054	4339	56395	54279	55196	28654	16090	1745	13457	3864	216	342289	X	X	X

For peer review only - http://bmjopen.bmj.com/site/about/guidelines.xhtml

For peer review only

Appendix A

Table (10): Per 1000 distribution of aged persons by number of living children for each sex and State/UT

Rural | Males

state/ut	aged persons with number of living children										no. of aged persons	
	0	1	2	3	4	5	6-7	8-9	10 & above	total	estd. (00)	sample
(1)	(2)	(3)	(4)	(5)	(6)	(7)	(8)	(9)	(10)	(11)	(12)	(13)
Andhra Pradesh	60	93	145	226	196	133	117	19	11	1000	18730	697
Arunachal Pradesh	265	140	117	94	131	116	118	18	2	1000	238	140
Assam	39	24	72	219	273	219	121	27	6	1000	6103	471
Bihar	49	54	127	193	200	191	141	38	8	1000	18861	912
Chhattisgarh	83	75	66	246	228	111	175	9	8	1000	4609	204
Delhi	0	117	0	207	90	375	211	0	0	1000	338	16
Goa	46	0	141	298	407	18	63	28	0	1000	506	19
Gujarat	33	47	134	181	232	159	156	46	13	1000	10458	351
Haryana	22	19	81	158	194	185	263	73	4	1000	5608	252
Himachal Pradesh	78	93	142	163	178	196	119	25	5	1000	2400	347
Jammu & Kashmir	36	92	81	239	191	196	135	30	0	1000	2246	230
Jharkhand	81	66	107	209	155	218	138	16	10	1000	6400	330
Karnataka	39	55	183	209	171	135	176	27	6	1000	11642	428
Kerala	28	59	149	155	221	136	197	46	11	1000	11673	505
Madhya Pradesh	76	72	95	171	175	149	205	49	10	1000	14666	549
Maharashtra	25	43	129	216	216	202	113	44	13	1000	23347	717
Manipur	225	61	61	112	88	205	197	43	8	1000	395	211
Meghalaya	84	81	152	192	105	131	212	35	9	1000	467	96
Mizoram	115	192	50	151	101	167	215	2	7	1000	119	89
Nagaland	132	48	123	114	136	54	306	86	0	1000	109	56
Orissa	63	81	142	210	219	121	120	39	5	1000	13371	525
Punjab	36	50	112	247	173	167	183	25	7	1000	6843	258
Rajasthan	61	91	108	133	156	141	239	66	4	1000	12196	552
Sikkim	28	69	64	128	244	225	159	76	7	1000	115	110
Tamil Nadu	25	95	195	255	180	139	95	15	1	1000	16961	571
Tripura	31	75	111	227	166	233	121	32	4	1000	927	171
Uttaranchal	80	41	110	161	221	196	109	82	0	1000	2674	102
Uttar Pradesh	81	75	95	146	175	173	190	48	17	1000	42788	1825
West Bengal	45	53	116	193	182	169	163	58	21	1000	18130	680
A & N Islands	0	0	84	267	251	198	108	92	0	1000	76	34

Continued

For peer review only

For peer review only - http://bmjopen.bmj.com/site/about/guidelines.xhtml

Appendix A

Table (10): Per 1000 distribution of aged persons by number of living children for each sex and State/UT

Rural — Males

state/ut	aged persons with number of living children										no. of aged persons	
	0	1	2	3	4	5	6-7	8-9	10 & above	total	estd. (00)	sample
(1)	(2)	(3)	(4)	(5)	(6)	(7)	(8)	(9)	(10)	(11)	(12)	(13)
Chandigarh	74	0	0	66	404	68	375	12	0	1000	7	17
Dadra & N. Haveli	0	100	43	409	255	135	59	0	0	1000	78	20
Daman & Diu	73	0	280	158	0	169	319	0	0	1000	4	7
Lakshadweep	160	0	126	184	60	361	110	0	0	1000	7	16
Pondicherry	0	270	133	208	196	74	68	51	0	1000	160	20
ONES	106	85	106	174	137	184	166	36	6	1000	2371	873
GUTs	6	153	99	264	222	124	86	46	0	1000	333	114
all-India	53	66	124	192	193	164	159	40	10	1000	253254	11528
estd. no. of aged persons (00)	13428	16779	31286	48517	48759	41491	40221	10162	2611	253254	X	X
sample aged persons	661	760	1320	2078	2207	2002	1861	499	140	11528	X	X

For peer review only - http://bmjopen.bmj.com/site/about/guidelines.xhtml

Appendix A

Table (10): Per 1000 distribution of aged persons by number of living children for each sex and State/UT

Rural												Females
state/ut	aged persons with number of living children										no. of aged persons	
	0	1	2	3	4	5	6-7	8-9	10 & above	total	estd. (00)	sample
(1)	(2)	(3)	(4)	(5)	(6)	(7)	(8)	(9)	(10)	(11)	(12)	(13)
Andhra Pradesh	63	150	164	195	162	125	125	15	3	1000	20035	688
Arunachal Pradesh	155	131	27	181	189	201	86	29	0	1000	204	118
Assam	59	56	63	194	278	215	112	21	0	1000	4273	323
Bihar	52	80	131	211	156	208	118	37	7	1000	15258	678
Chhattisgarh	121	70	113	146	217	160	154	4	15	1000	6680	290
Delhi	0	0	14	240	146	339	261	0	0	1000	322	17
Goa	106	44	70	267	371	122	0	21	0	1000	615	32
Gujarat	33	70	126	164	194	169	183	55	8	1000	10401	370
Haryana	0	12	63	117	210	204	290	91	13	1000	5510	232
Himachal Pradesh	88	178	94	124	128	177	173	32	6	1000	2546	346
Jammu & Kashmir	14	120	110	185	123	197	171	75	6	1000	1555	173
Jharkhand	60	76	91	197	191	235	107	41	1	1000	4441	231
Karnataka	52	107	190	178	166	147	121	34	5	1000	10878	403
Kerala	48	74	128	157	196	133	199	48	17	1000	15013	649
Madhya Pradesh	69	111	103	179	160	161	158	48	12	1000	14701	544
Maharashtra	73	85	133	157	204	174	128	37	9	1000	24333	716
Manipur	170	54	113	102	96	198	193	67	7	1000	279	163
Meghalaya	74	79	128	110	74	198	271	60	6	1000	431	95
Mizoram	197	114	113	134	110	131	192	10	0	1000	76	48
Nagaland	164	0	81	29	0	20	561	146	0	1000	39	24
Orissa	63	153	145	164	206	108	125	28	10	1000	12157	483
Punjab	4	48	130	205	167	217	195	33	0	1000	6518	239
Rajasthan	36	73	85	156	165	157	230	83	16	1000	12999	583
Sikkim	124	70	59	161	200	108	181	69	29	1000	109	94
Tamil Nadu	59	153	185	191	156	120	114	18	3	1000	17146	549
Tripura	31	115	93	184	201	230	128	19	0	1000	675	135
Uttaranchal	50	121	185	234	205	100	67	38	0	1000	2190	90
Uttar Pradesh	53	89	112	167	176	193	162	34	13	1000	41298	1649
West Bengal	63	63	129	197	144	137	178	63	28	1000	18472	686
A & N Islands	156	110	10	106	63	313	241	0	0	1000	43	15

Continued

For peer review only - http://bmjopen.bmj.com/site/about/guidelines.xhtml

Appendix A

Table (10): Per 1000 distribution of aged persons by number of living children for each sex and State/UT

Rural — Females

state/ut	aged persons with number of living children										no. of aged persons	
	0	1	2	3	4	5	6-7	8-9	10 & above	total	estd. (00)	sample
(1)	(2)	(3)	(4)	(5)	(6)	(7)	(8)	(9)	(10)	(11)	(12)	(13)
Chandigarh	0	195	61	24	280	146	284	11	0	1000	8	11
Dadra & N. Haveli	40	0	82	384	411	28	27	28	0	1000	39	10
Daman & Diu	0	97	156	116	0	106	369	156	0	1000	13	13
Lakshadweep	114	126	176	183	120	68	212	0	0	1000	9	22
Pondicherry	55	129	151	183	110	42	331	0	0	1000	148	18
ONES	92	94	95	147	145	198	182	43	4	1000	1813	677
GUTs	67	107	116	192	147	92	267	13	0	1000	261	89
all-India	56	95	129	175	177	163	155	39	10	1000	249416	10737
estd. no. of aged persons (00)	14024	23751	32051	43710	44245	40640	38607	9795	2592	249416	X	X
sample aged persons	649	931	1245	1816	1937	1809	1760	465	125	10737	X	X

For peer review only - http://bmjopen.bmj.com/site/about/guidelines.xhtml

Appendix A

Table (10): Per 1000 distribution of aged persons by number of living children for each sex and State/UT

Rural												Persons
state/ut	aged persons with number of living children										no. of aged persons	
	0	1	2	3	4	5	6-7	8-9	10 & above	total	estd. (00)	sample
(1)	(2)	(3)	(4)	(5)	(6)	(7)	(8)	(9)	(10)	(11)	(12)	(13)
Andhra Pradesh	61	123	154	210	178	129	121	17	7	1000	38765	1385
Arunachal Pradesh	214	136	75	134	158	155	103	23	1	1000	442	258
Assam	47	37	68	209	275	218	117	25	3	1000	10376	794
Bihar	50	66	128	201	180	199	131	37	8	1000	34119	1590
Chhattisgarh	105	72	93	187	222	140	163	6	12	1000	11289	494
Delhi	0	60	7	223	117	358	235	0	0	1000	661	33
Goa	79	24	102	281	387	75	28	24	0	1000	1121	51
Gujarat	33	58	130	172	213	164	169	50	10	1000	20859	721
Haryana	11	16	72	138	202	194	277	82	8	1000	11118	484
Himachal Pradesh	83	137	118	143	152	186	147	29	5	1000	4946	693
Jammu & Kashmir	27	104	93	217	163	197	150	49	2	1000	3801	403
Jharkhand	73	70	100	204	170	225	125	27	6	1000	10841	561
Karnataka	45	80	186	194	168	141	149	31	5	1000	22520	831
Kerala	39	68	137	156	207	134	198	47	14	1000	26687	1154
Madhya Pradesh	72	91	99	175	167	155	181	48	11	1000	29367	1093
Maharashtra	50	64	131	186	210	188	120	40	11	1000	47679	1433
Manipur	202	58	82	108	92	202	195	53	7	1000	673	374
Meghalaya	79	80	140	153	90	163	240	47	8	1000	898	191
Mizoram	147	161	75	144	105	153	206	5	4	1000	196	137
Nagaland	141	36	112	92	100	45	373	102	0	1000	148	80
Orissa	63	115	143	188	213	115	122	33	7	1000	25527	1008
Punjab	21	49	121	227	170	191	189	29	4	1000	13361	497
Rajasthan	48	82	96	145	161	149	235	74	10	1000	25196	1135
Sikkim	74	69	61	144	223	168	170	73	18	1000	224	204
Tamil Nadu	43	124	190	222	168	130	105	17	2	1000	34107	1120
Tripura	31	92	103	209	181	231	124	26	2	1000	1602	306
Uttaranchal	67	77	144	194	214	153	90	62	0	1000	4865	192
Uttar Pradesh	67	82	104	157	176	183	177	41	15	1000	84086	3474
West Bengal	54	58	123	195	163	153	170	60	24	1000	36602	1366
A & N Islands	56	40	57	209	183	240	156	59	0	1000	120	49

Continued

For peer review only - http://bmjopen.bmj.com/site/about/guidelines.xhtml

Appendix A

Table (10): Per 1000 distribution of aged persons by number of living children for each sex and State/UT

Rural　　　　Persons

state/ut	aged persons with number of living children										no. of aged persons	
	0	1	2	3	4	5	6-7	8-9	10 & above	total	estd. (00)	sample
(1)	(2)	(3)	(4)	(5)	(6)	(7)	(8)	(9)	(10)	(11)	(12)	(13)
Chandigarh	35	104	32	44	338	109	326	12	0	1000	16	28
Dadra & N. Haveli	13	67	56	400	307	100	48	9	0	1000	117	30
Daman & Diu	17	74	185	126	0	121	357	120	0	1000	17	20
Lakshadweep	134	70	154	184	93	198	167	0	0	1000	16	38
Pondicherry	26	202	142	196	155	58	194	27	0	1000	309	38
ONES	100	89	102	162	140	190	173	39	5	1000	4184	1550
GUTs	33	133	107	232	189	110	165	31	0	1000	594	203
all-India	55	81	126	183	185	163	157	40	10	1000	502670	22265
estd. no. of aged persons (00)	27451	40531	63336	92227	93003	82132	78828	19957	5204	502670	X	X
sample aged persons	1310	1691	2565	3894	4144	3811	3621	964	265	22265	X	X

For peer review only - http://bmjopen.bmj.com/site/about/guidelines.xhtml

For peer review only

Appendix A

Table (10): Per 1000 distribution of aged persons by number of living children for each sex and State/UT

Urban — Males

state/ut	aged persons with number of living children										no. of aged persons	
	0	1	2	3	4	5	6-7	8-9	10 & above	total	estd. (00)	sample
(1)	(2)	(3)	(4)	(5)	(6)	(7)	(8)	(9)	(10)	(11)	(12)	(13)
Andhra Pradesh	27	80	186	206	235	89	126	39	13	1000	5310	377
Arunachal Pradesh	178	80	83	229	99	259	73	0	0	1000	10	42
Assam	30	27	177	283	146	154	182	0	0	1000	647	109
Bihar	75	26	70	188	176	239	168	27	29	1000	2455	169
Chhattisgarh	9	2	186	175	275	255	73	26	0	1000	641	63
Delhi	34	137	227	177	158	119	147	1	0	1000	2212	211
Goa	144	100	77	219	289	167	5	0	0	1000	229	32
Gujarat	30	58	178	260	162	154	94	61	2	1000	4592	297
Haryana	23	25	21	200	271	224	198	35	4	1000	1315	132
Himachal Pradesh	52	60	191	139	200	193	110	43	12	1000	139	49
Jammu & Kashmir	56	41	197	90	179	286	107	43	0	1000	408	102
Jharkhand	11	117	69	149	229	225	162	7	31	1000	1372	166
Karnataka	25	77	181	280	214	93	71	56	2	1000	4025	345
Kerala	41	72	227	262	152	102	121	18	6	1000	3986	290
Madhya Pradesh	63	76	129	201	207	102	182	28	11	1000	4115	319
Maharashtra	45	65	169	225	276	94	103	17	6	1000	12844	596
Manipur	105	59	76	167	218	168	129	77	0	1000	192	116
Meghalaya	27	12	156	382	115	137	144	17	10	1000	42	34
Mizoram	199	111	96	148	82	220	126	10	7	1000	85	142
Nagaland	0	35	128	197	153	172	174	143	0	1000	68	24
Orissa	15	134	120	197	196	72	193	64	7	1000	1546	148
Punjab	52	41	119	176	208	180	177	14	33	1000	2565	165
Rajasthan	24	66	86	190	207	139	234	49	5	1000	3012	271
Sikkim	168	136	220	78	81	233	84	0	0	1000	11	16
Tamil Nadu	62	90	233	249	158	86	100	18	3	1000	7955	587
Tripura	0	68	158	207	253	225	68	21	0	1000	130	45
Uttaranchal	20	129	204	146	186	178	135	2	0	1000	558	49
Uttar Pradesh	57	73	94	152	162	195	195	36	39	1000	9104	620
West Bengal	89	110	270	169	107	109	117	23	6	1000	8725	522
A & N Islands	71	0	171	259	183	110	207	0	0	1000	18	18

Continued

For peer review only - http://bmjopen.bmj.com/site/about/guidelines.xhtml

Appendix A

Table (10): Per 1000 distribution of aged persons by number of living children for each sex and State/UT

Urban Males

state/ut	aged persons with number of living children										no. of aged persons	
	0	1	2	3	4	5	6-7	8-9	10 & above	total	estd. (00)	sample
(1)	(2)	(3)	(4)	(5)	(6)	(7)	(8)	(9)	(10)	(11)	(12)	(13)
Chandigarh	0	39	262	352	214	87	47	0	0	1000	197	69
Dadra & N. Haveli	0	0	84	330	227	360	0	0	0	1000	4	13
Daman & Diu	74	0	227	36	378	111	74	0	100	1000	10	16
Lakshadweep	0	203	25	293	101	149	25	35	169	1000	8	21
Pondicherry	57	41	63	184	197	179	224	55	0	1000	218	47
ONES	78	64	115	194	184	191	119	54	2	1000	538	419
GUTs	32	40	156	260	206	136	138	27	5	1000	455	184
all-India	49	77	170	208	193	129	135	29	11	1000	78751	6222
estd. no. of aged persons (00)	3856	6036	13360	16376	15166	10148	10642	2273	894	78751	X	X
sample aged persons	327	463	975	1323	1156	863	838	204	73	6222	X	X

For peer review only - http://bmjopen.bmj.com/site/about/guidelines.xhtml

Appendix A

Table (10): Per 1000 distribution of aged persons by number of living children for each sex and State/UT

Urban — Females

state/ut	aged persons with number of living children										no. of aged persons	
	0	1	2	3	4	5	6-7	8-9	10 & above	total	estd. (00)	sample
(1)	(2)	(3)	(4)	(5)	(6)	(7)	(8)	(9)	(10)	(11)	(12)	(13)
Andhra Pradesh	50	136	171	145	199	124	149	23	3	1000	6160	421
Arunachal Pradesh	79	109	113	233	263	123	79	0	0	1000	7	30
Assam	77	33	28	150	198	366	125	4	21	1000	532	85
Bihar	65	42	65	168	209	235	127	41	49	1000	1829	145
Chhattisgarh	80	101	174	123	98	243	85	79	18	1000	760	88
Delhi	51	77	212	199	233	126	86	6	10	1000	2129	207
Goa	106	256	29	122	273	151	38	25	0	1000	256	40
Gujarat	43	96	108	171	214	167	136	65	0	1000	5101	319
Haryana	57	33	57	141	189	276	200	47	0	1000	1725	143
Himachal Pradesh	23	72	80	218	195	148	265	0	0	1000	155	50
Jammu & Kashmir	46	29	72	236	169	209	175	63	0	1000	336	91
Jharkhand	34	105	114	120	196	238	102	62	29	1000	1292	139
Karnataka	49	122	147	214	164	131	151	17	4	1000	4073	353
Kerala	109	79	121	195	141	133	207	13	4	1000	4686	322
Madhya Pradesh	99	108	125	141	181	124	173	47	1	1000	4466	327
Maharashtra	64	84	101	177	249	122	166	33	4	1000	13669	672
Manipur	171	69	84	136	92	181	161	105	0	1000	178	115
Meghalaya	23	94	6	242	252	154	219	0	10	1000	48	46
Mizoram	155	253	92	112	77	185	98	13	14	1000	60	104
Nagaland	0	0	368	112	0	368	42	112	0	1000	8	5
Orissa	57	114	182	258	70	91	191	33	4	1000	1182	113
Punjab	25	66	72	177	202	192	137	98	31	1000	2347	164
Rajasthan	32	98	93	153	201	165	211	40	8	1000	3076	266
Sikkim	0	204	204	78	19	175	301	19	0	1000	7	13
Tamil Nadu	79	116	209	177	161	91	131	30	4	1000	8984	650
Tripura	8	202	164	90	157	131	217	31	0	1000	136	54
Uttaranchal	32	55	42	239	289	136	206	0	0	1000	693	49
Uttar Pradesh	69	70	140	158	139	191	170	37	27	1000	9590	621
West Bengal	83	103	225	135	152	124	154	21	3	1000	8315	506
A & N Islands	49	62	72	255	143	74	296	0	49	1000	15	22

Continued

For peer review only - http://bmjopen.bmj.com/site/about/guidelines.xhtml

Appendix A

Table (10): Per 1000 distribution of aged persons by number of living children for each sex and State/UT

Urban | Females

state/ut	aged persons with number of living children										no. of aged persons	
	0	1	2	3	4	5	6-7	8-9	10 & above	total	estd. (00)	sample
(1)	(2)	(3)	(4)	(5)	(6)	(7)	(8)	(9)	(10)	(11)	(12)	(13)
Chandigarh	53	22	214	240	251	114	69	37	0	1000	147	68
Dadra & N. Haveli	65	0	158	237	213	168	158	0	0	1000	3	14
Daman & Diu	42	100	190	233	86	33	316	0	0	1000	16	22
Lakshadweep	147	64	52	222	69	245	188	12	0	1000	10	24
Pondicherry	77	22	133	210	254	152	119	32	0	1000	368	56
ONES	96	139	109	130	127	166	175	56	3	1000	444	367
GUTs	70	26	153	220	242	138	118	31	1	1000	560	206
all-India	66	94	141	168	185	145	157	34	9	1000	82359	6344
estd. no. of aged persons (00)	5459	7712	11646	13820	15255	11978	12936	2836	718	82359	X	X
sample aged persons	429	593	853	1124	1114	942	975	253	61	6344	X	X

For peer review only - http://bmjopen.bmj.com/site/about/guidelines.xhtml

Appendix A

Table (10): Per 1000 distribution of aged persons by number of living children for each sex and State/UT

Urban Persons

state/ut	aged persons with number of living children										no. of aged persons	
	0	1	2	3	4	5	6-7	8-9	10 & above	total	estd. (00)	sample
(1)	(2)	(3)	(4)	(5)	(6)	(7)	(8)	(9)	(10)	(11)	(12)	(13)
Andhra Pradesh	39	110	178	174	215	108	138	30	8	1000	11470	798
Arunachal Pradesh	137	92	95	231	166	203	76	0	0	1000	17	72
Assam	51	30	110	223	170	250	156	2	9	1000	1180	194
Bihar	71	33	68	179	190	237	151	33	38	1000	4283	314
Chhattisgarh	47	55	179	147	179	249	79	54	10	1000	1401	151
Delhi	43	108	220	188	195	122	117	4	5	1000	4342	418
Goa	124	182	52	168	280	159	22	13	0	1000	485	72
Gujarat	37	78	141	213	189	161	116	63	1	1000	9693	616
Haryana	42	30	42	167	224	253	199	42	2	1000	3040	275
Himachal Pradesh	37	66	133	181	197	169	192	20	6	1000	294	99
Jammu & Kashmir	51	36	141	156	175	252	138	52	0	1000	744	193
Jharkhand	22	111	91	135	213	231	133	33	30	1000	2664	305
Karnataka	37	100	164	247	189	112	112	36	3	1000	8098	698
Kerala	78	76	169	226	146	119	167	15	5	1000	8672	612
Madhya Pradesh	82	93	127	170	194	113	177	38	6	1000	8580	646
Maharashtra	55	75	134	200	262	108	136	25	5	1000	26513	1268
Manipur	137	64	80	152	158	174	144	91	0	1000	369	231
Meghalaya	25	55	77	308	188	146	183	8	10	1000	90	80
Mizoram	181	170	94	133	80	206	114	12	10	1000	145	246
Nagaland	0	31	152	188	137	192	160	140	0	1000	76	29
Orissa	33	125	147	223	142	80	192	51	6	1000	2729	261
Punjab	39	53	96	177	205	186	158	54	32	1000	4912	329
Rajasthan	28	82	89	172	204	152	222	45	6	1000	6088	537
Sikkim	101	163	214	78	56	210	171	8	0	1000	18	29
Tamil Nadu	71	104	221	211	160	89	117	24	4	1000	16939	1237
Tripura	4	137	161	147	204	177	144	26	0	1000	267	99
Uttaranchal	27	88	115	198	243	155	174	1	0	1000	1252	98
Uttar Pradesh	63	71	117	155	150	193	182	36	32	1000	18694	1241
West Bengal	86	107	248	152	129	116	135	22	5	1000	17040	1028
A & N Islands	61	28	126	257	164	94	248	0	23	1000	33	40

Continued

For peer review only

For peer review only - http://bmjopen.bmj.com/site/about/guidelines.xhtml

Appendix A

Table (10): Per 1000 distribution of aged persons by number of living children for each sex and State/UT

Urban

Persons

state/ut	aged persons with number of living children										no. of aged persons	
	0	1	2	3	4	5	6-7	8-9	10 & above	total	estd. (00)	sample
(1)	(2)	(3)	(4)	(5)	(6)	(7)	(8)	(9)	(10)	(11)	(12)	(13)
Chandigarh	23	32	241	304	230	98	56	16	0	1000	344	137
Dadra & N. Haveli	31	0	119	286	221	269	75	0	0	1000	7	27
Daman & Diu	54	62	204	157	198	63	223	0	39	1000	26	38
Lakshadweep	82	126	40	253	83	203	116	22	74	1000	19	45
Pondicherry	70	29	107	201	233	162	158	41	0	1000	586	103
ONES	86	98	112	165	158	180	144	55	2	1000	983	786
GUTs	53	33	155	238	226	137	127	29	3	1000	1015	390
all-India	58	85	155	187	189	137	146	32	10	1000	161110	12566
estd. no. of aged persons (00)	9315	13748	25005	30196	30422	22126	23578	5109	1612	161110	X	X
sample aged persons	756	1056	1828	2447	2270	1805	1813	457	134	12566	X	X

For peer review only - http://bmjopen.bmj.com/site/about/guidelines.xhtml

For peer review only

Appendix A

Table (10): Per 1000 distribution of aged persons by number of living children for each sex and State/UT

Rural +Urban Males

state/ut	aged persons with number of living children										no. of aged persons	
	0	1	2	3	4	5	6-7	8-9	10 & above	total	estd. (00)	sample
(1)	(2)	(3)	(4)	(5)	(6)	(7)	(8)	(9)	(10)	(11)	(12)	(13)
Andhra Pradesh	52	90	154	222	205	124	119	23	12	1000	24041	1074
Arunachal Pradesh	261	138	116	100	130	122	116	17	2	1000	248	182
Assam	38	24	82	225	260	213	127	25	5	1000	6750	580
Bihar	52	51	120	192	197	196	144	37	11	1000	21316	1081
Chhattisgarh	74	66	80	238	234	129	162	11	7	1000	5250	267
Delhi	30	134	197	181	149	153	155	1	0	1000	2551	227
Goa	76	31	122	273	370	64	45	19	0	1000	735	51
Gujarat	32	51	147	205	210	157	137	51	10	1000	15050	648
Haryana	22	20	70	166	209	193	251	66	4	1000	6923	384
Himachal Pradesh	77	91	145	162	179	196	119	26	6	1000	2539	396
Jammu & Kashmir	39	84	99	216	189	210	131	32	0	1000	2654	332
Jharkhand	69	75	100	198	168	220	142	15	14	1000	7772	496
Karnataka	36	61	182	227	182	124	149	34	5	1000	15667	773
Kerala	31	62	169	182	203	127	177	39	10	1000	15659	795
Madhya Pradesh	73	73	103	177	182	139	200	44	10	1000	18781	868
Maharashtra	32	51	143	219	237	164	109	34	11	1000	36191	1313
Manipur	186	61	66	130	131	193	175	54	5	1000	587	327
Meghalaya	79	75	152	208	106	131	206	33	9	1000	510	130
Mizoram	150	158	69	150	93	189	178	5	7	1000	204	231
Nagaland	81	43	125	146	143	100	255	108	0	1000	178	80
Orissa	58	87	140	209	217	116	127	41	6	1000	14917	673
Punjab	40	48	114	228	182	170	181	22	14	1000	9408	423
Rajasthan	54	86	104	145	166	141	238	62	5	1000	15208	823
Sikkim	40	74	77	124	230	226	153	70	7	1000	126	126
Tamil Nadu	37	93	207	253	173	122	97	16	2	1000	24916	1158
Tripura	28	74	117	225	176	232	114	31	4	1000	1057	216
Uttaranchal	70	56	126	159	215	193	113	68	0	1000	3232	151
Uttar Pradesh	77	74	95	147	173	177	191	46	20	1000	51892	2445
West Bengal	59	71	166	185	158	149	148	47	16	1000	26855	1202
A & N Islands	13	0	100	265	238	182	127	74	0	1000	94	52

Continued

For peer review only - http://bmjopen.bmj.com/site/about/guidelines.xhtml

Appendix A

Table (10): Per 1000 distribution of aged persons by number of living children for each sex and State/UT

Rural +Urban Males

state/ut	aged persons with number of living children										no. of aged persons	
	0	1	2	3	4	5	6-7	8-9	10 & above	total	estd. (00)	sample
(1)	(2)	(3)	(4)	(5)	(6)	(7)	(8)	(9)	(10)	(11)	(12)	(13)
Chandigarh	3	38	253	342	220	86	59	0	0	1000	205	86
Dadra & N. Haveli	0	95	45	405	254	145	56	0	0	1000	82	33
Daman & Diu	74	0	243	72	267	128	146	0	71	1000	14	23
Lakshadweep	74	109	72	242	82	247	64	19	90	1000	15	37
Pondicherry	33	138	92	194	197	135	158	53	0	1000	379	67
ONES	101	81	108	178	145	186	157	39	5	1000	2909	1292
GUTs	21	88	132	262	213	131	116	35	3	1000	789	298
all-India	52	69	134	195	193	156	153	37	11	1000	332005	17750
estd. no. of aged persons (00)	17284	22815	44645	64893	63925	51639	50863	12435	3506	332005	X	X
sample aged persons	988	1223	2295	3401	3363	2865	2699	703	213	17750	X	X

For peer review only - http://bmjopen.bmj.com/site/about/guidelines.xhtml

Appendix A

Table (10): Per 1000 distribution of aged persons by number of living children for each sex and State/UT

Rural +Urban Females

state/ut	aged persons with number of living children										no. of aged persons	
	0	1	2	3	4	5	6-7	8-9	10 & above	total	estd. (00)	sample
(1)	(2)	(3)	(4)	(5)	(6)	(7)	(8)	(9)	(10)	(11)	(12)	(13)
Andhra Pradesh	60	147	165	184	170	124	130	17	3	1000	26195	1109
Arunachal Pradesh	153	131	30	182	192	199	86	28	0	1000	211	148
Assam	61	54	59	189	269	232	114	19	3	1000	4806	408
Bihar	53	76	124	206	162	211	119	37	12	1000	17087	823
Chhattisgarh	116	73	119	143	205	169	147	12	16	1000	7440	378
Delhi	44	67	186	205	221	154	109	5	9	1000	2452	224
Goa	106	107	58	224	342	130	11	22	0	1000	871	72
Gujarat	36	78	120	166	201	168	167	58	5	1000	15501	689
Haryana	14	17	61	123	205	221	269	81	10	1000	7235	375
Himachal Pradesh	84	172	94	129	132	175	178	30	5	1000	2701	396
Jammu & Kashmir	19	104	103	194	131	199	172	73	5	1000	1891	264
Jharkhand	54	83	96	180	192	235	106	46	7	1000	5733	370
Karnataka	51	111	179	188	166	142	129	30	5	1000	14951	756
Kerala	62	75	126	166	183	133	201	40	14	1000	19699	971
Madhya Pradesh	76	110	108	170	165	153	162	48	10	1000	19166	871
Maharashtra	70	85	121	164	220	155	141	35	8	1000	38001	1388
Manipur	170	60	102	116	95	191	180	82	4	1000	456	278
Meghalaya	69	80	116	123	91	194	265	54	6	1000	479	141
Mizoram	178	175	104	124	95	155	150	12	6	1000	137	152
Nagaland	137	0	128	42	0	77	476	140	0	1000	47	29
Orissa	62	149	148	172	194	106	131	28	9	1000	13339	596
Punjab	10	53	115	197	177	210	180	50	8	1000	8865	403
Rajasthan	35	78	87	155	172	159	226	75	14	1000	16075	849
Sikkim	116	78	68	156	189	112	188	66	27	1000	116	107
Tamil Nadu	66	141	193	186	158	110	120	22	3	1000	26130	1199
Tripura	27	130	105	168	194	213	143	21	0	1000	812	189
Uttaranchal	46	105	151	235	225	109	101	29	0	1000	2884	139
Uttar Pradesh	56	85	117	166	169	192	164	34	16	1000	50887	2270
West Bengal	69	75	159	178	146	133	171	50	20	1000	26787	1192
A & N Islands	128	98	26	145	83	251	255	0	13	1000	58	37

Continued

For peer review only - http://bmjopen.bmj.com/site/about/guidelines.xhtml

Appendix A

Table (10): Per 1000 distribution of aged persons by number of living children for each sex and State/UT

Rural +Urban — Females

state/ut	aged persons with number of living children										no. of aged persons	
	0	1	2	3	4	5	6-7	8-9	10 & above	total	estd. (00)	sample
(1)	(2)	(3)	(4)	(5)	(6)	(7)	(8)	(9)	(10)	(11)	(12)	(13)
Chandigarh	50	31	205	229	252	116	81	35	0	1000	155	79
Dadra & N. Haveli	42	0	88	372	395	40	37	26	0	1000	42	24
Daman & Diu	23	99	174	179	46	67	340	72	0	1000	29	35
Lakshadweep	132	93	110	204	92	163	199	7	0	1000	19	46
Pondicherry	71	53	138	202	212	120	180	23	0	1000	516	74
ONES	93	103	98	143	142	192	181	45	4	1000	2257	1044
GUTs	69	52	141	211	211	124	165	25	1	1000	821	295
all-India	59	95	132	173	179	159	155	38	10	1000	331775	17081
estd. no. of aged persons (00)	19483	31463	43696	57530	59500	52618	51543	12631	3310	331775	X	X
sample aged persons	1078	1524	2098	2940	3051	2751	2735	718	186	17081	X	X

For peer review only - http://bmjopen.bmj.com/site/about/guidelines.xhtml

Appendix A

Table (10): Per 1000 distribution of aged persons by number of living children for each sex and State/UT

Rural +Urban — Persons

state/ut	aged persons with number of living children										no. of aged persons	
	0	1	2	3	4	5	6-7	8-9	10 & above	total	estd. (00)	sample
(1)	(2)	(3)	(4)	(5)	(6)	(7)	(8)	(9)	(10)	(11)	(12)	(13)
Andhra Pradesh	56	120	160	202	187	124	125	20	7	1000	50236	2183
Arunachal Pradesh	211	134	76	138	158	157	102	22	1	1000	460	330
Assam	48	36	72	210	264	221	121	22	4	1000	11556	988
Bihar	53	62	122	199	181	203	133	37	11	1000	38403	1904
Chhattisgarh	99	70	103	182	217	152	154	11	12	1000	12690	645
Delhi	37	101	192	193	184	153	133	3	4	1000	5002	451
Goa	92	72	87	246	355	100	26	21	0	1000	1606	123
Gujarat	34	65	133	185	205	163	152	54	7	1000	30551	1337
Haryana	18	19	65	144	207	207	260	74	7	1000	14158	759
Himachal Pradesh	81	133	118	145	155	185	150	28	5	1000	5240	792
Jammu & Kashmir	31	93	101	207	165	206	148	49	2	1000	4545	596
Jharkhand	63	78	99	190	178	226	127	28	11	1000	13505	866
Karnataka	43	85	180	208	174	133	139	32	5	1000	30618	1529
Kerala	48	70	145	173	192	130	191	39	12	1000	35359	1766
Madhya Pradesh	74	92	105	173	173	146	180	46	10	1000	37947	1739
Maharashtra	51	68	132	191	228	159	126	35	9	1000	74193	2701
Manipur	179	60	82	124	115	192	177	66	5	1000	1043	605
Meghalaya	74	78	135	167	99	162	235	43	8	1000	988	271
Mizoram	162	165	83	139	94	175	167	8	7	1000	341	383
Nagaland	93	34	125	125	113	95	301	115	0	1000	224	109
Orissa	60	116	144	191	206	112	129	35	7	1000	28256	1269
Punjab	26	50	114	213	180	190	181	35	11	1000	18274	826
Rajasthan	44	82	95	150	169	150	232	69	9	1000	31284	1672
Sikkim	76	76	72	139	210	171	170	68	16	1000	242	233
Tamil Nadu	52	117	200	219	165	116	109	19	3	1000	51046	2357
Tripura	27	98	111	200	184	224	127	26	2	1000	1869	405
Uttaranchal	58	79	138	195	220	153	107	49	0	1000	6116	290
Uttar Pradesh	67	80	106	156	171	185	178	40	18	1000	102779	4715
West Bengal	64	73	163	181	152	141	159	48	18	1000	53642	2394
A & N Islands	57	37	72	219	179	208	176	46	5	1000	152	89

Continued

For peer review only - http://bmjopen.bmj.com/site/about/guidelines.xhtml

Appendix A

Table (10): Per 1000 distribution of aged persons by number of living children for each sex and State/UT

Rural +Urban — Persons

state/ut	aged persons with number of living children										no. of aged persons	
	0	1	2	3	4	5	6-7	8-9	10 & above	total	estd. (00)	sample
(1)	(2)	(3)	(4)	(5)	(6)	(7)	(8)	(9)	(10)	(11)	(12)	(13)
Chandigarh	23	35	232	293	234	99	68	16	0	1000	360	165
Dadra & N. Haveli	14	63	60	394	302	109	50	9	0	1000	124	57
Daman & Diu	39	67	196	145	118	87	277	49	23	1000	43	58
Lakshadweep	106	100	93	221	88	200	140	12	40	1000	35	83
Pondicherry	55	89	119	199	206	126	171	36	0	1000	895	141
ONES	97	90	104	163	144	188	168	42	4	1000	5167	2336
GUTs	45	70	137	236	212	127	141	30	2	1000	1609	593
all-India	55	82	133	184	186	157	154	38	10	1000	663779	34831
estd. no. of aged persons (00)	36766	54278	88342	122423	123425	104257	102406	25066	6815	663779	X	X
sample aged persons	2066	2747	4393	6341	6414	5616	5434	1421	399	34831	X	X

For peer review only - http://bmjopen.bmj.com/site/about/guidelines.xhtml

Appendix A

Table (11): Per 1000 distribution of aged persons by state of economic independence for each sex

Rural Males

state/ut	state of economic independence of aged persons					no. of aged persons	
	not dependent on others	partially dependent on others	fully dependent on others	n.r.	total	estd. (00)	sample
(1)	(2)	(3)	(4)	(5)	(6)	(7)	(8)
Andhra Pradesh	486	111	394	10	1000	18730	697
Arunachal Pradesh	319	376	60	244	1000	238	140
Assam	443	242	279	37	1000	6103	471
Bihar	582	156	248	15	1000	18861	912
Chhattisgarh	538	101	329	33	1000	4609	204
Delhi	405	255	340	0	1000	338	16
Goa	834	90	76	0	1000	506	19
Gujarat	494	147	354	6	1000	10458	351
Haryana	376	380	243	1	1000	5608	252
Himachal Pradesh	578	181	222	19	1000	2400	347
Jammu & Kashmir	653	119	205	23	1000	2246	230
Jharkhand	552	166	271	10	1000	6400	330
Karnataka	541	137	321	1	1000	11642	428
Kerala	361	204	432	2	1000	11673	505
Madhya Pradesh	576	105	297	23	1000	14666	549
Maharashtra	490	166	341	3	1000	23347	717
Manipur	278	299	231	192	1000	395	211
Meghalaya	575	191	217	18	1000	467	96
Mizoram	566	206	142	86	1000	119	89
Nagaland	486	319	162	33	1000	109	56
Orissa	447	206	324	23	1000	13371	525
Punjab	455	168	363	14	1000	6843	258
Rajasthan	469	147	377	7	1000	12196	552
Sikkim	611	205	179	6	1000	115	110
Tamil Nadu	487	159	355	0	1000	16961	571
Tripura	457	170	353	21	1000	927	171
Uttaranchal	615	49	277	59	1000	2674	102
Uttar Pradesh	598	100	279	22	1000	42788	1825
West Bengal	462	182	331	25	1000	18130	680
A & N Islands	844	34	122	0	1000	76	34
Chandigarh	980	20	0	0	1000	7	17
Dadra & N. Haveli	166	537	297	0	1000	78	20
Daman & Diu	744	256	0	0	1000	4	7
Lakshadweep	301	136	564	0	1000	7	16
Pondicherry	697	49	254	0	1000	160	20
ONES	451	226	248	74	1000	2371	873
GUTs	605	164	232	0	1000	333	114
all-India	513	152	320	15	1000	253254	11528
estd. no. of aged persons (00)	129840	38598	81116	3700	253254	X	X
sample aged persons	5855	1834	3638	201	11528	X	X

For peer review only - http://bmjopen.bmj.com/site/about/guidelines.xhtml

Appendix A

Table (11): Per 1000 distribution of aged persons by state of economic independence for each sex

Rural Females

state/ut	state of economic independence of aged persons					no. of aged persons	
	not dependent on others	partially dependent on others	fully dependent on others	n.r.	total	estd. (00)	sample
(1)	(2)	(3)	(4)	(5)	(6)	(7)	(8)
Andhra Pradesh	148	112	729	10	1000	20035	688
Arunachal Pradesh	108	401	347	145	1000	204	118
Assam	81	52	814	53	1000	4273	323
Bihar	167	117	696	19	1000	15258	678
Chhattisgarh	262	107	608	24	1000	6680	290
Delhi	0	287	713	0	1000	322	17
Goa	260	123	618	0	1000	615	32
Gujarat	129	98	772	1	1000	10401	370
Haryana	127	430	444	0	1000	5510	232
Himachal Pradesh	183	150	635	32	1000	2546	346
Jammu & Kashmir	107	126	760	7	1000	1555	173
Jharkhand	166	107	706	20	1000	4441	231
Karnataka	148	112	731	9	1000	10878	403
Kerala	102	183	700	15	1000	15013	649
Madhya Pradesh	152	122	698	28	1000	14701	544
Maharashtra	183	129	682	7	1000	24333	716
Manipur	89	294	485	132	1000	279	163
Meghalaya	177	230	544	49	1000	431	95
Mizoram	124	196	531	149	1000	76	48
Nagaland	230	444	326	0	1000	39	24
Orissa	75	124	774	27	1000	12157	483
Punjab	100	189	709	2	1000	6518	239
Rajasthan	95	127	778	0	1000	12999	583
Sikkim	111	194	618	76	1000	109	94
Tamil Nadu	193	165	642	0	1000	17146	549
Tripura	92	77	815	16	1000	675	135
Uttaranchal	350	47	593	10	1000	2190	90
Uttar Pradesh	127	79	770	24	1000	41298	1649
West Bengal	64	82	820	34	1000	18472	686
A & N Islands	463	98	283	156	1000	43	15
Chandigarh	253	147	600	0	1000	8	11
Dadra & N. Haveli	122	327	551	0	1000	39	10
Daman & Diu	254	0	746	0	1000	13	13
Lakshadweep	184	0	816	0	1000	9	22
Pondicherry	439	321	240	0	1000	148	18
ONES	119	203	613	65	1000	1813	677
GUTs	372	252	350	26	1000	261	89
all-India	139	124	720	16	1000	249416	10737
estd. no. of aged persons (00)	34658	30995	179695	4068	249416	X	X
sample aged persons	1411	1400	7710	216	10737	X	X

For peer review only - http://bmjopen.bmj.com/site/about/guidelines.xhtml

Appendix A

Table (11): Per 1000 distribution of aged persons by state of economic independence for each sex

Rural Persons

state/ut	state of economic independence of aged persons					no. of aged persons	
	not dependent on others	partially dependent on others	fully dependent on others	n.r.	total	estd. (00)	sample
(1)	(2)	(3)	(4)	(5)	(6)	(7)	(8)
Andhra Pradesh	312	112	567	10	1000	38765	1385
Arunachal Pradesh	222	388	192	198	1000	442	258
Assam	294	163	499	43	1000	10376	794
Bihar	396	138	448	17	1000	34119	1590
Chhattisgarh	374	104	494	27	1000	11289	494
Delhi	207	271	522	0	1000	661	33
Goa	519	108	373	0	1000	1121	51
Gujarat	312	122	563	3	1000	20859	721
Haryana	252	404	343	1	1000	11118	484
Himachal Pradesh	375	165	434	26	1000	4946	693
Jammu & Kashmir	430	122	432	16	1000	3801	403
Jharkhand	394	142	449	14	1000	10841	561
Karnataka	352	125	519	5	1000	22520	831
Kerala	216	192	582	9	1000	26687	1154
Madhya Pradesh	364	113	498	25	1000	29367	1093
Maharashtra	333	147	515	5	1000	47679	1433
Manipur	200	297	336	167	1000	673	374
Meghalaya	384	210	373	33	1000	898	191
Mizoram	394	202	294	110	1000	196	137
Nagaland	419	352	205	24	1000	148	80
Orissa	270	167	538	25	1000	25527	1008
Punjab	282	178	532	8	1000	13361	497
Rajasthan	276	136	584	3	1000	25196	1135
Sikkim	368	199	392	40	1000	224	204
Tamil Nadu	339	162	499	0	1000	34107	1120
Tripura	303	131	547	19	1000	1602	306
Uttaranchal	496	48	419	37	1000	4865	192
Uttar Pradesh	367	90	520	23	1000	84086	3474
West Bengal	261	131	578	29	1000	36602	1366
A & N Islands	706	57	180	56	1000	120	49
Chandigarh	591	88	321	0	1000	16	28
Dadra & N. Haveli	151	468	381	0	1000	117	30
Daman & Diu	368	60	572	0	1000	17	20
Lakshadweep	236	60	704	0	1000	16	38
Pondicherry	573	179	247	0	1000	309	38
ONES	307	216	406	70	1000	4184	1550
GUTs	502	202	284	11	1000	594	203
all-India	327	138	519	15	1000	502670	22265
estd. no. of aged persons (00)	164498	69592	260811	7768	502670	X	X
sample aged persons	7266	3234	11348	417	22265	X	X

For peer review only - http://bmjopen.bmj.com/site/about/guidelines.xhtml

Appendix A

Table (11): Per 1000 distribution of aged persons by state of economic independence for each sex

Urban Males

state/ut	state of economic independence of aged persons					no. of aged persons	
	not dependent on others	partially dependent on others	fully dependent on others	n.r.	total	estd. (00)	sample
(1)	(2)	(3)	(4)	(5)	(6)	(7)	(8)
Andhra Pradesh	567	104	327	3	1000	5310	377
Arunachal Pradesh	313	318	274	95	1000	10	42
Assam	563	151	286	0	1000	647	109
Bihar	441	123	379	57	1000	2455	169
Chhattisgarh	587	169	243	0	1000	641	63
Delhi	613	78	295	14	1000	2212	211
Goa	376	350	274	0	1000	229	32
Gujarat	525	114	362	0	1000	4592	297
Haryana	487	202	305	6	1000	1315	132
Himachal Pradesh	723	77	200	0	1000	139	49
Jammu & Kashmir	624	84	285	7	1000	408	102
Jharkhand	498	218	279	4	1000	1372	166
Karnataka	545	97	349	9	1000	4025	345
Kerala	468	183	345	4	1000	3986	290
Madhya Pradesh	637	70	276	17	1000	4115	319
Maharashtra	497	202	293	8	1000	12844	596
Manipur	380	348	168	105	1000	192	116
Meghalaya	909	46	45	0	1000	42	34
Mizoram	508	217	142	133	1000	85	142
Nagaland	730	256	14	0	1000	68	24
Orissa	502	153	333	12	1000	1546	148
Punjab	496	142	337	25	1000	2565	165
Rajasthan	552	136	310	2	1000	3012	271
Sikkim	761	91	149	0	1000	11	16
Tamil Nadu	543	139	318	0	1000	7955	587
Tripura	526	179	295	0	1000	130	45
Uttaranchal	827	59	114	0	1000	558	49
Uttar Pradesh	602	96	290	13	1000	9104	620
West Bengal	658	101	227	14	1000	8725	522
A & N Islands	857	0	114	29	1000	18	18
Chandigarh	883	79	38	0	1000	197	69
Dadra & N. Haveli	345	231	424	0	1000	4	13
Daman & Diu	270	358	372	0	1000	10	16
Lakshadweep	91	634	275	0	1000	8	21
Pondicherry	455	134	411	0	1000	218	47
ONES	528	245	167	60	1000	538	419
GUTs	645	119	235	1	1000	455	184
all-India	555	134	301	10	1000	78751	6222
estd. no. of aged persons (00)	43733	10529	23710	779	78751	X	X
sample aged persons	3512	833	1804	73	6222	X	X

For peer review only - http://bmjopen.bmj.com/site/about/guidelines.xhtml

Appendix A

Table (11): Per 1000 distribution of aged persons by state of economic independence for each sex

Urban Females

state/ut	state of economic independence of aged persons					no. of aged persons	
	not dependent on others	partially dependent on others	fully dependent on others	n.r.	total	estd. (00)	sample
(1)	(2)	(3)	(4)	(5)	(6)	(7)	(8)
Andhra Pradesh	246	92	646	15	1000	6160	421
Arunachal Pradesh	141	145	657	57	1000	7	30
Assam	228	29	673	70	1000	532	85
Bihar	157	70	731	42	1000	1829	145
Chhattisgarh	233	104	663	0	1000	760	88
Delhi	173	84	731	11	1000	2129	207
Goa	36	350	614	0	1000	256	40
Gujarat	117	91	784	9	1000	5101	319
Haryana	174	292	502	32	1000	1725	143
Himachal Pradesh	302	144	545	10	1000	155	50
Jammu & Kashmir	105	51	832	12	1000	336	91
Jharkhand	124	65	783	28	1000	1292	139
Karnataka	138	71	786	5	1000	4073	353
Kerala	189	155	640	17	1000	4686	322
Madhya Pradesh	180	117	669	33	1000	4466	327
Maharashtra	186	66	742	6	1000	13669	672
Manipur	82	161	623	134	1000	178	115
Meghalaya	234	123	634	9	1000	48	46
Mizoram	185	165	577	73	1000	60	104
Nagaland	409	112	479	0	1000	8	5
Orissa	62	98	800	40	1000	1182	113
Punjab	121	63	805	10	1000	2347	164
Rajasthan	129	82	789	0	1000	3076	266
Sikkim	359	262	379	0	1000	7	13
Tamil Nadu	193	119	688	0	1000	8984	650
Tripura	243	69	688	0	1000	136	54
Uttaranchal	187	70	711	32	1000	693	49
Uttar Pradesh	136	82	765	17	1000	9590	621
West Bengal	183	84	723	10	1000	8315	506
A & N Islands	185	49	766	0	1000	15	22
Chandigarh	403	24	563	10	1000	147	68
Dadra & N. Haveli	0	134	866	0	1000	3	14
Daman & Diu	56	27	875	42	1000	16	22
Lakshadweep	72	389	539	0	1000	10	24
Pondicherry	103	173	724	0	1000	368	56
ONES	173	130	632	65	1000	444	367
GUTs	181	130	685	4	1000	560	206
all-India	170	95	721	13	1000	82359	6344
estd. no. of aged persons (00)	14033	7855	59395	1076	82359	X	X
sample aged persons	1022	619	4591	112	6344	X	X

For peer review only - http://bmjopen.bmj.com/site/about/guidelines.xhtml

Appendix A

Table (11): Per 1000 distribution of aged persons by state of economic independence for each sex

Urban Persons

state/ut	state of economic independence of aged persons					no. of aged persons	
	not dependent on others	partially dependent on others	fully dependent on others	n.r.	total	estd. (00)	sample
(1)	(2)	(3)	(4)	(5)	(6)	(7)	(8)
Andhra Pradesh	395	98	498	10	1000	11470	798
Arunachal Pradesh	242	247	432	79	1000	17	72
Assam	412	96	461	31	1000	1180	194
Bihar	319	101	529	51	1000	4283	314
Chhattisgarh	395	134	471	0	1000	1401	151
Delhi	397	81	509	12	1000	4342	418
Goa	197	350	454	0	1000	485	72
Gujarat	310	102	584	5	1000	9693	616
Haryana	309	253	417	21	1000	3040	275
Himachal Pradesh	501	112	382	5	1000	294	99
Jammu & Kashmir	390	69	532	9	1000	744	193
Jharkhand	317	144	524	16	1000	2664	305
Karnataka	340	84	569	7	1000	8098	698
Kerala	317	168	504	11	1000	8672	612
Madhya Pradesh	399	95	481	25	1000	8580	646
Maharashtra	337	132	524	7	1000	26513	1268
Manipur	237	258	387	119	1000	369	231
Meghalaya	552	86	357	5	1000	90	80
Mizoram	373	195	323	108	1000	145	246
Nagaland	697	242	61	0	1000	76	29
Orissa	311	129	535	24	1000	2729	261
Punjab	317	105	561	18	1000	4912	329
Rajasthan	338	109	552	1	1000	6088	537
Sikkim	600	159	241	0	1000	18	29
Tamil Nadu	357	128	514	0	1000	16939	1237
Tripura	381	123	496	0	1000	267	99
Uttaranchal	472	65	445	18	1000	1252	98
Uttar Pradesh	363	89	533	15	1000	18694	1241
West Bengal	427	93	469	12	1000	17040	1028
A & N Islands	550	23	411	16	1000	33	40
Chandigarh	678	55	262	4	1000	344	137
Dadra & N. Haveli	182	186	632	0	1000	7	27
Daman & Diu	138	154	682	26	1000	26	38
Lakshadweep	80	497	423	0	1000	19	45
Pondicherry	234	158	608	0	1000	586	103
ONES	367	193	377	62	1000	983	786
GUTs	389	125	483	3	1000	1015	390
all-India	359	114	516	12	1000	161110	12566
estd. no. of aged persons (00)	57766	18384	83105	1855	161110	X	X
sample aged persons	4534	1452	6395	185	12566	X	X

For peer review only - http://bmjopen.bmj.com/site/about/guidelines.xhtml

Appendix A

Table (11): Per 1000 distribution of aged persons by state of economic independence for each sex

Rural + Urban — Males

state/ut	state of economic independence of aged persons					no. of aged persons	
	not dependent on others	partially dependent on others	fully dependent on others	n.r.	total	estd. (00)	sample
(1)	(2)	(3)	(4)	(5)	(6)	(7)	(8)
Andhra Pradesh	504	109	379	8	1000	24041	1074
Arunachal Pradesh	319	374	69	238	1000	248	182
Assam	455	233	279	33	1000	6750	580
Bihar	566	152	263	20	1000	21316	1081
Chhattisgarh	544	109	318	29	1000	5250	267
Delhi	585	102	301	12	1000	2551	227
Goa	691	171	138	0	1000	735	51
Gujarat	503	137	356	4	1000	15050	648
Haryana	397	346	255	2	1000	6923	384
Himachal Pradesh	586	175	221	18	1000	2539	396
Jammu & Kashmir	649	113	217	20	1000	2654	332
Jharkhand	543	176	272	9	1000	7772	496
Karnataka	542	127	328	3	1000	15667	773
Kerala	389	199	410	3	1000	15659	795
Madhya Pradesh	589	97	292	22	1000	18781	868
Maharashtra	492	179	324	5	1000	36191	1313
Manipur	311	315	211	163	1000	587	327
Meghalaya	603	179	202	16	1000	510	130
Mizoram	542	210	142	105	1000	204	231
Nagaland	580	295	105	20	1000	178	80
Orissa	452	201	325	22	1000	14917	673
Punjab	466	161	356	17	1000	9408	423
Rajasthan	486	145	364	6	1000	15208	823
Sikkim	623	195	176	5	1000	126	126
Tamil Nadu	505	152	343	0	1000	24916	1158
Tripura	466	171	345	18	1000	1057	216
Uttaranchal	652	51	249	49	1000	3232	151
Uttar Pradesh	599	100	281	20	1000	51892	2445
West Bengal	526	155	298	21	1000	26855	1202
A & N Islands	847	27	120	6	1000	94	52
Chandigarh	886	77	37	0	1000	205	86
Dadra & N. Haveli	174	523	303	0	1000	82	33
Daman & Diu	409	328	263	0	1000	14	23
Lakshadweep	188	403	409	0	1000	15	37
Pondicherry	558	98	345	0	1000	379	67
ONES	465	230	233	72	1000	2909	1292
GUTs	628	138	233	1	1000	789	298
all-India	523	148	316	13	1000	332005	17750
estd. no. of aged persons (00)	173573	49127	104826	4479	332005	X	X
sample aged persons	9367	2667	5442	274	17750	X	X

For peer review only - http://bmjopen.bmj.com/site/about/guidelines.xhtml

Appendix A

Table (11): Per 1000 distribution of aged persons by state of economic independence for each sex

Rural + Urban | Females

state/ut	state of economic independence of aged persons					no. of aged persons	
	not dependent on others	partially dependent on others	fully dependent on others	n.r.	total	estd. (00)	sample
(1)	(2)	(3)	(4)	(5)	(6)	(7)	(8)
Andhra Pradesh	171	108	710	11	1000	26195	1109
Arunachal Pradesh	109	392	357	142	1000	211	148
Assam	98	49	799	55	1000	4806	408
Bihar	166	112	700	22	1000	17087	823
Chhattisgarh	259	106	613	21	1000	7440	378
Delhi	151	111	729	10	1000	2452	224
Goa	194	189	617	0	1000	871	72
Gujarat	125	96	776	3	1000	15501	689
Haryana	138	397	458	8	1000	7235	375
Himachal Pradesh	190	150	629	30	1000	2701	396
Jammu & Kashmir	107	113	773	8	1000	1891	264
Jharkhand	157	98	723	22	1000	5733	370
Karnataka	146	101	746	8	1000	14951	756
Kerala	123	177	685	15	1000	19699	971
Madhya Pradesh	159	121	691	29	1000	19166	871
Maharashtra	184	106	703	7	1000	38001	1388
Manipur	86	242	539	133	1000	456	278
Meghalaya	182	220	553	45	1000	479	141
Mizoram	151	182	551	115	1000	137	152
Nagaland	260	389	352	0	1000	47	29
Orissa	74	122	776	28	1000	13339	596
Punjab	106	156	734	4	1000	8865	403
Rajasthan	101	118	781	0	1000	16075	849
Sikkim	127	198	604	72	1000	116	107
Tamil Nadu	193	149	658	0	1000	26130	1199
Tripura	118	76	794	13	1000	812	189
Uttaranchal	311	52	621	15	1000	2884	139
Uttar Pradesh	129	79	769	23	1000	50887	2270
West Bengal	101	83	790	27	1000	26787	1192
A & N Islands	391	86	407	116	1000	58	37
Chandigarh	395	31	565	10	1000	155	79
Dadra & N. Haveli	112	312	576	0	1000	42	24
Daman & Diu	147	15	816	23	1000	29	35
Lakshadweep	124	209	667	0	1000	19	46
Pondicherry	200	215	585	0	1000	516	74
ONES	130	189	617	65	1000	2257	1044
GUTs	242	169	578	11	1000	821	295
all-India	147	117	721	16	1000	331775	17081
estd. no. of aged persons (00)	48692	38850	239090	5144	331775	X	X
sample aged persons	2433	2019	12301	328	17081	X	X

For peer review only - http://bmjopen.bmj.com/site/about/guidelines.xhtml

Appendix A

Table (11): Per 1000 distribution of aged persons by state of economic independence for each sex

Rural + Urban Persons

state/ut	state of economic independence of aged persons					no. of aged persons	
	not dependent on others	partially dependent on others	fully dependent on others	n.r.	total	estd. (00)	sample
(1)	(2)	(3)	(4)	(5)	(6)	(7)	(8)
Andhra Pradesh	331	108	551	10	1000	50236	2183
Arunachal Pradesh	222	382	202	194	1000	460	330
Assam	306	157	495	42	1000	11556	988
Bihar	388	134	457	21	1000	38403	1904
Chhattisgarh	377	108	491	24	1000	12690	645
Delhi	372	106	511	11	1000	5002	451
Goa	421	181	397	0	1000	1606	123
Gujarat	311	116	569	4	1000	30551	1337
Haryana	264	372	359	5	1000	14158	759
Himachal Pradesh	382	162	431	24	1000	5240	792
Jammu & Kashmir	423	113	448	15	1000	4545	596
Jharkhand	379	143	464	15	1000	13505	866
Karnataka	349	114	532	5	1000	30618	1529
Kerala	241	186	563	10	1000	35359	1766
Madhya Pradesh	372	109	494	25	1000	37947	1739
Maharashtra	334	142	518	6	1000	74193	2701
Manipur	213	283	354	150	1000	1043	605
Meghalaya	399	199	372	30	1000	988	271
Mizoram	385	199	306	109	1000	341	383
Nagaland	513	314	156	16	1000	224	109
Orissa	274	163	538	25	1000	28256	1269
Punjab	291	158	539	11	1000	18274	826
Rajasthan	288	131	578	3	1000	31284	1672
Sikkim	385	197	381	37	1000	242	233
Tamil Nadu	345	151	504	0	1000	51046	2357
Tripura	314	129	540	16	1000	1869	405
Uttaranchal	491	52	424	33	1000	6116	290
Uttar Pradesh	366	90	523	21	1000	102779	4715
West Bengal	314	119	543	24	1000	53642	2394
A & N Islands	673	50	230	48	1000	152	89
Chandigarh	674	57	265	4	1000	360	165
Dadra & N. Haveli	153	451	396	0	1000	124	57
Daman & Diu	232	116	637	15	1000	43	58
Lakshadweep	152	295	553	0	1000	35	83
Pondicherry	351	166	483	0	1000	895	141
ONES	319	212	401	69	1000	5167	2336
GUTs	431	154	409	6	1000	1609	593
all-India	335	133	518	14	1000	663779	34831
estd. no. of aged persons (00)	222264	87976	343916	9623	663779	X	X
sample aged persons	11800	4686	17743	602	34831	X	X

For peer review only - http://bmjopen.bmj.com/site/about/guidelines.xhtml

Appendix A

Table (12): Per 1000 distribution of fully economically independent aged persons by number of dependants for each sex and State/UT

Rural | Males

state/ut	number of dependants									no. of eco. independent aged persons	
	0	1	2	3	4	5	6-8	9 & above	total	estd. (00)	sample
(1)	(2)	(3)	(4)	(5)	(6)	(7)	(8)	(9)	(10)	(11)	(12)
Andhra Pradesh	95	413	242	71	86	39	36	18	1000	9103	307
Arunachal Pradesh	0	91	352	200	102	121	93	41	1000	76	51
Assam	27	230	165	122	138	116	156	46	1000	2704	212
Bihar	42	225	262	113	101	57	145	55	1000	10974	539
Chhattisgarh	106	218	248	88	107	91	100	41	1000	2478	110
Delhi	321	332	347	0	0	0	0	0	1000	137	6
Goa	289	550	102	58	0	0	0	0	1000	422	13
Gujarat	219	373	161	98	43	22	70	15	1000	5162	177
Haryana	59	282	268	52	133	54	74	78	1000	2106	92
Himachal Pradesh	74	368	269	74	61	27	118	9	1000	1388	184
Jammu & Kashmir	98	318	284	63	121	63	54	0	1000	1468	146
Jharkhand	33	311	228	62	71	98	155	41	1000	3533	200
Karnataka	95	346	157	131	111	66	65	30	1000	6302	234
Kerala	22	450	329	81	45	32	33	7	1000	4220	190
Madhya Pradesh	113	357	139	64	75	79	111	63	1000	8442	313
Maharashtra	87	383	301	87	29	62	44	6	1000	11432	339
Manipur	0	176	315	81	163	141	107	18	1000	110	57
Meghalaya	17	133	424	105	136	13	171	0	1000	269	48
Mizoram	0	284	156	187	67	47	229	29	1000	68	54
Nagaland	0	162	392	167	223	0	56	0	1000	53	28
Orissa	53	340	260	101	80	72	88	7	1000	5973	224
Punjab	30	285	396	64	93	52	65	16	1000	3113	119
Rajasthan	80	395	129	89	95	43	73	95	1000	5726	259
Sikkim	33	104	326	242	75	83	131	5	1000	70	61
Tamil Nadu	72	420	245	125	54	46	35	4	1000	8253	263
Tripura	20	173	268	198	204	59	46	31	1000	424	77
Uttaranchal	68	215	142	109	145	224	91	5	1000	1645	73
Uttar Pradesh	34	261	239	92	87	87	121	79	1000	25604	1108
West Bengal	39	236	225	149	142	55	115	38	1000	8385	302

Continued

For peer review only - http://bmjopen.bmj.com/site/about/guidelines.xhtml

Appendix A

Table (12): Per 1000 distribution of fully economically independent aged persons by number of dependants for each sex and State/UT

Rural — Males

state/ut	number of dependants									no. of eco. independent aged persons	
	0	1	2	3	4	5	6-8	9 & above	total	estd. (00)	sample
(1)	(2)	(3)	(4)	(5)	(6)	(7)	(8)	(9)	(10)	(11)	(12)
A & N Islands	0	211	383	152	244	10	0	0	1000	64	28
Chandigarh	147	727	43	50	34	0	0	0	1000	7	16
Dadra & N. Haveli	0	232	768	0	0	0	0	0	1000	13	3
Daman & Diu	99	154	0	747	0	0	0	0	1000	3	4
Lakshadweep	27	80	0	0	216	69	608	0	1000	2	6
Pondicherry	173	462	0	154	0	0	89	122	1000	112	12
ONES	14	159	321	164	159	59	105	19	1000	1069	376
GUTs	103	368	173	147	81	4	56	68	1000	202	69
all-India	69	321	234	97	85	65	90	40	1000	129840	5855
estd. no. of eco. indep. aged persons (00)	8901	41652	30363	12533	11007	8481	11652	5251	129840	X	X
sample eco. indep. aged persons	361	1815	1379	587	503	393	540	277	5855	X	X

For peer review only - http://bmjopen.bmj.com/site/about/guidelines.xhtml

Appendix A

Table (12): Per 1000 distribution of fully economically independent aged persons by number of dependants for each sex and State/UT

Rural | Females

state/ut	number of dependants									no. of eco. independent aged persons	
	0	1	2	3	4	5	6-8	9 & above	total	estd. (00)	sample
(1)	(2)	(3)	(4)	(5)	(6)	(7)	(8)	(9)	(10)	(11)	(12)
Andhra Pradesh	365	211	366	1	36	21	0	0	1000	2974	89
Arunachal Pradesh	0	295	171	394	0	134	0	6	1000	22	11
Assam	227	255	402	117	0	0	0	0	1000	348	24
Bihar	199	110	478	93	8	2	96	14	1000	2553	123
Chhattisgarh	160	209	417	29	22	39	99	26	1000	1748	75
Delhi	0	0	0	0	0	0	0	0	0	0	0
Goa	482	35	483	0	0	0	0	0	1000	160	8
Gujarat	668	241	79	0	0	0	0	12	1000	1341	50
Haryana	438	32	450	0	59	0	0	21	1000	698	29
Himachal Pradesh	364	39	538	34	19	0	6	0	1000	466	63
Jammu & Kashmir	670	43	127	0	142	18	0	0	1000	167	18
Jharkhand	220	129	274	13	67	20	0	278	1000	739	29
Karnataka	579	109	190	49	28	34	10	0	1000	1614	55
Kerala	298	135	501	59	7	0	0	0	1000	1538	62
Madhya Pradesh	484	173	255	27	40	0	21	0	1000	2239	79
Maharashtra	316	156	419	73	13	2	20	0	1000	4456	112
Manipur	59	231	227	54	238	191	0	0	1000	25	14
Meghalaya	49	178	573	78	0	0	122	0	1000	76	23
Mizoram	0	450	144	165	0	0	241	0	1000	9	6
Nagaland	0	0	978	0	22	0	0	0	1000	9	5
Orissa	113	296	506	13	0	71	0	0	1000	909	36
Punjab	199	117	680	0	0	0	4	0	1000	654	23
Rajasthan	396	211	159	133	80	14	8	0	1000	1228	48
Sikkim	0	58	677	88	0	0	177	0	1000	12	10
Tamil Nadu	460	285	212	21	0	19	3	0	1000	3310	88
Tripura	300	335	366	0	0	0	0	0	1000	62	10
Uttaranchal	341	117	381	114	35	2	12	0	1000	768	37
Uttar Pradesh	134	137	461	80	76	26	42	45	1000	5257	213
West Bengal	237	261	446	46	0	10	0	0	1000	1180	48

Continued

For peer review only - http://bmjopen.bmj.com/site/about/guidelines.xhtml

Appendix A

Table (12): Per 1000 distribution of fully economically independent aged persons by number of dependants for each sex and State/UT

Rural | Females

state/ut	number of dependants									no. of eco. independent aged persons	
	0	1	2	3	4	5	6-8	9 & above	total	estd. (00)	sample
(1)	(2)	(3)	(4)	(5)	(6)	(7)	(8)	(9)	(10)	(11)	(12)
A & N Islands	0	230	635	0	136	0	0	0	1000	20	6
Chandigarh	113	887	0	0	0	0	0	0	1000	2	2
Dadra & N. Haveli	331	669	0	0	0	0	0	0	1000	5	2
Daman & Diu	1000	0	0	0	0	0	0	0	1000	3	3
Lakshadweep	339	0	268	0	0	0	0	393	1000	2	4
Pondicherry	504	496	0	0	0	0	0	0	1000	65	6
ONES	110	239	436	86	28	36	64	1	1000	216	79
GUTs	398	432	135	0	28	0	0	7	1000	97	23
all-India	320	175	370	50	30	15	24	16	1000	34658	1411
estd. no. of eco. indep. aged persons (00)	11105	6061	12816	1738	1026	518	841	553	34658	X	X
sample eco. indep. aged persons	416	245	547	69	49	25	34	26	1411	X	X

For peer review only - http://bmjopen.bmj.com/site/about/guidelines.xhtml

Appendix A

Table (12): Per 1000 distribution of fully economically independent aged persons by number of dependants for each sex and State/UT

Rural Persons

state/ut	number of dependants									no. of eco. independent aged persons	
	0	1	2	3	4	5	6-8	9 & above	total	estd. (00)	sample
(1)	(2)	(3)	(4)	(5)	(6)	(7)	(8)	(9)	(10)	(12)	(13)
Andhra Pradesh	162	363	272	54	74	34	27	14	1000	12077	396
Arunachal Pradesh	0	137	311	244	79	124	72	33	1000	98	62
Assam	49	232	192	122	123	103	138	41	1000	3053	236
Bihar	71	203	303	109	84	47	136	47	1000	13526	662
Chhattisgarh	128	214	318	64	72	69	99	35	1000	4226	185
Delhi	321	332	347	0	0	0	0	0	1000	137	6
Goa	342	409	207	42	0	0	0	0	1000	582	21
Gujarat	311	345	144	78	34	17	56	14	1000	6503	227
Haryana	153	220	313	39	115	40	55	64	1000	2804	121
Himachal Pradesh	147	285	337	64	51	20	90	6	1000	1854	247
Jammu & Kashmir	157	290	268	56	123	58	48	0	1000	1634	164
Jharkhand	66	280	236	53	70	85	128	82	1000	4273	229
Karnataka	194	298	164	114	94	59	54	24	1000	7916	289
Kerala	96	366	375	75	35	24	24	5	1000	5757	252
Madhya Pradesh	190	318	163	56	68	62	92	50	1000	10680	392
Maharashtra	151	319	334	83	25	45	38	5	1000	15888	451
Manipur	11	186	298	76	177	150	87	14	1000	135	71
Meghalaya	24	143	457	99	106	10	160	0	1000	345	71
Mizoram	0	304	155	184	59	41	231	26	1000	77	60
Nagaland	0	138	476	143	194	0	48	0	1000	62	33
Orissa	61	334	292	89	69	72	76	6	1000	6882	260
Punjab	59	256	445	53	77	43	54	13	1000	3767	142
Rajasthan	136	362	134	97	92	38	62	79	1000	6954	307
Sikkim	28	97	378	219	64	71	138	5	1000	83	71
Tamil Nadu	183	382	236	95	38	38	26	3	1000	11562	351
Tripura	56	194	281	173	177	52	40	27	1000	486	87
Uttaranchal	155	184	218	111	110	153	66	4	1000	2413	110
Uttar Pradesh	51	240	277	90	85	77	107	73	1000	30861	1321
West Bengal	63	239	252	137	125	50	101	33	1000	9565	350

Continued

For peer review only

For peer review only - http://bmjopen.bmj.com/site/about/guidelines.xhtml

Appendix A

Table (12): Per 1000 distribution of fully economically independent aged persons by number of dependants for each sex and State/UT

Rural Persons

state/ut	number of dependants									no. of eco. independent aged persons	
	0	1	2	3	4	5	6-8	9 & above	total	estd. (00)	sample
(1)	(2)	(3)	(4)	(5)	(6)	(7)	(8)	(9)	(10)	(12)	(13)
A & N Islands	0	216	442	116	218	8	0	0	1000	84	34
Chandigarh	139	763	33	38	26	0	0	0	1000	9	18
Dadra & N. Haveli	89	350	562	0	0	0	0	0	1000	18	5
Daman & Diu	576	72	0	352	0	0	0	0	1000	6	7
Lakshadweep	163	45	117	0	122	39	343	171	1000	4	10
Pondicherry	295	475	0	97	0	0	56	77	1000	177	18
ONES	31	173	340	151	137	55	98	16	1000	1285	455
GUTs	199	389	161	99	64	3	38	48	1000	299	92
all-India	122	290	262	87	73	55	76	35	1000	164498	7266
estd. no. of eco. indep. aged persons (00)	20006	47714	43179	14270	12033	8999	12493	5804	164498	X	X
sample eco. indep. aged persons	777	2060	1926	656	552	418	574	303	7266	X	X

For peer review only - http://bmjopen.bmj.com/site/about/guidelines.xhtml

Appendix A

Table (12): Per 1000 distribution of fully economically independent aged persons by number of dependants for each sex and State/UT

Urban — Males

state/ut	number of dependants									no. of eco. independent aged persons	
	0	1	2	3	4	5	6-8	9 & above	total	estd. (00)	sample
(1)	(2)	(3)	(4)	(5)	(6)	(7)	(8)	(9)	(10)	(11)	(12)
Andhra Pradesh	66	504	203	59	80	38	49	1	1000	3011	212
Arunachal Pradesh	0	386	478	0	51	65	0	20	1000	3	12
Assam	0	231	251	338	120	54	7	0	1000	364	65
Bihar	68	214	201	83	92	58	155	130	1000	1081	92
Chhattisgarh	156	120	376	171	68	82	27	0	1000	377	36
Delhi	70	543	292	24	62	3	4	2	1000	1356	128
Goa	0	323	68	391	170	48	0	0	1000	86	14
Gujarat	86	427	287	49	78	25	31	17	1000	2409	161
Haryana	43	510	282	8	58	63	13	24	1000	640	61
Himachal Pradesh	97	441	335	115	12	0	0	0	1000	101	34
Jammu & Kashmir	119	291	256	120	101	102	0	11	1000	255	70
Jharkhand	8	197	372	107	76	60	113	67	1000	684	92
Karnataka	87	374	222	150	48	53	38	28	1000	2194	196
Kerala	78	451	287	81	72	14	17	0	1000	1867	146
Madhya Pradesh	93	503	150	52	76	61	33	32	1000	2621	202
Maharashtra	95	494	265	92	19	13	22	0	1000	6389	333
Manipur	19	147	179	111	184	236	90	34	1000	73	41
Meghalaya	0	550	293	32	74	51	0	0	1000	38	28
Mizoram	0	237	271	141	95	31	214	10	1000	43	73
Nagaland	0	134	127	207	88	179	264	0	1000	50	15
Orissa	3	365	204	108	133	154	27	6	1000	776	72
Punjab	167	389	234	152	26	27	6	0	1000	1272	79
Rajasthan	177	350	174	55	96	61	86	1	1000	1663	148
Sikkim	0	268	272	272	0	187	0	0	1000	8	11
Tamil Nadu	137	374	269	108	46	38	24	4	1000	4319	301
Tripura	98	434	152	85	143	64	25	0	1000	69	28
Uttaranchal	32	490	271	0	11	26	84	86	1000	462	38
Uttar Pradesh	13	346	270	93	99	64	86	29	1000	5483	378
West Bengal	89	287	298	132	105	40	43	6	1000	5745	340

Continued

For peer review only - http://bmjopen.bmj.com/site/about/guidelines.xhtml

Appendix A

Table (12): Per 1000 distribution of fully economically independent aged persons by number of dependants for each sex and State/UT

Urban Males

state/ut	number of dependants									no. of eco. independent aged persons	
	0	1	2	3	4	5	6-8	9 & above	total	estd. (00)	sample
(1)	(2)	(3)	(4)	(5)	(6)	(7)	(8)	(9)	(10)	(11)	(12)
A & N Islands	0	386	129	113	147	169	56	0	1000	15	16
Chandigarh	135	340	397	10	50	0	69	0	1000	174	55
Dadra & N. Haveli	0	379	360	261	0	0	0	0	1000	1	7
Daman & Diu	59	74	0	866	0	0	0	0	1000	3	4
Lakshadweep	0	0	0	0	0	0	1000	0	1000	1	2
Pondicherry	143	425	228	67	37	75	26	0	1000	99	22
ONES	28	288	199	119	122	125	108	11	1000	284	208
GUTs	129	368	321	44	49	34	55	0	1000	294	106
all-India	83	396	256	94	70	42	44	15	1000	43733	3512
estd. no. of eco. indep. aged persons (00)	3634	17324	11174	4111	3069	1853	1915	653	43733	X	X
sample eco. indep. aged persons	269	1302	916	354	256	171	181	63	3512	X	X

For peer review only - http://bmjopen.bmj.com/site/about/guidelines.xhtml

Table (12): Per 1000 distribution of fully economically independent aged persons by number of dependants for each sex and State/UT

Urban Females

state/ut	number of dependants									no. of eco. independent aged persons	
	0	1	2	3	4	5	6-8	9 & above	total	estd. (00)	sample
(1)	(2)	(3)	(4)	(5)	(6)	(7)	(8)	(9)	(10)	(11)	(12)
Andhra Pradesh	285	138	545	7	0	23	2	0	1000	1518	80
Arunachal Pradesh	0	194	581	226	0	0	0	0	1000	1	5
Assam	0	301	309	120	271	0	0	0	1000	122	13
Bihar	193	351	239	13	0	0	182	22	1000	287	25
Chhattisgarh	297	133	440	0	0	130	0	0	1000	177	16
Delhi	157	304	470	53	8	0	8	0	1000	369	36
Goa	600	0	0	400	0	0	0	0	1000	9	2
Gujarat	445	81	361	90	23	0	0	0	1000	595	38
Haryana	287	107	606	0	0	0	0	0	1000	299	20
Himachal Pradesh	578	154	268	0	0	0	0	0	1000	47	13
Jammu & Kashmir	523	146	252	34	0	0	0	45	1000	35	16
Jharkhand	42	258	590	87	0	23	0	0	1000	160	14
Karnataka	290	184	404	27	95	0	0	0	1000	563	51
Kerala	336	135	486	12	19	0	0	12	1000	884	66
Madhya Pradesh	437	58	479	25	2	0	0	0	1000	806	62
Maharashtra	463	109	385	14	20	3	5	0	1000	2539	118
Manipur	60	205	516	166	0	53	0	0	1000	15	12
Meghalaya	74	53	873	0	0	0	0	0	1000	11	11
Mizoram	0	265	312	129	131	22	140	0	1000	11	20
Nagaland	0	0	898	0	0	102	0	0	1000	3	2
Orissa	70	343	571	0	16	0	0	0	1000	73	8
Punjab	279	197	323	0	201	0	0	0	1000	285	23
Rajasthan	518	111	179	0	121	0	71	0	1000	396	30
Sikkim	0	0	1000	0	0	0	0	0	1000	3	4
Tamil Nadu	547	127	261	46	6	14	0	0	1000	1730	108
Tripura	203	215	528	55	0	0	0	0	1000	33	10
Uttaranchal	98	180	232	0	490	0	0	0	1000	129	10
Uttar Pradesh	44	193	556	49	119	0	37	3	1000	1306	87
West Bengal	281	159	417	91	4	5	44	0	1000	1525	78

Continued

For peer review only - http://bmjopen.bmj.com/site/about/guidelines.xhtml

Appendix A

Table (12): Per 1000 distribution of fully economically independent aged persons by number of dependants for each sex and State/UT

Urban Females

state/ut	number of dependants									no. of eco. independent aged persons	
	0	1	2	3	4	5	6-8	9 & above	total	estd. (00)	sample
(1)	(2)	(3)	(4)	(5)	(6)	(7)	(8)	(9)	(10)	(11)	(12)
A & N Islands	0	156	511	111	0	222	0	0	1000	3	7
Chandigarh	309	157	485	0	49	0	0	0	1000	59	27
Dadra & N. Haveli	0	0	0	0	0	0	0	0	0	0	0
Daman & Diu	1000	0	0	0	0	0	0	0	1000	1	2
Lakshadweep	0	0	0	0	0	0	1000	0	1000	1	1
Pondicherry	616	0	384	0	0	0	0	0	1000	38	7
ONES	110	181	576	77	19	18	20	0	1000	77	64
GUTs	419	96	440	3	28	6	7	0	1000	102	44
all-India	341	146	417	35	37	7	15	2	1000	14033	1022
estd. no. of eco. indep. aged persons (00)	4782	2049	5852	492	517	103	217	22	14033	X	X
sample eco. indep. aged persons	320	176	415	43	33	12	17	6	1022	X	X

For peer review only - http://bmjopen.bmj.com/site/about/guidelines.xhtml

Appendix A

Table (12): Per 1000 distribution of fully economically independent aged persons by number of dependants for each sex and State/UT

Urban — Persons

state/ut	number of dependants									no. of eco. independent aged persons	
	0	1	2	3	4	5	6-8	9 & above	total	estd. (00)	sample
(1)	(2)	(3)	(4)	(5)	(6)	(7)	(8)	(9)	(10)	(11)	(12)
Andhra Pradesh	139	381	318	42	53	33	33	1	1000	4529	292
Arunachal Pradesh	0	340	502	54	39	49	0	15	1000	4	17
Assam	0	248	265	283	158	40	5	0	1000	486	78
Bihar	94	242	209	68	73	46	161	108	1000	1368	117
Chhattisgarh	201	124	396	116	46	98	19	0	1000	554	52
Delhi	89	492	330	30	50	2	5	1	1000	1725	164
Goa	59	292	61	392	153	43	0	0	1000	95	16
Gujarat	157	359	302	57	67	20	25	13	1000	3005	199
Haryana	121	382	385	5	39	43	9	17	1000	940	81
Himachal Pradesh	249	350	314	79	8	0	0	0	1000	148	47
Jammu & Kashmir	168	273	256	109	89	89	0	15	1000	290	86
Jharkhand	15	209	413	103	62	53	92	54	1000	844	106
Karnataka	129	335	259	125	57	43	30	22	1000	2757	247
Kerala	161	349	351	58	55	9	12	4	1000	2751	212
Madhya Pradesh	174	398	227	45	59	47	25	25	1000	3427	264
Maharashtra	200	385	299	70	19	10	17	0	1000	8928	451
Manipur	25	157	235	120	153	205	75	29	1000	87	53
Meghalaya	17	438	423	25	58	39	0	0	1000	50	39
Mizoram	0	243	280	139	103	29	199	8	1000	54	93
Nagaland	0	126	173	195	83	175	248	0	1000	53	17
Orissa	9	363	236	98	123	141	25	5	1000	849	80
Punjab	188	354	250	124	58	22	5	0	1000	1557	102
Rajasthan	242	304	175	45	101	49	83	1	1000	2059	178
Sikkim	0	204	447	207	0	142	0	0	1000	11	15
Tamil Nadu	254	303	267	91	34	31	17	3	1000	6049	409
Tripura	132	363	274	75	96	43	17	0	1000	102	38
Uttaranchal	47	422	263	0	116	20	65	67	1000	591	48
Uttar Pradesh	19	316	325	84	103	52	77	24	1000	6789	465
West Bengal	129	261	323	123	84	33	43	5	1000	7270	418

Continued

For peer review only - http://bmjopen.bmj.com/site/about/guidelines.xhtml

Appendix A

Table (12): Per 1000 distribution of fully economically independent aged persons by number of dependants for each sex and State/UT

Urban Persons

state/ut	number of dependants									no. of eco. independent aged persons	
	0	1	2	3	4	5	6-8	9 & above	total	estd. (00)	sample
(1)	(2)	(3)	(4)	(5)	(6)	(7)	(8)	(9)	(10)	(11)	(12)
A & N Islands	0	351	187	112	124	177	48	0	1000	18	23
Chandigarh	179	294	419	8	49	0	51	0	1000	233	82
Dadra & N. Haveli	0	379	360	261	0	0	0	0	1000	1	7
Daman & Diu	294	56	0	651	0	0	0	0	1000	4	6
Lakshadweep	0	0	0	0	0	0	1000	0	1000	1	3
Pondicherry	274	307	271	48	27	54	19	0	1000	137	29
ONES	46	265	279	110	100	102	89	8	1000	361	272
GUTs	203	298	351	33	44	27	43	0	1000	395	150
all-India	146	335	295	80	62	34	37	12	1000	57766	4534
estd. no. of eco. indep. aged persons (00)	8417	19373	17027	4603	3585	1955	2132	674	57766	X	X
sample eco. indep. aged persons	589	1478	1331	397	289	183	198	69	4534	X	X

For peer review only - http://bmjopen.bmj.com/site/about/guidelines.xhtml

Table (12): Per 1000 distribution of fully economically independent aged persons by number of dependants for each sex and State/UT

Rural + Urban — Males

state/ut	number of dependants									no. of eco. independent aged persons	
	0	1	2	3	4	5	6-8	9 & above	total	estd. (00)	sample
(1)	(2)	(3)	(4)	(5)	(6)	(7)	(8)	(9)	(10)	(11)	(12)
Andhra Pradesh	88	436	232	68	85	38	39	14	1000	12113	519
Arunachal Pradesh	0	103	357	192	100	119	89	40	1000	79	63
Assam	23	230	175	148	136	109	138	41	1000	3069	277
Bihar	44	224	256	110	100	57	146	62	1000	12055	631
Chhattisgarh	113	205	265	99	102	90	90	36	1000	2855	146
Delhi	93	524	297	22	56	3	4	2	1000	1493	134
Goa	240	512	96	115	29	8	0	0	1000	508	27
Gujarat	176	390	201	82	54	23	58	15	1000	7571	338
Haryana	55	335	271	42	115	56	59	66	1000	2746	153
Himachal Pradesh	76	373	273	77	58	25	110	8	1000	1489	218
Jammu & Kashmir	102	314	280	71	118	69	46	2	1000	1723	216
Jharkhand	29	293	251	69	72	92	148	45	1000	4217	292
Karnataka	93	354	174	136	94	63	58	29	1000	8496	430
Kerala	39	450	316	81	54	27	28	5	1000	6087	336
Madhya Pradesh	108	391	141	61	75	75	93	56	1000	11062	515
Maharashtra	90	423	288	89	26	45	36	4	1000	17821	672
Manipur	7	164	261	93	171	179	100	24	1000	183	98
Meghalaya	15	185	408	96	129	18	150	0	1000	307	76
Mizoram	0	265	201	169	78	41	223	22	1000	111	127
Nagaland	0	148	264	186	158	87	157	0	1000	103	43
Orissa	47	343	253	101	86	81	81	7	1000	6749	296
Punjab	70	315	349	89	73	44	48	11	1000	4385	198
Rajasthan	102	385	139	81	96	47	76	74	1000	7389	407
Sikkim	29	121	321	245	68	94	117	5	1000	79	72
Tamil Nadu	94	404	253	119	51	43	31	4	1000	12572	564
Tripura	31	209	252	183	195	60	43	26	1000	492	105
Uttaranchal	60	276	170	85	116	181	90	23	1000	2107	111
Uttar Pradesh	31	276	244	92	89	83	114	70	1000	31087	1486
West Bengal	59	257	255	142	127	49	86	25	1000	14130	642

Continued

For peer review only - http://bmjopen.bmj.com/site/about/guidelines.xhtml

Appendix A

Table (12): Per 1000 distribution of fully economically independent aged persons by number of dependants for each sex and State/UT

Rural + Urban Males

state/ut	number of dependants									no. of eco. independent aged persons	
	0	1	2	3	4	5	6-8	9 & above	total	estd. (00)	sample
(1)	(2)	(3)	(4)	(5)	(6)	(7)	(8)	(9)	(10)	(11)	(12)
A & N Islands	0	245	334	144	225	41	11	0	1000	80	44
Chandigarh	135	355	383	12	49	0	66	0	1000	181	71
Dadra & N. Haveli	0	246	730	24	0	0	0	0	1000	14	10
Daman & Diu	80	117	0	803	0	0	0	0	1000	6	8
Lakshadweep	20	60	0	0	160	51	709	0	1000	3	8
Pondicherry	159	445	107	113	17	35	60	65	1000	211	34
ONES	17	186	295	154	151	72	105	17	1000	1353	584
GUTs	118	368	261	86	62	22	55	28	1000	495	175
all-India	72	340	239	96	81	60	78	34	1000	173573	9367
estd. no. of eco. indep. aged persons (00)	12536	58976	41538	16644	14076	10333	13567	5903	173573	X	X
sample eco. indep. aged persons	630	3117	2295	941	759	564	721	340	9367	X	X

For peer review only - http://bmjopen.bmj.com/site/about/guidelines.xhtml

Appendix A

Table (12): Per 1000 distribution of fully economically independent aged persons by number of dependants for each sex and State/UT

Rural + Urban Females

state/ut	number of dependants									no. of eco. independent aged persons	
	0	1	2	3	4	5	6-8	9 & above	total	estd. (00)	sample
(1)	(2)	(3)	(4)	(5)	(6)	(7)	(8)	(9)	(10)	(12)	(13)
Andhra Pradesh	338	186	426	3	24	22	1	0	1000	4492	169
Arunachal Pradesh	0	291	189	387	0	128	0	5	1000	23	16
Assam	168	267	378	117	70	0	0	0	1000	470	37
Bihar	199	134	454	85	7	2	105	15	1000	2840	148
Chhattisgarh	172	202	419	26	20	47	90	24	1000	1926	91
Delhi	157	304	470	53	8	0	8	0	1000	369	36
Goa	489	33	457	22	0	0	0	0	1000	169	10
Gujarat	600	192	166	28	7	0	0	8	1000	1937	88
Haryana	393	55	497	0	41	0	0	14	1000	997	49
Himachal Pradesh	383	50	513	31	17	0	6	0	1000	513	76
Jammu & Kashmir	644	61	149	6	117	14	0	8	1000	202	34
Jharkhand	188	152	330	26	55	20	0	228	1000	899	43
Karnataka	505	129	245	44	45	25	7	0	1000	2176	106
Kerala	312	135	496	42	12	0	0	4	1000	2421	128
Madhya Pradesh	471	143	314	26	30	0	16	0	1000	3045	141
Maharashtra	369	139	407	52	16	2	15	0	1000	6995	230
Manipur	59	222	334	95	150	140	0	0	1000	39	26
Meghalaya	52	162	611	68	0	0	106	0	1000	87	34
Mizoram	0	350	235	146	71	12	186	0	1000	21	26
Nagaland	0	0	957	0	16	27	0	0	1000	12	7
Orissa	110	300	511	12	1	65	0	0	1000	982	44
Punjab	223	141	572	0	61	0	3	0	1000	939	46
Rajasthan	426	187	164	100	90	11	23	0	1000	1624	78
Sikkim	0	48	733	73	0	0	146	0	1000	15	14
Tamil Nadu	490	231	229	30	2	17	2	0	1000	5039	196
Tripura	266	293	422	19	0	0	0	0	1000	95	20
Uttaranchal	306	126	359	98	100	2	10	0	1000	897	47
Uttar Pradesh	116	148	480	74	84	21	41	36	1000	6563	300
West Bengal	262	203	430	71	2	7	25	0	1000	2705	126

Continued

For peer review only - http://bmjopen.bmj.com/site/about/guidelines.xhtml

Appendix A

Table (12): Per 1000 distribution of fully economically independent aged persons by number of dependants for each sex and State/UT

Rural + Urban Females

state/ut	number of dependants									no. of eco. independent aged persons	
	0	1	2	3	4	5	6-8	9 & above	total	estd. (00)	sample
(1)	(2)	(3)	(4)	(5)	(6)	(7)	(8)	(9)	(10)	(12)	(13)
A & N Islands	0	221	619	14	119	27	0	0	1000	23	13
Chandigarh	303	182	468	0	47	0	0	0	1000	61	29
Dadra & N. Haveli	331	669	0	0	0	0	0	0	1000	5	2
Daman & Diu	1000	0	0	0	0	0	0	0	1000	4	5
Lakshadweep	233	0	184	0	0	0	312	270	1000	2	5
Pondicherry	545	314	141	0	0	0	0	0	1000	103	13
ONES	110	224	473	84	26	31	52	0	1000	293	143
GUTs	409	260	291	2	28	3	4	3	1000	199	67
all-India	326	167	383	46	32	13	22	12	1000	48692	2433
estd. no. of eco. indep. aged persons (00)	15887	8110	18668	2229	1543	621	1058	575	48692	X	X
sample eco. indep. aged persons	736	421	962	112	82	37	51	32	2433	X	X

For peer review only

For peer review only - http://bmjopen.bmj.com/site/about/guidelines.xhtml

Appendix A

Table (12): Per 1000 distribution of fully economically independent aged persons by number of dependants for each sex and State/UT

Rural + Urban — Persons

state/ut	number of dependants									no. of eco. independent aged persons	
	0	1	2	3	4	5	6-8	9 & above	total	estd. (00)	sample
(1)	(2)	(3)	(4)	(5)	(6)	(7)	(8)	(9)	(10)	(11)	(12)
Andhra Pradesh	156	368	285	51	68	34	29	10	1000	16606	688
Arunachal Pradesh	0	145	319	236	77	121	69	32	1000	102	79
Assam	43	235	202	144	127	94	120	35	1000	3538	314
Bihar	74	207	294	105	83	47	138	53	1000	14895	779
Chhattisgarh	137	204	327	70	69	73	90	31	1000	4780	237
Delhi	106	480	331	28	47	2	5	1	1000	1862	170
Goa	302	392	186	92	22	6	0	0	1000	677	37
Gujarat	262	350	194	71	44	18	46	14	1000	9508	426
Haryana	145	261	331	31	96	41	44	52	1000	3744	202
Himachal Pradesh	155	290	335	65	48	19	83	6	1000	2002	294
Jammu & Kashmir	158	287	266	64	118	63	41	2	1000	1925	250
Jharkhand	57	268	265	62	69	79	122	77	1000	5117	335
Karnataka	177	308	188	117	84	55	47	23	1000	10672	536
Kerala	117	361	367	70	42	19	20	5	1000	8508	464
Madhya Pradesh	186	338	179	54	66	59	76	44	1000	14107	656
Maharashtra	169	343	321	78	23	33	30	3	1000	24816	902
Manipur	17	175	274	93	168	172	82	20	1000	222	124
Meghalaya	23	180	453	90	100	14	140	0	1000	394	110
Mizoram	0	279	206	165	77	36	218	19	1000	131	153
Nagaland	0	133	336	167	143	81	140	0	1000	115	50
Orissa	55	338	286	90	75	79	71	6	1000	7731	340
Punjab	97	284	388	74	71	37	40	9	1000	5324	244
Rajasthan	160	349	143	85	94	41	67	61	1000	9013	485
Sikkim	25	109	385	218	57	79	122	4	1000	93	86
Tamil Nadu	207	355	246	94	37	36	23	3	1000	17611	760
Tripura	69	223	280	156	163	50	36	22	1000	588	125
Uttaranchal	133	231	227	89	111	127	66	16	1000	3004	158
Uttar Pradesh	46	254	285	89	88	72	102	64	1000	37650	1786
West Bengal	92	249	283	131	107	42	76	21	1000	16835	768

Continued

For peer review only - http://bmjopen.bmj.com/site/about/guidelines.xhtml

Appendix A

Table (12): Per 1000 distribution of fully economically independent aged persons by number of dependants for each sex and State/UT

Rural + Urban Persons

state/ut	number of dependants									no. of eco. independent aged persons	
	0	1	2	3	4	5	6-8	9 & above	total	estd. (00)	sample
(1)	(2)	(3)	(4)	(5)	(6)	(7)	(8)	(9)	(10)	(11)	(12)
A & N Islands	0	240	397	115	202	38	8	0	1000	103	57
Chandigarh	177	312	405	9	48	0	49	0	1000	243	100
Dadra & N. Haveli	83	352	548	18	0	0	0	0	1000	19	12
Daman & Diu	476	67	0	458	0	0	0	0	1000	10	13
Lakshadweep	117	33	84	0	87	28	529	122	1000	5	13
Pondicherry	286	402	118	76	12	24	40	44	1000	314	47
ONES	34	193	327	142	129	65	96	14	1000	1646	727
GUTs	201	337	269	61	53	17	41	21	1000	694	242
all-India	128	302	271	85	70	49	66	29	1000	222264	11800
estd. no. of eco. indep. aged persons (00)	28423	67086	60206	18873	15619	10954	14626	6478	222264	X	X
sample eco. indep. aged persons	1366	3538	3257	1053	841	601	772	372	11800	X	X

For peer review only

For peer review only - http://bmjopen.bmj.com/site/about/guidelines.xhtml

Appendix A

Table (13): Per 1000 distribution of economically dependent aged persons by category of persons financially supporting the aged person for each sex

Rural Males

state/ut	persons supporting the aged person					no. of economically dependent aged persons	
	spouse	own children	grand children	others	total	estd. (00)	sample
(1)	(2)	(3)	(4)	(5)	(6)	(7)	(8)
Andhra Pradesh	102	835	19	45	1000	9448	386
Arunachal Pradesh	48	865	3	84	1000	104	62
Assam	13	948	23	16	1000	3175	245
Bihar	85	853	24	38	1000	7609	360
Chhattisgarh	106	713	35	147	1000	1981	92
Delhi	50	808	6	136	1000	201	10
Goa	0	1000	0	0	1000	84	6
Gujarat	15	931	19	36	1000	5236	173
Haryana	14	920	0	66	1000	3494	159
Himachal Pradesh	34	857	18	91	1000	965	156
Jammu & Kashmir	19	852	33	96	1000	727	79
Jharkhand	59	855	54	33	1000	2800	127
Karnataka	125	788	26	61	1000	5333	193
Kerala	79	856	8	56	1000	7425	314
Madhya Pradesh	51	829	57	62	1000	5892	226
Maharashtra	91	857	14	37	1000	11839	377
Manipur	153	752	67	29	1000	209	116
Meghalaya	38	962	0	0	1000	190	45
Mizoram	218	782	0	0	1000	42	28
Nagaland	73	827	101	0	1000	53	26
Orissa	131	785	25	59	1000	7092	293
Punjab	86	856	28	31	1000	3637	135
Rajasthan	29	855	34	82	1000	6387	291
Sikkim	25	889	40	45	1000	44	48
Tamil Nadu	99	867	12	22	1000	8708	308
Tripura	22	913	21	44	1000	484	91
Uttaranchal	99	838	44	19	1000	871	27
Uttar Pradesh	32	838	14	116	1000	16253	685
West Bengal	66	869	25	40	1000	9299	369
A & N Islands	357	619	0	24	1000	12	6
Chandigarh	1000	0	0	0	1000	0	1
Dadra & N. Haveli	0	854	0	146	1000	65	17
Daman & Diu	660	340	0	0	1000	1	3
Lakshadweep	0	772	0	228	1000	5	10
Pondicherry	0	1000	0	0	1000	49	8
ONES	61	877	28	34	1000	1126	416
GUTs	39	878	0	83	1000	132	45
all-India	70	850	22	57	1000	119714	5472
estd. no. of eco. dep. aged persons (00)	8426	101800	2630	6858	119714	X	X
sample eco. dep. aged persons	372	4650	129	321	5472	X	X

For peer review only - http://bmjopen.bmj.com/site/about/guidelines.xhtml

Appendix A

Table (13): Per 1000 distribution of economically dependent aged persons by category of persons financially supporting the aged person for each sex

Rural Females

state/ut	persons supporting the aged person					no. of economically dependent aged persons	
	spouse	own children	grand children	others	total	estd. (00)	sample
(1)	(2)	(3)	(4)	(5)	(6)	(7)	(8)
Andhra Pradesh	82	801	25	92	1000	16865	594
Arunachal Pradesh	34	871	2	93	1000	153	88
Assam	63	872	19	46	1000	3700	281
Bihar	230	707	23	40	1000	12411	539
Chhattisgarh	131	680	37	153	1000	4773	210
Delhi	282	580	53	85	1000	322	17
Goa	412	588	0	0	1000	455	24
Gujarat	144	785	34	37	1000	9051	319
Haryana	169	765	9	57	1000	4812	203
Himachal Pradesh	182	750	27	41	1000	1999	275
Jammu & Kashmir	233	728	29	11	1000	1378	151
Jharkhand	151	766	65	19	1000	3612	197
Karnataka	89	795	36	80	1000	9165	346
Kerala	88	831	26	55	1000	13254	576
Madhya Pradesh	216	675	33	76	1000	12051	452
Maharashtra	138	745	21	97	1000	19717	595
Manipur	82	779	46	92	1000	217	124
Meghalaya	21	889	0	90	1000	334	69
Mizoram	293	665	0	42	1000	55	38
Nagaland	153	847	0	0	1000	30	19
Orissa	152	749	43	55	1000	10915	435
Punjab	256	702	40	2	1000	5849	215
Rajasthan	195	728	46	31	1000	11768	534
Sikkim	330	536	72	62	1000	88	82
Tamil Nadu	109	788	30	72	1000	13836	461
Tripura	96	796	73	35	1000	602	124
Uttaranchal	205	754	27	14	1000	1401	51
Uttar Pradesh	236	672	26	66	1000	35057	1403
West Bengal	112	787	48	53	1000	16660	623
A & N Islands	299	601	64	37	1000	16	8
Chandigarh	284	276	0	440	1000	6	9
Dadra & N. Haveli	0	1000	0	0	1000	34	8
Daman & Diu	155	728	0	117	1000	10	10
Lakshadweep	24	751	85	140	1000	7	18
Pondicherry	0	902	0	98	1000	83	12
ONES	93	803	41	63	1000	1479	544
GUTs	53	849	11	87	1000	157	65
all-India	159	746	31	63	1000	210689	9110
estd. no. of eco. dep. aged persons (00)	33532	157196	6612	13349	210689	X	X
sample eco. dep. aged persons	1537	6763	274	536	9110	X	X

For peer review only - http://bmjopen.bmj.com/site/about/guidelines.xhtml

Appendix A

Table (13): Per 1000 distribution of economically dependent aged persons by category of persons financially supporting the aged person for each sex

Rural Persons

state/ut	persons supporting the aged person					no. of economically dependent aged persons	
	spouse	own children	grand children	others	total	estd. (00)	sample
(1)	(2)	(3)	(4)	(5)	(6)	(7)	(8)
Andhra Pradesh	89	813	23	75	1000	26313	980
Arunachal Pradesh	40	869	2	89	1000	256	150
Assam	40	907	21	32	1000	6875	526
Bihar	175	763	23	39	1000	20020	899
Chhattisgarh	123	689	36	151	1000	6754	302
Delhi	193	667	35	105	1000	524	27
Goa	348	652	0	0	1000	539	30
Gujarat	96	838	29	37	1000	14287	492
Haryana	104	830	5	61	1000	8306	362
Himachal Pradesh	134	785	24	57	1000	2964	431
Jammu & Kashmir	159	771	30	40	1000	2105	230
Jharkhand	110	805	60	25	1000	6412	324
Karnataka	102	792	33	73	1000	14498	539
Kerala	85	840	20	55	1000	20679	890
Madhya Pradesh	162	726	41	71	1000	17943	678
Maharashtra	120	787	18	74	1000	31555	972
Manipur	117	766	56	61	1000	426	240
Meghalaya	27	915	0	57	1000	524	114
Mizoram	261	715	0	24	1000	97	66
Nagaland	102	834	64	0	1000	83	45
Orissa	144	763	36	57	1000	18007	728
Punjab	190	761	35	13	1000	9487	350
Rajasthan	136	773	42	49	1000	18155	825
Sikkim	228	654	61	56	1000	133	130
Tamil Nadu	105	819	23	53	1000	22544	769
Tripura	63	848	50	39	1000	1086	215
Uttaranchal	164	786	34	16	1000	2272	78
Uttar Pradesh	172	724	22	82	1000	51310	2088
West Bengal	95	816	40	49	1000	25959	992
A & N Islands	323	609	37	31	1000	28	14
Chandigarh	300	270	0	430	1000	6	10
Dadra & N. Haveli	0	904	0	96	1000	99	25
Daman & Diu	203	692	0	106	1000	11	13
Lakshadweep	14	759	51	176	1000	12	28
Pondicherry	0	938	0	62	1000	132	20
ONES	79	835	35	50	1000	2605	960
GUTs	47	862	6	85	1000	289	110
all-India	127	784	28	61	1000	330404	14582
estd. no. of eco. dep. aged persons (00)	41958	258996	9242	20208	330404	X	X
sample eco. dep. aged persons	1909	11413	403	857	14582	X	X

For peer review only

For peer review only - http://bmjopen.bmj.com/site/about/guidelines.xhtml

Appendix A

Table (13): Per 1000 distribution of economically dependent aged persons by category of persons financially supporting the aged person for each sex

Urban Males

state/ut	persons supporting the aged person					no. of economically dependent aged persons	
	spouse	own children	grand children	others	total	estd. (00)	sample
(1)	(2)	(3)	(4)	(5)	(6)	(7)	(8)
Andhra Pradesh	51	880	15	54	1000	2284	164
Arunachal Pradesh	0	855	0	145	1000	6	26
Assam	147	827	0	26	1000	283	44
Bihar	53	911	1	35	1000	1233	71
Chhattisgarh	216	724	60	0	1000	265	27
Delhi	77	838	82	3	1000	826	80
Goa	85	659	0	255	1000	143	18
Gujarat	51	895	36	18	1000	2183	136
Haryana	88	882	12	18	1000	666	70
Himachal Pradesh	0	943	0	57	1000	39	15
Jammu & Kashmir	0	929	0	71	1000	151	30
Jharkhand	44	951	0	5	1000	682	73
Karnataka	67	847	25	60	1000	1794	147
Kerala	74	810	0	116	1000	2102	142
Madhya Pradesh	42	851	24	83	1000	1422	112
Maharashtra	19	932	11	39	1000	6359	260
Manipur	292	705	2	0	1000	99	66
Meghalaya	107	893	0	0	1000	4	6
Mizoram	36	878	0	86	1000	30	59
Nagaland	0	1000	0	0	1000	19	9
Orissa	69	808	44	79	1000	751	74
Punjab	172	771	19	38	1000	1229	82
Rajasthan	28	926	22	24	1000	1344	122
Sikkim	0	1000	0	0	1000	3	5
Tamil Nadu	51	856	25	68	1000	3635	286
Tripura	40	960	0	0	1000	62	17
Uttaranchal	0	928	0	72	1000	97	11
Uttar Pradesh	75	824	19	82	1000	3507	231
West Bengal	91	811	5	93	1000	2861	177
A & N Islands	0	1000	0	0	1000	2	1
Chandigarh	0	943	57	0	1000	23	14
Dadra & N. Haveli	0	1000	0	0	1000	2	6
Daman & Diu	128	780	0	92	1000	7	12
Lakshadweep	0	885	0	115	1000	7	19
Pondicherry	58	844	0	98	1000	119	25
ONES	148	835	1	16	1000	222	188
GUTs	48	861	8	82	1000	161	77
all-India	60	865	18	57	1000	34239	2637
estd. no. of eco. dep. aged persons (00)	2064	29604	616	1955	34239	X	X
sample eco. dep. aged persons	172	2250	52	163	2637	X	X

For peer review only - http://bmjopen.bmj.com/site/about/guidelines.xhtml

Appendix A

Table (13): Per 1000 distribution of economically dependent aged persons by category of persons financially supporting the aged person for each sex

Urban Females

state/ut	persons supporting the aged person					no. of economically dependent aged persons	
	spouse	own children	grand children	others	total	estd. (00)	sample
(1)	(2)	(3)	(4)	(5)	(6)	(7)	(8)
Andhra Pradesh	175	748	21	56	1000	4548	336
Arunachal Pradesh	11	780	45	163	1000	6	22
Assam	152	833	0	15	1000	374	68
Bihar	309	664	8	18	1000	1466	116
Chhattisgarh	233	659	48	60	1000	583	72
Delhi	287	564	114	34	1000	1736	165
Goa	117	766	5	112	1000	247	38
Gujarat	194	748	20	37	1000	4461	279
Haryana	154	783	8	54	1000	1370	119
Himachal Pradesh	233	727	20	20	1000	107	36
Jammu & Kashmir	231	746	0	23	1000	296	72
Jharkhand	213	722	18	47	1000	1096	121
Karnataka	132	760	41	66	1000	3491	299
Kerala	101	765	18	117	1000	3724	249
Madhya Pradesh	246	659	29	65	1000	3513	259
Maharashtra	153	751	21	74	1000	11043	549
Manipur	172	732	6	90	1000	139	93
Meghalaya	87	881	0	32	1000	36	34
Mizoram	166	801	7	25	1000	45	74
Nagaland	622	378	0	0	1000	5	3
Orissa	139	796	12	53	1000	1061	102
Punjab	285	656	34	24	1000	2038	138
Rajasthan	194	730	23	53	1000	2680	236
Sikkim	318	682	0	0	1000	5	9
Tamil Nadu	152	738	31	79	1000	7250	541
Tripura	30	879	16	75	1000	103	44
Uttaranchal	410	521	69	0	1000	542	38
Uttar Pradesh	243	636	46	75	1000	8119	522
West Bengal	224	652	28	96	1000	6710	417
A & N Islands	520	299	0	182	1000	12	15
Chandigarh	442	462	0	96	1000	86	39
Dadra & N. Haveli	223	659	29	89	1000	3	14
Daman & Diu	56	944	0	0	1000	14	19
Lakshadweep	33	809	0	158	1000	10	23
Pondicherry	62	795	36	107	1000	330	49
ONES	124	797	9	70	1000	339	279
GUTs	146	723	26	104	1000	456	159
all-India	192	710	30	68	1000	67250	5210
estd. no. of eco. dep. aged persons (00)	12917	47763	1991	4578	67250	X	X
sample eco. dep. aged persons	1003	3711	161	335	5210	X	X

For peer review only - http://bmjopen.bmj.com/site/about/guidelines.xhtml

Appendix A

Table (13): Per 1000 distribution of economically dependent aged persons by category of persons financially supporting the aged person for each sex

Urban Persons

state/ut	persons supporting the aged person					no. of economically dependent aged persons	
	spouse	own children	grand children	others	total	estd. (00)	sample
(1)	(2)	(3)	(4)	(5)	(6)	(7)	(8)
Andhra Pradesh	134	792	19	55	1000	6832	500
Arunachal Pradesh	6	818	22	154	1000	12	48
Assam	150	830	0	20	1000	657	112
Bihar	192	777	5	26	1000	2698	187
Chhattisgarh	227	679	52	42	1000	847	99
Delhi	219	653	104	24	1000	2563	245
Goa	106	727	3	164	1000	390	56
Gujarat	147	796	25	31	1000	6644	415
Haryana	132	816	10	42	1000	2036	189
Himachal Pradesh	171	785	14	30	1000	145	51
Jammu & Kashmir	153	807	0	39	1000	447	102
Jharkhand	148	810	11	31	1000	1778	194
Karnataka	110	790	36	64	1000	5286	446
Kerala	91	781	11	116	1000	5827	391
Madhya Pradesh	187	715	28	70	1000	4935	371
Maharashtra	104	817	17	61	1000	17402	809
Manipur	222	721	4	53	1000	238	159
Meghalaya	89	882	0	29	1000	40	40
Mizoram	114	832	4	50	1000	75	133
Nagaland	123	877	0	0	1000	23	12
Orissa	110	801	25	64	1000	1813	176
Punjab	243	699	29	29	1000	3267	220
Rajasthan	139	795	23	43	1000	4024	358
Sikkim	204	796	0	0	1000	7	14
Tamil Nadu	118	777	29	75	1000	10886	827
Tripura	34	909	10	47	1000	165	61
Uttaranchal	348	582	59	11	1000	638	49
Uttar Pradesh	192	693	38	77	1000	11626	753
West Bengal	184	700	21	95	1000	9571	594
A & N Islands	446	399	0	156	1000	14	16
Chandigarh	349	564	12	75	1000	109	53
Dadra & N. Haveli	129	804	17	51	1000	6	20
Daman & Diu	80	889	0	31	1000	21	31
Lakshadweep	19	842	0	140	1000	17	42
Pondicherry	61	808	27	105	1000	449	74
ONES	133	812	6	48	1000	561	467
GUTs	121	759	22	99	1000	617	236
all-India	148	762	26	64	1000	101489	7847
estd. no. of eco. dep. aged persons (00)	14981	77367	2607	6533	101489	X	X
sample eco. dep. aged persons	1175	5961	213	498	7847	X	X

For peer review only

For peer review only - http://bmjopen.bmj.com/site/about/guidelines.xhtml

Appendix A

Table (13): Per 1000 distribution of economically dependent aged persons by category of persons financially supporting the aged person for each sex

Rural + Urban Males

state/ut	persons supporting the aged person					no. of economically dependent aged persons	
	spouse	own children	grand children	others	total	estd. (00)	sample
(1)	(2)	(3)	(4)	(5)	(6)	(7)	(8)
Andhra Pradesh	92	844	18	46	1000	11732	550
Arunachal Pradesh	46	864	3	87	1000	110	88
Assam	24	938	21	17	1000	3458	289
Bihar	81	861	21	38	1000	8842	431
Chhattisgarh	119	714	38	129	1000	2245	119
Delhi	72	832	67	29	1000	1028	90
Goa	54	786	0	161	1000	227	24
Gujarat	25	920	24	30	1000	7419	309
Haryana	26	914	2	58	1000	4160	229
Himachal Pradesh	32	860	17	90	1000	1004	171
Jammu & Kashmir	16	865	27	92	1000	877	109
Jharkhand	56	873	43	28	1000	3482	200
Karnataka	110	803	26	61	1000	7127	340
Kerala	78	846	6	70	1000	9528	456
Madhya Pradesh	50	834	51	66	1000	7314	338
Maharashtra	66	883	13	38	1000	18197	637
Manipur	197	737	46	20	1000	308	182
Meghalaya	40	960	0	0	1000	194	51
Mizoram	141	823	0	36	1000	72	87
Nagaland	54	872	74	0	1000	71	35
Orissa	125	787	27	61	1000	7843	367
Punjab	108	834	26	33	1000	4867	217
Rajasthan	29	867	32	72	1000	7731	413
Sikkim	24	896	38	43	1000	47	53
Tamil Nadu	85	864	16	35	1000	12344	594
Tripura	24	918	18	39	1000	546	108
Uttaranchal	89	847	40	24	1000	968	38
Uttar Pradesh	40	836	15	110	1000	19760	916
West Bengal	72	855	20	52	1000	12160	546
A & N Islands	305	675	0	20	1000	14	7
Chandigarh	6	937	57	0	1000	23	15
Dadra & N. Haveli	0	859	0	141	1000	68	23
Daman & Diu	196	724	0	80	1000	8	15
Lakshadweep	0	840	0	160	1000	12	29
Pondicherry	41	889	0	70	1000	167	33
ONES	75	870	23	31	1000	1348	604
GUTs	44	869	5	83	1000	293	122
all-India	68	854	21	57	1000	153953	8109
estd. no. of eco. dep. aged persons (00)	10490	131404	3246	8813	153953	X	X
sample eco. dep. aged persons	544	6900	181	484	8109	X	X

For peer review only - http://bmjopen.bmj.com/site/about/guidelines.xhtml

Appendix A

Table (13): Per 1000 distribution of economically dependent aged persons by category of persons financially supporting the aged person for each sex

Rural + Urban — Females

state/ut	persons supporting the aged person					no. of economically dependent aged persons	
	spouse	own children	grand children	others	total	estd. (00)	sample
(1)	(2)	(3)	(4)	(5)	(6)	(7)	(8)
Andhra Pradesh	102	790	24	84	1000	21413	930
Arunachal Pradesh	33	868	3	96	1000	158	110
Assam	71	868	17	43	1000	4074	349
Bihar	238	703	21	38	1000	13877	655
Chhattisgarh	142	678	38	143	1000	5356	282
Delhi	286	567	105	42	1000	2058	182
Goa	309	650	2	39	1000	702	62
Gujarat	160	773	30	37	1000	13512	598
Haryana	166	769	9	56	1000	6182	322
Himachal Pradesh	185	749	26	40	1000	2106	311
Jammu & Kashmir	233	731	24	13	1000	1674	223
Jharkhand	165	756	54	25	1000	4708	318
Karnataka	101	785	38	76	1000	12657	645
Kerala	91	816	24	68	1000	16979	825
Madhya Pradesh	223	672	32	73	1000	15564	711
Maharashtra	143	747	21	89	1000	30760	1144
Manipur	117	761	31	91	1000	356	217
Meghalaya	28	888	0	84	1000	370	103
Mizoram	236	726	3	34	1000	100	112
Nagaland	215	785	0	0	1000	35	22
Orissa	151	754	41	55	1000	11977	537
Punjab	263	690	39	8	1000	7888	353
Rajasthan	195	729	42	35	1000	14448	770
Sikkim	330	543	69	59	1000	93	91
Tamil Nadu	124	771	31	75	1000	21086	1002
Tripura	86	808	65	41	1000	706	168
Uttaranchal	262	689	39	10	1000	1943	89
Uttar Pradesh	237	665	30	68	1000	43176	1925
West Bengal	144	748	42	66	1000	23370	1040
A & N Islands	393	472	36	98	1000	29	23
Chandigarh	431	450	0	119	1000	93	48
Dadra & N. Haveli	20	969	3	8	1000	37	22
Daman & Diu	97	855	0	48	1000	24	29
Lakshadweep	29	784	37	151	1000	17	41
Pondicherry	49	817	29	105	1000	413	61
ONES	99	802	35	64	1000	1818	823
GUTs	123	755	22	100	1000	613	224
all-India	167	737	31	65	1000	277939	14320
estd. no. of eco. dep. aged persons (00)	46449	204959	8603	17928	277939	X	X
sample eco. dep. aged persons	2540	10474	435	871	14320	X	X

For peer review only - http://bmjopen.bmj.com/site/about/guidelines.xhtml

Appendix A

Table (13): Per 1000 distribution of economically dependent aged persons by category of persons financially supporting the aged person for each sex

Rural + Urban Persons

state/ut	persons supporting the aged person					no. of economically dependent aged persons	
	spouse	own children	grand children	others	total	estd. (00)	sample
(1)	(2)	(3)	(4)	(5)	(6)	(7)	(8)
Andhra Pradesh	98	809	22	71	1000	33145	1480
Arunachal Pradesh	38	866	3	92	1000	268	198
Assam	49	901	19	31	1000	7532	638
Bihar	177	764	21	38	1000	22718	1086
Chhattisgarh	135	688	38	139	1000	7601	401
Delhi	215	655	92	38	1000	3086	272
Goa	246	683	1	69	1000	929	86
Gujarat	113	825	28	35	1000	20931	907
Haryana	110	827	6	57	1000	10342	551
Himachal Pradesh	135	785	23	56	1000	3110	482
Jammu & Kashmir	158	777	25	40	1000	2552	332
Jharkhand	119	806	49	26	1000	8190	518
Karnataka	104	792	33	71	1000	19784	985
Kerala	86	827	18	69	1000	26506	1281
Madhya Pradesh	167	724	38	71	1000	22878	1049
Maharashtra	114	798	18	70	1000	48957	1781
Manipur	154	750	38	58	1000	664	399
Meghalaya	32	913	0	55	1000	564	154
Mizoram	197	766	2	35	1000	172	199
Nagaland	106	843	50	0	1000	106	57
Orissa	140	767	35	57	1000	19820	904
Punjab	204	745	34	17	1000	12754	570
Rajasthan	137	777	38	48	1000	22179	1183
Sikkim	227	661	58	53	1000	140	144
Tamil Nadu	110	805	25	60	1000	33430	1596
Tripura	59	856	44	40	1000	1251	276
Uttaranchal	205	741	39	15	1000	2911	127
Uttar Pradesh	175	718	25	81	1000	62936	2841
West Bengal	119	785	35	61	1000	35530	1586
A & N Islands	364	538	25	73	1000	43	30
Chandigarh	346	548	11	95	1000	116	63
Dadra & N. Haveli	7	898	1	94	1000	105	45
Daman & Diu	122	822	0	56	1000	32	44
Lakshadweep	17	807	21	155	1000	29	70
Pondicherry	47	838	21	95	1000	581	94
ONES	89	831	30	50	1000	3165	1427
GUTs	97	792	17	94	1000	906	346
all-India	132	779	27	62	1000	431892	22429
estd. no. of eco. dep. aged persons (00)	56939	336363	11849	26741	431892	X	X
sample eco. dep. aged persons	3084	17374	616	1355	22429	X	X

For peer review only - http://bmjopen.bmj.com/site/about/guidelines.xhtml

Appendix A

Table (14): Per 1000 distribution of aged persons by type of living arrangement for each sex and State/UT

Rural Males

state/ut	living arrangement of aged persons									no. of aged persons	
	living alone		with spouse only	with spouse & other members	without spouse but with			n.r.	total	estd. (00)	sam-ple
	as an inmate of old age home	not as an inmate of old age home			children	other rela-tions	non-rela-tions				
(1)	(2)	(3)	(4)	(5)	(6)	(7)	(8)	(9)	(10)	(11)	(12)
Andhra Pradesh	1	36	324	479	138	11	1	10	1000	18730	69[illegible]
Arunachal Pradesh	0	21	45	560	125	0	0	250	1000	238	14[illegible]
Assam	0	3	42	733	165	9	1	47	1000	6103	47[illegible]
Bihar	12	19	149	599	193	7	3	19	1000	18861	91[illegible]
Chhattisgarh	24	13	181	510	204	13	22	33	1000	4609	20[illegible]
Delhi	0	0	0	757	114	129	0	0	1000	338	1[illegible]
Goa	0	0	270	657	28	46	0	0	1000	506	1[illegible]
Gujarat	0	40	182	551	207	14	0	6	1000	10458	35[illegible]
Haryana	0	0	70	696	209	23	0	1	1000	5608	25[illegible]
Himachal Pradesh	2	40	132	596	181	16	13	19	1000	2400	34[illegible]
Jammu & Kashmir	0	0	35	699	208	33	2	23	1000	2246	23[illegible]
Jharkhand	1	27	146	537	238	33	1	16	1000	6400	33[illegible]
Karnataka	2	19	138	685	129	26	0	1	1000	11642	42[illegible]
Kerala	0	13	127	698	137	22	1	2	1000	11673	50[illegible]
Madhya Pradesh	12	21	190	545	161	44	3	25	1000	14666	54[illegible]
Maharashtra	2	21	184	644	124	21	0	5	1000	23347	71[illegible]
Manipur	14	21	59	516	179	9	10	192	1000	395	21[illegible]
Meghalaya	0	32	108	700	115	2	0	44	1000	467	9[illegible]
Mizoram	0	10	30	630	236	0	0	94	1000	119	8[illegible]
Nagaland	0	11	114	675	167	0	0	33	1000	109	5[illegible]
Orissa	1	24	157	667	117	9	0	25	1000	13371	52[illegible]
Punjab	4	22	105	606	211	39	0	14	1000	6843	25[illegible]
Rajasthan	5	14	122	629	174	35	15	7	1000	12196	55[illegible]
Sikkim	0	8	57	590	297	17	0	31	1000	115	11[illegible]
Tamil Nadu	0	52	302	516	104	27	0	0	1000	16961	57[illegible]
Tripura	12	31	217	555	142	11	0	32	1000	927	17[illegible]
Uttaranchal	0	44	123	543	204	11	0	76	1000	2674	10[illegible]
Uttar Pradesh	3	31	115	549	224	45	3	29	1000	42788	182[illegible]
West Bengal	0	12	108	676	158	8	9	29	1000	18130	68[illegible]
A & N Islands	0	3	40	697	257	4	0	0	1000	76	3[illegible]
Chandigarh	0	51	409	349	117	74	0	0	1000	7	1[illegible]
Dadra & N. Haveli	0	0	67	801	132	0	0	0	1000	78	2[illegible]
Daman & Diu	0	73	158	725	0	44	0	0	1000	4	[illegible]
Lakshadweep	0	0	29	574	237	160	0	0	1000	7	1[illegible]
Pondicherry	0	60	358	451	130	0	0	0	1000	160	2[illegible]

Continued

For peer review only - http://bmjopen.bmj.com/site/about/guidelines.xhtml

Appendix A

Table (14): Per 1000 distribution of aged persons by type of living arrangement for each sex and State/UT

ural Males

tate/ut	living arrangement of aged persons									no. of aged persons	
	living alone		with spouse only	with spouse & other members	without spouse but with			n.r.	total	estd. (00)	sam-ple
	as an inmate of old age home	not as an inmate of old age home			children	other rela-tions	non-rela-tions				
1)	(2)	(3)	(4)	(5)	(6)	(7)	(8)	(9)	(10)	(11)	(12
NES	7	26	130	589	155	7	2	86	1000	2371	87[illegible]
UTs	0	32	208	593	160	6	0	0	1000	333	11[illegible]
ll-India	3	25	162	597	168	24	3	18	1000	253254	1152[illegible]
std. no. of aged persons (00)	866	6232	40931	151219	42647	6198	718	4443	253254	X	[illegible]
ample aged persons	41	260	1758	6940	1971	284	36	238	11528	X	[illegible]

For peer review only - http://bmjopen.bmj.com/site/about/guidelines.xhtml

Appendix A

Table (14): Per 1000 distribution of aged persons by type of living arrangement for each sex and State/UT

Rural — Females

state/ut	living arrangement of aged persons: living alone: as an inmate of old age home	living alone: not as an inmate of old age home	with spouse only	with spouse & other members	without spouse but with: children	without spouse but with: other relations	without spouse but with: non-relations	n.r.	total	no. of aged persons: estd. (00)	no. of aged persons: sample
(1)	(2)	(3)	(4)	(5)	(6)	(7)	(8)	(9)	(10)	(11)	(12)
Andhra Pradesh	5	139	134	151	478	80	2	11	1000	20035	68[illegible]
Arunachal Pradesh	0	50	18	338	403	24	0	167	1000	204	11[illegible]
Assam	0	64	32	212	626	14	0	53	1000	4273	32[illegible]
Bihar	15	38	90	390	416	14	7	30	1000	15258	67[illegible]
Chhattisgarh	43	75	87	212	498	48	15	22	1000	6680	29[illegible]
Delhi	0	39	0	641	235	85	0	0	1000	322	1[illegible]
Goa	0	5	127	329	371	168	0	0	1000	615	3[illegible]
Gujarat	3	85	113	282	494	21	0	1	1000	10401	37[illegible]
Haryana	0	8	56	519	383	20	14	0	1000	5510	23[illegible]
Himachal Pradesh	3	37	72	240	532	84	0	32	1000	2546	34[illegible]
Jammu & Kashmir	3	6	36	435	486	27	0	7	1000	1555	17[illegible]
Jharkhand	14	34	60	360	496	16	0	20	1000	4441	23[illegible]
Karnataka	0	97	65	195	552	80	1	9	1000	10878	40[illegible]
Kerala	0	42	56	281	539	60	7	15	1000	15013	64[illegible]
Madhya Pradesh	21	62	129	294	408	55	3	28	1000	14701	54[illegible]
Maharashtra	11	94	97	301	415	67	6	9	1000	24333	71[illegible]
Manipur	0	11	16	297	482	36	0	157	1000	279	16[illegible]
Meghalaya	0	42	88	247	547	11	0	65	1000	431	9[illegible]
Mizoram	0	0	5	392	429	8	18	149	1000	76	4[illegible]
Nagaland	0	0	164	560	276	0	0	0	1000	39	2[illegible]
Orissa	4	40	77	300	504	34	9	31	1000	12157	48[illegible]
Punjab	0	37	93	406	443	19	0	2	1000	6518	23[illegible]
Rajasthan	6	39	78	312	523	37	5	0	1000	12999	58[illegible]
Sikkim	25	0	48	344	450	57	0	76	1000	109	9[illegible]
Tamil Nadu	0	194	110	204	391	89	11	0	1000	17146	54[illegible]
Tripura	17	115	86	154	521	78	13	16	1000	675	13[illegible]
Uttaranchal	8	180	86	286	399	20	10	10	1000	2190	9[illegible]
Uttar Pradesh	7	44	85	343	445	39	10	27	1000	41298	164[illegible]
West Bengal	2	55	43	174	624	54	7	40	1000	18472	68[illegible]
A & N Islands	0	106	10	128	489	110	0	156	1000	43	1[illegible]
Chandigarh	0	0	54	264	682	0	0	0	1000	8	1[illegible]
Dadra & N. Haveli	0	40	57	382	521	0	0	0	1000	39	1[illegible]
Daman & Diu	0	381	13	97	509	0	0	0	1000	13	1[illegible]
Lakshadweep	0	45	0	120	739	96	0	0	1000	9	2[illegible]
Pondicherry	0	64	65	143	674	55	0	0	1000	148	1[illegible]

Continued

For peer review only - http://bmjopen.bmj.com/site/about/guidelines.xhtml

Appendix A

Table (14): Per 1000 distribution of aged persons by type of living arrangement for each sex and State/UT

Rural — Females

tate/ut	living arrangement of aged persons									no. of aged persons	
	living alone		with spouse only	with spouse & other members	without spouse but with			n.r.	total		
	as an inmate of old age home	not as an inmate of old age home			children	other rela-tions	non-rela-tions			estd. (00)	sam-ple
1)	(2)	(3)	(4)	(5)	(6)	(7)	(8)	(9)	(10)	(11)	(12
NES	8	60	64	249	494	44	6	75	1000	1813	67
UTs	0	81	50	177	614	53	0	26	1000	261	8
ll-India	7	73	87	284	475	50	6	19	1000	249416	1073
std. no. of aged ersons (00)	1768	18134	21731	70710	118353	12487	1607	4624	249416	X	[illegible]
ample aged ersons	65	662	959	3180	5077	500	49	245	10737	X	[illegible]

For peer review only - http://bmjopen.bmj.com/site/about/guidelines.xhtml

For peer review only

Appendix A

Table (14): Per 1000 distribution of aged persons by type of living arrangement for each sex and State/UT

Rural — Persons

state/ut	living arrangement of aged persons									no. of aged persons	
	living alone		with spouse only	with spouse & other members	without spouse but with			n.r.	total	estd. (00)	sam-ple
	as an inmate of old age home	not as an inmate of old age home			children	other rela-tions	non-rela-tions				
(1)	(2)	(3)	(4)	(5)	(6)	(7)	(8)	(9)	(10)	(11)	(12)
Andhra Pradesh	3	89	226	309	314	47	1	11	1000	38765	1385
Arunachal Pradesh	0	34	32	457	253	11	0	212	1000	442	258
Assam	0	28	38	518	355	11	1	50	1000	10376	794
Bihar	13	27	123	506	293	10	4	24	1000	34119	1590
Chhattisgarh	35	50	125	334	378	34	18	26	1000	11289	494
Delhi	0	19	0	701	173	107	0	0	1000	661	33
Goa	0	3	192	477	216	113	0	0	1000	1121	51
Gujarat	2	62	148	417	350	17	0	3	1000	20859	721
Haryana	0	4	63	608	295	21	7	1	1000	11118	484
Himachal Pradesh	3	39	101	413	362	51	6	26	1000	4946	693
Jammu & Kashmir	1	2	36	591	322	30	1	16	1000	3801	403
Jharkhand	6	30	111	465	344	26	1	18	1000	10841	561
Karnataka	1	57	103	448	334	52	1	5	1000	22520	831
Kerala	0	29	87	463	363	44	5	9	1000	26687	1154
Madhya Pradesh	16	41	160	419	285	49	3	26	1000	29367	1093
Maharashtra	6	58	139	469	272	44	3	7	1000	47679	1433
Manipur	8	17	41	426	304	20	6	177	1000	673	374
Meghalaya	0	37	98	483	322	6	0	54	1000	898	191
Mizoram	0	6	20	538	311	3	7	115	1000	196	137
Nagaland	0	8	127	645	196	0	0	24	1000	148	80
Orissa	2	32	119	492	301	21	4	28	1000	25527	1008
Punjab	2	29	99	509	324	29	0	8	1000	13361	497
Rajasthan	6	27	99	465	354	36	10	3	1000	25196	1135
Sikkim	12	4	53	470	371	36	0	53	1000	224	204
Tamil Nadu	0	123	205	359	248	58	6	0	1000	34107	1120
Tripura	14	67	162	386	302	39	6	25	1000	1602	306
Uttaranchal	4	105	106	427	292	15	5	46	1000	4865	192
Uttar Pradesh	5	37	101	448	333	42	6	28	1000	84086	3474
West Bengal	1	34	75	423	393	31	8	34	1000	36602	1366
A & N Islands	0	40	29	491	341	42	0	56	1000	120	49
Chandigarh	0	24	219	304	419	35	0	0	1000	16	28
Dadra & N. Haveli	0	13	63	662	261	0	0	0	1000	117	30
Daman & Diu	0	309	47	243	390	10	0	0	1000	17	20
Lakshadweep	0	25	13	322	516	124	0	0	1000	16	38
Pondicherry	0	62	217	303	391	26	0	0	1000	309	38

Continued

For peer review only

For peer review only - http://bmjopen.bmj.com/site/about/guidelines.xhtml

Appendix A

Table (14): Per 1000 distribution of aged persons by type of living arrangement for each sex and State/UT

Rural Persons

state/ut	living arrangement of aged persons									no. of aged persons	
	living alone		with spouse only	with spouse & other members	without spouse but with			n.r.	total	estd. (00)	sam-ple
	as an inmate of old age home	not as an inmate of old age home			children	other rela-tions	non-rela-tions				
(1)	(2)	(3)	(4)	(5)	(6)	(7)	(8)	(9)	(10)	(11)	(12)
ONES	7	41	101	441	302	23	3	81	1000	4184	1550
UTs	0	53	139	410	360	27	0	11	1000	594	20[illegible]
All-India	5	48	125	442	320	37	5	18	1000	502670	22265
estd. no. of aged persons (00)	2634	24366	62662	221929	161000	18685	2325	9067	502670	X	X
sample aged persons	106	922	2717	10120	7048	784	85	483	22265	X	X

For peer review only - http://bmjopen.bmj.com/site/about/guidelines.xhtml

For peer review only

Appendix A

Table (14): Per 1000 distribution of aged persons by type of living arrangement for each sex and State/UT

Urban Males

state/ut	living arrangement of aged persons									no. of aged persons	
	living alone		with spouse only	with spouse & other members	without spouse but with			n.r.	total	estd. (00)	sam-ple
	as an inmate of old age home	not as an inmate of old age home			children	other rela-tions	non-rela-tions				
(1)	(2)	(3)	(4)	(5)	(6)	(7)	(8)	(9)	(10)	(11)	(12)
Andhra Pradesh	7	20	215	579	150	24	1	3	1000	5310	37[illegible]
Arunachal Pradesh	0	25	57	281	324	41	0	270	1000	10	4[illegible]
Assam	10	0	25	800	141	18	3	2	1000	647	10[illegible]
Bihar	0	7	55	610	248	3	8	69	1000	2455	16[illegible]
Chhattisgarh	0	12	37	711	240	0	0	0	1000	641	6[illegible]
Delhi	2	16	125	646	188	6	4	14	1000	2212	21[illegible]
Goa	0	49	54	683	96	119	0	0	1000	229	3[illegible]
Gujarat	0	9	139	710	136	7	0	0	1000	4592	29[illegible]
Haryana	0	0	137	663	180	0	13	6	1000	1315	13[illegible]
Himachal Pradesh	0	0	149	634	168	49	0	0	1000	139	4[illegible]
Jammu & Kashmir	0	4	133	655	183	3	16	7	1000	408	10[illegible]
Jharkhand	6	37	44	712	159	5	0	37	1000	1372	16[illegible]
Karnataka	0	8	96	729	141	16	0	9	1000	4025	34[illegible]
Kerala	0	7	168	695	93	30	6	1	1000	3986	29[illegible]
Madhya Pradesh	10	10	103	649	169	35	3	21	1000	4115	31[illegible]
Maharashtra	0	17	130	653	176	13	2	8	1000	12844	59[illegible]
Manipur	2	0	66	703	75	48	0	105	1000	192	11[illegible]
Meghalaya	0	0	56	775	141	0	27	0	1000	42	3[illegible]
Mizoram	0	6	51	537	164	29	0	213	1000	85	14[illegible]
Nagaland	0	0	0	982	18	0	0	0	1000	68	2[illegible]
Orissa	0	12	166	650	133	9	3	28	1000	1546	14[illegible]
Punjab	6	15	96	732	109	15	2	25	1000	2565	16[illegible]
Rajasthan	2	22	112	643	190	19	10	2	1000	3012	27[illegible]
Sikkim	0	0	0	735	97	142	26	0	1000	11	1[illegible]
Tamil Nadu	8	27	221	587	121	33	3	0	1000	7955	58[illegible]
Tripura	0	40	122	786	52	0	0	0	1000	130	4[illegible]
Uttaranchal	0	0	200	586	202	13	0	0	1000	558	4[illegible]
Uttar Pradesh	0	33	112	601	203	16	11	24	1000	9104	62[illegible]
West Bengal	0	27	106	664	102	72	8	21	1000	8725	52[illegible]
A & N Islands	0	0	0	750	183	0	0	67	1000	18	1[illegible]
Chandigarh	9	21	168	593	137	61	11	0	1000	197	6[illegible]
Dadra & N. Haveli	0	0	131	668	201	0	0	0	1000	4	1[illegible]
Daman & Diu	0	94	74	648	118	67	0	0	1000	10	1[illegible]
Lakshadweep	0	0	103	620	138	140	0	0	1000	8	2[illegible]
Pondicherry	0	13	195	625	132	35	0	0	1000	218	4[illegible]

Continued

For peer review only - http://bmjopen.bmj.com/site/about/guidelines.xhtml

For peer review only

Appendix A

Table (14): Per 1000 distribution of aged persons by type of living arrangement for each sex and State/UT

Urban Males

state/ut	living arrangement of aged persons									no. of aged persons	
	living alone		with spouse only	with spouse & other members	without spouse but with			n.r.	total		
	as an inmate of old age home	not as an inmate of old age home			children	other relations	non-relations			estd. (00)	sample
(1)	(2)	(3)	(4)	(5)	(6)	(7)	(8)	(9)	(10)	(11)	(12)
ONES	1	11	66	731	87	25	3	76	1000	538	419
GUTs	4	17	171	617	137	47	5	3	1000	455	184
All-India	2	19	133	649	154	24	5	14	1000	78751	6222
estd. no. of aged persons (00)	187	1484	10479	51120	12138	1911	365	1067	78751	X	X
sample aged persons	15	121	741	4102	930	169	34	110	6222	X	X

For peer review only - http://bmjopen.bmj.com/site/about/guidelines.xhtml

Appendix A

Table (14): Per 1000 distribution of aged persons by type of living arrangement for each sex and State/UT

Urban Females

state/ut	living arrangement of aged persons									no. of aged persons	
	living alone		with spouse only	with spouse & other members	without spouse but with			n.r.	total	estd. (00)	sam-ple
	as an inmate of old age home	not as an inmate of old age home			children	other rela-tions	non-rela-tions				
(1)	(2)	(3)	(4)	(5)	(6)	(7)	(8)	(9)	(10)	(11)	(12)
Andhra Pradesh	5	86	95	194	537	66	1	16	1000	6160	42[illegible]
Arunachal Pradesh	50	20	77	415	331	0	0	107	1000	7	3[illegible]
Assam	0	0	0	328	544	17	13	98	1000	532	8[illegible]
Bihar	14	33	87	431	359	27	4	43	1000	1829	14[illegible]
Chhattisgarh	33	135	11	247	469	105	0	0	1000	760	8[illegible]
Delhi	0	59	83	362	463	19	2	11	1000	2129	20[illegible]
Goa	0	177	48	235	423	116	0	0	1000	256	4[illegible]
Gujarat	0	70	89	301	487	44	0	9	1000	5101	31[illegible]
Haryana	3	58	87	343	459	15	2	32	1000	1725	14[illegible]
Himachal Pradesh	0	0	61	236	680	14	0	10	1000	155	5[illegible]
Jammu & Kashmir	0	24	117	325	522	0	0	12	1000	336	9[illegible]
Jharkhand	3	28	39	365	482	21	8	54	1000	1292	13[illegible]
Karnataka	0	72	31	209	612	65	6	5	1000	4073	35[illegible]
Kerala	0	59	64	201	539	116	5	16	1000	4686	32[illegible]
Madhya Pradesh	8	57	52	313	462	75	1	33	1000	4466	32[illegible]
Maharashtra	2	65	83	303	482	49	4	10	1000	13669	67[illegible]
Manipur	0	49	0	348	428	42	0	134	1000	178	11[illegible]
Meghalaya	0	69	50	82	782	7	0	9	1000	48	4[illegible]
Mizoram	0	16	7	348	363	162	0	105	1000	60	10[illegible]
Nagaland	0	0	0	888	112	0	0	0	1000	8	[illegible]
Orissa	7	31	59	388	434	32	8	40	1000	1182	11[illegible]
Punjab	0	25	68	497	387	13	0	10	1000	2347	16[illegible]
Rajasthan	0	61	81	353	468	36	0	0	1000	3076	26[illegible]
Sikkim	0	0	0	359	641	0	0	0	1000	7	1[illegible]
Tamil Nadu	0	120	99	199	480	97	3	0	1000	8984	65[illegible]
Tripura	0	39	31	364	478	73	0	16	1000	136	5[illegible]
Uttaranchal	0	43	98	236	582	10	0	32	1000	693	4[illegible]
Uttar Pradesh	13	21	79	363	440	54	9	21	1000	9590	62[illegible]
West Bengal	3	34	61	308	462	97	16	18	1000	8315	50[illegible]
A & N Islands	0	12	0	502	342	95	0	49	1000	15	2[illegible]
Chandigarh	0	96	206	288	384	8	8	10	1000	147	6[illegible]
Dadra & N. Haveli	0	0	22	115	770	94	0	0	1000	3	1[illegible]
Daman & Diu	0	180	0	183	595	0	0	42	1000	16	2[illegible]
Lakshadweep	0	74	81	141	606	98	0	0	1000	10	2[illegible]
Pondicherry	0	87	67	227	598	22	0	0	1000	368	5[illegible]

Continued

For peer review only - http://bmjopen.bmj.com/site/about/guidelines.xhtml

Appendix A

[illegible]able (14): Per 1000 distribution of aged persons by type of living arrangement for each sex and State/UT

[illegible]rban Female[illegible]

[illegible]tate/ut	living arrangement of aged persons									no. of aged persons	
	living alone		with spouse only	with spouse & other members	without spouse but with			n.r.	total		
	as an inmate of old age home	not as an inmate of old age home			children	other rela-tions	non-rela-tions			estd. (00)	sam-ple
[illegible]1)	(2)	(3)	(4)	(5)	(6)	(7)	(8)	(9)	(10)	(11)	(12[illegible]
[illegible]NES	1	42	17	335	469	62	0	75	1000	444	36[illegible]
[illegible]UTs	0	89	100	247	536	22	2	5	1000	560	20[illegible]
[illegible]ll-India	4	61	75	294	482	62	5	16	1000	82359	634[illegible]
[illegible]std. no. of aged [illegible]ersons (00)	324	5033	6208	24252	39710	5123	412	1297	82359	X	[illegible]
[illegible]ample aged [illegible]ersons	19	326	417	1905	3148	368	27	134	6344	X	[illegible]

For peer review only - http://bmjopen.bmj.com/site/about/guidelines.xhtml

For peer review only

Appendix A

Table (14): Per 1000 distribution of aged persons by type of living arrangement for each sex and State/UT

Urban Persons

state/ut	living arrangement of aged persons									no. of aged persons	
	living alone		with spouse only	with spouse & other members	without spouse but with			n.r.	total	estd. (00)	sam-ple
	as an inmate of old age home	not as an inmate of old age home			children	other rela-tions	non-rela-tions				
(1)	(2)	(3)	(4)	(5)	(6)	(7)	(8)	(9)	(10)	(11)	(12)
Andhra Pradesh	6	56	151	372	358	47	1	10	1000	11470	798
Arunachal Pradesh	21	23	65	336	327	24	0	203	1000	17	72
Assam	6	0	14	587	323	18	7	45	1000	1180	194
Bihar	6	18	69	534	295	13	6	58	1000	4283	314
Chhattisgarh	18	79	23	459	364	57	0	0	1000	1401	151
Delhi	1	37	105	507	323	13	3	12	1000	4342	418
Goa	0	116	51	446	269	118	0	0	1000	485	72
Gujarat	0	41	112	495	321	27	0	5	1000	9693	616
Haryana	2	33	109	482	338	9	7	21	1000	3040	275
Himachal Pradesh	0	0	103	425	437	30	0	5	1000	294	99
Jammu & Kashmir	0	13	126	506	336	1	9	9	1000	744	193
Jharkhand	4	33	41	544	316	13	4	45	1000	2664	305
Karnataka	0	40	63	468	378	41	3	7	1000	8098	698
Kerala	0	35	112	428	334	76	6	9	1000	8672	612
Madhya Pradesh	9	34	77	474	321	56	2	28	1000	8580	646
Maharashtra	1	42	106	473	334	31	3	9	1000	26513	1268
Manipur	1	24	34	532	245	45	0	119	1000	369	231
Meghalaya	0	37	53	408	480	4	13	5	1000	90	80
Mizoram	0	10	32	458	247	84	0	168	1000	145	246
Nagaland	0	0	0	973	27	0	0	0	1000	76	29
Orissa	3	20	120	536	263	19	5	33	1000	2729	261
Punjab	3	20	83	619	242	14	1	18	1000	4912	329
Rajasthan	1	42	96	497	331	28	5	1	1000	6088	537
Sikkim	0	0	0	584	315	85	16	0	1000	18	29
Tamil Nadu	4	77	157	381	312	67	3	0	1000	16939	1237
Tripura	0	39	75	570	270	37	0	8	1000	267	99
Uttaranchal	0	24	143	392	412	11	0	18	1000	1252	98
Uttar Pradesh	7	27	95	479	325	35	10	22	1000	18694	1241
West Bengal	2	31	84	490	278	84	12	20	1000	17040	1028
A & N Islands	0	6	0	637	255	43	0	59	1000	33	40
Chandigarh	5	53	184	463	243	38	10	4	1000	344	137
Dadra & N. Haveli	0	0	79	407	469	44	0	0	1000	7	27
Daman & Diu	0	147	28	362	412	26	0	26	1000	26	38
Lakshadweep	0	41	91	352	399	116	0	0	1000	19	45
Pondicherry	0	59	114	375	424	27	0	0	1000	586	103

Continued

For peer review only - http://bmjopen.bmj.com/site/about/guidelines.xhtml

Appendix A

Table (14): Per 1000 distribution of aged persons by type of living arrangement for each sex and State/UT

Urban Persons

state/ut	living arrangement of aged persons									no. of aged persons	
	living alone		with spouse only	with spouse & other members	without spouse but with			n.r.	total		
	as an inmate of old age home	not as an inmate of old age home			children	other relations	non-relations			estd. (00)	sample
(1)	(2)	(3)	(4)	(5)	(6)	(7)	(8)	(9)	(10)	(11)	(12)
ONES	1	25	44	552	259	42	1	76	1000	983	78[illegible]
UTs	2	57	131	413	357	33	3	4	1000	1015	39[illegible]
All-India	3	40	104	468	322	44	5	15	1000	161110	1256[illegible]
estd. no. of aged persons (00)	511	6517	16687	75372	51848	7034	777	2365	161110	X	X
sample aged persons	34	447	1158	6007	4078	537	61	244	12566	X	X

For peer review only - http://bmjopen.bmj.com/site/about/guidelines.xhtml

Appendix A

Table (14): Per 1000 distribution of aged persons by type of living arrangement for each sex and State/UT

Rural+Urban Males

state/ut	living arrangement of aged persons									no. of aged persons	
	living alone		with spouse only	with spouse & other members	without spouse but with			n.r.	total	estd. (00)	sam-ple
	as an inmate of old age home	not as an inmate of old age home			children	other rela-tions	non-rela-tions				
(1)	(2)	(3)	(4)	(5)	(6)	(7)	(8)	(9)	(10)	(11)	(12
Andhra Pradesh	3	32	300	501	141	14	1	8	1000	24041	1074
Arunachal Pradesh	0	21	45	548	133	2	0	251	1000	248	182
Assam	1	3	40	739	163	10	1	43	1000	6750	580
Bihar	11	17	138	600	199	7	3	25	1000	21316	1081
Chhattisgarh	21	13	164	535	208	11	19	29	1000	5250	267
Delhi	1	14	109	661	178	23	3	12	1000	2551	227
Goa	0	15	203	665	49	69	0	0	1000	735	51
Gujarat	0	31	169	600	185	12	0	4	1000	15050	648
Haryana	0	0	83	690	203	18	3	2	1000	6923	384
Himachal Pradesh	2	38	133	598	180	18	12	18	1000	2539	390
Jammu & Kashmir	0	1	50	692	204	28	4	20	1000	2654	332
Jharkhand	2	29	128	568	224	28	1	20	1000	7772	490
Karnataka	1	16	128	696	132	24	0	3	1000	15667	773
Kerala	0	11	137	697	126	24	2	2	1000	15659	795
Madhya Pradesh	12	18	171	568	163	42	3	24	1000	18781	868
Maharashtra	1	20	165	647	142	18	1	6	1000	36191	1313
Manipur	10	14	61	577	145	22	7	163	1000	587	327
Meghalaya	0	29	103	706	117	1	2	40	1000	510	130
Mizoram	0	8	38	592	206	12	0	143	1000	204	231
Nagaland	0	7	70	793	109	0	0	20	1000	178	80
Orissa	1	23	158	665	119	9	0	25	1000	14917	673
Punjab	5	20	102	641	183	32	1	17	1000	9408	423
Rajasthan	4	16	120	632	177	32	14	6	1000	15208	823
Sikkim	0	8	52	602	280	28	2	28	1000	126	120
Tamil Nadu	3	44	276	539	109	29	1	0	1000	24916	1158
Tripura	10	32	206	583	131	9	0	28	1000	1057	210
Uttaranchal	0	36	136	550	203	11	0	63	1000	3232	151
Uttar Pradesh	3	31	115	558	221	40	4	28	1000	51892	2445
West Bengal	0	17	107	672	140	29	9	26	1000	26855	1202
A & N Islands	0	2	32	707	243	3	0	13	1000	94	52
Chandigarh	9	22	177	584	137	61	11	0	1000	205	80
Dadra & N. Haveli	0	0	70	795	136	0	0	0	1000	82	33
Daman & Diu	0	88	98	670	84	60	0	0	1000	14	23
Lakshadweep	0	0	69	599	184	149	0	0	1000	15	37
Pondicherry	0	33	264	551	131	20	0	0	1000	379	67

Continued

For peer review only - http://bmjopen.bmj.com/site/about/guidelines.xhtml

Appendix A

Table (14): Per 1000 distribution of aged persons by type of living arrangement for each sex and State/UT

Rural+Urban | Males

state/ut	living arrangement of aged persons									no. of aged persons	
	living alone		with spouse only	with spouse & other members	without spouse but with			n.r.	total		
	as an inmate of old age home	not as an inmate of old age home			children	other relations	non-relations			estd. (00)	sample
(1)	(2)	(3)	(4)	(5)	(6)	(7)	(8)	(9)	(10)	(11)	(12
ONES	6	23	118	615	142	10	2	84	1000	2909	129[illegible]
UTs	2	24	187	607	147	30	3	2	1000	789	29[illegible]
ll-India	3	23	155	609	165	24	3	17	1000	332005	1775[illegible]
estd. no. of aged persons (00)	1053	7716	51410	202339	54785	8110	1083	5510	332005	X	[illegible]
sample aged persons	56	381	2499	11042	2901	453	70	348	17750	X	[illegible]

For peer review only - http://bmjopen.bmj.com/site/about/guidelines.xhtml

Appendix A

Table (14): Per 1000 distribution of aged persons by type of living arrangement for each sex and State/UT

Rural+Urban Females

State/ut	living arrangement of aged persons									no. of aged persons	
	living alone		with spouse only	with spouse & other members	without spouse but with			n.r.	total	estd. (00)	sam-ple
	as an inmate of old age home	not as an inmate of old age home			children	other rela-tions	non-rela-tions				
(1)	(2)	(3)	(4)	(5)	(6)	(7)	(8)	(9)	(10)	(11)	(12
Andhra Pradesh	5	127	125	161	492	77	1	12	1000	26195	110[illegible]
Arunachal Pradesh	2	49	20	341	401	24	0	165	1000	211	14[illegible]
Assam	0	57	28	224	617	14	1	58	1000	4806	40[illegible]
Bihar	15	37	90	394	410	15	6	32	1000	17087	82[illegible]
Chhattisgarh	42	81	79	216	495	54	13	20	1000	7440	37[illegible]
Delhi	0	56	72	399	433	28	2	10	1000	2452	22[illegible]
Goa	0	55	104	301	387	152	0	0	1000	871	7[illegible]
Gujarat	2	80	105	289	492	29	0	3	1000	15501	68[illegible]
Haryana	1	20	63	477	402	19	11	8	1000	7235	37[illegible]
Himachal Pradesh	2	35	72	240	541	80	0	30	1000	2701	39[illegible]
Jammu & Kashmir	3	9	51	416	493	22	0	8	1000	1891	26[illegible]
Jharkhand	12	33	55	361	493	17	2	28	1000	5733	37[illegible]
Karnataka	0	91	56	199	569	76	3	8	1000	14951	75[illegible]
Kerala	0	46	58	262	539	73	7	15	1000	19699	97[illegible]
Madhya Pradesh	18	61	111	298	421	60	3	29	1000	19166	87[illegible]
Maharashtra	8	84	92	302	439	60	6	10	1000	38001	138[illegible]
Manipur	0	26	10	317	461	38	0	148	1000	456	27[illegible]
Meghalaya	0	44	84	231	570	11	0	60	1000	479	14[illegible]
Mizoram	0	7	6	372	400	76	10	129	1000	137	15[illegible]
Nagaland	0	0	137	614	249	0	0	0	1000	47	2[illegible]
Orissa	4	39	76	308	498	34	9	32	1000	13339	59[illegible]
Punjab	0	34	86	430	428	17	0	4	1000	8865	40[illegible]
Rajasthan	5	43	79	320	512	37	4	0	1000	16075	84[illegible]
Sikkim	24	0	45	345	462	53	0	72	1000	116	10[illegible]
Tamil Nadu	0	169	106	203	422	92	9	0	1000	26130	119[illegible]
Tripura	14	103	77	189	514	77	11	16	1000	812	18[illegible]
Uttaranchal	6	147	89	274	443	18	8	15	1000	2884	13[illegible]
Uttar Pradesh	8	39	84	347	444	42	10	26	1000	50887	227[illegible]
West Bengal	3	49	48	215	574	68	10	33	1000	26787	119[illegible]
A & N Islands	0	82	8	224	451	106	0	128	1000	58	3[illegible]
Chandigarh	0	91	198	287	400	8	8	10	1000	155	7[illegible]
Dadra & N. Haveli	0	37	54	361	541	8	0	0	1000	42	2[illegible]
Daman & Diu	0	272	6	144	555	0	0	23	1000	29	3[illegible]
Lakshadweep	0	60	44	131	668	97	0	0	1000	19	4[illegible]
Pondicherry	0	80	66	202	620	32	0	0	1000	516	7[illegible]

Continued

For peer review only - http://bmjopen.bmj.com/site/about/guidelines.xhtml

Appendix A

Table (14): Per 1000 distribution of aged persons by type of living arrangement for each sex and State/UT

Rural+Urban | Females

state/ut	living arrangement of aged persons									no. of aged persons	
	living alone		with spouse only	with spouse & other members	without spouse but with			n.r.	total		
	as an inmate of old age home	not as an inmate of old age home			children	other relations	non-relations			estd. (00)	sample
(1)	(2)	(3)	(4)	(5)	(6)	(7)	(8)	(9)	(10)	(11)	(12)
[illegible]NES	6	56	55	266	489	47	5	75	1000	2257	104[illegible]
[illegible]UTs	0	86	84	224	561	32	1	12	1000	821	29[illegible]
All-India	6	70	84	286	476	53	6	18	1000	331775	1708[illegible]
estd. no. of aged persons (00)	2092	23167	27939	94962	158064	17610	2019	5922	331775	X	X
sample aged persons	84	988	1376	5085	8225	868	76	379	17081	X	X

For peer review only - http://bmjopen.bmj.com/site/about/guidelines.xhtml

Appendix A

Table (14): Per 1000 distribution of aged persons by type of living arrangement for each sex and State/UT

Rural+Urban | Persons

State/ut	living arrangement of aged persons									no. of aged persons	
	living alone		with spouse only	with spouse & other members	without spouse but with			n.r.	total	estd. (00)	sam-ple
	as an inmate of old age home	not as an inmate of old age home			children	other rela-tions	non-rela-tions				
(1)	(2)	(3)	(4)	(5)	(6)	(7)	(8)	(9)	(10)	(11)	(12)
Andhra Pradesh	4	81	209	324	324	47	1	10	1000	50236	218[illegible]
Arunachal Pradesh	1	34	34	453	256	12	0	211	1000	460	33[illegible]
Assam	1	25	35	525	352	12	1	49	1000	11556	98[illegible]
Bihar	13	26	117	509	293	11	5	28	1000	38403	190[illegible]
Chhattisgarh	33	53	114	348	376	36	16	23	1000	12690	64[illegible]
Delhi	1	35	91	532	303	25	3	11	1000	5002	45[illegible]
Goa	0	37	149	468	232	114	0	0	1000	1606	12[illegible]
Gujarat	1	56	137	442	341	20	0	4	1000	30551	133[illegible]
Haryana	0	10	73	581	305	18	7	5	1000	14158	75[illegible]
Himachal Pradesh	2	36	102	413	366	50	6	24	1000	5240	79[illegible]
Jammu & Kashmir	1	4	50	577	324	25	2	15	1000	4545	59[illegible]
Jharkhand	6	31	97	480	338	23	1	23	1000	13505	86[illegible]
Karnataka	1	52	92	454	345	49	1	5	1000	30618	152[illegible]
Kerala	0	30	93	455	356	52	5	9	1000	35359	176[illegible]
Madhya Pradesh	15	40	141	432	293	51	3	27	1000	37947	173[illegible]
Maharashtra	4	53	127	470	294	40	3	8	1000	74193	270[illegible]
Manipur	6	19	39	463	283	29	4	157	1000	1043	60[illegible]
Meghalaya	0	37	94	476	337	6	1	49	1000	988	27[illegible]
Mizoram	0	8	25	504	284	38	4	138	1000	341	38[illegible]
Nagaland	0	6	84	756	138	0	0	16	1000	224	10[illegible]
Orissa	2	31	119	496	298	21	4	28	1000	28256	126[illegible]
Punjab	2	27	95	539	302	25	0	11	1000	18274	82[illegible]
Rajasthan	5	30	99	471	349	34	9	3	1000	31284	167[illegible]
Sikkim	11	4	49	479	367	40	1	49	1000	242	23[illegible]
Tamil Nadu	1	108	189	367	269	61	5	0	1000	51046	235[illegible]
Tripura	12	63	150	412	297	39	5	23	1000	1869	40[illegible]
Uttaranchal	3	89	114	420	316	14	4	40	1000	6116	29[illegible]
Uttar Pradesh	5	35	100	453	331	41	7	27	1000	102779	471[illegible]
West Bengal	1	33	78	444	356	48	9	30	1000	53642	239[illegible]
A & N Islands	0	33	23	523	322	42	0	57	1000	152	8[illegible]
Chandigarh	5	52	186	456	250	38	10	4	1000	360	16[illegible]
Dadra & N. Haveli	0	13	64	647	273	3	0	0	1000	124	5[illegible]
Daman & Diu	0	213	36	314	403	19	0	15	1000	43	5[illegible]
Lakshadweep	0	34	55	338	454	120	0	0	1000	35	8[illegible]
Pondicherry	0	60	150	350	413	27	0	0	1000	895	14[illegible]

Continued

For peer review only - http://bmjopen.bmj.com/site/about/guidelines.xhtml

Appendix A

[illegible]able (14): Per 1000 distribution of aged persons by type of living arrangement for each sex and State/UT

[illegible]ural+Urban Person[illegible]

[illegible]tate/ut	living arrangement of aged persons									no. of aged persons	
	living alone		with spouse only	with spouse & other members	without spouse but with			n.r.	total		
	as an inmate of old age home	not as an inmate of old age home			children	other relations	non-relations			estd. (00)	sam-ple
[illegible]1)	(2)	(3)	(4)	(5)	(6)	(7)	(8)	(9)	(10)	(11)	(12[illegible]
[illegible]NES	6	38	91	462	294	26	3	80	1000	5167	233[illegible]
[illegible]UTs	1	56	134	412	358	31	2	7	1000	1609	59[illegible]
[illegible]ll-India	5	47	120	448	321	39	5	17	1000	663779	3483[illegible]
[illegible]std. no. of aged [illegible]ersons (00)	3145	30883	79349	297301	212848	25719	3102	11432	663779	X	[illegible]
[illegible]ample aged [illegible]ersons	140	1369	3875	16127	11126	1321	146	727	34831	X	[illegible]

For peer review only - http://bmjopen.bmj.com/site/about/guidelines.xhtml

Appendix A

Table (15): Per 1000 distribution of aged persons living alone or living with spouse only by location of residence of any child/ grandchild or sibling for each sex

Rural Males

state/ut	location of residence of child/grandchild/sibling						no. of aged persons living alone or with spouse only	
	within the same building	within the village/ town	outside village/town	not applicable	n.r.	total	estd. (00)	sample
(1)	(2)	(3)	(4)	(5)	(6)	(7)	(8)	(9)
Andhra Pradesh	220	438	239	89	14	1000	6766	239
Arunachal Pradesh	288	208	150	79	274	1000	16	12
Assam	122	311	391	0	175	1000	274	30
Bihar	242	310	178	136	134	1000	3388	192
Chhattisgarh	211	412	150	223	4	1000	1009	47
Delhi	0	0	0	0	0	0	0	0
Goa	0	0	1000	0	0	1000	137	3
Gujarat	160	377	382	80	0	1000	2328	86
Haryana	226	481	207	86	0	1000	395	20
Himachal Pradesh	239	210	337	200	14	1000	420	58
Jammu & Kashmir	54	736	118	93	0	1000	80	12
Jharkhand	162	523	147	135	32	1000	1115	75
Karnataka	127	312	442	120	0	1000	1849	55
Kerala	68	331	392	209	0	1000	1624	65
Madhya Pradesh	189	479	200	125	7	1000	3266	123
Maharashtra	295	354	256	87	8	1000	4831	136
Manipur	464	217	93	160	66	1000	37	18
Meghalaya	28	350	128	336	159	1000	65	15
Mizoram	0	891	0	109	0	1000	5	4
Nagaland	0	708	292	0	0	1000	14	8
Orissa	213	270	262	175	79	1000	2437	97
Punjab	346	312	299	42	0	1000	896	29
Rajasthan	214	354	311	121	0	1000	1715	87
Sikkim	0	673	197	129	0	1000	8	9
Tamil Nadu	131	477	344	48	0	1000	5993	194
Tripura	640	108	77	153	21	1000	241	45
Uttaranchal	203	65	612	120	0	1000	447	20
Uttar Pradesh	175	315	237	169	104	1000	6407	270
West Bengal	364	271	199	150	17	1000	2185	87
A & N Islands	0	0	1000	0	0	1000	3	3
Chandigarh	76	817	0	108	0	1000	3	6
Dadra & N. Haveli	422	578	0	0	0	1000	5	3
Daman & Diu	188	495	0	317	0	1000	1	3
Lakshadweep	0	0	1000	0	0	1000	0	1
Pondicherry	52	316	487	144	0	1000	67	7
ONES	462	206	99	175	58	1000	385	111
GUTs	77	343	452	129	0	1000	80	23
all-India	205	372	272	117	34	1000	48029	2059
estd. no. of aged living alone or with spouse only (00)	9862	17850	13047	5640	1630	48029	X	X
sample aged living alone or with spouse only	444	747	531	258	79	2059	X	X

For peer review only - http://bmjopen.bmj.com/site/about/guidelines.xhtml

Appendix A

Table (15): Per 1000 distribution of aged persons living alone or living with spouse only by location of residence of any child/ grandchild or sibling for each sex

Rural Females

state/ut	location of residence of child/grandchild/sibling						no. of aged persons living alone or with spouse only	
	within the same building	within the village/ town	outside village/town	not applicable	n.r.	total	estd. (00)	sample
(1)	(2)	(3)	(4)	(5)	(6)	(7)	(8)	(9)
Andhra Pradesh	148	516	262	61	13	1000	5576	177
Arunachal Pradesh	709	100	133	58	0	1000	14	10
Assam	227	267	292	55	158	1000	408	38
Bihar	214	229	270	160	128	1000	2183	118
Chhattisgarh	186	446	144	197	27	1000	1370	57
Delhi	0	1000	0	0	0	1000	13	1
Goa	37	0	963	0	0	1000	81	3
Gujarat	145	399	324	132	0	1000	2093	80
Haryana	253	370	304	72	0	1000	353	19
Himachal Pradesh	227	232	408	132	0	1000	285	45
Jammu & Kashmir	257	608	135	0	0	1000	70	11
Jharkhand	88	386	249	234	44	1000	481	36
Karnataka	180	288	371	162	0	1000	1767	60
Kerala	31	468	280	198	23	1000	1466	64
Madhya Pradesh	245	416	217	123	0	1000	3109	110
Maharashtra	198	314	335	145	8	1000	4895	124
Manipur	157	644	0	199	0	1000	7	6
Meghalaya	88	352	0	93	467	1000	56	11
Mizoram	0	0	0	0	1000	1000	0	1
Nagaland	0	636	364	0	0	1000	6	3
Orissa	200	380	273	72	75	1000	1477	63
Punjab	299	217	484	0	0	1000	844	27
Rajasthan	163	454	247	135	0	1000	1610	76
Sikkim	0	528	423	50	0	1000	8	6
Tamil Nadu	53	585	314	48	0	1000	5211	166
Tripura	533	161	168	138	0	1000	147	24
Uttaranchal	30	301	625	45	0	1000	601	23
Uttar Pradesh	182	295	276	125	122	1000	5610	231
West Bengal	293	417	194	73	23	1000	1858	79
A & N Islands	0	0	1000	0	0	1000	5	2
Chandigarh	0	1000	0	0	0	1000	0	2
Dadra & N. Haveli	583	0	0	417	0	1000	4	2
Daman & Diu	141	859	0	0	0	1000	5	6
Lakshadweep	0	1000	0	0	0	1000	0	2
Pondicherry	0	0	494	506	0	1000	19	3
ONES	394	242	135	118	111	1000	239	61
GUTs	86	158	426	330	0	1000	34	17
all-India	169	398	289	110	34	1000	41633	1686
estd. no. of aged living alone or with spouse only (00)	7020	16587	12032	4585	1409	41633	X	X
sample aged living alone or with spouse only	323	651	458	190	64	1686	X	X

For peer review only - http://bmjopen.bmj.com/site/about/guidelines.xhtml

Appendix A

Table (15): Per 1000 distribution of aged persons living alone or living with spouse only by location of residence of any child/ grandchild or sibling for each sex

Rural Persons

state/ut	location of residence of child/grandchild/sibling						no. of aged persons living alone or with spouse only	
	within the same building	within the village/ town	outside village/town	not applicable	n.r.	total	estd. (00)	sample
(1)	(2)	(3)	(4)	(5)	(6)	(7)	(8)	(9)
Andhra Pradesh	188	473	249	76	14	1000	12342	416
Arunachal Pradesh	485	157	142	69	145	1000	29	22
Assam	185	285	332	33	165	1000	682	68
Bihar	231	278	214	145	132	1000	5571	310
Chhattisgarh	196	432	147	208	17	1000	2379	104
Delhi	0	1000	0	0	0	1000	13	1
Goa	14	0	986	0	0	1000	218	6
Gujarat	153	387	355	105	0	1000	4422	166
Haryana	239	429	253	79	0	1000	748	39
Himachal Pradesh	234	219	366	173	8	1000	705	103
Jammu & Kashmir	149	676	125	49	0	1000	150	23
Jharkhand	140	482	178	165	36	1000	1596	111
Karnataka	153	300	407	140	0	1000	3616	115
Kerala	50	396	339	204	11	1000	3090	129
Madhya Pradesh	216	448	208	124	4	1000	6375	233
Maharashtra	246	333	296	117	8	1000	9727	260
Manipur	413	289	78	166	55	1000	45	24
Meghalaya	56	351	69	224	301	1000	121	26
Mizoram	0	828	0	102	71	1000	5	5
Nagaland	0	685	315	0	0	1000	20	11
Orissa	208	312	266	136	78	1000	3914	160
Punjab	323	266	389	22	0	1000	1740	56
Rajasthan	189	403	280	128	0	1000	3325	163
Sikkim	0	598	313	88	0	1000	16	15
Tamil Nadu	94	527	330	48	0	1000	11204	360
Tripura	599	128	111	147	13	1000	388	69
Uttaranchal	103	200	619	77	0	1000	1048	43
Uttar Pradesh	178	306	255	148	113	1000	12017	501
West Bengal	331	338	196	115	20	1000	4043	166
A & N Islands	0	0	1000	0	0	1000	8	5
Chandigarh	67	839	0	95	0	1000	4	8
Dadra & N. Haveli	490	335	0	175	0	1000	9	5
Daman & Diu	148	804	0	48	0	1000	6	9
Lakshadweep	0	660	340	0	0	1000	1	3
Pondicherry	41	246	489	224	0	1000	86	10
ONES	436	220	113	153	78	1000	624	172
GUTs	79	288	444	189	0	1000	114	40
all-India	188	384	280	114	34	1000	89662	3745
estd. no. of aged living alone or with spouse only (00)	16883	34437	25078	10225	3039	89662	X	X
sample aged living alone or with spouse only	767	1398	989	448	143	3745	X	X

For peer review only - http://bmjopen.bmj.com/site/about/guidelines.xhtml

Appendix A

Table (15): Per 1000 distribution of aged persons living alone or living with spouse only by location of residence of any child/ grandchild or sibling for each sex

Urban — Males

state/ut	location of residence of child/grandchild/sibling						no. of aged persons living alone or with spouse only	
	within the same building	within the village/ town	outside village/town	not applicable	n.r.	total	estd. (00)	sample
(1)	(2)	(3)	(4)	(5)	(6)	(7)	(8)	(9)
Andhra Pradesh	218	264	404	114	0	1000	1290	90
Arunachal Pradesh	385	269	115	0	231	1000	1	6
Assam	92	0	319	336	252	1000	23	5
Bihar	356	214	84	96	250	1000	152	14
Chhattisgarh	599	227	0	174	0	1000	31	5
Delhi	126	346	303	226	0	1000	316	31
Goa	237	0	763	0	0	1000	24	4
Gujarat	171	200	489	140	0	1000	676	39
Haryana	552	83	365	0	0	1000	180	13
Himachal Pradesh	0	452	174	0	375	1000	21	4
Jammu & Kashmir	308	286	194	213	0	1000	56	11
Jharkhand	74	47	669	13	196	1000	118	18
Karnataka	116	295	447	143	0	1000	418	39
Kerala	106	306	345	243	0	1000	697	45
Madhya Pradesh	174	339	272	183	31	1000	508	46
Maharashtra	158	177	512	153	0	1000	1887	71
Manipur	902	0	98	0	0	1000	13	9
Meghalaya	0	688	312	0	0	1000	2	2
Mizoram	0	370	107	161	362	1000	5	9
Nagaland	0	0	0	0	0	0	0	0
Orissa	67	315	259	83	276	1000	274	25
Punjab	262	300	346	82	10	1000	301	21
Rajasthan	353	313	191	144	0	1000	409	36
Sikkim	0	0	0	0	0	0	0	0
Tamil Nadu	111	430	327	131	0	1000	2042	122
Tripura	434	0	0	308	258	1000	21	8
Uttaranchal	95	236	668	0	0	1000	112	10
Uttar Pradesh	207	175	386	169	63	1000	1321	98
West Bengal	90	299	394	146	70	1000	1164	70
A & N Islands	0	0	0	0	0	0	0	0
Chandigarh	203	175	582	40	0	1000	39	10
Dadra & N. Haveli	0	934	66	0	0	1000	0	3
Daman & Diu	0	560	0	440	0	1000	2	2
Lakshadweep	0	1000	0	0	0	1000	1	1
Pondicherry	274	509	111	105	0	1000	45	10
ONES	504	86	62	172	175	1000	42	34
GUTs	233	368	319	81	0	1000	87	26
all-India	169	275	384	144	28	1000	12150	877
estd. no. of aged living alone or with spouse only (00)	2052	3338	4670	1748	342	12150	X	X
sample aged living alone or with spouse only	178	250	287	133	29	877	X	X

For peer review only - http://bmjopen.bmj.com/site/about/guidelines.xhtml

Appendix A

Table (15): Per 1000 distribution of aged persons living alone or living with spouse only by location of residence of any child/ grandchild or sibling for each sex

Urban Females

state/ut	location of residence of child/grandchild/sibling						no. of aged persons living alone or with spouse only	
	within the same building	within the village/ town	outside village/town	not applicable	n.r.	total	estd. (00)	sample
(1)	(2)	(3)	(4)	(5)	(6)	(7)	(8)	(9)
Andhra Pradesh	204	365	320	111	0	1000	1145	79
Arunachal Pradesh	400	0	415	0	185	1000	1	7
Assam	0	0	0	0	0	0	0	0
Bihar	352	158	194	78	219	1000	247	17
Chhattisgarh	175	381	0	444	0	1000	137	15
Delhi	209	424	233	134	0	1000	303	27
Goa	0	624	279	97	0	1000	58	7
Gujarat	139	310	439	111	0	1000	810	41
Haryana	308	272	420	0	0	1000	255	17
Himachal Pradesh	0	1000	0	0	0	1000	9	1
Jammu & Kashmir	340	253	164	242	0	1000	47	10
Jharkhand	11	0	591	140	258	1000	90	9
Karnataka	186	244	451	119	0	1000	420	31
Kerala	22	452	371	155	0	1000	578	34
Madhya Pradesh	231	403	166	163	37	1000	521	45
Maharashtra	222	272	391	77	38	1000	2068	72
Manipur	414	181	405	0	0	1000	9	3
Meghalaya	290	579	131	0	0	1000	6	4
Mizoram	240	760	0	0	0	1000	1	4
Nagaland	0	0	0	0	0	0	0	0
Orissa	158	662	106	74	0	1000	115	12
Punjab	369	327	185	105	14	1000	220	16
Rajasthan	294	399	242	65	0	1000	437	32
Sikkim	0	0	0	0	0	0	0	0
Tamil Nadu	101	476	233	190	0	1000	1975	116
Tripura	615	156	73	156	0	1000	10	6
Uttaranchal	18	281	473	228	0	1000	97	10
Uttar Pradesh	181	165	327	177	150	1000	1082	74
West Bengal	118	422	291	164	5	1000	819	48
A & N Islands	0	1000	0	0	0	1000	0	1
Chandigarh	237	289	349	125	0	1000	44	11
Dadra & N. Haveli	0	1000	0	0	0	1000	0	1
Daman & Diu	0	140	860	0	0	1000	3	2
Lakshadweep	0	525	0	475	0	1000	2	2
Pondicherry	366	634	0	0	0	1000	56	8
ONES	450	281	205	56	7	1000	26	24
GUTs	296	475	170	60	0	1000	105	25
all-India	177	348	312	133	30	1000	11565	762
estd. no. of aged living alone or with spouse only (00)	2051	4023	3603	1543	345	11565	X	X
sample aged living alone or with spouse only	157	279	197	110	19	762	X	X

For peer review only - http://bmjopen.bmj.com/site/about/guidelines.xhtml

Appendix A

Table (15): Per 1000 distribution of aged persons living alone or living with spouse only by location of residence of any child/ grandchild or sibling for each sex

Urban Persons

state/ut	location of residence of child/grandchild/sibling						no. of aged persons living alone or with spouse only	
	within the same building	within the village/ town	outside village/town	not applicable	n.r.	total	estd. (00)	sample
(1)	(2)	(3)	(4)	(5)	(6)	(7)	(8)	(9)
Andhra Pradesh	212	312	364	113	0	1000	2435	169
Arunachal Pradesh	393	120	282	0	205	1000	2	13
Assam	92	0	319	336	252	1000	23	5
Bihar	353	179	152	85	231	1000	399	31
Chhattisgarh	254	352	0	394	0	1000	168	20
Delhi	166	384	269	181	0	1000	619	58
Goa	69	443	419	69	0	1000	81	11
Gujarat	154	260	462	124	0	1000	1486	80
Haryana	409	194	397	0	0	1000	435	30
Himachal Pradesh	0	622	120	0	258	1000	30	5
Jammu & Kashmir	323	271	180	226	0	1000	103	21
Jharkhand	47	27	635	68	223	1000	209	27
Karnataka	151	270	449	131	0	1000	838	70
Kerala	68	372	357	203	0	1000	1275	79
Madhya Pradesh	203	372	218	173	34	1000	1029	91
Maharashtra	192	226	448	113	20	1000	3955	143
Manipur	706	73	221	0	0	1000	22	12
Meghalaya	204	611	185	0	0	1000	8	6
Mizoram	54	457	83	125	281	1000	6	13
Nagaland	0	0	0	0	0	0	0	0
Orissa	94	418	214	80	194	1000	390	37
Punjab	307	311	278	92	12	1000	522	37
Rajasthan	323	358	217	103	0	1000	846	68
Sikkim	0	0	0	0	0	0	0	0
Tamil Nadu	106	453	281	160	0	1000	4017	238
Tripura	490	49	23	261	177	1000	31	14
Uttaranchal	59	257	577	106	0	1000	209	20
Uttar Pradesh	196	170	359	173	102	1000	2403	172
West Bengal	101	350	352	154	43	1000	1982	118
A & N Islands	0	1000	0	0	0	1000	0	1
Chandigarh	221	236	458	85	0	1000	84	21
Dadra & N. Haveli	0	943	57	0	0	1000	1	4
Daman & Diu	0	294	544	162	0	1000	4	4
Lakshadweep	0	688	0	312	0	1000	2	3
Pondicherry	325	578	50	47	0	1000	102	18
ONES	483	161	117	128	110	1000	69	58
GUTs	267	426	237	69	0	1000	193	51
all-India	173	310	349	139	29	1000	23715	1639
estd. no. of aged living alone or with spouse only (00)	4104	7361	8273	3290	687	23715	X	X
sample aged living alone or with spouse only	335	529	484	243	48	1639	X	X

For peer review only - http://bmjopen.bmj.com/site/about/guidelines.xhtml

Table (15): Per 1000 distribution of aged persons living alone or living with spouse only by location of residence of any child/ grandchild or sibling for each sex

Rural + Urban | Males

state/ut	location of residence of child/grandchild/sibling						no. of aged persons living alone or with spouse only	
	within the same building	within the village/ town	outside village/town	not applicable	n.r.	total	estd. (00)	sample
(1)	(2)	(3)	(4)	(5)	(6)	(7)	(8)	(9)
Andhra Pradesh	220	410	265	93	12	1000	8056	329
Arunachal Pradesh	293	211	149	75	272	1000	16	18
Assam	120	287	385	26	181	1000	297	35
Bihar	247	306	174	134	139	1000	3540	206
Chhattisgarh	222	407	146	222	4	1000	1040	52
Delhi	126	346	303	226	0	1000	316	31
Goa	35	0	965	0	0	1000	160	7
Gujarat	163	337	406	94	0	1000	3005	125
Haryana	328	356	256	59	0	1000	575	33
Himachal Pradesh	228	221	330	190	31	1000	441	62
Jammu & Kashmir	158	551	149	142	0	1000	136	23
Jharkhand	154	477	198	123	48	1000	1234	93
Karnataka	125	308	443	124	0	1000	2267	94
Kerala	79	324	378	219	0	1000	2321	110
Madhya Pradesh	187	461	210	133	10	1000	3774	169
Maharashtra	257	304	328	106	6	1000	6718	207
Manipur	578	161	94	118	49	1000	50	27
Meghalaya	27	362	134	324	153	1000	68	17
Mizoram	0	628	54	135	183	1000	10	13
Nagaland	0	708	292	0	0	1000	14	8
Orissa	199	275	262	166	99	1000	2711	122
Punjab	325	309	311	53	3	1000	1197	50
Rajasthan	241	346	288	125	0	1000	2124	123
Sikkim	0	673	197	129	0	1000	8	9
Tamil Nadu	126	465	340	69	0	1000	8035	316
Tripura	623	100	71	165	40	1000	263	53
Uttaranchal	181	99	623	96	0	1000	559	30
Uttar Pradesh	180	291	263	169	97	1000	7728	368
West Bengal	269	281	267	149	36	1000	3348	157
A & N Islands	0	0	1000	0	0	1000	3	3
Chandigarh	193	226	536	45	0	1000	43	16
Dadra & N. Haveli	385	609	6	0	0	1000	6	6
Daman & Diu	69	536	0	395	0	1000	3	5
Lakshadweep	0	803	197	0	0	1000	1	2
Pondicherry	142	394	336	129	0	1000	112	17
ONES	466	194	96	175	69	1000	427	145
GUTs	158	356	382	104	0	1000	168	49
all-India	198	352	294	123	33	1000	60179	2936
estd. no. of aged living alone or with spouse only (00)	11915	21188	17717	7388	1972	60179	X	X
sample aged living alone or with spouse only	622	997	818	391	108	2936	X	X

For peer review only

For peer review only - http://bmjopen.bmj.com/site/about/guidelines.xhtml

Appendix A

Table (15): Per 1000 distribution of aged persons living alone or living with spouse only by location of residence of any child/ grandchild or sibling for each sex

Rural + Urban Females

state/ut	location of residence of child/grandchild/sibling						no. of aged persons living alone or with spouse only	
	within the same building	within the village/ town	outside village/town	not applicable	n.r.	total	estd. (00)	sample
(1)	(2)	(3)	(4)	(5)	(6)	(7)	(8)	(9)
Andhra Pradesh	158	490	272	70	10	1000	6721	256
Arunachal Pradesh	687	93	153	54	13	1000	15	17
Assam	227	267	292	55	158	1000	408	38
Bihar	228	221	262	152	137	1000	2429	135
Chhattisgarh	185	440	131	220	24	1000	1507	72
Delhi	200	447	224	129	0	1000	316	28
Goa	22	259	679	40	0	1000	139	10
Gujarat	143	374	356	126	0	1000	2903	121
Haryana	276	329	353	42	0	1000	608	36
Himachal Pradesh	220	257	395	128	0	1000	294	46
Jammu & Kashmir	291	465	147	97	0	1000	117	21
Jharkhand	76	325	303	219	77	1000	571	45
Karnataka	181	279	386	154	0	1000	2187	91
Kerala	29	463	306	186	17	1000	2044	98
Madhya Pradesh	243	414	210	128	5	1000	3630	155
Maharashtra	205	301	352	125	17	1000	6963	196
Manipur	296	394	219	91	0	1000	16	9
Meghalaya	106	373	12	85	424	1000	62	15
Mizoram	190	603	0	0	206	1000	2	5
Nagaland	0	636	364	0	0	1000	6	3
Orissa	197	401	261	72	70	1000	1593	75
Punjab	313	240	422	22	3	1000	1065	43
Rajasthan	191	442	246	120	0	1000	2047	108
Sikkim	0	528	423	50	0	1000	8	6
Tamil Nadu	66	555	292	87	0	1000	7186	282
Tripura	538	161	162	139	0	1000	157	30
Uttaranchal	28	298	604	70	0	1000	698	33
Uttar Pradesh	182	274	284	133	127	1000	6692	305
West Bengal	239	419	224	101	18	1000	2677	127
A & N Islands	0	35	965	0	0	1000	5	3
Chandigarh	235	296	345	123	0	1000	45	13
Dadra & N. Haveli	572	19	0	409	0	1000	4	3
Daman & Diu	92	609	300	0	0	1000	8	8
Lakshadweep	0	620	0	380	0	1000	2	4
Pondicherry	273	473	125	128	0	1000	76	11
ONES	400	246	142	112	101	1000	265	85
GUTs	245	397	232	126	0	1000	140	42
all-India	171	387	294	115	33	1000	53198	2448
estd. no. of aged living alone or with spouse only (00)	9072	20610	15635	6128	1754	53198	X	X
sample aged living alone or with spouse only	480	930	655	300	83	2448	X	X

For peer review only - http://bmjopen.bmj.com/site/about/guidelines.xhtml

Appendix A

Table (15): Per 1000 distribution of aged persons living alone or living with spouse only by location of residence of any child/ grandchild or sibling for each sex

Rural + Urban Persons

state/ut	location of residence of child/grandchild/sibling						no. of aged persons living alone or with spouse only	
	within the same building	within the village/ town	outside village/town	not applicable	n.r.	total	estd. (00)	sample
(1)	(2)	(3)	(4)	(5)	(6)	(7)	(8)	(9)
Andhra Pradesh	192	446	268	82	11	1000	14777	585
Arunachal Pradesh	480	155	151	65	149	1000	31	35
Assam	182	276	331	43	168	1000	705	73
Bihar	239	272	210	141	138	1000	5969	341
Chhattisgarh	200	427	137	221	16	1000	2547	124
Delhi	163	396	263	177	0	1000	631	59
Goa	29	120	832	19	0	1000	299	17
Gujarat	153	355	382	110	0	1000	5908	246
Haryana	302	342	306	50	0	1000	1183	69
Himachal Pradesh	225	235	356	165	19	1000	736	108
Jammu & Kashmir	219	511	148	121	0	1000	254	44
Jharkhand	129	429	231	153	57	1000	1805	138
Karnataka	152	294	415	139	0	1000	4454	185
Kerala	56	389	344	203	8	1000	4365	208
Madhya Pradesh	214	438	210	131	8	1000	7403	324
Maharashtra	231	302	340	116	11	1000	13681	403
Manipur	509	218	125	112	37	1000	66	36
Meghalaya	65	367	76	210	282	1000	129	32
Mizoram	29	624	46	114	186	1000	11	18
Nagaland	0	685	315	0	0	1000	20	11
Orissa	198	321	261	131	88	1000	4304	197
Punjab	319	276	363	38	3	1000	2262	93
Rajasthan	216	393	267	123	0	1000	4171	231
Sikkim	0	598	313	88	0	1000	16	15
Tamil Nadu	98	508	317	78	0	1000	15222	598
Tripura	592	123	105	156	25	1000	419	83
Uttaranchal	96	210	612	82	0	1000	1257	63
Uttar Pradesh	181	283	272	152	111	1000	14420	673
West Bengal	256	342	247	127	28	1000	6025	284
A & N Islands	0	22	978	0	0	1000	8	6
Chandigarh	215	262	438	85	0	1000	87	29
Dadra & N. Haveli	460	372	3	165	0	1000	10	9
Daman & Diu	86	591	227	95	0	1000	11	13
Lakshadweep	0	683	67	250	0	1000	3	6
Pondicherry	195	426	251	128	0	1000	188	28
ONES	441	214	113	151	81	1000	693	230
GUTs	197	375	314	114	0	1000	307	91
all-India	185	369	294	119	33	1000	113377	5384
estd. no. of aged living alone or with spouse only (00)	20986	41798	33352	13515	3726	113377	X	X
sample aged living alone or with spouse only	1102	1927	1473	691	191	5384	X	X

For peer review only - http://bmjopen.bmj.com/site/about/guidelines.xhtml

Appendix A

Table (16): Per 1000 distribution of aged persons by state of physical mobility for each age–group and sex

all-India Rural

age-group	state of physical mobility of aged					no. of aged persons	
	mobile	immobile		n.r.	total	estd. (00)	sample
		confined to bed	confined to home				
(1)	(2)	(3)	(4)	(5)	(6)	(7)	(8)
male							
60-64	950	4	23	23	1000	92678	4200
64-69	938	7	44	11	1000	71760	3262
70-74	904	16	63	18	1000	47686	2164
75-79	869	23	94	14	1000	18822	903
80 & above	724	49	171	57	1000	22308	999
all aged	912	12	55	21	1000	253254	11528
estd. no. of aged (00)	230926	3160	13889	5278	253254	X	X
sample aged	10305	212	746	265	11528	X	X
female							
60-64	944	3	31	22	1000	91693	3971
64-69	934	6	44	15	1000	74886	3201
70-74	852	20	112	16	1000	46859	1970
75-79	818	33	130	19	1000	16962	744
80 & above	632	69	257	41	1000	19015	851
all aged	891	14	74	20	1000	249416	10737
estd. no. of aged (00)	222326	3585	18462	5043	249416	X	X
sample aged	9376	194	904	263	10737	X	X
persons							
60-64	947	4	27	23	1000	184371	8171
64-69	936	7	44	13	1000	146646	6463
70-74	878	18	87	17	1000	94546	4134
75-79	845	28	111	16	1000	35784	1647
80 & above	682	58	211	49	1000	41323	1850
all aged	902	13	64	21	1000	502670	22265
estd. no. of aged (00)	453253	6745	32351	10321	502670	X	X
sample aged	19681	406	1650	528	22265	X	X

For peer review only - http://bmjopen.bmj.com/site/about/guidelines.xhtml

Appendix A

Table (16): Per 1000 distribution of aged persons by state of physical mobility for each age–group and sex

all-India Urban

age-group	state of physical mobility of aged					no. of aged persons	
	mobile	immobile		n.r.	total	estd. (00)	sample
		confined to bed	confined to home				
(1)	(2)	(3)	(4)	(5)	(6)	(7)	(8)
male							
60-64	952	8	25	15	1000	27406	2199
64-69	951	11	23	15	1000	21927	1719
70-74	912	18	59	11	1000	14634	1186
75-79	885	18	95	3	1000	7322	569
80 & above	727	69	170	34	1000	7462	549
all aged	917	17	51	15	1000	78751	6222
estd. no. of aged (00)	72193	1348	4034	1177	78751	X	X
sample aged	5628	125	352	117	6222	X	X
female							
60-64	940	5	29	25	1000	28628	2156
64-69	925	8	58	10	1000	24032	1838
70-74	881	20	96	3	1000	14463	1164
75-79	797	28	157	18	1000	6941	549
80 & above	657	69	254	20	1000	8295	637
all aged	885	17	83	16	1000	82359	6344
estd. no. of aged (00)	72859	1394	6817	1288	82359	X	X
sample aged	5512	135	556	141	6344	X	X
persons							
60-64	946	6	27	20	1000	56034	4355
64-69	938	9	41	12	1000	45959	3557
70-74	896	19	78	7	1000	29097	2350
75-79	842	22	125	10	1000	14263	1118
80 & above	690	69	214	26	1000	15757	1186
all aged	900	17	67	15	1000	161110	12566
estd. no. of aged (00)	145052	2742	10851	2464	161110	X	X
sample aged	11140	260	908	258	12566	X	X

For peer review only - http://bmjopen.bmj.com/site/about/guidelines.xhtml

For peer review only

Appendix A

Table (16): Per 1000 distribution of aged persons by state of physical mobility for each age–group and sex

all-India Rural+Urban

age-group	state of physical mobility of aged					no. of aged persons	
	mobile	immobile		n.r.	total	estd. (00)	sample
		confined to bed	confined to home				
(1)	(2)	(3)	(4)	(5)	(6)	(7)	(8)
male							
60-64	950	5	24	21	1000	120083	6399
64-69	941	8	39	12	1000	93687	4981
70-74	906	16	62	16	1000	62320	3350
75-79	873	21	94	11	1000	26145	1472
80 & above	725	54	171	51	1000	29769	1548
all aged	913	14	54	19	1000	332005	17750
estd. no. of aged (00)	303119	4508	17923	6455	332005	X	X
sample aged	15933	337	1098	382	17750	X	X
female							
60-64	943	4	30	23	1000	120321	6127
64-69	932	6	47	14	1000	98918	5039
70-74	859	20	108	13	1000	61322	3134
75-79	812	32	138	18	1000	23903	1293
80 & above	640	69	256	35	1000	27310	1488
all aged	890	15	76	19	1000	331775	17081
estd. no. of aged (00)	295186	4980	25279	6330	331775	X	X
sample aged	14888	329	1460	404	17081	X	X
persons							
60-64	947	4	27	22	1000	240405	12526
64-69	936	7	43	13	1000	192605	10020
70-74	882	18	85	14	1000	123643	6484
75-79	844	26	115	15	1000	50048	2765
80 & above	684	61	212	43	1000	57080	3036
all aged	901	14	65	19	1000	663779	34831
estd. no. of aged (00)	598305	9488	43202	12785	663779	X	X
sample aged	30821	666	2558	786	34831	X	X

For peer review only - http://bmjopen.bmj.com/site/about/guidelines.xhtml

For peer review only

Appendix A

Table (17.1): Per 1000 distribution of aged persons with illness or otherwise by their perception about current state of health for each quintile class and sex

all-India Rural Males

mpce class	no. per 1000 of aged persons reporting illness	aged person with illness: own perception about current state of health					aged person without illness: own perception about current state of health					no. of aged persons	
		excellent/ very good	good/ fair	poor	n.r.	total	excellent/ very good	good/ fair	poor	n.r.	total	estd. (00)	sample
(1)	(2)	(3)	(4)	(5)	(6)	(7)	(8)	(9)	(10)	(11)	(12)	(13)	(14)
0 – 225	280	3	457	493	47	1000	41	710	161	88	1000	9537	399
225 – 255	191	0	432	486	81	1000	34	703	205	58	1000	8411	302
255 – 300	216	4	480	478	38	1000	67	724	147	62	1000	20867	805
300 – 340	213	0	518	480	1	1000	85	655	196	64	1000	17295	744
340 – 380	257	4	606	390	0	1000	53	703	176	67	1000	22031	964
380 – 420	273	4	534	450	12	1000	73	728	153	46	1000	24050	1046
420 – 470	299	61	497	407	34	1000	74	756	137	34	1000	26188	1184
470 – 525	284	10	575	384	31	1000	50	725	153	72	1000	27442	1281
525 – 615	283	7	568	408	17	1000	102	748	103	47	1000	31708	1492
615 – 775	342	16	631	340	13	1000	79	753	114	54	1000	32712	1576
775 – 950	357	38	641	308	13	1000	136	766	88	9	1000	14637	810
950 +	455	41	641	318	0	1000	122	774	70	34	1000	18376	925
all classes	292	19	569	393	19	1000	77	731	139	53	1000	253254	
estd. no. of aged persons (00)	253254	1415	42171	29092	1391	74068	13773	130933	24989	9491	179186	X	X
sample aged persons	11528	61	1785	1485	70	3401	630	5782	1223	492	8127	X	X

For peer review only - http://bmjopen.bmj.com/site/about/guidelines.xhtml

Appendix A

Table (17.1): Per 1000 distribution of aged persons with illness or otherwise by their perception about current state of health for each quintile class and sex

all-India — Rural — Females

mpce class	no. per 1000 of aged persons reporting illness	aged person with illness: own perception about current state of health					aged person without illness: own perception about current state of health					no. of aged persons	
		excellent/ very good	good/ fair	poor	n.r.	total	excellent/ very good	good/ fair	poor	n.r.	total	estd. (00)	sample
(1)	(2)	(3)	(4)	(5)	(6)	(7)	(8)	(9)	(10)	(11)	(12)	(13)	(14)
0 – 225	206	31	361	606	3	1000	35	709	191	65	1000	11222	415
225 – 255	188	0	371	629	0	1000	28	651	224	98	1000	8828	320
255 – 300	214	13	387	573	27	1000	13	743	185	59	1000	21088	797
300 – 340	259	1	438	532	30	1000	28	708	204	60	1000	17861	716
340 – 380	291	37	465	458	41	1000	26	731	191	52	1000	22651	891
380 – 420	248	5	510	457	28	1000	48	712	177	63	1000	22017	930
420 – 470	262	5	536	399	59	1000	49	710	188	52	1000	24398	1065
470 – 525	281	14	527	439	20	1000	49	715	186	49	1000	27514	1214
525 – 615	279	2	514	457	26	1000	46	750	162	42	1000	28621	1308
615 – 775	318	7	570	398	25	1000	38	766	152	44	1000	29982	1407
775 – 950	359	28	577	378	17	1000	63	783	134	20	1000	16256	776
950 +	460	28	574	390	8	1000	66	756	148	30	1000	18978	898
all classes	286	14	511	448	26	1000	41	731	177	51	1000	249416	10737
estd. no. of aged persons (00)	249416	1035	36519	32011	1836	71401	7235	130155	31515	9110	178015	X	X
sample aged persons	10737	33	1528	1469	84	3114	316	5403	1449	455	7623	X	X

For peer review only

For peer review only - http://bmjopen.bmj.com/site/about/guidelines.xhtml

Appendix A

Table (17.1): Per 1000 distribution of aged persons with illness or otherwise by their perception about current state of health for each quintile class and sex

all-India Rural Persons

mpce class	no. per 1000 of aged persons reporting illness	aged person with illness					aged person without illness					no. of aged persons	
		own perception about current state of health					own perception about current state of health						
		excellent/ very good	good/ fair	poor	n.r.	total	excellent/ very good	good/ fair	poor	n.r.	total	estd. (00)	sample
(1)	(2)	(3)	(4)	(5)	(6)	(7)	(8)	(9)	(10)	(11)	(12)	(13)	(14)
0 – 225	240	16	412	545	27	1000	38	709	178	75	1000	20759	814
225 – 255	189	0	401	559	40	1000	31	676	215	78	1000	17239	622
255 – 300	215	8	434	525	32	1000	40	734	166	60	1000	41955	1602
300 – 340	236	0	473	509	17	1000	57	681	200	62	1000	35156	1460
340 – 380	274	22	530	426	22	1000	40	717	184	60	1000	44682	1855
380 – 420	261	5	523	453	19	1000	61	720	165	55	1000	46067	1976
420 – 470	281	36	515	404	45	1000	62	733	162	43	1000	50586	2249
470 – 525	283	12	551	411	26	1000	50	720	170	61	1000	54956	2495
525 – 615	281	5	543	431	21	1000	75	749	131	45	1000	60329	2800
615 – 775	331	12	603	367	18	1000	59	760	133	49	1000	62693	2983
775 – 950	358	33	607	345	15	1000	98	775	112	15	1000	30893	1586
950 +	458	34	607	355	4	1000	94	765	109	32	1000	37355	1823
all classes	289	17	541	420	22	1000	59	731	158	52	1000	502670	22265
estd. no. of aged persons (00)	502670	2450	78690	61103	3227	145469	21008	261088	56504	18600	357200	X	X
sample aged persons	22265	94	3313	2954	154	6515	946	11185	2672	947	15750	X	X

For peer review only - http://bmjopen.bmj.com/site/about/guidelines.xhtml

Table (17.1): Per 1000 distribution of aged persons with illness or otherwise by their perception about current state of health for each quintile class and sex

all-India | Urban | Males

mpce class	no. per 1000 of aged persons reporting illness	aged person with illness					aged person without illness					no. of aged persons	
		own perception about current state of health					own perception about current state of health						
		excellent/ very good	good/ fair	poor	n.r.	total	excellent/ very good	good/ fair	poor	n.r.	total	estd. (00)	sample
(1)	(2)	(3)	(4)	(5)	(6)	(7)	(8)	(9)	(10)	(11)	(12)	(13)	(14)
0 – 225	290	12	362	625	0	1000	81	574	273	72	1000	1695	118
225 – 255	327	113	670	217	0	1000	99	669	202	30	1000	1314	83
255 – 300	291	0	507	478	15	1000	31	770	161	38	1000	3731	271
300 – 340	308	1	441	533	24	1000	75	716	186	24	1000	7743	576
340 – 380	348	3	582	415	0	1000	106	727	96	71	1000	3552	296
380 – 420	287	19	550	413	18	1000	70	788	100	42	1000	5877	493
420 – 470	317	20	557	397	25	1000	105	776	84	36	1000	8343	707
470 – 525	299	17	677	294	11	1000	84	785	96	34	1000	8126	727
525 – 615	340	25	650	323	1	1000	147	735	84	33	1000	10204	894
615 – 775	455	54	719	216	11	1000	120	744	97	39	1000	13511	1031
775 – 950	337	58	687	253	2	1000	117	771	85	28	1000	4443	350
950 +	474	37	712	244	7	1000	192	733	51	25	1000	10211	676
all classes	360	31	635	324	11	1000	110	747	107	36	1000	78751	6222
estd. no. of aged persons (00)	78751	873	17988	9173	307	28341	5534	37672	5396	1808	50410	X	X
sample aged persons	6222	50	1264	749	34	2097	448	3014	429	234	4125	X	X

For peer review only

For peer review only - http://bmjopen.bmj.com/site/about/guidelines.xhtml

Appendix A

Table (17.1): Per 1000 distribution of aged persons with illness or otherwise by their perception about current state of health for each quintile class and sex

all-India Urban Females

mpce class	no. per 1000 of aged persons reporting illness	aged person with illness: own perception about current state of health					aged person without illness: own perception about current state of health					no. of aged persons	
		excellent/ very good	good/ fair	poor	n.r.	total	excellent/ very good	good/ fair	poor	n.r.	total	estd. (00)	sample
(1)	(2)	(3)	(4)	(5)	(6)	(7)	(8)	(9)	(10)	(11)	(12)	(13)	(14)
0 – 225	206	0	487	513	0	1000	87	529	276	108	1000	2745	170
225 – 255	372	0	436	564	0	1000	137	445	354	64	1000	1461	111
255 – 300	254	0	531	444	24	1000	48	619	292	41	1000	4458	315
300 – 340	329	21	477	477	26	1000	47	797	137	19	1000	8766	643
340 – 380	375	2	566	425	6	1000	105	657	153	85	1000	4032	304
380 – 420	361	9	464	500	27	1000	43	808	103	47	1000	7321	565
420 – 470	383	17	627	334	23	1000	42	799	122	37	1000	8550	710
470 – 525	350	27	679	290	4	1000	38	827	113	21	1000	8554	733
525 – 615	440	15	642	337	6	1000	78	782	117	24	1000	10703	892
615 – 775	419	13	723	251	12	1000	79	789	108	24	1000	12163	972
775 – 950	461	0	554	440	5	1000	78	765	116	42	1000	4024	310
950 +	517	49	655	272	25	1000	131	714	133	22	1000	9582	619
all classes	390	19	611	355	15	1000	69	753	143	36	1000	82359	6344
estd. no. of aged persons (00)	82359	601	19637	11426	495	32159	3456	37785	7169	1790	50200	X	X
sample aged persons	6344	38	1399	848	43	2328	252	2962	564	238	4016	X	X

For peer review only - http://bmjopen.bmj.com/site/about/guidelines.xhtml

Appendix A

Table (17.1): Per 1000 distribution of aged persons with illness or otherwise by their perception about current state of health for each quintile class and sex

all-India						Urban							Persons
mpce class	no. per 1000 of aged persons reporting illness	aged person with illness					aged person without illness					no. of aged persons	
		own perception about current state of health					own perception about current state of health						
		excellent/ very good	good/ fair	poor	n.r.	total	excellent/ very good	good/ fair	poor	n.r.	total	estd. (00)	sample
(1)	(2)	(3)	(4)	(5)	(6)	(7)	(8)	(9)	(10)	(11)	(12)	(13)	(14)
0 – 225	238	6	429	565	0	1000	85	545	275	95	1000	4441	288
225 – 255	351	50	539	411	0	1000	119	555	279	47	1000	2775	194
255 – 300	271	0	520	461	20	1000	40	686	234	40	1000	8189	586
300 – 340	319	12	461	502	25	1000	60	758	160	21	1000	16508	1219
340 – 380	363	3	573	421	4	1000	105	690	126	79	1000	7583	600
380 – 420	328	13	498	466	23	1000	56	798	101	45	1000	13198	1058
420 – 470	350	18	596	362	24	1000	75	787	102	36	1000	16894	1417
470 – 525	325	23	678	292	7	1000	61	806	105	28	1000	16680	1460
525 – 615	391	19	646	331	4	1000	115	757	99	29	1000	20907	1786
615 – 775	438	36	721	232	11	1000	100	766	102	31	1000	25674	2003
775 – 950	396	26	613	357	4	1000	100	768	98	34	1000	8468	660
950 +	495	43	683	258	16	1000	163	724	89	24	1000	19793	1295
all classes	376	24	622	340	13	1000	89	750	125	36	1000	161110	12566
estd. no. of aged persons (00)	161110	1474	37625	20599	802	60500	8990	75457	12565	3598	100610	X	X
sample aged persons	12566	88	2663	1597	77	4425	700	5976	993	472	8141	X	X

For peer review only - http://bmjopen.bmj.com/site/about/guidelines.xhtml

For peer review only

Table (17.1): Per 1000 distribution of aged persons with illness or otherwise by their perception about current state of health for each quintile class and sex

all-India Rural + Urban Males

mpce class	no. per 1000 of aged persons reporting illness	aged person with illness: own perception about current state of health					aged person without illness: own perception about current state of health					no. of aged persons	
		excellent/ very good	good/ fair	poor	n.r.	total	excellent/ very good	good/ fair	poor	n.r.	total	estd. (00)	sample
(1)	(2)	(3)	(4)	(5)	(6)	(7)	(8)	(9)	(10)	(11)	(12)	(13)	(14)
0 – 225	281	5	442	513	40	1000	47	690	178	85	1000	11232	517
225 – 255	209	24	482	429	64	1000	42	699	205	54	1000	9725	385
255 – 300	227	3	486	478	33	1000	62	731	149	59	1000	24598	1076
300 – 340	242	1	488	501	10	1000	82	672	193	52	1000	25038	1320
340 – 380	270	4	602	394	0	1000	59	706	166	68	1000	25583	1260
380 – 420	276	7	537	442	13	1000	73	739	142	46	1000	29927	1539
420 – 470	303	51	512	405	32	1000	81	761	124	34	1000	34531	1891
470 – 525	287	12	599	363	26	1000	58	738	140	63	1000	35568	2008
525 – 615	297	12	591	384	12	1000	112	745	99	44	1000	41912	2386
615 – 775	375	30	662	296	12	1000	89	751	110	50	1000	46223	2607
775 – 950	352	42	651	296	11	1000	131	767	87	14	1000	19080	1160
950 +	462	39	667	291	3	1000	146	760	63	31	1000	28588	1601
all classes	308	22	587	374	17	1000	84	734	132	49	1000	332005	17750
estd. no. of aged persons (00)	332005	2288	60159	38265	1698	102409	19307	168605	30385	11298	229596	X	X
sample aged persons	17750	111	3049	2234	104	5498	1078	8796	1652	726	12252	X	X

For peer review only - http://bmjopen.bmj.com/site/about/guidelines.xhtml

Appendix A

Table (17.1): Per 1000 distribution of aged persons with illness or otherwise by their perception about current state of health for each quintile class and sex

all-India Rural + Urban Females

mpce class	no. per 1000 of aged persons reporting illness	aged person with illness: own perception about current state of health					aged person without illness: own perception about current state of health					no. of aged persons	
		excellent/ very good	good/ fair	poor	n.r.	total	excellent/ very good	good/ fair	poor	n.r.	total	estd. (00)	sample
(1)	(2)	(3)	(4)	(5)	(6)	(7)	(8)	(9)	(10)	(11)	(12)	(13)	(14)
0 – 225	206	25	385	588	2	1000	46	673	208	73	1000	13968	585
225 – 255	214	0	387	613	0	1000	40	627	239	94	1000	10289	431
255 – 300	221	11	416	547	26	1000	19	722	203	56	1000	25546	1112
300 – 340	282	8	453	511	28	1000	34	735	184	48	1000	26626	1359
340 – 380	304	30	484	451	34	1000	37	721	186	56	1000	26683	1195
380 – 420	276	6	495	471	28	1000	47	733	161	60	1000	29338	1495
420 – 470	293	9	567	377	47	1000	48	730	173	49	1000	32949	1775
470 – 525	298	18	570	397	16	1000	47	740	170	43	1000	36068	1947
525 – 615	323	7	562	413	19	1000	53	757	152	38	1000	39323	2200
615 – 775	347	9	624	347	20	1000	49	772	141	38	1000	42144	2379
775 – 950	379	21	571	393	14	1000	66	780	131	23	1000	20281	1086
950 +	480	35	603	347	14	1000	86	743	143	27	1000	28560	1517
all classes	312	16	542	419	23	1000	47	736	170	48	1000	331775	17081
estd. no. of aged persons (00)	331775	1636	56156	43437	2331	103560	10691	167940	38684	10899	228214	X	X
sample aged persons	17081	71	2927	2317	127	5442	568	8365	2013	693	11639	X	X

For peer review only - http://bmjopen.bmj.com/site/about/guidelines.xhtml

Appendix A

Table (17.1): Per 1000 distribution of aged persons with illness or otherwise by their perception about current state of health for each quintile class and sex

all-India Rural + Urban Persons

mpce class	no. per 1000 of aged persons reporting illness	aged person with illness: own perception about current state of health					aged person without illness: own perception about current state of health					no. of aged persons	
		excellent/ very good	good/ fair	poor	n.r.	total	excellent/ very good	good/ fair	poor	n.r.	total	estd. (00)	sample
(1)	(2)	(3)	(4)	(5)	(6)	(7)	(8)	(9)	(10)	(11)	(12)	(13)	(14)
0 – 225	240	14	415	549	22	1000	46	680	195	78	1000	25200	1102
225 – 255	212	11	433	525	31	1000	41	662	222	75	1000	20014	816
255 – 300	224	7	451	513	30	1000	40	726	176	57	1000	50144	2188
300 – 340	263	5	468	506	20	1000	58	704	188	50	1000	51664	2679
340 – 380	287	18	538	425	19	1000	48	714	176	62	1000	52266	2455
380 – 420	276	7	516	456	20	1000	60	736	152	53	1000	59265	3034
420 – 470	298	31	539	391	39	1000	65	746	148	41	1000	67479	3666
470 – 525	292	15	584	380	21	1000	52	739	155	53	1000	71636	3955
525 – 615	309	10	576	399	16	1000	84	751	124	41	1000	81235	4586
615 – 775	362	20	645	319	16	1000	69	761	125	44	1000	88367	4986
775 – 950	366	31	609	348	13	1000	98	774	109	19	1000	39361	2246
950 +	471	37	635	320	9	1000	117	751	103	29	1000	57148	3118
all classes	310	19	565	397	20	1000	66	735	151	48	1000	663779	34831
estd. no. of aged persons (00)	663779	3924	116315	81702	4029	205970	29998	336545	69069	22198	457810	X	X
sample aged persons	34831	182	5976	4551	231	10940	1646	17161	3665	1419	23891	X	X

For peer review only

For peer review only - http://bmjopen.bmj.com/site/about/guidelines.xhtml

Appendix A

Table (17.2): Per 1000 distribution of aged persons with illness or otherwise by their perception about current state of health for each sex

Rural | Males

state/ut	no. per 1000 of aged persons reporting illness	aged person with illness — own perception about current state of health					aged person without illness — own perception about current state of health					no. of aged persons	
		excellent/ very good	good/ fair	poor	n.r.	total	excellent/ very good	good/ fair	poor	n.r.	total	estd. (00)	sample
(1)	(2)	(3)	(4)	(5)	(6)	(7)	(8)	(9)	(10)	(11)	(12)	(13)	(14)
Andhra Pradesh	351	23	573	384	20	1000	39	787	152	22	1000	18730	697
Arunachal Pradesh	259	156	379	292	173	1000	54	375	56	515	1000	238	140
Assam	373	10	616	313	62	1000	22	716	141	120	1000	6103	471
Bihar	187	5	559	424	13	1000	87	667	157	89	1000	18861	912
Chhattisgarh	165	0	527	473	0	1000	68	773	100	58	1000	4609	204
Delhi	-	-	-	-	-	-	93	742	166	0	1000	338	16
Goa	650	0	822	178	0	1000	468	402	129	0	1000	506	19
Gujarat	372	40	761	199	0	1000	95	807	90	9	1000	10458	351
Haryana	251	7	622	371	0	1000	87	802	109	2	1000	5608	252
Himachal Pradesh	298	25	559	416	0	1000	104	790	79	27	1000	2400	347
Jammu & Kashmir	340	1	504	496	0	1000	94	803	68	35	1000	2246	230
Jharkhand	96	59	613	276	51	1000	42	661	240	57	1000	6400	330
Karnataka	338	8	572	420	0	1000	136	780	74	10	1000	11642	428
Kerala	582	5	443	532	20	1000	24	787	189	0	1000	11673	505
Madhya Pradesh	232	9	521	471	0	1000	84	711	167	38	1000	14666	549
Maharashtra	319	16	671	287	26	1000	106	749	134	11	1000	23347	717
Manipur	74	52	104	655	189	1000	203	428	90	279	1000	395	211
Meghalaya	171	0	685	315	0	1000	192	733	24	51	1000	467	96
Mizoram	92	0	491	404	104	1000	117	283	46	554	1000	119	89
Nagaland	251	150	363	414	73	1000	187	620	107	86	1000	109	56
Orissa	167	0	495	473	32	1000	58	720	184	37	1000	13371	525
Punjab	314	36	755	209	0	1000	99	799	82	20	1000	6843	258
Rajasthan	169	14	555	431	0	1000	93	750	149	8	1000	12196	552
Sikkim	197	0	429	571	0	1000	170	726	93	11	1000	115	110
Tamil Nadu	291	25	724	249	1	1000	70	827	101	2	1000	16961	571
Tripura	277	19	786	150	45	1000	69	768	78	85	1000	927	171

Continued

For peer review only

For peer review only - http://bmjopen.bmj.com/site/about/guidelines.xhtml

Appendix A

Table (17.2): Per 1000 distribution of aged persons with illness or otherwise by their perception about current state of health for each sex

Rural Males

state/ut	no. per 1000 of aged persons reporting illness	aged person with illness own perception about current state of health					aged person without illness own perception about current state of health					no. of aged persons	
		excellent/ very good	good/ fair	poor	n.r.	total	excellent/ very good	good/ fair	poor	n.r.	total	estd. (00)	sample
(1)	(2)	(3)	(4)	(5)	(6)	(7)	(8)	(9)	(10)	(11)	(12)	(13)	(14)
Uttaranchal	165	34	425	541	0	1000	45	789	96	71	1000	2674	102
Uttar Pradesh	292	31	533	401	34	1000	65	690	131	114	1000	42788	1825
West Bengal	371	20	394	560	26	1000	80	611	182	126	1000	18130	680
A & N Islands	312	0	983	17	0	1000	142	858	0	0	1000	76	34
Chandigarh	508	0	195	805	0	1000	0	475	525	0	1000	7	17
Dadra & N. Haveli	13	0	0	1000	0	1000	129	825	46	0	1000	78	20
Daman & Diu	0	0	0	0	0	0	0	1000	0	0	1000	4	7
Lakshadweep	402	0	873	127	0	1000	0	951	49	0	1000	7	16
Pondicherry	532	0	762	238	0	1000	0	1000	0	0	1000	160	20
OENS	206	41	631	265	63	1000	132	621	67	179	1000	2371	873
GUTs	350	0	785	215	0	1000	80	894	26	0	1000	333	114
all-India	292	19	569	393	19	1000	77	731	139	53	1000	253254	11528
estd. no. of aged persons (00)	253254	1415	42171	29092	1391	74068	13773	130933	24989	9491	179186	X	X
sample aged persons	11528	61	1785	1485	70	3401	630	5782	1223	492	8127	X	X

For peer review only

For peer review only - http://bmjopen.bmj.com/site/about/guidelines.xhtml

Appendix A

Table (17.2): Per 1000 distribution of aged persons with illness or otherwise by their perception about current state of health for each sex

Rural Females

state/ut	no. per 1000 of aged persons reporting illness	aged person with illness: own perception about current state of health					aged person without illness: own perception about current state of health					no. of aged persons	
		excellent/ very good	good/ fair	poor	n.r.	total	excellent/ very good	good/ fair	poor	n.r.	total	estd. (00)	sample
(1)	(2)	(3)	(4)	(5)	(6)	(7)	(8)	(9)	(10)	(11)	(12)	(13)	(14)
Andhra Pradesh	375	9	449	516	26	1000	33	799	146	22	1000	20035	688
Arunachal Pradesh	221	0	261	430	310	1000	71	465	55	410	1000	204	118
Assam	416	10	398	510	82	1000	1	759	105	135	1000	4273	323
Bihar	159	1	448	479	72	1000	33	610	233	125	1000	15258	678
Chhattisgarh	148	47	392	524	38	1000	36	798	126	40	1000	6680	290
Delhi	-	-	-	-	-	-	0	947	53	0	1000	322	17
Goa	628	0	796	204	0	1000	183	614	203	0	1000	615	32
Gujarat	245	44	673	284	0	1000	37	879	83	1	1000	10401	370
Haryana	216	0	613	387	0	1000	44	815	141	0	1000	5510	232
Himachal Pradesh	266	8	530	450	13	1000	20	862	79	39	1000	2546	346
Jammu & Kashmir	356	0	249	748	3	1000	32	782	178	8	1000	1555	173
Jharkhand	70	0	681	297	22	1000	65	552	344	39	1000	4441	231
Karnataka	264	0	498	502	0	1000	42	862	83	12	1000	10878	403
Kerala	593	3	490	499	8	1000	16	684	244	55	1000	15013	649
Madhya Pradesh	202	55	457	461	26	1000	57	728	175	41	1000	14701	544
Maharashtra	305	2	633	331	34	1000	44	831	107	18	1000	24333	716
Manipur	91	0	457	543	0	1000	92	592	123	194	1000	279	163
Meghalaya	187	0	449	475	76	1000	82	722	136	60	1000	431	95
Mizoram	107	0	379	283	338	1000	71	393	53	483	1000	76	48
Nagaland	147	0	379	621	0	1000	0	610	278	112	1000	39	24
Orissa	162	6	394	600	0	1000	21	628	320	30	1000	12157	483
Punjab	422	10	589	396	5	1000	63	777	160	0	1000	6518	239
Rajasthan	134	28	430	543	0	1000	58	750	192	0	1000	12999	583
Sikkim	205	0	631	369	0	1000	43	770	87	100	1000	109	94
Tamil Nadu	301	52	720	227	0	1000	70	847	79	4	1000	17146	549
Tripura	337	0	641	347	12	1000	8	812	150	30	1000	675	135

Continued

For peer review only - http://bmjopen.bmj.com/site/about/guidelines.xhtml

Appendix A

Table (17.2): Per 1000 distribution of aged persons with illness or otherwise by their perception about current state of health for each sex

Rural | Females

state/ut	no. per 1000 of aged persons reporting illness	aged person with illness: own perception about current state of health					aged person without illness: own perception about current state of health					no. of aged persons	
		excellent/ very good	good/ fair	poor	n.r.	total	excellent/ very good	good/ fair	poor	n.r.	total	estd. (00)	sample
(1)	(2)	(3)	(4)	(5)	(6)	(7)	(8)	(9)	(10)	(11)	(12)	(13)	(14)
Uttaranchal	135	0	812	188	0	1000	0	791	197	11	1000	2190	90
Uttar Pradesh	273	17	454	487	42	1000	43	657	193	107	1000	41298	1649
West Bengal	386	4	437	510	49	1000	17	567	292	124	1000	18472	686
A & N Islands	199	0	367	633	0	1000	256	550	0	195	1000	43	15
Chandigarh	323	0	887	113	0	1000	0	507	493	0	1000	8	11
Dadra & N. Haveli	95	0	0	1000	0	1000	0	970	30	0	1000	39	10
Daman & Diu	155	0	818	182	0	1000	0	984	16	0	1000	13	13
Lakshadweep	617	0	954	46	0	1000	47	953	0	0	1000	9	22
Pondicherry	724	0	799	201	0	1000	0	1000	0	0	1000	148	18
OENS	229	0	542	397	62	1000	54	682	125	139	1000	1813	677
GUTs	498	0	756	244	0	1000	69	849	31	51	1000	261	89
all-India	286	14	511	448	26	1000	41	731	177	51	1000	249416	10737
estd. no. of aged persons (00)	249416	1035	36519	32011	1836	71401	7235	130155	31515	9110	178015	X	X
sample aged persons	10737	33	1528	1469	84	3114	316	5403	1449	455	7623	X	X

For peer review only - http://bmjopen.bmj.com/site/about/guidelines.xhtml

For peer review only

Appendix A

Table (17.2): Per 1000 distribution of aged persons with illness or otherwise by their perception about current state of health for each sex

Rural | Persons

state/ut	no. per 1000 of aged persons reporting illness	aged person with illness: own perception about current state of health					aged person without illness: own perception about current state of health					no. of aged persons	
		excellent/ very good	good/ fair	poor	n.r.	total	excellent/ very good	good/ fair	poor	n.r.	total	estd. (00)	sample
(1)	(2)	(3)	(4)	(5)	(6)	(7)	(8)	(9)	(10)	(11)	(12)	(13)	(14)
Andhra Pradesh	364	15	507	454	23	1000	36	793	149	22	1000	38765	1385
Arunachal Pradesh	242	90	329	350	231	1000	62	418	56	465	1000	442	258
Assam	391	10	521	399	70	1000	14	733	127	126	1000	10376	794
Bihar	174	3	514	446	37	1000	62	641	191	105	1000	34119	1590
Chhattisgarh	155	26	451	502	21	1000	49	788	116	48	1000	11289	494
Delhi	-	-	-	-	-	-	47	842	111	0	1000	661	33
Goa	638	0	808	192	0	1000	308	521	171	0	1000	1121	51
Gujarat	309	41	726	233	0	1000	63	846	86	5	1000	20859	721
Haryana	234	4	618	378	0	1000	65	809	125	1	1000	11118	484
Himachal Pradesh	282	16	545	433	6	1000	60	828	79	33	1000	4946	693
Jammu & Kashmir	346	0	397	602	1	1000	69	795	113	24	1000	3801	403
Jharkhand	85	40	636	283	41	1000	52	616	283	49	1000	10841	561
Karnataka	302	4	541	455	0	1000	88	822	79	11	1000	22520	831
Kerala	588	4	470	513	13	1000	20	730	220	31	1000	26687	1154
Madhya Pradesh	217	30	491	466	12	1000	70	719	171	39	1000	29367	1093
Maharashtra	312	9	652	309	30	1000	74	791	120	15	1000	47679	1433
Manipur	81	28	268	603	101	1000	158	495	103	244	1000	673	374
Meghalaya	179	0	566	395	38	1000	140	728	77	55	1000	898	191
Mizoram	98	0	443	353	204	1000	99	326	49	527	1000	196	137
Nagaland	223	124	366	450	60	1000	133	617	157	94	1000	148	80
Orissa	164	3	448	532	17	1000	40	676	249	34	1000	25527	1008
Punjab	366	21	661	314	3	1000	83	789	117	11	1000	13361	497
Rajasthan	151	20	498	482	0	1000	75	750	172	4	1000	25196	1135
Sikkim	201	0	529	471	0	1000	109	747	90	54	1000	224	204
Tamil Nadu	296	39	722	238	1	1000	70	837	90	3	1000	34107	1120
Tripura	302	10	718	243	29	1000	45	785	107	63	1000	1602	306

Continued

For peer review only - http://bmjopen.bmj.com/site/about/guidelines.xhtml

Appendix A

Table (17.2): Per 1000 distribution of aged persons with illness or otherwise by their perception about current state of health for each sex

Rural | Persons

state/ut	no. per 1000 of aged persons reporting illness	aged person with illness — own perception about current state of health					aged person without illness — own perception about current state of health					no. of aged persons	
		excellent/ very good	good/ fair	poor	n.r.	total	excellent/ very good	good/ fair	poor	n.r.	total	estd. (00)	sample
(1)	(2)	(3)	(4)	(5)	(6)	(7)	(8)	(9)	(10)	(11)	(12)	(13)	(14)
Uttaranchal	152	20	580	400	0	1000	24	790	142	43	1000	4865	192
Uttar Pradesh	283	25	495	442	38	1000	54	674	162	110	1000	84086	3474
West Bengal	379	12	416	534	38	1000	49	589	237	125	1000	36602	1366
A & N Islands	271	0	819	181	0	1000	187	736	0	77	1000	120	49
Chandigarh	409	0	487	513	0	1000	0	495	505	0	1000	16	28
Dadra & N. Haveli	40	0	0	1000	0	1000	89	871	41	0	1000	117	30
Daman & Diu	119	0	818	182	0	1000	0	988	12	0	1000	17	20
Lakshadweep	522	0	926	74	0	1000	21	952	27	0	1000	16	38
Pondicherry	624	0	782	218	0	1000	0	1000	0	0	1000	309	38
OENS	216	22	590	326	62	1000	99	647	92	162	1000	4184	1550
GUTs	415	0	770	230	0	1000	76	877	28	19	1000	594	203
all-India	289	17	541	420	22	1000	59	731	158	52	1000	502670	22265
estd. no. of aged persons (00)	502670	2450	78690	61103	3227	145469	21008	261088	56504	18600	357200	X	X
sample aged persons	22265	94	3313	2954	154	6515	946	11185	2672	947	15750	X	X

For peer review only - http://bmjopen.bmj.com/site/about/guidelines.xhtml

Appendix A

Table (17.2): Per 1000 distribution of aged persons with illness or otherwise by their perception about current state of health for each sex

Urban Males

state/ut	no. per 1000 of aged persons reporting illness	aged person with illness: own perception about current state of health					aged person without illness: own perception about current state of health					no. of aged persons	
		excellent/ very good	good/ fair	poor	n.r.	total	excellent/ very good	good/ fair	poor	n.r.	total	estd. (00)	sample
(1)	(2)	(3)	(4)	(5)	(6)	(7)	(8)	(9)	(10)	(11)	(12)	(13)	(14)
Andhra Pradesh	524	10	665	312	13	1000	114	795	47	44	1000	5310	377
Arunachal Pradesh	60	0	158	526	316	1000	47	291	85	577	1000	10	42
Assam	401	33	525	359	83	1000	24	798	74	104	1000	647	109
Bihar	247	14	513	473	0	1000	106	658	138	99	1000	2455	169
Chhattisgarh	292	260	249	491	0	1000	102	674	224	0	1000	641	63
Delhi	136	0	663	337	0	1000	113	768	103	16	1000	2212	211
Goa	163	0	156	844	0	1000	284	691	25	0	1000	229	32
Gujarat	385	50	700	250	0	1000	160	791	49	0	1000	4592	297
Haryana	325	50	608	341	0	1000	165	738	87	10	1000	1315	132
Himachal Pradesh	317	0	640	360	0	1000	17	983	0	0	1000	139	49
Jammu & Kashmir	388	0	627	373	0	1000	143	672	173	11	1000	408	102
Jharkhand	204	0	606	394	0	1000	123	742	118	16	1000	1372	166
Karnataka	292	20	652	319	9	1000	105	803	73	19	1000	4025	345
Kerala	537	46	552	386	16	1000	153	749	78	20	1000	3986	290
Madhya Pradesh	251	0	504	496	0	1000	165	642	147	47	1000	4115	319
Maharashtra	409	30	683	280	6	1000	105	735	131	30	1000	12844	596
Manipur	42	0	75	925	0	1000	115	542	79	264	1000	192	116
Meghalaya	55	0	788	0	212	1000	135	839	18	8	1000	42	34
Mizoram	123	0	460	239	301	1000	42	367	17	574	1000	85	142
Nagaland	255	162	712	125	0	1000	80	727	24	169	1000	68	24
Orissa	152	62	329	609	0	1000	70	783	113	34	1000	1546	148
Punjab	296	154	545	253	48	1000	126	758	102	15	1000	2565	165
Rajasthan	290	9	412	579	0	1000	118	709	171	2	1000	3012	271
Sikkim	29	0	556	444	0	1000	67	907	27	0	1000	11	16
Tamil Nadu	311	14	858	128	0	1000	113	828	59	0	1000	7955	587
Tripura	294	0	975	25	0	1000	13	779	159	49	1000	130	45

Continued

For peer review only - http://bmjopen.bmj.com/site/about/guidelines.xhtml

For peer review only

Appendix A

Table (17.2): Per 1000 distribution of aged persons with illness or otherwise by their perception about current state of health for each sex

Urban Males

state/ut	no. per 1000 of aged persons reporting illness	aged person with illness own perception about current state of health					aged person without illness own perception about current state of health					no. of aged persons	
		excellent/ very good	good/ fair	poor	n.r.	total	excellent/ very good	good/ fair	poor	n.r.	total	estd. (00)	sample
(1)	(2)	(3)	(4)	(5)	(6)	(7)	(8)	(9)	(10)	(11)	(12)	(13)	(14)
Uttaranchal	338	0	832	168	0	1000	218	776	6	0	1000	558	49
Uttar Pradesh	305	18	574	386	21	1000	51	740	141	68	1000	9104	620
West Bengal	495	37	626	321	16	1000	94	706	123	76	1000	8725	522
A & N Islands	348	0	861	139	0	1000	164	770	21	45	1000	18	18
Chandigarh	181	112	876	12	0	1000	47	879	75	0	1000	197	69
Dadra & N. Haveli	9	0	0	1000	0	1000	86	894	19	0	1000	4	13
Daman & Diu	201	0	534	466	0	1000	117	678	205	0	1000	10	16
Lakshadweep	492	0	512	488	0	1000	0	1000	0	0	1000	8	21
Pondicherry	599	0	450	550	0	1000	129	830	41	0	1000	218	47
OENS	144	37	740	174	49	1000	78	610	72	239	1000	538	419
GUTs	393	22	552	426	0	1000	80	855	63	2	1000	455	184
all-India	360	31	635	324	11	1000	110	747	107	36	1000	78751	6222
estd. no. of aged persons (00)	78751	873	17988	9173	307	28341	5534	37672	5396	1808	50410	X	X
sample aged persons	6222	50	1264	749	34	2097	448	3014	429	234	4125	X	X

For peer review only - http://bmjopen.bmj.com/site/about/guidelines.xhtml

Appendix A

Table (17.2): Per 1000 distribution of aged persons with illness or otherwise by their perception about current state of health for each sex

Urban Females

state/ut	no. per 1000 of aged persons reporting illness	aged person with illness own perception about current state of health					aged person without illness own perception about current state of health					no. of aged persons	
		excellent/ very good	good/ fair	poor	n.r.	total	excellent/ very good	good/ fair	poor	n.r.	total	estd. (00)	sample
(1)	(2)	(3)	(4)	(5)	(6)	(7)	(8)	(9)	(10)	(11)	(12)	(13)	(14)
Andhra Pradesh	565	51	631	302	16	1000	9	879	83	29	1000	6160	421
Arunachal Pradesh	211	43	559	269	129	1000	81	293	48	578	1000	7	30
Assam	322	0	629	347	24	1000	0	742	125	134	1000	532	85
Bihar	252	0	565	421	14	1000	74	646	191	89	1000	1829	145
Chhattisgarh	229	0	573	427	0	1000	30	765	206	0	1000	760	88
Delhi	155	0	669	322	10	1000	93	782	112	13	1000	2129	207
Goa	420	0	376	624	0	1000	129	654	217	0	1000	256	40
Gujarat	297	12	683	305	0	1000	87	843	57	12	1000	5101	319
Haryana	258	17	656	327	0	1000	151	683	123	43	1000	1725	143
Himachal Pradesh	311	0	969	0	31	1000	15	909	76	0	1000	155	50
Jammu & Kashmir	317	0	613	369	17	1000	58	792	140	10	1000	336	91
Jharkhand	113	0	327	627	46	1000	37	653	282	28	1000	1292	139
Karnataka	372	0	622	378	0	1000	89	770	134	7	1000	4073	353
Kerala	601	23	574	403	0	1000	72	698	176	55	1000	4686	322
Madhya Pradesh	257	23	469	487	21	1000	72	686	186	56	1000	4466	327
Maharashtra	440	21	681	284	15	1000	96	775	111	18	1000	13669	672
Manipur	90	0	654	210	136	1000	82	465	108	346	1000	178	115
Meghalaya	218	0	607	393	0	1000	209	631	118	41	1000	48	46
Mizoram	124	0	296	275	429	1000	53	471	14	462	1000	60	104
Nagaland	0	0	0	0	0	0	735	265	0	0	1000	8	5
Orissa	211	32	624	344	0	1000	49	671	174	105	1000	1182	113
Punjab	341	39	607	354	0	1000	66	767	152	16	1000	2347	164
Rajasthan	257	0	541	459	0	1000	84	690	226	0	1000	3076	266
Sikkim	262	0	481	519	0	1000	0	1000	0	0	1000	7	13
Tamil Nadu	353	15	774	210	0	1000	80	848	71	1	1000	8984	650
Tripura	171	64	706	230	0	1000	11	669	221	100	1000	136	54

Continued

For peer review only - http://bmjopen.bmj.com/site/about/guidelines.xhtml

Appendix A

Table (17.2): Per 1000 distribution of aged persons with illness or otherwise by their perception about current state of health for each sex

Urban | Females

state/ut	no. per 1000 of aged persons reporting illness	aged person with illness: own perception about current state of health					aged person without illness: own perception about current state of health					no. of aged persons	
		excellent/ very good	good/ fair	poor	n.r.	total	excellent/ very good	good/ fair	poor	n.r.	total	estd. (00)	sample
(1)	(2)	(3)	(4)	(5)	(6)	(7)	(8)	(9)	(10)	(11)	(12)	(13)	(14)
Uttaranchal	156	0	197	803	0	1000	0	902	60	38	1000	693	49
Uttar Pradesh	377	10	500	443	46	1000	43	676	202	79	1000	9590	621
West Bengal	550	12	541	419	28	1000	31	697	212	59	1000	8315	506
A & N Islands	143	346	375	279	0	1000	158	674	101	67	1000	15	22
Chandigarh	364	0	739	261	0	1000	80	854	50	16	1000	147	68
Dadra & N. Haveli	86	0	0	1000	0	1000	0	745	255	0	1000	3	14
Daman & Diu	56	0	1000	0	0	1000	66	573	316	44	1000	16	22
Lakshadweep	582	0	747	253	0	1000	245	696	59	0	1000	10	24
Pondicherry	683	0	508	492	0	1000	29	971	0	0	1000	368	56
OENS	136	26	614	268	92	1000	81	543	125	252	1000	444	367
GUTs	562	2	552	446	0	1000	61	879	48	12	1000	560	206
all-India	390	19	611	355	15	1000	69	753	143	36	1000	82359	6344
estd. no. of aged persons (00)	82359	601	19637	11426	495	32159	3456	37785	7169	1790	50200	X	X
sample aged persons	6344	38	1399	848	43	2328	252	2962	564	238	4016	X	X

For peer review only - http://bmjopen.bmj.com/site/about/guidelines.xhtml

Appendix A

Table (17.2): Per 1000 distribution of aged persons with illness or otherwise by their perception about current state of health for each sex

Urban — Persons

state/ut	no. per 1000 of aged persons reporting illness	aged person with illness: own perception about current state of health					aged person without illness: own perception about current state of health					no. of aged persons	
		excellent/ very good	good/ fair	poor	n.r.	total	excellent/ very good	good/ fair	poor	n.r.	total	estd. (00)	sample
(1)	(2)	(3)	(4)	(5)	(6)	(7)	(8)	(9)	(10)	(11)	(12)	(13)	(14)
Andhra Pradesh	546	33	646	307	15	1000	60	838	65	36	1000	11470	798
Arunachal Pradesh	122	31	443	344	183	1000	60	292	71	577	1000	17	72
Assam	365	20	566	354	60	1000	12	771	98	118	1000	1180	194
Bihar	249	8	535	450	6	1000	92	653	161	95	1000	4283	314
Chhattisgarh	258	135	405	460	0	1000	61	725	214	0	1000	1401	151
Delhi	145	0	666	329	5	1000	103	775	107	15	1000	4342	418
Goa	299	0	319	681	0	1000	216	675	109	0	1000	485	72
Gujarat	339	32	692	275	0	1000	119	820	54	7	1000	9693	616
Haryana	287	33	633	334	0	1000	157	705	108	30	1000	3040	275
Himachal Pradesh	314	0	812	172	16	1000	16	944	40	0	1000	294	99
Jammu & Kashmir	356	0	622	372	7	1000	102	730	157	11	1000	744	193
Jharkhand	160	0	510	474	16	1000	79	696	202	22	1000	2664	305
Karnataka	332	9	635	352	4	1000	97	787	102	13	1000	8098	698
Kerala	572	33	564	396	7	1000	112	723	127	38	1000	8672	612
Madhya Pradesh	254	12	486	491	11	1000	117	665	167	52	1000	8580	646
Maharashtra	425	25	682	282	11	1000	100	755	121	24	1000	26513	1268
Manipur	65	0	461	449	90	1000	100	506	92	302	1000	369	231
Meghalaya	141	0	640	321	39	1000	171	739	66	24	1000	90	80
Mizoram	123	0	391	254	355	1000	46	410	16	528	1000	145	246
Nagaland	229	162	712	125	0	1000	166	666	21	147	1000	76	29
Orissa	177	47	481	472	0	1000	61	737	138	64	1000	2729	261
Punjab	317	95	577	305	24	1000	98	762	125	15	1000	4912	329
Rajasthan	274	5	473	522	0	1000	101	699	199	1	1000	6088	537
Sikkim	122	0	492	508	0	1000	44	938	18	0	1000	18	29
Tamil Nadu	333	15	811	174	0	1000	96	838	66	0	1000	16939	1237
Tripura	231	24	873	102	0	1000	12	718	193	77	1000	267	99

Continued

For peer review only - http://bmjopen.bmj.com/site/about/guidelines.xhtml

For peer review only

Appendix A

Table (17.2): Per 1000 distribution of aged persons with illness or otherwise by their perception about current state of health for each sex

Urban Persons

state/ut	no. per 1000 of aged persons reporting illness	aged person with illness: own perception about current state of health					aged person without illness: own perception about current state of health					no. of aged persons	
		excellent/ very good	good/ fair	poor	n.r.	total	excellent/ very good	good/ fair	poor	n.r.	total	estd. (00)	sample
(1)	(2)	(3)	(4)	(5)	(6)	(7)	(8)	(9)	(10)	(11)	(12)	(13)	(14)
Uttaranchal	237	0	600	400	0	1000	84	853	39	23	1000	1252	98
Uttar Pradesh	342	14	532	419	35	1000	47	709	170	73	1000	18694	1241
West Bengal	522	24	582	371	22	1000	66	702	164	69	1000	17040	1028
A & N Islands	254	88	737	174	0	1000	161	719	63	57	1000	33	40
Chandigarh	259	45	794	162	0	1000	59	870	65	6	1000	344	137
Dadra & N. Haveli	45	0	0	1000	0	1000	47	827	126	0	1000	7	27
Daman & Diu	112	0	678	322	0	1000	84	609	278	29	1000	26	38
Lakshadweep	542	0	653	347	0	1000	125	845	30	0	1000	19	45
Pondicherry	652	0	488	512	0	1000	72	911	18	0	1000	586	103
OENS	140	32	685	215	68	1000	80	580	96	245	1000	983	786
GUTs	486	10	552	439	0	1000	71	866	56	7	1000	1015	390
all-India	376	24	622	340	13	1000	89	750	125	36	1000	161110	12566
estd. no. of aged persons (00)	161110	1474	37625	20599	802	60500	8990	75457	12565	3598	100610	X	X
sample aged persons	12566	88	2663	1597	77	4425	700	5976	993	472	8141	X	X

For peer review only - http://bmjopen.bmj.com/site/about/guidelines.xhtml

Appendix A

Table (17.2): Per 1000 distribution of aged persons with illness or otherwise by their perception about current state of health for each sex

Rural + Urban Males

state/ut	no. per 1000 of aged persons reporting illness	aged person with illness: own perception about current state of health					aged person without illness: own perception about current state of health					no. of aged persons	
		excellent/ very good	good/ fair	poor	n.r.	total	excellent/ very good	good/ fair	poor	n.r.	total	estd. (00)	sample
(1)	(2)	(3)	(4)	(5)	(6)	(7)	(8)	(9)	(10)	(11)	(12)	(13)	(14)
Andhra Pradesh	389	19	600	363	18	1000	52	789	134	26	1000	24041	1074
Arunachal Pradesh	251	154	377	295	174	1000	54	371	57	518	1000	248	182
Assam	375	12	607	317	64	1000	23	724	135	119	1000	6750	580
Bihar	194	6	552	431	11	1000	89	666	155	90	1000	21316	1081
Chhattisgarh	181	51	472	476	0	1000	72	763	114	52	1000	5250	267
Delhi	118	0	663	337	0	1000	110	764	112	13	1000	2551	227
Goa	498	0	754	246	0	1000	372	552	75	0	1000	735	51
Gujarat	376	43	742	215	0	1000	114	802	77	6	1000	15050	648
Haryana	265	17	619	364	0	1000	101	791	105	3	1000	6923	384
Himachal Pradesh	299	23	564	413	0	1000	99	801	74	26	1000	2539	396
Jammu & Kashmir	347	0	525	475	0	1000	101	784	83	31	1000	2654	332
Jharkhand	115	41	611	313	35	1000	55	674	220	51	1000	7772	496
Karnataka	326	11	591	397	2	1000	127	786	74	13	1000	15667	773
Kerala	570	15	469	497	19	1000	60	776	159	6	1000	15659	795
Madhya Pradesh	236	7	517	477	0	1000	102	696	163	40	1000	18781	868
Maharashtra	351	22	676	284	18	1000	106	744	133	17	1000	36191	1313
Manipur	63	41	97	714	148	1000	174	466	86	274	1000	587	327
Meghalaya	161	0	688	306	6	1000	187	743	23	47	1000	510	130
Mizoram	105	0	476	324	200	1000	86	317	34	562	1000	204	231
Nagaland	252	155	499	302	44	1000	146	661	75	118	1000	178	80
Orissa	165	6	479	486	29	1000	59	727	177	37	1000	14917	673
Punjab	309	67	700	221	13	1000	107	787	88	18	1000	9408	423
Rajasthan	193	12	513	475	0	1000	97	743	153	7	1000	15208	823
Sikkim	183	0	430	570	0	1000	160	744	87	10	1000	126	126
Tamil Nadu	297	22	769	209	1	1000	83	827	88	1	1000	24916	1158
Tripura	279	16	811	134	39	1000	62	769	88	80	1000	1057	216

Continued

For peer review only - http://bmjopen.bmj.com/site/about/guidelines.xhtml

Appendix A

Table (17.2): Per 1000 distribution of aged persons with illness or otherwise by their perception about current state of health for each sex

Rural + Urban Males

state/ut	no. per 1000 of aged persons reporting illness	aged person with illness: own perception about current state of health					aged person without illness: own perception about current state of health					no. of aged persons	
		excellent/ very good	good/ fair	poor	n.r.	total	excellent/ very good	good/ fair	poor	n.r.	total	estd. (00)	sample
(1)	(2)	(3)	(4)	(5)	(6)	(7)	(8)	(9)	(10)	(11)	(12)	(13)	(14)
Uttaranchal	195	24	547	430	0	1000	69	787	83	61	1000	3232	151
Uttar Pradesh	294	29	541	399	32	1000	63	699	133	106	1000	51892	2445
West Bengal	411	27	485	467	22	1000	84	638	166	112	1000	26855	1202
A & N Islands	319	0	958	42	0	1000	146	842	4	8	1000	94	52
Chandigarh	192	101	812	87	0	1000	46	870	84	0	1000	205	86
Dadra & N. Haveli	13	0	0	1000	0	1000	127	828	45	0	1000	82	33
Daman & Diu	142	0	534	466	0	1000	77	788	135	0	1000	14	23
Lakshadweep	451	0	662	338	0	1000	0	975	25	0	1000	15	37
Pondicherry	571	0	573	427	0	1000	70	908	22	0	1000	379	67
OENS	194	40	646	253	61	1000	121	619	68	191	1000	2909	1292
GUTs	375	13	644	343	0	1000	80	872	47	1	1000	789	298
all-India	308	22	587	374	17	1000	84	734	132	49	1000	332005	17750
estd. no. of aged persons (00)	332005	2288	60159	38265	1698	102409	19307	168605	30385	11298	229596	X	X
sample aged persons	17750	111	3049	2234	104	5498	1078	8796	1652	726	12252	X	X

For peer review only

For peer review only - http://bmjopen.bmj.com/site/about/guidelines.xhtml

Appendix A

Table (17.2): Per 1000 distribution of aged persons with illness or otherwise by their perception about current state of health for each sex

Rural + Urban Females

state/ut	no. per 1000 of aged persons reporting illness	aged person with illness own perception about current state of health					aged person without illness own perception about current state of health					no. of aged persons	
		excellent/ very good	good/ fair	poor	n.r.	total	excellent/ very good	good/ fair	poor	n.r.	total	estd. (00)	sample
(1)	(2)	(3)	(4)	(5)	(6)	(7)	(8)	(9)	(10)	(11)	(12)	(13)	(14)
Andhra Pradesh	420	22	507	448	23	1000	29	813	135	23	1000	26195	1109
Arunachal Pradesh	221	1	270	425	304	1000	71	459	55	415	1000	211	148
Assam	406	9	418	496	77	1000	1	757	107	135	1000	4806	408
Bihar	168	1	467	470	62	1000	37	613	229	121	1000	17087	823
Chhattisgarh	156	40	419	509	32	1000	35	795	133	37	1000	7440	378
Delhi	134	0	669	322	10	1000	79	807	103	11	1000	2452	224
Goa	567	0	705	295	0	1000	162	630	209	0	1000	871	72
Gujarat	262	32	676	292	0	1000	52	868	75	5	1000	15501	689
Haryana	226	5	625	370	0	1000	68	785	137	10	1000	7235	375
Himachal Pradesh	269	7	559	420	14	1000	20	864	79	37	1000	2701	396
Jammu & Kashmir	349	0	308	687	5	1000	36	784	171	9	1000	1891	264
Jharkhand	79	0	567	403	29	1000	59	574	330	36	1000	5733	370
Karnataka	293	0	541	459	0	1000	53	840	96	11	1000	14951	756
Kerala	595	8	510	476	6	1000	29	688	228	55	1000	19699	971
Madhya Pradesh	215	46	461	468	25	1000	60	718	177	44	1000	19166	871
Maharashtra	353	10	654	310	25	1000	60	814	108	18	1000	38001	1388
Manipur	91	0	533	414	52	1000	88	542	117	253	1000	456	278
Meghalaya	190	0	467	466	67	1000	94	714	134	58	1000	479	141
Mizoram	114	0	339	279	382	1000	63	427	36	474	1000	137	152
Nagaland	123	0	379	621	0	1000	138	545	225	91	1000	47	29
Orissa	166	9	420	571	0	1000	23	632	308	37	1000	13339	596
Punjab	400	17	593	387	4	1000	64	774	158	5	1000	8865	403
Rajasthan	158	19	464	517	0	1000	63	740	197	0	1000	16075	849
Sikkim	208	0	619	381	0	1000	41	783	82	94	1000	116	107
Tamil Nadu	319	38	741	221	0	1000	73	848	77	3	1000	26130	1199
Tripura	309	6	647	336	11	1000	9	783	164	44	1000	812	189

Continued

For peer review only

For peer review only - http://bmjopen.bmj.com/site/about/guidelines.xhtml

Appendix A

Table (17.2): Per 1000 distribution of aged persons with illness or otherwise by their perception about current state of health for each sex

Rural + Urban | Females

state/ut	no. per 1000 of aged persons reporting illness	aged person with illness: own perception about current state of health					aged person without illness: own perception about current state of health					no. of aged persons	
		excellent/ very good	good/ fair	poor	n.r.	total	excellent/ very good	good/ fair	poor	n.r.	total	estd. (00)	sample
(1)	(2)	(3)	(4)	(5)	(6)	(7)	(8)	(9)	(10)	(11)	(12)	(13)	(14)
Uttaranchal	140	0	647	353	0	1000	0	817	165	18	1000	2884	139
Uttar Pradesh	293	16	465	477	43	1000	43	661	195	102	1000	50887	2270
West Bengal	437	7	477	474	41	1000	21	599	272	108	1000	26787	1192
A & N Islands	185	69	369	563	0	1000	229	583	27	160	1000	58	37
Chandigarh	361	0	746	254	0	1000	76	834	75	15	1000	155	79
Dadra & N. Haveli	94	0	0	1000	0	1000	0	952	48	0	1000	42	24
Daman & Diu	102	0	872	128	0	1000	38	751	186	25	1000	29	35
Lakshadweep	598	0	846	154	0	1000	158	809	33	0	1000	19	46
Pondicherry	695	0	595	405	0	1000	21	979	0	0	1000	516	74
OENS	211	3	551	380	65	1000	60	652	125	163	1000	2257	1044
GUTs	541	2	612	387	0	1000	64	869	42	26	1000	821	295
all-India	312	16	542	419	23	1000	47	736	170	48	1000	331775	17081
estd. no. of aged persons (00)	331775	1636	56156	43437	2331	103560	10691	167940	38684	10899	228214	X	X
sample aged persons	17081	71	2927	2317	127	5442	568	8365	2013	693	11639	X	X

For peer review only - http://bmjopen.bmj.com/site/about/guidelines.xhtml

Appendix A

Table (17.2): Per 1000 distribution of aged persons with illness or otherwise by their perception about current state of health for each sex

Rural + Urban Persons

state/ut	no. per 1000 of aged persons reporting illness	aged person with illness					aged person without illness					no. of aged persons	
		own perception about current state of health					own perception about current state of health						
		excellent/ very good	good/ fair	poor	n.r.	total	excellent/ very good	good/ fair	poor	n.r.	total	estd. (00)	sample
(1)	(2)	(3)	(4)	(5)	(6)	(7)	(8)	(9)	(10)	(11)	(12)	(13)	(14)
Andhra Pradesh	405	21	550	409	21	1000	40	801	134	25	1000	50236	2183
Arunachal Pradesh	237	89	331	350	230	1000	62	412	56	470	1000	460	330
Assam	388	11	525	395	69	1000	14	737	124	125	1000	11556	988
Bihar	183	4	517	447	32	1000	65	642	188	104	1000	38403	1904
Chhattisgarh	166	45	443	495	18	1000	50	782	125	43	1000	12690	645
Delhi	126	0	666	329	5	1000	95	785	108	12	1000	5002	451
Goa	536	0	726	274	0	1000	266	591	143	0	1000	1606	123
Gujarat	318	38	715	247	0	1000	80	838	76	5	1000	30551	1337
Haryana	245	11	622	367	0	1000	84	788	122	7	1000	14158	759
Himachal Pradesh	283	15	562	416	7	1000	58	834	77	32	1000	5240	792
Jammu & Kashmir	348	0	434	563	2	1000	74	784	120	22	1000	4545	596
Jharkhand	100	27	596	343	33	1000	57	631	268	44	1000	13505	866
Karnataka	310	6	568	426	1	1000	90	813	85	12	1000	30618	1529
Kerala	584	11	492	485	12	1000	43	728	196	32	1000	35359	1766
Madhya Pradesh	226	26	490	473	12	1000	80	707	170	42	1000	37947	1739
Maharashtra	352	16	665	297	22	1000	82	780	120	18	1000	74193	2701
Manipur	75	19	327	556	98	1000	137	499	99	265	1000	1043	605
Meghalaya	175	0	572	390	38	1000	143	729	76	52	1000	988	271
Mizoram	109	0	418	305	277	1000	77	361	35	527	1000	341	383
Nagaland	225	137	486	338	39	1000	144	634	111	112	1000	224	109
Orissa	165	7	451	526	15	1000	42	682	239	37	1000	28256	1269
Punjab	353	39	641	312	8	1000	87	781	119	12	1000	18274	826
Rajasthan	175	15	490	494	0	1000	79	741	176	4	1000	31284	1672
Sikkim	195	0	527	473	0	1000	104	763	84	50	1000	242	233
Tamil Nadu	308	30	754	215	0	1000	78	838	82	2	1000	51046	2357
Tripura	292	12	736	227	26	1000	40	775	120	65	1000	1869	405

Continued

For peer review only - http://bmjopen.bmj.com/site/about/guidelines.xhtml

Appendix A

Table (17.2): Per 1000 distribution of aged persons with illness or otherwise by their perception about current state of health for each sex

Rural + Urban Persons

state/ut	no. per 1000 of aged persons reporting illness	aged person with illness					aged person without illness					no. of aged persons	
		own perception about current state of health					own perception about current state of health						
		excellent/ very good	good/ fair	poor	n.r.	total	excellent/ very good	good/ fair	poor	n.r.	total	estd. (00)	sample
(1)	(2)	(3)	(4)	(5)	(6)	(7)	(8)	(9)	(10)	(11)	(12)	(13)	(14)
Uttaranchal	169	14	586	400	0	1000	35	802	123	40	1000	6116	290
Uttar Pradesh	293	22	503	437	37	1000	53	680	163	104	1000	102779	4715
West Bengal	424	17	481	471	32	1000	53	619	218	110	1000	53642	2394
A & N Islands	268	18	802	179	0	1000	181	732	14	73	1000	152	89
Chandigarh	265	42	773	185	0	1000	57	857	81	6	1000	360	165
Dadra & N. Haveli	41	0	0	1000	0	1000	86	868	46	0	1000	124	57
Daman & Diu	115	0	737	263	0	1000	50	763	170	17	1000	43	58
Lakshadweep	533	0	777	223	0	1000	76	896	28	0	1000	35	83
Pondicherry	642	0	587	413	0	1000	46	943	11	0	1000	895	141
OENS	202	23	602	311	63	1000	95	634	93	179	1000	5167	2336
GUTs	460	6	624	369	0	1000	73	871	45	12	1000	1609	593
all-India	310	19	565	397	20	1000	66	735	151	48	1000	663779	34831
estd. no. of aged persons (00)	663779	3924	116315	81702	4029	205970	29998	336545	69069	22198	457810	X	X
sample aged persons	34831	182	5976	4551	231	10940	1646	17161	3665	1419	23891	X	X

For peer review only - http://bmjopen.bmj.com/site/about/guidelines.xhtml

Appendix A

Table (18.1): Per 1000 distribution of aged persons with illness or otherwise by their perception about change in state of health for each quintile class and sex

all-India | Rural | Males

mpce class	aged person with illness							aged person without illness							no. of aged persons	
	compared to previous year							compared to previous year							estd. (00)	sample
	much better	some-what better	nearly the same	some-what worse	worse	n.r.	total	much better	some what better	nearly the same	some what worse	worse	n.r.	total		
(1)	(2)	(3)	(4)	(5)	(6)	(7)	(8)	(9)	(10)	(11)	(12)	(13)	(14)	(15)	(16)	(17)
0 – 225	9	85	485	315	69	37	1000	42	119	637	103	10	89	1000	9537	399
225 – 255	37	115	453	288	25	81	1000	30	63	698	125	26	58	1000	8411	302
255 – 300	30	81	451	317	83	38	1000	39	102	693	92	16	59	1000	20867	805
300 – 340	1	28	549	345	75	1	1000	23	101	676	120	16	64	1000	17295	744
340 – 380	6	70	557	337	23	7	1000	26	83	690	121	13	66	1000	22031	964
380 – 420	56	81	469	325	56	13	1000	47	127	658	104	16	48	1000	24050	1046
420 – 470	40	91	489	279	66	34	1000	28	95	729	107	7	34	1000	26188	1184
470 – 525	10	72	460	372	54	31	1000	30	102	663	128	5	72	1000	27442	1281
525 – 615	16	92	505	312	58	17	1000	61	98	684	107	2	47	1000	31708	1492
615 – 775	19	90	521	319	38	13	1000	31	106	693	110	6	54	1000	32712	1576
775 – 950	30	125	504	294	33	13	1000	47	148	709	82	4	9	1000	14637	810
950 +	29	109	489	339	34	0	1000	39	155	694	71	7	34	1000	18376	925
all classes	24	88	497	322	50	19	1000	37	106	686	108	10	53	1000	253254	11528
estd. no. of aged persons(00)	1770	6482	36817	23875	3717	1408	74068	6685	19051	122990	19280	1732	9447	179186	X	X
sample aged persons	70	336	1576	1109	237	73	3401	313	935	5328	964	94	493	8127	X	X

For peer review only - http://bmjopen.bmj.com/site/about/guidelines.xhtml

Appendix A

Table (18.1): Per 1000 distribution of aged persons with illness or otherwise by their perception about change in state of health for each quintile class and sex

all-India | Rural | Females

mpce class	aged person with illness							aged person without illness							no. of aged persons	
	compared to previous year							compared to previous year							estd. (00)	sample
	much better	some-what better	nearly the same	some-what worse	worse	n.r.	total	much better	some what better	nearly the same	some what worse	worse	n.r.	total		
(1)	(2)	(3)	(4)	(5)	(6)	(7)	(8)	(9)	(10)	(11)	(12)	(13)	(14)	(15)	(16)	(17)
0 – 225	15	48	539	348	46	4	1000	21	150	609	145	11	65	1000	11222	415
225 – 255	0	93	521	335	52	0	1000	24	66	617	167	34	92	1000	8828	320
255 – 300	32	58	422	356	105	27	1000	20	101	668	143	10	59	1000	21088	797
300 – 340	14	27	532	288	109	30	1000	16	115	685	119	4	60	1000	17861	716
340 – 380	25	89	469	312	64	41	1000	16	75	691	147	20	52	1000	22651	891
380 – 420	18	56	530	287	81	28	1000	20	91	679	129	18	63	1000	22017	930
420 – 470	23	59	542	263	53	59	1000	20	79	704	130	15	52	1000	24398	1065
470 – 525	19	49	516	311	84	20	1000	26	84	665	162	13	49	1000	27514	1214
525 – 615	5	74	513	326	57	26	1000	47	91	659	150	12	42	1000	28621	1308
615 – 775	26	78	507	328	36	24	1000	25	105	684	139	4	44	1000	29982	1407
775 – 950	23	67	512	350	30	17	1000	33	147	659	131	10	20	1000	16256	776
950 +	4	118	527	312	31	8	1000	28	153	641	129	13	36	1000	18978	898
all classes	18	71	511	315	60	26	1000	25	100	669	141	13	51	1000	249416	10737
estd. no. of aged persons(00)	1257	5060	36465	22515	4268	1836	71401	4459	17874	119180	25104	2266	9131	178015	X	X
sample aged persons	58	234	1499	1023	216	84	3114	208	737	4951	1146	122	459	7623	X	X

For peer review only - http://bmjopen.bmj.com/site/about/guidelines.xhtml

Appendix A

Table (18.1): Per 1000 distribution of aged persons with illness or otherwise by their perception about change in state of health for each quintile class and sex

all-India Rural Persons

mpce class	aged person with illness							aged person without illness							no. of aged persons	
	compared to previous year							compared to previous year							estd. (00)	sample
	much better	some-what better	nearly the same	some-what worse	worse	n.r.	total	much better	some what better	nearly the same	some what worse	worse	n.r.	total		
(1)	(2)	(3)	(4)	(5)	(6)	(7)	(8)	(9)	(10)	(11)	(12)	(13)	(14)	(15)	(16)	(17)
0 – 225	12	68	510	330	58	22	1000	30	136	621	126	10	75	1000	20759	814
225 – 255	18	104	487	312	39	40	1000	27	65	656	147	30	75	1000	17239	622
255 – 300	31	70	436	336	94	32	1000	29	101	680	118	13	59	1000	41955	1602
300 – 340	8	28	540	314	94	17	1000	20	108	681	120	10	62	1000	35156	1460
340 – 380	16	80	510	324	45	25	1000	21	79	691	134	17	59	1000	44682	1855
380 – 420	38	70	497	308	67	20	1000	34	109	668	116	17	55	1000	46067	1976
420 – 470	33	77	513	272	60	45	1000	24	87	717	118	11	43	1000	50586	2249
470 – 525	14	61	488	342	69	26	1000	28	93	664	145	9	61	1000	54956	2495
525 – 615	11	83	509	318	58	21	1000	54	95	672	128	7	45	1000	60329	2800
615 – 775	22	84	515	323	37	18	1000	28	105	689	124	5	49	1000	62693	2983
775 – 950	26	95	508	324	32	15	1000	40	147	682	108	7	15	1000	30893	1586
950 +	16	113	509	325	32	4	1000	33	154	667	100	10	35	1000	37355	1823
all classes	21	79	504	319	55	22	1000	31	103	678	124	11	52	1000	502670	22265
estd. no. of aged persons(00)	3027	11542	73283	46390	7984	3244	145469	11144	36925	242171	44384	3998	18578	357200	X	X
sample aged persons	128	570	3075	2132	453	157	6515	521	1672	10279	2110	216	952	15750	X	X

For peer review only - http://bmjopen.bmj.com/site/about/guidelines.xhtml

Appendix A

Table (18.1): Per 1000 distribution of aged persons with illness or otherwise by their perception about change in state of health for each quintile class and sex

all-India Urban Males

mpce class	aged person with illness							aged person without illness							no. of aged persons	
	compared to previous year							compared to previous year							estd. (00)	sample
	much better	some-what better	nearly the same	some-what worse	worse	n.r.	total	much better	some what better	nearly the same	some what worse	worse	n.r.	total		
(1)	(2)	(3)	(4)	(5)	(6)	(7)	(8)	(9)	(10)	(11)	(12)	(13)	(14)	(15)	(16)	(17)
0 – 225	17	25	565	297	96	0	1000	6	106	567	210	38	72	1000	1695	118
225 – 255	0	58	798	133	12	0	1000	23	11	856	73	7	30	1000	1314	83
255 – 300	13	84	483	361	44	15	1000	27	165	569	186	15	38	1000	3731	271
300 – 340	6	70	562	312	27	24	1000	36	95	633	202	10	24	1000	7743	576
340 – 380	8	50	576	293	66	6	1000	39	98	690	94	7	71	1000	3552	296
380 – 420	36	65	525	268	88	18	1000	12	86	754	113	1	34	1000	5877	493
420 – 470	7	75	504	315	74	25	1000	25	142	660	139	0	33	1000	8343	707
470 – 525	38	88	626	226	10	11	1000	32	148	711	69	6	34	1000	8126	727
525 – 615	24	96	531	312	37	1	1000	57	113	719	66	12	33	1000	10204	894
615 – 775	31	68	712	144	33	11	1000	45	130	715	69	3	39	1000	13511	1031
775 – 950	28	136	619	199	16	2	1000	32	115	786	32	7	28	1000	4443	350
950 +	37	99	599	209	48	7	1000	60	147	722	39	7	25	1000	10211	676
all classes	25	82	599	240	42	11	1000	37	122	699	100	7	35	1000	78751	6222
estd. no. of aged persons(00)	719	2313	16983	6809	1201	316	28341	1879	6169	35215	5020	366	1760	50410	X	X
sample aged persons	55	227	1115	562	103	35	2097	157	524	2809	363	43	229	4125	X	X

For peer review only - http://bmjopen.bmj.com/site/about/guidelines.xhtml

Appendix A

Table (18.1): Per 1000 distribution of aged persons with illness or otherwise by their perception about change in state of health for each quintile class and sex

all-India Urban Females

mpce class	aged person with illness							aged person without illness							no. of aged persons	
	compared to previous year							compared to previous year							estd. (00)	sample
	much better	some-what better	nearly the same	some-what worse	worse	n.r.	total	much better	some what better	nearly the same	some what worse	worse	n.r.	total		
(1)	(2)	(3)	(4)	(5)	(6)	(7)	(8)	(9)	(10)	(11)	(12)	(13)	(14)	(15)	(16)	(17)
0 – 225	0	60	591	280	68	0	1000	60	74	556	189	17	104	1000	2745	170
225 – 255	0	40	505	415	40	0	1000	52	18	646	210	10	64	1000	1461	111
255 – 300	7	30	463	406	70	24	1000	36	146	601	149	27	41	1000	4458	315
300 – 340	2	51	554	306	62	26	1000	18	100	736	122	4	19	1000	8766	643
340 – 380	11	35	591	301	55	6	1000	42	107	691	64	11	85	1000	4032	304
380 – 420	30	90	505	260	89	27	1000	13	72	777	78	13	47	1000	7321	565
420 – 470	31	50	614	239	43	23	1000	31	138	699	89	6	37	1000	8550	710
470 – 525	32	89	567	281	27	4	1000	27	109	704	123	17	21	1000	8554	733
525 – 615	6	140	492	296	59	6	1000	41	129	693	109	5	24	1000	10703	892
615 – 775	16	81	679	194	18	12	1000	37	134	713	76	16	24	1000	12163	972
775 – 950	0	95	563	316	20	5	1000	27	129	724	62	16	42	1000	4024	310
950 +	40	78	590	224	42	25	1000	47	112	726	81	11	22	1000	9582	619
all classes	19	81	572	266	46	15	1000	33	114	702	104	12	35	1000	82359	6344
estd. no. of aged persons(00)	618	2600	18411	8569	1470	491	32159	1668	5715	35239	5196	602	1778	50200	X	X
sample aged persons	48	210	1268	645	115	42	2328	127	482	2704	428	40	235	4016	X	X

For peer review only

For peer review only - http://bmjopen.bmj.com/site/about/guidelines.xhtml

Appendix A

Table (18.1): Per 1000 distribution of aged persons with illness or otherwise by their perception about change in state of health for each quintile class and sex

all-India Urban Persons

mpce class	aged person with illness							aged person without illness							no. of aged persons	
	compared to previous year							compared to previous year							estd. (00)	sample
	much better	some-what better	nearly the same	some-what worse	worse	n.r.	total	much better	some what better	nearly the same	some what worse	worse	n.r.	total		
(1)	(2)	(3)	(4)	(5)	(6)	(7)	(8)	(9)	(10)	(11)	(12)	(13)	(14)	(15)	(16)	(17)
0 – 225	8	44	579	288	81	0	1000	41	86	560	196	24	93	1000	4441	288
225 – 255	0	48	634	290	28	0	1000	38	14	749	143	9	47	1000	2775	194
255 – 300	10	57	473	384	57	20	1000	32	155	587	165	21	40	1000	8189	586
300 – 340	4	60	557	308	46	25	1000	26	98	687	160	7	21	1000	16508	1219
340 – 380	10	42	584	298	60	6	1000	40	103	691	79	9	79	1000	7583	600
380 – 420	33	80	512	263	88	23	1000	13	79	766	95	7	41	1000	13198	1058
420 – 470	20	61	565	273	57	24	1000	28	140	679	115	3	35	1000	16894	1417
470 – 525	35	89	594	256	20	7	1000	30	128	708	95	11	28	1000	16680	1460
525 – 615	14	121	508	302	50	4	1000	49	120	707	86	9	29	1000	20907	1786
615 – 775	25	74	697	167	26	11	1000	41	132	714	72	10	31	1000	25674	2003
775 – 950	12	114	588	264	18	4	1000	30	121	760	45	11	34	1000	8468	660
950 +	39	89	595	217	45	16	1000	54	131	724	58	9	23	1000	19793	1295
all classes	22	81	585	254	44	13	1000	35	118	700	102	10	35	1000	161110	12566
estd. no. of aged persons(00)	1337	4912	35394	15379	2671	808	60500	3547	11884	70454	10217	968	3539	100610	X	X
sample aged persons	103	437	2383	1207	218	77	4425	284	1006	5513	791	83	464	8141	X	X

For peer review only - http://bmjopen.bmj.com/site/about/guidelines.xhtml

Appendix A

Table (18.1): Per 1000 distribution of aged persons with illness or otherwise by their perception about change in state of health for each quintile class and sex

all-India								Rural + Urban								Males
mpce class	aged person with illness							aged person without illness							no. of aged persons	
	compared to previous year							compared to previous year							estd. (00)	sample
	much better	some-what better	nearly the same	some-what worse	worse	n.r.	total	much better	some what better	nearly the same	some what worse	worse	n.r.	total		
(1)	(2)	(3)	(4)	(5)	(6)	(7)	(8)	(9)	(10)	(11)	(12)	(13)	(14)	(15)	(16)	(17)
0 – 225	10	76	498	312	73	31	1000	37	117	627	119	14	86	1000	11232	517
225 – 255	29	103	526	255	22	64	1000	30	57	716	119	24	54	1000	9725	385
255 – 300	27	82	457	325	76	33	1000	37	110	675	105	16	56	1000	24598	1076
300 – 340	3	44	554	332	56	10	1000	27	99	664	143	14	52	1000	25038	1320
340 – 380	7	66	560	329	31	7	1000	27	85	690	118	12	67	1000	25583	1260
380 – 420	52	78	480	313	63	14	1000	40	119	677	106	13	45	1000	29927	1539
420 – 470	32	87	493	288	68	32	1000	27	106	713	115	5	34	1000	34531	1891
470 – 525	17	76	500	338	44	27	1000	31	112	674	115	5	63	1000	35568	2008
525 – 615	18	93	512	312	52	12	1000	60	101	692	98	4	44	1000	41912	2386
615 – 775	23	82	589	257	36	12	1000	35	112	699	99	5	50	1000	46223	2607
775 – 950	30	128	529	273	29	11	1000	43	140	727	70	5	14	1000	19080	1160
950 +	32	105	530	292	39	3	1000	46	152	704	60	7	31	1000	28588	1601
all classes	24	86	525	300	48	17	1000	37	110	689	106	9	49	1000	332005	17750
estd. no. of aged persons(00)	2488	8795	53801	30684	4917	1724	102409	8564	25220	158205	24300	2098	11207	229596	X	X
sample aged persons	125	563	2691	1671	340	108	5498	470	1459	8137	1327	137	722	12252	X	X

For peer review only - http://bmjopen.bmj.com/site/about/guidelines.xhtml

Appendix A

Table (18.1): Per 1000 distribution of aged persons with illness or otherwise by their perception about change in state of health for each quintile class and sex

all-India Rural + Urban Females

mpce class	aged person with illness							aged person without illness							no. of aged persons	
	compared to previous year							compared to previous year							estd. (00)	sample
	much better	some-what better	nearly the same	some-what worse	worse	n.r.	total	much better	some what better	nearly the same	some what worse	worse	n.r.	total		
(1)	(2)	(3)	(4)	(5)	(6)	(7)	(8)	(9)	(10)	(11)	(12)	(13)	(14)	(15)	(16)	(17)
0 – 225	12	50	549	335	50	3	1000	29	135	599	153	12	72	1000	13968	585
225 – 255	0	80	517	355	49	0	1000	27	61	620	172	31	89	1000	10289	431
255 – 300	27	53	430	366	98	26	1000	23	108	657	144	13	56	1000	25546	1112
300 – 340	9	36	541	295	91	28	1000	17	110	701	120	4	48	1000	26626	1359
340 – 380	22	79	492	310	63	34	1000	19	79	691	135	19	56	1000	26683	1195
380 – 420	22	67	522	278	83	28	1000	19	86	701	118	17	60	1000	29338	1495
420 – 470	26	56	567	255	50	47	1000	22	92	703	121	13	49	1000	32949	1775
470 – 525	23	60	531	303	68	16	1000	26	89	674	154	14	43	1000	36068	1947
525 – 615	5	98	505	315	58	19	1000	45	100	667	141	10	38	1000	39323	2200
615 – 775	23	79	567	281	30	20	1000	28	112	692	123	7	38	1000	42144	2379
775 – 950	17	74	524	342	28	14	1000	32	144	670	120	11	24	1000	20281	1086
950 +	17	104	550	280	35	14	1000	34	140	667	114	13	32	1000	28560	1517
all classes	18	74	530	300	55	22	1000	27	103	677	133	13	48	1000	331775	17081
estd. no. of aged persons(00)	1875	7659	54877	31084	5738	2327	103560	6127	23589	154420	30300	2868	10909	228214	X	X
sample aged persons	106	444	2767	1668	331	126	5442	335	1219	7655	1574	162	694	11639	X	X

For peer review only

For peer review only - http://bmjopen.bmj.com/site/about/guidelines.xhtml

Appendix A

Table (18.1): Per 1000 distribution of aged persons with illness or otherwise by their perception about change in state of health for each quintile class and sex

all-India | Rural + Urban | Persons

mpce class	aged person with illness							aged person without illness							no. of aged persons	
	compared to previous year							compared to previous year							estd. (00)	sample
	much better	some-what better	nearly the same	some-what worse	worse	n.r.	total	much better	some what better	nearly the same	some what worse	worse	n.r.	total		
(1)	(2)	(3)	(4)	(5)	(6)	(7)	(8)	(9)	(10)	(11)	(12)	(13)	(14)	(15)	(16)	(17)
0 – 225	11	64	522	323	62	18	1000	32	127	610	139	13	78	1000	25200	1102
225 – 255	14	91	521	307	36	31	1000	28	59	667	146	27	72	1000	20014	816
255 – 300	27	67	444	346	87	30	1000	30	109	666	125	14	56	1000	50144	2188
300 – 340	6	40	546	312	75	20	1000	22	105	683	132	9	50	1000	51664	2679
340 – 380	15	73	523	319	48	22	1000	23	82	691	127	16	61	1000	52266	2455
380 – 420	37	73	501	296	73	21	1000	30	103	688	112	15	52	1000	59265	3034
420 – 470	29	72	528	272	59	39	1000	25	99	708	118	9	41	1000	67479	3666
470 – 525	20	68	515	320	56	21	1000	29	101	674	134	9	53	1000	71636	3955
525 – 615	12	96	509	313	55	16	1000	53	100	680	118	7	41	1000	81235	4586
615 – 775	23	81	579	268	33	16	1000	31	112	695	111	6	44	1000	88367	4986
775 – 950	23	99	527	310	28	13	1000	38	142	698	95	8	19	1000	39361	2246
950 +	24	104	540	286	37	9	1000	40	146	686	87	10	31	1000	57148	3118
all classes	21	80	528	300	52	20	1000	32	107	683	119	11	48	1000	663779	34831
estd. no. of aged persons(00)	4363	16454	108677	61768	10655	4051	205970	14692	48810	312625	54601	4966	22117	457810	X	X
sample aged persons	231	1007	5458	3339	671	234	10940	805	2678	15792	2901	299	1416	23891	X	X

For peer review only

For peer review only - http://bmjopen.bmj.com/site/about/guidelines.xhtml

Appendix A

Table (18.2): Per 1000 distribution of aged persons with illness or otherwise by their perception about change in state of health for each sex

Rural Males

state/ut	aged person with illness							aged person without illness							no. of aged persons	
	compared to previous year							compared to previous year							estd. (00)	sample
	much better	some-what better	nearly the same	some-what worse	worse	n.r.	total	much better	some what better	nearly the same	some what worse	worse	n.r.	total		
(1)	(2)	(3)	(4)	(5)	(6)	(7)	(8)	(9)	(10)	(11)	(12)	(13)	(14)	(15)	(16)	(17)
Andhra Pradesh	9	79	511	348	32	20	1000	27	71	801	79	0	22	1000	18730	697
Arunachal Pradesh	130	384	57	174	38	217	1000	146	138	151	31	7	526	1000	238	140
Assam	7	98	496	284	37	78	1000	42	127	613	83	21	115	1000	6103	471
Bihar	55	92	401	403	36	13	1000	50	66	681	101	12	90	1000	18861	912
Chhattisgarh	0	230	516	172	82	0	1000	32	221	577	100	12	58	1000	4609	204
Delhi	-	-	-	-	-	-	-	0	175	786	40	0	0	1000	338	16
Goa	0	0	121	879	0	0	1000	139	54	593	214	0	0	1000	506	19
Gujarat	55	67	634	230	13	0	1000	19	189	700	83	0	9	1000	10458	351
Haryana	0	77	390	479	54	0	1000	15	89	741	143	10	2	1000	5608	252
Himachal Pradesh	20	83	399	486	12	0	1000	74	75	676	143	3	27	1000	2400	347
Jammu & Kashmir	0	81	408	362	148	0	1000	36	141	736	53	0	35	1000	2246	230
Jharkhand	17	85	579	227	40	51	1000	5	54	639	208	37	57	1000	6400	330
Karnataka	42	109	517	313	19	0	1000	99	128	722	39	2	10	1000	11642	428
Kerala	9	70	456	363	82	20	1000	14	160	716	111	0	0	1000	11673	505
Madhya Pradesh	3	79	575	308	35	0	1000	17	85	756	93	11	38	1000	14666	549
Maharashtra	19	141	515	270	29	26	1000	66	142	674	102	4	11	1000	23347	717
Manipur	97	103	66	441	103	189	1000	69	107	436	109	0	279	1000	395	211
Meghalaya	0	194	384	422	0	0	1000	18	130	784	5	12	51	1000	467	96
Mizoram	281	0	158	456	0	104	1000	0	201	211	11	17	560	1000	119	89
Nagaland	28	248	237	414	0	73	1000	0	34	855	25	0	86	1000	109	56
Orissa	36	72	462	382	28	20	1000	51	93	667	136	19	33	1000	13371	525
Punjab	0	70	583	306	41	0	1000	32	96	772	73	7	20	1000	6843	258
Rajasthan	4	99	580	244	73	0	1000	18	67	779	117	10	8	1000	12196	552
Sikkim	0	129	343	375	152	0	1000	92	165	704	29	0	11	1000	115	110
Tamil Nadu	55	90	662	188	4	1	1000	68	102	735	92	0	2	1000	16961	571
Tripura	0	161	674	50	70	45	1000	29	250	540	58	38	85	1000	927	171

Continued

For peer review only

For peer review only - http://bmjopen.bmj.com/site/about/guidelines.xhtml

Appendix A

Table (18.2): Per 1000 distribution of aged persons with illness or otherwise by their perception about change in state of health for each sex

Rural | Males

state/ut	aged person with illness							aged person without illness							no. of aged persons	
	compared to previous year							compared to previous year							estd. (00)	sample
	much better	some-what better	nearly the same	some-what worse	worse	n.r.	total	much better	some what better	nearly the same	some what worse	worse	n.r.	total		
(1)	(2)	(3)	(4)	(5)	(6)	(7)	(8)	(9)	(10)	(11)	(12)	(13)	(14)	(15)	(16)	(17)
Uttaranchal	0	39	320	455	185	0	1000	0	53	758	118	0	71	1000	2674	102
Uttar Pradesh	24	72	454	350	65	35	1000	20	102	643	107	14	114	1000	42788	1825
West Bengal	31	74	404	353	112	26	1000	32	137	511	179	15	127	1000	18130	680
A & N Islands	0	188	720	92	0	0	1000	11	292	677	19	0	0	1000	76	34
Chandigarh	0	96	660	151	93	0	1000	0	0	918	82	0	0	1000	7	17
Dadra & N. Haveli	0	0	1000	0	0	0	1000	0	0	858	142	0	0	1000	78	20
Daman & Diu	0	0	0	0	0	0	0	0	0	1000	0	0	0	1000	4	7
Lakshadweep	0	89	753	158	0	0	1000	0	0	1000	0	0	0	1000	7	16
Pondicherry	0	20	753	227	0	0	1000	283	0	559	158	0	0	1000	160	20
OENS	30	191	461	194	55	69	1000	46	171	536	49	18	181	1000	2371	873
GUTs	0	58	745	193	3	0	1000	101	71	717	112	0	0	1000	333	114
all-India	24	88	497	322	50	19	1000	37	106	686	108	10	53	1000	253254	11528
estd. no. of aged persons(00)	1770	6482	36817	23875	3717	1408	74068	6685	19051	122990	19280	1732	9447	179186	X	X
sample aged persons	70	336	1576	1109	237	73	3401	313	935	5328	964	94	493	8127	X	X

For peer review only - http://bmjopen.bmj.com/site/about/guidelines.xhtml

Appendix A

Table (18.2): Per 1000 distribution of aged persons with illness or otherwise by their perception about change in state of health for each sex

Rural Females

state/ut	aged person with illness							aged person without illness							no. of aged persons	
	compared to previous year							compared to previous year							estd. (00)	sample
	much better	some-what better	nearly the same	some-what worse	worse	n.r.	total	much better	some what better	nearly the same	some what worse	worse	n.r.	total		
(1)	(2)	(3)	(4)	(5)	(6)	(7)	(8)	(9)	(10)	(11)	(12)	(13)	(14)	(15)	(16)	(17)
Andhra Pradesh	0	20	586	336	32	26	1000	9	76	771	117	5	22	1000	20035	688
Arunachal Pradesh	64	277	119	139	30	371	1000	129	109	260	71	10	421	1000	204	118
Assam	17	42	499	266	94	82	1000	17	80	647	113	9	135	1000	4273	323
Bihar	28	79	473	248	101	72	1000	15	76	609	147	28	125	1000	15258	678
Chhattisgarh	0	31	589	238	105	38	1000	24	245	558	116	17	40	1000	6680	290
Delhi	-	-	-	-	-	-	-	0	272	728	0	0	0	1000	322	17
Goa	0	0	113	865	22	0	1000	143	118	485	254	0	0	1000	615	32
Gujarat	0	134	594	252	19	0	1000	45	172	720	59	4	1	1000	10401	370
Haryana	94	24	257	562	63	0	1000	21	80	681	206	12	0	1000	5510	232
Himachal Pradesh	17	70	505	320	76	13	1000	24	69	717	145	6	39	1000	2546	346
Jammu & Kashmir	0	18	475	307	198	3	1000	30	116	684	143	20	8	1000	1555	173
Jharkhand	0	44	705	167	62	22	1000	7	45	607	227	74	39	1000	4441	231
Karnataka	40	133	444	336	46	0	1000	49	89	735	113	2	12	1000	10878	403
Kerala	5	61	498	373	55	8	1000	4	110	702	108	21	55	1000	15013	649
Madhya Pradesh	0	63	624	262	24	26	1000	12	62	740	136	9	41	1000	14701	544
Maharashtra	20	93	599	199	55	34	1000	30	181	656	114	1	18	1000	24333	716
Manipur	0	39	163	629	168	0	1000	51	87	503	157	8	194	1000	279	163
Meghalaya	0	118	416	389	0	76	1000	38	115	712	71	6	60	1000	431	95
Mizoram	0	349	313	0	0	338	1000	128	115	235	37	0	485	1000	76	48
Nagaland	0	0	659	341	0	0	1000	0	0	623	265	0	112	1000	39	24
Orissa	22	73	415	399	92	0	1000	25	79	672	180	14	30	1000	12157	483
Punjab	7	93	453	387	54	5	1000	20	86	742	133	12	7	1000	6518	239
Rajasthan	8	53	402	442	96	0	1000	22	70	759	127	22	0	1000	12999	583
Sikkim	0	0	388	612	0	0	1000	74	210	559	57	0	100	1000	109	94
Tamil Nadu	42	103	674	171	10	0	1000	76	125	733	59	4	4	1000	17146	549
Tripura	0	222	596	54	117	12	1000	16	203	648	77	26	30	1000	675	135

Continued

For peer review only - http://bmjopen.bmj.com/site/about/guidelines.xhtml

Appendix A

Table (18.2): Per 1000 distribution of aged persons with illness or otherwise by their perception about change in state of health for each sex

Rural | Females

state/ut	aged person with illness							aged person without illness							no. of aged persons	
	compared to previous year							compared to previous year							estd. (00)	sample
	much better	some-what better	nearly the same	some-what worse	worse	n.r.	total	much better	some what better	nearly the same	some what worse	worse	n.r.	total		
(1)	(2)	(3)	(4)	(5)	(6)	(7)	(8)	(9)	(10)	(11)	(12)	(13)	(14)	(15)	(16)	(17)
Uttaranchal	0	301	370	329	0	0	1000	0	67	724	190	7	11	1000	2190	90
Uttar Pradesh	17	78	483	318	63	41	1000	14	75	630	166	10	106	1000	41298	1649
West Bengal	35	42	374	388	113	49	1000	31	81	472	266	23	127	1000	18472	686
A & N Islands	0	0	386	614	0	0	1000	0	155	650	0	0	195	1000	43	15
Chandigarh	0	0	605	282	113	0	1000	0	0	787	213	0	0	1000	8	11
Dadra & N. Haveli	0	0	0	1000	0	0	1000	0	90	865	45	0	0	1000	39	10
Daman & Diu	0	545	455	0	0	0	1000	0	22	978	0	0	0	1000	13	13
Lakshadweep	0	0	807	193	0	0	1000	186	0	814	0	0	0	1000	9	22
Pondicherry	0	0	852	148	0	0	1000	0	0	802	198	0	0	1000	148	18
OENS	7	184	467	197	78	68	1000	49	140	567	91	12	140	1000	1813	677
GUTs	0	9	784	205	2	0	1000	5	67	794	83	0	51	1000	261	89
all-India	18	71	511	315	60	26	1000	25	100	669	141	13	51	1000	249416	10737
estd. no. of aged persons(00)	1257	5060	36465	22515	4268	1836	71401	4459	17874	119180	25104	2266	9131	178015	X	X
sample aged persons	58	234	1499	1023	216	84	3114	208	737	4951	1146	122	459	7623	X	X

For peer review only - http://bmjopen.bmj.com/site/about/guidelines.xhtml

Appendix A

Table (18.2): Per 1000 distribution of aged persons with illness or otherwise by their perception about change in state of health for each sex

Rural Persons

state/ut	aged person with illness							aged person without illness							no. of aged persons	
	compared to previous year							compared to previous year							estd. (00)	sample
	much better	some-what better	nearly the same	some-what worse	worse	n.r.	total	much better	some what better	nearly the same	some what worse	worse	n.r.	total		
(1)	(2)	(3)	(4)	(5)	(6)	(7)	(8)	(9)	(10)	(11)	(12)	(13)	(14)	(15)	(16)	(17)
Andhra Pradesh	4	48	551	341	32	23	1000	18	74	785	98	3	22	1000	38765	1385
Arunachal Pradesh	102	339	83	159	35	282	1000	138	124	203	50	8	476	1000	442	258
Assam	11	74	498	276	62	79	1000	32	108	626	94	16	123	1000	10376	794
Bihar	44	87	430	340	62	37	1000	34	70	648	122	19	106	1000	34119	1590
Chhattisgarh	0	118	557	209	95	21	1000	27	235	566	109	15	48	1000	11289	494
Delhi	-	-	-	-	-	-	-	0	222	758	20	0	0	1000	661	33
Goa	0	0	117	871	12	0	1000	141	90	532	237	0	0	1000	1121	51
Gujarat	33	94	618	239	15	0	1000	33	180	711	70	2	5	1000	20859	721
Haryana	43	53	329	517	58	0	1000	18	84	711	175	11	1	1000	11118	484
Himachal Pradesh	18	77	451	405	43	6	1000	48	72	698	144	5	33	1000	4946	693
Jammu & Kashmir	0	54	436	339	169	1	1000	33	131	715	89	8	24	1000	3801	403
Jharkhand	12	72	621	207	48	41	1000	6	50	626	216	53	49	1000	10841	561
Karnataka	41	119	486	323	31	0	1000	74	108	729	77	2	11	1000	22520	831
Kerala	7	65	480	369	66	13	1000	8	132	708	109	11	31	1000	26687	1154
Madhya Pradesh	2	72	598	287	30	12	1000	14	74	748	115	10	39	1000	29367	1093
Maharashtra	19	117	557	235	42	30	1000	48	162	665	108	2	15	1000	47679	1433
Manipur	52	74	112	529	133	101	1000	62	99	463	129	3	244	1000	673	374
Meghalaya	0	156	400	405	0	38	1000	27	123	749	37	9	55	1000	898	191
Mizoram	161	149	224	262	0	204	1000	49	168	220	21	10	532	1000	196	137
Nagaland	23	205	310	402	0	60	1000	0	24	788	95	0	94	1000	148	80
Orissa	29	72	440	390	58	11	1000	38	87	670	157	17	32	1000	25527	1008
Punjab	4	83	510	352	48	3	1000	27	92	759	100	9	14	1000	13361	497
Rajasthan	6	78	498	335	83	0	1000	20	69	769	122	16	4	1000	25196	1135
Sikkim	0	65	365	492	77	0	1000	83	187	634	42	0	54	1000	224	204
Tamil Nadu	48	96	668	180	7	1	1000	72	114	734	76	2	3	1000	34107	1120
Tripura	0	189	637	52	92	29	1000	24	231	583	66	33	63	1000	1602	306

Continued

For peer review only - http://bmjopen.bmj.com/site/about/guidelines.xhtml

For peer review only

Appendix A

Table (18.2): Per 1000 distribution of aged persons with illness or otherwise by their perception about change in state of health for each sex

Rural | Persons

state/ut	aged person with illness							aged person without illness							no. of aged persons	
	compared to previous year							compared to previous year							estd. (00)	sample
	much better	some-what better	nearly the same	some-what worse	worse	n.r.	total	much better	some what better	nearly the same	some what worse	worse	n.r.	total		
(1)	(2)	(3)	(4)	(5)	(6)	(7)	(8)	(9)	(10)	(11)	(12)	(13)	(14)	(15)	(16)	(17)
Uttaranchal	0	144	340	405	111	0	1000	0	59	743	151	3	43	1000	4865	192
Uttar Pradesh	21	75	468	335	64	38	1000	17	89	636	137	12	110	1000	84086	3474
West Bengal	33	57	388	371	112	38	1000	32	109	491	222	19	127	1000	36602	1366
A & N Islands	0	138	631	231	0	0	1000	7	237	667	12	0	77	1000	120	49
Chandigarh	0	55	637	206	102	0	1000	0	0	838	162	0	0	1000	16	28
Dadra & N. Haveli	0	0	221	779	0	0	1000	0	28	860	112	0	0	1000	117	30
Daman & Diu	0	545	455	0	0	0	1000	0	16	984	0	0	0	1000	17	20
Lakshadweep	0	30	789	181	0	0	1000	83	0	917	0	0	0	1000	16	38
Pondicherry	0	9	808	183	0	0	1000	183	0	645	172	0	0	1000	309	38
OENS	19	188	464	195	65	68	1000	47	158	550	67	15	163	1000	4184	1550
GUTs	0	32	766	200	3	0	1000	65	69	746	101	0	19	1000	594	203
all-India	21	79	504	319	55	22	1000	31	103	678	124	11	52	1000	502670	22265
estd. no. of aged persons(00)	3027	11542	73283	46390	7984	3244	145469	11144	36925	242171	44384	3998	18578	357200	X	X
sample aged persons	128	570	3075	2132	453	157	6515	521	1672	10279	2110	216	952	15750	X	X

For peer review only - http://bmjopen.bmj.com/site/about/guidelines.xhtml

Appendix A

Table (18.2): Per 1000 distribution of aged persons with illness or otherwise by their perception about change in state of health for each sex

Urban Males

state/ut	aged person with illness							aged person without illness							no. of aged persons	
	compared to previous year							compared to previous year							estd. (00)	sample
	much better	some-what better	nearly the same	some-what worse	worse	n.r.	total	much better	some what better	nearly the same	some what worse	worse	n.r.	total		
(1)	(2)	(3)	(4)	(5)	(6)	(7)	(8)	(9)	(10)	(11)	(12)	(13)	(14)	(15)	(16)	(17)
Andhra Pradesh	6	62	671	213	35	13	1000	36	66	771	82	0	44	1000	5310	377
Arunachal Pradesh	0	158	526	0	0	316	1000	17	108	264	34	0	577	1000	10	42
Assam	0	108	535	147	128	83	1000	46	146	623	71	11	104	1000	647	109
Bihar	14	18	641	220	106	0	1000	19	92	548	227	15	99	1000	2455	169
Chhattisgarh	44	71	371	514	0	0	1000	104	82	766	32	16	0	1000	641	63
Delhi	14	53	809	110	14	0	1000	2	214	711	57	0	16	1000	2212	211
Goa	156	0	183	661	0	0	1000	55	150	710	85	0	0	1000	229	32
Gujarat	0	106	681	196	16	0	1000	34	92	801	71	1	0	1000	4592	297
Haryana	7	83	520	329	61	0	1000	48	51	740	151	0	10	1000	1315	132
Himachal Pradesh	0	0	529	471	0	0	1000	0	38	840	122	0	0	1000	139	49
Jammu & Kashmir	22	38	497	410	33	0	1000	11	105	768	105	0	11	1000	408	102
Jharkhand	0	226	390	365	20	0	1000	20	178	651	131	8	12	1000	1372	166
Karnataka	71	95	533	253	40	9	1000	56	175	658	91	0	19	1000	4025	345
Kerala	27	41	636	213	66	16	1000	59	99	747	76	16	3	1000	3986	290
Madhya Pradesh	15	77	559	336	13	0	1000	26	132	654	127	14	47	1000	4115	319
Maharashtra	21	99	615	222	36	6	1000	36	141	686	97	10	30	1000	12844	596
Manipur	0	145	226	291	338	0	1000	23	182	472	45	18	259	1000	192	116
Meghalaya	0	448	340	0	0	212	1000	19	217	436	309	10	8	1000	42	34
Mizoram	0	75	208	416	0	301	1000	11	59	326	38	0	565	1000	85	142
Nagaland	627	0	318	55	0	0	1000	0	0	824	6	0	169	1000	68	24
Orissa	65	26	508	382	20	0	1000	2	117	589	236	22	34	1000	1546	148
Punjab	135	112	419	260	26	48	1000	10	67	900	7	1	15	1000	2565	165
Rajasthan	38	22	538	377	24	0	1000	31	45	811	110	2	2	1000	3012	271
Sikkim	0	0	556	444	0	0	1000	207	213	580	0	0	0	1000	11	16
Tamil Nadu	40	117	682	152	9	0	1000	98	166	697	37	2	0	1000	7955	587
Tripura	64	369	474	68	25	0	1000	40	222	581	109	0	49	1000	130	45

Continued

For peer review only - http://bmjopen.bmj.com/site/about/guidelines.xhtml

Appendix A

Table (18.2): Per 1000 distribution of aged persons with illness or otherwise by their perception about change in state of health for each sex

Urban — Males

state/ut	aged person with illness							aged person without illness							no. of aged persons	
	compared to previous year							compared to previous year							estd. (00)	sample
	much better	some-what better	nearly the same	some-what worse	worse	n.r.	total	much better	some what better	nearly the same	some what worse	worse	n.r.	total		
(1)	(2)	(3)	(4)	(5)	(6)	(7)	(8)	(9)	(10)	(11)	(12)	(13)	(14)	(15)	(16)	(17)
Uttaranchal	0	57	554	254	135	0	1000	43	88	846	6	17	0	1000	558	49
Uttar Pradesh	33	64	498	341	40	24	1000	18	69	681	153	12	67	1000	9104	620
West Bengal	12	79	601	212	78	17	1000	27	172	626	92	6	76	1000	8725	522
A & N Islands	0	535	208	257	0	0	1000	0	53	881	21	0	45	1000	18	18
Chandigarh	0	211	724	65	0	0	1000	0	43	911	46	0	0	1000	197	69
Dadra & N. Haveli	0	0	1000	0	0	0	1000	0	0	981	19	0	0	1000	4	13
Daman & Diu	0	0	911	89	0	0	1000	0	0	916	84	0	0	1000	10	16
Lakshadweep	0	512	177	71	240	0	1000	0	221	649	130	0	0	1000	8	21
Pondicherry	0	101	691	208	0	0	1000	0	274	726	0	0	0	1000	218	47
OENS	173	223	374	134	47	49	1000	26	152	504	74	8	236	1000	538	419
GUTs	0	146	671	177	5	0	1000	0	118	848	32	0	2	1000	455	184
all-India	25	82	599	240	42	11	1000	37	122	699	100	7	35	1000	78751	6222
estd. no. of aged persons(00)	719	2313	16983	6809	1201	316	28341	1879	6169	35215	5020	366	1760	50410	X	X
sample aged persons	55	227	1115	562	103	35	2097	157	524	2809	363	43	229	4125	X	X

For peer review only - http://bmjopen.bmj.com/site/about/guidelines.xhtml

Appendix A

Table (18.2): Per 1000 distribution of aged persons with illness or otherwise by their perception about change in state of health for each sex

Urban | Females

state/ut	aged person with illness							aged person without illness							no. of aged persons	
	compared to previous year							compared to previous year							estd. (00)	sample
	much better	some-what better	nearly the same	some-what worse	worse	n.r.	total	much better	some what better	nearly the same	some what worse	worse	n.r.	total		
(1)	(2)	(3)	(4)	(5)	(6)	(7)	(8)	(9)	(10)	(11)	(12)	(13)	(14)	(15)	(16)	(17)
Andhra Pradesh	6	42	690	211	35	16	1000	8	93	736	119	15	29	1000	6160	421
Arunachal Pradesh	129	473	215	54	0	129	1000	69	11	342	0	0	578	1000	7	30
Assam	0	59	421	366	131	24	1000	23	80	628	134	0	134	1000	532	85
Bihar	0	89	507	339	51	14	1000	8	59	753	40	50	89	1000	1829	145
Chhattisgarh	49	143	394	197	217	0	1000	110	209	497	167	18	0	1000	760	88
Delhi	0	27	813	150	0	10	1000	16	149	736	85	0	13	1000	2129	207
Goa	0	0	672	319	8	0	1000	41	27	796	136	0	0	1000	256	40
Gujarat	12	48	655	267	18	0	1000	20	141	722	95	9	12	1000	5101	319
Haryana	0	46	395	438	120	0	1000	24	45	690	185	13	43	1000	1725	143
Himachal Pradesh	0	224	564	180	0	31	1000	0	62	809	129	0	0	1000	155	50
Jammu & Kashmir	0	60	687	235	0	17	1000	9	107	778	97	0	10	1000	336	91
Jharkhand	0	53	673	191	37	46	1000	28	51	729	150	14	28	1000	1292	139
Karnataka	57	88	599	210	45	0	1000	90	137	659	88	19	7	1000	4073	353
Kerala	10	46	600	296	49	0	1000	6	90	773	76	0	55	1000	4686	322
Madhya Pradesh	17	104	418	344	97	21	1000	20	174	606	132	13	54	1000	4466	327
Maharashtra	25	112	601	211	37	15	1000	40	115	727	90	10	18	1000	13669	672
Manipur	0	10	326	425	104	136	1000	27	99	454	75	4	341	1000	178	115
Meghalaya	0	191	286	523	0	0	1000	27	76	818	38	0	41	1000	48	46
Mizoram	0	0	92	479	0	429	1000	7	79	382	71	0	462	1000	60	104
Nagaland	0	0	0	0	0	0	0	368	0	632	0	0	0	1000	8	5
Orissa	0	46	743	156	55	0	1000	16	74	679	121	6	105	1000	1182	113
Punjab	49	202	472	228	49	0	1000	62	64	789	69	0	16	1000	2347	164
Rajasthan	8	51	528	302	109	0	1000	39	48	770	137	7	0	1000	3076	266
Sikkim	0	0	481	519	0	0	1000	461	276	263	0	0	0	1000	7	13
Tamil Nadu	15	87	670	211	17	0	1000	72	164	713	50	0	1	1000	8984	650
Tripura	64	217	562	0	157	0	1000	11	328	449	89	24	100	1000	136	54

Continued

For peer review only - http://bmjopen.bmj.com/site/about/guidelines.xhtml

Appendix A

Table (18.2): Per 1000 distribution of aged persons with illness or otherwise by their perception about change in state of health for each sex

Urban Females

state/ut	aged person with illness							aged person without illness							no. of aged persons	
	compared to previous year							compared to previous year							estd. (00)	sample
	much better	some-what better	nearly the same	some-what worse	worse	n.r.	total	much better	some what better	nearly the same	some what worse	worse	n.r.	total		
(1)	(2)	(3)	(4)	(5)	(6)	(7)	(8)	(9)	(10)	(11)	(12)	(13)	(14)	(15)	(16)	(17)
Uttaranchal	0	0	664	220	116	0	1000	0	42	838	71	11	38	1000	693	49
Uttar Pradesh	22	55	450	381	45	46	1000	15	86	642	167	11	79	1000	9590	621
West Bengal	25	102	483	311	52	27	1000	14	128	675	85	40	59	1000	8315	506
A & N Islands	0	375	346	279	0	0	1000	0	309	504	120	0	67	1000	15	22
Chandigarh	0	0	671	315	14	0	1000	0	0	942	11	31	16	1000	147	68
Dadra & N. Haveli	0	0	1000	0	0	0	1000	0	0	916	84	0	0	1000	3	14
Daman & Diu	0	0	1000	0	0	0	1000	13	35	863	44	0	44	1000	16	22
Lakshadweep	0	479	253	268	0	0	1000	0	81	919	0	0	0	1000	10	24
Pondicherry	0	117	668	129	86	0	1000	0	130	811	59	0	0	1000	368	56
OENS	28	131	383	278	88	92	1000	33	161	477	72	8	250	1000	444	367
GUTs	0	105	659	164	72	0	1000	1	82	851	42	12	12	1000	560	206
all-India	19	81	572	266	46	15	1000	33	114	702	104	12	35	1000	82359	6344
estd. no. of aged persons(00)	618	2600	18411	8569	1470	491	32159	1668	5715	35239	5196	602	1778	50200	X	X
sample aged persons	48	210	1268	645	115	42	2328	127	482	2704	428	40	235	4016	X	X

For peer review only - http://bmjopen.bmj.com/site/about/guidelines.xhtml

Appendix A

Table (18.2): Per 1000 distribution of aged persons with illness or otherwise by their perception about change in state of health for each sex

Urban Persons

state/ut	aged person with illness							aged person without illness							no. of aged persons	
	compared to previous year							compared to previous year							estd. (00)	sample
	much better	some-what better	nearly the same	some-what worse	worse	n.r.	total	much better	some what better	nearly the same	some what worse	worse	n.r.	total		
(1)	(2)	(3)	(4)	(5)	(6)	(7)	(8)	(9)	(10)	(11)	(12)	(13)	(14)	(15)	(16)	(17)
Andhra Pradesh	6	51	681	212	35	15	1000	22	80	753	101	8	36	1000	11470	798
Arunachal Pradesh	92	382	305	38	0	183	1000	36	72	293	21	0	577	1000	17	72
Assam	0	88	489	234	129	60	1000	35	115	625	101	6	118	1000	1180	194
Bihar	8	49	583	271	82	6	1000	15	78	635	148	30	95	1000	4283	314
Chhattisgarh	46	106	382	361	105	0	1000	107	154	615	108	17	0	1000	1401	151
Delhi	7	40	811	131	7	5	1000	9	183	723	71	0	15	1000	4342	418
Goa	40	0	546	407	6	0	1000	49	96	747	107	0	0	1000	485	72
Gujarat	5	79	669	229	17	0	1000	26	120	757	85	6	7	1000	9693	616
Haryana	3	64	457	385	91	0	1000	34	47	711	171	8	30	1000	3040	275
Himachal Pradesh	0	117	548	319	0	16	1000	0	50	824	126	0	0	1000	294	99
Jammu & Kashmir	13	47	574	340	20	7	1000	10	106	773	101	0	11	1000	744	193
Jharkhand	0	167	487	305	26	16	1000	24	113	691	141	11	20	1000	2664	305
Karnataka	63	91	570	229	43	4	1000	72	157	659	90	9	13	1000	8098	698
Kerala	17	44	616	260	56	7	1000	32	95	760	76	8	29	1000	8672	612
Madhya Pradesh	16	91	485	340	57	11	1000	23	154	629	130	14	50	1000	8580	646
Maharashtra	23	106	608	216	37	11	1000	38	128	706	94	10	24	1000	26513	1268
Manipur	0	55	292	380	182	90	1000	25	143	463	59	11	298	1000	369	231
Meghalaya	0	238	296	427	0	39	1000	22	149	620	178	5	24	1000	90	80
Mizoram	0	44	159	443	0	355	1000	9	67	350	52	0	522	1000	145	246
Nagaland	627	0	318	55	0	0	1000	48	0	799	5	0	147	1000	76	29
Orissa	31	36	629	266	38	0	1000	8	99	626	188	15	64	1000	2729	261
Punjab	91	158	446	244	38	24	1000	34	66	849	36	1	15	1000	4912	329
Rajasthan	24	36	534	342	65	0	1000	35	46	790	124	4	1	1000	6088	537
Sikkim	0	0	492	508	0	0	1000	292	235	473	0	0	0	1000	18	29
Tamil Nadu	26	100	675	185	13	0	1000	85	165	705	44	1	0	1000	16939	1237
Tripura	64	312	507	42	75	0	1000	24	280	508	98	13	77	1000	267	99

Continued

For peer review only - http://bmjopen.bmj.com/site/about/guidelines.xhtml

Appendix A

Table (18.2): Per 1000 distribution of aged persons with illness or otherwise by their perception about change in state of health for each sex

Urban | Persons

state/ut	aged person with illness							aged person without illness							no. of aged persons	
	compared to previous year							compared to previous year							estd. (00)	sample
	much better	some-what better	nearly the same	some-what worse	worse	n.r.	total	much better	some what better	nearly the same	some what worse	worse	n.r.	total		
(1)	(2)	(3)	(4)	(5)	(6)	(7)	(8)	(9)	(10)	(11)	(12)	(13)	(14)	(15)	(16)	(17)
Uttaranchal	0	36	594	242	128	0	1000	17	60	841	46	13	23	1000	1252	98
Uttar Pradesh	27	59	471	364	43	37	1000	17	77	662	160	11	73	1000	18694	1241
West Bengal	18	91	540	263	65	22	1000	21	152	648	89	22	68	1000	17040	1028
A & N Islands	0	494	243	263	0	0	1000	0	187	683	73	0	57	1000	33	40
Chandigarh	0	84	692	215	9	0	1000	0	27	922	33	11	6	1000	344	137
Dadra & N. Haveli	0	0	1000	0	0	0	1000	0	0	951	49	0	0	1000	7	27
Daman & Diu	0	0	939	61	0	0	1000	9	23	881	58	0	29	1000	26	38
Lakshadweep	0	492	222	189	96	0	1000	0	150	787	64	0	0	1000	19	45
Pondicherry	0	111	676	156	57	0	1000	0	192	774	34	0	0	1000	586	103
OENS	109	182	378	197	65	68	1000	29	156	492	73	8	242	1000	983	786
GUTs	0	120	664	169	48	0	1000	0	101	850	37	6	7	1000	1015	390
all-India	22	81	585	254	44	13	1000	35	118	700	102	10	35	1000	161110	12566
estd. no. of aged persons(00)	1337	4912	35394	15379	2671	808	60500	3547	11884	70454	10217	968	3539	100610	X	X
sample aged persons	103	437	2383	1207	218	77	4425	284	1006	5513	791	83	464	8141	X	X

For peer review only

For peer review only - http://bmjopen.bmj.com/site/about/guidelines.xhtml

Appendix A

Table (18.2): Per 1000 distribution of aged persons with illness or otherwise by their perception about change in state of health for each sex

Rural + Urban Males

state/ut	aged person with illness							aged person without illness							no. of aged persons	
	compared to previous year							compared to previous year							estd. (00)	sample
	much better	some-what better	nearly the same	some-what worse	worse	n.r.	total	much better	some what better	nearly the same	some what worse	worse	n.r.	total		
(1)	(2)	(3)	(4)	(5)	(6)	(7)	(8)	(9)	(10)	(11)	(12)	(13)	(14)	(15)	(16)	(17)
Andhra Pradesh	8	74	559	307	33	18	1000	28	70	796	80	0	26	1000	24041	1074
Arunachal Pradesh	129	381	62	172	37	218	1000	139	137	157	31	6	529	1000	248	182
Assam	6	99	500	270	46	78	1000	42	129	614	82	20	114	1000	6750	580
Bihar	49	81	437	376	46	11	1000	47	69	667	115	12	91	1000	21316	1081
Chhattisgarh	9	198	487	240	66	0	1000	40	207	597	93	12	52	1000	5250	267
Delhi	14	53	809	110	14	0	1000	2	208	722	55	0	13	1000	2551	227
Goa	16	0	127	857	0	0	1000	96	104	654	147	0	0	1000	735	51
Gujarat	38	79	649	220	14	0	1000	23	160	731	79	0	6	1000	15050	648
Haryana	2	78	421	444	56	0	1000	21	82	741	144	8	3	1000	6923	384
Himachal Pradesh	18	78	407	485	11	0	1000	70	73	685	142	3	26	1000	2539	396
Jammu & Kashmir	4	74	423	371	128	0	1000	32	136	741	60	0	31	1000	2654	332
Jharkhand	12	129	520	270	34	35	1000	7	74	640	196	33	50	1000	7772	496
Karnataka	49	105	520	300	24	2	1000	87	141	705	53	1	13	1000	15667	773
Kerala	13	63	499	327	78	19	1000	26	143	724	101	5	1	1000	15659	795
Madhya Pradesh	6	78	571	315	30	0	1000	19	95	734	100	12	40	1000	18781	868
Maharashtra	19	124	557	250	32	18	1000	57	142	678	100	6	17	1000	36191	1313
Manipur	76	112	101	409	154	148	1000	54	132	448	88	6	272	1000	587	327
Meghalaya	0	202	383	410	0	6	1000	18	138	751	34	12	47	1000	510	130
Mizoram	144	37	182	437	0	200	1000	4	144	258	22	10	562	1000	204	231
Nagaland	261	152	269	275	0	44	1000	0	21	843	18	0	118	1000	178	80
Orissa	39	68	466	382	27	18	1000	46	96	659	147	20	33	1000	14917	673
Punjab	35	81	540	294	37	13	1000	26	88	808	55	5	18	1000	9408	423
Rajasthan	14	76	567	284	58	0	1000	20	63	785	116	9	7	1000	15208	823
Sikkim	0	128	346	376	150	0	1000	103	170	692	26	0	10	1000	126	126
Tamil Nadu	50	99	669	176	6	1	1000	77	122	723	75	1	1	1000	24916	1158
Tripura	8	188	648	52	65	39	1000	30	247	545	64	34	80	1000	1057	216

Continued

For peer review only - http://bmjopen.bmj.com/site/about/guidelines.xhtml

Appendix A

Table (18.2): Per 1000 distribution of aged persons with illness or otherwise by their perception about change in state of health for each sex

Rural + Urban | Males

state/ut	aged person with illness							aged person without illness							no. of aged persons	
	compared to previous year							compared to previous year							estd. (00)	sample
	much better	some-what better	nearly the same	some-what worse	worse	n.r.	total	much better	some what better	nearly the same	some what worse	worse	n.r.	total		
(1)	(2)	(3)	(4)	(5)	(6)	(7)	(8)	(9)	(10)	(11)	(12)	(13)	(14)	(15)	(16)	(17)
Uttaranchal	0	44	390	395	170	0	1000	6	58	771	102	2	61	1000	3232	151
Uttar Pradesh	26	71	462	348	60	33	1000	20	96	650	115	13	106	1000	51892	2445
West Bengal	24	76	481	298	99	23	1000	31	147	543	155	12	113	1000	26855	1202
A & N Islands	0	260	614	126	0	0	1000	9	248	714	20	0	8	1000	94	52
Chandigarh	0	200	718	73	9	0	1000	0	42	911	47	0	0	1000	205	86
Dadra & N. Haveli	0	0	1000	0	0	0	1000	0	0	863	137	0	0	1000	82	33
Daman & Diu	0	0	911	89	0	0	1000	0	0	945	55	0	0	1000	14	23
Lakshadweep	0	337	416	107	141	0	1000	0	110	826	64	0	0	1000	15	37
Pondicherry	0	69	715	215	0	0	1000	131	148	649	73	0	0	1000	379	67
OENS	50	195	449	186	54	66	1000	42	167	530	54	16	192	1000	2909	1292
GUTs	0	112	701	183	4	0	1000	44	97	791	67	0	1	1000	789	298
all-India	24	86	525	300	48	17	1000	37	110	689	106	9	49	1000	332005	17750
estd. no. of aged persons(00)	2488	8795	53801	30684	4917	1724	102409	8564	25220	158205	24300	2098	11207	229596	X	X
sample aged persons	125	563	2691	1671	340	108	5498	470	1459	8137	1327	137	722	12252	X	X

For peer review only - http://bmjopen.bmj.com/site/about/guidelines.xhtml

Appendix A

Table (18.2): Per 1000 distribution of aged persons with illness or otherwise by their perception about change in state of health for each sex

Rural + Urban Females

state/ut	aged person with illness							aged person without illness							no. of aged persons	
	compared to previous year							compared to previous year							estd. (00)	sample
	much better	some-what better	nearly the same	some-what worse	worse	n.r.	total	much better	some what better	nearly the same	some what worse	worse	n.r.	total		
(1)	(2)	(3)	(4)	(5)	(6)	(7)	(8)	(9)	(10)	(11)	(12)	(13)	(14)	(15)	(16)	(17)
Andhra Pradesh	2	27	619	296	33	23	1000	9	79	765	117	6	23	1000	26195	1109
Arunachal Pradesh	66	283	123	136	29	363	1000	127	106	263	69	9	426	1000	211	148
Assam	15	43	492	275	98	77	1000	17	80	645	115	8	135	1000	4806	408
Bihar	23	81	478	263	93	62	1000	15	74	623	137	30	121	1000	17087	823
Chhattisgarh	7	48	560	232	121	32	1000	32	242	552	121	17	37	1000	7440	378
Delhi	0	27	813	150	0	10	1000	14	168	735	72	0	11	1000	2452	224
Goa	0	0	235	746	19	0	1000	103	82	608	208	0	0	1000	871	72
Gujarat	4	102	617	258	19	0	1000	37	162	720	70	5	5	1000	15501	689
Haryana	69	30	295	528	79	0	1000	22	72	683	201	12	10	1000	7235	375
Himachal Pradesh	16	80	509	311	71	14	1000	23	69	722	144	6	37	1000	2701	396
Jammu & Kashmir	0	24	509	295	166	5	1000	26	114	701	134	16	9	1000	1891	264
Jharkhand	0	47	694	175	54	29	1000	12	47	634	210	61	36	1000	5733	370
Karnataka	46	118	498	293	46	0	1000	59	101	717	107	6	11	1000	14951	756
Kerala	6	57	523	354	53	6	1000	4	106	719	100	16	55	1000	19699	971
Madhya Pradesh	5	75	567	285	44	25	1000	14	87	710	135	10	43	1000	19166	871
Maharashtra	22	101	600	204	47	25	1000	33	161	678	106	4	18	1000	38001	1388
Manipur	0	28	226	550	143	52	1000	42	92	484	125	6	251	1000	456	278
Meghalaya	0	127	401	405	0	67	1000	37	111	722	68	5	58	1000	479	141
Mizoram	0	182	207	230	0	382	1000	75	99	299	52	0	475	1000	137	152
Nagaland	0	0	659	341	0	0	1000	69	0	625	215	0	91	1000	47	29
Orissa	19	70	452	372	88	0	1000	24	79	673	175	13	36	1000	13339	596
Punjab	17	118	457	351	53	4	1000	32	80	756	115	8	9	1000	8865	403
Rajasthan	8	52	441	398	100	0	1000	25	66	761	128	19	0	1000	16075	849
Sikkim	0	0	395	605	0	0	1000	96	214	542	54	0	94	1000	116	107
Tamil Nadu	32	97	672	186	13	0	1000	75	138	726	56	3	3	1000	26130	1199
Tripura	6	221	593	49	120	11	1000	15	228	608	80	25	44	1000	812	189

Continued

For peer review only - http://bmjopen.bmj.com/site/about/guidelines.xhtml

For peer review only

Appendix A

Table (18.2): Per 1000 distribution of aged persons with illness or otherwise by their perception about change in state of health for each sex

Rural + Urban | Females

state/ut	aged person with illness							aged person without illness							no. of aged persons	
	compared to previous year							compared to previous year							estd. (00)	sample
	much better	some-what better	nearly the same	some-what worse	worse	n.r.	total	much better	some what better	nearly the same	some what worse	worse	n.r.	total		
(1)	(2)	(3)	(4)	(5)	(6)	(7)	(8)	(9)	(10)	(11)	(12)	(13)	(14)	(15)	(16)	(17)
Uttaranchal	0	220	449	300	31	0	1000	0	61	751	162	8	18	1000	2884	139
Uttar Pradesh	18	73	475	333	59	43	1000	14	77	632	166	10	101	1000	50887	2270
West Bengal	31	65	417	358	89	41	1000	27	92	522	221	27	110	1000	26787	1192
A & N Islands	0	75	378	547	0	0	1000	0	197	610	32	0	160	1000	58	37
Chandigarh	0	0	668	313	19	0	1000	0	0	934	22	29	15	1000	155	79
Dadra & N. Haveli	0	0	74	926	0	0	1000	0	83	869	48	0	0	1000	42	24
Daman & Diu	0	383	617	0	0	0	1000	8	30	913	25	0	25	1000	29	35
Lakshadweep	0	251	517	232	0	0	1000	82	45	873	0	0	0	1000	19	46
Pondicherry	0	82	723	135	61	0	1000	0	96	809	95	0	0	1000	516	74
OENS	10	177	456	207	79	71	1000	46	145	548	87	11	164	1000	2257	1044
GUTs	0	77	696	176	51	0	1000	2	77	831	57	8	26	1000	821	295
all-India	18	74	530	300	55	22	1000	27	103	677	133	13	48	1000	331775	17081
estd. no. of aged persons(00)	1875	7659	54877	31084	5738	2327	103560	6127	23589	154420	30300	2868	10909	228214	X	X
sample aged persons	106	444	2767	1668	331	126	5442	335	1219	7655	1574	162	694	11639	X	X

For peer review only

For peer review only - http://bmjopen.bmj.com/site/about/guidelines.xhtml

Appendix A

Table (18.2): Per 1000 distribution of aged persons with illness or otherwise by their perception about change in state of health for each sex

Rural + Urban Persons

state/ut	aged person with illness							aged person without illness							no. of aged persons	
	compared to previous year							compared to previous year							estd. (00)	sample
	much better	some-what better	nearly the same	some-what worse	worse	n.r.	total	much better	some what better	nearly the same	some what worse	worse	n.r.	total		
(1)	(2)	(3)	(4)	(5)	(6)	(7)	(8)	(9)	(10)	(11)	(12)	(13)	(14)	(15)	(16)	(17)
Andhra Pradesh	5	49	591	301	33	21	1000	19	75	780	99	3	25	1000	50236	2183
Arunachal Pradesh	102	339	88	157	34	280	1000	134	122	207	49	8	481	1000	460	330
Assam	10	75	497	272	68	78	1000	32	109	626	95	15	122	1000	11556	988
Bihar	38	81	454	330	66	32	1000	32	71	647	125	20	105	1000	38403	1904
Chhattisgarh	8	116	527	235	97	18	1000	35	227	570	109	15	43	1000	12690	645
Delhi	7	40	811	131	7	5	1000	8	189	728	63	0	12	1000	5002	451
Goa	7	0	189	793	11	0	1000	99	93	630	178	0	0	1000	1606	123
Gujarat	24	89	636	236	16	0	1000	31	161	725	74	3	5	1000	30551	1337
Haryana	33	56	361	483	67	0	1000	21	77	711	174	10	7	1000	14158	759
Himachal Pradesh	17	79	457	400	40	7	1000	45	71	705	143	5	32	1000	5240	792
Jammu & Kashmir	2	53	459	339	144	2	1000	29	127	724	91	7	22	1000	4545	596
Jharkhand	8	102	579	238	41	33	1000	9	62	638	202	45	44	1000	13505	866
Karnataka	47	111	510	296	34	1	1000	73	121	711	80	3	12	1000	30618	1529
Kerala	9	60	512	343	64	12	1000	14	123	721	101	11	30	1000	35359	1766
Madhya Pradesh	5	77	569	300	37	12	1000	16	91	722	118	11	42	1000	37947	1739
Maharashtra	21	112	579	227	40	22	1000	45	151	678	104	5	18	1000	74193	2701
Manipur	36	68	167	483	148	98	1000	49	115	463	104	6	263	1000	1043	605
Meghalaya	0	162	392	407	0	38	1000	27	125	737	50	9	52	1000	988	271
Mizoram	83	98	193	349	0	277	1000	33	126	274	34	6	528	1000	341	383
Nagaland	231	134	313	282	0	39	1000	16	16	792	65	0	112	1000	224	109
Orissa	30	69	459	377	56	10	1000	35	88	666	160	17	35	1000	28256	1269
Punjab	25	101	494	326	46	8	1000	29	84	784	82	7	14	1000	18274	826
Rajasthan	11	65	509	337	78	0	1000	23	65	772	122	14	4	1000	31284	1672
Sikkim	0	62	371	493	74	0	1000	100	190	621	39	0	50	1000	242	233
Tamil Nadu	40	98	671	182	9	0	1000	76	130	725	65	2	2	1000	51046	2357
Tripura	7	203	623	51	90	26	1000	24	239	571	71	30	65	1000	1869	405

Continued

For peer review only - http://bmjopen.bmj.com/site/about/guidelines.xhtml

Appendix A

Table (18.2): Per 1000 distribution of aged persons with illness or otherwise by their perception about change in state of health for each sex

Rural + Urban Persons

state/ut	aged person with illness							aged person without illness							no. of aged persons	
	compared to previous year							compared to previous year							estd. (00)	sample
	much better	some-what better	nearly the same	some-what worse	worse	n.r.	total	much better	some what better	nearly the same	some what worse	worse	n.r.	total		
(1)	(2)	(3)	(4)	(5)	(6)	(7)	(8)	(9)	(10)	(11)	(12)	(13)	(14)	(15)	(16)	(17)
Uttaranchal	0	113	413	358	116	0	1000	3	60	761	131	5	40	1000	6116	290
Uttar Pradesh	22	72	468	341	59	38	1000	17	87	641	141	12	103	1000	102779	4715
West Bengal	27	71	448	329	94	32	1000	29	120	533	187	20	111	1000	53642	2394
A & N Islands	0	211	552	237	0	0	1000	5	226	670	25	0	73	1000	152	89
Chandigarh	0	82	688	214	15	0	1000	0	27	920	37	11	6	1000	360	165
Dadra & N. Haveli	0	0	271	729	0	0	1000	0	27	865	108	0	0	1000	124	57
Daman & Diu	0	230	735	35	0	0	1000	5	20	923	34	0	17	1000	43	58
Lakshadweep	0	283	479	185	53	0	1000	39	79	849	33	0	0	1000	35	83
Pondicherry	0	77	720	165	38	0	1000	66	122	727	84	0	0	1000	895	141
OENS	31	187	452	196	65	68	1000	43	157	538	68	14	180	1000	5167	2336
GUTs	0	91	698	179	33	0	1000	26	88	808	63	3	12	1000	1609	593
all-India	21	80	528	300	52	20	1000	32	107	683	119	11	48	1000	663779	34831
estd. no. of aged persons(00)	4363	16454	108677	61768	10655	4051	205970	14692	48810	312625	54601	4966	22117	457810	X	X
sample aged persons	231	1007	5458	3339	671	234	10940	805	2678	15792	2901	299	1416	23891	X	X

For peer review only

For peer review only - http://bmjopen.bmj.com/site/about/guidelines.xhtml

Appendix A

Table (19): Number of hospitalisation cases* during last 365 days and number of cases among these where any medical services were provided by the employer by source of treatment for each age-group

All-India Rural

age-group	hospitalised cases receiving medical treatment as inpatient during last 365 days								
	male			female			persons		
	estd. number (00)	no. of cases getting any free medical services from employer		estd. number (00)	no. of cases getting any free medical services from employer		estd. number (00)	no. of cases getting any free medical services from employer	
		govt.	pvt.		govt.	pvt.		govt.	pvt.
(1)	(2)	(3)	(4)	(5)	(6)	(7)	(8)	(9)	(10)
0 – 4	12565	20623	5827	6494	8716	8188	19059	29339	14015
5 – 9	5551	3399	2206	3275	5732	7000	8826	9131	9206
10 – 14	6409	6511	3924	3954	3287	2763	10362	9797	6687
0 – 14	24525	30532	11957	13722	17735	17951	38248	48266	29908
15 – 19	5133	3895	3460	4208	2325	1445	9342	6220	4905
20 – 24	6155	7160	14414	7830	12039	16877	13986	19199	31291
25 – 29	5801	6772	11159	8143	10742	11173	13944	17514	22332
15 – 29	17089	17826	29033	20182	25107	29495	37271	42932	58528
30 – 34	5617	5277	21139	7375	15621	11784	12992	20898	32922
35 – 39	6506	12032	5877	6925	9476	10029	13430	21507	15906
40 – 44	6236	24398	4410	6594	17982	5437	12830	42381	9847
30 – 44	18359	41706	31426	20894	43079	27250	39252	84786	58676
45 – 49	6671	20692	34754	7055	16712	2025	13727	37404	36780
50 – 54	5563	14515	7587	5318	10766	8278	10881	25280	15865
55 – 59	7400	22424	2126	5629	7161	2030	13029	29585	4156
45 – 59	19633	57631	44467	18003	34639	12333	37636	92270	56800
60 & above	19684	24276	15966	14479	28668	9760	34163	52944	25726
all	99290	171971	132849	87280	149227	96789	186570	321198	229639
estd. no. of hosp. cases (00)	99290	1720	1328	87280	1492	968	186570	3212	2296
sample hosp. cases	10967	290	132	9567	285	123	20534	575	255

* cases receiving medical treatment as inpatient

For peer review only

For peer review only - http://bmjopen.bmj.com/site/about/guidelines.xhtml

Appendix A

Table (19): Number of hospitalisation cases* during last 365 days and number of cases among these where any medical services were provided by the employer by source of treatment for each age-group

All-India Urban

age-group	hospitalised cases receiving medical treatment as inpatient during last 365 days								
	male			female			persons		
	estd. number (00)	no. of cases getting any free medical services from employer		estd. number (00)	no. of cases getting any free medical services from employer		estd. number (00)	no. of cases getting any free medical services from employer	
		govt.	pvt.		govt.	pvt.		govt.	pvt.
(1)	(2)	(3)	(4)	(5)	(6)	(7)	(8)	(9)	(10)
0 – 4	4834	15538	8015	2890	7330	4255	7724	22868	12270
5 – 9	2293	20074	302	1695	5009	1415	3988	25083	1718
10 – 14	2011	10068	4680	1634	10275	5218	3645	20342	9898
0 – 14	9138	45680	12997	6219	22614	10888	15357	68293	23886
15 – 19	2632	12706	5845	2130	10364	2456	4761	23070	8301
20 – 24	2555	11414	6146	3726	8849	2915	6282	20263	9061
25 – 29	2147	18785	5526	3438	11890	4740	5586	30675	10266
15 – 29	7334	42905	17517	9294	31103	10112	16629	74008	27629
30 – 34	2220	17941	11646	2654	11116	11295	4874	29057	22940
35 – 39	3151	20917	25790	2964	16486	16638	6114	37403	42428
40 – 44	3429	23077	5621	3125	19054	11326	6554	42131	16947
30 – 44	8800	61935	43057	8743	46656	39258	17542	108591	82315
45 – 49	3583	42866	3868	2955	29562	4986	6537	72428	8854
50 – 54	3463	44612	9150	2312	20446	3106	5775	65058	12256
55 – 59	2875	45316	4131	2747	9031	3294	5622	54347	7425
45 – 59	9921	132794	17149	8014	59039	11386	17935	191833	28535
60 & above	9554	62477	9870	8154	77057	12954	17709	139534	22825
all	44748	345791	100591	40424	236469	84599	85172	582260	185190
estd. no. of hosp. cases (00)	44748	3458	1006	40424	2365	846	85172	5823	1852
sample hosp. cases	6339	494	165	5792	416	119	12131	910	284

* cases receiving medical treatment as inpatient

For peer review only - http://bmjopen.bmj.com/site/about/guidelines.xhtml

Table (19): Number of hospitalisation cases* during last 365 days and number of cases among these where any medical services were provided by the employer by source of treatment for each age-group

All-India Rural+Urban

age-group	hospitalised cases receiving medical treatment as inpatient during last 365 days								
	male			female			persons		
	estd. number (00)	no. of cases getting any free medical services from employer		estd. number (00)	no. of cases getting any free medical services from employer		estd. number (00)	no. of cases getting any free medical services from employer	
		govt.	pvt.		govt.	pvt.		govt.	pvt.
(1)	(2)	(3)	(4)	(5)	(6)	(7)	(8)	(9)	(10)
0 – 4	17399	36160	13842	9384	16046	12442	26783	52206	26284
5 – 9	7844	23473	2509	4970	10741	8415	12814	34214	10924
10 – 14	8420	16578	8604	5588	13561	7981	14008	30140	16586
0 – 14	33663	76212	24955	19942	40348	28839	53605	116560	53793
15 – 19	7765	16600	9305	6338	12689	3901	14103	29290	13206
20 – 24	8711	18574	20560	11557	20888	19793	20267	39462	40353
25 – 29	7948	25557	16685	11582	22632	15913	19529	48189	32598
15 – 29	24423	60731	46550	29476	56209	39607	53900	116940	86157
30 – 34	7837	23218	32784	10029	26737	23079	17866	49955	55863
35 – 39	9656	32949	31667	9888	25962	26667	19545	58911	58335
40 – 44	9665	47475	10031	9719	37036	16762	19384	84511	26794
30 – 44	27159	103641	74483	29636	89735	66508	56795	193377	140991
45 – 49	10254	63558	38622	10010	46274	7012	20264	109833	45634
50 – 54	9026	59126	16736	7630	31212	11385	16656	90338	28121
55 – 59	10275	67740	6257	8377	16192	5324	18651	83932	11581
45 – 59	29555	190425	61616	26016	93678	23720	55571	284103	85336
60 & above	29238	86753	25836	22633	105725	22715	51872	192478	48551
all	144038	517762	233440	127704	385696	181389	271741	903458	414829
estd. no. of hosp. cases (00)	144038	5178	2334	127704	3857	1814	271741	9035	4148
sample hosp. cases	17306	784	297	15359	701	242	32665	1485	539

* cases receiving medical treatment as inpatient

For peer review only - http://bmjopen.bmj.com/site/about/guidelines.xhtml

For peer review only

Appendix A

Table (20): Rate of hospitalisation (number per 1,00,000) during last 365 days by sex and broad age-group

All India　　Rural

age-group	rate of hospitalisation during last 365 days			estd. no. (00)		sample	
	males	females	persons	hospita-lisation cases	persons	hospita-lisation cases	persons
(1)	(2)	(3)	(4)	(5)	(6)	(7)	(8)
0 – 14	1786	1092	1455	38248	2629534	3967	87616
15 – 29	1881	2240	2060	37271	1809280	4266	65376
30 – 44	2657	3032	2844	39252	1380084	4647	46363
45 – 59	4626	4280	4454	37636	845015	4152	29855
60 & above	7551	5698	6636	34163	514787	3502	22768
all	2715	2479	2599	186570	7178701	20534	251978
estd. hospitali-sation cases during 365 days (00)	99290	87280	186570	X	X	X	X
sample hospitali-sation cases during 365 days	10967	9567	20534	X	X	X	X
estd. persons (00)	3657509	3521192	7178701	X	X	X	X
sample persons	128629	123349	251978	X	X	X	X

Table (20): Rate of hospitalisation (number per 1,00,000) during last 365 days by sex and broad age-group

All India　　Urban

age-group	rate of hospitalisation during last 365 days			estd. no. (00)		sample	
	males	females	persons	hospita-lisation cases	persons	hospita-lisation cases	persons
(1)	(2)	(3)	(4)	(5)	(6)	(7)	(8)
0 – 14	2400	1831	2132	15357	720314	2140	38544
15 – 29	1947	2769	2334	16629	712301	2397	37633
30 – 44	3242	3374	3307	17542	530515	2533	27340
45 – 59	5932	5302	5633	17935	318376	2637	16759
60 & above	11842	9695	10746	17709	164795	2424	12801
all	3505	3456	3482	85172	2446300	12131	133077
estd. hospitali-sation cases during 365 days (00)	44748	40424	85172	X	X	X	X
sample hospitali-sation cases during 365 days	6339	5792	12131	X	X	X	X
estd. persons (00)	1276650	1169650	2446300	X	X	X	X
sample persons	68070	65007	133077	X	X	X	X

For peer review only - http://bmjopen.bmj.com/site/about/guidelines.xhtml

Appendix A

Table (20): Rate of hospitalisation (number per 1,00,000) during last 365 days by sex and broad age-group

All India — Rural + Urban

age-group	rate of hospitalisation during last 365 days			estd. no. (00)		sample	
	males	females	persons	hospita-lisation cases	persons	hospita-lisation cases	persons
(1)	(2)	(3)	(4)	(5)	(6)	(7)	(8)
0 – 14	1920	1249	1600	53605	3349848	6107	126160
15 – 29	1900	2384	2138	53900	2521581	6663	103009
30 – 44	2822	3125	2973	56795	1910599	7180	73703
45 – 59	4995	4550	4777	55571	1163390	6789	46614
60 & above	8565	6692	7633	51872	679582	5926	35569
all	2919	2722	2823	271741	9625001	32665	385055
estd. hospitali-sation cases during 365 days (00)	144038	127704	271741	X	X	X	X
sample hospitali-sation cases during 365 days	17306	15359	32665	X	X	X	X
estd. persons (00)	4934159	4690841	9625001	X	X	X	X
sample persons	196699	188356	385055	X	X	X	X

For peer review only

For peer review only - http://bmjopen.bmj.com/site/about/guidelines.xhtml

Appendix A

Table (21): Rate of hospitalised persons (number per 1,00,000) during last 365 days by sex and broad age-group

All India Rural

age-group	rate of hospitalised persons during last 365 days			estd. no. (00)		sample	
	males	females	persons	hospita-lised persons	persons	hospita-lised persons	persons
(1)	(2)	(3)	(4)	(5)	(6)	(7)	(8)
0 – 14	1498	958	1240	32601	2629534	3659	87616
15 – 29	1710	2072	1890	34202	1809280	3986	65376
30 – 44	2331	2722	2526	34859	1380084	4243	46363
45 – 59	4064	3733	3899	32949	845015	3765	29855
60 & above	6303	4894	5607	28867	514787	3094	22768
all	2348	2204	2277	163479	7178701	18747	251978
estd. hospitali-sation cases during 365 days (00)	85885	77594	163479	X	X	X	X
sample hospitali-sation cases during 365 days	9971	8776	18747	X	X	X	X
estd. persons (00)	3657509	3521192	7178701	X	X	X	X
sample persons	128629	123349	251978	X	X	X	X

Table (21): Rate of hospitalised persons (number per 1,00,000) during last 365 days by sex and broad age-group

All India Urban

age-group	rate of hospitalised persons during last 365 days			estd. no. (00)		sample	
	males	females	persons	hospita-lised persons	persons	hospita-lised persons	persons
(1)	(2)	(3)	(4)	(5)	(6)	(7)	(8)
0 – 14	2176	1713	1958	14101	720314	1980	38544
15 – 29	1764	2560	2139	15236	712301	2216	37633
30 – 44	2824	3059	2939	15590	530515	2291	27340
45 – 59	5080	4464	4788	15244	318376	2318	16759
60 & above	10194	8085	9118	15026	164795	2113	12801
all	3079	3068	3074	75196	2446300	10918	133077
estd. hospitali-sation cases during 365 days (00)	39311	35885	75196	X	X	X	X
sample hospitali-sation cases during 365 days	5685	5233	10918	X	X	X	X
estd. persons (00)	1276650	1169650	2446300	X	X	X	X
sample persons	68070	65007	133077	X	X	X	X

For peer review only - http://bmjopen.bmj.com/site/about/guidelines.xhtml

Appendix A

Table (21): Rate of hospitalised persons (number per 1,00,000) during last 365 days by sex and broad age-group

All India — Rural + Urban

age-group	rate of hospitalised persons during last 365 days			estd. no. (00)		sample	
	males	females	persons	hospita-lised persons	persons	hospita-lised persons	persons
(1)	(2)	(3)	(4)	(5)	(6)	(7)	(8)
0 – 14	1645	1118	1394	46702	3349848	5639	126160
15 – 29	1726	2205	1961	49438	2521581	6202	103009
30 – 44	2470	2814	2640	50449	1910599	6534	73703
45 – 59	4352	3926	4142	48193	1163390	6083	46614
60 & above	7223	5688	6459	43892	679582	5207	35569
all	2537	2419	2480	238675	9625001	29665	385055
estd. hospitali-sation cases during 365 days (00)	125196	113479	238675	X	X	X	X
sample hospitali-sation cases during 365 days	15656	14009	29665	X	X	X	X
estd. persons (00)	4934159	4690841	9625001	X	X	X	X
sample persons	196699	188356	385055	X	X	X	X

For peer review only

For peer review only - http://bmjopen.bmj.com/site/about/guidelines.xhtml

Appendix A

Table (22): Per 1000 distribution of hospitalised cases during last 365 days by nature of ailment for different age-groups and sex

All-India Rural Males

nature of ailment	hospitalised cases by age of the person						average duration of stay per case (in 0.0 days)	cases of hospitalisation	
	0-14	15-29	30-44	45-59	60 & above	all		estd. no. (00)	sample
(1)	(2)	(3)	(4)	(5)	(6)	(7)	(8)	(9)	(10)
**gastro-intestinal**									
diarrhoea/ dysentery	151	70	51	38	33	73	5.3	7207	948
gastritis/ gastric or peptic ulcer	15	59	53	51	37	41	8.9	4081	574
worm infestation	6	3	4	2	4	4	6.1	386	47
amoebiosis	5	4	4	1	4	4	11.6	365	43
hepatitis/ jaundice	26	46	15	9	5	20	8.9	1955	241
**cardio-vascular diseases**									
heart disease	10	14	32	94	95	48	11	4770	450
hypertension	0	5	7	31	23	13	5.3	1279	140
respiratory including ear/ nose/ throat ailments	106	17	15	17	25	40	6.4	4007	295
tuberculosis	7	21	59	63	42	37	16.1	3662	469
bronchial asthma	22	7	25	42	111	41	7.8	4118	386
disorders of joints and bones	19	19	25	31	30	25	16.6	2453	273
diseases of kidney/ urinary system	27	35	67	53	78	51	11.1	5085	593
prostatic disorders	2	2	4	4	12	5	10.5	489	75
gynaecological disorders	0	7	2	1	0	2	7.1	182	15
neurological disorders	28	46	25	33	40	34	13	3375	361
psychiatric disorders	3	21	17	15	9	12	16.4	1224	117
**eye ailment**									
conjunctivitis	3	1	1	1	6	2	4.9	247	33
glaucoma	1	2	2	13	14	6	6.3	607	54
cataract	1	0	2	42	77	24	4.6	2412	276
diseases of skin	3	4	10	4	10	6	9	588	70
goitre	1	0	0	1	0	0	7.6	46	8
diabetes mellitus	0	4	12	24	30	14	12.5	1358	140
under-nutrition	2	0	0	0	2	1	7.9	101	11
anaemia	9	5	3	8	2	6	12.5	552	63
sexually transmitted diseases	0	0	1	1	1	0	29	40	8
**febrile illness**									
malaria	42	54	40	26	12	35	5.8	3437	474
eruptive	5	0	1	0	3	2	7.5	205	15
mumps	2	1	0	0	0	1	5.4	65	12
diphtheria	1	0	0	2	0	1	5.2	70	12
whooping cough	15	5	0	8	8	8	4.9	771	62
fever of unknown origin	163	77	63	55	43	84	6.2	8371	794
tetanus	11	4	4	2	0	5	15.8	460	34
filariasis/elephantiasis	1	1	3	0	2	1	7.3	123	14
**disability**									
locomotor	24	11	18	12	17	17	13.1	1685	188
visual including blindness (excluding cataract)	1	2	2	5	12	4	6.9	402	60
speech	1	0	0	0	0	0	15	16	1
hearing	3	4	2	3	0	2	5.3	227	22

Continued

For peer review only - http://bmjopen.bmj.com/site/about/guidelines.xhtml

For peer review only

Appendix A

Table (22): Per 1000 distribution of hospitalised cases during last 365 days by nature of ailment for different age-groups and sex

All-India Rural Males

nature of ailment	hospitalised cases by age of the person						average duration of stay per case (in 0.0 days)	cases of hospitalisation	
	0-14	15-29	30-44	45-59	60 & above	all		estd. no. (00)	sample
(1)	(2)	(3)	(4)	(5)	(6)	(7)	(8)	(9)	(10)
diseases of mouth/teeth/gum	1	1	3	7	2	3	8.3	258	34
accidents/ injuries/ burns/ fractures/ poisoning	101	191	202	136	74	137	13.2	13575	1496
cancer and other tumours	6	36	18	20	18	19	15.5	1845	192
other diagnosed ailments	155	199	190	136	98	154	9.5	15288	1643
other undiagnosed ailments	20	20	18	11	18	18	9.5	1771	198
n.r.	1	1	1	1	3	1	8.9	135	26
total	1000	1000	1000	1000	1000	1000	9.8	99290	10967
estd. no. of hosp. cases(00)	24525	17089	18359	19633	19684	99290	X	X	X
sample hosp. cases	2564	1948	2145	2222	2088	10967	X	X	X

For peer review only - http://bmjopen.bmj.com/site/about/guidelines.xhtml

Appendix A

Table (22): Per 1000 distribution of hospitalised cases during last 365 days by nature of ailment for different age-groups and sex

All-India Rural Females

nature of ailment	hospitalised cases by age of the person						average duration of stay per case (in 0.0 days)	cases of hospitalisation	
	0-14	15-29	30-44	45-59	60 & above	all		estd. no. (00)	sample
(1)	(2)	(3)	(4)	(5)	(6)	(7)	(8)	(9)	(10)
**gastro-intestinal**									
diarrhoea/ dysentery	200	51	52	68	62	80	4.2	6992	830
gastritis/ gastric or peptic ulcer	25	53	68	72	54	57	7.8	4932	539
worm infestation	9	2	5	4	0	4	6.7	344	57
amoebiosis	5	2	2	1	5	3	5.8	230	27
hepatitis/ jaundice	24	9	7	5	4	9	8.7	799	143
**cardio-vascular diseases**									
heart disease	6	16	38	63	59	36	9.3	3185	361
hypertension	0	10	17	46	53	24	8.4	2138	196
respiratory including ear/ nose/ throat ailments	78	28	10	15	25	28	7.2	2484	227
tuberculosis	12	19	22	42	18	23	17.9	2015	242
bronchial asthma	20	9	21	39	47	26	10.3	2265	220
disorders of joints and bones	10	17	23	33	40	24	10	2132	199
diseases of kidney/ urinary system	11	13	28	24	28	21	9.4	1832	246
prostatic disorders	0	3	3	3	0	2	8.3	194	21
gynaecological disorders	1	240	155	66	14	109	8.4	9493	1137
neurological disorders	23	26	31	29	35	29	9.4	2504	281
psychiatric disorders	7	9	12	4	5	8	12.6	667	78
**eye ailment**									
conjunctivitis	0	0	0	3	3	1	9.1	107	21
glaucoma	2	0	0	5	9	3	5.7	249	39
cataract	4	1	8	43	132	34	4.9	2935	295
diseases of skin	11	3	7	2	8	6	30	515	48
goitre	4	0	1	0	1	1	5.4	90	10
diabetes mellitus	13	1	5	48	52	22	8.9	1938	126
under-nutrition	1	1	0	0	1	1	11.5	62	9
anaemia	11	15	17	11	6	12	6.2	1087	134
sexually transmitted diseases	0	4	7	3	0	3	7.6	265	34
**febrile illness**									
malaria	48	32	24	28	10	28	7.1	2441	348
eruptive	3	0	16	0	2	4	2.7	387	10
mumps	2	1	0	2	0	1	3.7	88	7
diphtheria	10	0	2	0	0	2	5	193	14
whooping cough	12	3	4	2	6	5	8.4	441	40
fever of unknown origin	147	68	61	60	44	73	6.7	6373	599
tetanus	3	2	0	0	0	1	7.9	85	14
filariasis/elephantiasis	0	1	2	2	2	1	8.4	121	21
**disability**									
locomotor	10	5	4	11	20	9	11.6	808	97
visual including blindness (excluding cataract)	0	1	1	7	14	4	8.8	373	50
speech	1	5	0	1	0	1	9.5	117	6
hearing	1	2	0	2	0	1	5.5	96	10

Continued

For peer review only - http://bmjopen.bmj.com/site/about/guidelines.xhtml

Appendix A

Table (22): Per 1000 distribution of hospitalised cases during last 365 days by nature of ailment for different age-groups and sex

All-India Rural Females

nature of ailment	hospitalised cases by age of the person						average duration of stay per case (in 0.0 days)	cases of hospitalisation	
	0-14	15-29	30-44	45-59	60 & above	all		estd. no. (00)	sample
(1)	(2)	(3)	(4)	(5)	(6)	(7)	(8)	(9)	(10)
diseases of mouth/teeth/gum	1	2	3	4	1	2	13.8	206	27
accidents/ injuries/ burns/ fractures/ poisoning	87	55	53	51	65	60	10.3	5274	558
cancer and other tumours	11	27	56	58	36	39	14.8	3408	343
other diagnosed ailments	148	242	205	128	127	176	8.4	15334	1655
other undiagnosed ailments	26	22	27	14	13	21	7.4	1813	229
n.r.	11	3	3	0	0	3	148.8	268	19
total	1000	1000	1000	1000	1000	1000	9	87280	9567
estd. no. of hosp. cases(00)	13722	20182	20894	18003	14479	87280	X	X	X
sample hosp. cases	1403	2318	2502	1930	1414	9567	X	X	X

For peer review only - http://bmjopen.bmj.com/site/about/guidelines.xhtml

Appendix A

Table (22): Per 1000 distribution of hospitalised cases during last 365 days by nature of ailment for different age-groups and sex

All-India Rural Persons

nature of ailment	hospitalised cases by age of the person						average duration of stay per case (in 0.0 days)	cases of hospitalisation	
	0-14	15-29	30-44	45-59	60 & above	all		estd. no. (00)	sample
(1)	(2)	(3)	(4)	(5)	(6)	(7)	(8)	(9)	(10)
**gastro-intestinal**									
diarrhoea/ dysentery	168	60	52	52	45	76	4.8	14200	1778
gastritis/ gastric or peptic ulcer	19	56	61	61	44	48	8.3	9013	1113
worm infestation	7	3	5	3	2	4	6.4	729	104
amoebiosis	5	3	3	1	4	3	9.4	595	70
hepatitis/ jaundice	25	26	10	7	5	15	8.9	2753	384
**cardio-vascular diseases**									
heart disease	8	15	35	79	80	43	10.3	7956	811
hypertension	0	8	12	38	36	18	7.2	3417	336
respiratory including ear/ nose/ throat ailments	96	23	13	17	25	35	6.7	6492	522
tuberculosis	8	20	39	53	32	30	16.8	5676	711
bronchial asthma	21	8	23	40	84	34	8.7	6383	606
disorders of joints and bones	16	18	24	32	34	25	13.5	4584	472
diseases of kidney/ urinary system	21	23	46	39	57	37	10.6	6916	839
prostatic disorders	2	3	4	4	7	4	9.9	683	96
gynaecological disorders	0	133	84	32	6	52	8.4	9675	1152
neurological disorders	26	35	28	31	38	32	11.5	5879	642
psychiatric disorders	4	15	15	9	8	10	15.1	1891	195
**eye ailment**									
conjunctivitis	2	0	1	2	5	2	6.2	354	54
glaucoma	1	1	1	9	11	5	6.2	856	93
cataract	2	1	5	43	100	29	4.8	5347	571
diseases of skin	6	3	8	3	9	6	18.8	1102	118
goitre	2	0	0	0	1	1	6.2	136	18
diabetes mellitus	5	3	8	35	40	18	10.4	3297	266
under-nutrition	2	1	0	0	1	1	9.2	163	20
anaemia	10	10	10	9	4	9	8.4	1638	197
sexually transmitted diseases	0	2	4	2	0	2	10.4	305	42
**febrile illness**									
malaria	44	42	32	27	11	32	6.3	5878	822
eruptive	4	0	9	0	2	3	4.4	592	25
mumps	2	1	0	1	0	1	4.4	153	19
diphtheria	4	0	1	1	0	1	5.1	263	26
whooping cough	14	4	2	5	7	6	6.2	1212	102
fever of unknown origin	157	72	62	57	43	79	6.4	14744	1393
tetanus	8	3	2	1	0	3	14.6	545	48
filariasis/elephantiasis	1	1	2	1	2	1	7.8	244	35
**disability**									
locomotor	19	8	10	11	18	13	12.6	2493	285
visual including blindness (excluding cataract)	0	1	2	6	13	4	7.8	775	110
speech	1	2	0	0	0	1	10.1	133	7
hearing	2	3	1	2	0	2	5.3	323	32

Continued

For peer review only - http://bmjopen.bmj.com/site/about/guidelines.xhtml

For peer review only

Appendix A

Table (22): Per 1000 distribution of hospitalised cases during last 365 days by nature of ailment for different age-groups and sex

All-India Rural Persons

nature of ailment	hospitalised cases by age of the person						average duration of stay per case (in 0.0 days)	cases of hospitalisation	
	0-14	15-29	30-44	45-59	60 & above	all		estd. no. (00)	sample
(1)	(2)	(3)	(4)	(5)	(6)	(7)	(8)	(9)	(10)
diseases of mouth/teeth/gum	1	2	3	6	1	2	10.7	464	61
accidents/ injuries/ burns/ fractures/ poisoning	96	117	123	95	70	101	12.4	18849	2054
cancer and other tumours	8	31	38	38	26	28	15.1	5253	535
other diagnosed ailments	153	222	198	132	110	164	9	30622	3298
other undiagnosed ailments	22	22	23	13	16	19	8.4	3584	427
n.r.	5	2	2	0	2	2	102	402	45
total	1000	1000	1000	1000	1000	1000	9.4	186570	20534
estd. no. of hosp. cases(00)	38248	37271	39252	37636	34163	186570	X	X	X
sample hosp. cases	3967	4266	4647	4152	3502	20534	X	X	X

For peer review only - http://bmjopen.bmj.com/site/about/guidelines.xhtml

Appendix A

Table (22): Per 1000 distribution of hospitalised cases during last 365 days by nature of ailment for different age-groups and sex

All-India Urban Males

nature of ailment	hospitalised cases by age of the person						average duration of stay per case (in 0.0 days)	cases of hospitalisation	
	0-14	15-29	30-44	45-59	60 & above	all		estd. no. (00)	sample
(1)	(2)	(3)	(4)	(5)	(6)	(7)	(8)	(9)	(10)
**gastro-intestinal**									
diarrhoea/ dysentery	157	54	59	23	22	62	3.9	2787	435
gastritis/ gastric or peptic ulcer	10	52	64	51	30	41	7.4	1834	311
worm infestation	9	2	4	5	1	4	5.9	199	34
amoebiosis	8	0	3	1	1	3	7.1	123	22
hepatitis/ jaundice	37	46	20	20	14	26	8.7	1185	169
**cardio-vascular diseases**									
heart disease	13	26	61	166	165	91	9.3	4083	584
hypertension	0	12	36	49	50	31	6.3	1366	184
respiratory including ear/ nose/ throat ailments	58	34	8	22	21	28	6.9	1263	175
tuberculosis	3	13	55	27	11	22	16.9	987	127
bronchial asthma	31	7	7	35	80	34	7.7	1505	190
disorders of joints and bones	13	25	28	33	26	25	9.7	1131	156
diseases of kidney/ urinary system	42	44	73	61	89	63	8.2	2809	372
prostatic disorders	1	5	5	10	14	7	8.6	318	49
gynaecological disorders	0	1	0	0	1	0	3.5	21	9
neurological disorders	44	41	27	28	51	38	16.9	1709	254
psychiatric disorders	2	11	6	9	1	6	17.9	253	45
**eye ailment**									
conjunctivitis	2	4	0	6	1	3	6.9	113	14
glaucoma	0	0	0	4	6	2	5.1	92	17
cataract	0	2	1	25	64	20	4.7	889	138
diseases of skin	4	8	7	9	1	6	14.6	261	32
goitre	0	0	6	0	0	1	11.4	51	4
diabetes mellitus	0	6	5	44	68	26	10.1	1185	152
under-nutrition	2	0	0	0	1	1	5.4	32	5
anaemia	8	8	28	7	3	11	3.8	479	42
sexually transmitted diseases	0	1	0	1	0	0	4.3	21	4
**febrile illness**									
malaria	58	67	67	20	4	41	5.1	1843	239
eruptive	0	0	1	1	0	1	5.7	23	5
mumps	0	0	0	0	0	0	10	0	1
diphtheria	5	13	1	8	0	5	6.8	228	12
whooping cough	29	1	4	1	4	8	4.8	357	48
fever of unknown origin	139	95	58	27	25	67	6.6	2987	414
tetanus	3	1	0	2	5	2	16.5	109	8
filariasis/elephantiasis	2	2	1	3	1	2	8.4	82	15
**disability**									
locomotor	3	5	6	13	22	10	13.7	458	83
visual including blindness (excluding cataract)	0	5	0	4	5	3	5.1	125	16
speech	0	0	0	0	0	0	8.2	9	3
hearing	2	0	1	2	2	1	12.7	51	5

Continued

For peer review only - http://bmjopen.bmj.com/site/about/guidelines.xhtml

Appendix A

Table (22): Per 1000 distribution of hospitalised cases during last 365 days by nature of ailment for different age-groups and sex

All-India Urban Males

nature of ailment	hospitalised cases by age of the person						average duration of stay per case (in 0.0 days)	cases of hospitalisation	
	0-14	15-29	30-44	45-59	60 & above	all		estd. no. (00)	sample
(1)	(2)	(3)	(4)	(5)	(6)	(7)	(8)	(9)	(10)
diseases of mouth/teeth/gum	2	2	1	2	0	1	6	62	13
accidents/ injuries/ burns/ fractures/ poisoning	90	198	157	98	52	115	14.8	5128	771
cancer and other tumours	9	31	38	51	25	31	10.3	1387	126
other diagnosed ailments	189	165	150	128	115	148	8.4	6637	969
other undiagnosed ailments	17	12	12	2	11	11	7.6	471	75
n.r.	1	1	0	0	8	2	5.7	93	12
total	1000	1000	1000	1000	1000	1000	9.1	44748	6339
estd. no. of hosp. cases(00)	9138	7334	8800	9921	9554	44748	X	X	X
sample hosp. cases	1296	1063	1202	1426	1352	6339	X	X	X

For peer review only - http://bmjopen.bmj.com/site/about/guidelines.xhtml

Appendix A

Table (22): Per 1000 distribution of hospitalised cases during last 365 days by nature of ailment for different age-groups and sex

All-India Urban Females

nature of ailment	hospitalised cases by age of the person						average duration of stay per case (in 0.0 days)	cases of hospitalisation	
	0-14	15-29	30-44	45-59	60 & above	all		estd. no. (00)	sample
(1)	(2)	(3)	(4)	(5)	(6)	(7)	(8)	(9)	(10)
**gastro-intestinal**									
diarrhoea/ dysentery	138	67	45	46	33	62	4.7	2514	442
gastritis/ gastric or peptic ulcer	8	51	54	41	24	38	7	1517	257
worm infestation	17	1	3	2	2	4	4.2	175	26
amoebiosis	10	2	9	2	1	5	6.6	186	26
hepatitis/ jaundice	57	14	13	3	4	16	7.5	659	83
**cardio-vascular diseases**									
heart disease	15	29	48	80	162	68	9.5	2750	341
hypertension	2	14	38	49	59	33	6.2	1342	195
respiratory including ear/ nose/ throat ailments	63	20	20	30	34	31	7.5	1264	167
tuberculosis	7	22	9	18	5	12	14.9	499	82
bronchial asthma	14	10	19	37	54	27	8.2	1078	158
disorders of joints and bones	15	4	22	48	45	27	12	1079	161
diseases of kidney/ urinary system	26	32	41	34	33	34	8.1	1361	215
prostatic disorders	2	1	3	0	0	1	8.9	44	14
gynaecological disorders	2	239	148	74	18	106	7.7	4270	658
neurological disorders	39	14	27	16	33	25	9.1	1017	161
psychiatric disorders	6	4	4	10	3	6	14.1	225	27
**eye ailment**									
conjunctivitis	0	0	1	3	3	1	7.5	60	10
glaucoma	1	2	4	8	50	13	3.7	526	33
cataract	0	2	8	37	94	28	4	1151	166
diseases of skin	5	7	12	6	1	6	6.2	252	35
goitre	0	2	3	3	0	2	12.3	71	11
diabetes mellitus	0	0	14	51	36	20	7.8	828	125
under-nutrition	15	2	0	0	0	3	7.7	110	10
anaemia	2	11	18	14	5	11	6.3	428	63
sexually transmitted diseases	0	1	1	1	0	1	6.9	27	9
**febrile illness**									
malaria	49	40	39	22	6	31	6.5	1249	202
eruptive	4	2	1	0	0	1	5.6	54	9
mumps	2	1	0	0	0	1	7.6	24	5
diphtheria	22	0	0	0	0	4	3.8	145	8
whooping cough	13	2	2	1	2	4	6.2	142	18
fever of unknown origin	162	77	54	39	31	68	5.7	2756	356
tetanus	5	2	1	0	0	1	12.8	60	4
filariasis/elephantiasis	0	0	1	2	0	1	6.5	25	5
**disability**									
locomotor	7	2	4	7	16	7	12.5	275	43
visual including blindness (excluding cataract)	2	0	1	7	7	3	10.3	130	23
speech	0	0	0	1	0	0	15	11	1
hearing	0	1	2	1	0	1	7.3	26	6

Continued

For peer review only - http://bmjopen.bmj.com/site/about/guidelines.xhtml

Appendix A

Table (22): Per 1000 distribution of hospitalised cases during last 365 days by nature of ailment for different age-groups and sex

All-India Urban Females

nature of ailment	hospitalised cases by age of the person						average duration of stay per case (in 0.0 days)	cases of hospitalisation	
	0-14	15-29	30-44	45-59	60 & above	all		estd. no. (00)	sample
(1)	(2)	(3)	(4)	(5)	(6)	(7)	(8)	(9)	(10)
diseases of mouth/teeth/gum	3	2	1	5	0	2	12	83	17
accidents/ injuries/ burns/ fractures/ poisoning	98	33	49	56	67	58	9.8	2346	294
cancer and other tumours	11	19	34	42	56	33	19.5	1337	191
other diagnosed ailments	165	247	202	192	103	185	7.9	7460	1015
other undiagnosed ailments	13	21	37	11	10	19	7.1	776	103
n.r.	1	1	6	0	2	2	5.5	92	17
total	1000	1000	1000	1000	1000	1000	8	40424	5792
estd. no. of hosp. cases(00)	6219	9294	8743	8014	8154	40424	X	X	X
sample hosp. cases	844	1334	1331	1211	1072	5792	X	X	X

For peer review only - http://bmjopen.bmj.com/site/about/guidelines.xhtml

Appendix A

Table (22): Per 1000 distribution of hospitalised cases during last 365 days by nature of ailment for different age-groups and sex

All-India Urban Persons

nature of ailment	hospitalised cases by age of the person						average duration of stay per case (in 0.0 days)	cases of hospitalisation	
	0-14	15-29	30-44	45-59	60 & above	all		estd. no. (00)	sample
(1)	(2)	(3)	(4)	(5)	(6)	(7)	(8)	(9)	(10)
**gastro-intestinal**									
diarrhoea/ dysentery	150	61	52	33	27	62	4.3	5300	877
gastritis/ gastric or peptic ulcer	9	51	59	47	27	39	7.2	3350	568
worm infestation	12	1	4	4	1	4	5.1	374	60
amoebiosis	9	1	6	2	1	4	6.8	309	48
hepatitis/ jaundice	45	28	17	13	9	22	8.2	1844	252
**cardio-vascular diseases**									
heart disease	14	28	55	128	164	80	9.4	6834	925
hypertension	1	13	37	49	54	32	6.2	2708	379
respiratory including ear/ nose/ throat ailments	60	26	14	25	27	30	7.2	2526	342
tuberculosis	5	18	32	23	8	17	16.3	1486	209
bronchial asthma	24	8	13	36	68	30	7.9	2583	348
disorders of joints and bones	14	13	25	40	35	26	10.8	2210	317
diseases of kidney/ urinary system	36	38	57	49	63	49	8.1	4171	587
prostatic disorders	2	3	4	5	7	4	8.7	362	63
gynaecological disorders	1	134	74	33	9	50	7.7	4291	667
neurological disorders	42	26	27	23	43	32	14	2726	415
psychiatric disorders	3	7	5	10	2	6	16.1	478	72
**eye ailment**									
conjunctivitis	1	2	1	5	2	2	7.1	173	24
glaucoma	0	1	2	6	26	7	3.9	618	50
cataract	0	2	5	30	78	24	4.3	2040	304
diseases of skin	4	8	9	8	1	6	10.5	514	67
goitre	0	1	4	1	0	1	11.9	122	15
diabetes mellitus	0	3	10	47	53	24	9.2	2013	277
under-nutrition	7	1	0	0	1	2	7.2	142	15
anaemia	6	10	23	10	4	11	5	908	105
sexually transmitted diseases	0	1	1	1	0	1	5.8	47	13
**febrile illness**									
malaria	54	52	53	21	5	36	5.7	3092	441
eruptive	2	1	1	1	0	1	5.7	77	14
mumps	1	1	0	0	0	0	7.6	24	6
diphtheria	12	6	0	4	0	4	5.6	373	20
whooping cough	23	1	3	1	3	6	5.2	499	66
fever of unknown origin	149	85	56	32	28	67	6.1	5744	770
tetanus	4	2	0	1	3	2	15.2	169	12
filariasis/elephantiasis	2	1	1	2	1	1	7.9	108	20
**disability**									
locomotor	5	3	5	10	19	9	13.2	733	126
visual including blindness (excluding cataract)	1	2	0	5	6	3	7.8	255	39
speech	0	0	0	1	0	0	11.9	21	4
hearing	1	0	1	1	1	1	10.8	77	11

Continued

For peer review only - http://bmjopen.bmj.com/site/about/guidelines.xhtml

Appendix A

Table (22): Per 1000 distribution of hospitalised cases during last 365 days by nature of ailment for different age-groups and sex

All-India Urban Persons

nature of ailment	hospitalised cases by age of the person						average duration of stay per case (in 0.0 days)	cases of hospitalisation	
	0-14	15-29	30-44	45-59	60 & above	all		estd. no. (00)	sample
(1)	(2)	(3)	(4)	(5)	(6)	(7)	(8)	(9)	(10)
diseases of mouth/teeth/gum	2	2	1	4	0	2	9.4	145	30
accidents/ injuries/ burns/ fractures/ poisoning	93	106	103	80	59	88	13.2	7473	1065
cancer and other tumours	10	24	36	47	39	32	14.8	2724	317
other diagnosed ailments	179	211	176	156	110	166	8.1	14097	1984
other undiagnosed ailments	16	17	25	6	10	15	7.3	1248	178
n.r.	1	1	3	0	5	2	5.6	185	29
total	1000	1000	1000	1000	1000	1000	8.6	85172	12131
estd. no. of hosp. cases(00)	15357	16629	17542	17935	17709	85172	X	X	X
sample hosp. cases	2140	2397	2533	2637	2424	12131	X	X	X

For peer review only - http://bmjopen.bmj.com/site/about/guidelines.xhtml

Appendix A

Table (22): Per 1000 distribution of hospitalised cases during last 365 days by nature of ailment for different age-groups and sex

All-India Rural + Urban Males

nature of ailment	hospitalised cases by age of the person						average duration of stay per case (in 0.0 days)	cases of hospitalisation	
	0-14	15-29	30-44	45-59	60 & above	all		estd. no. (00)	sample
(1)	(2)	(3)	(4)	(5)	(6)	(7)	(8)	(9)	(10)
**gastro-intestinal**									
diarrhoea/ dysentery	152	65	54	33	29	69	4.9	9994	1383
gastritis/ gastric or peptic ulcer	14	57	57	51	34	41	8.4	5914	885
worm infestation	7	3	4	3	3	4	6	585	81
amoebiosis	6	3	3	1	3	3	10.5	488	65
hepatitis/ jaundice	29	46	16	13	8	22	8.8	3140	410
**cardio-vascular diseases**									
heart disease	11	18	41	118	118	61	10.2	8853	1034
hypertension	0	7	16	37	32	18	5.8	2645	324
respiratory including ear/ nose/ throat ailments	93	22	13	19	24	37	6.5	5270	470
tuberculosis	6	18	58	51	32	32	16.3	4649	596
bronchial asthma	24	7	19	40	101	39	7.7	5623	576
disorders of joints and bones	18	21	26	32	29	25	14.4	3584	429
diseases of kidney/ urinary system	31	38	69	56	82	55	10	7894	965
prostatic disorders	2	3	4	6	12	6	9.8	807	124
gynaecological disorders	0	5	2	1	0	1	6.7	203	24
neurological disorders	33	45	26	31	44	35	14.3	5084	615
psychiatric disorders	2	18	14	13	7	10	16.7	1476	162
**eye ailment**									
conjunctivitis	3	2	1	3	4	2	5.6	360	47
glaucoma	1	1	1	10	11	5	6.2	699	71
cataract	1	1	2	37	73	23	4.6	3301	414
diseases of skin	3	5	9	6	7	6	10.8	849	102
goitre	1	0	2	0	0	1	9.6	97	12
diabetes mellitus	0	5	9	31	43	18	11.4	2543	292
under-nutrition	2	0	0	0	2	1	7.3	132	16
anaemia	9	6	11	8	3	7	8.4	1031	105
sexually transmitted diseases	0	1	1	1	0	0	20.5	60	12
**febrile illness**									
malaria	47	58	49	24	9	37	5.6	5280	713
eruptive	3	0	1	1	2	2	7.3	228	20
mumps	1	0	0	0	0	0	5.4	65	13
diphtheria	2	4	0	4	0	2	6.4	298	24
whooping cough	19	4	1	5	7	8	4.9	1128	110
fever of unknown origin	156	82	61	45	37	79	6.3	11358	1208
tetanus	9	3	2	2	2	4	15.9	569	42
filariasis/elephantiasis	1	1	2	1	2	1	7.7	205	29
**disability**									
locomotor	19	10	14	12	19	15	13.2	2144	271
visual including blindness (excluding cataract)	1	3	1	4	9	4	6.5	527	76
speech	1	0	0	0	0	0	12.5	25	4
hearing	2	3	2	2	1	2	6.6	278	27

Continued

For peer review only

For peer review only - http://bmjopen.bmj.com/site/about/guidelines.xhtml

Appendix A

Table (22): Per 1000 distribution of hospitalised cases during last 365 days by nature of ailment for different age-groups and sex

All-India Rural + Urban Males

nature of ailment	hospitalised cases by age of the person						average duration of stay per case (in 0.0 days)	cases of hospitalisation	
	0-14	15-29	30-44	45-59	60 & above	all		estd. no. (00)	sample
(1)	(2)	(3)	(4)	(5)	(6)	(7)	(8)	(9)	(10)
diseases of mouth/teeth/gum	1	1	2	5	1	2	7.9	320	47
accidents/ injuries/ burns/ fractures/ poisoning	98	193	187	123	67	130	13.6	18703	2267
cancer and other tumours	7	34	24	30	20	22	13.3	3232	318
other diagnosed ailments	165	189	177	133	103	152	9.2	21925	2612
other undiagnosed ailments	20	18	16	8	16	16	9.1	2242	273
n.r.	1	1	1	1	5	2	7.6	228	38
total	1000	1000	1000	1000	1000	1000	9.6	144038	17306
estd. no. of hosp. cases(00)	33663	24423	27159	29555	29238	144038	X	X	X
sample hosp. cases	3860	3011	3347	3648	3440	17306	X	X	X

For peer review only - http://bmjopen.bmj.com/site/about/guidelines.xhtml

Appendix A

Table (22): Per 1000 distribution of hospitalised cases during last 365 days by nature of ailment for different age-groups and sex

All-India Rural + Urban Females

nature of ailment	hospitalised cases by age of the person						average duration of stay per case (in 0.0 days)	cases of hospitalisation	
	0-14	15-29	30-44	45-59	60 & above	all		estd. no. (00)	sample
(1)	(2)	(3)	(4)	(5)	(6)	(7)	(8)	(9)	(10)
**gastro-intestinal**									
diarrhoea/ dysentery	181	56	50	61	52	74	4.4	9506	1272
gastritis/ gastric or peptic ulcer	20	53	64	63	43	50	7.6	6449	796
worm infestation	11	2	4	3	1	4	5.9	519	83
amoebiosis	7	2	4	1	4	3	6.1	416	53
hepatitis/ jaundice	35	10	9	4	4	11	8.1	1457	226
**cardio-vascular diseases**									
heart disease	9	20	41	68	96	46	9.4	5936	702
hypertension	0	11	23	47	55	27	7.5	3480	391
respiratory including ear/ nose/ throat ailments	73	25	13	20	28	29	7.3	3748	394
tuberculosis	10	20	18	34	14	20	17.3	2513	324
bronchial asthma	18	9	20	38	49	26	9.6	3343	378
disorders of joints and bones	11	13	23	38	42	25	10.7	3210	360
diseases of kidney/ urinary system	16	19	32	27	30	25	8.9	3193	461
prostatic disorders	1	2	3	2	0	2	8.4	238	35
gynaecological disorders	1	240	153	68	16	108	8.2	13763	1795
neurological disorders	28	22	30	25	34	28	9.3	3522	442
psychiatric disorders	7	8	10	6	4	7	13	893	105
**eye ailment**									
conjunctivitis	0	0	1	3	3	1	8.5	167	31
glaucoma	2	0	1	6	24	6	4.3	775	72
cataract	3	1	8	41	118	32	4.7	4085	461
diseases of skin	9	4	9	3	5	6	22.2	767	83
goitre	2	1	1	1	1	1	8.5	161	21
diabetes mellitus	9	1	8	49	46	22	8.6	2766	251
under-nutrition	6	1	0	0	1	1	9	172	19
anaemia	8	13	17	12	6	12	6.3	1515	197
sexually transmitted diseases	0	3	5	2	0	2	7.6	292	43
**febrile illness**									
malaria	48	34	29	26	9	29	6.9	3690	550
eruptive	3	1	11	0	1	3	3.1	441	19
mumps	2	1	0	2	0	1	4.6	112	12
diphtheria	14	0	2	0	0	3	4.5	339	22
whooping cough	13	2	4	2	4	5	7.9	583	58
fever of unknown origin	152	71	59	54	39	71	6.4	9129	955
tetanus	4	2	0	0	0	1	9.9	145	18
filariasis/elephantiasis	0	0	2	2	1	1	8	147	26
**disability**									
locomotor	9	4	4	10	18	8	11.8	1083	140
visual including blindness (excluding cataract)	1	1	1	7	11	4	9.2	503	73
speech	1	3	0	1	0	1	9.9	129	7
hearing	1	1	1	2	0	1	5.9	122	16

Continued

For peer review only - http://bmjopen.bmj.com/site/about/guidelines.xhtml

Appendix A

Table (22): Per 1000 distribution of hospitalised cases during last 365 days by nature of ailment for different age-groups and sex

All-India Rural + Urban Females

nature of ailment	hospitalised cases by age of the person						average duration of stay per case (in 0.0 days)	cases of hospitalisation	
	0-14	15-29	30-44	45-59	60 & above	all		estd. no. (00)	sample
(1)	(2)	(3)	(4)	(5)	(6)	(7)	(8)	(9)	(10)
diseases of mouth/teeth/gum	2	2	2	5	0	2	13.3	289	44
accidents/ injuries/ burns/ fractures/ poisoning	91	48	52	53	66	60	10.1	7619	852
cancer and other tumours	11	24	49	53	43	37	16.1	4745	534
other diagnosed ailments	153	243	204	148	119	178	8.2	22794	2670
other undiagnosed ailments	22	22	30	13	12	20	7.3	2589	332
n.r.	8	2	4	0	1	3	112.3	359	36
total	1000	1000	1000	1000	1000	1000	8.7	127704	15359
estd. no. of hosp. cases(00)	19942	29476	29636	26016	22633	127704	X	X	X
sample hosp. cases	2247	3652	3833	3141	2486	15359	X	X	X

For peer review only - http://bmjopen.bmj.com/site/about/guidelines.xhtml

Appendix A

Table (22): Per 1000 distribution of hospitalised cases during last 365 days by nature of ailment for different age-groups and sex

All-India Rural + Urban Persons

nature of ailment	hospitalised cases by age of the person						average duration of stay per case (in 0.0 days)	cases of hospitalisation	
	0-14	15-29	30-44	45-59	60 & above	all		estd. no. (00)	sample
(1)	(2)	(3)	(4)	(5)	(6)	(7)	(8)	(9)	(10)
**gastro-intestinal**									
diarrhoea/ dysentery	163	60	52	46	39	72	4.7	19500	2655
gastritis/ gastric or peptic ulcer	16	54	61	57	38	45	8	12363	1681
worm infestation	9	2	4	3	2	4	6	1103	164
amoebiosis	6	2	4	1	3	3	8.5	904	118
hepatitis/ jaundice	31	26	12	9	6	17	8.6	4597	636
**cardio-vascular diseases**									
heart disease	10	19	41	95	109	54	9.9	14789	1736
hypertension	0	9	20	42	42	23	6.8	6125	715
respiratory including ear/ nose/ throat ailments	86	24	13	19	26	33	6.9	9018	864
tuberculosis	7	19	37	43	24	26	16.7	7162	920
bronchial asthma	22	8	20	39	78	33	8.4	8966	954
disorders of joints and bones	15	17	24	34	34	25	12.6	6794	789
diseases of kidney/ urinary system	26	28	50	42	59	41	9.7	11087	1426
prostatic disorders	2	3	4	4	7	4	9.5	1046	159
gynaecological disorders	1	134	81	32	7	51	8.2	13967	1819
neurological disorders	31	32	28	28	40	32	12.3	8606	1057
psychiatric disorders	4	12	12	9	6	9	15.3	2369	267
**eye ailment**									
conjunctivitis	2	1	1	3	4	2	6.5	527	78
glaucoma	1	1	1	8	16	5	5.2	1474	143
cataract	2	1	5	39	92	27	4.7	7386	875
diseases of skin	6	5	9	5	6	6	16.2	1616	185
goitre	1	1	1	1	0	1	8.9	258	33
diabetes mellitus	4	3	9	39	44	20	9.9	5309	543
under-nutrition	3	1	0	0	1	1	8.3	304	35
anaemia	8	10	14	10	4	9	7.2	2546	302
sexually transmitted diseases	0	2	3	1	0	1	9.8	352	55
**febrile illness**									
malaria	47	45	38	25	9	33	6.1	8970	1263
eruptive	3	1	6	0	2	2	4.5	669	39
mumps	2	1	0	1	0	1	4.9	177	25
diphtheria	7	2	1	2	0	2	5.4	636	46
whooping cough	17	3	3	4	6	6	5.9	1711	168
fever of unknown origin	155	76	60	49	38	75	6.3	20487	2163
tetanus	7	2	1	1	1	3	14.7	714	60
filariasis/elephantiasis	1	1	2	1	2	1	7.9	351	55
**disability**									
locomotor	15	7	9	11	18	12	12.8	3226	411
visual including blindness (excluding cataract)	1	2	1	6	10	4	7.8	1030	149
speech	1	2	0	0	0	1	10.4	154	11
hearing	2	2	1	2	0	1	6.4	400	43

Continued

For peer review only

For peer review only - http://bmjopen.bmj.com/site/about/guidelines.xhtml

Appendix A

Table (22): Per 1000 distribution of hospitalised cases during last 365 days by nature of ailment for different age-groups and sex

All-India Rural + Urban Persons

nature of ailment	hospitalised cases by age of the person						average duration of stay per case (in 0.0 days)	cases of hospitalisation	
	0-14	15-29	30-44	45-59	60 & above	all		estd. no. (00)	sample
(1)	(2)	(3)	(4)	(5)	(6)	(7)	(8)	(9)	(10)
diseases of mouth/teeth/gum	2	2	2	5	1	2	10.4	609	91
accidents/ injuries/ burns/ fractures/ poisoning	95	114	117	90	66	97	12.6	26322	3119
cancer and other tumours	8	29	37	41	30	29	15	7977	852
other diagnosed ailments	160	219	191	140	110	165	8.7	44719	5282
other undiagnosed ailments	20	20	23	11	14	18	8.1	4831	605
n.r.	4	2	2	0	3	2	71.6	587	74
total	1000	1000	1000	1000	1000	1000	9.2	271741	32665
estd. no. of hosp. cases(00)	53605	53900	56795	55571	51872	271741	X	X	X
sample hosp. cases	6107	6663	7180	6789	5926	32665	X	X	X

For peer review only - http://bmjopen.bmj.com/site/about/guidelines.xhtml

Appendix A

Table (23): Per 1000 distribution of hospitalisation cases by type of hospital and type of ward for each mpce class and sex

All-India Rural Males

mpce class	type of hospital									cases of hospitalisation	
	public hospital+ public dispensary				private hospital				total (incl. n.r. cases)	estd. no. (00)	sample
	type of ward				type of ward						
	free ward	paying general ward	paying special ward	all (incl. n.r. cases)	free ward	paying general ward	paying special ward	all (incl. n.r. cases)			
(1)	(2)	(3)	(4)	(5)	(6)	(7)	(8)	(9)	(10)	(11)	(12)
0 – 225	561	35	0	596	37	324	39	400	1000	3080	427
225 – 255	514	69	0	583	13	372	30	415	1000	2130	304
255 – 300	436	93	3	532	32	393	40	465	1000	5692	764
300 – 340	439	52	0	491	41	389	76	506	1000	6401	787
340 – 380	396	59	9	464	58	431	46	536	1000	7313	848
380 – 420	477	39	3	520	14	402	64	479	1000	9633	1007
420 – 470	366	98	7	472	15	430	83	527	1000	8529	1038
470 – 525	375	88	6	469	34	414	81	530	1000	10480	1202
525 – 615	349	45	4	398	29	470	101	600	1000	12360	1354
615 – 775	264	67	4	336	19	555	90	664	1000	14954	1484
775 – 950	268	51	19	338	15	507	139	662	1000	7186	760
950 +	161	61	15	236	3	501	260	764	1000	11533	992
all	350	64	7	420	24	454	101	579	1000	99290	10967
estd. no. of hosp. cases (00)	34712	6308	661	41690	2408	45084	10000	57491	99290	X	X
sample no. of hosp. cases	4434	784	69	5291	273	4583	798	5654	10967	X	X

For peer review only - http://bmjopen.bmj.com/site/about/guidelines.xhtml

Appendix A

Table (23): Per 1000 distribution of hospitalisation cases by type of hospital and type of ward for each mpce class and sex

All-India	Rural									Females	
mpce class	type of hospital									cases of hospitalisation	
	public hospital+ public dispensary				private hospital				total (incl. n.r. cases)	estd. no. (00)	sample
	type of ward				type of ward						
	free ward	paying general ward	paying special ward	all (incl. n.r. cases)	free ward	paying general ward	paying special ward	all (incl. n.r. cases)			
(1)	(2)	(3)	(4)	(5)	(6)	(7)	(8)	(9)	(10)	(11)	(12)
0 – 225	558	64	1	676	50	235	11	297	1000	2872	331
225 – 255	507	37	0	544	16	401	39	456	1000	2563	267
255 – 300	479	63	1	543	30	394	31	455	1000	4744	632
300 – 340	440	51	0	492	40	432	27	499	1000	5145	638
340 – 380	424	84	2	510	44	423	21	488	1000	6206	723
380 – 420	437	69	3	508	23	407	61	492	1000	7597	879
420 – 470	331	69	2	402	31	499	66	597	1000	8211	939
470 – 525	368	46	2	416	29	459	96	584	1000	10220	1127
525 – 615	341	48	3	392	20	501	83	605	1000	10177	1129
615 – 775	265	60	3	328	15	504	153	672	1000	12654	1301
775 – 950	283	45	13	341	39	479	141	659	1000	7000	706
950 +	188	48	16	252	16	427	305	748	1000	9891	895
all	350	57	4	413	27	451	107	585	1000	87280	9567
estd. no. of hosp. cases (00)	30521	4958	379	36022	2360	39367	9335	51064	87280	X	X
sample no. of hosp. cases	3827	625	41	4500	232	4052	762	5047	9567	X	X

For peer review only - http://bmjopen.bmj.com/site/about/guidelines.xhtml

Appendix A

Table (23): Per 1000 distribution of hospitalisation cases by type of hospital and type of ward for each mpce class and sex

All-India Rural Persons

mpce class	type of hospital									cases of hospitalisation	
	public hospital+ public dispensary				private hospital				total (incl. n.r. cases)	estd. no. (00)	sample
	type of ward				type of ward						
	free ward	paying general ward	paying special ward	all (incl. n.r. cases)	free ward	paying general ward	paying special ward	all (incl. n.r. cases)			
(1)	(2)	(3)	(4)	(5)	(6)	(7)	(8)	(9)	(10)	(11)	(12)
0 – 225	560	49	1	635	43	281	26	350	1000	5952	758
225 – 255	510	52	0	562	15	388	35	437	1000	4693	571
255 – 300	456	79	2	537	31	393	36	461	1000	10436	1396
300 – 340	439	51	0	491	41	408	54	503	1000	11546	1425
340 – 380	409	71	6	485	52	428	35	514	1000	13519	1571
380 – 420	460	52	3	515	18	404	63	485	1000	17230	1886
420 – 470	349	84	5	438	23	464	75	561	1000	16740	1977
470 – 525	371	67	4	443	31	437	89	557	1000	20700	2329
525 – 615	345	46	3	395	25	484	93	602	1000	22538	2483
615 – 775	264	64	4	332	17	532	119	668	1000	27607	2785
775 – 950	275	48	16	339	27	493	140	660	1000	14186	1466
950 +	173	55	15	244	9	467	281	756	1000	21424	1887
all	350	60	6	417	26	453	104	582	1000	186570	20534
estd. no. of hosp. cases (00)	65233	11266	1040	77712	4767	84450	19334	108555	186570	X	X
sample no. of hosp. cases	8261	1409	110	9791	505	8635	1560	10701	20534	X	X

For peer review only - http://bmjopen.bmj.com/site/about/guidelines.xhtml

Appendix A

Table (23): Per 1000 distribution of hospitalisation cases by type of hospital and type of ward for each mpce class and sex

All-India Urban Males

mpce class	type of hospital									cases of hospitalisation	
	public hospital+ public dispensary				private hospital				total (incl. n.r. cases)	estd. no. (00)	sample
	type of ward				type of ward						
	free ward	paying general ward	paying special ward	all (incl. n.r. cases)	free ward	paying general ward	paying special ward	all (incl. n.r. cases)			
(1)	(2)	(3)	(4)	(5)	(6)	(7)	(8)	(9)	(10)	(11)	(12)
0 –300	517	56	22	595	7	336	61	405	1000	1257	180
300 – 350	559	50	0	610	4	365	22	390	1000	743	115
350 – 425	490	69	6	565	10	356	67	435	1000	2240	327
425 – 500	452	79	1	532	11	413	44	468	1000	4502	606
500 – 575	387	62	15	464	13	366	156	536	1000	2184	313
575 – 665	307	61	21	388	10	516	86	612	1000	3918	534
665 – 775	323	66	4	393	12	496	96	605	1000	5848	806
775 – 915	316	82	7	406	12	413	169	594	1000	4982	730
915 – 1120	292	53	18	363	12	485	140	637	1000	5424	838
1120 – 1500	219	69	15	303	13	428	256	697	1000	7520	1053
1500 – 1925	168	64	13	245	10	360	384	755	1000	2358	295
1925 +	115	62	18	196	10	371	424	804	1000	3771	542
all	308	67	12	386	11	430	172	614	1000	44748	6339
estd. no. of hosp. cases (00)	13775	2981	526	17282	509	19231	7709	27458	44748	X	X
sample no. of hosp. cases	2269	459	68	2798	99	2536	900	3537	6339	X	X

For peer review only - http://bmjopen.bmj.com/site/about/guidelines.xhtml

Table (23): Per 1000 distribution of hospitalisation cases by type of hospital and type of ward for each mpce class and sex

All-India Urban Females

mpce class	type of hospital									cases of hospitalisation	
	public hospital+ public dispensary				private hospital				total (incl. n.r. cases)	estd. no. (00)	sample
	type of ward				type of ward						
	free ward	paying general ward	paying special ward	all (incl. n.r. cases)	free ward	paying general ward	paying special ward	all (incl. n.r. cases)			
(1)	(2)	(3)	(4)	(5)	(6)	(7)	(8)	(9)	(10)	(11)	(12)
0 –300	435	90	31	556	43	326	76	444	1000	890	130
300 – 350	570	14	0	584	20	378	18	416	1000	755	99
350 – 425	483	67	13	564	42	361	30	436	1000	2052	293
425 – 500	460	97	1	558	18	374	46	438	1000	3854	550
500 – 575	496	72	22	590	30	327	53	410	1000	2100	287
575 – 665	326	57	1	383	20	504	92	616	1000	4187	525
665 – 775	366	86	6	459	25	414	101	541	1000	4422	637
775 – 915	285	76	15	376	6	486	132	624	1000	4745	703
915 – 1120	283	65	11	360	28	498	115	640	1000	5611	834
1120 – 1500	183	63	8	254	15	473	259	746	1000	5769	870
1500 – 1925	94	44	17	154	15	475	356	846	1000	2050	311
1925 +	86	37	22	146	17	362	475	854	1000	3988	553
all	300	67	11	378	21	436	165	622	1000	40424	5792
estd. no. of hosp. cases (00)	12140	2697	444	15282	840	17631	6651	25126	40424	X	X
sample no. of hosp. cases	1982	416	71	2470	121	2334	860	3316	5792	X	X

For peer review only - http://bmjopen.bmj.com/site/about/guidelines.xhtml

Table (23): Per 1000 distribution of hospitalisation cases by type of hospital and type of ward for each mpce class and sex

All-India Urban Persons

mpce class	type of hospital									cases of hospitalisation	
	public hospital+ public dispensary				private hospital				total (incl. n.r. cases)	estd. no. (00)	sample
	type of ward				type of ward						
	free ward	paying general ward	paying special ward	all (incl. n.r. cases)	free ward	paying general ward	paying special ward	all (incl. n.r. cases)			
(1)	(2)	(3)	(4)	(5)	(6)	(7)	(8)	(9)	(10)	(11)	(12)
0 –300	483	70	26	579	22	332	67	421	1000	2148	310
300 – 350	565	32	0	597	12	372	20	403	1000	1498	214
350 – 425	487	68	9	564	26	359	49	435	1000	4292	620
425 – 500	456	87	1	544	14	395	45	455	1000	8357	1156
500 – 575	440	67	19	526	21	347	106	474	1000	4284	600
575 – 665	317	59	10	386	15	510	89	614	1000	8105	1059
665 – 775	342	75	5	422	18	461	98	578	1000	10270	1443
775 – 915	301	79	11	391	9	448	151	609	1000	9727	1433
915 – 1120	288	59	14	361	20	492	127	639	1000	11035	1672
1120 – 1500	203	66	12	281	14	447	257	719	1000	13288	1923
1500 – 1925	133	55	15	203	12	413	371	797	1000	4408	606
1925 +	100	50	20	170	14	366	450	830	1000	7759	1095
all	304	67	11	382	16	433	169	617	1000	85172	12131
estd. no. of hosp. cases (00)	25915	5678	970	32565	1349	36862	14360	52585	85172	X	X
sample no. of hosp. cases	4251	875	139	5268	220	4870	1760	6853	12131	X	X

For peer review only - http://bmjopen.bmj.com/site/about/guidelines.xhtml

For peer review only

Table (23): Per 1000 distribution of hospitalisation cases by type of hospital and type of ward for each mpce class and sex

All-India Rural + Urban Males

mpce class	type of hospital									cases of hospitalisation	
	public hospital+ public dispensary				private hospital				total (incl. n.r. cases)	estd. no. (00)	sample
	type of ward				type of ward						
	free ward	paying general ward	paying special ward	all (incl. n.r. cases)	free ward	paying general ward	paying special ward	all (incl. n.r. cases)			
(1)	(2)	(3)	(4)	(5)	(6)	(7)	(8)	(9)	(10)	(11)	(12)
0 – 235	548	41	6	596	28	328	45	401	1000	4338	607
235 – 265	526	64	0	590	11	370	28	409	1000	2873	419
265 – 320	451	86	4	541	26	383	48	457	1000	7932	1091
320 – 365	445	63	0	508	29	399	63	491	1000	10903	1393
365 – 410	394	60	10	464	48	416	71	536	1000	9497	1161
410 – 460	428	45	8	482	12	435	70	518	1000	13551	1541
460 – 520	349	85	6	440	14	457	88	559	1000	14377	1844
520 – 605	356	86	6	449	27	414	109	550	1000	15461	1932
605 – 730	332	47	8	387	24	474	113	611	1000	17784	2192
730 – 980	249	68	8	325	17	513	146	675	1000	22473	2537
980 – 1285	244	54	18	315	14	471	200	685	1000	9544	1055
1285 +	150	61	16	226	5	469	300	774	1000	15304	1534
all	337	64	8	409	20	447	123	590	1000	144038	17306
estd. no. of hosp. cases (00)	48487	9289	1187	58973	2917	64315	17708	84949	144038	X	X
sample no. of hosp. cases	6703	1243	137	8089	372	7119	1698	9191	17306	X	X

For peer review only

For peer review only - http://bmjopen.bmj.com/site/about/guidelines.xhtml

Appendix A

Table (23): Per 1000 distribution of hospitalisation cases by type of hospital and type of ward for each mpce class and sex

All-India Rural + Urban Females

mpce class	type of hospital									cases of hospitalisation	
	public hospital+ public dispensary				private hospital				total (incl. n.r. cases)	estd. no. (00)	sample
	type of ward				type of ward						
	free ward	paying general ward	paying special ward	all (incl. n.r. cases)	free ward	paying general ward	paying special ward	all (incl. n.r. cases)			
(1)	(2)	(3)	(4)	(5)	(6)	(7)	(8)	(9)	(10)	(11)	(12)
0 – 235	529	70	8	648	48	257	26	332	1000	3762	461
235 – 265	521	32	0	553	17	396	34	447	1000	3318	366
265 – 320	481	64	4	549	34	384	31	450	1000	6796	925
320 – 365	448	71	1	520	31	407	35	473	1000	8999	1188
365 – 410	442	81	7	530	41	399	29	469	1000	8306	1010
410 – 460	397	64	2	464	22	442	72	536	1000	11784	1404
460 – 520	344	75	3	422	29	470	78	577	1000	12633	1576
520 – 605	342	56	6	403	22	468	108	597	1000	14966	1830
605 – 730	320	54	6	381	23	500	94	617	1000	15788	1963
730 – 980	239	61	4	305	15	494	186	695	1000	18422	2171
980 – 1285	240	44	14	298	33	478	190	702	1000	9050	1017
1285 +	159	45	18	221	16	408	354	779	1000	13879	1448
all	334	60	6	402	25	446	125	597	1000	127704	15359
estd. no. of hosp. cases (00)	42661	7655	823	51304	3199	56998	15986	76190	127704	X	X
sample no. of hosp. cases	5809	1041	112	6970	353	6386	1622	8363	15359	X	X

For peer review only - http://bmjopen.bmj.com/site/about/guidelines.xhtml

Appendix A

Table (23): Per 1000 distribution of hospitalisation cases by type of hospital and type of ward for each mpce class and sex

All-India					Rural + Urban						Persons
mpce class	type of hospital									cases of hospitalisation	
	public hospital+ public dispensary				private hospital				total (incl. n.r. cases)	estd. no. (00)	sample
	type of ward				type of ward						
	free ward	paying general ward	paying special ward	all (incl. n.r. cases)	free ward	paying general ward	paying special ward	all (incl. n.r. cases)			
(1)	(2)	(3)	(4)	(5)	(6)	(7)	(8)	(9)	(10)	(11)	(12)
0 – 235	539	55	7	620	37	295	37	369	1000	8100	1068
235 – 265	523	47	0	570	14	384	31	429	1000	6191	785
265 – 320	465	76	4	545	30	383	40	453	1000	14728	2016
320 – 365	446	66	1	513	30	403	50	483	1000	19902	2581
365 – 410	416	70	9	495	44	408	52	504	1000	17803	2171
410 – 460	414	54	5	474	17	438	71	526	1000	25336	2945
460 – 520	346	80	5	431	21	463	84	568	1000	27010	3420
520 – 605	349	71	6	426	24	440	108	573	1000	30427	3762
605 – 730	327	51	7	384	23	486	104	614	1000	33572	4155
730 – 980	244	65	6	316	16	504	164	684	1000	40895	4708
980 – 1285	242	49	16	307	23	474	195	693	1000	18594	2072
1285 +	154	54	17	224	10	440	326	776	1000	29183	2982
all	335	62	7	406	23	446	124	593	1000	271741	32665
estd. no. of hosp. cases (00)	91148	16945	2010	110277	6116	121313	33694	161139	271741	X	X
sample no. of hosp. cases	12512	2284	249	15059	725	13505	3320	17554	32665	X	X

For peer review only - http://bmjopen.bmj.com/site/about/guidelines.xhtml

For peer review only

Table (24): Per 1000 distribution of hospitalised cases receiving treatment during last 365 days by time when admitted into hospital and by time when discharged from hospital for each type of hospital and sex of patient

All-India Rural

type of hospital	cases receiving treatment as inpatient in a hospital										hospitalisation cases receiving treatment	
	admitted					discharged					estd. no. (00)	sam-ple
	during last 15 days	16 days to 365 days ago	more than 365 days ago	n.r.	total	not yet	during last 15 days	16 days to 365 days ago	n.r.	total		
(1)	(2)	(3)	(4)	(5)	(6)	(7)	(8)	(9)	(10)	(11)	(12)	(13)
males												
public hospital	35	954	11	0	1000	23	103	873	0	1000	40223	5094
public dispensary	9	988	3	0	1000	12	72	915	0	1000	1467	197
private hospital	40	945	15	0	1000	14	110	876	1	1000	57491	5654
all	37	949	13	1	1000	18	107	875	1	1000	99290	10967
females												
public hospital	30	960	10	0	1000	19	104	876	0	1000	34558	4347
public dispensary	26	968	6	0	1000	2	197	800	0	1000	1464	153
private hospital	32	957	11	0	1000	11	97	892	0	1000	51064	5047
all	31	957	11	1	1000	14	101	883	1	1000	87280	9567
persons												
public hospital	33	957	10	0	1000	21	104	875	0	1000	74781	9441
public dispensary	18	978	4	0	1000	7	135	858	0	1000	2931	350
private hospital	36	951	13	0	1000	12	104	883	0	1000	108555	10701
all	34	953	12	1	1000	16	104	879	1	1000	186570	20534

For peer review only

For peer review only - http://bmjopen.bmj.com/site/about/guidelines.xhtml

Appendix A

Table (24): Per 1000 distribution of hospitalised cases receiving treatment during last 365 days by time when admitted into hospital and by time when discharged from hospital for each type of hospital and sex of patient

All-India Urban

type of hospital	cases receiving treatment as inpatient in a hospital										hospitalisation cases receiving treatment	
	admitted					discharged					estd. no. (00)	sam-ple
	during last 15 days	16 days to 365 days ago	more than 365 days ago	n.r.	total	not yet	during last 15 days	16 days to 365 days ago	n.r.	total		
(1)	(2)	(3)	(4)	(5)	(6)	(7)	(8)	(9)	(10)	(11)	(12)	(13)
males												
public hospital	36	951	12	0	1000	22	73	905	0	1000	15871	2614
public dispensary	79	905	17	0	1000	16	135	849	0	1000	1412	184
private hospital	38	946	16	0	1000	15	83	902	0	1000	27458	3537
all	39	946	15	0	1000	17	81	901	0	1000	44748	6339
females												
public hospital	31	963	5	1	1000	12	87	901	0	1000	14157	2303
public dispensary	35	944	20	0	1000	34	79	888	0	1000	1125	167
private hospital	49	941	10	0	1000	13	98	889	0	1000	25126	3316
all	43	949	9	0	1000	13	93	893	0	1000	40424	5792
persons												
public hospital	34	957	9	0	1000	17	80	903	0	1000	30028	4917
public dispensary	59	922	18	0	1000	24	110	866	0	1000	2537	351
private hospital	44	943	13	0	1000	14	90	896	0	1000	52585	6853
all	41	948	12	0	1000	15	87	897	0	1000	85172	12131

For peer review only

For peer review only - http://bmjopen.bmj.com/site/about/guidelines.xhtml

Appendix A

Table (24): Per 1000 distribution of hospitalised cases receiving treatment during last 365 days by time when admitted into hospital and by time when discharged from hospital for each type of hospital and sex of patient

All-India | Rural + Urban

type of hospital	cases receiving treatment as inpatient in a hospital										hospitalisation cases receiving treatment	
	admitted					discharged					estd. no. (00)	sam-ple
	during last 15 days	16 days to 365 days ago	more than 365 days ago	n.r.	total	not yet	during last 15 days	16 days to 365 days ago	n.r.	total		
(1)	(2)	(3)	(4)	(5)	(6)	(7)	(8)	(9)	(10)	(11)	(12)	(13)
**males**												
public hospital	35	953	11	0	1000	23	95	882	0	1000	56094	7708
public dispensary	43	947	9	0	1000	14	103	883	0	1000	2879	381
private hospital	39	946	15	0	1000	14	101	884	1	1000	84949	9191
all	38	948	14	0	1000	17	99	883	1	1000	144038	17306
**females**												
public hospital	30	961	8	0	1000	17	99	883	0	1000	48715	6650
public dispensary	30	958	12	0	1000	16	146	838	0	1000	2589	320
private hospital	37	952	11	0	1000	11	97	891	0	1000	76190	8363
all	35	955	10	1	1000	14	99	887	1	1000	127704	15359
**persons**												
public hospital	33	957	10	0	1000	20	97	883	0	1000	104809	14358
public dispensary	37	952	11	0	1000	15	123	862	0	1000	5468	701
private hospital	38	948	13	0	1000	13	99	887	0	1000	161139	17554
all	36	951	12	1	1000	16	99	885	1	1000	271741	32665

For peer review only

For peer review only - http://bmjopen.bmj.com/site/about/guidelines.xhtml

Appendix A

Table (25): Per 1000 distribution of hospitalisation cases by duration of stay in hospital and average duration of stay in hospital (in 0.0 days) for each type of hospital

All-India
Rural

type of hospital	duration of stay in hospital								hospitalised cases	
	less than 7 days	7 to less than 15 days	15 to less than 30 days	30 to less than 90 days	90 to less than 180 days	180 to 365 days	total	average duration of stay in hosp. (0.0 days)	estd. no. (00)	sample
(1)	(2)	(3)	(4)	(5)	(6)	(7)	(8)	(9)	(10)	(11)
public hospital (incl. PHC/ sub centres / CHC)	516	284	126	61	11	3	1000	11	74781	9441
public dispensary (incl.CGHS/ESI)	509	293	148	42	6	2	1000	9.7	2931	350
public sector hospital	516	285	126	60	10	3	1000	10.9	77712	9791
private hospital	558	297	105	35	4	1	1000	8.3	108555	10701
all	540	292	114	46	7	2	1000	9.4	186570	20534
estd. no. of hosp. cases (00)	100760	54442	21234	8508	1283	343	186570	X	X	X
sample hosp. cases	10435	6170	2606	1109	163	51	20534	X	X	X

For peer review only - http://bmjopen.bmj.com/site/about/guidelines.xhtml

Table (25): Per 1000 distribution of hospitalisation cases by duration of stay in hospital and average duration of stay in hospital (in 0.0 days) for each type of hospital

All-India
Urban

type of hospital	duration of stay in hospital								hospitalised cases	
	less than 7 days	7 to less than 15 days	15 to less than 30 days	30 to less than 90 days	90 to less than 180 days	180 to 365 days	total	average duration of stay in hosp. (0.0 days)	estd. no. (00)	sample
(1)	(2)	(3)	(4)	(5)	(6)	(7)	(8)	(9)	(10)	(11)
public hospital (incl. PHC/ sub centres / CHC)	519	290	118	56	13	4	1000	10.9	30028	4917
public dispensary (incl.CGHS/ESI)	511	301	126	55	6	0	1000	9.4	2537	351
public sector hospital	518	291	119	56	13	3	1000	10.8	32565	5268
private hospital	619	268	88	21	4	0	1000	7.3	52585	6853
all	580	277	100	34	7	2	1000	8.6	85172	12131
estd. no. of hosp. cases (00)	49425	23566	8497	2928	627	129	85172	X	X	X
sample hosp. cases	6704	3458	1359	522	77	11	12131	X	X	X

For peer review only - http://bmjopen.bmj.com/site/about/guidelines.xhtml

Appendix A

Table (25): Per 1000 distribution of hospitalisation cases by duration of stay in hospital and average duration of stay in hospital (in 0.0 days) for each type of hospital

All-India Urban   Rural +

type of hospital	duration of stay in hospital								hospitalised cases	
	less than 7 days	7 to less than 15 days	15 to less than 30 days	30 to less than 90 days	90 to less than 180 days	180 to 365 days	total	average duration of stay in hosp. (0.0 days)	estd. no. (00)	sample
(1)	(2)	(3)	(4)	(5)	(6)	(7)	(8)	(9)	(10)	(11)
public hospital (incl. PHC/ sub centres / CHC)	517	286	123	60	11	3	1000	11	104809	14358
public dispensary (incl.CGHS/ESI)	510	297	138	48	6	1	1000	9.6	5468	701
public sector hospital	516	287	124	59	11	3	1000	10.9	110277	15059
private hospital	578	287	100	31	4	1	1000	8	161139	17554
all	553	287	109	42	7	2	1000	9.2	271741	32665
estd. no. of hosp. cases (00)	150185	78009	29730	11436	1910	472	271741	X	X	X
sample hosp. cases	17139	9628	3965	1631	240	62	32665	X	X	X

For peer review only - http://bmjopen.bmj.com/site/about/guidelines.xhtml

Table (26): Per 1000 distribution of hospitalisation cases by duration of stay in hospital and average duration of stay in hospital separately for government and private hospitals and for each mpce class

All-India Rural

mpce classes	government hospital: duration of stay in hospital							private hospitals: duration of stay in hospital							total	average duration of stay (0.0 days)		hospitalised cases	
	less than 7 days	7 to less than 15 days	15 to less than 30 days	30 to less than 90 day	90 to less than 180 days	180 to 365 days	all	less than 7 days	7 to less than 15 days	15 to less than 30 days	30 to less than 90 days	90 to less than 180 days	180 to 365 days	all		in govt. hosp	in pvt. hosp.	estd. no. (00)	sam-ple
(1)	(2)	(3)	(4)	(5)	(6)	(7)	(8)	(9)	(10)	(11)	(12)	(13)	(14)	(15)	(16)	(17)	(18)	(19)	(20)
0 – 225	335	166	68	35	18	13	635	193	86	54	17	0	0	350	1000	19.2	8.5	5952	758
225 – 255	330	127	64	24	17	0	562	309	88	31	7	2	0	437	1000	11.1	6.6	4693	571
255 – 300	304	158	45	23	6	1	537	233	156	44	24	3	0	461	1000	9.2	9	10436	1396
300 – 340	250	139	60	34	8	0	491	258	167	54	23	2	0	503	1000	10.9	8.6	11546	1425
340 – 380	256	133	52	37	6	2	485	282	164	49	16	1	2	514	1000	12	8.2	13519	1571
380 – 420	270	153	63	25	4	1	515	274	147	43	17	3	1	485	1000	9.6	8.1	17230	1886
420 – 470	229	120	55	30	2	1	438	308	159	64	27	3	1	561	1000	10.6	9	16740	1977
470 – 525	231	128	57	25	2	0	443	309	161	67	18	0	0	557	1000	9.2	8	20700	2329
525 – 615	185	122	56	26	4	3	395	350	171	61	17	2	1	602	1000	11.8	8.2	22538	2483
615 – 775	165	98	45	21	3	0	332	386	186	70	21	5	0	668	1000	10.9	8.1	27607	2785
775 – 950	175	91	48	24	1	1	339	336	213	86	22	3	1	660	1000	10.4	9	14186	1466
950 +	119	68	40	14	2	0	244	428	230	68	27	3	1	756	1000	10.3	8.3	21424	1887
all	215	119	53	25	4	1	417	325	173	61	21	3	1	582	1000	10.9	8.3	186570	20534
estd. no. of hosp. cases (00)	40063	22119	9819	4674	814	224	77712	60543	32198	11408	3834	469	102	108555	186570	X	X	X	X
sample hosp. cases	4893	2797	1338	628	105	30	9791	5516	3363	1265	481	58	18	10701	20534	X	X	X	X

For peer review only - http://bmjopen.bmj.com/site/about/guidelines.xhtml

For peer review only

Table (26): Per 1000 distribution of hospitalisation cases by duration of stay in hospital and average duration of stay in hospital separately for government and private hospitals and for each mpce class

All India Urban

mpce classes	government hospital							private hospitals							total	average duration of stay (0.0 days)		hospitalised cases	
	duration of stay in hospital							duration of stay in hospital											
	less than 7 days	7 to less than 15 days	15 to less than 30 days	30 to less than 90 day	90 to less than 180 days	180 to 365 days	all	less than 7 days	7 to less than 15 days	15 to less than 30 days	30 to less than 90 days	90 to less than 180 days	180 to 365 days	all		in govt. hosp	in pvt. hosp.	estd. no. (00)	sam-ple
(1)	(2)	(3)	(4)	(5)	(6)	(7)	(8)	(9)	(10)	(11)	(12)	(13)	(14)	(15)	(16)	(17)	(18)	(19)	(20)
0 –300	396	114	48	14	7	0	579	261	97	55	8	0	0	421	1000	7.4	7.2	2148	310
300 – 350	358	165	45	28	0	0	597	230	131	23	11	0	9	403	1000	7.6	15.2	1498	214
350 – 425	332	144	50	18	7	15	564	257	133	34	12	0	0	435	1000	14.5	7.1	4292	620
425 – 500	228	187	72	41	15	0	544	262	131	48	14	0	0	455	1000	12.6	7.1	8357	1156
500 – 575	311	113	65	30	7	0	526	309	132	23	10	0	0	474	1000	10.2	5.9	4284	600
575 – 665	169	131	58	23	4	1	386	423	141	36	13	0	1	614	1000	11.1	6.3	8105	1059
665 – 775	210	131	48	26	3	3	422	329	174	61	9	5	0	578	1000	10.8	7.8	10270	1443
775 – 915	207	108	47	21	8	0	391	364	181	52	10	1	0	609	1000	11.1	6.8	9727	1433
915 – 1120	198	98	45	19	1	0	361	394	155	70	17	1	0	639	1000	9.1	7.4	11035	1672
1120 – 1500	148	87	30	14	2	0	281	467	176	60	10	5	0	719	1000	9.4	7.1	13288	1923
1500 – 1925	103	54	22	24	0	0	203	485	232	58	17	6	0	797	1000	10.1	7.6	4408	606
1925 +	87	48	24	7	4	0	170	514	216	74	21	5	0	830	1000	10.9	7.6	7759	1095
all	198	111	45	21	5	1	382	382	165	54	13	2	0	617	1000	10.8	7.3	85172	12131
estd. no. of hosp. cases (00)	16869	9480	3864	1831	415	106	32565	32543	14076	4633	1097	211	23	52585	85172	X	X	X	X
sample hosp. cases	2691	1524	674	314	58	7	5268	4005	1932	685	208	19	4	6853	12131	X	X	X	X

For peer review only - http://bmjopen.bmj.com/site/about/guidelines.xhtml

For peer review only

Appendix A

Table (26): Per 1000 distribution of hospitalisation cases by duration of stay in hospital and average duration of stay in hospital separately for government and private hospitals and for each mpce class

All India | Rural + Urban

mpce classes	government hospital							private hospitals							total	average duration of stay (0.0 days)		hospitalised cases	
	duration of stay in hospital							duration of stay in hospital											
	less than 7 days	7 to less than 15 days	15 to less than 30 days	30 to less than 90 day	90 to less than 180 days	180 to 365 days	all	less than 7 days	7 to less than 15 days	15 to less than 30 days	30 to less than 90 days	90 to less than 180 days	180 to 365 days	all		in govt. hosp.	in pvt. hosp.	estd. no. (00)	sam-ple
(1)	(2)	(3)	(4)	(5)	(6)	(7)	(8)	(9)	(10)	(11)	(12)	(13)	(14)	(15)	(16)	(17)	(18)	(19)	(20)
0 – 235	351	152	62	30	15	9	620	211	89	54	15	0	0	369	1000	16.3	8.1	8100	1068
235 – 265	337	136	59	25	13	0	570	290	99	29	8	1	2	429	1000	10.2	8.5	6191	785
265 – 320	312	154	46	21	6	5	545	240	150	41	21	2	0	453	1000	10.8	8.5	14728	2016
320 – 365	241	159	65	37	11	0	513	260	152	51	19	1	0	483	1000	11.7	8	19902	2581
365 – 410	269	128	55	35	6	2	495	289	156	43	15	1	1	504	1000	11.5	7.7	17803	2171
410 – 460	238	146	61	24	4	1	474	321	145	41	16	2	1	526	1000	10	7.5	25336	2945
460 – 520	222	124	53	28	3	2	431	316	165	63	20	3	0	568	1000	10.7	8.6	27010	3420
520 – 605	223	122	54	24	4	0	426	327	168	63	16	1	0	573	1000	9.8	7.6	30427	3762
605 – 730	189	114	52	24	3	2	384	364	166	64	17	2	1	614	1000	11	7.9	33572	4155
730 – 980	159	95	40	18	3	0	316	412	183	67	18	5	0	684	1000	10.4	7.8	40895	4708
980 – 1285	158	82	42	24	1	1	307	371	217	79	21	4	1	693	1000	10.4	8.6	18594	2072
1285 +	110	63	36	12	3	0	224	451	226	70	25	4	1	776	1000	10.4	8.1	29183	2982
all	210	116	50	24	5	1	406	343	170	59	18	3	0	593	1000	10.9	8	271741	32665
estd. no. of hosp. cases (00)	56932	31599	13683	6504	1229	330	110277	93086	46274	16042	4931	680	125	161139	271741	X	X	X	X
sample hosp. cases	7584	4321	2012	942	163	37	15059	9521	5295	1950	689	77	22	17554	32665	X	X	X	X

For peer review only

For peer review only - http://bmjopen.bmj.com/site/about/guidelines.xhtml

Appendix A

Table (27): Number per 1000 cases of hospitalisation receiving specific types of medical service and their break-up by payment category for different types of hospital

All-India | Rural | Males

type of hospital	medical services received as inpatient																			
	no. per 1000 cases recg. surgery	surgery				no. per 1000 cases recg. medi-cine	medicine				no. per 1000 cases recg. X-ray/ ECG/ EEG/Scan	X-ray/ECG/ EEG/Scan				no. per 1000 cases recg. other diag-nostic tests	other diagnostic tests			
		service received			all		service received			all		service received			all		service received			all
		free	partly free	on pay-ment			free	partly free	on pay-ment			free	partly free	on pay-ment			free	partly free	on pay-ment	
(1)	(2)	(3)	(4)	(5)	(6)	(7)	(8)	(9)	(10)	(11)	(12)	(13)	(14)	(15)	(16)	(17)	(18)	(19)	(20)	(21)
public hosp.	200	707	86	208	1000	963	185	416	399	1000	499	293	160	547	1000	702	373	153	474	1000
public dis.	237	585	185	230	1000	993	191	304	505	1000	584	316	154	530	1000	796	390	72	538	1000
private hos.	230	40	21	939	1000	979	8	18	974	1000	598	13	10	977	1000	773	15	11	973	1000
all	218	296	48	656	1000	972	82	182	736	1000	557	119	67	814	1000	744	158	66	776	1000
estd. no. of hosp. cases (00)	21636	6410	1035	14191	21636	96527	7917	17586	71024	96527	55337	6598	3690	45050	55337	73875	11667	4900	57309	73875
sample hosp. cases	2628	765	133	1730	2628	10539	824	2027	7688	10539	6253	776	467	5010	6253	8113	1378	601	6134	8113

For peer review only

For peer review only - http://bmjopen.bmj.com/site/about/guidelines.xhtml

Appendix A

Table (27): Number per 1000 cases of hospitalisation receiving specific types of medical service and their break-up by payment category for different types of hospital

All-India Rural Females

type of hospital	medical services received as inpatient																			
	no. per 1000 cases recg. surgery	surgery				no. per 1000 cases recg. medi-cine	medicine				no. per 1000 cases recg. X-ray/ ECG/ EEG/Scan	X-ray/ECG/ EEG/Scan				no. per 1000 cases recg. other diag-nostic tests	other diagnostic tests			
		service received			all		service received			all		service received			all		service received			all
		free	partly free	on pay-ment			free	partly free	on pay-ment			free	partly free	on pay-ment			free	partly free	on pay-ment	
(1)	(2)	(3)	(4)	(5)	(6)	(7)	(8)	(9)	(10)	(11)	(12)	(13)	(14)	(15)	(16)	(17)	(18)	(19)	(20)	(21)
public hosp.	208	700	122	178	1000	970	196	400	405	1000	451	284	158	557	1000	685	383	141	476	1000
public dis.	262	406	177	417	1000	980	223	256	521	1000	492	285	155	560	1000	763	430	65	505	1000
private hos.	266	71	9	921	1000	972	19	11	970	1000	585	9	9	983	1000	777	17	6	977	1000
all	244	294	50	655	1000	970	93	169	738	1000	530	106	61	832	1000	739	158	57	785	1000
estd. no. of hosp. cases (00)	21266	6262	1065	13938	21266	84657	7843	14331	62484	84657	46219	4911	2833	38474	46219	64530	10199	3653	50677	64530
sample hosp. cases	2618	714	125	1779	2618	9224	768	1708	6748	9224	5309	610	354	4345	5309	7138	1197	493	5448	7138

For peer review only - http://bmjopen.bmj.com/site/about/guidelines.xhtml

Appendix A

Table (27): Number per 1000 cases of hospitalisation receiving specific types of medical service and their break-up by payment category for different types of hospital

All-India | Rural | Persons

type of hospital	medical services received as inpatient																			
	no. per 1000 cases recg. surgery	surgery				no. per 1000 cases recg. medi-cine	medicine				no. per 1000 cases recg. X-ray/ ECG/ EEG /Scan	X-ray/ECG/ EEG/Scan				no. per 1000 cases recg. other diag-nostic tests	other diagnostic tests			
		service received			all		service received			all		service received			all		service received			all
		free	partly free	on pay-ment			free	partly free	on pay-ment			free	partly free	on pay-ment			free	partly free	on pay-ment	
(1)	(2)	(3)	(4)	(5)	(6)	(7)	(8)	(9)	(10)	(11)	(12)	(13)	(14)	(15)	(16)	(17)	(18)	(19)	(20)	(21)
public hosp.	204	704	103	193	1000	966	190	409	401	1000	477	289	159	552	1000	694	378	148	475	1000
public dis.	250	491	181	328	1000	987	206	281	513	1000	538	302	154	544	1000	780	409	68	522	1000
private hos.	247	56	15	930	1000	976	14	15	972	1000	592	11	9	980	1000	775	16	9	975	1000
all	230	295	49	656	1000	971	87	176	737	1000	544	113	64	822	1000	742	158	62	780	1000
estd. no. of hosp. cases (00)	42901	12673	2100	28129	42901	181184	15760	31917	133508	181184	101555	11508	6523	83524	101555	138404	21866	8553	107986	138404
sample hosp. cases	5246	1479	258	3509	5246	19763	1592	3735	14436	19763	11562	1386	821	9355	11562	15251	2575	1094	11582	15251

For peer review only - http://bmjopen.bmj.com/site/about/guidelines.xhtml

Appendix A

Table (27): Number per 1000 cases of hospitalisation receiving specific types of medical service and their break-up by payment category for different types of hospital

All-India Urban Males

type of hospital	medical services received as inpatient																			
	no. per 1000 cases recg. surgery	surgery				no. per 1000 cases recg. medi-cine	medicine				no. per 1000 cases recg. X-ray/ ECG/ EEG /Scan	X-ray/ECG/ EEG/Scan				no. per 1000 cases recg. other diag-nostic tests	other diagnostic tests			
		service received			all		service received			all		service received			all		service received			all
		free	partly free	on pay-ment			free	partly free	on pay-ment			free	partly free	on pay-ment			free	partly free	on pay-ment	
(1)	(2)	(3)	(4)	(5)	(6)	(7)	(8)	(9)	(10)	(11)	(12)	(13)	(14)	(15)	(16)	(17)	(18)	(19)	(20)	(21)
public hosp.	194	657	135	208	1000	967	256	378	365	1000	586	395	165	441	1000	739	441	163	396	1000
public dis.	226	792	26	181	1000	983	311	436	253	1000	626	592	134	275	1000	809	610	156	233	1000
private hos.	254	34	16	950	1000	972	13	15	972	1000	655	20	8	972	1000	845	19	9	971	1000
all	232	243	52	705	1000	971	109	157	735	1000	629	162	64	774	1000	806	175	64	761	1000
estd. no. of hosp. cases (00)	10380	2520	538	7322	10380	43438	4722	6807	31909	43438	28161	4555	1801	21805	28161	36083	6319	2311	27453	36083
sample hosp. cases	1581	401	78	1102	1581	6165	675	995	4495	6165	4069	656	285	3128	4069	5063	928	357	3778	5063

For peer review only

For peer review only - http://bmjopen.bmj.com/site/about/guidelines.xhtml

Appendix A

Table (27): Number per 1000 cases of hospitalisation receiving specific types of medical service and their break-up by payment category for different types of hospital

All-India | Urban | Females

type of hospital	medical services received as inpatient																			
	no. per 1000 cases recg. surgery	surgery				no. per 1000 cases recg. medi-cine	medicine				no. per 1000 cases recg. X-ray/ ECG/ EEG /Scan	X-ray/ECG/ EEG/Scan				no. per 1000 cases recg. other diag-nostic tests	other diagnostic tests			
		service received			all		service received			all		service received			all		service received			all
		free	partly free	on pay-ment			free	partly free	on pay-ment			free	partly free	on pay-ment			free	partly free	on pay-ment	
(1)	(2)	(3)	(4)	(5)	(6)	(7)	(8)	(9)	(10)	(11)	(12)	(13)	(14)	(15)	(16)	(17)	(18)	(19)	(20)	(21)
public hosp.	234	667	108	225	1000	980	234	365	401	1000	550	379	171	450	1000	729	448	153	399	1000
public dis.	301	858	23	119	1000	976	359	347	294	1000	613	485	191	324	1000	794	484	218	298	1000
private hos.	300	27	8	964	1000	972	13	14	973	1000	628	13	10	977	1000	836	15	8	977	1000
all	277	242	38	720	1000	975	100	147	753	1000	600	144	67	789	1000	797	166	60	773	1000
estd. no. of hosp. cases (00)	11187	2704	427	8056	11187	39409	3959	5785	29665	39409	24260	3491	1617	19152	24260	32231	5363	1946	24922	32231
sample hosp. cases	1726	454	70	1202	1726	5612	590	829	4193	5612	3587	552	242	2793	3587	4659	860	281	3518	4659

For peer review only - http://bmjopen.bmj.com/site/about/guidelines.xhtml

For peer review only

Table (27): Number per 1000 cases of hospitalisation receiving specific types of medical service and their break-up by payment category for different types of hospital

All-India Urban Persons

type of hospital	medical services received as inpatient																			
	no. per 1000 cases recg. surgery	surgery				no. per 1000 cases recg. medi-cine	medicine				no. per 1000 cases recg. X-ray/ ECG/ EEG /Scan	X-ray/ECG/ EEG/Scan				no. per 1000 cases recg. other diag-nostic tests	other diagnostic tests			
		service received			all		service received			all		service received			all		service received			all
		free	partly free	on pay-ment			free	partly free	on pay-ment			free	partly free	on pay-ment			free	partly free	on pay-ment	
(1)	(2)	(3)	(4)	(5)	(6)	(7)	(8)	(9)	(10)	(11)	(12)	(13)	(14)	(15)	(16)	(17)	(18)	(19)	(20)	(21)
public hosp.	213	662	121	217	1000	973	246	372	382	1000	569	387	168	445	1000	734	444	159	397	1000
public dis.	259	826	25	149	1000	980	332	397	271	1000	621	545	159	296	1000	802	555	183	262	1000
private hos.	276	31	12	957	1000	972	13	14	973	1000	642	17	9	974	1000	841	17	9	974	1000
all	253	242	45	713	1000	973	105	152	743	1000	615	153	65	781	1000	802	171	62	767	1000
estd. no. of hosp. cases (00)	21567	5224	965	15378	21567	82847	8681	12592	61574	82847	52420	8046	3418	40956	52420	68314	11682	4258	52374	68314
sample hosp. cases	3307	855	148	2304	3307	11777	1265	1824	8688	11777	7656	1208	527	5921	7656	9722	1788	638	7296	9722

For peer review only - http://bmjopen.bmj.com/site/about/guidelines.xhtml

Table (27): Number per 1000 cases of hospitalisation receiving specific types of medical service and their break-up by payment category for different types of hospital

All-India | Rural +Urban | Males

type of hospital	medical services received as inpatient																			
	no. per 1000 cases recg. surgery	surgery				no. per 1000 cases recg. medi-cine	medicine				no. per 1000 cases recg. X-ray/ ECG/ EEG /Scan	X-ray/ECG/ EEG/Scan				no. per 1000 cases recg. other diag-nostic tests	other diagnostic tests			
		service received			all		service received			all		service received			all		service received			all
		free	partly free	on pay-ment			free	partly free	on pay-ment			free	partly free	on pay-ment			free	partly free	on pay-ment	
(1)	(2)	(3)	(4)	(5)	(6)	(7)	(8)	(9)	(10)	(11)	(12)	(13)	(14)	(15)	(16)	(17)	(18)	(19)	(20)	(21)
public hosp.	198	693	99	208	1000	964	205	406	389	1000	524	325	162	513	1000	712	393	156	451	1000
public dis.	232	684	109	207	1000	988	249	369	382	1000	605	456	143	400	1000	803	499	114	388	1000
private hos.	238	38	20	942	1000	977	10	17	973	1000	616	15	9	975	1000	796	17	11	973	1000
all	222	279	49	672	1000	972	90	174	735	1000	580	134	66	801	1000	763	164	66	771	1000
estd. no. of hosp. cases (00)	32016	8930	1573	21513	32016	139965	12639	24393	102933	139965	83497	11152	5491	66854	83497	109958	17985	7211	84761	109958
sample hosp. cases	4209	1166	211	2832	4209	16704	1499	3022	12183	16704	10322	1432	752	8138	10322	13176	2306	958	9912	13176

For peer review only - http://bmjopen.bmj.com/site/about/guidelines.xhtml

Appendix A

Table (27): Number per 1000 cases of hospitalisation receiving specific types of medical service and their break-up by payment category for different types of hospital

All-India Rural +Urban Females

type of hospital	medical services received as inpatient																			
	no. per 1000 cases recg. surgery	surgery				no. per 1000 cases recg. medi-cine	medicine				no. per 1000 cases recg. X-ray/ ECG/ EEG /Scan	X-ray/ECG/ EEG/Scan				no. per 1000 cases recg. other diag-nostic tests	other diagnostic tests			
		service received			all		service received			all		service received			all		service received			all
		free	partly free	on pay-ment			free	partly free	on pay-ment			free	partly free	on pay-ment			free	partly free	on pay-ment	
(1)	(2)	(3)	(4)	(5)	(6)	(7)	(8)	(9)	(10)	(11)	(12)	(13)	(14)	(15)	(16)	(17)	(18)	(19)	(20)	(21)
public hosp.	216	690	118	192	1000	973	207	390	403	1000	480	316	162	522	1000	698	402	145	453	1000
public dis.	279	617	105	278	1000	979	282	296	422	1000	545	383	173	445	1000	777	454	133	413	1000
private hos.	277	55	8	936	1000	972	17	12	971	1000	599	10	9	981	1000	797	16	7	977	1000
all	254	276	46	678	1000	972	95	162	743	1000	552	119	63	818	1000	758	161	58	781	1000
estd. no. of hosp. cases (00)	32452	8966	1492	21994	32452	124067	11802	20116	92149	124067	70478	8402	4450	57626	70478	96761	15562	5600	75599	96761
sample hosp. cases	4344	1168	195	2981	4344	14836	1358	2537	10941	14836	8896	1162	596	7138	8896	11797	2057	774	8966	11797

For peer review only - http://bmjopen.bmj.com/site/about/guidelines.xhtml

Appendix A

Table (27): Number per 1000 cases of hospitalisation receiving specific types of medical service and their break-up by payment category for different types of hospital

All-India — Rural +Urban — Persons

type of hospital	medical services received as inpatient																			
	no. per 1000 cases recg. surgery	surgery				no. per 1000 cases recg. medi-cine	medicine				no. per 1000 cases recg. X-ray/ ECG/ EEG /Scan	X-ray/ECG/ EEG/Scan				no. per 1000 cases recg. other diag-nostic tests	other diagnostic tests			
		service received			all		service received			all		service received			all		service received			all
		free	partly free	on pay-ment			free	partly free	on pay-ment			free	partly free	on pay-ment			free	partly free	on pay-ment	
(1)	(2)	(3)	(4)	(5)	(6)	(7)	(8)	(9)	(10)	(11)	(12)	(13)	(14)	(15)	(16)	(17)	(18)	(19)	(20)	(21)
public hosp.	206	691	108	200	1000	968	206	398	396	1000	503	321	162	517	1000	705	397	151	452	1000
public dis.	254	649	107	243	1000	984	265	334	401	1000	576	423	157	420	1000	790	478	123	399	1000
private hos.	256	47	14	939	1000	974	13	14	972	1000	608	13	9	978	1000	797	16	9	975	1000
all	237	278	48	675	1000	972	93	169	739	1000	567	127	65	808	1000	761	162	62	776	1000
estd. no. of hosp. cases (00)	64468	17897	3065	43507	64468	264032	24441	44509	195082	264032	153976	19554	9941	124480	153976	206718	33547	12810	160360	206718
sample hosp. cases	8553	2334	406	5813	8553	31540	2857	5559	23124	31540	19218	2594	1348	15276	19218	24973	4363	1732	18878	24973

For peer review only - http://bmjopen.bmj.com/site/about/guidelines.xhtml

Appendix A

Table (28): Number per 1000 of hospitalisation cases receiving treatment before hospitalisation and per 1000 distribution of such cases by source of treatment availed of for each type of hospital where admitted and sex

All-India Rural

type of hospital where admitted	no. per 1000 of hospi-talisation cases that received treatment before hospitali-sation	average duration of treat-ment before hospita-lisation (in 0.0 days)	hosp. cases receiving treatment before hospitalisation from						hosp. cases treated before hospitalisation	
			public hospital (incl. PHC/ sub-centres/ CHC)	public dispen-sary (in-clude CGHS/ ESI	private hospital	private doctor	n.r.	total	estd. no. (00)	sample
(1)	(2)	(3)	(4)	(5)	(6)	(7)	(8)	(9)	(10)	(11)
**males**										
public hosp.	515	101.5	488	24	114	373	2	1000	20730	2488
public disp.	478	42.5	184	217	296	304	0	1000	701	95
private hosp.	530	87.5	121	14	324	541	0	1000	30495	3157
all	523	92.4	268	21	240	471	1	1000	51968	5749
**females**										
public hosp.	498	86	453	42	113	388	3	1000	17219	2145
public disp.	509	100.8	154	275	74	498	0	1000	744	72
private hosp.	609	106.8	118	17	329	535	0	1000	31086	3142
all	563	99.3	236	30	249	483	1	1000	49136	5372
**persons**										
public hosp.	507	94.5	472	32	114	380	2	1000	37949	4633
public disp.	493	72.5	168	247	182	404	0	1000	1446	167
private hosp.	567	97.2	119	16	327	538	0	1000	61581	6299
all	542	95.7	253	25	244	477	1	1000	101103	11121

For peer review only - http://bmjopen.bmj.com/site/about/guidelines.xhtml

Appendix A

Table (28): Number per 1000 of hospitalisation cases receiving treatment before hospitalisation and per 1000 distribution of such cases by source of treatment availed of for each type of hospital where admitted and sex

All-India | Urban

type of hospital where admitted	no. per 1000 of hospi-talisation cases that received treatment before hospitali-sation	average duration of treat-ment before hospita-lisation (in 0.0 days)	hosp. cases receiving treatment before hospitalisation from						hosp. cases treated before hospitalisation	
			public hospital (incl. PHC/ sub-centres/ CHC)	public dispen-sary (in-clude CGHS/ ESI	private hospital	private doctor	n.r.	total	estd. no. (00)	sample
(1)	(2)	(3)	(4)	(5)	(6)	(7)	(8)	(9)	(10)	(11)
males										
public hosp.	524	104.9	450	57	131	361	1	1000	8312	1296
public disp.	492	108.6	67	514	137	282	0	1000	694	93
private hosp.	579	95.6	85	15	339	559	1	1000	15898	1934
all	557	99.0	206	43	264	485	1	1000	24905	3324
females										
public hosp.	566	99.2	506	25	93	375	0	1000	8015	1247
public disp.	571	79.3	145	411	195	249	0	1000	642	88
private hosp.	594	109.6	65	10	354	571	1	1000	14930	1979
all	584	105.2	217	26	261	496	0	1000	23593	3315
persons										
public hosp.	544	102.1	478	42	113	368	0	1000	16327	2543
public disp.	527	94.5	105	465	165	266	0	1000	1336	181
private hosp.	586	102.3	75	13	346	565	1	1000	30828	3913
all	569	102.0	211	35	263	490	1	1000	48498	6639

For peer review only - http://bmjopen.bmj.com/site/about/guidelines.xhtml

Appendix A

Table (28): Number per 1000 of hospitalisation cases receiving treatment before hospitalisation and per 1000 distribution of such cases by source of treatment availed of for each type of hospital where admitted and sex

All-India Rural + Urban

type of hospital where admitted	no. per 1000 of hospi-talisation cases that received treatment before hospitali-sation	average duration of treat-ment before hospita-lisation (in 0.0 days)	hosp. cases receiving treatment before hospitalisation from						hosp. cases treated before hospitalisation	
			public hospital (incl. PHC/ sub-centres/ CHC)	public dispen-sary (in-clude CGHS/ ESI	private hospital	private doctor	n.r.	total	estd. no. (00)	sample
(1)	(2)	(3)	(4)	(5)	(6)	(7)	(8)	(9)	(10)	(11)
**males**										
public hosp.	518	102.5	477	33	119	369	2	1000	29043	3784
public disp.	485	75.3	126	365	217	293	0	1000	1395	188
private hosp.	546	90.2	109	14	329	547	1	1000	46393	5091
all	534	94.6	248	28	248	475	1	1000	76872	9073
**females**										
public hosp.	518	90.2	470	37	107	384	2	1000	25233	3392
public disp.	536	90.9	150	338	130	382	0	1000	1387	160
private hosp.	604	107.7	100	15	337	547	0	1000	46016	5121
all	570	101.2	230	29	253	487	1	1000	72729	8687
**persons**										
public hosp.	518	96.8	474	35	113	376	2	1000	54276	7176
public disp.	509	83.1	138	351	173	338	0	1000	2782	348
private hosp.	573	99	105	15	333	547	1	1000	92409	10212
all	551	97.8	239	28	250	481	1	1000	149601	17760

For peer review only - http://bmjopen.bmj.com/site/about/guidelines.xhtml

Appendix A

Table (29): Number per 1000 cases of hospitalisation that received treatment after discharge from hospital and per 1000 distribution of such cases by source of treatment availed of for each type of hospital where admitted and sex

All-India Rural

type of hospital from where discharged	no. per 1000 of hospitalisation cases that received treatment after hospitalisation	average duration of treatment after hospitalisation (in 0.0 days)	source of post-discharge treatment for cases which received treatment after hospitalisation						cases treated after hospitalisation	
			public hospital (incl. PHC/ sub-centres/ CHC)	public dispensary (include CGHS/ ESI	private hospital	private doctor	n.r.	total	estd. no. (00)	sample
(1)	(2)	(3)	(4)	(5)	(6)	(7)	(8)	(9)	(10)	(11)
males										
public hosp.	698	47.9	830	11	44	112	3	1000	28079	3447
public disp.	740	48.3	142	597	155	106	0	1000	1086	135
private hosp.	792	45	37	4	687	271	0	1000	45544	4453
all	753	46.1	336	15	437	209	1	1000	74750	8045
females										
public hosp.	697	40.7	822	13	42	122	1	1000	24096	2868
public disp.	687	33.8	100	629	101	171	0	1000	1005	99
private hosp.	779	49.9	36	5	650	308	1	1000	39759	3887
all	744	46.2	329	17	416	237	1	1000	64919	6867
persons										
public hosp.	698	44.6	826	12	43	117	2	1000	52176	6315
public disp.	713	41.3	121	612	129	137	0	1000	2091	234
private hosp.	786	47.2	37	5	670	289	0	1000	85303	8340
all	749	46.1	333	16	427	222	1	1000	139670	14912

For peer review only - http://bmjopen.bmj.com/site/about/guidelines.xhtml

Appendix A

Table (29): Number per 1000 cases of hospitalisation that received treatment after discharge from hospital and per 1000 distribution of such cases by source of treatment availed of for each type of hospital where admitted and sex

All-India Urban

type of hospital from where discharged	no. per 1000 of hospitali-sation cases that received treatment after hospitali-sation	average duration of treat-ment after hospita-lisation (in 0.0 days)	source of post-discharge treatment for cases which received treatment after hospitalisation						cases treated after hospitalisation	
			public hospital (incl. PHC/ sub-centres/ CHC)	public dispen-sary (in-clude CGHS/ ESI	private hospital	private doctor	n.r.	total	estd. no. (00)	sample
(1)	(2)	(3)	(4)	(5)	(6)	(7)	(8)	(9)	(10)	(11)
					males					
public hosp.	729	49.1	793	37	51	118	2	1000	11573	1828
public disp.	835	34.7	33	811	80	77	0	1000	1178	144
private hosp.	807	47.8	26	5	708	260	1	1000	22165	2783
all	780	47.8	280	43	469	207	1	1000	34921	4757
					females					
public hosp.	739	45.5	829	11	51	108	1	1000	10460	1584
public disp.	862	39.1	114	722	127	33	3	1000	969	136
private hosp.	805	44.1	20	3	689	287	1	1000	20234	2570
all	783	44.4	290	28	461	220	1	1000	31666	4292
					persons					
public hosp.	734	47.4	810	25	51	113	2	1000	22033	3412
public disp.	847	36.7	69	771	101	57	1	1000	2148	280
private hosp.	806	46	23	4	699	273	1	1000	42399	5353
all	782	46.2	285	36	465	213	1	1000	66587	9049

For peer review only

For peer review only - http://bmjopen.bmj.com/site/about/guidelines.xhtml

Table (29): Number per 1000 cases of hospitalisation that received treatment after discharge from hospital and per 1000 distribution of such cases by source of treatment availed of for each type of hospital where admitted and sex

All-India | Rural + Urban

type of hospital from where discharged	no. per 1000 of hospitalisation cases that received treatment after hospitalisation	average duration of treatment after hospitalisation (in 0.0 days)	source of post-discharge treatment for cases which received treatment after hospitalisation						cases treated after hospitalisation	
			public hospital (incl. PHC/ sub-centres/ CHC)	public dispensary (include CGHS/ ESI	private hospital	private doctor	n.r.	total	estd. no. (00)	sample
(1)	(2)	(3)	(4)	(5)	(6)	(7)	(8)	(9)	(10)	(11)
males										
public hosp.	707	48.3	819	19	46	114	3	1000	39653	5275
public disp.	787	41.2	85	708	116	91	0	1000	2264	279
private hosp.	797	45.9	33	5	694	268	1	1000	67709	7236
all	761	46.6	318	24	448	208	1	1000	109671	12802
females										
public hosp.	709	42.2	824	12	45	118	1	1000	34556	4452
public disp.	763	36.4	107	675	114	103	2	1000	1974	235
private hosp.	787	47.9	31	4	663	301	1	1000	59994	6457
all	756	45.6	316	21	431	232	1	1000	96586	11159
persons										
public hosp.	708	45.4	821	16	45	116	2	1000	74209	9727
public disp.	775	39	95	693	115	97	1	1000	4239	514
private hosp.	792	46.8	32	5	680	283	1	1000	127702	13693
all	759	46.2	317	23	440	219	1	1000	206257	23961

For peer review only - http://bmjopen.bmj.com/site/about/guidelines.xhtml

Appendix A

Table (30): Average total medical expenditure (Rs.) for treatment per hospitalisation case during the stay at hospital (as inpatient) for last 365 days by type of hospital (Govt./Pvt), mpce class and sex

All-India Rural

mpce class	average total medical expenditure for treatment (Rs.) per case									no. of cases of hospitalisation	
	males			females			persons				
	type of hospital			type of hospital			type of hospital				
	govern-ment hospital	private hospital	all hospitals (incl. n.r. cases)	govern-ment hospital	private hospital	all hospitals (incl. n.r. cases)	govern-ment hospital	private hospital	all hospitals (incl. n.r. cases)	estd. (00)	sample
(1)	(2)	(3)	(4)	(5)	(6)	(7)	(8)	(9)	(10)	(11)	(12)
0 – 225	2206	5247	3428	2862	5689	3794	2530	5431	3598	5469	702
225 – 255	2584	6236	4107	3701	3883	3786	3173	4886	3931	4577	553
255 – 300	2294	5213	3648	1841	5942	3731	2087	5543	3686	10188	1348
300 – 340	3410	6532	5010	2384	4788	3568	2950	5777	4373	11216	1367
340 – 380	2705	5153	4022	2464	5365	3857	2586	5245	3945	12839	1512
380 – 420	2132	6992	4464	2043	6776	4432	2094	6895	4450	16686	1806
420 – 470	3645	6498	5164	1939	5601	4165	2884	6028	4674	16252	1876
470 – 525	3389	6676	5156	2580	6878	5143	3017	6781	5149	19670	2216
525 – 615	3559	7648	6054	3446	5797	4895	3509	6811	5532	21763	2339
615 – 775	4471	7300	6381	3539	7358	6118	4049	7327	6262	26486	2641
775 – 950	4621	12009	9657	3782	7623	6341	4192	9843	8001	13464	1377
950 +	7857	9893	9427	4724	11777	10062	6374	10749	9718	20582	1785
all classes	3550	7640	5946	2874	7145	5406	3238	7408	5695	179191	19522
estd. no. of hosp. cases (00)	39575	56057	95674	33907	49513	83517	73482	105570	179191	X	X
sample no. of hosp. cases	4944	5506	10461	4152	4891	9061	9096	10397	19522	X	X

For peer review only - http://bmjopen.bmj.com/site/about/guidelines.xhtml

Appendix A

Table (30): Average total medical expenditure (Rs.) for treatment per hospitalisation case during the stay at hospital (as inpatient) for last 365 days by type of hospital (Govt./Pvt), mpce class and sex

All-India | Urban

mpce class	average total medical expenditure for treatment (Rs.) per case									no. of cases of hospitalisation	
	males			females			persons				
	type of hospital			type of hospital			type of hospital				
	govern-ment hospital	private hospital	all hospitals (incl. n.r. cases)	govern-ment hospital	private hospital	all hospitals (incl. n.r. cases)	govern-ment hospital	private hospital	all hospitals (incl. n.r. cases)	estd. (00)	sample
(1)	(2)	(3)	(4)	(5)	(6)	(7)	(8)	(9)	(10)	(11)	(12)
0 –300	1961	10862	5637	1395	7880	4412	1746	9576	5144	2032	287
300 – 350	1609	2978	2136	1673	3707	2514	1640	3357	2325	1455	204
350 – 425	3661	4662	4086	1415	5050	3008	2594	4852	3567	4188	597
425 – 500	2887	8014	5365	2477	8554	5249	2690	8258	5311	7793	1078
500 – 575	2806	6557	4847	2022	5618	3487	2365	6158	4171	3952	550
575 – 665	3030	6435	5159	2940	4509	3930	2984	5442	4528	7719	975
665 – 775	3545	12426	9452	2472	8397	5846	3011	10823	7883	9262	1291
775 – 915	5653	8040	7177	2887	8108	6282	4304	8074	6733	8775	1275
915 – 1120	4896	10712	8846	5567	9622	8405	5226	10149	8622	9635	1461
1120 – 1500	6476	15392	13244	3912	11770	10058	5402	13758	11830	11545	1668
1500 – 1925	4214	18401	16275	12960	14431	14236	8231	16433	15275	3849	536
1925 +	9757	24574	22535	21457	21469	21468	15132	22918	21976	6800	935
all classes	4135	12448	9535	3600	10580	8112	3877	11553	8851	77005	10857
estd. no. of hosp. cases (00)	14020	25983	40009	13074	23906	36995	27094	49889	77005	X	X
sample no. of hosp. cases	2342	3296	5641	2081	3131	5216	4423	6427	10857	X	X

For peer review only - http://bmjopen.bmj.com/site/about/guidelines.xhtml

Appendix A

Table (30): Average total medical expenditure (Rs.) for treatment per hospitalisation case during the stay at hospital (as inpatient) for last 365 days by type of hospital (Govt./Pvt), mpce class and sex

All-India Rural + Urban

mpce class	average total medical expenditure for treatment (Rs.) per case									no. of cases of hospitalisation	
	males			females			persons				
	type of hospital			type of hospital			type of hospital				
	govern-ment hospital	private hospital	all hospitals (incl. n.r. cases)	govern-ment hospital	private hospital	all hospitals (incl. n.r. cases)	govern-ment hospital	private hospital	all hospitals (incl. n.r. cases)	estd. (00)	sample
(1)	(2)	(3)	(4)	(5)	(6)	(7)	(8)	(9)	(10)	(11)	(12)
0 – 235	2135	6926	4076	2563	6373	3944	2334	6694	4017	7501	989
235 – 265	2320	5442	3596	3210	3847	3499	2781	4546	3544	6031	757
265 – 320	2699	5067	3771	1708	5681	3512	2241	5352	3651	14376	1945
320 – 365	3194	7101	5151	2426	6306	4282	2839	6751	4757	19009	2445
365 – 410	2727	5470	4206	2342	5421	3765	2531	5449	3999	16791	2062
410 – 460	2333	6801	4662	2301	5858	4255	2319	6354	4475	24405	2781
460 – 520	3614	9111	6825	2129	6499	4730	2926	7870	5839	25515	3167
520 – 605	3974	7148	5778	2666	7282	5495	3362	7216	5638	28445	3491
605 – 730	3888	8586	6846	4044	7184	6064	3960	7922	6480	31397	3800
730 – 980	4968	9994	8491	3622	8816	7296	4368	9461	7952	38031	4309
980 – 1285	4572	13711	11148	4701	9446	8072	4637	11602	9618	17313	1913
1285 +	8139	13491	12375	7091	14768	13190	7645	14099	12762	27382	2720
all classes	3703	9163	7004	3076	8264	6237	3410	8738	6643	256196	30379
estd. no. of hosp. cases (00)	53595	82039	135684	46981	73419	120513	100576	155459	256196	X	X
sample no. of hosp. cases	7286	8802	16102	6233	8022	14277	13519	16824	30379	X	X

For peer review only - http://bmjopen.bmj.com/site/about/guidelines.xhtml

Appendix A

Table (31): Average expenditure (Rs.) per hospitalisation case on account of hospitalisation for each mpce class and sex

All-India		Males				Rural	
mpce class	average total expenditure (Rs.) on account of hospitalisation			loss of household income (Rs.) due to hospitalisation	estd. total expenditure (000) for hospitalisation	hospitalisation cases	
	medical expenditure on treatment during stay at hospital	other expenses incurred for hospitalisation	total expenditure for hospitalisation			estd. no. (00)	sample
(1)	(2)	(3)	(4)	(5)	(6)	(7)	(8)
0 – 225	3428	364	3792	468	1109714	2926	393
225 – 255	4107	446	4552	741	942940	2071	294
255 – 300	3648	376	4025	586	2236714	5557	740
300 – 340	5010	505	5515	812	3452510	6260	753
340 – 380	4022	512	4534	722	3130709	6905	817
380 – 420	4464	506	4971	624	4677799	9411	970
420 – 470	5164	461	5625	775	4657609	8280	986
470 – 525	5156	510	5665	764	5637824	9951	1143
525 – 615	6054	519	6574	757	7861199	11959	1287
615 – 775	6381	581	6963	898	10074240	14469	1417
775 – 950	9657	795	10451	821	7043219	6739	707
950 +	9427	737	10164	809	11327808	11145	954
all classes	5946	550	6496	760	62152284	95674	10461
estd. total exp. (000) for hospitalisation	56889402	5262882	62152284	7271251	X	X	X

Table (31): Average expenditure (Rs.) per hospitalisation case on account of hospitalisation for each mpce class and sex

All-India		Females				Rural	
mpce class	average total expenditure (Rs.) on account of hospitalisation			loss of household income (Rs.) due to hospitalisation	estd. total expenditure (000) for hospitalisation	hospitalisation cases	
	medical expenditure on treatment during stay at hospital	other expenses incurred for hospitalisation	total expenditure for hospitalisation			estd. no. (00)	sample
(1)	(2)	(3)	(4)	(5)	(6)	(7)	(8)
0 – 225	3794	393	4186	508	1064259	2542	309
225 – 255	3786	548	4334	301	1085824	2505	259
255 – 300	3731	305	4036	401	1868653	4630	608
300 – 340	3568	347	3916	382	1940571	4956	614
340 – 380	3857	453	4310	347	2557178	5934	695
380 – 420	4432	447	4879	424	3549379	7275	836
420 – 470	4165	431	4597	547	3664487	7972	890
470 – 525	5143	506	5649	625	5490017	9719	1073
525 – 615	4895	480	5375	467	5269480	9804	1052
615 – 775	6118	696	6814	498	8189161	12018	1224
775 – 950	6341	523	6865	587	4616416	6725	670
950 +	10062	638	10700	559	10097137	9437	831
all classes	5406	508	5914	493	49392564	83517	9061
estd. total exp. (000) for hospitalisation	45152014	4240549	49392564	4116921	X	X	X

For peer review only - http://bmjopen.bmj.com/site/about/guidelines.xhtml

Appendix A

Table (31): Average expenditure (Rs.) per hospitalisation case on account of hospitalisation for each mpce class and sex

All-India — Persons — Rural

mpce class	average total expenditure (Rs.) on account of hospitalisation			loss of household income (Rs.) due to hospi-talisation	estd. total expen-diture (000) for hospita-lisation	hospitalisation cases	
	medical expenditure on treatment during stay at hospital	other expenses incurred for hospitalisation	total expenditure for hospitalisation			estd. no. (00)	sam-ple
(1)	(2)	(3)	(4)	(5)	(6)	(7)	(8)
0 – 225	3598	377	3975	486	2173973	5469	702
225 – 255	3931	502	4433	500	2028763	4577	553
255 – 300	3686	344	4030	502	4105367	10188	1348
300 – 340	4373	436	4808	622	5393081	11216	1367
340 – 380	3945	485	4430	549	5687887	12839	1512
380 – 420	4450	480	4930	537	8227179	16686	1806
420 – 470	4674	446	5121	663	8322096	16252	1876
470 – 525	5149	508	5657	696	11127840	19670	2216
525 – 615	5532	502	6034	626	13130679	21763	2339
615 – 775	6262	633	6895	716	18263402	26486	2641
775 – 950	8001	659	8660	704	11659636	13464	1377
950 +	9718	692	10410	695	21424945	20582	1785
all classes	5695	530	6225	636	111544848	179191	19522
estd. total exp. (000) for hospita-lisation	102041416	9503432	111544848	11388172	X	X	X

Table (31): Average expenditure (Rs.) per hospitalisation case on account of hospitalisation for each mpce class and sex

All-India — Males — Urban

mpce class	average total expenditure (Rs.) on account of hospitalisation			loss of household income (Rs.) due to hospi-talisation	estd. total expen-diture (000) for hospita-lisation	hospitalisation cases	
	medical expenditure on treatment during stay at hospital	other expenses incurred for hospitalisation	total expenditure for hospitalisation			estd. no. (00)	sam-ple
(1)	(2)	(3)	(4)	(5)	(6)	(7)	(8)
0 –300	5637	214	5850	687	710826	1215	170
300 – 350	2136	201	2337	451	169335	725	110
350 – 425	4086	366	4451	1413	967575	2174	314
425 – 500	5365	341	5705	740	2361804	4140	563
500 – 575	4847	358	5205	594	1034723	1988	283
575 – 665	5159	317	5476	807	2057950	3758	497
665 – 775	9452	531	9983	891	5223424	5232	717
775 – 915	7177	320	7497	772	3316490	4424	644
915 – 1120	8846	426	9273	1079	4391785	4736	722
1120 – 1500	13244	1010	14254	1879	9154925	6423	910
1500 – 1925	16275	723	16999	895	3333078	1961	252
1925 +	22535	984	23519	1369	7606889	3234	459
all classes	9535	545	10080	1073	40328805	40009	5641
estd. total exp. (000) for hospita-lisation	38147506	2181299	40328805	4294445	X	X	X

For peer review only - http://bmjopen.bmj.com/site/about/guidelines.xhtml

Appendix A

Table (31): Average expenditure (Rs.) per hospitalisation case on account of hospitalisation for each mpce class and sex

All-India Females Urban

mpce class	average total expenditure (Rs.) on account of hospitalisation			loss of household income (Rs.) due to hospi-talisation	estd. total expen-diture (000) for hospita-lisation	hospitalisation cases	
	medical expenditure on treatment during stay at hospital	other expenses incurred for hospitalisation	total expenditure for hospitalisation			estd. no. (00)	sam-ple
(1)	(2)	(3)	(4)	(5)	(6)	(7)	(8)
0 –300	4412	314	4726	354	386196	817	117
300 – 350	2514	115	2629	249	191921	730	94
350 – 425	3008	239	3247	281	653994	2014	283
425 – 500	5249	375	5625	398	2054567	3653	515
500 – 575	3487	308	3794	441	745270	1964	267
575 – 665	3930	235	4164	247	1649481	3961	478
665 – 775	5846	267	6113	425	2463704	4030	574
775 – 915	6282	338	6620	349	2880076	4351	631
915 – 1120	8405	546	8951	505	4384493	4899	739
1120 – 1500	10058	989	11047	352	5658412	5122	758
1500 – 1925	14236	465	14701	485	2776306	1888	284
1925 +	21468	851	22318	478	7958234	3566	476
all classes	8112	485	8596	391	31802653	36995	5216
estd. total exp. (000) for hospita-lisation	30010203	1792450	31802653	1446166	X	X	X

Table (31): Average expenditure (Rs.) per hospitalisation case on account of hospitalisation for each mpce class and sex

All-India Persons Urban

mpce class	average total expenditure (Rs.) on account of hospitalisation			loss of household income (Rs.) due to hospi-talisation	estd. total expen-diture (000) for hospita-lisation	hospitalisation cases	
	medical expenditure on treatment during stay at hospital	other expenses incurred for hospitalisation	total expenditure for hospitalisation			estd. no. (00)	sam-ple
(1)	(2)	(3)	(4)	(5)	(6)	(7)	(8)
0 –300	5144	254	5398	553	1097022	2032	287
300 – 350	2325	158	2483	350	361256	1455	204
350 – 425	3567	305	3872	868	1621569	4188	597
425 – 500	5311	357	5667	580	4416370	7793	1078
500 – 575	4171	333	4504	518	1779993	3952	550
575 – 665	4528	275	4803	520	3707432	7719	975
665 – 775	7883	416	8299	688	7687128	9262	1291
775 – 915	6733	329	7062	562	6196566	8775	1275
915 – 1120	8622	487	9109	787	8776277	9635	1461
1120 – 1500	11830	1001	12831	1201	14813337	11545	1668
1500 – 1925	15275	597	15872	694	6109384	3849	536
1925 +	21976	914	22890	902	15565123	6800	935
all classes	8851	516	9367	745	72131458	77005	10857
estd. total exp. (000) for hospita-lisation	68157709	3973749	72131458	5740611	X	X	X

For peer review only - http://bmjopen.bmj.com/site/about/guidelines.xhtml

Appendix A

Table (31): Average expenditure (Rs.) per hospitalisation case on account of hospitalisation for each mpce class and sex

All-India	Males					Rural + Urban	
mpce class	average total expenditure (Rs.) on account of hospitalisation			loss of household income (Rs.) due to hospitalisation	estd. total expenditure (000) for hospitalisation	hospitalisation cases	
	medical expenditure on treatment during stay at hospital	other expenses incurred for hospitalisation	total expenditure for hospitalisation			estd. no. (00)	sample
(1)	(2)	(3)	(4)	(5)	(6)	(7)	(8)
0 – 235	4076	320	4396	532	1820540	4141	563
235 – 265	3596	382	3978	666	1112274	2796	404
265 – 320	3771	373	4145	819	3204289	7731	1054
320 – 365	5151	440	5591	783	5814314	10400	1316
365 – 410	4206	478	4684	693	4165432	8893	1100
410 – 460	4662	452	5115	676	6735750	13169	1467
460 – 520	6825	488	7313	820	9881033	13512	1703
520 – 605	5778	451	6229	767	8954314	14375	1787
605 – 730	6846	493	7339	848	12252984	16695	2009
730 – 980	8491	713	9204	1199	19229166	20891	2327
980 – 1285	11148	779	11927	838	10376297	8700	959
1285 +	12375	793	13168	935	18934697	14379	1413
all classes	7004	549	7553	852	102481089	135684	16102
estd. total exp. (000) for hospitalisation	95036908	7444181	102481089	11565696	X	X	X

Table (31): Average expenditure (Rs.) per hospitalisation case on account of hospitalisation for each mpce class and sex

All-India	Females					Rural + Urban	
mpce class	average total expenditure (Rs.) on account of hospitalisation			loss of household income (Rs.) due to hospitalisation	estd. total expenditure (000) for hospitalisation	hospitalisation cases	
	medical expenditure on treatment during stay at hospital	other expenses incurred for hospitalisation	total expenditure for hospitalisation			estd. no. (00)	sample
(1)	(2)	(3)	(4)	(5)	(6)	(7)	(8)
0 – 235	3944	374	4318	471	1450455	3359	426
235 – 265	3499	450	3949	289	1277745	3235	353
265 – 320	3512	285	3797	364	2522647	6645	891
320 – 365	4282	359	4641	389	3995138	8609	1129
365 – 410	3765	417	4181	371	3302449	7898	962
410 – 460	4255	372	4627	361	5198861	11236	1314
460 – 520	4730	376	5106	506	6128191	12002	1464
520 – 605	5495	454	5949	540	8370093	14069	1704
605 – 730	6064	502	6566	480	9653973	14703	1791
730 – 980	7296	783	8079	455	13847573	17140	1982
980 – 1285	8072	510	8583	565	7392722	8614	954
1285 +	13190	696	13886	537	18055371	13003	1307
all classes	6237	501	6737	462	81195217	120513	14277
estd. total exp. (000) for hospitalisation	75162217	6033000	81195217	5563087	X	X	X

For peer review only - http://bmjopen.bmj.com/site/about/guidelines.xhtml

Appendix A

Table (31): Average expenditure (Rs.) per hospitalisation case on account of hospitalisation for each mpce class and sex

All-India	Persons						Rural + Urban
mpce class	average total expenditure (Rs.) on account of hospitalisation			loss of household income (Rs.) due to hospi-talisation	estd. total expen-diture (000) for hospita-lisation	hospitalisation cases	
	medical expenditure on treatment during stay at hospital	other expenses incurred for hospitalisation	total expenditure for hospitalisation			estd. no. (00)	sam-ple
(1)	(2)	(3)	(4)	(5)	(6)	(7)	(8)
0 – 235	4017	344	4361	504	3270995	7501	989
235 – 265	3544	419	3963	464	2390019	6031	757
265 – 320	3651	332	3984	609	5726936	14376	1945
320 – 365	4757	403	5161	605	9809452	19009	2445
365 – 410	3999	449	4448	542	7467880	16791	2062
410 – 460	4475	415	4890	531	11934611	24405	2781
460 – 520	5839	435	6275	672	16009224	25515	3167
520 – 605	5638	453	6091	654	17324407	28445	3491
605 – 730	6480	497	6977	676	21906956	31397	3800
730 – 980	7952	745	8697	864	33076739	38031	4309
980 – 1285	9618	645	10263	702	17769020	17313	1913
1285 +	12762	747	13509	746	36990068	27382	2720
all classes	6643	526	7169	669	183676306	256196	30379
estd. total exp. (000) for hospita-lisation	170199125	13477181	183676306	17128783	X	X	X

For peer review only - http://bmjopen.bmj.com/site/about/guidelines.xhtml

For peer review only

Appendix A

Table (32): Average total expenditure on account of hospitalisation per household and per 1000 distribution of total household expenditure on account of hospitalisation by source of finance for each mpce class

All-India Rural

mpce class	average total expen-diture on account of hosp. per hhs. (Rs.)	distribution of expenses of hospitalisation by source of finance					estd. total expen-diture for hosp. (000)	households with hospitalisation cases during last 365 days	
		house-hold income/ savings	borro-wings	contri-bution from friends and relatives	other sources (incl. sale of ornaments and other physical assets, draught animals, etc.)	total		estd no. of hhs.(00)	sample hhs.
(1)	(2)	(3)	(4)	(5)	(6)	(7)	(8)	(9)	(10)
0 – 225	3975	332	435	152	81	1000	2173973	5028	655
225 – 255	4433	265	556	109	69	1000	2028763	4267	512
255 – 300	4030	314	488	131	67	1000	4105367	9058	1248
300 – 340	4808	349	462	142	47	1000	5393081	9697	1250
340 – 380	4430	379	461	121	40	1000	5687887	11099	1344
380 – 420	4930	347	402	154	97	1000	8227179	13235	1605
420 – 470	5121	391	451	104	54	1000	8322096	13929	1733
470 – 525	5657	399	435	102	64	1000	11127840	16502	1988
525 – 615	6034	420	411	119	50	1000	13130679	17455	2110
615 – 775	6895	418	406	132	44	1000	18263402	21154	2344
775 – 950	8660	419	411	106	64	1000	11659636	11120	1228
950 +	10410	485	331	143	41	1000	21424945	15660	1486
all	6225	409	411	126	55	1000	111544848	148206	17503
estd. expn. (000)	X	47268294	47485229	14579049	6324977	115657549	X	X	X
estd. no. of hhs. with hosp. cases (00)	X	125176	70846	28964	10196	148206	X	X	X
sample hhs. with hosp. cases	X	15197	7934	3423	1279	17503	X	X	X

For peer review only - http://bmjopen.bmj.com/site/about/guidelines.xhtml

Appendix A

Table (32): Average total expenditure on account of hospitalisation per household and per 1000 distribution of total household expenditure on account of hospitalisation by source of finance for each mpce class

All-India Urban

mpce class	average total expen-diture on account of hosp. per hhs. (Rs.)	distribution of expenses of hospitalisation by source of finance					estd. total expen-diture for hosp. (000)	households with hospitalisation cases during last 365 days	
		house-hold income/ savings	borro-wings	contri-bution from friends and relatives	other sources (incl. sale of ornaments and other physical assets, draught animals, etc.)	total		estd no. of hhs.(00)	sample hhs.
(1)	(2)	(3)	(4)	(5)	(6)	(7)	(8)	(9)	(10)
0 –300	5398	541	308	123	28	1000	1097022	1628	246
300 – 350	2483	338	401	193	69	1000	361256	1277	181
350 – 425	3872	322	334	171	172	1000	1621569	3537	532
425 – 500	5667	345	486	110	59	1000	4416370	6867	992
500 – 575	4504	472	359	148	21	1000	1779993	3234	480
575 – 665	4803	419	407	106	68	1000	3707432	6004	867
665 – 775	8299	573	295	108	24	1000	7687128	7961	1168
775 – 915	7062	526	283	124	67	1000	6196566	7735	1192
915 – 1120	9109	625	191	142	42	1000	8776277	9165	1415
1120 – 1500	12831	585	203	78	134	1000	14813337	10920	1632
1500 – 1925	15872	707	116	159	18	1000	6109384	3603	502
1925 +	22890	665	107	119	110	1000	15565123	6232	907
all	9367	578	227	117	78	1000	72131458	68163	10114
estd. expn. (000)	X	44941929	17627649	9101180	6057128	77727887	X	X	X
estd. no. of hhs. with hosp. cases (00)	X	60627	20126	10584	2518	68163	X	X	X
sample hhs. with hosp. cases	X	9148	2782	1582	377	10114	X	X	X

For peer review only - http://bmjopen.bmj.com/site/about/guidelines.xhtml

Table (32): Average total expenditure on account of hospitalisation per household and per 1000 distribution of total household expenditure on account of hospitalisation by source of finance for each mpce class

All-India — Rural + Urban

mpce class	average total expen-diture on account of hosp. per hhs. (Rs.)	distribution of expenses of hospitalisation by source of finance					estd. total expen-diture for hosp. (000)	households with hospitalisation cases during last 365 days	
		house-hold income/ savings	borro-wings	contri-bution from friends and relatives	other sources (incl. sale of ornaments and other physical assets, draught animals, etc.)	total		estd no. of hhs.(00)	sample hhs.
(1)	(2)	(3)	(4)	(5)	(6)	(7)	(8)	(9)	(10)
0 – 235	4361	400	394	142	64	1000	3270995	6656	901
235 – 265	3963	277	532	122	69	1000	2390019	5544	693
265 – 320	3984	316	445	142	97	1000	5726936	12595	1780
320 – 365	5161	347	473	127	52	1000	9809452	16564	2242
365 – 410	4448	400	437	127	35	1000	7467880	14334	1824
410 – 460	4890	370	404	138	88	1000	11934611	19239	2472
460 – 520	6275	479	375	106	40	1000	16009224	21889	2901
520 – 605	6091	446	378	110	65	1000	17324407	24237	3180
605 – 730	6977	509	316	129	46	1000	21906956	26620	3525
730 – 980	8697	495	313	107	85	1000	33076739	32074	3976
980 – 1285	10263	518	310	124	48	1000	17769020	14723	1730
1285 +	13509	562	235	133	70	1000	36990068	21892	2393
all	7169	477	337	122	64	1000	183676306	216369	27617
estd. expn. (000)	X	92210223	65112879	23680229	12382106	193385436	X	X	X
estd. no. of hhs. with hosp. cases (00)	X	185803	90972	39548	12714	216369	X	X	X
sample hhs. with hosp. cases	X	24345	10716	5005	1656	27617	X	X	X

For peer review only - http://bmjopen.bmj.com/site/about/guidelines.xhtml

Appendix A

Table (33): Average medical expenditure (Rs.) for treatment under different heads of treatment during stay at hospitals as inpatient during last 365 days per hospitalisation case receiving medical treatment for each type of hospital, mpce class of household

All-India Rural type of hospital: public hospital

mpce class	average medical expenditure (Rs.) for treatment under different heads for treatment during stay at hospitals: services: doctor's/ surgeons fee: hospital staff	other specia-list	diag-nostic test	bed char-ges	atten-dant char-ges	physio-therapy	other servi-ces	goods: medicines: from hos-pital	from out-side	perso-nal medi-cal applia-nces	food etc.	blood, oxy-gen, etc.	total	estd. expn. (000)	hosp. cases receiving med. treatment on payment (00): estd. (00)	sample
(1)	(2)	(3)	(4)	(5)	(6)	(7)	(8)	(9)	(10)	(11)	(12)	(13)	(14)	(15)	(16)	(17)
0 – 225	20	2	29	4	10	0	2	16	713	10	110	97	1013	107373	1060	129
225 – 255	15	14	94	8	3	0	12	26	959	3	96	40	1269	133875	1055	111
255 – 300	19	13	79	6	3	0	15	20	881	7	109	48	1200	253536	2113	258
300 – 340	7	25	79	2	1	0	10	30	837	2	146	16	1156	262785	2274	247
340 – 380	39	15	210	14	4	0	20	51	1012	7	185	10	1567	299683	1912	262
380 – 420	27	13	94	15	8	1	12	32	784	10	153	29	1177	365652	3106	321
420 – 470	25	47	231	59	10	0	15	37	1027	9	123	19	1601	378700	2365	286
470 – 525	30	50	250	37	17	1	21	54	1003	27	143	62	1695	533669	3148	384
525 – 615	7	30	171	30	21	6	29	29	775	58	136	8	1300	460969	3546	391
615 – 775	85	16	160	24	15	0	10	53	831	22	117	39	1372	429620	3131	387
775 – 950	61	61	386	48	52	14	79	71	1436	13	146	66	2434	420046	1726	194
950 +	94	10	279	80	7	2	32	62	1230	5	151	67	2019	309494	1533	178
all classes	35	26	175	28	13	2	21	41	935	18	137	37	1467	3955401	26969	3148

For peer review only - http://bmjopen.bmj.com/site/about/guidelines.xhtml

Appendix A

Table (33): Average medical expenditure (Rs.) for treatment under different heads of treatment during stay at hospitals as inpatient during last 365 days per hospitalisation case receiving medical treatment for each type of hospital, mpce class of household

All-India Rural type of hospital: public dispensary

mpce class	average medical expenditure (Rs.) for treatment under different heads for treatment during stay at hospitals													estd. expn. (000)	hosp. cases receiving med. treatment on payment (00)	
	services							goods					total		estd. (00)	sample
	doctor's/ surgeons fee		diag- nostic test	bed char- ges	atten- dant char- ges	physio- therapy	other servi- ces	medicines		perso- nal medi- cal applia- nces	food etc.	blood, oxy- gen, etc.				
	hospital staff	other specia- list						from hos- pital	from out- side							
(1)	(2)	(3)	(4)	(5)	(6)	(7)	(8)	(9)	(10)	(11)	(12)	(13)	(14)	(15)	(16)	(17)
0 – 225	0	0	0	0	0	0	0	0	228	0	299	0	527	638	12	2
225 – 255	12	80	90	0	0	0	9	0	307	0	179	0	677	2119	31	7
255 – 300	0	0	2	0	0	0	0	0	142	0	70	0	213	1811	85	8
300 – 340	46	493	56	0	0	0	0	26	4616	0	253	77	5566	13254	24	6
340 – 380	153	0	152	61	0	0	0	152	81	0	110	0	708	5100	72	4
380 – 420	0	0	53	7	8	0	0	3	1301	0	223	21	1616	13939	86	13
420 – 470	243	0	192	73	0	0	0	0	810	0	83	27	1427	20023	140	11
470 – 525	0	20	122	20	0	0	0	0	376	8	53	0	599	7234	121	10
525 – 615	278	75	794	248	0	0	0	259	1372	31	637	58	3753	28384	76	14
615 – 775	0	73	138	0	0	0	2	122	1186	0	97	0	1618	11149	69	8
775 – 950	1042	18	301	473	28	252	340	982	3640	12	194	27	7310	67575	92	13
950 +	2	343	51	51	0	0	0	103	690	0	343	0	1583	6310	40	3
all classes	193	50	190	97	4	27	38	159	1132	5	180	17	2092	177538	848	99

For peer review only - http://bmjopen.bmj.com/site/about/guidelines.xhtml

For peer review only

Appendix A

Table (33): Average medical expenditure (Rs.) for treatment under different heads of treatment during stay at hospitals as inpatient during last 365 days per hospitalisation case receiving medical treatment for each type of hospital, mpce class of household

All-India Rural type of hospital: public hospital + public dispensary

mpce class	average medical expenditure (Rs.) for treatment under different heads for treatment during stay at hospitals													estd. expn. (000)	hosp. cases receiving med. treatment on payment (00)	
	services							goods					total		estd. (00)	sample
	doctor's/ surgeons fee		diag-nostic test	bed char-ges	atten-dant char-ges	physio-therapy	other servi-ces	medicines		perso-nal medi-cal applia-nces	food etc.	blood, oxy-gen, etc.				
	hospital staff	other specia-list						from hos-pital	from out-side							
(1)	(2)	(3)	(4)	(5)	(6)	(7)	(8)	(9)	(10)	(11)	(12)	(13)	(14)	(15)	(16)	(17)
0 – 225	20	2	29	3	10	0	2	16	708	10	112	96	1008	108011	1072	131
225 – 255	14	16	94	8	2	0	12	26	940	3	98	39	1252	135995	1087	118
255 – 300	18	12	76	5	3	0	14	19	853	6	108	47	1162	255347	2198	266
300 – 340	8	30	79	2	1	0	10	30	876	2	148	17	1201	276039	2298	253
340 – 380	43	14	208	16	4	0	19	55	978	7	182	10	1536	304783	1984	266
380 – 420	26	13	93	15	8	1	11	31	798	9	154	29	1189	379591	3192	334
420 – 470	37	44	229	60	9	0	14	35	1015	8	121	19	1592	398722	2505	297
470 – 525	29	49	245	36	17	1	20	52	980	26	140	59	1655	540903	3269	394
525 – 615	13	30	184	35	21	6	28	34	788	57	146	9	1351	489352	3622	405
615 – 775	83	17	160	23	15	0	10	54	838	22	117	38	1378	440769	3200	395
775 – 950	110	59	382	70	50	26	92	118	1548	13	149	64	2682	487622	1818	207
950 +	92	18	273	80	7	2	31	64	1216	5	156	66	2008	315804	1572	181
all classes	40	27	175	30	13	3	21	44	941	18	138	36	1486	4132939	27817	3247

For peer review only - http://bmjopen.bmj.com/site/about/guidelines.xhtml

Appendix A

Table (33): Average medical expenditure (Rs.) for treatment under different heads of treatment during stay at hospitals as inpatient during last 365 days per hospitalisation case receiving medical treatment for each type of hospital, mpce class of household

All-India Rural type of hospital: private hospital

mpce class	average medical expenditure (Rs.) for treatment under different heads for treatment during stay at hospitals: services: doctor's/ surgeons fee: hospital staff	services: doctor's/ surgeons fee: other specia-list	services: diag-nostic test	services: bed char-ges	services: atten-dant char-ges	services: physio-therapy	services: other servi-ces	goods: medicines: from hos-pital	goods: medicines: from out-side	goods: perso-nal medi-cal applia-nces	goods: food etc.	goods: blood, oxy-gen, etc.	total	estd. expn. (000)	hosp. cases receiving med. treatment on payment (00): estd. (00)	hosp. cases receiving med. treatment on payment (00): sample
(1)	(2)	(3)	(4)	(5)	(6)	(7)	(8)	(9)	(10)	(11)	(12)	(13)	(14)	(15)	(16)	(17)
0 – 225	164	153	160	269	4	0	0	134	975	25	133	17	2034	92797	456	53
225 – 255	325	154	311	491	83	0	1	31	970	2	180	20	2569	167548	652	41
255 – 300	964	193	354	565	37	1	14	517	1410	24	230	18	4326	458708	1060	131
300 – 340	346	264	199	429	23	0	10	520	928	3	148	140	3010	426750	1418	153
340 – 380	524	312	369	424	34	19	52	314	1203	6	271	20	3548	556074	1567	162
380 – 420	711	366	390	425	50	40	36	301	1267	27	256	133	4001	677391	1693	198
420 – 470	831	287	400	515	48	29	31	611	1082	4	246	78	4162	978331	2351	250
470 – 525	652	389	496	625	95	34	43	476	1491	9	195	197	4702	1409496	2998	293
525 – 615	1109	167	344	670	50	1	45	440	1323	9	237	34	4428	1388452	3135	303
615 – 775	1312	397	524	829	120	17	167	711	1680	127	244	147	6276	2401439	3827	344
775 – 950	1150	163	511	579	77	48	44	261	1834	33	278	121	5100	967494	1897	171
950 +	1271	462	697	946	83	46	70	632	1913	46	265	94	6525	2116415	3244	255
all classes	927	311	449	640	70	24	60	489	1448	35	235	102	4791	11640894	24297	2354

For peer review only - http://bmjopen.bmj.com/site/about/guidelines.xhtml

For peer review only

Table (33): Average medical expenditure (Rs.) for treatment under different heads of treatment during stay at hospitals as inpatient during last 365 days per hospitalisation case receiving medical treatment for each type of hospital, mpce class of household

All-India | Rural | type of hospital: all

mpce class	average medical expenditure (Rs.) for treatment under different heads for treatment during stay at hospitals													estd. expn. (000)	hosp. cases receiving med. treatment on payment (00)	
	services							goods					total		estd. (00)	sample
	doctor's/ surgeons fee		diag-nostic test	bed char-ges	atten-dant char-ges	physio-therapy	other servi-ces	medicines		perso-nal medi-cal applia-nces	food etc.	blood, oxy-gen, etc.				
	hospital staff	other specia-list						from hos-pital	from out-side							
(1)	(2)	(3)	(4)	(5)	(6)	(7)	(8)	(9)	(10)	(11)	(12)	(13)	(14)	(15)	(16)	(17)
0 – 225	63	47	68	83	8	0	1	51	788	14	119	72	1314	200808	1528	184
225 – 255	131	68	175	189	33	0	7	28	951	3	129	32	1746	303542	1739	159
255 – 300	326	71	166	187	14	0	14	181	1034	12	148	37	2191	714055	3258	397
300 – 340	137	119	125	165	9	0	10	217	896	2	148	64	1892	702789	3715	406
340 – 380	255	145	278	196	17	8	33	169	1078	7	221	14	2424	862888	3560	430
380 – 420	263	135	196	157	23	14	20	125	961	15	190	65	2165	1058612	4890	533
420 – 470	421	162	312	280	28	14	22	314	1048	6	181	47	2836	1377592	4858	548
470 – 525	327	212	365	318	54	17	31	255	1224	18	166	125	3112	1950399	6267	687
525 – 615	521	95	258	330	34	4	36	222	1035	35	188	21	2780	1879007	6760	709
615 – 775	753	224	358	462	72	9	95	412	1297	79	186	98	4045	2842208	7026	739
775 – 950	641	112	448	330	64	38	68	191	1694	23	215	93	3917	1455116	3715	378
950 +	886	317	558	663	58	32	57	446	1685	33	229	84	5050	2432218	4816	436
all classes	454	160	303	314	39	13	39	252	1177	26	183	67	3027	1577923<br>5	52133	5606

For peer review only

For peer review only - http://bmjopen.bmj.com/site/about/guidelines.xhtml

Appendix A

Table (33): Average medical expenditure (Rs.) for treatment under different heads of treatment during stay at hospitals as inpatient during last 365 days per hospitalisation case receiving medical treatment for each type of hospital, mpce class of household

All-India Urban type of hospital: public hospital

mpce class	average medical expenditure (Rs.) for treatment under different heads for treatment during stay at hospitals													estd. expn. (000)	hosp. cases receiving med. treatment on payment (00)	
	services							goods					total		estd. (00)	sample
	doctor's/ surgeons fee		diag-nostic test	bed char-ges	atten-dant char-ges	physio-therapy	other servi-ces	medicines		perso-nal medi-cal applia-nces	food etc.	blood, oxy-gen, etc.				
	hospital staff	other specia-list						from hos-pital	from out-side							
(1)	(2)	(3)	(4)	(5)	(6)	(7)	(8)	(9)	(10)	(11)	(12)	(13)	(14)	(15)	(16)	(17)
0 –300	6	1	73	1	1	0	1	62	496	3	79	22	744	25074	337	57
300 – 350	76	1	148	0	20	0	33	38	891	27	71	179	1485	64742	436	53
350 – 425	4	0	114	4	22	0	23	17	450	10	68	25	736	63053	857	120
425 – 500	25	3	167	15	3	0	7	33	557	9	65	17	902	136710	1516	224
500 – 575	6	2	179	20	1	0	13	14	1072	2	109	7	1426	107303	753	102
575 – 665	51	7	104	21	2	7	29	39	594	14	113	51	1031	113405	1100	184
665 – 775	69	20	118	34	5	22	11	11	789	47	104	40	1270	169965	1338	216
775 – 915	38	14	97	14	13	2	25	31	720	4	138	12	1107	119423	1079	203
915 – 1120	47	23	412	70	25	0	25	105	1245	17	124	34	2128	201427	947	207
1120 – 1500	26	74	689	76	121	1	53	99	1454	144	204	14	2956	223058	755	192
1500 – 1925	268	7	572	46	86	3	82	153	1531	27	116	50	2940	53288	181	49
1925 +	219	211	348	260	43	18	37	155	1439	104	93	112	3039	84502	278	73
all classes	46	20	215	34	21	5	23	47	839	29	107	36	1422	1361951	9577	1680

For peer review only

For peer review only - http://bmjopen.bmj.com/site/about/guidelines.xhtml

Appendix A

Table (33): Average medical expenditure (Rs.) for treatment under different heads of treatment during stay at hospitals as inpatient during last 365 days per hospitalisation case receiving medical treatment for each type of hospital, mpce class of household

All-India Urban type of hospital: public dispensary

mpce class	average medical expenditure (Rs.) for treatment under different heads for treatment during stay at hospitals													estd. expn. (000)	hosp. cases receiving med. treatment on payment (00)	
	services							goods					total		estd. (00)	sample
	doctor's/ surgeons fee		diag-nostic test	bed char-ges	atten-dant char-ges	physio-therapy	other servi-ces	medicines		perso-nal medi-cal applia-nces	food etc.	blood, oxy-gen, etc.				
	hospital staff	other specia-list						from hos-pital	from out-side							
(1)	(2)	(3)	(4)	(5)	(6)	(7)	(8)	(9)	(10)	(11)	(12)	(13)	(14)	(15)	(16)	(17)
0 –300	15	0	3	0	28	0	23	0	820	0	71	0	960	2838	30	3
300 – 350	0	0	0	0	0	0	0	0	10000	0	0	0	10000	3447	3	1
350 – 425	8	0	223	0	0	0	0	35	329	0	121	0	717	1502	21	3
425 – 500	1	0	92	0	0	0	0	17	551	0	144	25	830	5405	65	7
500 – 575	355	0	1454	199	65	0	112	0	2385	0	335	0	4904	19576	40	5
575 – 665	30	0	12	0	0	0	0	0	385	0	137	0	563	1431	25	4
665 – 775	0	26	10	4	0	155	9	0	877	0	17	0	1098	2639	24	5
775 – 915	619	0	96	0	0	0	24	7	1252	0	84	48	2130	24941	117	13
915 – 1120	81	0	167	61	0	0	14	0	1054	0	107	14	1498	6259	42	8
1120 – 1500	0	357	355	714	57	0	160	0	1328	67	184	0	3221	14077	44	3
1500 – 1925	0	0	0	0	0	0	0	0	0	0	0	0	0	0	0	0
1925 +	7500	2000	3450	2725	1350	2850	2000	0	5250	1500	5000	2375	36000	24047	7	2
all classes	339	71	302	144	36	55	70	6	1224	31	208	57	2541	106163	418	54

For peer review only

For peer review only - http://bmjopen.bmj.com/site/about/guidelines.xhtml

Appendix A

Table (33): Average medical expenditure (Rs.) for treatment under different heads of treatment during stay at hospitals as inpatient during last 365 days per hospitalisation case receiving medical treatment for each type of hospital, mpce class of household

All-India Urban type of hospital: public hospital + public dispensary

mpce class	average medical expenditure (Rs.) for treatment under different heads for treatment during stay at hospitals													estd. expn. (000)	hosp. cases receiving med. treatment on payment (00)	
	services							goods					total		estd. (00)	sample
	doctor's/ surgeons fee		diag-nostic test	bed char-ges	atten-dant char-ges	physio-therapy	other servi-ces	medicines		perso-nal medi-cal applia-nces	food etc.	blood, oxy-gen, etc.				
	hospital staff	other specia-list						from hos-pital	from out-side							
(1)	(2)	(3)	(4)	(5)	(6)	(7)	(8)	(9)	(10)	(11)	(12)	(13)	(14)	(15)	(16)	(17)
0 –300	7	1	68	1	3	0	3	57	522	2	78	20	761	27912	367	60
300 – 350	76	1	147	0	20	0	33	38	962	27	70	178	1551	68189	440	54
350 – 425	4	0	116	4	21	0	22	18	447	9	69	24	735	64556	878	123
425 – 500	24	3	164	14	3	0	7	32	557	8	69	17	899	142115	1581	231
500 – 575	23	1	244	29	5	0	18	14	1138	2	120	7	1601	126880	793	107
575 – 665	51	6	102	20	2	7	28	38	589	13	113	50	1020	114836	1126	188
665 – 775	68	20	116	34	5	24	11	11	790	47	103	39	1267	172604	1362	221
775 – 915	94	13	97	12	12	2	25	28	772	4	133	16	1207	144364	1196	216
915 – 1120	48	22	402	70	24	0	25	100	1237	16	123	33	2101	207686	988	215
1120 – 1500	24	89	670	111	117	1	59	94	1448	140	203	13	2970	237135	798	195
1500 – 1925	268	7	572	46	86	3	82	153	1531	27	116	50	2940	53288	181	49
1925 +	390	253	420	318	74	84	83	151	1528	137	208	165	3812	108550	285	75
all classes	59	22	219	39	22	7	25	45	855	29	111	37	1469	1468114	9995	1734

For peer review only - http://bmjopen.bmj.com/site/about/guidelines.xhtml

For peer review only

Appendix A

Table (33): Average medical expenditure (Rs.) for treatment under different heads of treatment during stay at hospitals as inpatient during last 365 days per hospitalisation case receiving medical treatment for each type of hospital, mpce class of household

All-India Urban type of hospital: private hospital

mpce class	average medical expenditure (Rs.) for treatment under different heads for treatment during stay at hospitals: services: doctor's/surgeons fee: hospital staff	services: doctor's/surgeons fee: other specia-list	services: diag-nostic test	services: bed char-ges	services: atten-dant char-ges	services: physio-therapy	services: other servi-ces	goods: medicines: from hos-pital	goods: medicines: from out-side	goods: perso-nal medi-cal applia-nces	goods: food etc.	goods: blood, oxy-gen, etc.	total	estd. expn. (000)	hosp. cases receiving med. treatment on payment (00): estd. (00)	hosp. cases receiving med. treatment on payment (00): sample
(1)	(2)	(3)	(4)	(5)	(6)	(7)	(8)	(9)	(10)	(11)	(12)	(13)	(14)	(15)	(16)	(17)
0 –300	1086	450	347	420	20	0	0	513	2646	10	157	54	5703	185815	326	38
300 – 350	122	363	181	563	79	19	0	253	923	0	75	40	2618	25869	99	21
350 – 425	521	181	269	314	23	6	2	451	1052	3	149	28	3000	117664	392	63
425 – 500	754	599	807	833	22	4	78	630	2811	143	182	208	7070	581934	823	123
500 – 575	443	165	261	614	52	18	14	449	415	21	108	32	2592	70193	271	50
575 – 665	921	61	279	654	14	2	56	449	1058	506	90	56	4148	452497	1091	127
665 – 775	951	338	327	521	56	7	5	479	852	15	177	37	3766	444346	1180	154
775 – 915	1126	361	582	849	72	54	52	997	1521	98	159	111	5982	595027	995	158
915 – 1120	1708	1020	1070	888	119	52	68	901	1562	31	185	128	7731	998813	1292	179
1120 – 1500	937	309	464	732	121	35	68	758	1145	184	140	101	4992	878318	1760	215
1500 – 1925	1205	636	911	1244	115	46	23	594	1890	70	383	58	7176	478087	666	71
1925 +	1654	837	1166	1277	83	62	142	762	2043	103	258	431	8819	782496	887	128
all classes	1092	475	622	791	74	30	55	677	1490	132	177	122	5737	5611059	9781	1327

For peer review only - http://bmjopen.bmj.com/site/about/guidelines.xhtml

Appendix A

Table (33): Average medical expenditure (Rs.) for treatment under different heads of treatment during stay at hospitals as inpatient during last 365 days per hospitalisation case receiving medical treatment for each type of hospital, mpce class of household

All-India Urban type of hospital: all

For peer review only

mpce class	average medical expenditure (Rs.) for treatment under different heads for treatment during stay at hospitals													estd. expn. (000)	hosp. cases receiving med. treatment on payment (00)	
	services							goods					total		estd. (00)	sample
	doctor's/ surgeons fee		diag-nostic test	bed char-ges	atten-dant char-ges	physio-therapy	other servi-ces	medicines		perso-nal medi-cal applia-nces	food etc.	blood, oxy-gen, etc.				
	hospital staff	other specia-list						from hos-pital	from out-side							
(1)	(2)	(3)	(4)	(5)	(6)	(7)	(8)	(9)	(10)	(11)	(12)	(13)	(14)	(15)	(16)	(17)
0 –300	515	212	199	198	11	0	1	271	1521	6	115	36	3086	213727	692	98
300 – 350	84	67	153	103	31	4	27	77	955	22	71	152	1747	94058	538	75
350 – 425	164	56	163	99	22	2	16	152	634	8	94	25	1435	182219	1270	186
425 – 500	274	207	384	295	10	1	31	237	1328	54	108	83	3011	724050	2405	354
500 – 575	130	43	248	178	17	5	17	125	954	7	117	13	1853	197073	1063	157
575 – 665	479	34	189	332	8	5	42	241	820	256	102	53	2560	567332	2216	315
665 – 775	478	167	214	260	29	16	8	228	819	32	137	38	2427	616950	2542	375
775 – 915	563	171	317	392	39	25	37	468	1112	46	145	59	3376	739391	2190	374
915 – 1120	989	587	780	534	78	30	49	554	1421	25	158	87	5291	1206499	2280	394
1120 – 1500	652	241	528	538	120	24	66	550	1240	170	160	74	4361	1115453	2558	410
1500 – 1925	1005	501	839	988	109	36	36	500	1813	61	326	57	6270	531375	847	120
1925 +	1347	695	985	1044	81	68	128	613	1918	111	246	367	7602	891046	1172	203
all classes	570	246	418	411	48	18	40	358	1169	80	144	79	3580	7079172	19776	3061

For peer review only - http://bmjopen.bmj.com/site/about/guidelines.xhtml

Appendix A

Table (33): Average medical expenditure (Rs.) for treatment under different heads of treatment during stay at hospitals as inpatient during last 365 days per hospitalisation case receiving medical treatment for each type of hospital, mpce class of household

All-India | Rural + Urban | type of hospital: public hospital

mpce class	average medical expenditure (Rs.) for treatment under different heads for treatment during stay at hospitals: services: doctor's/surgeons fee: hospital staff	services: doctor's/surgeons fee: other specia-list	services: diag-nostic test	services: bed char-ges	services: atten-dant char-ges	services: physio-therapy	services: other servi-ces	goods: medicines: from hos-pital	goods: medicines: from out-side	goods: perso-nal medi-cal applia-nces	goods: food etc.	goods: blood, oxy-gen, etc.	total	estd. expn. (000)	hosp. cases receiving med. treatment on payment (00): estd. (00)	hosp. cases receiving med. treatment on payment (00): sample
(1)	(2)	(3)	(4)	(5)	(6)	(7)	(8)	(9)	(10)	(11)	(12)	(13)	(14)	(15)	(16)	(17)
0 – 235	17	2	40	3	8	0	1	27	661	8	103	79	948	132447	1397	186
235 – 265	33	10	110	5	8	0	18	30	939	10	88	81	1332	198617	1491	164
265 – 320	14	9	89	5	9	0	17	19	757	8	97	42	1066	316589	2970	378
320 – 365	14	16	114	7	2	0	9	31	725	4	114	17	1054	399496	3790	471
365 – 410	30	11	201	16	3	0	18	41	1029	6	163	9	1527	406986	2665	364
410 – 460	33	11	96	16	7	2	16	34	734	11	142	35	1139	479057	4206	505
460 – 520	41	37	190	50	8	8	13	28	941	23	116	26	1482	548665	3703	502
520 – 605	32	41	211	31	16	1	22	48	931	21	142	49	1545	653091	4227	587
605 – 730	16	28	222	39	22	5	28	45	874	49	133	13	1474	662396	4493	598
730 – 980	74	27	263	34	36	0	18	62	952	46	134	34	1680	652678	3885	579
980 – 1285	80	56	404	48	55	13	79	79	1445	14	143	64	2482	473334	1907	243
1285 +	114	41	289	108	12	4	33	77	1262	20	142	74	2176	393996	1811	251
all classes	38	25	185	29	15	3	21	42	909	21	129	37	1455	5317352	36545	4828

For peer review only - http://bmjopen.bmj.com/site/about/guidelines.xhtml

Appendix A

Table (33): Average medical expenditure (Rs.) for treatment under different heads of treatment during stay at hospitals as inpatient during last 365 days per hospitalisation case receiving medical treatment for each type of hospital, mpce class of household

All-India Rural + Urban type of hospital: public dispensary

mpce class	average medical expenditure (Rs.) for treatment under different heads for treatment during stay at hospitals													estd. expn. (000)	hosp. cases receiving med. treatment on payment (00)	
	services							goods					total		estd. (00)	sample
	doctor's/ surgeons fee		diag- nostic test	bed char- ges	atten- dant char- ges	physio- therapy	other servi- ces	medicines		perso- nal medi- cal applia- nces	food etc.	blood, oxy- gen, etc.				
	hospital staff	other specia- list						from hos- pital	from out- side							
(1)	(2)	(3)	(4)	(5)	(6)	(7)	(8)	(9)	(10)	(11)	(12)	(13)	(14)	(15)	(16)	(17)
0 – 235	11	0	2	0	20	0	16	0	648	0	137	0	834	3476	42	5
235 – 265	11	72	81	0	0	0	8	0	1269	0	161	0	1602	5566	35	8
265 – 320	1	0	46	0	0	0	0	7	179	0	80	0	313	3314	106	11
320 – 365	13	132	83	0	0	0	0	19	1640	0	173	38	2098	18659	89	13
365 – 410	225	0	616	110	23	0	40	98	902	0	190	0	2203	24677	112	9
410 – 460	7	0	43	5	6	0	0	3	1093	0	204	16	1377	15370	112	17
460 – 520	207	4	165	63	0	23	1	0	820	0	73	23	1379	22662	164	16
520 – 605	305	10	109	10	0	0	12	3	807	4	68	24	1352	32175	238	23
605 – 730	208	48	571	181	0	0	5	167	1259	20	449	42	2950	34643	117	22
730 – 980	0	183	222	277	22	0	63	75	1241	26	131	0	2240	25226	113	11
980 – 1285	1042	18	301	473	28	252	340	982	3640	12	194	27	7310	67575	92	13
1285 +	1078	581	539	435	194	409	287	88	1345	215	1012	341	6524	30357	47	5
all classes	241	57	227	112	14	36	48	108	1163	14	190	30	2241	283701	1266	153

For peer review only - http://bmjopen.bmj.com/site/about/guidelines.xhtml

Appendix A

Table (33): Average medical expenditure (Rs.) for treatment under different heads of treatment during stay at hospitals as inpatient during last 365 days per hospitalisation case receiving medical treatment for each type of hospital, mpce class of household

All-India Rural + Urban type of hospital: public hospital + public dispensary

mpce class	average medical expenditure (Rs.) for treatment under different heads for treatment during stay at hospitals: services: doctor's/surgeons fee: hospital staff	services: doctor's/surgeons fee: other specia-list	services: diag-nostic test	services: bed char-ges	services: atten-dant char-ges	services: physio-therapy	services: other servi-ces	goods: medicines: from hos-pital	goods: medicines: from out-side	goods: perso-nal medi-cal applia-nces	goods: food etc.	goods: blood, oxy-gen, etc.	total	estd. expn. (000)	hosp. cases receiving med. treatment on payment (00): estd. (00)	hosp. cases receiving med. treatment on payment (00): sample
(1)	(2)	(3)	(4)	(5)	(6)	(7)	(8)	(9)	(10)	(11)	(12)	(13)	(14)	(15)	(16)	(17)
0 – 235	16	2	39	3	8	0	2	27	660	8	104	77	945	135923	1438	191
235 – 265	32	11	109	5	8	0	18	29	947	10	90	79	1338	204183	1526	172
265 – 320	14	9	87	5	8	0	16	19	737	7	97	40	1040	319903	3076	389
320 – 365	14	19	114	7	2	0	8	31	746	4	115	17	1078	418155	3879	484
365 – 410	37	11	218	20	4	0	19	43	1024	6	164	9	1554	431663	2777	373
410 – 460	33	11	95	16	7	2	16	33	744	10	144	35	1145	494427	4318	522
460 – 520	48	35	189	51	8	9	13	27	936	22	114	26	1477	571327	3867	518
520 – 605	47	39	206	30	15	1	21	46	924	20	138	48	1535	685267	4465	610
605 – 730	20	29	231	42	21	5	28	48	884	49	141	14	1512	697039	4610	620
730 – 980	72	32	262	41	35	0	20	62	960	45	134	33	1696	677904	3998	590
980 – 1285	125	54	399	68	54	24	91	121	1547	14	146	62	2705	540909	1999	256
1285 +	138	54	296	116	17	14	39	77	1264	25	164	81	2285	424353	1857	256
all classes	45	26	187	32	15	4	22	45	918	21	131	37	1481	5601053	37811	4981

For peer review only - http://bmjopen.bmj.com/site/about/guidelines.xhtml

Appendix A

Table (33): Average medical expenditure (Rs.) for treatment under different heads of treatment during stay at hospitals as inpatient during last 365 days per hospitalisation case receiving medical treatment for each type of hospital, mpce class of household

All-India Rural + Urban type of hospital: private hospital

mpce class	average medical expenditure (Rs.) for treatment under different heads for treatment during stay at hospitals													estd. expn. (000)	hosp. cases receiving med. treatment on payment (00)	
	services							goods					total		estd. (00)	sample
	doctor's/ surgeons fee		diag-nostic test	bed char-ges	atten-dant char-ges	physio-therapy	other servi-ces	medicines		perso-nal medi-cal applia-nces	food etc.	blood, oxy-gen, etc.				
	hospital staff	other specia-list						from hos-pital	from out-side							
(1)	(2)	(3)	(4)	(5)	(6)	(7)	(8)	(9)	(10)	(11)	(12)	(13)	(14)	(15)	(16)	(17)
0 – 235	548	277	238	332	11	0	0	292	1671	19	143	32	3563	278613	782	91
235 – 265	299	182	294	501	82	3	1	60	964	2	166	23	2576	193417	751	62
265 – 320	844	190	331	497	33	3	11	499	1313	18	208	21	3968	576371	1452	194
320 – 365	496	387	422	578	22	1	35	561	1620	54	161	165	4501	1008684	2241	276
365 – 410	512	290	353	452	37	19	46	334	1087	8	247	22	3407	626267	1838	212
410 – 460	793	247	347	515	36	25	44	359	1185	215	191	103	4059	1129887	2784	325
460 – 520	871	304	376	517	51	22	22	567	1005	8	223	64	4030	1422677	3531	404
520 – 605	770	382	517	681	90	39	45	606	1499	31	186	176	5021	2004523	3992	451
605 – 730	1284	416	556	734	70	16	52	574	1392	15	222	62	5392	2387265	4427	482
730 – 980	1194	370	505	799	120	23	136	726	1511	145	211	133	5871	3279757	5586	559
980 – 1285	1164	286	615	752	87	48	39	348	1849	43	305	105	5640	1445581	2563	242
1285 +	1354	543	798	1017	83	50	85	660	1941	58	264	166	7017	2898911	4131	383
all classes	975	358	499	684	71	26	59	543	1460	63	218	108	5062	1725195<br>3	34078	3681

For peer review only - http://bmjopen.bmj.com/site/about/guidelines.xhtml

Appendix A

Table (33): Average medical expenditure (Rs.) for treatment under different heads of treatment during stay at hospitals as inpatient during last 365 days per hospitalisation case receiving medical treatment for each type of hospital, mpce class of household

All-India Rural + Urban type of hospital: all

mpce class	average medical expenditure (Rs.) for treatment under different heads for treatment during stay at hospitals													estd. expn. (000)	hosp. cases receiving med. treatment on payment (00)	
	services							goods					total		estd. (00)	sample
	doctor's/ surgeons fee		diag-nostic test	bed char-ges	atten-dant char-ges	physio-therapy	other servi-ces	medicines		perso-nal medi-cal applia-nces	food etc.	blood, oxy-gen, etc.				
	hospital staff	other specia-list						from hos-pital	from out-side							
(1)	(2)	(3)	(4)	(5)	(6)	(7)	(8)	(9)	(10)	(11)	(12)	(13)	(14)	(15)	(16)	(17)
0 – 235	204	99	109	119	9	0	1	120	1016	12	118	61	1867	414536	2220	282
235 – 265	120	68	170	169	32	1	12	39	952	7	115	60	1746	397600	2277	234
265 – 320	280	67	165	163	16	1	15	173	922	11	132	34	1979	896275	4528	583
320 – 365	191	153	227	216	9	0	18	225	1066	23	132	71	2331	1426839	6120	760
365 – 410	227	122	271	192	17	8	30	159	1050	7	197	14	2292	1059960	4624	587
410 – 460	330	104	194	212	18	11	27	161	917	90	162	61	2288	1625945	7107	848
460 – 520	441	164	278	273	28	15	17	284	969	15	166	44	2695	1994542	7400	923
520 – 605	388	201	353	337	50	19	32	310	1195	25	161	108	3180	2689790	8457	1061
605 – 730	639	220	390	381	45	10	39	306	1133	32	181	37	3413	3085506	9040	1103
730 – 980	726	229	403	482	85	13	87	449	1281	103	179	91	4129	3957661	9584	1149
980 – 1285	709	185	520	452	72	37	62	248	1716	30	235	86	4354	1986491	4563	498
1285 +	977	391	642	738	62	39	71	479	1731	48	233	140	5550	3323264	5988	639
all classes	486	183	335	341	42	14	39	281	1175	41	172	70	3179	2285840 7	71909	8667

For peer review only - http://bmjopen.bmj.com/site/about/guidelines.xhtml

Table (34): Number per 1000 of hospitalised cases getting reimbursement, average amount of reimbursement and per 1000 distribution of such cases by source for mpce class of households

All-India Rural

mpce class	no. per 1000 of hospitalised cases getting reimbursement	average amount of reimbursement (Rs.) per hosp. case	distribution of expenses reimbursed by source					hospitalised cases getting reimbursement	
			employer		medical insurance companies	other agencies	total	estd. (00)	sample
			government	private					
(1)	(2)	(3)	(4)	(5)	(6)	(7)	(8)	(9)	(10)
					males				
0 – 225	9	16	796	19	92	92	1000	27	3
225 – 255	2	3	0	500	0	500	1000	3	1
255 – 300	3	8	252	69	118	561	1000	15	4
300 – 340	2	3	692	103	103	103	1000	11	4
340 – 380	12	161	150	763	0	87	1000	80	7
380 – 420	4	15	191	415	6	388	1000	40	6
420 – 470	5	64	94	750	6	151	1000	42	7
470 – 525	1	10	332	415	107	146	1000	11	6
525 – 615	3	16	1000	0	0	0	1000	34	6
615 – 775	17	111	662	152	0	187	1000	242	19
775 – 950	11	330	37	36	927	0	1000	76	10
950 +	14	400	695	180	26	99	1000	158	30
all classes	8	109	455	237	210	98	1000	740	103
					females				
0 – 225	2	10	0	0	0	1000	1000	5	1
225 – 255	3	15	250	250	250	250	1000	6	1
255 – 300	10	30	411	222	183	183	1000	44	4
300 – 340	0	2	1000	0	0	0	1000	2	1
340 – 380	1	2	655	345	0	0	1000	7	2
380 – 420	3	3	1000	0	0	0	1000	25	3
420 – 470	14	48	327	216	161	296	1000	111	8
470 – 525	3	8	8	420	3	569	1000	27	7
525 – 615	3	19	931	9	9	52	1000	31	6
615 – 775	5	47	592	407	0	0	1000	61	8
775 – 950	6	31	996	1	1	3	1000	40	9
950 +	16	194	821	0	165	14	1000	147	22
all classes	6	42	701	112	115	72	1000	506	72
					persons				
0 – 225	6	13	514	12	60	414	1000	32	4
225 – 255	2	10	213	287	213	287	1000	10	2
255 – 300	6	18	371	184	167	278	1000	59	8
300 – 340	1	3	780	73	73	73	1000	13	5
340 – 380	7	87	154	759	0	86	1000	87	9
380 – 420	4	10	314	352	5	329	1000	65	9
420 – 470	9	56	192	526	71	212	1000	152	15
470 – 525	2	9	191	418	62	330	1000	38	13
525 – 615	3	17	966	4	4	26	1000	65	12
615 – 775	11	82	644	218	0	138	1000	303	27
775 – 950	9	181	119	33	847	0	1000	116	19
950 +	15	305	732	127	66	75	1000	305	52
all classes	7	78	517	205	186	91	1000	1247	175

For peer review only

For peer review only - http://bmjopen.bmj.com/site/about/guidelines.xhtml

Table (34): Number per 1000 of hospitalised cases getting reimbursement, average amount of reimbursement and per 1000 distribution of such cases by source for mpce class of households

All-India Urban

mpce class	no. per 1000 of hospi-talised cases getting reim-burse-ment	average amount of reimbur-sement (Rs.) per hosp. case	distribution of expenses reimbursed by source					hospitalised cases getting reimbursement	
			employer		medical insurance compa-nies	other agencies	total	estd. (00)	sample
			govern-ment	private					
(1)	(2)	(3)	(4)	(5)	(6)	(7)	(8)	(9)	(10)
					males				
0 –300	24	1689	998	2	0	0	1000	30	4
300 – 350	5	23	1000	0	0	0	1000	4	1
350 – 425	0	0	0	0	0	0	0	0	0
425 – 500	8	127	9	366	613	12	1000	33	6
500 – 575	11	115	463	8	522	8	1000	21	4
575 – 665	7	141	48	73	0	879	1000	28	9
665 – 775	19	61	590	181	221	8	1000	97	14
775 – 915	32	200	608	270	53	69	1000	141	18
915 – 1120	25	360	701	63	230	7	1000	119	24
1120 – 1500	28	698	703	178	102	17	1000	179	42
1500 – 1925	181	3133	658	84	253	5	1000	355	40
1925 +	188	5224	221	218	514	47	1000	609	69
all classes	40	844	445	167	345	43	1000	1617	231
					females				
0 –300	1	14	1000	0	0	0	1000	1	2
300 – 350	0	0	0	0	0	0	0	0	0
350 – 425	0	1	1000	0	0	0	1000	0	1
425 – 500	1	3	15	985	0	0	1000	4	3
500 – 575	14	147	45	45	866	45	1000	27	3
575 – 665	2	24	257	248	248	248	1000	9	2
665 – 775	27	119	399	105	390	105	1000	108	10
775 – 915	29	87	225	1	501	273	1000	128	19
915 – 1120	29	284	229	17	752	3	1000	144	28
1120 – 1500	51	546	293	146	560	1	1000	262	30
1500 – 1925	100	1091	724	27	211	38	1000	189	26
1925 +	153	3046	188	197	449	166	1000	544	72
all classes	38	497	272	148	467	113	1000	1417	196
					persons				
0 –300	15	1016	998	2	0	0	1000	30	6
300 – 350	3	11	1000	0	0	0	1000	4	1
350 – 425	0	0	1000	0	0	0	1000	0	1
425 – 500	5	69	10	381	598	11	1000	37	9
500 – 575	12	131	229	28	714	28	1000	49	7
575 – 665	5	81	80	100	38	782	1000	37	11
665 – 775	22	86	475	135	323	67	1000	206	24
775 – 915	31	144	493	189	187	130	1000	270	37
915 – 1120	27	322	489	42	464	5	1000	263	52
1120 – 1500	38	630	545	166	278	11	1000	441	72
1500 – 1925	141	2131	675	69	242	13	1000	543	66
1925 +	170	4082	208	210	489	94	1000	1154	141
all classes	39	677	384	160	388	68	1000	3034	427

For peer review only - http://bmjopen.bmj.com/site/about/guidelines.xhtml

Appendix A

Table (34): Number per 1000 of hospitalised cases getting reimbursement, average amount of reimbursement and per 1000 distribution of such cases by source for mpce class of households

All-India Rural + Urban

mpce class	no. per 1000 of hospi-talised cases getting reim-burse-ment	average amount of reimbur-sement (Rs.) per hosp. case	distribution of expenses reimbursed by source					hospitalised cases getting reimbursement	
			employer		medical insurance compa-nies	other agencies	total	estd. (00)	sample
			govern-ment	private					
(1)	(2)	(3)	(4)	(5)	(6)	(7)	(8)	(9)	(10)
					males				
0 – 235	14	507	994	2	2	2	1000	57	7
235 – 265	3	8	714	143	0	143	1000	7	2
265 – 320	2	6	252	69	118	561	1000	15	4
320 – 365	4	52	37	356	592	15	1000	44	10
365 – 410	11	151	203	634	89	74	1000	101	11
410 – 460	5	51	77	144	1	777	1000	68	15
460 – 520	10	63	279	537	86	97	1000	139	21
520 – 605	11	68	581	284	58	77	1000	153	24
605 – 730	9	114	731	56	207	6	1000	154	30
730 – 980	20	291	692	171	75	62	1000	421	61
980 – 1285	50	962	493	71	432	4	1000	431	50
1285 +	53	1485	320	210	412	58	1000	767	99
all classes	17	326	448	183	313	56	1000	2357	334
					females				
0 – 235	2	11	313	0	0	687	1000	6	3
235 – 265	2	12	250	250	250	250	1000	6	1
265 – 320	7	21	418	220	181	181	1000	45	5
320 – 365	1	2	416	584	0	0	1000	6	4
365 – 410	4	38	64	54	838	43	1000	35	5
410 – 460	3	11	409	197	197	197	1000	34	5
460 – 520	18	72	367	154	288	190	1000	219	18
520 – 605	11	32	190	69	420	321	1000	155	26
605 – 730	12	107	311	16	665	8	1000	174	34
730 – 980	19	196	343	190	465	1	1000	323	38
980 – 1285	27	264	749	25	192	35	1000	228	35
1285 +	53	976	279	169	408	144	1000	692	94
all classes	16	182	340	143	411	106	1000	1923	268
					persons				
0 – 235	8	285	982	2	2	14	1000	63	10
235 – 265	2	10	424	210	156	210	1000	14	3
265 – 320	4	13	376	182	165	276	1000	59	9
320 – 365	3	30	51	364	570	15	1000	50	14
365 – 410	8	98	178	529	225	68	1000	136	16
410 – 460	4	32	128	152	31	688	1000	102	20
460 – 520	14	67	324	344	188	144	1000	358	39
520 – 605	11	50	457	216	173	154	1000	308	50
605 – 730	10	111	540	38	415	7	1000	328	64
730 – 980	20	248	568	178	214	40	1000	744	99
980 – 1285	38	614	548	61	381	10	1000	660	85
1285 +	53	1243	305	195	411	90	1000	1459	193
all classes	17	258	412	170	345	73	1000	4280	602

For peer review only - http://bmjopen.bmj.com/site/about/guidelines.xhtml

Appendix A

Table (35): Number per 1000 of persons reporting ailment during the last 15 days by sex and age-group for each State/UT

Rural

state / ut	male age group (*years*)						female age group (*years*)						person age group (*years*)						persons reporting ailment*	
	0 - 14	15 - 29	30 - 44	45 - 59	60 & above	all	0 - 14	15 - 29	30 - 44	45 - 59	60 & above	all	0 - 14	15 - 29	30 - 44	45 - 59	60 & above	all	estd. no. (00)	sample
(1)	(2)	(3)	(4)	(5)	(6)	(7)	(8)	(9)	(10)	(11)	(12)	(13)	(14)	(15)	(16)	(17)	(18)	(19)	(20)	(21)
Andhra Pradesh	76	30	57	123	344	86	57	38	79	145	372	93	67	34	68	134	359	90	46725	1619
Arunachal Pradesh	55	48	38	46	246	61	49	62	30	79	214	61	52	55	34	61	231	61	480	242
Assam	96	34	35	65	363	78	97	43	52	94	407	86	96	38	43	79	381	82	17667	884
Bihar	61	22	22	51	180	53	63	22	38	62	157	53	62	22	30	56	170	53	32720	1101
Chhattisgarh	63	46	72	88	156	69	59	49	80	79	146	70	61	47	76	83	150	69	12059	445
Delhi	9	0	0	0	0	4	0	9	0	0	0	4	5	6	0	0	0	4	69	3
Goa	173	0	93	136	650	137	51	11	95	203	628	138	106	5	94	167	638	138	1544	39
Gujarat	58	29	50	62	358	70	52	38	52	103	244	68	55	34	51	83	302	69	21330	632
Haryana	67	63	76	119	245	86	61	96	132	140	213	106	65	78	104	129	229	95	13735	508
Himachal Pradesh	44	31	50	109	288	73	38	41	151	173	261	101	41	37	101	143	274	87	4610	655
Jammu & Kashmir	68	18	36	86	338	70	51	29	72	82	353	69	60	23	54	84	344	70	4157	351
Jharkhand	33	7	9	16	96	24	46	37	31	53	69	43	39	20	20	34	85	33	6398	249
Karnataka	45	19	38	90	331	63	47	27	47	107	258	64	46	23	42	98	296	64	20918	707
Kerala	222	118	192	315	562	242	186	160	220	390	586	266	205	141	207	353	575	255	59661	2155
Madhya Pradesh	48	36	49	51	225	57	40	47	78	101	200	66	44	42	63	74	213	61	27731	848
Maharashtra	78	33	51	100	313	84	83	60	91	118	301	103	81	46	71	109	307	93	52275	1449
Manipur	41	11	18	22	70	27	36	14	30	33	86	30	38	12	25	27	77	28	411	158
Meghalaya	71	30	25	9	165	51	48	26	35	115	172	51	60	28	30	59	168	51	970	154
Mizoram	34	17	3	12	84	23	27	4	13	0	105	18	31	10	8	6	92	20	80	34
Nagaland	114	12	0	31	251	61	76	92	6	69	147	63	95	50	3	50	223	62	257	74
Orissa	83	49	64	87	163	79	74	48	73	90	159	76	79	49	68	89	161	77	23398	761
Punjab	95	69	99	157	303	114	78	99	202	261	417	160	87	83	151	205	358	136	21168	647
Rajasthan	46	26	47	83	164	52	36	46	83	97	135	61	42	36	65	90	149	57	22848	921
Sikkim	83	38	36	105	195	69	47	1	56	18	203	40	64	19	46	68	199	55	212	124

Continued

For peer review only

For peer review only - http://bmjopen.bmj.com/site/about/guidelines.xhtml

Appendix A

Table (35): Number per 1000 of persons reporting ailment during the last 15 days by sex and age-group for each State/UT

Rural

state / ut	male						female						person						persons reporting ailment*	
	age group (*years*)						age group (*years*)						age group (*years*)							
	0 - 14	15 - 29	30 - 44	45 - 59	60 & above	all	0 - 14	15 - 29	30 - 44	45 - 59	60 & above	all	0 - 14	15 - 29	30 - 44	45 - 59	60 & above	all	estd. no. (00)	sample
(1)	(2)	(3)	(4)	(5)	(6)	(7)	(8)	(9)	(10)	(11)	(12)	(13)	(14)	(15)	(16)	(17)	(18)	(19)	(20)	(21)
Tamil Nadu	69	29	57	138	287	86	56	54	130	122	296	103	63	43	95	130	292	95	37581	1102
Tripura	186	71	85	104	264	126	147	88	124	155	330	135	169	80	103	131	291	130	3407	501
Uttaranchal	61	39	11	39	165	54	23	49	83	22	135	49	45	44	48	30	152	52	3293	120
Uttar Pradesh	87	55	87	119	286	96	72	81	118	162	266	104	80	68	103	140	276	100	128696	4368
West Bengal	110	55	99	162	357	115	98	61	100	155	369	113	104	58	99	158	363	114	66800	1839
A & N Islands	10	0	53	63	297	41	106	23	16	87	187	63	50	12	36	73	257	51	115	36
Chandigarh	23	4	37	15	508	26	116	53	75	58	323	89	70	22	51	33	409	52	47	40
Dadra & N. Haveli	16	5	13	0	13	10	22	25	0	25	95	21	19	15	7	11	39	15	35	13
Daman & Diu	37	2	0	0	0	6	34	28	0	263	155	43	35	10	0	127	119	19	18	14
Lakshadweep	67	49	49	95	380	81	67	72	62	372	617	142	67	61	55	228	508	112	27	58
Pondicherry	196	124	84	34	532	162	108	0	94	84	724	132	161	70	89	62	624	149	490	58
ONES	99	42	44	56	197	73	72	50	60	99	220	73	86	46	52	77	207	73	5817	1287
GUTs	75	38	48	31	343	69	79	21	50	82	492	79	77	31	49	55	408	73	733	219
all-India	76	41	64	107	285	83	68	57	93	132	282	93	72	49	78	119	283	88	631934	22909
estd. no. of persons reporting ailment* (00)	104236	36970	43897	45571	74362	305036	85021	51105	63830	55366	71576	326898	189257	88074	107728	100937	145938	631934	X	X
sample persons reporting ailment*	3251	1399	1536	1688	3413	11287	2576	1727	2083	2116	3120	11622	5827	3126	3619	3804	6533	22909	X	X
estd. no. of persons (00)	1373037	908479	690923	424407	260664	3657509	1256497	900801	689161	420608	254124	3521192	2629534	1809280	1380084	845015	514787	7178701	X	X
sample persons	45943	33016	23467	14369	11834	128629	41673	32360	22896	15486	10934	123349	87616	65376	46363	29855	22768	251978	X	X

*during the last 15 days

For peer review only

For peer review only - http://bmjopen.bmj.com/site/about/guidelines.xhtml

Appendix A

Table (35): Number per 1000 of persons reporting ailment during the last 15 days by sex and age-group for each State/UT

Urban

state / ut	male						female						person						persons reporting ailment*	
	age group (*years*)						age group (*years*)						age group (*years*)							
	0 - 14	15 - 29	30 - 44	45 - 59	60 & above	all	0 - 14	15 - 29	30 - 44	45 - 59	60 & above	all	0 - 14	15 - 29	30 - 44	45 - 59	60 & above	all	estd. no. (00)	sample
(1)	(2)	(3)	(4)	(5)	(6)	(7)	(8)	(9)	(10)	(11)	(12)	(13)	(14)	(15)	(16)	(17)	(18)	(19)	(20)	(21)
Andhra Pradesh	77	35	106	142	511	102	104	68	74	197	562	127	90	51	91	170	538	114	22574	1152
Arunachal Pradesh	71	54	15	7	60	46	78	29	56	3	211	57	75	41	34	6	122	51	52	73
Assam	67	32	54	59	400	72	106	42	79	120	322	95	85	37	66	85	365	83	1868	185
Bihar	31	32	27	179	246	63	59	37	57	65	239	64	44	34	41	126	243	63	4631	243
Chhattisgarh	48	44	23	132	249	63	46	38	75	220	202	81	47	41	48	171	224	72	2035	170
Delhi	9	11	5	13	135	15	8	6	15	17	155	17	8	9	10	15	145	16	1575	111
Goa	71	85	94	11	163	77	107	50	89	102	399	115	88	69	91	55	291	95	532	51
Gujarat	80	29	54	94	380	77	50	36	75	122	288	78	66	32	64	108	331	78	12335	605
Haryana	52	82	63	125	320	86	41	64	109	134	252	89	47	74	84	129	281	87	4202	277
Himachal Pradesh	42	5	14	59	317	39	53	43	95	104	310	78	48	24	54	81	313	59	341	88
Jammu & Kashmir	86	34	17	103	383	75	70	18	56	196	312	81	78	26	37	147	351	78	1062	166
Jharkhand	35	34	9	44	203	42	30	36	36	181	111	59	33	35	22	109	158	50	1968	152
Karnataka	39	28	32	73	285	53	33	15	55	88	363	61	36	21	44	81	324	57	7758	595
Kerala	255	122	137	322	529	235	198	136	153	363	574	244	226	129	146	344	554	240	19647	1063
Madhya Pradesh	74	25	41	52	246	60	52	40	74	104	252	71	64	32	57	77	249	65	9589	561
Maharashtra	121	60	71	162	400	115	79	59	105	198	437	122	101	60	88	179	419	118	44858	1667
Manipur	40	10	13	2	42	20	44	11	40	24	87	34	42	11	28	13	64	27	151	79
Meghalaya	51	3	7	15	55	20	95	49	11	186	180	75	76	30	9	94	127	50	140	60
Mizoram	16	9	4	6	120	17	30	5	8	9	120	18	23	7	6	8	120	17	50	90
Nagaland	102	0	0	7	255	58	86	13	15	68	0	46	95	7	7	37	229	52	125	34
Orissa	89	8	47	55	149	56	56	29	27	78	199	52	74	19	38	66	171	54	2195	177
Punjab	82	76	90	119	288	100	72	48	113	260	341	115	77	63	100	185	313	107	8130	408
Rajasthan	64	35	59	81	278	69	51	44	107	76	254	76	58	40	84	79	266	72	7589	520
Sikkim	4	14	0	0	29	8	16	14	0	33	262	18	10	14	0	10	122	13	7	13

Continued

For peer review only - http://bmjopen.bmj.com/site/about/guidelines.xhtml

For peer review only

Appendix A

Table (35): Number per 1000 of persons reporting ailment during the last 15 days by sex and age-group for each State/UT

Urban

state / ut	male						female						person						persons reporting ailment*	
	age group (*years*)						age group (*years*)						age group (*years*)							
	0 - 14	15 - 29	30 - 44	45 - 59	60 & above	all	0 - 14	15 - 29	30 - 44	45 - 59	60 & above	all	0 - 14	15 - 29	30 - 44	45 - 59	60 & above	all	estd. no. (00)	sample
(1)	(2)	(3)	(4)	(5)	(6)	(7)	(8)	(9)	(10)	(11)	(12)	(13)	(14)	(15)	(16)	(17)	(18)	(19)	(20)	(21)
Tamil Nadu	77	32	61	138	303	87	77	54	85	142	346	106	77	43	73	140	326	96	20654	1255
Tripura	85	18	83	67	292	72	61	46	88	77	170	71	75	33	85	72	229	72	305	93
Uttaranchal	93	18	19	40	336	65	40	35	67	111	155	65	73	27	42	77	235	65	1103	104
Uttar Pradesh	88	53	69	149	298	93	81	77	138	238	371	126	85	64	103	189	335	108	36455	1824
West Bengal	137	61	88	139	479	137	114	90	154	249	542	178	126	76	118	191	509	157	28999	1336
A & N Islands	12	98	11	138	336	58	55	59	63	50	143	61	34	78	31	99	249	59	61	39
Chandigarh	49	37	30	89	179	55	64	101	59	62	330	89	56	64	43	77	247	71	452	145
Dadra & N. Haveli	44	9	0	0	9	18	89	0	0	0	86	34	62	6	0	0	45	24	7	7
Daman & Diu	12	0	0	165	201	30	13	52	69	0	55	41	13	19	37	90	111	35	13	15
Lakshadweep	94	22	93	218	474	112	141	108	105	252	582	168	116	66	99	234	533	140	40	74
Pondicherry	159	59	58	232	579	149	168	77	96	402	680	225	163	68	75	313	641	186	1175	169
ONES	59	12	24	21	143	37	61	27	38	61	131	48	60	20	31	40	137	42	830	442
GUTs	99	46	39	151	384	94	111	85	75	206	545	148	105	64	55	177	473	119	1748	449
all-India	84	44	64	127	352	91	74	56	95	173	383	108	79	50	79	149	368	99	242677	13601
estd. no. of persons reporting ailment* (00)	32106	16745	17448	21321	28366	115986	25005	18704	24598	26164	32220	126691	57111	35450	42046	47484	60586	242677	X	X
sample persons reporting ailment*	1602	765	869	1118	2100	6454	1273	973	1148	1422	2331	7147	2875	1738	2017	2540	4431	13601	X	X
estd. no. of persons (00)	380698	376633	271398	167236	80686	1276650	339616	335668	259117	151140	84109	1169650	720314	712301	530515	318376	164795	2446300	X	X
sample persons	20212	19004	14366	8134	6354	68070	18332	18629	12974	8625	6447	65007	38544	37633	27340	16759	12801	133077	X	X

*during the last 15 days

For peer review only

For peer review only - http://bmjopen.bmj.com/site/about/guidelines.xhtml

Table (35): Number per 1000 of persons reporting ailment during the last 15 days by sex and age-group for each State/UT

Rural+Urban

state / ut	male						female						person						persons reporting ailment*	
	age group (*years*)						age group (*years*)						age group (*years*)							
	0 - 14	15 - 29	30 - 44	45 - 59	60 & above	all	0 - 14	15 - 29	30 - 44	45 - 59	60 & above	all	0 - 14	15 - 29	30 - 44	45 - 59	60 & above	all	estd. no. (00)	sample
(1)	(2)	(3)	(4)	(5)	(6)	(7)	(8)	(9)	(10)	(11)	(12)	(13)	(14)	(15)	(16)	(17)	(18)	(19)	(20)	(21)
Andhra Pradesh	76	32	72	128	381	91	69	47	77	158	417	102	73	39	75	143	400	96	69299	2771
Arunachal Pradesh	57	49	34	42	239	59	53	58	34	74	214	61	55	54	34	57	227	60	532	315
Assam	94	34	37	65	366	78	97	43	55	97	398	87	95	38	45	80	380	82	19535	1069
Bihar	59	24	23	68	188	54	62	24	40	63	166	54	60	24	31	65	178	54	37351	1344
Chhattisgarh	61	46	65	96	168	68	58	47	79	99	152	71	60	46	72	97	159	70	14094	615
Delhi	9	10	4	11	117	13	7	7	13	15	134	15	8	9	9	13	126	14	1644	114
Goa	134	29	93	91	498	117	69	26	93	164	558	131	100	28	93	125	531	124	2076	90
Gujarat	65	29	51	73	365	72	51	38	60	110	259	71	58	33	56	91	311	72	33665	1237
Haryana	64	68	72	121	259	86	57	88	126	138	223	102	61	77	98	129	241	93	17937	785
Himachal Pradesh	44	29	45	106	289	70	40	42	144	169	264	99	42	35	95	139	276	84	4951	743
Jammu & Kashmir	71	21	32	90	345	71	54	27	69	106	345	72	63	24	51	97	345	71	5219	517
Jharkhand	33	12	9	21	115	27	44	37	32	78	79	45	38	23	20	49	100	36	8366	401
Karnataka	43	22	36	85	319	60	43	23	49	101	286	63	43	22	43	93	303	62	28676	1302
Kerala	229	119	177	317	554	240	189	154	203	382	583	261	210	138	191	350	570	251	79308	3218
Madhya Pradesh	54	33	47	51	230	58	43	45	77	102	212	67	49	39	61	75	221	62	37320	1409
Maharashtra	93	45	60	127	344	97	82	60	97	149	349	110	88	52	78	138	346	103	97133	3116
Manipur	41	11	17	17	61	25	38	13	33	31	87	31	39	12	25	23	72	28	562	237
Meghalaya	69	26	23	10	156	48	53	30	32	127	173	54	61	28	27	66	164	51	1111	214
Mizoram	26	14	3	9	98	20	28	4	11	4	112	18	27	9	7	7	104	19	129	124
Nagaland	109	9	0	23	252	60	79	63	9	69	123	57	95	36	5	45	225	58	382	108
Orissa	84	43	61	83	161	76	72	45	68	89	163	73	78	44	65	86	162	75	25592	938
Punjab	91	71	96	144	299	109	76	81	174	261	397	146	84	76	134	198	346	127	29297	1055
Rajasthan	49	28	49	83	187	56	39	46	89	93	158	64	44	37	69	88	172	60	30437	1441
Sikkim	73	33	33	90	181	61	43	3	45	20	207	37	58	18	39	61	193	49	220	137

Continued

For peer review only - http://bmjopen.bmj.com/site/about/guidelines.xhtml

For peer review only

Appendix A

Table (35): Number per 1000 of persons reporting ailment during the last 15 days by sex and age-group for each State/UT

Rural+Urban

state / ut	male						female						person						persons reporting ailment*	
	age group (*years*)						age group (*years*)						age group (*years*)							
	0 - 14	15 - 29	30 - 44	45 - 59	60 & above	all	0 - 14	15 - 29	30 - 44	45 - 59	60 & above	all	0 - 14	15 - 29	30 - 44	45 - 59	60 & above	all	estd. no. (00)	sample
(1)	(2)	(3)	(4)	(5)	(6)	(7)	(8)	(9)	(10)	(11)	(12)	(13)	(14)	(15)	(16)	(17)	(18)	(19)	(20)	(21)
Tamil Nadu	71	30	58	138	292	86	63	54	114	129	314	104	68	43	87	133	303	95	58235	2357
Tripura	174	63	85	98	267	118	137	82	119	144	304	126	158	72	101	122	283	122	3712	594
Uttaranchal	66	34	13	39	195	57	25	46	79	42	140	53	49	40	46	41	169	55	4397	224
Uttar Pradesh	87	55	83	127	288	95	74	80	122	179	286	109	81	67	103	151	287	102	165152	6192
West Bengal	115	56	96	155	396	120	101	68	115	181	422	128	108	62	105	168	409	124	95799	3175
A & N Islands	11	31	35	82	304	46	90	36	33	76	176	62	46	33	34	80	255	53	177	75
Chandigarh	44	32	31	83	191	51	75	96	60	62	330	89	59	59	44	73	253	68	499	185
Dadra & N. Haveli	20	6	12	0	13	11	28	23	0	22	94	22	24	14	6	10	40	16	41	20
Daman & Diu	27	2	0	90	142	12	26	32	38	127	101	42	27	12	12	107	114	23	32	29
Lakshadweep	83	34	70	164	430	98	107	91	85	307	598	156	95	64	77	231	522	127	67	132
Pondicherry	173	84	67	181	559	154	148	53	95	298	692	194	162	69	80	240	636	173	1665	227
ONES	92	36	40	48	187	66	70	45	56	90	202	68	81	40	48	69	194	67	6647	1729
GUTs	88	43	43	113	367	83	96	60	65	165	529	121	92	51	53	138	449	101	2481	668
all-India	78	42	64	113	301	85	69	56	93	143	307	97	74	49	78	128	304	91	874611	36510
estd. no. of persons reporting ailment* (00)	136342	53715	61345	66892	102729	421022	110026	69809	88428	81530	103796	453589	246368	123524	149773	148422	206524	874611	X	X
sample persons reporting ailment*	4853	2164	2405	2806	5513	17741	3849	2700	3231	3538	5451	18769	8702	4864	5636	6344	10964	36510	X	X
estd. no. of persons (00)	1753735	1285112	962321	591642	341349	4934159	1596113	1236469	948278	571748	338233	4690841	3349848	2521581	1910599	1163390	679582	9625001	X	X
sample persons	66155	52020	37833	22503	18188	196699	60005	50989	35870	24111	17381	188356	126160	103009	73703	46614	35569	385055	X	X

*during the last 15 days

For peer review only - http://bmjopen.bmj.com/site/about/guidelines.xhtml

Appendix A

Table (36): Number per 1000 of persons reporting ailment during the last 15 days by mpce class and social group for each sex and State/UT

Rural

state / ut	sex	mpce class													social group				persons reporting ailment*	
		0 – 225	225 – 255	255 – 300	300 – 340	340 – 380	380 – 420	420 – 470	470 – 525	525 – 615	615 – 775	775 – 950	950 +	all	ST	SC	OBC	others	estd. no. (00)	sample
(1)	(2)	(3)	(4)	(5)	(6)	(7)	(8)	(9)	(10)	(11)	(12)	(13)	(14)	(15)	(16)	(17)	(18)	(19)	(20)	(21)
Andhra Pradesh	male	74	50	77	78	84	63	73	100	80	103	200	123	86	66	86	77	115	22346	815
	female	46	50	75	98	80	76	90	116	91	94	121	231	93	73	78	92	120	24380	804
	person	59	50	76	87	82	70	82	108	85	99	158	173	90	70	82	84	118	46725	1619
Arunachal Pradesh	male	73	0	0	88	27	64	97	102	47	48	65	46	61	71	271	0	22	248	124
	female	93	191	59	77	39	24	25	30	76	51	94	60	61	69	141	20	31	232	118
	person	83	102	26	82	33	44	62	68	61	49	77	53	61	70	220	10	26	480	242
Assam	male	119	59	139	72	65	91	95	62	55	74	88	75	78	68	65	89	80	8998	451
	female	196	31	127	71	110	88	82	74	72	76	51	151	86	66	86	84	93	8669	433
	person	158	43	133	72	87	89	89	67	63	75	71	110	82	67	75	87	86	17667	884
Bihar	male	40	31	43	37	61	65	51	58	90	56	89	65	53	53	55	55	44	16897	565
	female	24	19	35	49	52	63	79	90	63	69	78	79	53	53	52	54	53	15823	536
	person	32	25	40	43	57	64	64	73	78	62	84	71	53	53	53	54	48	32720	1101
Chhattisgarh	male	98	55	69	49	57	63	90	65	73	103	0	5	69	58	95	73	36	5978	222
	female	69	67	64	38	64	69	93	157	61	214	81	5	70	44	100	87	47	6080	223
	person	83	61	67	44	61	66	92	110	67	153	37	5	69	51	97	80	41	12059	445
Delhi	male	0	0	0	0	0	0	0	0	0	16	0	0	4	0	0	5	0	34	1
	female	0	0	0	0	0	0	0	0	0	0	21	3	4	0	0	3	6	35	2
	person	0	0	0	0	0	0	0	0	0	8	10	2	4	0	0	4	3	69	3
Goa	male	0	0	0	0	0	0	0	114	102	11	194	213	137	823	0	422	120	765	17
	female	0	0	0	0	0	0	107	81	240	98	16	174	138	478	0	422	125	779	22
	person	0	0	0	0	0	0	63	100	192	50	117	192	138	625	0	422	123	1544	39

Continued

For peer review only - http://bmjopen.bmj.com/site/about/guidelines.xhtml

Table (36): Number per 1000 of persons reporting ailment during the last 15 days by mpce class and social group for each sex and State/UT

Rural

state / ut	sex	mpce class													social group				persons reporting ailment*	
		0 – 225	225 – 255	255 – 300	300 – 340	340 – 380	380 – 420	420 – 470	470 – 525	525 – 615	615 – 775	775 – 950	950 +	all	ST	SC	OBC	others	estd. no. (00)	sample
(1)	(2)	(3)	(4)	(5)	(6)	(7)	(8)	(9)	(10)	(11)	(12)	(13)	(14)	(15)	(16)	(17)	(18)	(19)	(20)	(21)
Gujarat	male	23	3	46	34	68	88	69	65	76	82	78	91	70	51	90	69	76	10922	335
	female	38	25	38	57	61	68	86	51	50	93	101	77	68	61	67	56	91	10408	297
	person	30	13	42	45	64	77	77	58	64	87	90	84	69	56	79	63	84	21330	632
Haryana	male	109	21	87	41	114	26	70	57	76	128	82	91	86	0	105	97	68	6693	271
	female	0	215	47	94	111	119	70	47	72	115	137	167	106	0	102	103	111	7042	237
	person	46	92	64	64	112	64	70	52	74	122	107	124	95	0	104	100	88	13735	508
Himachal Pradesh	male	0	16	88	56	32	61	88	70	59	88	90	74	73	87	66	87	71	1897	284
	female	114	69	53	102	74	63	59	64	65	115	148	198	101	53	91	129	102	2713	371
	person	45	40	68	79	53	62	73	67	62	101	119	134	87	69	79	110	86	4610	655
Jammu & Kashmir	male	119	0	0	52	48	40	49	89	112	55	55	68	70	94	83	85	62	2159	174
	female	0	0	129	1	140	22	50	60	60	85	71	92	69	0	95	34	70	1998	177
	person	90	0	73	29	96	31	50	74	89	69	62	81	70	47	89	61	66	4157	351
Jharkhand	male	26	18	22	28	14	27	25	31	10	42	27	78	24	21	16	23	45	2448	112
	female	15	26	41	40	33	47	54	90	47	29	21	66	43	41	20	50	48	3950	137
	person	20	22	31	33	23	37	38	60	28	36	24	72	33	30	18	36	46	6398	249
Karnataka	male	31	57	38	43	95	67	62	67	61	58	65	146	63	50	65	72	58	10368	373
	female	27	73	48	50	64	61	68	80	68	63	44	163	64	70	54	66	66	10550	334
	person	29	66	43	46	80	64	65	73	64	60	54	154	64	60	60	69	62	20918	707
Kerala	male	390	133	276	290	161	250	168	219	247	245	273	266	242	221	245	242	242	26935	966
	female	229	266	190	217	237	214	158	289	239	257	316	335	266	304	299	258	268	32726	1189
	person	304	202	228	252	203	231	163	257	243	251	296	302	255	264	272	250	255	59661	2155

Continued

For peer review only

For peer review only - http://bmjopen.bmj.com/site/about/guidelines.xhtml

Appendix A

Table (36): Number per 1000 of persons reporting ailment during the last 15 days by mpce class and social group for each sex and State/UT

Rural

state / ut	sex	mpce class													social group				persons reporting ailment*	
		0 – 225	225 – 255	255 – 300	300 – 340	340 – 380	380 – 420	420 – 470	470 – 525	525 – 615	615 – 775	775 – 950	950 +	all	ST	SC	OBC	others	estd. no. (00)	sample
(1)	(2)	(3)	(4)	(5)	(6)	(7)	(8)	(9)	(10)	(11)	(12)	(13)	(14)	(15)	(16)	(17)	(18)	(19)	(20)	(21)
Madhya Pradesh	male	52	56	45	56	52	45	62	62	46	108	65	126	57	43	66	60	60	13403	425
	female	58	41	73	45	63	69	59	64	78	113	107	139	66	70	54	66	74	14328	423
	person	55	49	59	51	58	57	60	63	60	110	84	133	61	56	60	63	66	27731	848
Maharashtra	male	77	92	46	49	57	87	81	89	90	115	110	140	84	53	58	86	105	23624	685
	female	64	67	70	70	74	87	94	104	121	120	192	222	103	99	84	111	103	28651	764
	person	70	79	57	60	65	87	88	96	106	117	150	180	93	76	71	99	104	52275	1449
Manipur	male	0	0	0	50	4	19	12	8	12	56	53	19	27	7	0	44	0	196	73
	female	0	0	0	0	32	8	4	20	4	58	92	38	30	6	0	52	0	215	85
	person	0	0	0	23	17	13	8	13	8	57	72	28	28	6	0	48	0	411	158
Meghalaya	male	250	0	180	0	85	18	45	31	40	88	68	85	51	54	0	0	42	502	76
	female	0	0	42	29	45	12	62	52	30	40	171	146	51	53	0	8	40	468	78
	person	111	0	110	14	64	15	53	42	35	64	115	115	51	54	0	4	41	970	154
Mizoram	male	0	0	0	18	0	0	0	85	36	32	10	12	23	23	0	0	0	46	21
	female	0	0	0	0	0	0	0	0	39	32	0	18	18	18	0	0	0	33	13
	person	0	0	0	10	0	0	0	36	38	32	5	15	20	21	0	0	0	80	34
Nagaland	male	0	0	0	0	0	0	19	0	0	71	48	96	61	57	500	0	0	129	41
	female	0	0	0	0	0	0	0	0	0	44	85	80	63	64	0	0	0	128	33
	person	0	0	0	0	0	0	14	0	0	60	67	88	62	60	333	0	0	257	74
Orissa	male	61	98	63	65	55	108	84	96	88	150	86	199	79	70	87	79	81	11747	388
	female	76	88	66	66	79	67	79	51	87	107	188	148	76	60	115	64	86	11651	373
	person	69	93	64	66	67	88	82	73	88	128	136	175	77	65	101	72	83	23398	761

Continued

For peer review only

For peer review only - http://bmjopen.bmj.com/site/about/guidelines.xhtml

Appendix A

Table (36): Number per 1000 of persons reporting ailment during the last 15 days by mpce class and social group for each sex and State/UT

Rural

state / ut	sex	mpce class													social group				persons reporting ailment*	
		0 – 225	225 – 255	255 – 300	300 – 340	340 – 380	380 – 420	420 – 470	470 – 525	525 – 615	615 – 775	775 – 950	950 +	all	ST	SC	OBC	others	estd. no. (00)	sample
(1)	(2)	(3)	(4)	(5)	(6)	(7)	(8)	(9)	(10)	(11)	(12)	(13)	(14)	(15)	(16)	(17)	(18)	(19)	(20)	(21)
Punjab	male	225	0	21	47	140	46	108	89	28	95	186	155	114	0	119	100	115	9207	293
	female	62	0	51	91	242	73	78	129	162	175	153	228	160	0	157	123	177	11960	354
	person	143	0	38	70	187	60	94	108	89	134	170	190	136	0	138	110	144	21168	647
Rajasthan	male	13	77	58	38	36	75	45	40	45	65	60	122	52	33	49	57	56	10886	475
	female	33	22	59	31	48	36	40	62	82	87	125	115	61	41	74	63	58	11962	446
	person	23	48	58	34	42	55	43	50	62	75	94	119	57	37	60	60	57	22848	921
Sikkim	male	444	0	12	156	15	123	78	45	62	42	136	82	69	72	80	69	15	136	69
	female	0	0	6	35	17	161	85	31	28	5	11	0	40	61	15	28	77	76	55
	person	268	0	9	90	16	145	81	39	45	22	78	48	55	66	46	49	51	212	124
Tamil Nadu	male	82	64	62	74	87	61	60	65	93	127	130	132	86	15	78	92	90	16812	525
	female	90	97	69	87	93	91	117	75	94	123	165	157	103	1	103	106	98	20770	577
	person	86	80	66	81	90	76	90	70	94	125	148	144	95	8	91	100	94	37581	1102
Tripura	male	392	68	71	84	128	123	155	137	170	115	147	104	126	131	140	85	139	1754	251
	female	237	147	60	104	197	154	187	80	150	147	165	54	135	148	151	115	126	1654	250
	person	312	104	66	93	159	138	171	110	162	129	156	82	130	139	145	99	133	3407	501
Uttaranchal	male	0	0	0	27	54	23	22	79	164	65	166	38	54	73	22	69	63	1815	65
	female	0	2	0	43	36	78	2	74	72	31	48	150	49	30	32	44	59	1479	55
	person	0	1	0	34	45	46	13	77	118	48	105	99	52	53	26	58	61	3293	120
Uttar Pradesh	male	69	63	76	96	87	93	104	116	102	106	121	147	96	18	92	89	120	63959	2176
	female	69	61	85	75	105	93	125	116	127	132	144	181	104	69	101	101	116	64737	2192
	person	69	62	81	85	96	93	115	116	114	118	132	163	100	43	96	95	118	128696	4368

Continued

For peer review only - http://bmjopen.bmj.com/site/about/guidelines.xhtml

Table (36): Number per 1000 of persons reporting ailment during the last 15 days by mpce class and social group for each sex and State/UT

Rural

state / ut	sex	mpce class													social group				persons reporting ailment*	
		0 – 225	225 – 255	255 – 300	300 – 340	340 – 380	380 – 420	420 – 470	470 – 525	525 – 615	615 – 775	775 – 950	950 +	all	ST	SC	OBC	others	estd. no. (00)	sample
(1)	(2)	(3)	(4)	(5)	(6)	(7)	(8)	(9)	(10)	(11)	(12)	(13)	(14)	(15)	(16)	(17)	(18)	(19)	(20)	(21)
West Bengal	male	96	55	71	102	106	113	135	122	147	121	142	189	115	60	107	121	124	33754	908
	female	76	57	67	105	99	106	123	121	129	139	225	204	113	53	112	125	120	33046	931
	person	86	56	69	103	102	109	129	121	138	130	180	197	114	56	110	123	122	66800	1839
A & N Islands	male	0	0	0	0	3	186	21	4	114	30	22	48	41	17	0	0	45	52	23
	female	0	0	0	0	0	0	0	0	0	56	23	165	63	22	0	0	69	64	13
	person	0	0	0	0	2	97	6	2	64	39	22	108	51	20	0	0	56	115	36
Chandigarh	male	0	0	0	0	0	0	0	0	0	29	14	42	26	0	41	2	32	13	19
	female	0	0	0	0	186	0	0	0	18	115	29	89	89	250	76	72	94	34	21
	person	0	0	0	0	102	0	0	0	11	57	21	60	52	200	56	17	61	47	40
Dadra & N. Haveli	male	0	0	0	0	0	62	0	0	8	10	6	5	10	10	0	0	17	11	7
	female	0	0	0	0	0	52	10	51	81	0	0	0	21	17	0	0	83	23	6
	person	0	0	0	0	0	58	5	26	47	5	3	4	15	13	0	0	47	35	13
Daman & Diu	male	0	0	0	0	0	0	0	0	39	1	4	2	6	0	0	25	0	4	4
	female	0	0	0	0	0	0	0	0	26	79	84	26	43	0	0	102	0	14	10
	person	0	0	0	0	0	0	0	0	34	20	48	9	19	0	0	62	0	18	14
Lakshadweep	male	0	0	83	0	0	0	56	0	51	100	176	47	81	72	0	0	482	10	19
	female	0	0	0	0	96	280	108	108	39	126	121	292	142	140	0	500	0	18	39
	person	0	0	43	0	39	141	100	57	45	117	149	154	112	108	0	250	390	27	58
Pondicherry	male	1000	0	334	0	218	133	406	188	66	181	88	107	162	0	180	147	267	289	34
	female	0	0	515	19	45	250	102	0	266	112	59	92	132	0	117	141	0	201	24
	person	1000	0	423	13	156	202	238	94	142	159	74	101	149	0	154	144	211	490	58

For peer review only - http://bmjopen.bmj.com/site/about/guidelines.xhtml

Appendix A

Table (36): Number per 1000 of persons reporting ailment during the last 15 days by mpce class and social group for each sex and State/UT

Rural

state / ut	sex	mpce class													social group				persons reporting ailment*	
		0 – 225	225 – 255	255 – 300	300 – 340	340 – 380	380 – 420	420 – 470	470 – 525	525 – 615	615 – 775	775 – 950	950 +	all	ST	SC	OBC	others	estd. no. (00)	sample
(1)	(2)	(3)	(4)	(5)	(6)	(7)	(8)	(9)	(10)	(11)	(12)	(13)	(14)	(15)	(16)	(17)	(18)	(19)	(20)	(21)
ONES	male	181	56	66	73	95	87	85	66	54	76	64	67	73	60	140	60	107	3011	655
	female	132	152	56	79	124	99	100	48	40	65	94	66	73	61	143	67	101	2806	632
	person	157	101	61	76	108	93	92	57	47	71	78	66	73	60	141	63	104	5817	1287
GUTs	male	504	0	114	0	121	111	208	58	56	47	33	47	69	17	165	109	35	378	106
	female	0	0	186	19	61	169	58	22	134	52	32	107	79	27	112	127	72	354	113
	person	337	0	148	13	95	140	126	40	90	49	32	71	73	22	143	117	49	733	219
All India	male	65	57	61	65	72	79	80	88	88	106	123	142	83	52	83	82	98	305036	11287
	female	63	56	66	67	81	81	92	99	100	119	151	189	93	63	93	92	106	326898	11622
	person	64	56	64	66	77	80	86	93	94	112	137	165	88	58	88	87	102	631934	22909

* during the last 15 days

For peer review only - http://bmjopen.bmj.com/site/about/guidelines.xhtml

For peer review only

Appendix A

Table (36): Number per 1000 of persons reporting ailment during the last 15 days by mpce class and social group for each sex and State/UT

Urban

state / ut	sex	mpce class													social group				persons reporting ailment*	
		0 – 300	300 – 350	350 – 425	425 – 500	500 – 575	575 – 665	665 – 775	775 – 915	915 – 1120	1120 – 1500	1500 – 1925	1925 +	all	ST	SC	OBC	others	estd. no. (00)	sample
(1)	(2)	(3)	(4)	(5)	(6)	(7)	(8)	(9)	(10)	(11)	(12)	(13)	(14)	(15)	(16)	(17)	(18)	(19)	(20)	(21)
Andhra Pradesh	male	87	85	89	107	70	117	82	80	77	125	107	148	102	70	84	99	114	10278	499
	female	31	235	114	75	46	159	136	134	120	161	118	189	127	60	88	118	153	12295	653
	person	63	157	101	90	57	137	109	106	99	142	113	166	114	66	86	108	133	22574	1152
Arunachal Pradesh	male	0	0	5	7	42	6	138	37	45	0	12	0	46	68	0	0	25	24	35
	female	68	0	0	44	0	5	145	60	56	12	5	0	57	73	0	333	26	28	38
	person	23	0	2	26	25	5	141	47	50	7	9	0	51	71	0	131	26	52	73
Assam	male	8	13	334	45	125	91	152	60	68	31	56	48	72	19	113	93	67	862	91
	female	0	23	310	86	16	136	148	67	67	136	27	53	95	51	118	89	96	1006	94
	person	4	17	320	65	67	111	150	63	67	79	41	50	83	32	116	91	80	1868	185
Bihar	male	51	120	69	58	23	40	17	65	108	161	22	30	63	10	66	53	83	2497	126
	female	42	365	57	55	35	59	71	35	51	41	33	8	64	61	149	45	57	2134	117
	person	48	250	63	56	28	48	40	50	81	103	25	20	63	22	107	50	70	4631	243
Chhattisgarh	male	70	49	55	60	20	32	81	63	57	76	75	268	63	34	56	58	91	950	81
	female	51	74	52	34	138	16	67	101	167	89	32	139	81	41	64	91	84	1085	89
	person	60	58	54	46	83	25	75	79	109	82	55	208	72	37	60	73	88	2035	170
Delhi	male	0	27	7	11	55	3	9	32	9	14	5	21	15	0	13	23	13	812	58
	female	0	103	0	34	0	39	19	3	2	26	13	22	17	0	19	9	19	763	53
	person	0	68	4	23	24	18	14	19	6	18	8	21	16	0	16	18	16	1575	111
Goa	male	0	0	0	216	57	40	0	94	133	48	0	0	77	0	25	34	83	217	23
	female	0	0	0	92	403	0	45	219	7	58	128	126	115	0	273	213	101	315	28
	person	0	0	0	150	187	23	13	161	71	52	95	68	95	0	159	100	92	532	51

Continued

For peer review only - http://bmjopen.bmj.com/site/about/guidelines.xhtml

For peer review only

Appendix A

Table (36): Number per 1000 of persons reporting ailment during the last 15 days by mpce class and social group for each sex and State/UT

Urban

state / ut	sex	mpce class													social group				persons reporting ailment*	
		0 – 300	300 – 350	350 – 425	425 – 500	500 – 575	575 – 665	665 – 775	775 – 915	915 – 1120	1120 – 1500	1500 – 1925	1925 +	all	ST	SC	OBC	others	estd. no. (00)	sample
(1)	(2)	(3)	(4)	(5)	(6)	(7)	(8)	(9)	(10)	(11)	(12)	(13)	(14)	(15)	(16)	(17)	(18)	(19)	(20)	(21)
Gujarat	male	43	12	15	72	107	45	43	92	69	89	79	153	77	114	38	68	91	6535	299
	female	0	5	8	74	130	109	72	74	75	65	78	126	78	95	77	73	83	5800	306
	person	21	9	12	73	117	77	57	83	72	78	79	141	78	105	57	70	87	12335	605
Haryana	male	178	0	56	18	87	82	22	76	103	144	97	193	86	332	71	98	82	2195	139
	female	0	0	30	43	75	93	48	145	32	186	79	207	89	435	98	48	99	2007	138
	person	146	0	44	30	82	87	35	109	72	163	89	200	87	396	84	76	90	4202	277
Himachal Pradesh	male	0	0	0	30	16	83	12	10	78	89	33	15	39	0	10	80	46	110	38
	female	0	56	255	143	19	115	39	70	59	70	0	213	78	0	44	266	68	231	50
	person	0	26	127	81	17	102	27	41	68	80	15	103	59	0	28	152	58	341	88
Jammu & Kashmir	male	500	158	106	103	254	38	37	45	108	92	59	51	75	275	81	162	69	514	84
	female	0	107	141	80	57	79	66	84	115	73	0	39	81	667	128	75	75	548	82
	person	200	127	122	88	190	57	50	64	112	82	36	45	78	413	103	113	72	1062	166
Jharkhand	male	21	28	33	36	61	66	12	14	43	110	19	68	42	14	7	50	46	855	76
	female	30	42	20	21	90	36	70	67	71	64	28	167	59	60	61	51	66	1113	76
	person	26	36	27	29	75	51	38	41	55	86	23	120	50	35	32	50	56	1968	152
Karnataka	male	64	59	40	61	38	43	52	44	47	67	32	77	53	87	45	60	49	3699	296
	female	54	74	48	39	40	55	83	72	64	40	48	131	61	40	51	56	67	4058	299
	person	59	66	43	50	39	49	68	57	56	54	40	100	57	61	48	58	58	7758	595
Kerala	male	187	179	136	171	222	185	179	223	243	310	299	369	235	0	227	214	279	9126	480
	female	173	169	204	200	152	282	235	199	240	321	302	285	244	149	264	224	277	10520	583
	person	179	174	172	186	184	238	208	211	242	316	301	324	240	79	249	220	278	19647	1063

Continued

For peer review only

For peer review only - http://bmjopen.bmj.com/site/about/guidelines.xhtml

Appendix A

Table (36): Number per 1000 of persons reporting ailment during the last 15 days by mpce class and social group for each sex and State/UT

Urban

state / ut	sex	mpce class													social group				persons reporting ailment*	
		0 – 300	300 – 350	350 – 425	425 – 500	500 – 575	575 – 665	665 – 775	775 – 915	915 – 1120	1120 – 1500	1500 – 1925	1925 +	all	ST	SC	OBC	others	estd. no. (00)	sample
(1)	(2)	(3)	(4)	(5)	(6)	(7)	(8)	(9)	(10)	(11)	(12)	(13)	(14)	(15)	(16)	(17)	(18)	(19)	(20)	(21)
Madhya Pradesh	male	80	57	37	45	42	48	86	62	71	42	90	199	60	69	82	54	58	4583	275
	female	55	70	60	78	68	66	65	66	78	102	204	73	71	63	75	69	73	5006	286
	person	68	64	48	60	54	57	76	64	75	69	144	138	65	66	78	61	65	9589	561
Maharashtra	male	45	129	112	92	73	50	123	100	101	136	163	170	115	78	79	79	141	22832	803
	female	12	45	85	75	18	55	144	127	159	134	126	210	122	61	84	93	146	22026	864
	person	27	91	97	84	44	52	133	113	126	135	144	190	118	69	81	85	143	44858	1667
Manipur	male	0	0	0	0	14	1	43	19	18	50	0	0	20	3	0	24	16	56	33
	female	0	0	4	20	0	10	58	19	64	103	0	0	34	60	0	38	7	95	46
	person	0	0	2	11	7	6	50	19	41	78	0	0	27	28	0	31	11	151	79
Meghalaya	male	0	200	0	104	19	96	11	4	5	32	10	6	20	27	0	99	4	27	22
	female	0	250	0	0	253	4	15	21	31	95	111	258	75	84	109	35	55	114	38
	person	0	231	0	51	150	50	13	13	18	69	58	162	50	58	49	61	30	140	60
Mizoram	male	45	0	0	0	0	6	25	2	9	17	30	58	17	17	0	0	0	23	48
	female	22	0	0	52	0	0	28	10	20	17	29	12	18	18	0	0	0	26	42
	person	33	0	0	27	0	3	26	6	15	17	29	34	17	18	0	0	0	50	90
Nagaland	male	0	0	0	0	0	250	36	47	59	61	36	133	58	66	3	0	78	74	21
	female	0	0	0	0	0	0	0	36	73	65	22	0	46	47	146	0	0	52	13
	person	0	0	0	0	0	167	22	41	64	63	28	68	52	57	57	0	53	125	34
Orissa	male	52	53	82	43	59	41	52	66	77	56	35	29	56	41	43	56	67	1176	98
	female	40	24	20	45	102	13	88	22	120	41	8	36	52	58	49	37	66	1019	79
	person	47	37	49	44	79	30	71	44	98	48	19	32	54	49	46	47	67	2195	177

Continued

For peer review only - http://bmjopen.bmj.com/site/about/guidelines.xhtml

Appendix A

Table (36): Number per 1000 of persons reporting ailment during the last 15 days by mpce class and social group for each sex and State/UT

Urban

state / ut	sex	mpce class													social group				persons reporting ailment*	
		0 – 300	300 – 350	350 – 425	425 – 500	500 – 575	575 – 665	665 – 775	775 – 915	915 – 1120	1120 – 1500	1500 – 1925	1925 +	all	ST	SC	OBC	others	estd. no. (00)	sample
(1)	(2)	(3)	(4)	(5)	(6)	(7)	(8)	(9)	(10)	(11)	(12)	(13)	(14)	(15)	(16)	(17)	(18)	(19)	(20)	(21)
Punjab	male	41	51	92	55	45	77	182	98	120	78	131	139	100	0	101	107	97	4104	192
	female	0	0	36	76	109	72	103	142	162	142	232	171	115	0	82	109	139	4026	216
	person	23	24	62	65	78	75	147	118	135	108	180	153	107	0	92	108	117	8130	408
Rajasthan	male	0	157	59	55	84	74	46	73	97	58	100	37	69	80	71	77	61	3748	259
	female	1	58	85	61	33	56	68	115	61	70	184	117	76	68	84	65	83	3841	261
	person	1	108	72	58	57	66	57	92	81	64	142	76	72	74	77	71	72	7589	520
Sikkim	male	0	0	0	0	0	0	0	22	1	22	0	0	8	13	21	6	0	2	6
	female	0	0	0	0	0	0	5	45	10	18	0	0	18	39	64	3	0	5	7
	person	0	0	0	0	0	0	3	34	5	20	0	0	13	26	37	5	0	7	13
Tamil Nadu	male	91	70	85	69	75	64	114	94	99	111	54	71	87	5	84	89	76	9461	572
	female	73	133	136	106	128	93	111	90	116	116	99	80	106	0	95	111	86	11192	683
	person	82	101	112	87	101	78	113	92	107	114	75	75	96	3	90	100	80	20654	1255
Tripura	male	0	111	18	44	42	59	129	120	61	146	0	30	72	0	69	33	106	156	44
	female	0	0	112	58	124	15	116	113	101	26	10	101	71	17	37	65	98	148	49
	person	0	65	53	51	74	34	123	116	83	83	5	66	72	10	55	48	102	305	93
Uttaranchal	male	30	330	65	31	69	8	13	16	132	90	42	69	65	0	28	19	97	593	50
	female	57	260	70	88	159	21	26	41	54	124	66	66	65	0	77	36	72	510	54
	person	45	296	67	54	103	14	18	29	93	104	56	68	65	0	50	26	85	1103	104
Uttar Pradesh	male	56	89	82	79	101	92	124	106	68	123	109	98	93	67	92	84	104	16435	893
	female	92	91	72	124	138	94	163	118	152	166	134	230	126	165	94	106	162	20021	931
	person	74	90	77	101	119	93	142	112	104	143	121	158	108	113	93	94	131	36455	1824

Continued

For peer review only

For peer review only - http://bmjopen.bmj.com/site/about/guidelines.xhtml

Appendix A

Table (36): Number per 1000 of persons reporting ailment during the last 15 days by mpce class and social group for each sex and State/UT

Urban

state / ut	sex	mpce class													social group				persons reporting ailment*	
		0 – 300	300 – 350	350 – 425	425 – 500	500 – 575	575 – 665	665 – 775	775 – 915	915 – 1120	1120 – 1500	1500 – 1925	1925 +	all	ST	SC	OBC	others	estd. no. (00)	sample
(1)	(2)	(3)	(4)	(5)	(6)	(7)	(8)	(9)	(10)	(11)	(12)	(13)	(14)	(15)	(16)	(17)	(18)	(19)	(20)	(21)
West Bengal	male	109	75	160	141	115	110	124	98	124	157	142	230	137	86	126	92	145	13306	613
	female	74	74	183	121	148	185	165	104	204	204	252	318	178	73	138	111	198	15693	723
	person	89	75	171	131	131	146	143	101	157	180	196	274	157	79	132	101	170	28999	1336
A & N Islands	male	0	0	0	0	0	257	22	5	62	118	27	124	58	0	0	0	58	32	18
	female	0	0	0	0	0	0	77	110	17	27	32	11	61	0	0	0	61	29	21
	person	0	0	0	0	0	166	46	58	41	81	29	62	59	0	0	0	59	61	39
Chandigarh	male	0	0	0	21	0	69	104	86	64	62	17	60	55	0	12	45	63	194	67
	female	25	0	0	17	0	3	4	65	100	58	117	102	89	0	113	54	89	258	78
	person	9	0	0	19	0	31	59	75	83	61	58	81	71	0	54	49	74	452	145
Dadra & N. Haveli	male	0	0	0	0	0	0	0	22	13	0	142	0	18	4	0	0	22	3	5
	female	0	0	0	0	0	0	0	225	0	0	26	0	34	0	0	0	57	4	2
	person	0	0	0	0	0	0	0	99	10	0	88	0	24	2	0	0	34	7	7
Daman & Diu	male	0	0	0	0	0	0	0	27	13	68	0	0	30	0	0	51	31	6	8
	female	0	0	0	0	0	0	0	75	38	57	0	0	41	0	250	55	38	7	7
	person	0	0	0	0	0	0	0	51	25	63	0	0	35	0	125	53	34	13	15
Lakshadweep	male	0	0	0	0	159	35	174	55	185	83	184	683	112	105	0	230	1000	16	30
	female	0	0	0	89	276	192	0	171	167	302	231	333	168	168	0	0	0	24	44
	person	0	0	0	43	221	109	87	111	175	175	205	513	140	137	0	230	1000	40	74
Pondicherry	male	446	148	89	99	160	65	103	283	41	78	171	377	149	95	94	145	219	484	72
	female	631	286	354	171	194	293	157	241	211	153	113	504	225	125	163	239	208	691	97
	person	532	224	220	137	177	156	130	261	124	115	140	427	186	115	124	191	214	1175	169

Continued

For peer review only - http://bmjopen.bmj.com/site/about/guidelines.xhtml

For peer review only

Appendix A

Table (36): Number per 1000 of persons reporting ailment during the last 15 days by mpce class and social group for each sex and State/UT

Urban

state / ut	sex	mpce class													social group				persons reporting ailment*	
		0 – 300	300 – 350	350 – 425	425 – 500	500 – 575	575 – 665	665 – 775	775 – 915	915 – 1120	1120 – 1500	1500 – 1925	1925 +	all	ST	SC	OBC	others	estd. no. (00)	sample
(1)	(2)	(3)	(4)	(5)	(6)	(7)	(8)	(9)	(10)	(11)	(12)	(13)	(14)	(15)	(16)	(17)	(18)	(19)	(20)	(21)
ONES	male	12	116	14	22	25	20	57	31	29	61	16	59	37	34	49	24	55	363	209
	female	13	35	76	39	72	9	60	37	53	54	45	85	48	48	39	41	60	467	233
	person	12	76	40	31	47	15	59	34	41	57	31	72	42	41	45	32	57	830	442
GUTs	male	311	145	63	85	153	67	91	162	51	69	70	102	94	84	36	140	74	735	200
	female	501	284	208	150	186	262	130	178	144	84	107	133	148	120	125	229	92	1013	249
	person	394	221	142	118	169	147	110	170	96	75	87	117	119	106	74	183	82	1748	449
all India	male	68	86	80	77	80	73	91	85	88	111	104	132	91	60	80	84	102	115986	6454
	female	61	92	85	89	90	98	113	102	115	126	127	168	108	63	91	98	125	126691	7147
	person	65	89	83	83	85	85	101	93	100	118	115	149	99	61	86	91	113	242677	13601

* during the last 15 days

For peer review only - http://bmjopen.bmj.com/site/about/guidelines.xhtml

Appendix A

Table (36): Number per 1000 of persons reporting ailment during the last 15 days by mpce class and social group for each sex and State/UT

Rural + Urban

state / ut	sex	mpce class													social group				persons reporting ailment*	
		0 – 235	235 – 265	265 – 320	320 – 365	365 – 410	410 – 460	460 – 520	520 – 605	605 – 730	730 – 980	980 – 1285	1285 +	all	ST	SC	OBC	others	estd. no. (00)	sample
(1)	(2)	(3)	(4)	(5)	(6)	(7)	(8)	(9)	(10)	(11)	(12)	(13)	(14)	(15)	(16)	(17)	(18)	(19)	(20)	(21)
Andhra Pradesh	male	77	54	79	87	81	78	75	95	79	110	173	135	91	67	86	83	114	32624	1314
	female	43	69	83	90	73	97	103	120	99	115	120	211	102	72	80	98	134	36675	1457
	person	59	61	81	89	77	88	89	108	89	112	145	170	96	69	83	90	124	69299	2771
Arunachal Pradesh	male	72	0	1	64	28	55	107	89	47	47	62	45	59	70	194	0	23	272	159
	female	92	190	44	67	37	21	57	35	73	50	90	59	61	69	108	69	30	260	156
	person	82	101	20	65	32	38	83	64	59	48	74	52	60	70	162	34	26	532	315
Assam	male	117	51	146	69	68	91	99	62	56	69	84	69	78	66	69	90	78	9860	542
	female	193	30	135	73	104	92	87	73	71	83	48	129	87	66	90	85	93	9675	527
	person	156	40	140	71	86	91	94	67	63	76	67	97	82	66	79	87	85	19535	1069
Bihar	male	43	36	45	39	59	63	47	59	93	72	79	54	54	45	55	54	51	19394	691
	female	27	42	38	49	52	63	78	84	61	64	74	56	54	53	58	53	54	17957	653
	person	36	39	42	44	55	63	62	71	78	68	77	55	54	49	57	54	52	37351	1344
Chhattisgarh	male	96	55	68	51	54	59	88	64	65	88	15	125	68	57	89	71	69	6928	303
	female	68	67	63	38	71	63	87	142	112	147	71	61	71	43	95	87	70	7165	312
	person	82	61	66	44	63	61	88	100	87	115	41	94	70	50	92	79	69	14094	615
Delhi	male	0	27	7	11	55	3	9	28	8	14	4	15	13	0	13	16	12	846	59
	female	0	103	0	34	0	38	19	3	1	20	15	15	15	0	19	6	18	798	55
	person	0	68	4	23	24	18	14	16	6	16	8	15	14	0	16	12	15	1644	114
Goa	male	0	0	0	216	57	36	0	106	127	20	142	200	117	823	25	153	109	982	40
	female	0	0	0	92	403	0	91	148	80	90	81	171	131	478	273	303	118	1094	50
	person	0	0	0	150	187	21	42	126	102	50	107	184	124	625	159	216	113	2076	90

Continued

For peer review only - http://bmjopen.bmj.com/site/about/guidelines.xhtml

Appendix A

Table (36): Number per 1000 of persons reporting ailment during the last 15 days by mpce class and social group for each sex and State/UT

Rural + Urban

state / ut	sex	mpce class													social group				persons reporting ailment*	
		0 – 235	235 – 265	265 – 320	320 – 365	365 – 410	410 – 460	460 – 520	520 – 605	605 – 730	730 – 980	980 – 1285	1285 +	all	ST	SC	OBC	others	estd. no. (00)	sample
(1)	(2)	(3)	(4)	(5)	(6)	(7)	(8)	(9)	(10)	(11)	(12)	(13)	(14)	(15)	(16)	(17)	(18)	(19)	(20)	(21)
Gujarat	male	25	5	42	46	75	76	56	74	73	85	79	110	72	54	71	69	84	17457	634
	female	34	22	34	63	72	78	79	59	60	81	96	91	71	63	71	61	87	16208	603
	person	29	13	38	54	74	77	68	67	67	83	87	101	72	59	71	65	85	33665	1237
Haryana	male	145	21	67	28	104	38	49	63	85	130	83	101	86	241	97	97	72	8888	410
	female	0	212	38	63	99	112	60	78	60	126	132	172	102	371	101	92	108	9049	375
	person	82	91	52	44	102	69	54	70	73	128	105	132	93	317	99	95	89	17937	785
Himachal Pradesh	male	0	14	86	52	30	63	79	61	60	88	87	69	70	85	60	86	68	2007	322
	female	36	67	56	108	68	70	57	65	65	110	137	199	99	52	85	136	98	2944	421
	person	20	38	69	80	49	67	67	63	63	99	112	131	84	68	73	113	83	4951	743
Jammu & Kashmir	male	127	157	30	65	69	40	46	77	112	61	55	67	71	96	83	90	64	2673	258
	female	0	107	132	36	136	32	54	66	68	83	64	89	72	5	99	38	71	2546	259
	person	94	127	85	50	103	36	50	72	92	71	59	78	71	51	91	65	67	5219	517
Jharkhand	male	25	19	23	29	17	31	23	26	20	65	25	71	27	21	16	27	45	3304	188
	female	17	27	39	36	37	46	56	84	53	42	22	137	45	42	23	50	56	5062	213
	person	21	23	31	33	26	38	38	55	35	54	24	105	36	31	19	38	51	8366	401
Karnataka	male	41	58	38	50	84	60	60	60	57	62	55	110	60	56	61	69	54	14067	669
	female	33	73	48	46	60	60	72	77	67	54	45	148	63	65	53	63	67	14609	633
	person	37	66	43	48	72	60	66	68	62	58	50	127	62	61	57	66	60	28676	1302
Kerala	male	267	154	214	213	186	225	171	220	246	258	278	280	240	190	242	234	252	36061	1446
	female	194	221	196	206	203	241	184	269	239	270	314	328	261	282	292	249	271	43247	1772
	person	228	189	204	210	195	234	178	246	243	264	297	305	251	238	268	242	261	79308	3218

Continued

For peer review only

For peer review only - http://bmjopen.bmj.com/site/about/guidelines.xhtml

Table (36): Number per 1000 of persons reporting ailment during the last 15 days by mpce class and social group for each sex and State/UT

Rural + Urban

state / ut	sex	mpce class													social group				persons reporting ailment*	
		0 – 235	235 – 265	265 – 320	320 – 365	365 – 410	410 – 460	460 – 520	520 – 605	605 – 730	730 – 980	980 – 1285	1285 +	all	ST	SC	OBC	others	estd. no. (00)	sample
(1)	(2)	(3)	(4)	(5)	(6)	(7)	(8)	(9)	(10)	(11)	(12)	(13)	(14)	(15)	(16)	(17)	(18)	(19)	(20)	(21)
Madhya Pradesh	male	56	56	43	52	49	46	67	62	52	92	68	158	58	44	69	58	59	17985	700
	female	58	47	69	56	64	68	60	65	78	110	121	110	67	70	58	67	73	19334	709
	person	57	52	56	54	57	57	64	63	64	100	92	135	62	56	64	63	66	37320	1409
Maharashtra	male	69	106	63	68	60	76	98	94	96	126	129	159	97	56	67	84	125	46456	1488
	female	49	60	75	72	62	78	112	113	138	127	167	214	110	95	84	106	126	50677	1628
	person	58	83	69	70	61	77	105	103	116	126	148	186	103	75	75	95	126	97133	3116
Manipur	male	0	0	0	7	11	6	27	10	13	55	51	18	25	7	0	37	12	252	106
	female	0	0	3	17	10	10	31	20	10	62	88	38	31	9	0	47	6	310	131
	person	0	0	2	13	10	8	29	15	11	59	69	28	28	8	0	42	9	562	237
Meghalaya	male	250	200	172	2	77	30	42	27	37	81	51	76	48	52	0	4	29	529	98
	female	0	250	39	29	74	11	56	48	30	49	153	166	54	57	35	9	46	582	116
	person	111	231	104	15	75	21	49	37	34	64	99	122	51	54	14	7	37	1111	214
Mizoram	male	23	0	0	7	0	3	17	29	24	25	14	19	20	21	0	0	0	70	69
	female	11	0	0	36	0	0	21	6	30	26	5	17	18	18	0	0	0	59	55
	person	16	0	0	21	0	2	19	17	27	25	9	18	19	20	0	0	0	129	124
Nagaland	male	0	0	0	0	0	69	34	21	44	66	46	101	60	60	126	0	78	202	62
	female	0	0	0	0	0	0	0	17	44	56	71	71	57	58	115	0	0	180	46
	person	0	0	0	0	0	43	21	19	44	61	60	86	58	59	122	0	53	382	108
Orissa	male	61	96	64	61	55	100	78	92	85	111	73	152	76	68	81	77	78	12923	486
	female	75	84	63	64	81	63	81	47	97	82	125	118	73	60	106	61	81	12670	452
	person	68	90	63	62	68	82	79	70	91	96	100	136	75	64	94	69	80	25592	938

Continued

For peer review only - http://bmjopen.bmj.com/site/about/guidelines.xhtml

Appendix A

Table (36): Number per 1000 of persons reporting ailment during the last 15 days by mpce class and social group for each sex and State/UT

Rural + Urban

state / ut	sex	mpce class													social group				persons reporting ailment*	
		0 – 235	235 – 265	265 – 320	320 – 365	365 – 410	410 – 460	460 – 520	520 – 605	605 – 730	730 – 980	980 – 1285	1285 +	all	ST	SC	OBC	others	estd. no. (00)	sample
(1)	(2)	(3)	(4)	(5)	(6)	(7)	(8)	(9)	(10)	(11)	(12)	(13)	(14)	(15)	(16)	(17)	(18)	(19)	(20)	(21)
Punjab	male	104	30	59	52	97	58	148	93	68	88	182	153	109	0	113	102	109	13311	485
	female	25	0	44	81	175	73	91	134	162	163	159	223	146	0	136	119	163	15986	570
	person	67	15	51	67	135	66	122	112	107	124	171	186	127	0	124	110	134	29297	1055
Rajasthan	male	12	90	58	44	40	75	45	47	58	64	67	107	56	36	52	61	58	14634	734
	female	29	27	63	40	46	40	46	73	77	83	134	116	64	43	76	64	67	15803	707
	person	21	57	61	42	43	58	46	59	67	73	102	111	60	39	63	62	62	30437	1441
Sikkim	male	444	0	12	144	15	115	73	42	47	39	112	73	61	68	59	65	4	139	75
	female	0	0	6	33	17	149	78	33	24	7	8	0	37	59	26	26	29	81	62
	person	268	0	9	85	16	135	75	38	36	21	62	44	49	63	43	46	16	220	137
Tamil Nadu	male	85	65	69	72	83	62	82	74	95	121	106	103	86	14	79	91	81	26273	1097
	female	84	102	89	96	102	92	115	80	101	121	146	121	104	1	102	108	90	31962	1260
	person	84	83	80	84	93	77	99	77	98	121	126	111	95	8	91	100	85	58235	2357
Tripura	male	370	71	63	75	117	119	152	135	153	121	115	94	118	127	127	77	134	1910	295
	female	218	138	66	92	190	141	179	84	138	118	132	62	126	144	131	108	120	1802	299
	person	291	101	64	83	149	130	166	111	146	119	123	80	122	135	129	91	127	3712	594
Uttaranchal	male	30	5	19	28	55	21	21	72	152	73	107	49	57	67	23	56	71	2408	115
	female	57	6	18	51	37	67	5	71	65	58	58	131	53	28	39	42	62	1989	109
	person	45	5	19	37	46	41	14	71	109	66	80	90	55	49	30	50	67	4397	224
Uttar Pradesh	male	67	67	78	90	89	93	108	115	95	109	119	132	95	28	92	88	114	80394	3069
	female	74	67	82	91	111	93	133	117	131	138	143	196	109	87	100	102	131	84758	3123
	person	70	67	80	91	100	93	120	116	112	123	130	161	102	57	96	95	122	165152	6192

Continued

For peer review only - http://bmjopen.bmj.com/site/about/guidelines.xhtml

Appendix A

Table (36): Number per 1000 of persons reporting ailment during the last 15 days by mpce class and social group for each sex and State/UT

Rural + Urban

state / ut	sex	mpce class													social group				persons reporting ailment*	
		0 – 235	235 – 265	265 – 320	320 – 365	365 – 410	410 – 460	460 – 520	520 – 605	605 – 730	730 – 980	980 – 1285	1285 +	all	ST	SC	OBC	others	estd. no. (00)	sample
(1)	(2)	(3)	(4)	(5)	(6)	(7)	(8)	(9)	(10)	(11)	(12)	(13)	(14)	(15)	(16)	(17)	(18)	(19)	(20)	(21)
West Bengal	male	99	58	82	113	107	112	132	117	139	134	142	206	120	61	111	114	130	47060	1521
	female	75	60	80	109	106	120	132	118	150	162	233	253	128	54	118	122	141	48739	1654
	person	87	59	81	111	107	116	132	118	144	147	184	230	124	57	114	118	135	95799	3175
A & N Islands	male	0	0	0	0	3	195	22	5	90	56	22	59	46	17	0	0	49	84	41
	female	0	0	0	0	0	0	40	77	8	46	23	140	62	22	0	0	66	93	34
	person	0	0	0	0	2	104	32	43	53	52	23	101	53	20	0	0	57	177	75
Chandigarh	male	0	0	0	21	0	35	102	67	59	60	16	58	51	0	16	22	60	207	86
	female	25	0	0	17	186	2	3	56	91	63	102	101	89	6	108	58	89	292	99
	person	9	0	0	19	102	19	57	62	76	61	52	79	68	5	54	35	73	499	185
Dadra & N. Haveli	male	0	0	0	0	0	62	0	3	10	9	17	5	11	10	0	0	20	14	12
	female	0	0	0	0	0	51	10	66	74	0	1	0	22	16	0	0	72	27	8
	person	0	0	0	0	0	58	5	34	40	4	8	4	16	13	0	0	41	41	20
Daman & Diu	male	0	0	0	0	0	0	0	20	28	19	3	2	12	0	0	28	8	10	12
	female	0	0	0	0	0	0	0	46	32	68	67	25	42	0	88	93	25	22	17
	person	0	0	0	0	0	0	0	34	29	34	36	9	23	0	44	61	12	32	29
Lakshadweep	male	0	0	83	0	94	30	167	49	135	90	176	75	98	91	0	173	590	25	49
	female	0	0	0	89	222	207	28	163	125	186	129	294	156	155	0	500	0	42	83
	person	0	0	43	43	156	114	89	105	130	143	153	171	127	124	0	238	498	67	132
Pondicherry	male	511	148	187	81	187	83	185	262	53	118	142	218	154	95	161	146	222	773	106
	female	631	286	419	126	143	275	139	192	231	143	97	248	194	125	128	210	202	892	121
	person	563	224	302	106	168	171	161	226	132	129	119	230	173	115	147	177	214	1665	227

Continued

For peer review only - http://bmjopen.bmj.com/site/about/guidelines.xhtml

Table (36): Number per 1000 of persons reporting ailment during the last 15 days by mpce class and social group for each sex and State/UT

Rural + Urban

state / ut	sex	mpce class													social group				persons reporting ailment*	
		0 – 235	235 – 265	265 – 320	320 – 365	365 – 410	410 – 460	460 – 520	520 – 605	605 – 730	730 – 980	980 – 1285	1285 +	all	ST	SC	OBC	others	estd. no. (00)	sample
(1)	(2)	(3)	(4)	(5)	(6)	(7)	(8)	(9)	(10)	(11)	(12)	(13)	(14)	(15)	(16)	(17)	(18)	(19)	(20)	(21)
ONES	male	174	60	57	58	81	72	78	59	49	73	56	66	66	56	118	51	94	3374	864
	female	126	144	58	66	114	77	90	46	42	63	86	68	68	59	120	60	89	3274	865
	person	150	99	58	62	96	74	84	53	46	68	70	67	67	58	119	55	92	6647	1729
GUTs	male	341	123	93	72	134	85	133	129	53	61	51	79	83	29	105	129	61	1114	306
	female	448	257	196	115	119	214	100	127	140	71	67	124	121	50	118	196	86	1368	362
	person	386	194	145	96	127	144	116	128	93	65	58	100	101	40	110	161	72	2481	668
all India	male	66	62	65	69	74	77	83	87	88	107	119	139	85	53	83	82	99	421022	17741
	female	62	61	70	74	82	84	98	100	104	121	145	181	97	63	93	93	113	453589	18769
	person	64	62	67	71	78	81	90	93	96	114	132	159	91	58	88	88	106	874611	36510

* during the last 15 days

For peer review only - http://bmjopen.bmj.com/site/about/guidelines.xhtml

Appendix A

able (37): Number per 1000 of persons suffering from ailment on the day before the date of survey by sex and age-group

ural

state / ut	male						female						person						persons reporting ailment on previous day		total persons	
	age group (*years*)						age group (*years*)						age group (*years*)									
	0 – 14	15 - 29	30 - 44	45 - 59	60 & above	all	0 - 14	15 - 29	30 - 44	45 - 59	60 & above	all	0 - 14	15 - 29	30 - 44	45 - 59	60 & above	all	estd. no. (00)	sam-ple	estd. no. (00)	sam-ple
)	(2)	(3)	(4)	(5)	(6)	(7)	(8)	(9)	(10)	(11)	(12)	(13)	(14)	(15)	(16)	(17)	(18)	(19)	(20)	(21)	(22)	(23)
ndhra radesh	38	17	40	98	292	60	31	27	57	109	315	68	35	22	48	103	304	64	33260	1265	520883	14285
runachal radesh	17	10	8	11	74	16	17	19	0	25	74	18	17	15	5	18	74	17	136	94	7871	4048
ssam	34	12	18	30	229	35	33	14	25	32	270	37	34	13	21	31	246	36	7744	440	215464	11828
ihar	23	8	8	25	123	24	27	11	22	34	114	28	25	10	15	30	119	26	15905	679	617042	20141
hhattisgarh	24	19	35	66	110	34	28	28	54	43	107	41	26	24	44	54	108	38	6585	277	174063	5868
elhi	0	0	0	0	0	0	0	0	0	0	0	0	0	0	0	0	0	0	0	0	18376	450
oa	150	0	93	50	598	115	44	4	95	60	591	113	92	2	94	54	594	114	1273	30	11210	371
ujarat	29	20	30	43	283	46	17	29	25	87	198	43	23	24	27	65	241	45	13777	455	309444	8043
aryana	36	22	48	80	188	50	37	50	96	68	181	68	36	35	71	75	184	58	8402	340	144027	4905
imachal radesh	15	23	34	86	236	51	15	28	99	152	236	75	15	26	67	121	236	63	3343	494	52882	6274
ammu & ashmir	22	7	26	52	276	41	30	10	36	58	350	47	26	9	31	55	307	44	2611	234	59682	4785
harkhand	4	0	6	9	15	5	4	9	6	20	15	8	4	4	6	15	15	6	1199	71	195210	7581
arnataka	13	10	17	70	304	42	9	11	28	66	229	36	11	11	23	68	268	39	12875	511	327791	9423
erala	112	62	142	263	486	170	80	87	165	329	518	192	97	76	154	297	504	182	42544	1562	234249	8683
adhya radesh	20	17	26	39	173	32	13	25	50	61	150	37	17	21	37	49	162	35	15648	527	452556	12942
aharashtra	30	16	26	58	252	48	33	25	45	81	259	60	32	21	36	70	256	54	30280	903	559949	13639
anipur	20	3	9	9	54	13	12	8	13	8	55	12	16	5	11	9	55	13	186	80	14473	5796
eghalaya	50	23	1	3	156	34	16	17	10	25	172	23	34	19	5	14	164	29	555	89	19056	2881
izoram	17	13	3	12	75	16	26	4	0	0	52	12	22	8	1	6	67	14	55	26	3894	2152
agaland	17	0	0	0	100	11	6	0	0	22	50	6	12	0	0	11	87	9	36	21	4125	1220
rissa	22	17	29	49	105	33	19	19	29	65	109	34	20	18	29	57	107	34	10166	378	302260	10356
unjab	46	24	79	98	240	70	37	60	143	227	370	116	42	41	111	158	302	92	14306	464	155189	4603
ajasthan	21	19	36	68	127	34	14	26	64	74	108	39	17	23	50	71	117	37	14802	709	403824	13432
ikkim	42	28	35	80	189	50	22	1	28	9	120	21	32	14	32	50	156	36	138	92	3859	2178
amil Nadu	26	14	35	94	258	55	21	28	82	72	241	63	24	22	59	82	249	59	23601	756	396832	10388
ripura	74	17	34	45	120	48	51	11	32	60	157	42	64	14	33	53	135	45	1180	179	26222	3821

Continued

For peer review only

For peer review only - http://bmjopen.bmj.com/site/about/guidelines.xhtml

Appendix A

'able (37): Number per 1000 of persons suffering from ailment on the day before the date of survey by sex and age-group

ural

state / ut	male						female						person						persons reporting ailment on previous day		total persons	
	age group (*years*)						age group (*years*)						age group (*years*)									
	0 – 14	15 - 29	30 - 44	45 - 59	60 & above	all	0 - 14	15 - 29	30 - 44	45 - 59	60 & above	all	0 - 14	15 - 29	30 - 44	45 - 59	60 & above	all	estd. no. (00)	sam-ple	estd. no. (00)	sam-ple
1)	(2)	(3)	(4)	(5)	(6)	(7)	(8)	(9)	(10)	(11)	(12)	(13)	(14)	(15)	(16)	(17)	(18)	(19)	(20)	(21)	(22)	(23)
ttaranchal	17	38	11	22	91	27	1	32	45	19	68	25	10	35	28	21	80	26	1628	77	63340	1708
ttar Pradesh	42	28	61	86	220	58	34	49	84	107	204	65	38	38	72	96	212	61	78714	2892	1289072	41352
Vest Bengal	53	36	66	110	263	72	49	37	68	102	299	73	51	37	67	106	281	72	42258	1263	585873	16203
. & N lands	10	0	23	62	250	32	48	23	16	87	119	42	26	12	20	72	202	36	82	26	2270	711
handigarh	1	4	13	15	160	9	96	4	36	58	127	56	49	4	22	33	143	28	26	28	901	370
adra & N. laveli	0	0	0	0	13	1	22	16	0	0	95	15	11	8	0	0	39	8	18	5	2281	430
aman & iu	0	1	0	0	0	1	34	0	0	263	71	24	16	1	0	127	54	9	9	7	992	341
akshadweep	12	31	49	54	380	55	59	12	49	330	610	113	37	21	49	186	504	85	20	47	241	448
ondicherry	128	67	55	34	532	120	101	0	44	58	724	114	117	38	50	47	624	117	386	45	3299	322
NES	45	14	16	24	111	32	25	11	16	34	127	25	35	13	16	29	118	29	2285	581	79499	22096
UTs	45	20	26	29	325	48	60	10	26	64	470	61	52	16	26	46	388	54	541	158	9985	2622
ll-India	33	21	40	76	224	50	28	31	60	91	229	57	31	26	50	83	226	53	383746	15066	7178701	251978
std. persons eptg. ilment on ne previous ay (00)	45352	18740	27921	32099	58419	182531	35485	28146	41340	38077	58167	201215	80837	46886	69260	70177	116586	383746	X	X	X	X
ample per- ons reptg. ilment on ne previous ay	1505	837	1050	1271	2757	7420	1125	1004	1417	1543	2557	7646	2630	1841	2467	2814	5314	15066	X	X	X	X
std. no. of ersons (00)	1373037	908479	690923	424407	260664	3657509	1256497	900801	689161	420608	254124	3521192	2629534	1809280	1380084	845015	514787	7178701	X	X	X	X
ample ersons	45943	33016	23467	14369	11834	128629	41673	32360	22896	15486	10934	123349	87616	65376	46363	29855	22768	251978	X	X	X	X

For peer review only

For peer review only - http://bmjopen.bmj.com/site/about/guidelines.xhtml

Appendix A

able (37): Number per 1000 of persons suffering from ailment on the day before the date of survey by sex and age-group

rban

state / ut	male						female						person						persons reporting ailment on previous day		total persons	
	age group (*years*)						age group (*years*)						age group (*years*)									
	0 – 14	15 - 29	30 - 44	45 - 59	60 & above	all	0 - 14	15 - 29	30 - 44	45 - 59	60 & above	all	0 - 14	15 - 29	30 - 44	45 - 59	60 & above	all	estd. no. (00)	sam-ple	estd. no. (00)	sam-ple
)	(2)	(3)	(4)	(5)	(6)	(7)	(8)	(9)	(10)	(11)	(12)	(13)	(14)	(15)	(16)	(17)	(18)	(19)	(20)	(21)	(22)	(23)
ndhra radesh	39	19	52	114	460	67	34	33	39	176	496	82	37	26	46	145	480	74	14700	898	197699	8195
runachal radesh	61	31	11	3	29	35	61	20	54	0	43	45	61	25	31	2	35	40	40	37	1016	1572
ssam	30	14	26	54	237	39	20	23	39	38	242	39	26	18	32	47	239	39	886	90	22636	2322
ihar	10	24	17	106	177	38	6	7	36	45	189	28	8	16	26	77	182	33	2425	155	73097	3800
hhattisgarh	19	1	15	105	135	30	11	10	47	136	140	42	15	5	31	119	138	36	1017	97	28419	2077
elhi	2	3	5	4	115	8	1	2	8	3	104	8	2	3	6	3	110	8	796	72	99289	4761
oa	27	3	94	11	113	36	13	36	18	102	369	71	21	18	53	55	252	53	296	35	5575	535
ujarat	33	20	45	83	351	56	31	23	62	109	260	62	32	21	53	95	303	59	9327	497	158711	6594
aryana	15	37	40	107	244	50	19	36	87	105	239	65	17	36	62	106	241	57	2746	210	48126	2904
imachal radesh	38	2	8	52	304	35	29	32	78	74	249	57	33	17	43	63	275	46	265	70	5752	904
ammu & ashmir	47	23	16	102	281	56	29	17	28	133	297	53	37	20	22	116	288	55	742	137	13578	2014
harkhand	6	1	6	16	55	9	4	14	14	39	75	19	5	7	10	27	65	14	540	51	39035	3410
arnataka	15	16	15	65	276	37	16	7	41	66	349	47	16	12	28	65	313	42	5718	456	136791	7624
erala	129	52	79	228	487	153	115	84	104	261	534	178	122	68	92	245	513	166	13622	786	81932	4720
adhya radesh	37	12	23	49	194	36	31	19	35	92	182	45	34	15	29	69	188	40	5916	382	146839	7141
aharashtra	57	33	43	136	383	79	32	22	78	161	408	85	45	28	60	148	396	82	31014	1231	378789	13048
anipur	24	1	8	2	39	12	18	9	25	14	67	20	21	5	18	8	53	16	87	50	5611	2845
eghalaya	7	2	3	6	34	5	37	29	8	79	137	37	23	18	6	40	94	22	63	33	2825	1202
izoram	5	3	2	1	65	7	17	4	5	8	85	12	11	4	3	5	73	10	27	56	2847	3247
agaland	30	0	0	7	32	15	63	0	0	0	0	22	44	0	0	4	29	18	44	13	2415	595
rissa	21	4	32	53	129	30	35	5	13	65	132	30	28	4	24	59	130	30	1221	103	40544	2726
unjab	42	47	69	91	243	68	35	19	78	173	317	75	39	34	73	129	278	71	5454	288	76302	3458
ajasthan	43	9	46	71	265	50	31	31	96	64	216	60	38	20	72	68	240	55	5738	432	104919	5922
ikkim	4	14	0	0	29	8	16	14	0	33	262	18	10	14	0	10	122	13	7	13	580	380
amil Nadu	22	15	35	114	288	56	24	17	37	102	332	63	23	16	36	108	311	60	12840	865	215244	10991
ripura	38	18	16	26	100	29	20	32	30	3	54	26	30	25	23	14	76	28	117	43	4253	1023

Continued

For peer review only

For peer review only - http://bmjopen.bmj.com/site/about/guidelines.xhtml

Appendix A

able (37): Number per 1000 of persons suffering from ailment on the day before the date of survey by sex and age-group

rban

state / ut	male						female						person						persons reporting ailment on previous day		total persons	
	age group (*years*)						age group (*years*)						age group (*years*)									
	0 – 14	15 - 29	30 - 44	45 - 59	60 & above	all	0 - 14	15 - 29	30 - 44	45 - 59	60 & above	all	0 - 14	15 - 29	30 - 44	45 - 59	60 & above	all	estd. no. (00)	sam-ple	estd. no. (00)	sam-ple
1)	(2)	(3)	(4)	(5)	(6)	(7)	(8)	(9)	(10)	(11)	(12)	(13)	(14)	(15)	(16)	(17)	(18)	(19)	(20)	(21)	(22)	(23)
ttaranchal	52	3	19	40	314	46	17	27	61	111	145	54	38	15	39	77	221	50	847	81	17021	946
ttar Pradesh	34	34	46	113	247	57	33	48	98	181	300	82	34	40	72	143	274	69	23132	1235	336625	15162
Vest Bengal	74	36	65	96	421	98	56	57	99	212	498	132	65	47	81	150	458	114	21129	1009	185135	8837
& N slands	5	0	11	103	193	22	12	18	14	36	101	19	8	9	12	73	152	21	21	27	1034	545
handigarh	17	9	14	54	144	28	31	55	37	62	265	60	24	29	24	58	198	42	269	107	6402	1447
adra & N. laveli	30	9	0	0	9	13	0	0	0	0	86	3	18	6	0	0	45	9	2	4	269	355
aman & iu	0	0	0	165	201	27	13	14	4	0	55	13	6	5	2	90	111	21	8	12	384	389
akshadweep	59	0	70	137	451	77	30	49	51	231	487	96	45	25	59	180	471	87	25	47	286	520
ondicherry	53	41	30	226	579	109	88	43	91	364	680	190	69	42	57	292	641	148	936	137	6321	866
NES	25	7	7	9	56	15	31	17	19	17	76	24	28	13	13	13	65	20	386	245	19546	10864
UTs	34	19	20	128	362	61	54	45	57	189	524	114	43	31	36	156	452	86	1261	334	14695	4122
ll-India	38	24	41	100	312	60	32	28	63	134	342	73	35	26	52	116	327	66	162021	9759	2446300	133077
std. persons eptg. ilment on ne previous ay (00)	14395	8878	11066	16712	25166	76217	10727	9542	16419	20321	28795	85803	25122	18419	27485	37033	53961	162021	X	X	X	X
ample per-ons reptg. ilment on ne previous ay	760	464	605	908	1850	4587	586	575	811	1141	2059	5172	1346	1039	1416	2049	3909	9759	X	X	X	X
std. no. of ersons (00)	380698	376633	271398	167236	80686	1276650	339616	335668	259117	151140	84109	1169650	720314	712301	530515	318376	164795	2446300	X	X	X	X
ample ersons	20212	19004	14366	8134	6354	68070	18332	18629	12974	8625	6447	65007	38544	37633	27340	16759	12801	133077	X	X	X	X

For peer review only - http://bmjopen.bmj.com/site/about/guidelines.xhtml

Appendix A

able (37): Number per 1000 of persons suffering from ailment on the day before the date of survey by sex and age-group

ural + Urban

state / ut	male						female						person						persons reporting ailment on previous day		total persons	
	age group (*years*)						age group (*years*)						age group (*years*)									
	0 – 14	15 - 29	30 - 44	45 - 59	60 & above	all	0 - 14	15 - 29	30 - 44	45 - 59	60 & above	all	0 - 14	15 - 29	30 - 44	45 - 59	60 & above	all	estd. no. (00)	sam-ple	estd. no. (00)	sam-ple
)	(2)	(3)	(4)	(5)	(6)	(7)	(8)	(9)	(10)	(11)	(12)	(13)	(14)	(15)	(16)	(17)	(18)	(19)	(20)	(21)	(22)	(23)
ndhra radesh	38	18	43	102	329	62	32	29	52	125	358	72	35	23	48	113	344	67	47961	2163	718581	22480
runachal radesh	22	12	9	11	72	19	22	19	8	23	73	21	22	16	9	16	73	20	176	131	8886	5620
ssam	34	12	19	32	230	36	32	15	27	33	267	37	33	14	23	33	245	36	8630	530	238101	14150
ihar	22	10	9	36	129	25	26	11	23	35	122	28	24	11	16	36	126	27	18330	834	690139	23941
hhattisgarh	23	16	32	73	113	34	26	25	53	56	111	41	24	20	42	65	112	38	7602	374	202482	7945
elhi	2	3	4	3	100	7	0	2	7	3	91	7	1	2	6	3	95	7	796	72	117665	5211
oa	103	1	93	36	447	88	34	17	75	76	523	99	67	8	83	55	488	93	1569	65	16785	906
ujarat	30	20	36	57	303	49	21	27	38	94	219	49	26	23	37	75	261	49	23104	952	468155	14637
aryana	31	26	46	88	198	50	33	47	94	78	195	67	32	36	69	83	196	58	11147	550	192154	7809
imachal radesh	17	21	31	84	240	49	17	28	97	148	237	73	17	25	64	118	238	62	3608	564	58634	7178
ammu & ashmir	26	11	24	63	277	44	30	12	34	74	341	48	28	11	29	68	304	46	3353	371	73260	6799
harkhand	4	0	6	11	22	5	4	10	7	24	29	10	4	5	7	17	25	7	1739	122	234245	10991
arnataka	14	12	17	69	297	41	11	10	32	66	262	39	12	11	24	67	280	40	18593	967	464582	17047
erala	116	59	125	253	486	166	89	86	149	309	522	188	103	74	138	282	506	178	56166	2348	316181	13403
adhya radesh	24	16	25	41	178	33	17	24	46	69	158	39	20	20	35	54	168	36	21564	909	599395	20083
aharashtra	40	24	33	93	299	61	33	24	59	113	312	70	36	24	46	103	306	65	61294	2134	938738	26687
anipur	21	2	9	7	49	13	14	8	16	9	60	14	17	5	13	8	54	14	273	130	20084	8641
eghalaya	46	19	1	3	146	31	18	19	10	35	168	25	33	19	5	18	157	28	618	122	21881	4083
izoram	12	9	2	7	71	12	22	4	2	4	67	12	17	6	2	5	69	12	82	82	6741	5399
agaland	22	0	0	2	74	13	26	0	0	15	42	12	24	0	0	8	67	12	81	34	6540	1815
rissa	22	15	29	49	108	33	20	17	27	65	111	34	21	16	28	58	109	33	11387	481	342804	13082
unjab	45	32	76	95	241	69	37	46	122	208	356	103	41	39	98	148	296	85	19761	752	231492	8061
ajasthan	24	17	39	69	155	38	17	27	71	72	128	43	21	22	55	70	141	40	20540	1141	508743	19354
ikkim	38	26	32	69	176	44	21	3	23	11	129	20	29	14	27	45	153	33	145	105	4438	2558
amil Nadu	24	14	35	101	268	56	22	25	66	82	272	63	23	20	51	91	270	60	36441	1621	612076	21379
ripura	70	17	31	42	117	45	48	15	32	52	140	39	60	16	32	47	127	43	1297	222	30475	4844

Continued

For peer review only

For peer review only - http://bmjopen.bmj.com/site/about/guidelines.xhtml

Appendix A

able (37): Number per 1000 of persons suffering from ailment on the day before the date of survey by sex and age-group

ural + Urban

state / ut	male						female						person						persons reporting ailment on previous day		total persons	
	age group (*years*)						age group (*years*)						age group (*years*)									
	0 – 14	15 - 29	30 - 44	45 - 59	60 & above	all	0 - 14	15 - 29	30 - 44	45 - 59	60 & above	all	0 - 14	15 - 29	30 - 44	45 - 59	60 & above	all	estd. no. (00)	sam-ple	estd. no. (00)	sam-ple
1)	(2)	(3)	(4)	(5)	(6)	(7)	(8)	(9)	(10)	(11)	(12)	(13)	(14)	(15)	(16)	(17)	(18)	(19)	(20)	(21)	(22)	(23)
ttaranchal	22	29	13	27	129	31	3	31	49	40	87	31	15	30	31	34	109	31	2475	158	80361	2654
ttar Pradesh	41	29	58	93	224	57	34	49	87	123	222	68	37	39	72	107	223	63	101847	4127	1625696	56514
Vest Bengal	57	36	66	106	314	78	50	42	76	133	359	87	53	39	71	119	337	82	63387	2272	771007	25040
& N lands	9	0	18	72	239	29	37	22	15	73	114	34	22	11	17	72	191	31	104	53	3303	1256
handigarh	14	8	13	51	144	25	45	50	37	62	258	59	29	26	24	56	196	40	295	135	7304	1817
adra & N. aveli	4	1	0	0	13	2	20	15	0	0	94	14	12	8	0	0	40	8	21	9	2550	785
aman & iu	0	1	0	90	142	7	26	3	2	127	62	20	12	2	1	107	88	12	16	19	1376	730
akshadweep	40	14	59	101	418	67	43	31	50	276	544	104	42	23	54	183	487	86	45	94	527	968
ondicherry	81	51	39	176	559	113	92	30	72	264	692	164	86	40	54	220	636	137	1321	182	9620	1188
NES	41	13	14	21	101	29	26	13	17	30	117	25	34	13	15	25	108	27	2672	826	99045	32960
UTs	39	20	23	97	346	56	56	31	44	148	507	93	47	25	32	121	428	73	1802	492	24681	6744
ll-India	34	21	41	83	245	52	29	30	61	102	257	61	32	26	51	92	251	57	545767	24825	9625001	385055
std. persons eptg. ilment on ne previous ay (00)	59747	27618	38987	48812	83585	258748	46212	37688	57759	58398	86962	287019	105959	65305	96746	107210	170547	545767	X	X	X	X
ample per-ons reptg. ilment on ne previous ay	2265	1301	1655	2179	4607	12007	1711	1579	2228	2684	4616	12818	3976	2880	3883	4863	9223	24825	X	X	X	X
std. no. of ersons (00)	1753735	1285112	962321	591642	341349	4934159	1596113	1236469	948278	571748	338233	4690841	3349848	2521581	1910599	1163390	679582	9625001	X	X	X	X
ample ersons	66155	52020	37833	22503	18188	196699	60005	50989	35870	24111	17381	188356	126160	103009	73703	46614	35569	385055	X	X	X	X

For peer review only - http://bmjopen.bmj.com/site/about/guidelines.xhtml

Appendix A

Table (38): Number of persons per 1,00,000 reporting onset of ailments during last 15 days and average duration of ailment by sex and age-group

All-India Rural

age-group	incidence rate of ailment during last 15 days			average duration of ailment in days (0.0)			persons reporting onset of ailment during last 15 days		all persons	
	male	female	persons	male	female	persons	estd. no. (00)	sam-ple	estd. no. (00)	sam-ple
(1)	(2)	(3)	(4)	(5)	(6)	(7)	(8)	(9)	(10)	(11)
0 – 4	9671	8833	9259	7.7	5.4	6.6	79417	2383	857690	28189
5 – 9	4407	3779	4107	10.7	6.3	8.8	37742	1111	918975	30709
10 – 14	3197	2632	2934	10.0	13.3	11.4	25025	734	852869	28718
5 – 14	5678	5111	5407	8.9	6.9	8.0	142184	4228	2629534	87616
15 – 19	2837	3177	2994	6.5	8.8	7.6	19424	592	648792	23587
20 – 24	2258	3153	2725	16.9	19.4	18.4	16037	518	588414	21533
25 – 29	2280	3646	2982	6.6	10.5	9.0	17057	529	572075	20256
15 – 29	2487	3322	2903	9.5	12.8	11.4	52518	1639	1809280	65376
30 – 34	2888	4026	3489	25.0	7.5	14.4	18089	544	518521	17695
35 – 39	2837	4160	3479	15.4	18.7	17.3	16735	487	481044	16063
40 – 44	3812	5107	4432	53.3	13.4	31.3	16864	445	380519	12605
30 – 44	3135	4357	3745	31.8	13.0	20.9	51688	1476	1380084	46363
45 – 49	4197	4946	4552	17.7	26.8	22.4	14638	435	321561	10882
50 – 54	4003	5450	4741	8.3	25.3	18.2	12178	371	256843	9070
55 – 59	4012	5272	4660	15.8	16.8	16.3	12424	426	266611	9903
45 – 59	4083	5209	4644	14.4	23.0	19.2	39240	1232	845015	29855
60 & above	6516	7022	6766	38.3	46.1	42.3	34828	1527	514787	22768
15 & above	3439	4403	3919	23.0	21.2	22.0	178275	5874	4549167	164362
all ages	4280	4655	4464	16	15.6	15.8	320459	10102	7178701	251978
estd. no. of persons rep.ailment during last 15 days (00)	156534	163925	320459	X	X	X	X	X	X	X
sample persons rep.ailment during last 15 days	4963	5139	10102	X	X	X	X	X	X	X
estd. no. of persons(00)	3657509	3521192	7178701	X	X	X	X	X	X	X
sample persons	128629	123349	251978	X	X	X	X	X	X	X

For peer review only - http://bmjopen.bmj.com/site/about/guidelines.xhtml

Appendix A

Table (38): Number of persons per 1,00,000 reporting onset of ailments during last 15 days and average duration of ailment by sex and age-group

All-India | Urban

age-group	incidence rate of ailment during last 15 days			average duration of ailment in days (0.0)			persons reporting onset of ailment during last 15 days		all persons	
	male	female	persons	male	female	persons	estd. no. (00)	sam-ple	estd. no. (00)	sam-ple
(1)	(2)	(3)	(4)	(5)	(6)	(7)	(8)	(9)	(10)	(11)
0 – 4	10703	9779	10277	6.2	5.2	5.8	22085	1204	214896	13137
5 – 9	5684	4424	5080	8.1	7.6	7.9	11936	526	234965	12478
10 – 14	3207	2950	3086	12.3	14.1	13.1	8345	345	270453	12929
5 – 14	6284	5431	5882	8.0	7.6	7.9	42367	2075	720314	38544
15 – 19	2930	3132	3022	5.1	33.4	18.4	7728	332	255743	12606
20 – 24	2794	3415	3094	5.1	12.3	8.9	7614	333	246050	12901
25 – 29	2891	4351	3587	6.7	8.6	7.8	7551	327	210508	12126
15 – 29	2873	3597	3214	5.6	17.3	11.7	22892	992	712301	37633
30 – 34	3362	3975	3660	10.4	6.1	8.1	7167	275	195796	10654
35 – 39	3695	4086	3891	9.0	20.2	14.9	7077	292	181886	9238
40 – 44	3508	4203	3839	9.4	7.6	8.5	5867	230	152833	7448
30 – 44	3516	4078	3791	9.6	11.5	10.6	20111	797	530515	27340
45 – 49	2651	4971	3708	22.0	48.5	38.3	4773	223	128711	6347
50 – 54	2304	6481	4328	25.0	96.3	77.5	4316	203	99725	5188
55 – 59	4384	4012	4201	15.9	24.9	20.1	3778	200	89940	5224
45 – 59	3019	5173	4042	20.3	62.9	46.5	12867	626	318376	16759
60 & above	4788	6144	5480	104.5	96	99.6	9031	700	164795	12801
15 & above	3267	4292	3760	22.9	37.6	31	64901	3115	1725986	94533
all ages	4167	4623	4385	16.2	27.5	21.9	107268	5190	2446300	133077
estd. no. of persons rep.ailment during last 15 days (00)	53197	54071	107268	X	X	X	X	X	X	X
sample persons rep.ailment during last 15 days	2538	2652	5190	X	X	X	X	X	X	X
estd. no. of persons(00)	1276650	1169650	2446300	X	X	X	X	X	X	X
sample persons	68070	65007	133077	X	X	X	X	X	X	X

For peer review only - http://bmjopen.bmj.com/site/about/guidelines.xhtml

Appendix A

Table (38): Number of persons per 1,00,000 reporting onset of ailments during last 15 days and average duration of ailment by sex and age-group

All-India Rural+Urban

age-group	incidence rate of ailment during last 15 days			average duration of ailment in days (0.0)			persons reporting onset of ailment during last 15 days		all persons	
	male	female	persons	male	female	persons	estd. no. (00)	sam-ple	estd. no. (00)	sam-ple
(1)	(2)	(3)	(4)	(5)	(6)	(7)	(8)	(9)	(10)	(11)
0 – 4	9888	9013	9463	7.4	5.4	6.5	101502	3587	1072586	41326
5 – 9	4666	3911	4305	10.1	6.6	8.6	49678	1637	1153941	43187
10 – 14	3199	2710	2971	10.6	13.5	11.8	33370	1079	1123322	41647
5 – 14	5810	5179	5509	8.7	7.1	8.0	184550	6303	3349848	126160
15 – 19	2863	3164	3002	6.1	15.5	10.7	27152	924	904535	36193
20 – 24	2424	3227	2834	12.6	17.3	15.3	23651	851	834463	34434
25 – 29	2453	3826	3144	6.6	10.0	8.7	24608	856	782583	32382
15 – 29	2600	3397	2991	8.2	14.1	11.5	75411	2631	2521581	103009
30 – 34	3026	4013	3536	20.3	7.2	12.6	25256	819	714317	28349
35 – 39	3067	4139	3592	13.3	19.1	16.6	23812	779	662930	25301
40 – 44	3725	4849	4262	41.4	12.0	25.4	22731	675	533352	20053
30 – 44	3243	4281	3758	25.0	12.6	18.0	71799	2273	1910599	73703
45 – 49	3745	4953	4311	18.6	33.0	26.3	19411	658	450272	17229
50 – 54	3510	5728	4626	11.4	47.8	34.2	16493	574	356568	14258
55 – 59	4109	4965	4544	15.8	18.4	17.2	16203	626	356551	15127
45 – 59	3782	5200	4479	15.7	33.7	26	52108	1858	1163390	46614
60 & above	6108	6803	6454	50.9	57.5	54.4	43859	2227	679582	35569
15 & above	3391	4373	3875	23	25.6	24.4	243176	8989	6275153	258895
all ages	4251	4647	4444	16	18.6	17.3	427727	15292	9625001	385055
estd. no. of persons rep.ailment during last 15 days (00)	209731	217995	427727	X	X	X	X	X	X	X
sample persons rep.ailment during last 15 days	7501	7791	15292	X	X	X	X	X	X	X
estd. no. of persons(00)	4934159	4690841	9625001	X	X	X	X	X	X	X
sample persons	196699	188356	385055	X	X	X	X	X	X	X

For peer review only

For peer review only - http://bmjopen.bmj.com/site/about/guidelines.xhtml

Appendix A

Table (39): Number per 1,00,000 of persons reporting onset of specific ailments during last 15 days and average duration of ailment by broad ailment type

All-India Rural

broad ailment type	incidence rate of ailment during last 15 days			average duration of ailment in days (0.0)			persons reporting onset of ailment during last 15 days	
	male	female	persons	male	female	persons	estd. no. (00)	sam-ple
(1)	(2)	(3)	(4)	(5)	(6)	(7)	(8)	(9)
**gastro-intestinal**								
diarrhoea/ dysentery	403	408	406	5.4	5.5	5.4	29110	958
gastritis/ gastric or peptic ulcer	88	163	125	23.4	17.9	19.9	8987	339
worm infestation	29	29	29	5.9	42.2	23.6	2105	66
amoebiosis	20	26	23	8.5	4.4	6.2	1643	59
hepatitis/ jaundice	15	12	14	9.4	16.6	12.6	975	30
**cardio-vascular diseases**								
heart disease	15	18	16	42.2	108.0	77.5	1152	60
hypertension	31	48	39	219.6	85.7	138.8	2822	103
respiratory including ear/ nose/ throat ailments	453	392	423	8.0	6.6	7.4	30389	990
tuberculosis	9	4	6	201.3	35.9	156.1	455	29
bronchial asthma	82	46	64	85.5	66.5	78.9	4611	147
disorders of joints and bones	63	115	89	65.9	65.0	65.3	6355	235
diseases of kidney/ urinary system	19	13	16	101.4	76.4	91.5	1137	57
prostatic disorders	3	1	2	15.5	5.0	13.2	159	9
gynaecological disorders	3	63	33	2.0	17.4	16.5	2339	90
neurological disorders	32	50	41	28.2	10.0	17.4	2927	87
psychiatric disorders	8	4	6	556.0	16.5	392.6	442	18
**eye ailment**								
conjunctivitis	14	19	17	9.2	5.8	7.2	1189	39
glaucoma	1	5	3	12.0	756.6	649.6	202	6
cataract	7	6	6	96.4	155.1	123.8	451	25
diseases of skin	69	69	69	10.4	20.5	15.4	4962	137
goitre	2	1	1	6.4	6.9	6.5	107	7
diabetes mellitus	13	11	12	88.6	112.9	99.6	853	26
under-nutrition	0	2	1	10.0	3.4	3.5	63	5
anaemia	2	16	9	4.1	10.5	9.7	654	26
sexually transmitted diseases	0	3	1	6.9	30.6	28.7	105	8
**febrile illness**								
malaria	131	140	135	7.1	6.7	6.9	9702	279
eruptive	29	53	41	14.1	7.5	9.9	2937	56
mumps	12	24	18	6.0	27.0	19.7	1295	41
diphtheria	10	6	8	4.4	44.8	19.0	592	16

Continued

For peer review only

Appendix A

Table (39): Number per 1,00,000 of persons reporting onset of specific ailments during last 15 days and average duration of ailment by broad ailment type

All-India Rural

broad ailment type	incidence rate of ailment during last 15 days			average duration of ailment in days (0.0)			persons reporting onset of ailment during last 15 days	
	male	female	persons	male	female	persons	estd. no. (00)	sam-ple
(1)	(2)	(3)	(4)	(5)	(6)	(7)	(8)	(9)
whooping cough	142	188	164	6.7	9.0	8.0	11803	329
fever of unknown origin	1458	1604	1530	5.7	4.9	5.3	109804	3150
tetanus	2	2	2	14.4	7.7	11.3	134	6
filariasis/ elephantiasis	1	1	1	18.5	5.0	12.7	46	5
**disability**								
locomotor	12	25	18	162.7	47.6	86.9	1319	56
visual including blindness (excluding cataract)	3	13	8	136	10.5	37.8	568	24
speech	2	0	1	999.0	0	999.0	87	1
hearing	10	7	8	9.5	9.1	9.3	597	20
diseases of mouth/teeth/ gum	46	70	57	99.2	21.5	53.0	4120	122
accidents/ injuries/ burns/ fractures/ poisoning	187	81	135	7.6	26.6	13.2	9697	303
cancer and other tumours	11	4	8	13.7	31.6	18.4	563	25
other diagnosed ailments	646	720	682	9.7	23.4	16.8	48983	1472
other undiagnosed ailments	117	123	120	17.1	24.7	20.9	8593	274
all	4280	4655	4464	16	15.6	15.8	320459	10102
estd. no. of persons rep. ailment during last 15 days (00)	156534	163925	320459	X	X	X	X	X
sample persons rep. ailment during last 15 days	4963	5139	10102	X	X	X	X	X
estd. no. of persons(00)	3657509	3521192	7178701	X	X	X	X	X
sample persons	128629	123349	251978	X	X	X	X	X

For peer review only - http://bmjopen.bmj.com/site/about/guidelines.xhtml

Appendix A

Table (39): Number per 1,00,000 of persons reporting onset of specific ailments during last 15 days and average duration of ailment by broad ailment type

All-India Urban

broad ailment type	incidence rate of ailment during last 15 days			average duration of ailment in days (0.0)			persons reporting onset of ailment during last 15 days	
	male	female	persons	male	female	persons	estd. no. (00)	sam-ple
(1)	(2)	(3)	(4)	(5)	(6)	(7)	(8)	(9)
**gastro-intestinal**								
diarrhoea/ dysentery	333	286	310	7.8	4.0	6.2	7590	368
gastritis/ gastric or peptic ulcer	75	133	103	8.2	40.1	27.9	2516	117
worm infestation	21	30	25	6.4	29.3	19.6	619	27
amoebiosis	18	14	17	3.8	6.2	4.8	405	23
hepatitis/ jaundice	21	10	16	4.9	21.9	9.9	383	30
**cardio-vascular diseases**								
heart disease	28	30	29	102.6	123.5	113.0	711	60
hypertension	61	116	87	246.3	190.1	210.7	2139	97
respiratory including ear/ nose/ throat ailments	527	474	502	5.7	12.4	8.7	12270	589
tuberculosis	8	1	5	19.1	15.	18.6	122	12
bronchial asthma	59	46	53	75.7	96.3	84.3	1296	73
disorders of joints and bones	67	132	98	75.2	99.5	90.9	2401	128
diseases of kidney/ urinary system	20	35	27	52.3	31.2	39.3	668	37
prostatic disorders	2	1	1	60.0	12.3	46.5	31	3
gynaecological disorders	0	67	32	0	16.7	16.7	785	58
neurological disorders	14	31	22	93.4	251.9	198.2	546	35
psychiatric disorders	6	3	5	2.9	9.4	4.8	110	6
**eye ailment**								
conjunctivitis	34	13	24	8.0	3.5	6.9	577	22
glaucoma	1	4	2	5.0	186.7	163.2	59	6
cataract	4	7	6	204.7	63.9	119.1	143	15
diseases of skin	45	83	64	9.5	17.3	14.4	1554	69
goitre	0	2	1	12.0	17.8	17.0	24	3
diabetes mellitus	20	34	27	191.4	619.6	453.9	655	41
under-nutrition	0	8	4	0	5.3	5.3	89	5
anaemia	5	15	10	79.5	7.1	26.4	240	17
sexually transmitted diseases	0	0	0	0	0	0	0	0
**febrile illness**								
malaria	69	77	73	5.7	6.2	6.0	1783	91
eruptive	13	19	16	9.7	7.1	8.2	388	14
mumps	6	31	18	5.2	6.2	6.0	441	19
diphtheria	28	7	18	8.8	6.7	8.4	438	13

Continued

For peer review only - http://bmjopen.bmj.com/site/about/guidelines.xhtml

Appendix A

Table (39): Number per 1,00,000 of persons reporting onset of specific ailments during last 15 days and average duration of ailment by broad ailment type

All-India Urban

broad ailment type	incidence rate of ailment during last 15 days			average duration of ailment in days (0.0)			persons reporting onset of ailment during last 15 days	
	male	female	persons	male	female	persons	estd. no. (00)	sam-ple
(1)	(2)	(3)	(4)	(5)	(6)	(7)	(8)	(9)
whooping cough	226	108	169	6.2	43	17.4	4143	196
fever of unknown origin	1212	1497	1349	5.1	5.8	5.4	32991	1495
tetanus	-	-	-	-	-	-	0	0
filariasis/ elephantiasis	0	2	1	0	6.7	6.7	22	2
**disability**								
locomotor	16	18	17	402.0	72.0	235.2	411	23
visual including blindness (excluding cataract)	3	19	11	13.9	202.2	174.2	264	13
speech	0	0	0	999.0	0	999.0	3	1
hearing	27	11	19	39.6	65	46.4	464	17
diseases of mouth/teeth/ gum	54	88	70	16.7	11.5	13.6	1722	77
accidents/ injuries/ burns/ fractures/ poisoning	219	82	154	12.8	6.8	11.2	3758	166
cancer and other tumours	13	4	9	12.1	474.8	124.5	212	16
other diagnosed ailments	792	914	851	6.6	14.7	10.7	20812	880
other undiagnosed ailments	83	115	98	7.3	5.2	6.1	2404	127
all	4167	4623	4385	16.2	27.5	21.9	107268	5190
estd. no. of persons rep. ailment during last 15 days (00)	53197	54071	107268	X	X	X	X	X
sample persons rep. ailment during last 15 days	2538	2652	5190	X	X	X	X	X
estd. no. of persons(00)	1276650	1169650	2446300	X	X	X	X	X
sample persons	68070	65007	133077	X	X	X	X	X

For peer review only - http://bmjopen.bmj.com/site/about/guidelines.xhtml

Appendix A

Table (39): Number per 1,00,000 of persons reporting onset of specific ailments during last 15 days and average duration of ailment by broad ailment type

All-India Rural + Urban

broad ailment type	incidence rate of ailment during last 15 days			average duration of ailment in days (0.0)			persons reporting onset of ailment during last 15 days	
	male	female	persons	male	female	persons	estd. no. (00)	sam-ple
(1)	(2)	(3)	(4)	(5)	(6)	(7)	(8)	(9)
**gastro-intestinal**								
diarrhoea/ dysentery	385	378	381	5.9	5.2	5.6	36701	1326
gastritis/ gastric or peptic ulcer	85	156	120	19.9	22.6	21.6	11503	456
worm infestation	27	30	28	6.0	38.9	22.7	2724	93
amoebiosis	19	23	21	7.3	4.7	5.9	2047	82
hepatitis/ jaundice	16	12	14	7.9	17.7	11.8	1358	60
**cardio-vascular diseases**								
heart disease	18	21	19	66.4	113.6	91.0	1863	120
hypertension	39	65	52	230.6	132.0	169.8	4961	200
respiratory including ear/ nose/ throat ailments	473	412	443	7.3	8.3	7.8	42659	1579
tuberculosis	9	3	6	156.5	33.8	127.1	577	41
bronchial asthma	76	46	61	83.6	74.0	80.1	5908	220
disorders of joints and bones	64	120	91	68.4	74.6	72.3	8756	363
diseases of kidney/ urinary system	19	18	19	88.0	54.9	72.2	1805	94
prostatic disorders	3	1	2	22.3	6.5	18.6	190	12
gynaecological disorders	3	64	32	2.0	17.2	16.6	3124	148
neurological disorders	28	45	36	37.1	51.4	45.8	3472	122
psychiatric disorders	8	4	6	443.3	15.2	315.2	553	24
**eye ailment**								
conjunctivitis	19	18	18	8.7	5.4	7.1	1766	61
glaucoma	1	5	3	10.5	626.2	539.8	261	12
cataract	6	6	6	116.8	128.6	122.7	594	40
diseases of skin	63	73	68	10.2	19.6	15.1	6515	206
goitre	2	1	1	6.6	11.3	8.0	131	10
diabetes mellitus	15	17	16	124.8	370.8	253.6	1508	67
under-nutrition	0	3	2	10.0	4.5	4.5	152	10
anaemia	3	16	9	37.3	9.7	14.2	894	43
sexually transmitted diseases	0	2	1	6.9	30.6	28.7	105	8
**febrile illness**								
malaria	115	124	119	6.9	6.7	6.8	11484	370
eruptive	25	45	35	13.5	7.4	9.7	3324	70
mumps	11	26	18	5.9	20.7	16.2	1736	60
diphtheria	15	6	11	6.5	34.4	14.5	1030	29

Continued

For peer review only - http://bmjopen.bmj.com/site/about/guidelines.xhtml

For peer review only

Table (39): Number per 1,00,000 of persons reporting onset of specific ailments during last 15 days and average duration of ailment by broad ailment type

All-India — Rural + Urban

broad ailment type	incidence rate of ailment during last 15 days			average duration of ailment in days (0.0)			persons reporting onset of ailment during last 15 days	
	male	female	persons	male	female	persons	estd. no. (00)	sam-ple
(1)	(2)	(3)	(4)	(5)	(6)	(7)	(8)	(9)
whooping cough	163	168	166	6.5	14.5	10.5	15946	525
fever of unknown origin	1395	1577	1484	5.6	5.1	5.3	142795	4645
tetanus	1	1	1	14.4	7.7	11.3	134	6
filariasis/ elephantiasis	1	1	1	18.5	5.9	10.7	68	7
**disability**								
locomotor	13	23	18	237.1	52.3	122.2	1730	79
visual including blindness (excluding cataract)	3	14	9	106.5	74.9	81.1	833	37
speech	2	0	1	999.0	0	999.0	90	2
hearing	14	8	11	24	28.7	25.6	1062	37
diseases of mouth/teeth/ gum	48	74	61	75.0	18.6	41.4	5842	199
accidents/ injuries/ burns/ fractures/ poisoning	195	81	140	9.1	21.6	12.6	13455	469
cancer and other tumours	12	4	8	13.2	146.4	47.4	775	41
other diagnosed ailments	684	768	725	8.8	20.8	15	69795	2352
other undiagnosed ailments	108	121	114	15.1	20.1	17.7	10998	401
all	4251	4647	4444	16	18.6	17.3	427727	15292
estd. no. of persons rep. ailment during last 15 days (00)	209731	217995	427727	X	X	X	X	X
sample persons rep. ailment during last 15 days	7501	7791	15292	X	X	X	X	X
estd. no. of persons(00)	4934159	4690841	9625001	X	X	X	X	X
sample persons	196699	188356	385055	X	X	X	X	X

For peer review only - http://bmjopen.bmj.com/site/about/guidelines.xhtml

Appendix A

Table (40): Average number of spells of ailment during last 15 days, average time of indisposition during last 15 days, average total duration of ailment and average loss of household income due to ailment during last 15 days for each age-group

All-India Rural

broad age-group	average number of spells of ailment* (0.0)	average (per person) number of days during last 15 days (bl. 9)			total duration of ailment from commencement	average loss of household income due to ailment (Rs.)	number of spells of ailments		persons reporting ailment during last 15 days	
		ill	on restricted activity	confined to bed			estd. (00)	sample	estd. no. (00)	sample
(1)	(2)	(3)	(4)	(5)	(6)	(7)	(8)	(9)	(10)	(11)
males										
0 – 14	1.0	7	2	1	44	35	105393	3280	104236	3251
15 – 29	1.0	8	3	1	98	155	37390	1414	36970	1399
30 – 44	1.0	9	3	1	181	284	44602	1561	43897	1536
45 – 59	1.0	11	4	1	269	566	47563	1747	45571	1688
60 & above	1.1	14	4	1	438	113	84430	3836	74362	3413
all	1.0	10	3	1	200	184	319377	11838	305036	11287
females										
0 – 14	1.0	6	2	1	31	35	85568	2587	85021	2576
15 – 29	1.0	8	2	1	99	64	51632	1742	51105	1727
30 – 44	1.0	10	3	1	166	83	64963	2123	63830	2083
45 – 59	1.0	11	3	1	267	75	57385	2191	55366	2116
60 & above	1.1	14	4	1	479	37	81964	3530	71576	3120
all	1.0	10	3	1	206	56	341512	12173	326898	11622
persons										
0 – 14	1.0	6	2	1	38	35	190961	5867	189257	5827
15 – 29	1.0	8	3	1	99	102	89022	3156	88074	3126
30 – 44	1.0	10	3	1	172	165	109565	3684	107728	3619
45 – 59	1.0	11	3	1	268	297	104948	3938	100937	3804
60 & above	1.1	14	4	1	458	76	166394	7366	145938	6533
all	1.0	10	3	1	203	118	660890	24011	631934	22909

* begun, ended or continuing per person reporting ailment during last 15 days

For peer review only

Table (40): Average number of spells of ailment during last 15 days, average time of indisposition during last 15 days, average total duration of ailment and average loss of household income due to ailment during last 15 days for each age-group

All-India Urban

broad age-group	average number of spells of ail-ment* (0.0)	average (per person) number of days during last 15 days (bl. 9)			total duration of ailment from commen-cement	average loss of household income due to ailment (Rs.)	number of spells of ailments		persons reporting ailment during last 15 days	
		ill	on restric-ted activity	con-fined to bed			estd. (00)	sample	estd. no. (00)	sample
(1)	(2)	(3)	(4)	(5)	(6)	(7)	(8)	(9)	(10)	(11)
					males					
0 – 14	1.0	6	1	1	48	25	32827	1618	32106	1602
15 – 29	1.0	8	2	1	102	135	17039	776	16745	765
30 – 44	1.0	10	2	1	167	393	17978	897	17448	869
45 – 59	1.0	13	3	1	402	273	22333	1184	21321	1118
60 & above	1.2	16	4	1	669	75	34375	2510	28366	2100
all	1.1	11	2	1	291	154	124552	6985	115986	6454
					females					
0 – 14	1.0	6	2	1	50	25	25293	1283	25005	1273
15 – 29	1.0	9	2	0	124	44	19119	988	18704	973
30 – 44	1.0	11	2	0	242	44	25472	1175	24598	1148
45 – 59	1.1	13	2	1	411	31	28164	1547	26164	1422
60 & above	1.2	16	4	1	654	20	39377	2814	32220	2331
all	1.1	11	2	1	326	31	137425	7807	126691	7147
					persons					
0 – 14	1.0	6	1	1	49	25	58120	2901	57111	2875
15 – 29	1.0	8	2	1	114	87	36157	1764	35450	1738
30 – 44	1.0	10	2	1	211	189	43450	2072	42046	2017
45 – 59	1.1	13	2	1	407	140	50498	2731	47484	2540
60 & above	1.2	16	4	1	661	46	73753	5324	60586	4431
all	1.1	11	2	1	309	90	261977	14792	242677	13601

* begun, ended or continuing per person reporting ailment during last 15 days

For peer review only

For peer review only - http://bmjopen.bmj.com/site/about/guidelines.xhtml

Appendix A

Table (40): Average number of spells of ailment during last 15 days, average time of indisposition during last 15 days, average total duration of ailment and average loss of household income due to ailment during last 15 days for each age-group

All-India Rural+Urban

broad age-group	average number of spells of ail-ment* (0.0)	average (per person) number of days during last 15 days (bl. 9)			total duration of ailment from commen-cement	average loss of household income due to ailment (Rs.)	number of spells of ailments		persons reporting ailment during last 15 days	
		ill	on restric-ted activity	con-fined to bed			estd. (00)	sample	estd. no. (00)	sample
(1)	(2)	(3)	(4)	(5)	(6)	(7)	(8)	(9)	(10)	(11)
males										
0 – 14	1.0	7	2	1	45	33	138219	4898	136342	4853
15 – 29	1.0	8	3	1	99	149	54428	2190	53715	2164
30 – 44	1.0	9	3	1	177	315	62580	2458	61345	2405
45 – 59	1.0	12	3	1	311	473	69896	2931	66892	2806
60 & above	1.2	15	4	1	502	103	118805	6346	102729	5513
all	1.1	10	3	1	225	176	443929	18823	421022	17741
females										
0 – 14	1.0	6	2	1	35	33	110861	3870	110026	3849
15 – 29	1.0	8	2	1	106	59	70750	2730	69809	2700
30 – 44	1.0	10	2	1	187	72	90435	3298	88428	3231
45 – 59	1.0	12	3	1	313	61	85550	3738	81530	3538
60 & above	1.2	15	4	1	533	32	121342	6344	103796	5451
all	1.1	10	3	1	240	49	478938	19980	453589	18769
persons										
0 – 14	1.0	6	2	1	41	33	249080	8768	246368	8702
15 – 29	1.0	8	2	1	103	98	125179	4920	123524	4864
30 – 44	1.0	10	3	1	183	172	153015	5756	149773	5636
45 – 59	1.0	12	3	1	312	247	155446	6669	148422	6344
60 & above	1.2	15	4	1	518	67	240147	12690	206524	10964
all	1.1	10	3	1	232	110	922867	38803	874611	36510

* begun, ended or continuing per person reporting ailment during last 15 days

For peer review only

For peer review only - http://bmjopen.bmj.com/site/about/guidelines.xhtml

Appendix A

Table (41): Number per 1000 of spells of ailment during last 15 days treated on medical advice and per 1000 distribution of untreated spells of ailments not given medical treatment by reason for not taking treatment for each mpce class and social-group

All-India Rural Males

mpce class/ social group	number per 1000 of spells ailments treated on medical advice during last 15 days from			spells of ailments not given medical treatment by reason								untreated spells of ailment		spells of ailment	
				no medical facilities available in the neighbour-hood	facilities available but no treatment sought owing to					n.r.	total	estd. no. (00)	sample	estd. no. (00)	sample
	govern-ment	private	all		lack of faith	long waiting	financial reason	ailment not considered serious	others						
(1)	(2)	(3)	(4)	(5)	(6)	(7)	(8)	(9)	(10)	(11)	(12)	(13)	(14)	(15)	(16)
0 – 225	204	523	727	229	49	0	431	64	115	111	1000	3897	108	14300	451
225 – 255	174	630	804	66	58	0	329	194	172	181	1000	1852	73	9454	312
255 – 300	186	602	787	22	33	8	341	321	115	161	1000	4937	171	23222	779
300 – 340	209	551	760	152	17	0	368	254	168	40	1000	5244	155	21865	733
340 – 380	181	626	807	139	27	0	414	259	93	69	1000	5294	177	27412	921
380 – 420	177	617	794	133	28	25	245	291	148	130	1000	6238	198	30282	1069
420 – 470	194	632	827	147	22	7	189	433	115	88	1000	5575	208	32143	1198
470 – 525	139	657	797	156	60	35	216	284	164	86	1000	7239	247	35617	1330
525 – 615	184	665	849	126	35	2	228	334	166	108	1000	5237	241	34649	1416
615 – 775	178	704	881	69	23	0	248	390	200	70	1000	4845	194	40836	1632
775 – 950	215	658	873	156	64	35	174	292	145	134	1000	2873	100	22606	863
950 +	140	749	889	45	10	0	54	543	126	221	1000	3009	115	26991	1134
all	179	645	824	125	34	11	272	307	144	107	1000	56241	1987	319377	11838
social group:															
ST	248	502	749	213	18	2	278	197	174	118	1000	4892	258	19511	1034
SC	200	600	800	146	23	0	348	270	103	110	1000	13647	416	68068	2261
OBC	165	663	828	93	42	6	266	325	160	108	1000	22554	775	131074	4821
others	171	679	850	125	39	30	210	350	148	98	1000	15147	538	100724	3722
all	179	645	824	125	34	11	272	307	144	107	1000	56241	1987	319377	11838

For peer review only

For peer review only - http://bmjopen.bmj.com/site/about/guidelines.xhtml

Appendix A

Table (41): Number per 1000 of spells of ailment during last 15 days treated on medical advice and per 1000 distribution of untreated spells of ailments not given medical treatment by reason for not taking treatment for each mpce class and social-group

All-India | Rural | Females

mpce class/ social group	number per 1000 of spells ailments treated on medical advice during last 15 days from			spells of ailments not given medical treatment by reason								untreated spells of ailment		spells of ailment	
				no medical facilities available in the neighbour-hood	facilities available but no treatment sought owing to					n.r.	total	estd. no. (00)	sample	estd. no. (00)	sample
	govern-ment	private	all		lack of faith	long waiting	financial reason	ailment not considered serious	others						
(1)	(2)	(3)	(4)	(5)	(6)	(7)	(8)	(9)	(10)	(11)	(12)	(13)	(14)	(15)	(16)
0 – 225	246	545	791	131	35	0	405	196	170	64	1000	2853	83	13676	420
225 – 255	235	518	753	80	51	0	357	374	61	78	1000	2291	82	9287	318
255 – 300	212	554	766	114	38	7	409	248	100	83	1000	5756	187	24627	779
300 – 340	187	566	753	42	21	0	316	307	185	129	1000	5439	162	22035	765
340 – 380	160	626	786	103	16	1	375	261	153	91	1000	6210	173	28996	973
380 – 420	177	640	817	186	9	0	356	327	71	51	1000	5599	176	30553	1054
420 – 470	170	644	814	100	50	15	213	364	211	48	1000	6678	227	35942	1216
470 – 525	189	642	831	130	13	12	289	320	153	82	1000	6386	224	37794	1315
525 – 615	192	617	809	106	27	3	220	422	167	55	1000	6900	248	36180	1382
615 – 775	193	664	857	75	10	23	236	346	189	121	1000	6079	218	42567	1706
775 – 950	191	690	881	145	32	7	179	401	167	69	1000	3127	110	26282	960
950 +	176	722	897	48	33	5	131	440	146	197	1000	3450	134	33574	1285
all	188	634	822	106	26	7	290	333	153	86	1000	60768	2024	341512	12173
social group:															
ST	250	532	782	183	0	4	361	230	152	69	1000	5073	235	23253	1022
SC	217	579	796	120	26	2	382	294	141	35	1000	14627	429	71568	2340
OBC	175	658	833	68	24	5	249	408	147	99	1000	23760	794	142093	4995
others	173	662	835	123	37	14	245	292	170	118	1000	17308	566	104598	3816
all	188	634	822	106	26	7	290	333	153	86	1000	60768	2024	341512	12173

For peer review only

For peer review only - http://bmjopen.bmj.com/site/about/guidelines.xhtml

Table (41): Number per 1000 of spells of ailment during last 15 days treated on medical advice and per 1000 distribution of untreated spells of ailments not given medical treatment by reason for not taking treatment for each mpce class and social-group

All-India Rural Persons

mpce class/ social group	number per 1000 of spells ailments treated on medical advice during last 15 days from			spells of ailments not given medical treatment by reason								untreated spells of ailment		spells of ailment	
				no medical facilities available in the neighbour-hood	facilities available but no treatment sought owing to					n.r.	total	estd. no. (00)	sample	estd. no. (00)	sample
	govern-ment	private	all		lack of faith	long waiting	financial reason	ailment not considered serious	others						
(1)	(2)	(3)	(4)	(5)	(6)	(7)	(8)	(9)	(10)	(11)	(12)	(13)	(14)	(15)	(16)
0 – 225	225	534	759	188	43	0	420	120	138	91	1000	6750	191	27976	871
225 – 255	204	575	779	74	54	0	344	294	111	124	1000	4143	155	18741	630
255 – 300	200	577	777	72	36	7	378	282	107	119	1000	10693	358	47850	1558
300 – 340	198	558	757	96	19	0	341	281	177	85	1000	10683	317	43900	1498
340 – 380	170	626	796	119	21	0	393	260	125	81	1000	11505	350	56408	1894
380 – 420	177	629	805	158	19	13	298	308	112	93	1000	11837	374	60834	2123
420 – 470	182	638	820	121	37	11	202	395	167	66	1000	12253	435	68085	2414
470 – 525	165	650	814	144	38	24	250	301	159	84	1000	13625	471	73412	2645
525 – 615	188	641	829	115	31	3	223	384	167	78	1000	12137	489	70828	2798
615 – 775	186	683	869	72	16	13	241	365	194	98	1000	10925	412	83403	3338
775 – 950	202	676	877	150	47	21	177	349	156	100	1000	6000	210	48888	1823
950 +	160	734	893	47	23	2	95	488	137	208	1000	6459	249	60565	2419
all	184	639	823	115	30	9	281	321	148	96	1000	117009	4011	660890	24011
social group:															
ST	249	518	767	198	9	3	321	213	163	93	1000	9965	493	42764	2056
SC	209	589	798	133	24	1	366	283	123	71	1000	28274	845	139636	4601
OBC	170	660	830	80	33	6	258	368	153	103	1000	46315	1569	273167	9816
others	172	670	842	124	38	21	229	319	160	109	1000	32455	1104	205322	7538
all	184	639	823	115	30	9	281	321	148	96	1000	117009	4011	660890	24011

For peer review only - http://bmjopen.bmj.com/site/about/guidelines.xhtml

For peer review only

Appendix A

Table (41): Number per 1000 of spells of ailment during last 15 days treated on medical advice and per 1000 distribution of untreated spells of ailments not given medical treatment by reason for not taking treatment for each mpce class and social-group

All-India | Urban | Males

mpce class/ social group	number per 1000 of spells ailments treated on medical advice during last 15 days from: govern-ment	private	all	spells of ailments not given medical treatment by reason: no medical facilities available in the neighbour-hood	facilities available but no treatment sought owing to: lack of faith	long waiting	financial reason	ailment not considered serious	others	n.r.	total	untreated spells of ailment: estd. no. (00)	sample	spells of ailment: estd. no. (00)	sample
(1)	(2)	(3)	(4)	(5)	(6)	(7)	(8)	(9)	(10)	(11)	(12)	(13)	(14)	(15)	(16)
0 –300	217	620	837	6	32	0	372	428	111	52	1000	512	33	3134	163
300 – 350	181	675	856	0	136	2	310	473	45	34	1000	469	18	3248	129
350 – 425	200	663	863	0	3	10	297	322	151	217	1000	919	66	6690	364
425 – 500	202	657	859	19	20	11	310	480	68	91	1000	1766	108	12528	726
500 – 575	224	685	909	0	4	7	247	538	106	97	1000	552	53	6039	383
575 – 665	251	601	852	0	2	6	275	423	140	154	1000	1281	80	8641	532
665 – 775	153	761	914	52	9	0	161	384	196	196	1000	1206	90	14040	799
775 – 915	216	697	913	10	45	8	158	495	117	166	1000	1067	81	12207	775
915 – 1120	192	718	910	0	0	5	59	778	34	124	1000	1468	82	16341	913
1120 – 1500	118	788	906	2	7	8	74	655	173	82	1000	1984	108	21069	1093
1500 – 1925	171	720	891	0	19	16	0	885	0	81	1000	644	32	5914	381
1925 +	96	840	936	0	4	0	13	498	228	258	1000	934	60	14701	727
all	172	725	897	9	17	6	176	538	120	133	1000	12802	811	124552	6985
social group:															
ST	205	689	895	39	2	4	129	384	373	69	1000	199	54	1890	254
SC	208	655	863	1	37	7	330	469	104	52	1000	2360	135	17238	914
OBC	188	716	904	0	24	14	142	524	102	194	1000	3922	271	40801	2403
others	152	750	902	16	5	2	141	578	130	128	1000	6321	351	64623	3414
all	172	725	897	9	17	6	176	538	120	133	1000	12802	811	124552	6985

For peer review only

For peer review only - http://bmjopen.bmj.com/site/about/guidelines.xhtml

Appendix A

Table (41): Number per 1000 of spells of ailment during last 15 days treated on medical advice and per 1000 distribution of untreated spells of ailments not given medical treatment by reason for not taking treatment for each mpce class and social-group

All-India | Urban | Females

mpce class/ social group	number per 1000 of spells ailments treated on medical advice during last 15 days from			spells of ailments not given medical treatment by reason								untreated spells of ailment		spells of ailment	
				no medical facilities available in the neighbour-hood	facilities available but no treatment sought owing to					n.r.	total	estd. no. (00)	sample	estd. no. (00)	sample
	govern-ment	private	all		lack of faith	long waiting	financial reason	ailment not considered serious	others						
(1)	(2)	(3)	(4)	(5)	(6)	(7)	(8)	(9)	(10)	(11)	(12)	(13)	(14)	(15)	(16)
0 –300	187	534	721	4	8	0	408	337	193	49	1000	777	47	2788	163
300 – 350	194	703	897	95	0	31	577	204	16	77	1000	309	26	2996	152
350 – 425	231	602	833	0	21	0	386	414	117	63	1000	1196	67	7165	401
425 – 500	230	619	848	11	19	2	312	401	189	66	1000	2085	117	13742	777
500 – 575	190	694	884	37	53	43	327	359	17	163	1000	752	53	6470	390
575 – 665	204	667	872	0	2	41	325	371	147	114	1000	1420	95	11064	607
665 – 775	157	741	898	12	43	78	194	351	166	156	1000	1690	106	16641	910
775 – 915	178	732	910	80	64	9	261	352	123	110	1000	1232	101	13655	881
915 – 1120	152	742	893	7	19	12	172	570	95	123	1000	1893	107	17770	1047
1120 – 1500	162	724	886	1	3	45	59	604	140	147	1000	2382	128	20930	1226
1500 – 1925	136	750	886	0	0	0	65	714	61	160	1000	775	40	6810	426
1925 +	110	844	954	0	5	0	0	780	102	113	1000	806	48	17392	827
all	170	718	889	14	21	25	227	469	130	115	1000	15318	935	137425	7807
social group:															
ST	320	542	862	369	1	5	93	319	131	81	1000	268	52	1944	284
SC	208	663	871	17	10	32	373	336	180	53	1000	2409	144	18635	972
OBC	176	700	876	5	30	43	270	420	121	112	1000	5450	355	44013	2690
others	153	748	901	7	18	9	152	556	119	139	1000	7191	384	72833	3861
all	170	718	889	14	21	25	227	469	130	115	1000	15318	935	137425	7807

For peer review only

For peer review only - http://bmjopen.bmj.com/site/about/guidelines.xhtml

Appendix A

Table (41): Number per 1000 of spells of ailment during last 15 days treated on medical advice and per 1000 distribution of untreated spells of ailments not given medical treatment by reason for not taking treatment for each mpce class and social-group

All-India Urban Persons

mpce class/ social group	number per 1000 of spells ailments treated on medical advice during last 15 days from			spells of ailments not given medical treatment by reason								untreated spells of ailment		spells of ailment	
				no medical facilities available in the neighbour-hood	facilities available but no treatment sought owing to					n.r.	total	estd. no. (00)	sample	estd. no. (00)	sample
	govern-ment	private	all		lack of faith	long waiting	financial reason	ailment not considered serious	others						
(1)	(2)	(3)	(4)	(5)	(6)	(7)	(8)	(9)	(10)	(11)	(12)	(13)	(14)	(15)	(16)
0 –300	203	579	782	5	17	0	394	373	160	50	1000	1289	80	5922	326
300 – 350	187	688	875	38	82	13	416	366	34	51	1000	779	44	6245	281
350 – 425	216	631	847	0	13	4	347	374	131	130	1000	2115	133	13854	765
425 – 500	216	637	853	15	20	6	311	437	134	78	1000	3852	225	26270	1503
500 – 575	206	690	896	22	32	28	293	435	55	135	1000	1304	106	12509	773
575 – 665	225	638	863	0	2	24	301	396	144	133	1000	2701	175	19705	1139
665 – 775	155	750	906	29	29	46	180	365	178	173	1000	2896	196	30682	1709
775 – 915	196	715	911	48	56	9	213	418	120	136	1000	2299	182	25862	1656
915 – 1120	171	730	901	4	11	9	123	661	68	124	1000	3361	189	34111	1960
1120 – 1500	140	756	896	1	5	28	66	627	155	117	1000	4366	236	42000	2319
1500 – 1925	152	736	889	0	9	7	36	791	33	124	1000	1419	72	12724	807
1925 +	103	842	946	0	4	0	7	629	169	191	1000	1739	108	32093	1554
all	171	721	893	12	19	16	204	500	125	123	1000	28120	1746	261977	14792
social group:															
ST	264	614	878	228	2	5	109	347	234	76	1000	467	106	3834	538
SC	208	659	867	9	23	19	352	402	143	52	1000	4769	279	35873	1886
OBC	182	708	890	3	27	31	216	463	113	146	1000	9372	626	84814	5093
others	153	749	902	12	12	6	147	566	124	134	1000	13512	735	137456	7275
all	171	721	893	12	19	16	204	500	125	123	1000	28120	1746	261977	14792

For peer review only - http://bmjopen.bmj.com/site/about/guidelines.xhtml

Appendix A

Table (41): Number per 1000 of spells of ailment during last 15 days treated on medical advice and per 1000 distribution of untreated spells of ailments not given medical treatment by reason for not taking treatment for each mpce class and social-group

All-India — Rural + Urban — Males

mpce class/ social group	number per 1000 of spells ailments treated on medical advice during last 15 days from			spells of ailments not given medical treatment by reason								untreated spells of ailment		spells of ailment	
				no medical facilities available in the neighbourhood	facilities available but no treatment sought owing to					n.r.	total	estd. no. (00)	sample	estd. no. (00)	sample
	govern-ment	private	all		lack of faith	long waiting	financial reason	ailment not considered serious	others						
(1)	(2)	(3)	(4)	(5)	(6)	(7)	(8)	(9)	(10)	(11)	(12)	(13)	(14)	(15)	(16)
0 – 235	207	540	747	203	47	0	424	107	115	104	1000	4410	141	17434	614
235 – 265	176	641	817	53	74	0	325	251	146	151	1000	2321	91	12703	441
265 – 320	189	615	804	18	29	8	334	321	121	170	1000	5856	237	29912	1143
320 – 365	207	589	796	119	18	3	354	311	143	53	1000	7010	263	34392	1459
365 – 410	189	637	825	126	24	1	398	285	94	72	1000	5846	230	33451	1304
410 – 460	193	614	807	111	24	21	250	313	147	134	1000	7519	278	38923	1601
460 – 520	182	672	853	130	19	5	184	425	129	107	1000	6781	298	46183	1997
520 – 605	159	668	826	137	58	31	209	311	158	96	1000	8306	328	47824	2105
605 – 730	186	682	868	99	28	3	191	431	137	111	1000	6705	323	50990	2329
730 – 980	157	732	890	49	19	2	197	467	192	73	1000	6829	302	61906	2725
980 – 1285	206	671	877	127	56	31	142	401	119	124	1000	3517	132	28520	1244
1285 +	124	781	905	35	9	0	44	532	150	230	1000	3942	175	41692	1861
all	177	667	844	103	31	10	254	350	140	112	1000	69043	2798	443929	18823
social group:															
ST	244	518	762	206	17	2	272	204	182	116	1000	5092	312	21401	1288
SC	202	611	812	125	25	1	345	299	103	101	1000	16007	551	85306	3175
OBC	170	676	846	79	39	7	248	355	151	121	1000	26476	1046	171875	7224
others	164	707	870	93	29	22	190	417	143	107	1000	21468	889	165347	7136
all	177	667	844	103	31	10	254	350	140	112	1000	69043	2798	443929	18823

For peer review only - http://bmjopen.bmj.com/site/about/guidelines.xhtml

Appendix A

Table (41): Number per 1000 of spells of ailment during last 15 days treated on medical advice and per 1000 distribution of untreated spells of ailments not given medical treatment by reason for not taking treatment for each mpce class and social-group

All-India | Rural + Urban | Females

mpce class/ social group	number per 1000 of spells ailments treated on medical advice during last 15 days from			spells of ailments not given medical treatment by reason								untreated spells of ailment		spells of ailment	
				no medical facilities available in the neighbour-hood	facilities available but no treatment sought owing to					n.r.	total	estd. no. (00)	sample	estd. no. (00)	sample
	govern-ment	private	all		lack of faith	long waiting	financial reason	ailment not considered serious	others						
(1)	(2)	(3)	(4)	(5)	(6)	(7)	(8)	(9)	(10)	(11)	(12)	(13)	(14)	(15)	(16)
0 – 235	236	543	780	104	29	0	405	226	175	61	1000	3630	130	16464	583
235 – 265	225	563	788	82	45	4	383	354	56	78	1000	2600	108	12283	470
265 – 320	217	565	781	95	35	5	405	277	103	80	1000	6952	254	31792	1180
320 – 365	203	586	790	33	21	0	315	333	186	112	1000	7525	279	35777	1542
365 – 410	165	639	804	96	20	5	369	272	139	99	1000	6963	226	35466	1363
410 – 460	184	647	831	148	8	8	350	336	86	64	1000	7019	271	41617	1661
460 – 520	166	675	841	82	49	28	209	361	202	70	1000	8367	333	52583	2126
520 – 605	186	666	852	122	22	12	285	325	148	87	1000	7618	325	51450	2196
605 – 730	179	658	837	85	25	5	210	454	152	70	1000	8793	355	53950	2429
730 – 980	183	684	867	54	8	30	186	418	175	129	1000	8461	346	63497	2932
980 – 1285	179	703	882	117	26	6	156	463	146	87	1000	3901	150	33092	1386
1285 +	153	763	916	39	28	4	106	505	137	181	1000	4256	182	50967	2112
all	183	658	841	87	25	10	277	360	148	92	1000	76086	2959	478938	19980
social group:															
ST	256	532	788	192	0	4	348	234	151	70	1000	5341	287	25197	1306
SC	215	596	811	105	23	6	381	300	147	37	1000	17036	573	90203	3312
OBC	175	668	843	56	25	13	253	410	142	101	1000	29210	1149	186106	7685
others	165	697	862	89	32	12	218	370	155	124	1000	24499	950	177431	7677
all	183	658	841	87	25	10	277	360	148	92	1000	76086	2959	478938	19980

For peer review only

For peer review only - http://bmjopen.bmj.com/site/about/guidelines.xhtml

Appendix A

Table (41): Number per 1000 of spells of ailment during last 15 days treated on medical advice and per 1000 distribution of untreated spells of ailments not given medical treatment by reason for not taking treatment for each mpce class and social-group

All-India — Rural + Urban — Persones

mpce class/ social group	number per 1000 of spells ailments treated on medical advice during last 15 days from			spells of ailments not given medical treatment by reason								untreated spells of ailment		spells of ailment	
				no medical facilities available in the neighbour-hood	facilities available but no treatment sought owing to					n.r.	total	estd. no. (00)	sample	estd. no. (00)	sample
	govern-ment	private	all		lack of faith	long waiting	financial reason	ailment not considered serious	others						
(1)	(2)	(3)	(4)	(5)	(6)	(7)	(8)	(9)	(10)	(11)	(12)	(13)	(14)	(15)	(16)
0 – 235	221	542	763	158	39	0	416	160	142	84	1000	8040	271	33898	1197
235 – 265	200	603	803	68	58	2	356	305	99	112	1000	4921	199	24985	911
265 – 320	203	589	792	60	32	7	373	297	111	121	1000	12808	491	61704	2323
320 – 365	205	588	793	75	19	2	333	322	166	83	1000	14535	542	70170	3001
365 – 410	177	638	814	110	22	3	382	278	118	87	1000	12809	456	68918	2667
410 – 460	188	631	819	129	16	15	298	324	118	100	1000	14538	549	80540	3262
460 – 520	173	673	847	103	36	18	198	390	169	87	1000	15149	631	98766	4123
520 – 605	173	667	840	130	41	22	245	318	153	92	1000	15924	653	99274	4301
605 – 730	182	670	852	91	26	4	202	444	146	88	1000	15498	678	104940	4758
730 – 980	170	708	878	52	13	17	191	440	183	104	1000	15291	648	125403	5657
980 – 1285	191	688	880	122	40	18	150	433	133	104	1000	7418	282	61612	2630
1285 +	140	771	912	37	19	2	76	518	144	205	1000	8198	357	92658	3973
all	180	663	843	95	28	10	266	355	144	101	1000	145129	5757	922867	38803
social group:															
ST	250	526	776	199	9	3	311	219	166	93	1000	10433	599	46597	2594
SC	208	603	812	115	24	4	364	300	125	68	1000	33042	1124	175509	6487
OBC	173	672	844	67	32	10	251	384	146	110	1000	55687	2195	357982	14909
others	164	702	866	91	30	17	205	392	149	116	1000	45967	1839	342779	14813
all	180	663	843	95	28	10	266	355	144	101	1000	145129	5757	922867	38803

For peer review only - http://bmjopen.bmj.com/site/about/guidelines.xhtml

Appendix A

Table (42): Number of spells of ailments receiving treatment from non- government sources per 1000 of treated spells during last 15 days and their distribution by reason for not taking treatment from govt. sources for each mpce class

All-India | Rural | Males

mpce class	no. of spells of ailment receiving treatment from non-govt. sources per 1000 treated spells	spells of ailment receiving treatment from non-govt. sources by reason for not using government sources							spells of ailment receiving treatment from non-govt. sources	
		government doctor/ facilities too far	not satisfied with medical treatment by government doctor/ facilities	long waiting	required specific services not available	others	n.r.	total	estd. no. (00)	sample
(1)	(2)	(3)	(4)	(5)	(6)	(7)	(8)	(9)	(10)	(11)
0 – 225	719	246	260	42	36	205	212	1000	7478	217
225 – 255	783	233	356	57	34	139	181	1000	5955	168
255 – 300	764	279	255	43	51	210	162	1000	13971	427
300 – 340	725	254	353	26	38	160	169	1000	12042	384
340 – 380	776	237	410	98	37	105	114	1000	17158	544
380 – 420	777	258	377	65	61	115	122	1000	18691	640
420 – 470	765	254	402	55	34	122	133	1000	20330	707
470 – 525	825	174	430	98	40	130	128	1000	23415	813
525 – 615	784	211	438	89	55	121	87	1000	23050	847
615 – 775	798	185	474	82	53	120	87	1000	28733	1050
775 – 950	754	122	450	123	84	137	84	1000	14882	539
950 +	843	141	493	110	76	115	65	1000	20209	796
all	783	210	411	79	52	133	116	1000	205915	7132
spells receiving treatment from non govt sources: estd no. (00)	X	43144	84548	16328	10616	27285	23952	205874	X	X
sample	X	1293	3022	558	411	989	857	7130	X	X

For peer review only - http://bmjopen.bmj.com/site/about/guidelines.xhtml

Appendix A

Table (42): Number of spells of ailments receiving treatment from non- government sources per 1000 of treated spells during last 15 days and their distribution by reason for not taking treatment from govt. sources for each mpce class

All-India | Rural | Females

mpce class	no. of spells of ailment receiving treatment from non-govt. sources per 1000 treated spells	spells of ailment receiving treatment from non-govt. sources by reason for not using government sources							spells of ailment receiving treatment from non-govt. sources	
		government doctor/ facilities too far	not satisfied with medical treatment by government doctor/ facilities	long waiting	required specific services not available	others	n.r.	total	estd. no. (00)	sample
(1)	(2)	(3)	(4)	(5)	(6)	(7)	(8)	(9)	(10)	(11)
0 – 225	689	211	273	8	50	205	252	1000	7452	221
225 – 255	688	330	300	37	38	162	133	1000	4813	164
255 – 300	723	281	322	37	37	128	195	1000	13639	422
300 – 340	752	224	386	81	46	120	142	1000	12473	419
340 – 380	797	258	334	100	67	116	126	1000	18158	605
380 – 420	784	198	346	64	71	174	146	1000	19555	644
420 – 470	791	302	349	68	34	111	136	1000	23137	756
470 – 525	773	203	422	69	47	149	109	1000	24278	816
525 – 615	763	198	433	95	34	121	118	1000	22336	817
615 – 775	775	152	495	112	39	115	88	1000	28268	1085
775 – 950	784	183	463	113	54	124	64	1000	18147	618
950 +	804	134	465	134	102	104	62	1000	24230	859
all	771	210	403	86	53	129	119	1000	216486	7426
spells receiving treatment from non govt sources:										
estd no. (00)	X	45555	87172	18654	11488	27933	25684	216486	X	X
sample	X	1359	3025	597	424	1051	970	7426	X	X

For peer review only - http://bmjopen.bmj.com/site/about/guidelines.xhtml

Appendix A

Table (42): Number of spells of ailments receiving treatment from non- government sources per 1000 of treated spells during last 15 days and their distribution by reason for not taking treatment from govt. sources for each mpce class

All-India Rural Persons

mpce class	no. of spells of ailment receiving treatment from non-govt. sources per 1000 treated spells	spells of ailment receiving treatment from non-govt. sources by reason for not using government sources							spells of ailment receiving treatment from non-govt. sources	
		government doctor/ facilities too far	not satisfied with medical treatment by government doctor/ facilities	long waiting	required specific services not available	others	n.r.	total	estd. no. (00)	sample
(1)	(2)	(3)	(4)	(5)	(6)	(7)	(8)	(9)	(10)	(11)
0 – 225	703	228	266	25	43	205	232	1000	14931	438
225 – 255	738	276	331	48	36	149	160	1000	10768	332
255 – 300	743	280	288	40	44	169	178	1000	27609	849
300 – 340	738	239	370	54	42	140	156	1000	24515	803
340 – 380	786	248	371	99	52	110	120	1000	35316	1149
380 – 420	781	228	362	64	66	145	135	1000	38245	1284
420 – 470	779	279	374	62	34	116	135	1000	43466	1463
470 – 525	798	189	426	83	43	140	118	1000	47693	1629
525 – 615	773	205	435	92	45	121	102	1000	45386	1664
615 – 775	786	168	484	97	46	117	87	1000	57001	2135
775 – 950	770	155	457	118	68	129	73	1000	33030	1157
950 +	821	137	477	123	91	109	63	1000	44439	1655
all	777	210	407	83	52	131	118	1000	422401	14558
spells receiving treatment from non govt sources: estd no. (00)	X	88699	171720	34982	22104	55219	49636	422359	X	X
sample	X	2652	6047	1155	835	2040	1827	14556	X	X

For peer review only - http://bmjopen.bmj.com/site/about/guidelines.xhtml

Appendix A

Table (42): Number of spells of ailments receiving treatment from non- government sources per 1000 of treated spells during last 15 days and their distribution by reason for not taking treatment from govt. sources for each mpce class

All-India | Urban | Males

mpce class	no. of spells of ailment receiving treatment from non-govt. sources per 1000 treated spells	spells of ailment receiving treatment from non-govt. sources by reason for not using government sources							spells of ailment receiving treatment from non-govt. sources	
		government doctor/ facilities too far	not satisfied with medical treatment by government doctor/ facilities	long waiting	required specific services not available	others	n.r.	total	estd. no. (00)	sample
(1)	(2)	(3)	(4)	(5)	(6)	(7)	(8)	(9)	(10)	(11)
0 –300	741	163	432	138	20	138	110	1000	1943	84
300 – 350	788	49	704	57	21	92	77	1000	2191	79
350 – 425	769	136	479	207	39	76	64	1000	4436	212
425 – 500	765	102	440	176	41	116	124	1000	8231	440
500 – 575	754	186	385	128	52	106	142	1000	4135	230
575 – 665	705	144	394	183	37	181	61	1000	5192	312
665 – 775	832	95	453	155	58	169	70	1000	10682	525
775 – 915	764	144	467	140	38	144	67	1000	8509	493
915 – 1120	789	174	449	178	12	115	71	1000	11738	635
1120 – 1500	870	100	519	146	32	138	65	1000	16601	780
1500 – 1925	808	67	446	228	19	210	28	1000	4259	281
1925 +	897	118	390	133	46	280	33	1000	12354	569
all	808	123	456	158	36	157	70	1000	90271	4640
spells receiving treatment from non govt sources:										
estd no. (00)	X	11083	41188	14220	3251	14170	6360	90271	X	X
sample	X	422	2170	752	215	713	368	4640	X	X

For peer review only - http://bmjopen.bmj.com/site/about/guidelines.xhtml

Appendix A

Table (42): Number of spells of ailments receiving treatment from non- government sources per 1000 of treated spells during last 15 days and their distribution by reason for not taking treatment from govt. sources for each mpce class

All-India Urban Females

mpce class	no. of spells of ailment receiving treatment from non-govt. sources per 1000 treated spells	spells of ailment receiving treatment from non-govt. sources by reason for not using government sources							spells of ailment receiving treatment from non-govt. sources	
		gover-nment doctor/ facili-ties too far	not satisfied with medical treatment by government doctor/ facilities	long waiting	required specific services not available	others	n.r.	total	estd. no. (00)	sample
(1)	(2)	(3)	(4)	(5)	(6)	(7)	(8)	(9)	(10)	(11)
0 –300	740	76	211	177	59	325	152	1000	1489	69
300 – 350	784	42	537	67	25	202	127	1000	2106	87
350 – 425	722	100	403	217	25	106	150	1000	4312	234
425 – 500	729	174	440	152	65	95	74	1000	8501	470
500 – 575	785	167	396	108	31	205	93	1000	4490	254
575 – 665	766	225	371	207	34	118	44	1000	7383	378
665 – 775	825	118	433	124	37	132	156	1000	12336	618
775 – 915	805	112	511	161	39	134	42	1000	9994	565
915 – 1120	830	157	456	179	22	146	40	1000	13179	724
1120 – 1500	817	121	496	183	44	113	44	1000	15157	884
1500 – 1925	847	116	448	185	41	181	29	1000	5110	330
1925 +	885	199	383	165	35	170	48	1000	14678	675
all	809	147	439	165	38	142	70	1000	98737	5288
spells receiving treatment from non govt sources:										
estd no. (00)	X	14523	43330	16293	3714	13973	6883	98715	X	X
sample	X	583	2436	863	234	805	366	5287	X	X

For peer review only - http://bmjopen.bmj.com/site/about/guidelines.xhtml

Table (42): Number of spells of ailments receiving treatment from non- government sources per 1000 of treated spells during last 15 days and their distribution by reason for not taking treatment from govt. sources for each mpce class

All-India Urban Persons

mpce class	no. of spells of ailment receiving treatment from non-govt. sources per 1000 treated spells	spells of ailment receiving treatment from non-govt. sources by reason for not using government sources							spells of ailment receiving treatment from non-govt. sources	
		government doctor/ facilities too far	not satisfied with medical treatment by government doctor/ facilities	long waiting	required specific services not available	others	n.r.	total	estd. no. (00)	sample
(1)	(2)	(3)	(4)	(5)	(6)	(7)	(8)	(9)	(10)	(11)
0 –300	741	125	336	155	37	219	128	1000	3431	153
300 – 350	786	46	622	62	23	146	101	1000	4298	166
350 – 425	745	118	441	212	32	91	106	1000	8748	446
425 – 500	746	139	440	164	53	105	99	1000	16732	910
500 – 575	770	176	391	118	41	158	117	1000	8625	484
575 – 665	740	192	381	197	35	144	51	1000	12575	690
665 – 775	828	107	442	139	47	149	116	1000	23018	1143
775 – 915	785	127	491	152	38	139	54	1000	18504	1058
915 – 1120	810	165	453	179	17	132	55	1000	24917	1359
1120 – 1500	844	110	508	163	37	126	55	1000	31758	1664
1500 – 1925	829	94	447	205	31	194	29	1000	9369	611
1925 +	891	162	386	150	40	220	41	1000	27032	1244
all	808	135	447	161	37	149	70	1000	189008	9928
spells receiving treatment from non govt sources:										
estd no. (00)	X	25606	84517	30512	6965	28143	13243	188986	X	X
sample	X	1005	4606	1615	449	1518	734	9927	X	X

For peer review only - http://bmjopen.bmj.com/site/about/guidelines.xhtml

Appendix A

Table (42): Number of spells of ailments receiving treatment from non- government sources per 1000 of treated spells during last 15 days and their distribution by reason for not taking treatment from govt. sources for each mpce class

All-India — Rural + Urban — Males

mpce class	no. of spells of ailment receiving treatment from non-govt. sources per 1000 treated spells	spells of ailment receiving treatment from non-govt. sources by reason for not using government sources: government doctor/ facilities too far	not satisfied with medical treatment by government doctor/ facilities	long waiting	required specific services not available	others	n.r.	total	spells of ailment receiving treatment from non-govt. sources: estd. no. (00)	sample
(1)	(2)	(3)	(4)	(5)	(6)	(7)	(8)	(9)	(10)	(11)
0 – 235	723	228	295	61	32	191	191	1000	9421	301
235 – 265	785	183	450	57	31	126	153	1000	8146	247
265 – 320	765	244	309	83	48	177	138	1000	18407	639
320 – 365	740	192	388	87	39	142	151	1000	20273	824
365 – 410	771	227	405	104	40	105	119	1000	21293	774
410 – 460	760	234	381	91	56	130	109	1000	23883	952
460 – 520	787	199	420	90	42	139	111	1000	31012	1232
520 – 605	808	166	440	109	39	134	112	1000	31925	1306
605 – 730	786	199	442	119	41	119	82	1000	34788	1482
730 – 980	823	154	490	105	45	127	79	1000	45334	1830
980 – 1285	766	110	449	147	70	153	72	1000	19141	820
1285 +	863	132	454	119	65	178	53	1000	32563	1365
all	790	183	425	103	47	140	102	1000	296186	11772
spells receiving treatment from non govt sources:										
estd no. (00)	X	54227	125736	30547	13867	41456	30312	296145	X	X
sample	X	1715	5192	1310	626	1702	1225	11770	X	X

For peer review only - http://bmjopen.bmj.com/site/about/guidelines.xhtml

Table (42): Number of spells of ailments receiving treatment from non- government sources per 1000 of treated spells during last 15 days and their distribution by reason for not taking treatment from govt. sources for each mpce class

All-India — Rural + Urban — Females

mpce class	no. of spells of ailment receiving treatment from non-govt. sources per 1000 treated spells	spells of ailment receiving treatment from non-govt. sources by reason for not using government sources							spells of ailment receiving treatment from non-govt. sources	
		gover-nment doctor/ facili-ties too far	not satisfied with medical treatment by government doctor/ facilities	long waiting	required specific services not available	others	n.r.	total	estd. no. (00)	sample
(1)	(2)	(3)	(4)	(5)	(6)	(7)	(8)	(9)	(10)	(11)
0 – 235	697	189	263	37	51	225	235	1000	8941	290
235 – 265	715	242	372	46	34	174	132	1000	6919	251
265 – 320	723	238	341	81	34	123	184	1000	17951	656
320 – 365	742	204	408	110	54	110	115	1000	20974	889
365 – 410	795	240	346	102	60	133	119	1000	22648	859
410 – 460	779	206	353	103	61	158	118	1000	26938	1022
460 – 520	802	238	379	87	35	118	143	1000	35473	1374
520 – 605	782	177	448	96	44	145	90	1000	34272	1381
605 – 730	786	183	441	127	30	130	89	1000	35515	1541
730 – 980	789	141	496	136	41	114	72	1000	43425	1969
980 – 1285	797	168	459	129	51	136	56	1000	23258	948
1285 +	833	158	434	146	77	129	56	1000	38908	1534
all	782	191	414	111	48	133	103	1000	315222	12714
spells receiving treatment from non govt sources:										
estd no. (00)	X	60078	130501	34947	15202	41906	32566	315200	X	X
sample	X	1942	5461	1460	658	1856	1336	12713	X	X

For peer review only - http://bmjopen.bmj.com/site/about/guidelines.xhtml

Table (42): Number of spells of ailments receiving treatment from non- government sources per 1000 of treated spells during last 15 days and their distribution by reason for not taking treatment from govt. sources for each mpce class

All-India Rural + Urban Persons

mpce class	no. of spells of ailment receiving treatment from non-govt. sources per 1000 treated spells	spells of ailment receiving treatment from non-govt. sources by reason for not using government sources							spells of ailment receiving treatment from non-govt. sources	
		government doctor/ facilities too far	not satisfied with medical treatment by government doctor/ facilities	long waiting	required specific services not available	others	n.r.	total	estd. no. (00)	sample
(1)	(2)	(3)	(4)	(5)	(6)	(7)	(8)	(9)	(10)	(11)
0 – 235	710	209	279	49	42	208	213	1000	18362	591
235 – 265	751	210	414	52	32	148	143	1000	15066	498
265 – 320	744	241	325	82	41	150	161	1000	36357	1295
320 – 365	741	198	399	98	47	126	132	1000	41247	1713
365 – 410	783	234	375	103	50	120	119	1000	43942	1633
410 – 460	770	219	366	97	59	145	114	1000	50821	1974
460 – 520	795	220	398	88	38	128	128	1000	66485	2606
520 – 605	794	172	444	102	42	139	100	1000	66197	2687
605 – 730	786	191	441	123	35	125	85	1000	70302	3023
730 – 980	806	148	493	120	43	120	76	1000	88759	3799
980 – 1285	782	142	455	137	59	144	63	1000	42399	1768
1285 +	846	147	443	133	71	151	55	1000	71472	2899
all	786	187	419	107	48	136	103	1000	611408	24486
spells receiving treatment from non govt sources:										
estd no. (00)	X	114305	256237	65494	29069	83362	62879	611345	X	X
sample	X	3657	10653	2770	1284	3558	2561	24483	X	X

For peer review only

For peer review only - http://bmjopen.bmj.com/site/about/guidelines.xhtml

Appendix A

Table (43): Number per 1000 untreated spells of ailment reporting measures taken for recovery/relief (other than medical treatment), per 1000 distribution of such spells of ailments by measure taken for recovery/relief and average expenditure incurred for such measures for recovery/relief separately for each mpce class

All-India | Rural | Males

mpce class	no. per 1000 of spells of ailment reporting recovery /relief measure other than medical treatment	measure taken for recovery/relief other than medical advice — whom consulted: self/other household member/ friend	medicine shop	others	n.r.	total	av. exp. incurred on recovery / relief measure other than medical consul-tation (Rs. 0.0)	untreated spells of ailment during the last 15 days for which some relief measures taken: estd. no. (00)	sample	untreated spells of ailment during the last 15 days: estd. no. (00)	sample
(1)	(2)	(3)	(4)	(5)	(6)	(7)	(8)	(9)	(10)	(11)	(12)
0 – 225	545	156	367	477	0	1000	39.3	2123	47	3897	108
225 – 255	444	114	404	482	0	1000	13.7	822	29	1852	73
255 – 300	525	148	375	476	0	1000	21.7	2593	79	4937	171
300 – 340	602	308	389	303	0	1000	31.3	3158	73	5244	155
340 – 380	421	196	387	417	0	1000	20.8	2226	69	5294	177
380 – 420	518	194	449	357	1	1000	57.9	3234	86	6238	198
420 – 470	495	217	411	372	0	1000	29.1	2760	89	5575	208
470 – 525	650	307	206	484	3	1000	44.9	4703	127	7239	247
525 – 615	553	179	457	365	0	1000	31.3	2897	113	5237	241
615 – 775	546	337	406	257	0	1000	20	2643	83	4845	194
775 – 950	452	297	228	475	0	1000	36.3	1297	39	2873	100
950 +	496	591	331	78	0	1000	161	1492	46	3009	115
all	533	252	364	383	1	1000	40.2	29950	880	56241	1987

For peer review only - http://bmjopen.bmj.com/site/about/guidelines.xhtml

Appendix A

Table (43): Number per 1000 untreated spells of ailment reporting measures taken for recovery/relief (other than medical treatment), per 1000 distribution of such spells of ailments by measure taken for recovery/relief and average expenditure incurred for such measures for recovery/relief separately for each mpce class

All-India Rural Females

mpce class	no. per 1000 of spells of ailment reporting recovery /relief measure other than medical treatment	measure taken for recovery/relief other than medical advice: whom consulted: self/other household member/ friend	medicine shop	others	n.r.	total	av. exp. incurred on recovery / relief measure other than medical consul-tation (Rs. 0.0)	untreated spells of ailment during the last 15 days for which some relief measures taken: estd. no. (00)	sample	untreated spells of ailment during the last 15 days: estd. no. (00)	sample
(1)	(2)	(3)	(4)	(5)	(6)	(7)	(8)	(9)	(10)	(11)	(12)
0 – 225	604	252	393	355	0	1000	21.6	1722	35	2853	83
225 – 255	322	322	136	543	0	1000	14	737	26	2291	82
255 – 300	501	224	176	600	0	1000	28.5	2883	81	5756	187
300 – 340	499	311	358	330	0	1000	17.9	2716	70	5439	162
340 – 380	505	217	325	458	0	1000	29.6	3139	71	6210	173
380 – 420	525	267	237	496	0	1000	37.8	2941	76	5599	176
420 – 470	664	243	371	385	0	1000	51.8	4432	133	6678	227
470 – 525	614	217	292	491	0	1000	33.5	3924	101	6386	224
525 – 615	584	281	406	283	30	1000	25.6	4030	124	6900	248
615 – 775	524	263	455	282	0	1000	19.9	3183	95	6079	218
775 – 950	500	253	405	341	0	1000	29.4	1562	44	3127	110
950 +	372	317	241	442	0	1000	27.6	1285	47	3450	134
all	536	256	332	409	4	1000	29.5	32554	903	60768	2024

For peer review only - http://bmjopen.bmj.com/site/about/guidelines.xhtml

Appendix A

Table (43): Number per 1000 untreated spells of ailment reporting measures taken for recovery/relief (other than medical treatment), per 1000 distribution of such spells of ailments by measure taken for recovery/relief and average expenditure incurred for such measures for recovery/relief separately for each mpce class

All-India | Rural | Persons

mpce class	no. per 1000 of spells of ailment reporting recovery /relief measure other than medical treatment	measure taken for recovery/relief other than medical advice: whom consulted					av. exp. incurred on recovery / relief measure other than medical consul-tation (Rs. 0.0)	untreated spells of ailment during the last 15 days for which some relief measures taken		untreated spells of ailment during the last 15 days	
		self/other household member/ friend	medicine shop	others	n.r.	total		estd. no. (00)	sample	estd. no. (00)	sample
(1)	(2)	(3)	(4)	(5)	(6)	(7)	(8)	(9)	(10)	(11)	(12)
0 – 225	570	199	378	423	0	1000	31.8	3845	82	6750	191
225 – 255	376	212	277	511	0	1000	13.9	1559	55	4143	155
255 – 300	512	188	270	541	0	1000	25.4	5476	160	10693	358
300 – 340	550	310	375	316	0	1000	24.5	5874	143	10683	317
340 – 380	466	208	351	441	0	1000	25.6	5365	140	11505	350
380 – 420	522	229	348	423	1	1000	48.4	6175	162	11837	374
420 – 470	587	233	387	380	0	1000	41.5	7192	222	12253	435
470 – 525	633	266	245	487	2	1000	39.5	8627	228	13625	471
525 – 615	571	238	427	317	18	1000	28.1	6927	237	12137	489
615 – 775	533	297	433	271	0	1000	19.9	5826	178	10925	412
775 – 950	477	273	325	402	0	1000	32.7	2859	83	6000	210
950 +	430	464	289	247	0	1000	89.8	2777	93	6459	249
all	534	254	347	396	2	1000	34.6	62503	1783	117009	4011

For peer review only - http://bmjopen.bmj.com/site/about/guidelines.xhtml

Appendix A

Table (43): Number per 1000 untreated spells of ailment reporting measures taken for recovery/relief (other than medical treatment), per 1000 distribution of such spells of ailments by measure taken for recovery/relief and average expenditure incurred for such measures for recovery/relief separately for each mpce class

All-India Urban Males

mpce class	no. per 1000 of spells of ailment reporting recovery /relief measure other than medical treatment	measure taken for recovery/relief other than medical advice: whom consulted: self/other household member/ friend	medicine shop	others	n.r.	total	av. exp. incurred on recovery / relief measure other than medical consul-tation (Rs. 0.0)	untreated spells of ailment during the last 15 days for which some relief measures taken: estd. no. (00)	sample	untreated spells of ailment during the last 15 days: estd. no. (00)	sample
(1)	(2)	(3)	(4)	(5)	(6)	(7)	(8)	(9)	(10)	(11)	(12)
0 –300	642	399	465	136	0	1000	20.5	329	16	512	33
300 – 350	607	514	449	37	0	1000	12.5	285	10	469	18
350 – 425	478	417	261	322	0	1000	32.9	439	21	919	66
425 – 500	475	78	697	225	0	1000	13.2	840	35	1766	108
500 – 575	284	45	701	253	0	1000	9.2	157	19	552	53
575 – 665	564	201	541	259	0	1000	48.7	722	39	1281	80
665 – 775	395	491	450	59	0	1000	9.1	477	22	1206	90
775 – 915	518	356	585	59	0	1000	14.9	552	29	1067	81
915 – 1120	684	463	500	37	0	1000	76.2	1005	40	1468	82
1120 – 1500	493	342	530	128	0	1000	15.6	979	44	1984	108
1500 – 1925	809	710	271	19	0	1000	19.7	521	17	644	32
1925 +	520	764	49	187	0	1000	39.6	485	22	934	60
all	530	390	472	138	0	1000	27.8	6790	314	12802	811

For peer review only - http://bmjopen.bmj.com/site/about/guidelines.xhtml

Appendix A

Table (43): Number per 1000 untreated spells of ailment reporting measures taken for recovery/relief (other than medical treatment), per 1000 distribution of such spells of ailments by measure taken for recovery/relief and average expenditure incurred for such measures for recovery/relief separately for each mpce class

All-India Urban Females

mpce class	no. per 1000 of spells of ailment reporting recovery /relief measure other than medical treatment	measure taken for recovery/relief other than medical advice: whom consulted: self/other household member/ friend	medicine shop	others	n.r.	total	av. exp. incurred on recovery / relief measure other than medical consul-tation (Rs. 0.0)	untreated spells of ailment during the last 15 days for which some relief measures taken: estd. no. (00)	sample	untreated spells of ailment during the last 15 days: estd. no. (00)	sample
(1)	(2)	(3)	(4)	(5)	(6)	(7)	(8)	(9)	(10)	(11)	(12)
0 –300	303	0	756	244	0	1000	5.9	235	15	777	47
300 – 350	304	17	720	263	0	1000	12.2	94	10	309	26
350 – 425	689	269	722	10	0	1000	7.4	824	31	1196	67
425 – 500	465	94	680	226	0	1000	17.3	970	45	2085	117
500 – 575	381	103	879	18	0	1000	9.9	286	23	752	53
575 – 665	498	169	721	110	0	1000	29	707	41	1420	95
665 – 775	432	415	375	210	0	1000	18.9	730	37	1690	106
775 – 915	369	245	708	47	0	1000	9.2	455	31	1232	101
915 – 1120	562	463	428	109	0	1000	12.1	1064	42	1893	107
1120 – 1500	633	677	297	26	0	1000	17.9	1507	56	2382	128
1500 – 1925	635	583	354	63	0	1000	16.3	492	18	775	40
1925 +	688	868	81	51	0	1000	30.3	555	23	806	48
all	517	399	503	98	0	1000	16.2	7919	372	15318	935

For peer review only - http://bmjopen.bmj.com/site/about/guidelines.xhtml

Appendix A

Table (43): Number per 1000 untreated spells of ailment reporting measures taken for recovery/relief (other than medical treatment), per 1000 distribution of such spells of ailments by measure taken for recovery/relief and average expenditure incurred for such measures for recovery/relief separately for each mpce class

All-India Urban Persons

mpce class	no. per 1000 of spells of ailment reporting recovery /relief measure other than medical treatment	measure taken for recovery/relief other than medical advice: whom consulted					av. exp. incurred on recovery / relief measure other than medical consul-tation (Rs. 0.0)	untreated spells of ailment during the last 15 days for which some relief measures taken		untreated spells of ailment during the last 15 days	
		self/other household member/ friend	medicine shop	others	n.r.	total		estd. no. (00)	sample	estd. no. (00)	sample
(1)	(2)	(3)	(4)	(5)	(6)	(7)	(8)	(9)	(10)	(11)	(12)
0 –300	438	233	586	181	0	1000	11.7	564	31	1289	80
300 – 350	486	390	517	93	0	1000	12.4	379	20	779	44
350 – 425	597	320	561	118	0	1000	18.5	1263	52	2115	133
425 – 500	470	87	688	225	0	1000	15.4	1810	80	3852	225
500 – 575	340	83	816	101	0	1000	9.6	443	42	1304	106
575 – 665	529	185	630	185	0	1000	38.3	1430	80	2701	175
665 – 775	417	445	405	150	0	1000	14.8	1206	59	2896	196
775 – 915	438	306	641	53	0	1000	11.9	1007	60	2299	182
915 – 1120	615	463	463	74	0	1000	40.1	2069	82	3361	189
1120 – 1500	569	545	389	66	0	1000	16.8	2486	100	4366	236
1500 – 1925	714	648	312	40	0	1000	17.8	1012	35	1419	72
1925 +	598	819	66	115	0	1000	35.3	1040	45	1739	108
all	523	395	489	117	0	1000	21.5	14710	686	28120	1746

For peer review only - http://bmjopen.bmj.com/site/about/guidelines.xhtml

Appendix A

Table (43): Number per 1000 untreated spells of ailment reporting measures taken for recovery/relief (other than medical treatment), per 1000 distribution of such spells of ailments by measure taken for recovery/relief and average expenditure incurred for such measures for recovery/relief separately for each mpce class

All-India | Rural +Urban | Males

mpce class	no. per 1000 of spells of ailment reporting recovery /relief measure other than medical treatment	measure taken for recovery/relief other than medical advice: whom consulted: self/other household member/ friend	medicine shop	others	n.r.	total	av. exp. incurred on recovery / relief measure other than medical consul-tation (Rs. 0.0)	untreated spells of ailment during the last 15 days for which some relief measures taken: estd. no. (00)	sample	untreated spells of ailment during the last 15 days: estd. no. (00)	sample
(1)	(2)	(3)	(4)	(5)	(6)	(7)	(8)	(9)	(10)	(11)	(12)
0 – 235	556	189	380	432	0	1000	37.1	2452	63	4410	141
235 – 265	477	217	415	367	0	1000	13.5	1107	39	2321	91
265 – 320	518	187	359	454	0	1000	23.4	3033	100	5856	237
320 – 365	570	260	453	287	0	1000	26.8	3997	108	7010	263
365 – 410	408	186	408	407	0	1000	19.7	2383	88	5846	230
410 – 460	526	195	466	339	1	1000	56.4	3956	125	7519	278
460 – 520	477	257	417	326	0	1000	25.6	3237	111	6781	298
520 – 605	633	312	246	439	3	1000	41	5256	156	8306	328
605 – 730	582	252	468	280	0	1000	41.1	3902	153	6705	323
730 – 980	530	338	439	222	0	1000	18.7	3622	127	6829	302
980 – 1285	517	415	240	345	0	1000	33.3	1818	56	3517	132
1285 +	502	634	262	105	0	1000	132.3	1977	68	3942	175
all	532	278	384	337	0	1000	37.9	36740	1194	69043	2798

For peer review only - http://bmjopen.bmj.com/site/about/guidelines.xhtml

Appendix A

Table (43): Number per 1000 untreated spells of ailment reporting measures taken for recovery/relief (other than medical treatment), per 1000 distribution of such spells of ailments by measure taken for recovery/relief and average expenditure incurred for such measures for recovery/relief separately for each mpce class

All-India Rural +Urban Females

mpce class	no. per 1000 of spells of ailment reporting recovery /relief measure other than medical treatment	measure taken for recovery/relief other than medical advice: whom consulted: self/other household member/ friend	medicine shop	others	n.r.	total	av. exp. incurred on recovery / relief measure other than medical consul-tation (Rs. 0.0)	untreated spells of ailment during the last 15 days for which some relief measures taken: estd. no. (00)	sample	untreated spells of ailment during the last 15 days: estd. no. (00)	sample
(1)	(2)	(3)	(4)	(5)	(6)	(7)	(8)	(9)	(10)	(11)	(12)
0 – 235	539	222	436	342	0	1000	18.2	1958	50	3630	130
235 – 265	319	287	202	511	0	1000	13.8	831	36	2600	108
265 – 320	533	234	297	469	0	1000	24.9	3707	112	6952	254
320 – 365	490	254	443	303	0	1000	17.7	3687	115	7525	279
365 – 410	492	208	372	421	0	1000	27.5	3425	94	6963	226
410 – 460	520	248	331	421	0	1000	36	3648	117	7019	271
460 – 520	617	268	372	360	0	1000	45.2	5161	170	8367	333
520 – 605	575	220	335	444	0	1000	29.5	4379	132	7618	325
605 – 730	579	319	411	246	24	1000	22.7	5095	166	8793	355
730 – 980	554	396	404	199	0	1000	19.3	4690	151	8461	346
980 – 1285	526	332	393	275	0	1000	26.8	2054	62	3901	150
1285 +	432	483	193	325	0	1000	28.1	1840	70	4256	182
all	532	284	365	348	3	1000	26.9	40473	1275	76086	2959

For peer review only - http://bmjopen.bmj.com/site/about/guidelines.xhtml

Appendix A

Table (43): Number per 1000 untreated spells of ailment reporting measures taken for recovery/relief (other than medical treatment), per 1000 distribution of such spells of ailments by measure taken for recovery/relief and average expenditure incurred for such measures for recovery/relief separately for each mpce class

All-India Rural +Urban Persons

mpce class	no. per 1000 of spells of ailment reporting recovery /relief measure other than medical treatment	measure taken for recovery/relief other than medical advice: whom consulted: self/other household member/ friend	medicine shop	others	n.r.	total	av. exp. incurred on recovery / relief measure other than medical consul-tation (Rs. 0.0)	untreated spells of ailment during the last 15 days for which some relief measures taken: estd. no. (00)	sample	untreated spells of ailment during the last 15 days: estd. no. (00)	sample
(1)	(2)	(3)	(4)	(5)	(6)	(7)	(8)	(9)	(10)	(11)	(12)
0 – 235	548	203	405	392	0	1000	28.6	4409	113	8040	271
235 – 265	394	247	324	429	0	1000	13.6	1937	75	4921	199
265 – 320	526	213	325	462	0	1000	24.2	6740	212	12808	491
320 – 365	529	257	448	294	0	1000	22.1	7684	223	14535	542
365 – 410	453	199	386	415	0	1000	23.9	5808	182	12809	456
410 – 460	523	220	401	378	0	1000	46.5	7604	242	14538	549
460 – 520	554	264	389	347	0	1000	36.4	8398	281	15149	631
520 – 605	605	270	287	442	1	1000	35.5	9634	288	15924	653
605 – 730	580	290	435	261	14	1000	30.7	8996	319	15498	678
730 – 980	544	371	420	209	0	1000	19	8312	278	15291	648
980 – 1285	522	371	321	308	0	1000	29.9	3872	118	7418	282
1285 +	466	561	228	211	0	1000	78.2	3817	138	8198	357
all	532	281	374	343	2	1000	32.1	77213	2469	145129	5757

For peer review only - http://bmjopen.bmj.com/site/about/guidelines.xhtml

Appendix A

Table (44): Average medical expenditure (Rs.) for treatment (other than as inpatient) under different heads per ailing person medically treated during last 15 days by mpce class

all-India Rural Males

mpce class	average medical expenditure (Rs.) for treatment under different heads for treatment other than as inpatient incurred on														estd. expn. (000)	persons treated medically on payment during last 15 days (00)		persons treated medically during last 15 days	
	services						goods					total (govt.)	total (pvt.)	total		estd. (00)	sam-ple	estd. (00)	sam-ple
	doctor's/ surgeons fee		diag-nostic test	atten-dant char-ges	physio the-rapy	other services	medicines		perso-nal medi-cal applia-nces	food etc.	blood oxy-gen, etc.								
	hospi-tal staff	other specia-list					from hos-pital	from out-side											
(1)	(2)	(3)	(4)	(5)	(6)	(7)	(8)	(9)	(10)	(11)	(12)	(13)	(14)	(15)	(16)	(17)	(18)	(19)	(20)
0 – 225	10	3	5	0	0	0	8	88	0	3	0	5	111	116	82862	7076	233	10079	334
225 – 255	5	8	6	0	0	0	2	106	0	12	0	3	133	136	74433	5334	160	7463	230
255 – 300	8	5	9	0	0	0	5	116	0	6	0	5	145	150	182276	12112	402	17461	579
300 – 340	8	4	3	0	0	0	7	99	0	3	0	6	119	125	138285	11103	389	16024	557
340 – 380	10	13	5	1	0	0	3	142	0	4	2	18	163	181	283097	15624	517	21552	725
380 – 420	11	4	7	0	0	2	7	152	0	4	0	11	175	187	328008	17502	627	23503	848
420 – 470	17	11	7	0	0	1	18	143	2	3	0	8	194	202	371375	18262	699	25656	949
470 – 525	11	11	8	0	0	2	24	150	4	6	0	14	200	215	445216	20695	768	27510	1044
525 – 615	16	11	14	0	0	0	24	139	0	5	0	5	203	208	439401	21021	818	27930	1109
615 – 775	16	11	19	0	0	0	16	159	0	2	0	4	218	222	571059	25668	1028	34167	1365
775 – 950	19	11	10	0	0	1	17	155	0	2	0	4	211	215	285511	13262	504	18481	708
950 +	17	9	19	0	0	1	28	228	4	3	1	5	302	308	504638	16385	680	21901	942
all classes	13	9	10	0	0	1	15	147	1	4	0	8	193	201	3706161	184044	6825	251729	9390
social group																			
ST	6	7	5	0	0	0	8	114	0	6	0	4	142	146	152801	10457	544	14244	763
SC	11	7	8	0	0	1	15	121	1	4	0	6	161	167	657295	39292	1319	52606	1782
OBC	16	7	10	0	0	1	16	147	1	4	0	7	194	201	1513312	74955	2750	104030	3848
others	13	14	13	0	0	1	16	170	2	3	1	11	221	232	1382747	59339	2211	80849	2996
all	13	9	10	0	0	1	15	147	1	4	0	8	193	201	3706161	184044	6825	251729	9390

For peer review only

For peer review only - http://bmjopen.bmj.com/site/about/guidelines.xhtml

Appendix A

Table (44): Average medical expenditure (Rs.) for treatment (other than as inpatient) under different heads per ailing person medically treated during last 15 days by mpce class

all-India Rural Female s

mpce class	average medical expenditure (Rs.) for treatment under different heads for treatment other than as inpatient incurred on: services: doctor's/ surgeons fee: hospital staff	services: doctor's/ surgeons fee: other specialist	services: diagnostic test	services: attendant charges	services: physio therapy	services: other services	goods: medicines: from hospital	goods: medicines: from outside	goods: personal medical appliances	goods: food etc.	goods: blood oxygen, etc.	total (govt.)	total (pvt.)	total	estd. expn. (000)	persons treated medically on payment during last 15 days (00): estd. (00)	persons treated medically on payment during last 15 days (00): sample	persons treated medically during last 15 days: estd. (00)	persons treated medically during last 15 days: sample
(1)	(2)	(3)	(4)	(5)	(6)	(7)	(8)	(9)	(10)	(11)	(12)	(13)	(14)	(15)	(16)	(17)	(18)	(19)	(20)
0 – 225	8	6	2	0	0	0	3	80	0	3	0	5	96	102	72785	7162	218	10659	328
225 – 255	11	5	15	4	1	0	6	134	0	13	0	6	181	187	85556	4556	150	6830	229
255 – 300	10	4	4	0	0	0	2	116	0	5	0	16	125	141	184704	13001	409	18164	574
300 – 340	12	7	9	0	0	0	6	120	0	4	0	8	151	158	182695	11528	406	16093	579
340 – 380	9	8	6	0	0	1	8	125	0	5	0	6	155	161	258994	16058	574	22381	784
380 – 420	16	6	21	0	0	3	12	120	0	3	0	15	165	180	323074	17943	644	23951	835
420 – 470	9	9	3	0	0	0	12	125	0	2	0	6	154	160	353254	21973	731	28355	957
470 – 525	15	9	7	0	0	0	16	160	0	5	1	11	201	212	503000	23628	796	30357	1056
525 – 615	14	12	8	0	0	0	17	148	0	2	0	5	198	203	433455	21337	811	27994	1084
615 – 775	14	8	13	1	0	0	20	157	1	3	0	5	212	217	578478	26522	1080	34734	1424
775 – 950	20	8	12	0	0	0	9	193	0	3	0	10	236	245	419541	17043	593	21768	793
950 +	18	11	13	3	1	1	15	221	0	4	2	12	275	287	590491	20502	767	27570	1052
all classes	14	8	10	1	0	1	12	149	0	4	0	9	189	197	3986027	201253	7179	268855	9695
social group																			
ST	10	6	9	0	0	1	5	109	0	4	0	5	139	144	188618	13083	555	17867	772
SC	11	8	6	0	0	0	13	126	0	4	0	8	159	167	705010	41909	1379	55753	1863
OBC	16	7	10	1	0	0	13	149	0	4	0	12	189	201	1667690	82821	2949	113110	3999
others	13	11	11	1	0	1	12	171	0	3	1	6	217	223	1419382	63350	2294	82035	3059
all	14	8	10	1	0	1	12	149	0	4	0	9	189	197	3986027	201253	7179	268855	9695

For peer review only - http://bmjopen.bmj.com/site/about/guidelines.xhtml

Appendix A

Table (44): Average medical expenditure (Rs.) for treatment (other than as inpatient) under different heads per ailing person medically treated during last 15 days by mpce class

all-India Rural Persons

mpce class	average medical expenditure (Rs.) for treatment under different heads for treatment other than as inpatient incurred on														estd. expn. (000)	persons treated medically on payment during last 15 days (00)		persons treated medically during last 15 days	
	services						goods					total (govt.)	total (pvt.)	total		estd. (00)	sam-ple	estd. (00)	sam-ple
	doctor's/ surgeons fee		diag-nostic test	atten-dant char-ges	physio the-rapy	other services	medicines		perso-nal medi-cal applia-nces	food etc.	blood oxy-gen, etc.								
	hospi-tal staff	other specia-list					from hos-pital	from out-side											
(1)	(2)	(3)	(4)	(5)	(6)	(7)	(8)	(9)	(10)	(11)	(12)	(13)	(14)	(15)	(16)	(17)	(18)	(19)	(20)
0 – 225	9	5	3	0	0	0	5	84	0	3	0	5	104	109	155647	14238	451	20738	662
225 – 255	8	7	10	2	0	0	4	119	0	12	0	4	155	160	159989	9890	310	14294	459
255 – 300	9	5	7	0	0	0	4	116	0	5	0	11	135	146	366980	25113	811	35625	1153
300 – 340	10	6	6	0	0	0	6	110	0	3	0	7	135	142	320980	22632	795	32117	1136
340 – 380	9	10	6	0	0	1	6	134	0	4	1	12	159	171	542091	31682	1091	43933	1509
380 – 420	14	5	14	0	0	3	10	135	0	3	0	13	170	183	651082	35444	1271	47455	1683
420 – 470	13	10	5	0	0	1	14	133	1	3	0	7	172	179	724629	40235	1430	54011	1906
470 – 525	13	10	8	0	0	1	19	155	2	6	0	13	201	214	948216	44322	1564	57867	2100
525 – 615	15	11	11	0	0	0	20	144	0	3	0	5	201	205	872856	42358	1629	55924	2193
615 – 775	15	10	16	0	0	0	18	158	1	3	0	4	215	219	1149537	52189	2108	68901	2789
775 – 950	20	9	11	0	0	1	12	176	0	3	0	7	225	232	705053	30305	1097	40249	1501
950 +	18	10	15	2	1	1	20	224	2	3	1	9	287	296	1095128	36887	1447	49471	1994
all classes	14	9	10	0	0	1	14	148	1	4	0	8	191	199	7692188	385296	14004	520584	19085
social group																			
ST	8	6	7	0	0	1	6	111	0	5	0	5	140	145	341419	23540	1099	32111	1535
SC	11	7	7	0	0	1	14	124	0	4	0	7	160	167	1362305	81201	2698	108358	3645
OBC	16	7	10	0	0	1	14	148	1	4	0	10	191	201	3181001	157776	5699	217140	7847
others	13	13	12	1	0	1	14	171	1	3	1	8	219	228	2802129	122689	4505	162884	6055
all	14	9	10	0	0	1	14	148	1	4	0	8	191	199	7692188	385296	14004	520584	19085

For peer review only

For peer review only - http://bmjopen.bmj.com/site/about/guidelines.xhtml

Appendix A

Table (44): Average medical expenditure (Rs.) for treatment (other than as inpatient) under different heads per ailing person medically treated during last 15 days by mpce class

all-India Urban Males

mpce class	average medical expenditure (Rs.) for treatment under different heads for treatment other than as inpatient incurred on: services – doctor's/ surgeons fee – hospital staff	services – doctor's/ surgeons fee – other specialist	services – diagnostic test	services – attendant charges	services – physio therapy	services – other services	goods – medicines – from hospital	goods – medicines – from outside	goods – personal medical appliances	goods – food etc.	goods – blood oxygen, etc.	total (govt.)	total (pvt.)	total	estd. expn. (000)	persons treated medically on payment during last 15 days (00) – estd. (00)	persons treated medically on payment during last 15 days (00) – sample	persons treated medically during last 15 days – estd. (00)	persons treated medically during last 15 days – sample
(1)	(2)	(3)	(4)	(5)	(6)	(7)	(8)	(9)	(10)	(11)	(12)	(13)	(14)	(15)	(16)	(17)	(18)	(19)	(20)
0 –300	12	10	3	0	0	0	7	134	0	2	0	6	161	167	33433	1992	95	2508	123
300 – 350	6	20	0	0	0	0	2	121	0	0	0	1	149	150	25197	1680	78	2367	108
350 – 425	9	9	14	0	0	0	4	111	0	2	1	4	146	150	62735	4178	203	5573	283
425 – 500	10	17	4	0	0	0	14	136	0	2	0	4	179	183	133133	7257	416	9920	587
500 – 575	12	17	7	0	0	1	11	149	0	1	0	4	194	197	76622	3886	229	5258	311
575 – 665	11	8	3	0	0	1	6	156	0	4	0	6	181	187	100269	5344	321	7133	436
665 – 775	12	10	7	0	0	0	11	125	0	1	0	8	159	166	158034	9489	475	12276	667
775 – 915	18	14	10	0	0	1	13	215	0	1	0	5	263	268	202409	7460	477	10553	653
915 – 1120	17	13	28	0	0	0	17	166	0	2	0	3	239	242	255764	10498	580	13803	762
1120 – 1500	23	24	27	0	2	0	6	262	0	4	0	2	347	349	458676	13129	653	17495	883
1500 – 1925	14	30	16	0	3	0	14	198	0	1	0	5	270	275	91687	3338	207	4646	299
1925 +	40	33	165	14	0	2	22	269	0	1	0	3	543	545	505502	9249	417	12166	575
all classes	18	18	32	2	1	1	12	187	0	2	0	4	267	271	2103462	77500	4151	103700	5687
social group																			
ST	25	8	12	0	1	0	8	150	0	2	0	13	194	207	22048	1063	127	1677	192
SC	14	21	37	0	0	0	18	135	0	3	0	3	223	226	223268	9805	496	14048	738
OBC	18	13	12	1	1	0	17	147	0	2	0	5	203	208	553033	26464	1495	34504	1993
others	19	20	45	3	1	1	7	226	0	2	0	4	321	325	1305113	40168	2033	53465	2763
all	18	18	32	2	1	1	12	187	0	2	0	4	267	271	2103462	77500	4151	103700	5687

For peer review only

For peer review only - http://bmjopen.bmj.com/site/about/guidelines.xhtml

Appendix A

Table (44): Average medical expenditure (Rs.) for treatment (other than as inpatient) under different heads per ailing person medically treated during last 15 days by mpce class

all-India | Urban | Females

mpce class	average medical expenditure (Rs.) for treatment under different heads for treatment other than as inpatient incurred on														estd. expn. (000)	persons treated medically on payment during last 15 days (00)		persons treated medically during last 15 days	
	services						goods					total (govt.)	total (pvt.)	total		estd. (00)	sam-ple	estd. (00)	sam-ple
	doctor's/ surgeons fee		diag-nostic test	atten-dant char-ges	physio the-rapy	other services	medicines		perso-nal medi-cal applia-nces	food etc.	blood oxy-gen, etc.								
	hospi-tal staff	other specia-list					from hos-pital	from out-side											
(1)	(2)	(3)	(4)	(5)	(6)	(7)	(8)	(9)	(10)	(11)	(12)	(13)	(14)	(15)	(16)	(17)	(18)	(19)	(20)
0 –300	32	24	4	0	0	0	18	179	0	29	0	2	284	286	41540	1452	78	1957	111
300 – 350	7	9	4	0	0	0	2	104	0	2	0	4	124	128	23364	1824	81	2554	118
350 – 425	12	7	6	0	0	0	10	110	0	1	0	4	140	144	62005	4297	219	5677	316
425 – 500	12	9	7	0	0	1	23	115	0	1	0	1	166	168	143876	8575	479	11153	629
500 – 575	12	8	13	1	0	0	20	105	0	3	0	3	158	161	73298	4542	259	5619	325
575 – 665	12	16	12	1	0	0	14	136	0	5	0	4	191	194	129799	6596	373	8951	486
665 – 775	14	11	21	0	0	0	7	155	0	3	0	3	208	212	229030	10784	567	13922	737
775 – 915	21	11	13	2	1	0	11	175	0	1	0	3	232	235	199357	8474	554	11641	731
915 – 1120	21	19	15	0	1	1	10	201	1	0	0	3	265	268	294280	10955	655	14504	849
1120 – 1500	19	28	24	1	0	1	8	282	0	3	1	3	365	368	468133	12690	728	16574	982
1500 – 1925	22	35	23	1	2	0	13	199	2	2	0	3	296	299	118514	3952	259	5394	337
1925 +	25	33	25	1	3	0	8	271	0	2	0	3	364	367	392626	10687	476	14347	649
all classes	18	19	16	1	1	1	12	187	0	3	0	3	253	256	2175821	84829	4728	112292	6270
social group																			
ST	11	6	8	0	0	0	7	142	0	0	0	16	159	175	19792	1131	161	1641	226
SC	13	15	16	0	0	0	20	205	0	1	0	2	270	271	311329	11479	565	14951	764
OBC	20	11	15	0	0	0	15	146	0	3	0	2	209	211	577900	27354	1660	36172	2152
others	17	24	18	1	1	1	8	209	0	3	0	3	278	281	1266800	44864	2342	59524	3127
all	18	19	16	1	1	1	12	187	0	3	0	3	253	256	2175821	84829	4728	112292	6270

For peer review only - http://bmjopen.bmj.com/site/about/guidelines.xhtml

Appendix A

Table (44): Average medical expenditure (Rs.) for treatment (other than as inpatient) under different heads per ailing person medically treated during last 15 days by mpce class

all-India Urban Persons

mpce class	average medical expenditure (Rs.) for treatment under different heads for treatment other than as inpatient incurred on														estd. expn. (000)	persons treated medically on payment during last 15 days (00)		persons treated medically during last 15 days	
	services						goods					total (govt.)	total (pvt.)	total		estd. (00)	sam-ple	estd. (00)	sam-ple
	doctor's/ surgeons fee		diag-nostic test	atten-dant char-ges	physio the-rapy	other services	medicines		perso-nal medi-cal applia-nces	food etc.	blood oxy-gen, etc.								
	hospi-tal staff	other specia-list					from hos-pital	from out-side											
(1)	(2)	(3)	(4)	(5)	(6)	(7)	(8)	(9)	(10)	(11)	(12)	(13)	(14)	(15)	(16)	(17)	(18)	(19)	(20)
0 –300	20	16	3	0	0	0	12	153	0	13	0	4	213	217	74973	3444	173	4465	234
300 – 350	6	14	2	0	0	0	2	112	0	1	0	3	136	138	48561	3504	159	4921	226
350 – 425	10	8	10	0	0	0	7	110	0	1	0	4	143	147	124740	8475	422	11251	599
425 – 500	11	13	6	0	0	1	19	125	0	1	0	3	172	175	277009	15832	895	21073	1216
500 – 575	12	12	10	0	0	0	16	125	0	2	0	3	175	178	149920	8428	488	10877	636
575 – 665	12	12	8	0	0	0	10	145	0	4	0	5	186	191	230068	11940	694	16084	922
665 – 775	13	11	14	0	0	0	9	141	0	2	0	5	185	190	387063	20273	1042	26198	1404
775 – 915	19	12	12	1	0	1	12	194	0	1	0	4	246	251	401766	15934	1031	22194	1384
915 – 1120	19	16	21	0	0	1	13	184	0	1	0	3	252	256	550044	21453	1235	28308	1611
1120 – 1500	21	26	25	1	1	1	7	272	0	4	1	3	356	358	926809	25819	1381	34069	1865
1500 – 1925	18	33	20	0	2	0	13	199	1	1	0	4	284	288	210202	7290	466	10040	636
1925 +	32	33	90	7	2	1	15	270	0	1	0	3	447	450	898128	19936	893	26513	1224
all classes	18	18	24	1	1	1	12	187	0	2	0	4	259	263	4279283	162328	8879	215992	11957
social group																			
ST	18	7	10	0	0	0	7	146	0	1	0	15	176	191	41840	2194	288	3318	418
SC	13	17	26	0	0	0	19	173	0	2	0	2	248	250	534597	21284	1061	28999	1502
OBC	19	12	13	1	0	0	16	146	0	2	0	3	206	210	1130933	53818	3155	70677	4145
others	18	22	31	2	1	1	7	217	0	2	0	4	298	302	2571914	85033	4375	112988	5890
all	18	18	24	1	1	1	12	187	0	2	0	4	259	263	4279283	162328	8879	215992	11957

For peer review only - http://bmjopen.bmj.com/site/about/guidelines.xhtml

Appendix A

Table (44): Average medical expenditure (Rs.) for treatment (other than as inpatient) under different heads per ailing person medically treated during last 15 days by mpce class

all-India Rural + Urban Males

mpce class	average medical expenditure (Rs.) for treatment under different heads for treatment other than as inpatient incurred on: services: doctor's/ surgeons fee: hospital staff	services: doctor's/ surgeons fee: other specialist	services: diagnostic test	services: attendant charges	services: physio therapy	services: other services	goods: medicines: from hospital	goods: medicines: from outside	goods: personal medical appliances	goods: food etc.	goods: blood oxygen, etc.	total (govt.)	total (pvt.)	total	estd. expn. (000)	persons treated medically on payment during last 15 days (00): estd. (00)	persons treated medically on payment during last 15 days (00): sample	persons treated medically during last 15 days: estd. (00)	persons treated medically during last 15 days: sample
(1)	(2)	(3)	(4)	(5)	(6)	(7)	(8)	(9)	(10)	(11)	(12)	(13)	(14)	(15)	(16)	(17)	(18)	(19)	(20)
0 – 235	10	5	5	0	0	0	7	98	0	3	0	5	122	128	116296	9068	328	12587	457
235 – 265	5	11	5	0	0	0	2	110	0	9	0	3	137	139	99631	7015	238	9830	338
265 – 320	8	6	10	0	0	0	5	115	0	5	0	5	145	150	245012	16290	605	23035	862
320 – 365	9	9	4	0	0	0	10	114	0	2	0	5	143	148	271417	18361	805	25944	1144
365 – 410	10	14	5	1	0	0	5	144	0	3	2	15	169	184	359719	19509	746	26810	1036
410 – 460	11	5	6	0	0	2	7	153	0	4	0	10	177	187	428277	22846	948	30637	1284
460 – 520	15	11	7	0	0	1	16	137	1	2	0	8	182	190	529408	27751	1174	37932	1616
520 – 605	13	12	8	0	0	1	21	167	3	4	0	12	217	229	647625	28155	1245	38064	1697
605 – 730	17	12	19	0	0	0	21	148	0	4	0	4	215	220	695165	31519	1398	41734	1871
730 – 980	19	15	21	0	1	0	13	194	0	2	0	3	261	265	1029735	38796	1681	51662	2248
980 – 1285	18	15	11	0	1	1	16	163	0	2	0	4	223	227	377199	16600	711	23127	1007
1285 +	25	18	71	5	0	2	26	243	2	2	0	4	389	394	1010140	25634	1097	34067	1517
all classes	15	12	17	1	0	1	14	159	1	3	0	7	215	222	5809623	261544	10976	355429	15077
social group																			
ST	8	7	5	0	0	0	8	117	0	6	0	5	147	152	174849	11519	671	15921	955
SC	11	10	14	0	0	1	16	124	0	3	0	5	174	179	880564	49097	1815	66653	2520
OBC	17	8	10	0	0	1	16	147	1	3	0	7	196	203	2066344	101419	4245	138534	5841
others	15	17	26	1	0	1	12	193	1	3	0	8	261	270	2687860	99508	4244	134313	5759
all	15	12	17	1	0	1	14	159	1	3	0	7	215	222	5809623	261544	10976	355429	15077

For peer review only - http://bmjopen.bmj.com/site/about/guidelines.xhtml

Appendix A

Table (44): Average medical expenditure (Rs.) for treatment (other than as inpatient) under different heads per ailing person medically treated during last 15 days by mpce class

all-India | Rural + Urban | Females

mpce class	average medical expenditure (Rs.) for treatment under different heads for treatment other than as inpatient incurred on														estd. expn. (000)	persons treated medically on payment during last 15 days (00)		persons treated medically during last 15 days	
	services						goods					total (govt.)	total (pvt.)	total		estd. (00)	sam-ple	estd. (00)	sam-ple
	doctor's/ surgeons fee		diag-nostic test	atten-dant char-ges	physio the-rapy	other services	medicines		perso-nal medi-cal applia-nces	food etc.	blood oxy-gen, etc.								
	hospi-tal staff	other specia-list					from hos-pital	from out-side											
(1)	(2)	(3)	(4)	(5)	(6)	(7)	(8)	(9)	(10)	(11)	(12)	(13)	(14)	(15)	(16)	(17)	(18)	(19)	(20)
0 – 235	12	9	2	0	0	0	6	96	0	7	0	5	128	133	114325	8614	296	12615	439
235 – 265	10	6	12	3	0	0	5	126	0	10	0	5	165	170	108920	6380	231	9384	347
265 – 320	10	5	5	0	0	0	4	114	0	4	0	13	129	142	246708	17298	628	23841	890
320 – 365	12	8	8	0	0	1	13	118	0	3	0	5	158	162	326571	20103	885	27246	1208
365 – 410	9	8	8	0	0	1	10	121	0	4	0	6	156	161	332292	20600	833	27999	1109
410 – 460	15	8	19	0	0	2	12	124	0	4	0	12	172	184	452874	24539	1017	32902	1321
460 – 520	11	10	9	0	0	0	10	135	0	2	0	5	172	177	582284	32757	1298	42277	1694
520 – 605	16	9	9	1	0	0	14	164	0	4	1	9	209	218	702357	32101	1350	41997	1787
605 – 730	16	15	11	0	0	1	15	166	0	1	0	4	221	225	727734	32292	1466	42498	1933
730 – 980	16	15	17	1	0	1	16	197	1	3	1	4	262	266	1046611	39212	1808	51308	2406
980 – 1285	20	13	14	0	0	0	10	194	0	3	0	8	247	255	538056	20996	852	27162	1130
1285 +	20	18	17	2	2	1	12	238	0	3	1	9	305	314	983117	31189	1243	41917	1701
all classes	15	11	12	1	0	1	12	160	0	3	0	7	208	215	6161848	286081	11907	381147	15965
social group																			
ST	10	6	9	0	0	1	5	111	0	4	0	6	141	147	208410	14214	716	19508	998
SC	11	9	9	0	0	0	14	143	0	3	0	7	183	189	1016339	53387	1944	70704	2627
OBC	17	8	11	1	0	0	14	148	0	4	0	9	194	203	2245589	110175	4609	149282	6151
others	15	17	14	1	1	1	10	187	0	3	0	5	242	247	2686182	108214	4636	141559	6186
all	15	11	12	1	0	1	12	160	0	3	0	7	208	215	6161848	286081	11907	381147	15965

For peer review only

For peer review only - http://bmjopen.bmj.com/site/about/guidelines.xhtml

Appendix A

Table (44): Average medical expenditure (Rs.) for treatment (other than as inpatient) under different heads per ailing person medically treated during last 15 days by mpce class

all-India Rural + Urban Persons

mpce class	average medical expenditure (Rs.) for treatment under different heads for treatment other than as inpatient incurred on														estd. expn. (000)	persons treated medically on payment during last 15 days (00)		persons treated medically during last 15 days	
	services						goods					total (govt.)	total (pvt.)	total		estd. (00)	sample	estd. (00)	sample
	doctor's/ surgeons fee		diagnostic test	attendant charges	physio therapy	other services	medicines		personal medical appliances	food etc.	blood oxygen, etc.								
	hospital staff	other specialist					from hospital	from outside											
(1)	(2)	(3)	(4)	(5)	(6)	(7)	(8)	(9)	(10)	(11)	(12)	(13)	(14)	(15)	(16)	(17)	(18)	(19)	(20)
0 – 235	11	7	3	0	0	0	7	97	0	5	0	5	125	130	230620	17682	624	25203	896
235 – 265	7	8	8	1	0	0	3	117	0	9	0	4	150	154	208551	13394	469	19214	685
265 – 320	10	6	7	0	0	0	5	115	0	4	0	9	137	146	491720	33588	1233	46876	1752
320 – 365	11	9	6	0	0	0	11	116	0	2	0	5	150	155	597989	38464	1690	53190	2352
365 – 410	10	11	7	0	0	1	8	132	0	4	1	10	162	173	692011	40109	1579	54810	2145
410 – 460	13	7	13	0	0	2	10	138	0	4	0	11	174	185	881151	47385	1965	63539	2605
460 – 520	13	10	8	0	0	1	13	136	1	2	0	6	176	183	1111692	60508	2472	80209	3310
520 – 605	15	10	9	0	0	1	17	165	1	4	0	11	213	223	1349982	60256	2595	80061	3484
605 – 730	16	13	15	0	0	1	18	157	0	3	0	4	218	222	1422899	63811	2864	84232	3804
730 – 980	17	15	19	1	0	0	14	195	1	3	0	4	262	265	2076346	78008	3489	102970	4654
980 – 1285	19	14	13	0	1	1	13	181	0	2	0	7	236	243	915254	37596	1563	50289	2137
1285 +	23	18	41	3	1	1	18	240	1	3	1	7	343	350	1993256	56823	2340	75984	3218
all classes	15	12	14	1	0	1	13	159	0	3	0	7	211	218	11971471	547625	22883	736576	31042
social group																			
ST	9	6	7	0	0	1	6	114	0	5	0	5	143	149	383259	25734	1387	35429	1953
SC	11	9	11	0	0	0	15	134	0	3	0	6	178	184	1896902	102484	3759	137358	5147
OBC	17	8	11	0	0	1	15	147	1	4	0	8	195	203	4311934	211594	8854	287816	11992
others	15	17	20	1	1	1	11	190	1	3	0	7	252	258	5374042	207722	8880	275872	11945
all	15	12	14	1	0	1	13	159	0	3	0	7	211	218	11971471	547625	22883	736576	31042

For peer review only - http://bmjopen.bmj.com/site/about/guidelines.xhtml

Appendix A

Table (45): Average total expenditure incurred during the last 15 days on account of treatment per ailing person (but not as inpatient of a hospital) for each sex and mpce class

all-India Rural Males

mpce class	average total expenditure (Rs) per treated person during the last 15 days on account of ailment (but not as inpatient of a hospital)				average loss of hhs. income due to ailment (Rs.) per ailing person	persons reporting treatment of ailment during last 15 days		persons reporting ailment during last 15 days	
	medical treatment (Govt.)	medical treatment (Pvt.)	other expenses incurred due to ailment	total expenditure		estd. no. (00)	sample	estd. no. (00)	sample
(1)	(2)	(3)	(4)	(5)	(6)	(7)	(8)	(9)	(10)
0 – 225	9	194	26	229	1245	9858	316	10079	334
225 – 255	9	193	15	218	183	7176	219	7463	230
255 – 300	11	201	22	234	128	16524	550	17461	579
300 – 340	31	168	34	234	102	15538	535	16024	557
340 – 380	17	190	24	231	140	20324	694	21552	725
380 – 420	15	204	24	243	132	22850	819	23503	848
420 – 470	11	237	27	275	241	24790	910	25656	949
470 – 525	16	391	28	435	280	26664	1001	27510	1044
525 – 615	9	239	27	275	175	27029	1062	27930	1109
615 – 775	4	274	29	308	135	33159	1316	34167	1365
775 – 950	13	291	39	343	148	17310	677	18481	708
950 +	8	434	39	482	128	20917	896	21901	942
all	12	263	28	304	211	242140	8995	251729	9390

Table (45): Average total expenditure incurred during the last 15 days on account of treatment per ailing person (but not as inpatient of a hospital) for each sex and mpce class

all-India Rural Females

mpce class	average total expenditure (Rs) per treated person during the last 15 days on account of ailment (but not as inpatient of a hospital)				average loss of hhs. income due to ailment (Rs.) per ailing person	persons reporting treatment of ailment during last 15 days		persons reporting ailment during last 15 days	
	medical treatment (Govt.)	medical treatment (Pvt.)	other expenses incurred due to ailment	total expenditure		estd. no. (00)	sample	estd. no. (00)	sample
(1)	(2)	(3)	(4)	(5)	(6)	(7)	(8)	(9)	(10)
0 – 225	9	132	13	154	46	10174	306	10659	328
225 – 255	7	234	22	262	53	6173	210	6830	229
255 – 300	19	149	21	189	63	17747	550	18164	574
300 – 340	7	213	21	240	58	15483	556	16093	579
340 – 380	7	198	23	228	67	21410	754	22381	784
380 – 420	23	201	32	256	60	23311	806	23951	835
420 – 470	8	188	18	214	78	27523	916	28355	957
470 – 525	11	247	30	288	62	29044	1011	30357	1056
525 – 615	5	244	29	277	79	26837	1034	27994	1084
615 – 775	4	251	30	286	61	33636	1373	34734	1424
775 – 950	9	270	27	306	56	20753	756	21768	793
950 +	15	333	35	383	65	26851	1002	27570	1052
all	10	230	27	267	64	258940	9274	268855	9695

For peer review only - http://bmjopen.bmj.com/site/about/guidelines.xhtml

Appendix A

Table (45): Average total expenditure incurred during the last 15 days on account of treatment per ailing person (but not as inpatient of a hospital) for each sex and mpce class

all-India Rural Persons

mpce class	average total expenditure (Rs) per treated person during the last 15 days on account of ailment (but not as inpatient of a hospital)				average loss of hhs. income due to ailment (Rs.) per ailing person	persons reporting treatment of ailment during last 15 days		persons reporting ailment during last 15 days	
	medical treatment (Govt.)	medical treatment (Pvt.)	other expenses incurred due to ailment	total expenditure		estd. no. (00)	sample	estd. no. (00)	sample
(1)	(2)	(3)	(4)	(5)	(6)	(7)	(8)	(9)	(10)
0 – 225	9	163	19	191	636	20032	622	20738	662
225 – 255	8	212	18	238	123	13349	429	14294	459
255 – 300	15	174	21	210	94	34271	1100	35625	1153
300 – 340	19	190	27	237	80	31021	1091	32117	1136
340 – 380	12	194	24	229	102	41734	1448	43933	1509
380 – 420	19	202	28	250	96	46160	1625	47455	1683
420 – 470	9	211	22	243	155	52314	1826	54011	1906
470 – 525	13	316	29	358	166	55708	2012	57867	2100
525 – 615	7	241	28	276	127	53866	2096	55924	2193
615 – 775	4	263	30	297	98	66795	2689	68901	2789
775 – 950	11	280	32	323	98	38063	1433	40249	1501
950 +	12	377	37	426	93	47768	1898	49471	1994
all	11	246	27	285	135	501080	18269	520584	19085

Table (45): Average total expenditure incurred during the last 15 days on account of treatment per ailing person (but not as inpatient of a hospital) for each sex and mpce class

all-India Urban Males

mpce class	average total expenditure (Rs) per treated person during the last 15 days on account of ailment (but not as inpatient of a hospital)				average loss of hhs. income due to ailment (Rs.) per ailing person	persons reporting treatment of ailment during last 15 days		persons reporting ailment during last 15 days	
	medical treatment (Govt.)	medical treatment (Pvt.)	other expenses incurred due to ailment	total expenditure		estd. no. (00)	sample	estd. no. (00)	sample
(1)	(2)	(3)	(4)	(5)	(6)	(7)	(8)	(9)	(10)
0 –300	5	174	35	214	416	2466	120	2508	123
300 – 350	1	324	8	333	171	2359	107	2367	108
350 – 425	4	206	7	217	340	5363	274	5573	283
425 – 500	5	213	15	234	151	9453	552	9920	587
500 – 575	16	238	17	271	161	5098	296	5258	311
575 – 665	9	269	14	292	98	6877	410	7133	436
665 – 775	8	224	19	252	135	11763	613	12276	667
775 – 915	7	311	16	334	92	9686	599	10553	653
915 – 1120	3	252	16	270	93	12918	714	13803	762
1120 – 1500	4	402	23	430	202	16286	812	17495	883
1500 – 1925	5	317	15	337	125	4217	265	4646	299
1925 +	6	608	47	662	200	10808	508	12166	575
all	6	316	21	343	163	97294	5270	103700	5687

For peer review only - http://bmjopen.bmj.com/site/about/guidelines.xhtml

Table (45): Average total expenditure incurred during the last 15 days on account of treatment per ailing person (but not as inpatient of a hospital) for each sex and mpce class

all-India Urban Females

mpce class	average total expenditure (Rs) per treated person during the last 15 days on account of ailment (but not as inpatient of a hospital)				average loss of hhs. income due to ailment (Rs.) per ailing person	persons reporting treatment of ailment during last 15 days		persons reporting ailment during last 15 days	
	medical treatment (Govt.)	medical treatment (Pvt.)	other expenses incurred due to ailment	total expenditure		estd. no. (00)	sample	estd. no. (00)	sample
(1)	(2)	(3)	(4)	(5)	(6)	(7)	(8)	(9)	(10)
0 –300	2	242	11	254	51	1867	102	1957	111
300 – 350	4	172	12	188	27	2500	110	2554	118
350 – 425	6	165	10	180	40	5338	292	5677	316
425 – 500	5	260	15	281	38	10737	599	11153	629
500 – 575	3	190	11	204	35	5528	314	5619	325
575 – 665	7	223	26	255	51	8160	454	8951	486
665 – 775	7	226	20	253	21	13205	702	13922	737
775 – 915	3	252	21	276	51	10580	671	11641	731
915 – 1120	3	286	19	308	42	13712	796	14504	849
1120 – 1500	4	375	28	406	18	15580	898	16574	982
1500 – 1925	3	384	15	402	20	4552	305	5394	337
1925 +	27	400	24	451	22	12696	580	14347	649
all	7	284	20	311	33	104457	5823	112292	6270

Table (45): Average total expenditure incurred during the last 15 days on account of treatment per ailing person (but not as inpatient of a hospital) for each sex and mpce class

all-India Urban Persons

mpce class	average total expenditure (Rs) per treated person during the last 15 days on account of ailment (but not as inpatient of a hospital)				average loss of hhs. income due to ailment (Rs.) per ailing person	persons reporting treatment of ailment during last 15 days		persons reporting ailment during last 15 days	
	medical treatment (Govt.)	medical treatment (Pvt.)	other expenses incurred due to ailment	total expenditure		estd. no. (00)	sample	estd. no. (00)	sample
(1)	(2)	(3)	(4)	(5)	(6)	(7)	(8)	(9)	(10)
0 –300	4	203	25	231	259	4333	222	4465	234
300 – 350	2	246	10	258	97	4860	217	4921	226
350 – 425	5	185	9	199	190	10701	566	11251	599
425 – 500	5	238	15	259	91	20191	1151	21073	1216
500 – 575	9	213	14	236	96	10626	610	10877	636
575 – 665	8	244	20	272	72	15037	864	16084	922
665 – 775	8	225	20	253	75	24968	1315	26198	1404
775 – 915	5	280	18	304	70	20267	1270	22194	1384
915 – 1120	3	270	17	290	67	26630	1510	28308	1611
1120 – 1500	4	389	26	418	112	31866	1710	34069	1865
1500 – 1925	4	352	15	371	70	8769	570	10040	636
1925 +	17	496	35	548	104	23504	1088	26513	1224
all	7	299	20	326	96	201751	11093	215992	11957

For peer review only - http://bmjopen.bmj.com/site/about/guidelines.xhtml

Table (45): Average total expenditure incurred during the last 15 days on account of treatment per ailing person (but not as inpatient of a hospital) for each sex and mpce class

all-India Rural + Urban Males

mpce class	average total expenditure (Rs) per treated person during the last 15 days on account of ailment (but not as inpatient of a hospital)				average loss of hhs. income due to ailment (Rs.) per ailing person	persons reporting treatment of ailment during last 15 days		persons reporting ailment during last 15 days	
	medical treatment (Govt.)	medical treatment (Pvt.)	other expenses incurred due to ailment	total expenditure		estd. no. (00)	sample	estd. no. (00)	sample
(1)	(2)	(3)	(4)	(5)	(6)	(7)	(8)	(9)	(10)
0 – 235	9	190	28	226	1079	12324	436	12587	457
235 – 265	7	226	13	246	180	9536	326	9830	338
265 – 320	9	202	18	230	180	21888	824	23035	862
320 – 365	21	185	27	234	121	24992	1087	25944	1144
365 – 410	17	199	23	239	144	25422	990	26810	1036
410 – 460	14	219	22	255	124	29726	1229	30637	1284
460 – 520	10	233	24	268	207	36553	1523	37932	1616
520 – 605	13	370	25	408	230	36351	1600	38064	1697
605 – 730	7	243	23	274	149	39947	1776	41734	1871
730 – 980	4	316	27	348	157	49445	2128	51662	2248
980 – 1285	12	296	34	342	144	21527	942	23127	1007
1285 +	8	493	42	543	153	31725	1404	34067	1517
all	11	278	26	315	197	339434	14265	355429	15077

Table (45): Average total expenditure incurred during the last 15 days on account of treatment per ailing person (but not as inpatient of a hospital) for each sex and mpce class

all-India Rural + Urban Females

mpce class	average total expenditure (Rs) per treated person during the last 15 days on account of ailment (but not as inpatient of a hospital)				average loss of hhs. income due to ailment (Rs.) per ailing person	persons reporting treatment of ailment during last 15 days		persons reporting ailment during last 15 days	
	medical treatment (Govt.)	medical treatment (Pvt.)	other expenses incurred due to ailment	total expenditure		estd. no. (00)	sample	estd. no. (00)	sample
(1)	(2)	(3)	(4)	(5)	(6)	(7)	(8)	(9)	(10)
0 – 235	8	149	13	170	47	12041	408	12615	439
235 – 265	6	216	19	241	45	8673	320	9384	347
265 – 320	16	152	18	187	58	23085	842	23841	890
320 – 365	6	232	19	257	50	26220	1155	27246	1208
365 – 410	6	196	20	223	60	26939	1068	27999	1109
410 – 460	19	207	30	256	58	31471	1260	32902	1321
460 – 520	8	200	19	226	59	40728	1618	42277	1694
520 – 605	9	249	27	285	59	39624	1682	41997	1787
605 – 730	4	258	26	288	66	40549	1830	42498	1933
730 – 980	4	290	29	324	48	49216	2271	51308	2406
980 – 1285	8	291	25	323	49	25305	1061	27162	1130
1285 +	19	354	32	405	51	39547	1582	41917	1701
all	9	245	25	279	55	363396	15097	381147	15965

For peer review only

For peer review only - http://bmjopen.bmj.com/site/about/guidelines.xhtml

Appendix A

Table (45): Average total expenditure incurred during the last 15 days on account of treatment per ailing person (but not as inpatient of a hospital) for each sex and mpce class

all-India Rural + Urban Persons

mpce class	average total expenditure (Rs) per treated person during the last 15 days on account of ailment (but not as inpatient of a hospital)				average loss of hhs. income due to ailment (Rs.) per ailing person	persons reporting treatment of ailment during last 15 days		persons reporting ailment during last 15 days	
	medical treatment (Govt.)	medical treatment (Pvt.)	other expenses incurred due to ailment	total expenditure		estd. no. (00)	sample	estd. no. (00)	sample
(1)	(2)	(3)	(4)	(5)	(6)	(7)	(8)	(9)	(10)
0 – 235	8	170	20	198	569	24365	844	25203	896
235 – 265	7	221	16	244	116	18209	646	19214	685
265 – 320	13	177	18	208	117	44972	1666	46876	1752
320 – 365	13	209	23	245	84	51211	2242	53190	2352
365 – 410	11	198	22	231	101	52360	2058	54810	2145
410 – 460	16	213	26	255	90	61197	2489	63539	2605
460 – 520	9	216	21	246	129	77281	3141	80209	3310
520 – 605	11	307	26	344	141	75974	3282	80061	3484
605 – 730	6	251	25	281	107	80496	3606	84232	3804
730 – 980	4	303	28	336	102	98661	4399	102970	4654
980 – 1285	9	293	29	332	93	46831	2003	50289	2137
1285 +	14	416	36	466	96	71272	2986	75984	3218
all	10	261	25	297	124	702831	29362	736576	31042

For peer review only - http://bmjopen.bmj.com/site/about/guidelines.xhtml

Appendix A

Table (46): Per 1000 distribution of ailing persons among those who were medically treated but not as inpatient of hospital during last 15 days by type of payment made for availing some services for each category of specific medical service for each State/UT

medical service: surgery Rural

state/ut	type of payment for services				total	number of persons with ailments treated	
	medical service not received / required	services received				estd. no. (00)	sample
		free	partly free	on payment			
(1)	(2)	(3)	(4)	(5)	(6)	(7)	(8)
Andhra Pradesh	991	4	0	5	1000	34263	1255
Arunachal Pradesh	923	24	10	43	1000	204	95
Assam	990	0	0	10	1000	12124	611
Bihar	972	12	0	16	1000	23319	802
Chhattisgarh	979	1	3	17	1000	10732	377
Delhi	1000	0	0	0	1000	69	3
Goa	1000	0	0	0	1000	1504	35
Gujarat	980	11	1	8	1000	18147	540
Haryana	994	1	0	5	1000	12716	469
Himachal Pradesh	982	0	0	18	1000	4317	600
Jammu & Kashmir	989	1	5	6	1000	3411	309
Jharkhand	972	12	0	16	1000	5430	207
Karnataka	992	2	0	6	1000	16399	537
Kerala	982	2	3	12	1000	49700	1797
Madhya Pradesh	989	2	0	8	1000	23706	726
Maharashtra	975	6	0	19	1000	44473	1248
Manipur	742	107	0	151	1000	304	106
Meghalaya	986	14	0	0	1000	295	55
Mizoram	1000	0	0	0	1000	11	6
Nagaland	1000	0	0	0	1000	222	67
Orissa	990	5	0	5	1000	17523	565
Punjab	985	7	0	9	1000	19786	599
Rajasthan	987	8	0	6	1000	20451	814
Sikkim	988	9	0	3	1000	119	62
Tamil Nadu	998	2	0	0	1000	29491	895
Tripura	993	3	0	4	1000	3064	446
Uttaranchal	992	2	0	6	1000	3051	105
Uttar Pradesh	980	1	0	19	1000	95349	3297
West Bengal	988	1	0	12	1000	49120	1371
A & N Islands	1000	0	0	0	1000	99	30
Chandigarh	865	0	0	135	1000	46	37
Dadra & N. Haveli	1000	0	0	0	1000	32	12
Daman & Diu	1000	0	0	0	1000	18	13
Lakshadweep	958	42	0	0	1000	20	44
Pondicherry	1000	0	0	0	1000	244	31
ONES	971	12	1	16	1000	4218	837
GUTs	984	2	0	14	1000	460	167
all-India	984	4	0	12	1000	499762	18166

For peer review only - http://bmjopen.bmj.com/site/about/guidelines.xhtml

Appendix A

Table (46): Per 1000 distribution of ailing persons among those who were medically treated but not as inpatient of hospital during last 15 days by type of payment made for availing some services for each category of specific medical service for each State/UT

medical service: surgery Urban

state/ut	type of payment for services				total	number of persons with ailments treated	
	medical service not received / required	services received				estd. no. (00)	sample
		free	partly free	on payment			
(1)	(2)	(3)	(4)	(5)	(6)	(7)	(8)
Andhra Pradesh	982	0	0	17	1000	18553	969
Arunachal Pradesh	416	352	0	232	1000	7	11
Assam	974	26	0	0	1000	1603	150
Bihar	991	5	0	4	1000	3975	189
Chhattisgarh	992	8	0	0	1000	1830	150
Delhi	973	2	1	23	1000	1483	105
Goa	984	16	0	0	1000	431	43
Gujarat	982	1	0	17	1000	11437	558
Haryana	967	2	0	31	1000	3992	254
Himachal Pradesh	995	0	0	5	1000	312	81
Jammu & Kashmir	931	6	0	63	1000	994	150
Jharkhand	978	0	0	22	1000	1689	118
Karnataka	991	6	0	3	1000	6748	515
Kerala	995	1	0	4	1000	17272	939
Madhya Pradesh	973	2	0	25	1000	8984	510
Maharashtra	992	2	0	6	1000	39662	1467
Manipur	759	153	0	88	1000	110	62
Meghalaya	944	0	0	56	1000	102	39
Mizoram	971	0	0	29	1000	19	28
Nagaland	1000	0	0	0	1000	125	34
Orissa	989	9	2	0	1000	1702	137
Punjab	967	6	0	26	1000	7775	386
Rajasthan	961	1	0	38	1000	6804	477
Sikkim	1000	0	0	0	1000	5	10
Tamil Nadu	999	1	0	0	1000	17788	1051
Tripura	1000	0	0	0	1000	230	68
Uttaranchal	991	0	0	9	1000	1045	95
Uttar Pradesh	992	1	0	7	1000	31435	1531
West Bengal	989	9	0	2	1000	23247	1085
A & N Islands	1000	0	0	0	1000	59	34
Chandigarh	990	0	0	10	1000	400	128
Dadra & N. Haveli	1000	0	0	0	1000	7	7
Daman & Diu	948	0	0	52	1000	13	14
Lakshadweep	986	14	0	0	1000	39	71
Pondicherry	1000	0	0	0	1000	810	115
ONES	938	32	0	29	1000	599	252
GUTs	996	0	0	3	1000	1328	369
all-India	987	3	0	10	1000	210686	11581

For peer review only - http://bmjopen.bmj.com/site/about/guidelines.xhtml

Appendix A

Table (46): Per 1000 distribution of ailing persons among those who were medically treated but not as inpatient of hospital during last 15 days by type of payment made for availing some services for each category of specific medical service for each State/UT

medical service: surgery — Rural + Urban

state/ut	type of payment for services				total	number of persons with ailments treated	
	medical service not received / required	services received					
		free	partly free	on payment		estd. no. (00)	sample
(1)	(2)	(3)	(4)	(5)	(6)	(7)	(8)
Andhra Pradesh	988	2	0	9	1000	52816	2224
Arunachal Pradesh	905	35	10	50	1000	212	106
Assam	988	3	0	9	1000	13727	761
Bihar	975	11	0	14	1000	27295	991
Chhattisgarh	981	2	3	14	1000	12562	527
Delhi	975	2	1	22	1000	1552	108
Goa	996	4	0	0	1000	1935	78
Gujarat	981	7	1	11	1000	29585	1098
Haryana	988	1	0	12	1000	16708	723
Himachal Pradesh	983	0	0	17	1000	4629	681
Jammu & Kashmir	976	2	4	19	1000	4405	459
Jharkhand	973	9	0	18	1000	7119	325
Karnataka	992	3	0	5	1000	23147	1052
Kerala	986	2	2	10	1000	66971	2736
Madhya Pradesh	985	2	0	13	1000	32690	1236
Maharashtra	983	4	0	13	1000	84135	2715
Manipur	747	119	0	134	1000	413	168
Meghalaya	975	10	0	14	1000	397	94
Mizoram	982	0	0	18	1000	29	34
Nagaland	1000	0	0	0	1000	347	101
Orissa	990	5	0	4	1000	19225	702
Punjab	980	7	0	14	1000	27561	985
Rajasthan	980	6	0	14	1000	27255	1291
Sikkim	988	9	0	3	1000	124	72
Tamil Nadu	998	2	0	0	1000	47280	1946
Tripura	993	3	0	4	1000	3294	514
Uttaranchal	992	1	0	7	1000	4095	200
Uttar Pradesh	982	1	0	16	1000	126785	4828
West Bengal	988	3	0	9	1000	72367	2456
A & N Islands	1000	0	0	0	1000	159	64
Chandigarh	977	0	0	23	1000	447	165
Dadra & N. Haveli	1000	0	0	0	1000	39	19
Daman & Diu	979	0	0	21	1000	31	27
Lakshadweep	976	24	0	0	1000	59	115
Pondicherry	1000	0	0	0	1000	1054	146
ONES	967	15	0	18	1000	4818	1089
GUTs	993	1	0	6	1000	1789	536
all-India	985	3	0	11	1000	710448	29747

For peer review only - http://bmjopen.bmj.com/site/about/guidelines.xhtml

Appendix A

Table (46): Per 1000 distribution of ailing persons among those who were medically treated but not as inpatient of hospital during last 15 days by type of payment made for availing some services for each category of specific medical service for each State/UT

medical service: medicine Rural

state/ut	type of payment for services				total	number of persons with ailments treated	
	medical service not received / required	services received				estd. no. (00)	sample
		free	partly free	on payment			
(1)	(2)	(3)	(4)	(5)	(6)	(7)	(8)
Andhra Pradesh	92	103	35	769	1000	34510	1263
Arunachal Pradesh	419	214	183	184	1000	288	143
Assam	183	27	81	709	1000	13073	658
Bihar	256	3	5	737	1000	24756	836
Chhattisgarh	150	21	12	818	1000	10739	377
Delhi	0	0	0	1000	1000	69	3
Goa	142	52	0	806	1000	1504	35
Gujarat	48	86	64	802	1000	18147	540
Haryana	13	13	34	940	1000	12716	469
Himachal Pradesh	46	36	59	859	1000	4317	600
Jammu & Kashmir	63	36	147	754	1000	3411	309
Jharkhand	90	0	4	906	1000	5726	216
Karnataka	0	146	99	754	1000	16399	537
Kerala	28	111	85	776	1000	49861	1802
Madhya Pradesh	108	32	55	804	1000	23816	728
Maharashtra	117	63	33	786	1000	44478	1249
Manipur	58	0	0	942	1000	338	114
Meghalaya	110	180	113	596	1000	295	55
Mizoram	90	21	0	889	1000	11	6
Nagaland	100	5	12	883	1000	222	67
Orissa	237	78	42	643	1000	17583	573
Punjab	378	12	39	571	1000	19786	599
Rajasthan	42	32	12	915	1000	20451	814
Sikkim	224	143	77	556	1000	124	65
Tamil Nadu	13	257	38	692	1000	29491	895
Tripura	173	15	190	623	1000	3074	448
Uttaranchal	90	11	1	898	1000	3051	105
Uttar Pradesh	95	22	15	868	1000	95953	3318
West Bengal	91	40	57	811	1000	50589	1407
A & N Islands	160	702	129	9	1000	99	30
Chandigarh	0	0	17	983	1000	46	37
Dadra & N. Haveli	0	34	32	934	1000	32	12
Daman & Diu	0	21	0	979	1000	18	13
Lakshadweep	64	779	0	156	1000	20	44
Pondicherry	0	619	0	381	1000	244	31
ONES	174	41	157	629	1000	4352	898
GUTs	37	517	32	414	1000	460	167
all-India	104	64	42	790	1000	505239	18398

For peer review only - http://bmjopen.bmj.com/site/about/guidelines.xhtml

Appendix A

Table (46): Per 1000 distribution of ailing persons among those who were medically treated but not as inpatient of hospital during last 15 days by type of payment made for availing some services for each category of specific medical service for each State/UT

medical service: medicine Urban

state/ut	type of payment for services				total	number of persons with ailments treated	
	medical service not received / required	services received				estd. no. (00)	sample
		free	partly free	on payment			
(1)	(2)	(3)	(4)	(5)	(6)	(7)	(8)
Andhra Pradesh	62	69	47	821	1000	18878	974
Arunachal Pradesh	6	4	5	985	1000	36	34
Assam	62	56	105	777	1000	1679	157
Bihar	611	18	24	347	1000	4012	195
Chhattisgarh	75	192	32	701	1000	1830	150
Delhi	57	100	82	761	1000	1483	105
Goa	204	114	35	647	1000	431	43
Gujarat	49	117	27	808	1000	11437	558
Haryana	27	31	19	923	1000	3988	253
Himachal Pradesh	15	89	90	805	1000	312	81
Jammu & Kashmir	71	28	68	834	1000	998	151
Jharkhand	108	83	32	777	1000	1715	126
Karnataka	11	48	79	862	1000	6748	515
Kerala	50	66	63	821	1000	17330	942
Madhya Pradesh	83	53	31	832	1000	9013	513
Maharashtra	128	45	37	790	1000	39704	1471
Manipur	26	5	4	965	1000	122	65
Meghalaya	21	86	120	773	1000	93	39
Mizoram	148	34	0	818	1000	17	27
Nagaland	10	0	30	960	1000	125	34
Orissa	219	50	25	706	1000	1795	143
Punjab	252	16	8	724	1000	7775	386
Rajasthan	28	75	27	870	1000	6804	477
Sikkim	0	111	167	722	1000	5	10
Tamil Nadu	4	206	53	736	1000	17788	1051
Tripura	281	0	87	632	1000	234	69
Uttaranchal	49	98	2	851	1000	1045	95
Uttar Pradesh	62	43	16	879	1000	31539	1541
West Bengal	37	49	40	874	1000	23482	1102
A & N Islands	0	566	34	399	1000	59	34
Chandigarh	6	38	0	957	1000	400	128
Dadra & N. Haveli	0	181	0	819	1000	7	7
Daman & Diu	14	58	0	928	1000	13	14
Lakshadweep	0	971	0	29	1000	39	71
Pondicherry	0	330	8	662	1000	810	115
ONES	118	16	58	808	1000	633	278
GUTs	2	268	6	724	1000	1328	369
all-India	82	70	38	811	1000	211746	11676

For peer review only - http://bmjopen.bmj.com/site/about/guidelines.xhtml

Table (46): Per 1000 distribution of ailing persons among those who were medically treated but not as inpatient of hospital during last 15 days by type of payment made for availing some services for each category of specific medical service for each State/UT

medical service: medicine Rural + Urban

state/ut	type of payment for services				total	number of persons with ailments treated	
	medical service not received / required	services received				estd. no. (00)	sample
		free	partly free	on payment			
(1)	(2)	(3)	(4)	(5)	(6)	(7)	(8)
Andhra Pradesh	82	91	39	788	1000	53387	2237
Arunachal Pradesh	373	191	163	273	1000	325	177
Assam	169	31	84	716	1000	14753	815
Bihar	305	5	7	683	1000	28768	1031
Chhattisgarh	139	45	14	801	1000	12569	527
Delhi	54	96	78	772	1000	1552	108
Goa	156	66	8	771	1000	1935	78
Gujarat	48	98	49	804	1000	29585	1098
Haryana	16	17	30	936	1000	16704	722
Himachal Pradesh	44	40	61	855	1000	4629	681
Jammu & Kashmir	65	34	129	772	1000	4409	460
Jharkhand	94	19	11	876	1000	7441	342
Karnataka	4	118	94	785	1000	23147	1052
Kerala	33	99	79	788	1000	67191	2744
Madhya Pradesh	101	38	49	812	1000	32828	1241
Maharashtra	122	55	35	788	1000	84182	2720
Manipur	50	1	1	948	1000	460	179
Meghalaya	89	158	115	639	1000	388	94
Mizoram	126	29	0	845	1000	28	33
Nagaland	67	3	19	911	1000	347	101
Orissa	235	76	40	649	1000	19379	716
Punjab	343	13	31	614	1000	27561	985
Rajasthan	38	42	16	904	1000	27255	1291
Sikkim	215	142	81	563	1000	129	75
Tamil Nadu	10	238	44	709	1000	47280	1946
Tripura	181	14	183	623	1000	3308	517
Uttaranchal	80	33	1	886	1000	4095	200
Uttar Pradesh	87	27	15	871	1000	127492	4859
West Bengal	74	43	52	831	1000	74071	2509
A & N Islands	100	651	93	155	1000	159	64
Chandigarh	5	34	2	959	1000	447	165
Dadra & N. Haveli	0	59	27	914	1000	39	19
Daman & Diu	6	36	0	959	1000	31	27
Lakshadweep	22	905	0	73	1000	59	115
Pondicherry	0	397	6	597	1000	1054	146
ONES	167	38	144	651	1000	4985	1176
GUTs	11	332	13	644	1000	1789	536
all-India	97	66	41	796	1000	716986	30074

For peer review only - http://bmjopen.bmj.com/site/about/guidelines.xhtml

Appendix A

Table (46): Per 1000 distribution of ailing persons among those who were medically treated but not as inpatient of hospital during last 15 days by type of payment made for availing some services for each category of specific medical service for each State/UT

medical service: X-ray, ECG, EEG, Scan Rural

state/ut	type of payment for services				total	number of persons with ailments treated	
	medical service not received / required	services received				estd. no. (00)	sample
		free	partly free	on payment			
(1)	(2)	(3)	(4)	(5)	(6)	(7)	(8)
Andhra Pradesh	942	8	1	48	1000	34316	1258
Arunachal Pradesh	880	20	33	67	1000	203	96
Assam	943	2	0	55	1000	12158	611
Bihar	949	13	0	37	1000	23628	805
Chhattisgarh	946	8	0	46	1000	10735	377
Delhi	1000	0	0	0	1000	69	3
Goa	1000	0	0	0	1000	1504	35
Gujarat	935	3	2	60	1000	18147	540
Haryana	952	1	2	45	1000	12716	469
Himachal Pradesh	913	3	3	82	1000	4317	600
Jammu & Kashmir	887	11	32	70	1000	3411	309
Jharkhand	921	5	0	73	1000	5434	205
Karnataka	927	11	0	63	1000	16399	537
Kerala	959	7	5	29	1000	49598	1794
Madhya Pradesh	964	6	0	30	1000	23694	723
Maharashtra	930	7	0	63	1000	44416	1248
Manipur	921	0	15	63	1000	302	104
Meghalaya	986	14	0	0	1000	295	55
Mizoram	857	0	0	143	1000	11	6
Nagaland	968	0	0	32	1000	222	67
Orissa	969	8	1	21	1000	17525	566
Punjab	983	1	2	14	1000	19786	599
Rajasthan	923	3	3	72	1000	20451	814
Sikkim	974	18	0	9	1000	119	60
Tamil Nadu	962	17	0	21	1000	29491	895
Tripura	983	0	3	14	1000	3074	448
Uttaranchal	897	2	0	101	1000	3051	105
Uttar Pradesh	951	2	1	46	1000	95398	3292
West Bengal	946	5	2	47	1000	48942	1371
A & N Islands	957	43	0	0	1000	99	30
Chandigarh	798	0	0	202	1000	46	37
Dadra & N. Haveli	1000	0	0	0	1000	32	12
Daman & Diu	1000	0	0	0	1000	18	13
Lakshadweep	807	178	15	0	1000	20	44
Pondicherry	922	0	0	78	1000	244	31
ONES	972	2	5	20	1000	4225	836
GUTs	921	17	1	62	1000	460	167
all-India	948	6	1	44	1000	499872	18159

For peer review only

For peer review only - http://bmjopen.bmj.com/site/about/guidelines.xhtml

Appendix A

Table (46): Per 1000 distribution of ailing persons among those who were medically treated but not as inpatient of hospital during last 15 days by type of payment made for availing some services for each category of specific medical service for each State/UT

medical service: X-ray, ECG, EEG, Scan — Urban

state/ut	type of payment for services: medical service not received / required	services received: free	services received: partly free	services received: on payment	total	number of persons with ailments treated: estd. no. (00)	number of persons with ailments treated: sample
(1)	(2)	(3)	(4)	(5)	(6)	(7)	(8)
Andhra Pradesh	897	6	0	97	1000	18538	967
Arunachal Pradesh	466	0	0	534	1000	6	7
Assam	891	12	0	97	1000	1656	154
Bihar	932	2	0	66	1000	3970	188
Chhattisgarh	924	11	0	65	1000	1830	150
Delhi	921	26	1	52	1000	1483	105
Goa	992	8	0	0	1000	431	43
Gujarat	906	7	2	85	1000	11437	558
Haryana	967	2	2	29	1000	3988	253
Himachal Pradesh	930	36	11	23	1000	312	81
Jammu & Kashmir	847	45	2	106	1000	998	151
Jharkhand	860	28	0	111	1000	1694	121
Karnataka	968	2	1	29	1000	6748	515
Kerala	965	1	12	22	1000	17227	937
Madhya Pradesh	938	4	4	54	1000	8914	509
Maharashtra	929	13	1	58	1000	39640	1465
Manipur	887	0	0	113	1000	108	58
Meghalaya	893	21	0	86	1000	93	38
Mizoram	969	0	0	31	1000	14	24
Nagaland	1000	0	0	0	1000	125	34
Orissa	892	32	0	76	1000	1737	139
Punjab	936	1	6	56	1000	7775	386
Rajasthan	935	1	0	64	1000	6804	477
Sikkim	889	111	0	0	1000	5	10
Tamil Nadu	942	11	0	47	1000	17788	1051
Tripura	986	14	0	0	1000	234	69
Uttaranchal	977	2	0	21	1000	1045	95
Uttar Pradesh	965	2	2	32	1000	31389	1523
West Bengal	933	11	1	55	1000	23254	1089
A & N Islands	969	20	0	11	1000	59	34
Chandigarh	965	12	0	24	1000	400	128
Dadra & N. Haveli	1000	0	0	0	1000	7	7
Daman & Diu	948	0	0	52	1000	13	14
Lakshadweep	971	16	0	13	1000	39	71
Pondicherry	929	15	0	56	1000	810	115
ONES	949	10	0	41	1000	586	240
GUTs	943	14	0	43	1000	1328	369
all-India	936	8	2	54	1000	210571	11566

For peer review only - http://bmjopen.bmj.com/site/about/guidelines.xhtml

Appendix A

Table (46): Per 1000 distribution of ailing persons among those who were medically treated but not as inpatient of hospital during last 15 days by type of payment made for availing some services for each category of specific medical service for each State/UT

medical service: X-ray, ECG, EEG, Scan — Rural + Urban

state/ut	type of payment for services				total	number of persons with ailments treated	
	medical service not received / required	services received				estd. no. (00)	sample
		free	partly free	on payment			
(1)	(2)	(3)	(4)	(5)	(6)	(7)	(8)
Andhra Pradesh	927	8	1	65	1000	52854	2225
Arunachal Pradesh	867	20	32	81	1000	209	103
Assam	937	3	0	60	1000	13814	765
Bihar	947	12	0	42	1000	27598	993
Chhattisgarh	943	8	0	49	1000	12566	527
Delhi	924	25	1	50	1000	1552	108
Goa	998	2	0	0	1000	1935	78
Gujarat	924	4	2	70	1000	29585	1098
Haryana	955	2	2	41	1000	16704	722
Himachal Pradesh	914	5	3	78	1000	4629	681
Jammu & Kashmir	878	18	25	78	1000	4409	460
Jharkhand	907	11	0	82	1000	7128	326
Karnataka	939	8	0	53	1000	23147	1052
Kerala	961	5	7	27	1000	66825	2731
Madhya Pradesh	957	6	1	36	1000	32608	1232
Maharashtra	930	10	1	60	1000	84056	2713
Manipur	912	0	11	76	1000	410	162
Meghalaya	964	15	0	20	1000	388	93
Mizoram	922	0	0	78	1000	25	30
Nagaland	979	0	0	21	1000	347	101
Orissa	962	10	1	26	1000	19262	705
Punjab	970	1	3	26	1000	27561	985
Rajasthan	926	2	2	70	1000	27255	1291
Sikkim	970	21	0	9	1000	124	70
Tamil Nadu	954	15	0	31	1000	47280	1946
Tripura	983	1	3	13	1000	3308	517
Uttaranchal	918	2	0	81	1000	4095	200
Uttar Pradesh	954	2	1	42	1000	126786	4815
West Bengal	942	7	2	50	1000	72195	2460
A & N Islands	962	34	0	4	1000	159	64
Chandigarh	947	10	0	42	1000	447	165
Dadra & N. Haveli	1000	0	0	0	1000	39	19
Daman & Diu	979	0	0	21	1000	31	27
Lakshadweep	915	72	5	9	1000	59	115
Pondicherry	927	12	0	61	1000	1054	146
ONES	970	3	5	22	1000	4811	1076
GUTs	937	15	0	47	1000	1789	536
all-India	945	6	2	47	1000	710443	29725

For peer review only - http://bmjopen.bmj.com/site/about/guidelines.xhtml

Appendix A

Table (46): Per 1000 distribution of ailing persons among those who were medically treated but not as inpatient of hospital during last 15 days by type of payment made for availing some services for each category of specific medical service for each State/UT

medical service: other diagnostic tests Rural

state/ut	type of payment for services				total	number of persons with ailments treated	
	medical service not received / required	services received				estd. no. (00)	sample
		free	partly free	on payment			
(1)	(2)	(3)	(4)	(5)	(6)	(7)	(8)
Andhra Pradesh	886	13	1	100	1000	34311	1257
Arunachal Pradesh	655	212	20	113	1000	225	109
Assam	862	5	10	122	1000	12299	613
Bihar	895	13	0	91	1000	23581	806
Chhattisgarh	847	8	0	145	1000	10735	377
Delhi	1000	0	0	0	1000	69	3
Goa	1000	0	0	0	1000	1504	35
Gujarat	893	20	7	79	1000	18147	540
Haryana	931	2	1	66	1000	12716	469
Himachal Pradesh	857	38	6	98	1000	4317	600
Jammu & Kashmir	775	10	36	180	1000	3411	309
Jharkhand	786	0	17	196	1000	5361	200
Karnataka	851	26	5	117	1000	16399	537
Kerala	888	16	9	88	1000	49395	1788
Madhya Pradesh	898	7	2	93	1000	23691	722
Maharashtra	848	21	6	125	1000	44307	1246
Manipur	909	0	17	74	1000	300	104
Meghalaya	901	76	0	23	1000	295	55
Mizoram	1000	0	0	0	1000	11	6
Nagaland	825	122	0	52	1000	222	67
Orissa	849	26	0	125	1000	17518	565
Punjab	959	5	2	34	1000	19786	599
Rajasthan	863	10	1	126	1000	20451	814
Sikkim	927	10	0	63	1000	114	60
Tamil Nadu	841	63	0	96	1000	29491	895
Tripura	938	8	2	52	1000	3064	446
Uttaranchal	858	9	0	133	1000	3051	105
Uttar Pradesh	930	1	1	68	1000	94987	3281
West Bengal	923	5	2	70	1000	48971	1371
A & N Islands	817	183	0	0	1000	99	30
Chandigarh	813	3	0	183	1000	46	37
Dadra & N. Haveli	1000	0	0	0	1000	32	12
Daman & Diu	1000	0	0	0	1000	18	13
Lakshadweep	704	296	0	0	1000	20	44
Pondicherry	613	203	0	184	1000	244	31
ONES	912	29	4	55	1000	4230	847
GUTs	724	160	0	116	1000	460	167
all-India	891	14	3	92	1000	499190	18146

For peer review only - http://bmjopen.bmj.com/site/about/guidelines.xhtml

Appendix A

Table (46): Per 1000 distribution of ailing persons among those who were medically treated but not as inpatient of hospital during last 15 days by type of payment made for availing some services for each category of specific medical service for each State/UT

medical service: other diagnostic tests Urban

state/ut	type of payment for services				total	number of persons with ailments treated	
	medical service not received / required	services received				estd. no. (00)	sample
		free	partly free	on payment			
(1)	(2)	(3)	(4)	(5)	(6)	(7)	(8)
Andhra Pradesh	818	16	0	165	1000	18711	970
Arunachal Pradesh	0	4	7	989	1000	31	19
Assam	723	14	59	204	1000	1659	154
Bihar	917	1	0	82	1000	3969	187
Chhattisgarh	864	14	12	111	1000	1825	149
Delhi	789	64	1	146	1000	1483	105
Goa	857	8	2	133	1000	431	43
Gujarat	847	9	2	142	1000	11437	558
Haryana	903	0	3	94	1000	3988	253
Himachal Pradesh	813	152	7	28	1000	312	81
Jammu & Kashmir	646	23	3	328	1000	998	151
Jharkhand	843	21	0	136	1000	1662	120
Karnataka	839	6	2	152	1000	6748	515
Kerala	892	10	4	94	1000	17240	938
Madhya Pradesh	898	14	4	84	1000	8890	507
Maharashtra	878	11	11	100	1000	39630	1465
Manipur	678	0	0	322	1000	112	60
Meghalaya	863	21	0	116	1000	93	38
Mizoram	647	0	21	332	1000	17	23
Nagaland	853	96	0	51	1000	125	34
Orissa	803	46	0	151	1000	1737	139
Punjab	917	2	7	73	1000	7775	386
Rajasthan	856	13	0	131	1000	6804	477
Sikkim	799	28	111	63	1000	5	10
Tamil Nadu	827	58	3	111	1000	17788	1051
Tripura	920	14	0	66	1000	234	69
Uttaranchal	911	13	0	76	1000	1045	95
Uttar Pradesh	901	15	3	81	1000	31306	1515
West Bengal	907	13	2	78	1000	23187	1088
A & N Islands	905	75	0	20	1000	59	34
Chandigarh	912	14	0	74	1000	400	128
Dadra & N. Haveli	745	0	0	255	1000	7	7
Daman & Diu	656	0	0	344	1000	13	14
Lakshadweep	753	234	0	13	1000	39	71
Pondicherry	796	105	0	99	1000	810	115
ONES	799	28	2	170	1000	617	253
GUTs	833	79	0	88	1000	1328	369
all-India	872	17	5	107	1000	210569	11569

For peer review only - http://bmjopen.bmj.com/site/about/guidelines.xhtml

Appendix A

Table (46): Per 1000 distribution of ailing persons among those who were medically treated but not as inpatient of hospital during last 15 days by type of payment made for availing some services for each category of specific medical service for each State/UT

medical service: other diagnostic tests Rural + Urban

state/ut	type of payment for services				total	number of persons with ailments treated	
	medical service not received / required	services received				estd. no. (00)	sample
		free	partly free	on payment			
(1)	(2)	(3)	(4)	(5)	(6)	(7)	(8)
Andhra Pradesh	862	14	1	123	1000	53021	2227
Arunachal Pradesh	576	187	19	218	1000	256	128
Assam	846	6	16	132	1000	13958	767
Bihar	898	12	0	90	1000	27550	993
Chhattisgarh	850	9	2	140	1000	12561	526
Delhi	798	61	1	139	1000	1552	108
Goa	968	2	0	30	1000	1935	78
Gujarat	875	16	5	103	1000	29585	1098
Haryana	925	1	1	73	1000	16704	722
Himachal Pradesh	854	46	6	94	1000	4629	681
Jammu & Kashmir	746	13	28	214	1000	4409	460
Jharkhand	800	5	13	182	1000	7022	320
Karnataka	847	21	5	128	1000	23147	1052
Kerala	889	14	7	90	1000	66635	2726
Madhya Pradesh	898	9	2	91	1000	32581	1229
Maharashtra	862	16	8	113	1000	83937	2711
Manipur	846	0	12	142	1000	413	164
Meghalaya	892	63	0	45	1000	388	93
Mizoram	785	0	13	203	1000	27	29
Nagaland	835	113	0	52	1000	347	101
Orissa	845	28	0	127	1000	19254	704
Punjab	947	4	3	45	1000	27561	985
Rajasthan	861	11	1	127	1000	27255	1291
Sikkim	921	11	5	63	1000	119	70
Tamil Nadu	836	62	1	101	1000	47280	1946
Tripura	937	8	2	53	1000	3298	515
Uttaranchal	871	10	0	119	1000	4095	200
Uttar Pradesh	923	4	1	71	1000	126293	4796
West Bengal	918	8	2	72	1000	72158	2459
A & N Islands	850	143	0	8	1000	159	64
Chandigarh	902	13	0	85	1000	447	165
Dadra & N. Haveli	957	0	0	43	1000	39	19
Daman & Diu	860	0	0	140	1000	31	27
Lakshadweep	736	255	0	9	1000	59	115
Pondicherry	754	128	0	119	1000	1054	146
ONES	898	29	4	70	1000	4847	1100
GUTs	805	100	0	95	1000	1789	536
all-India	885	15	4	96	1000	709758	29715

For peer review only

For peer review only - http://bmjopen.bmj.com/site/about/guidelines.xhtml

Appendix A

Table (47): Per 1000 distribution of total household expenditure incurred during the last 15 days in connection with treatment (other than as inpatient of hospital) by source of finance for each mpce class and social group

all-India Rural

mpce class / social group	distn. of expenditure (Rs.) on treatment by source of finance					estd. total expen-diture (000) for treat-ment	persons medically treated during last 15 days	
	house-hold income/ savings	borro-wings	contri-bution from friends and relatives	other sources (incl.sale of ornaments and other physical assets, draught animals, etc.)	total		estd. no. (00)	sample
(1)	(2)	(3)	(4)	(5)	(6)	(7)	(8)	(9)
0 – 225	785	134	48	33	1000	353528	15647	494
225 – 255	688	214	80	18	1000	310049	11493	373
255 – 300	785	176	23	16	1000	684787	28799	918
300 – 340	689	262	28	22	1000	723704	25889	917
340 – 380	722	164	91	23	1000	924428	34654	1165
380 – 420	725	196	45	35	1000	1116262	36042	1285
420 – 470	740	177	53	30	1000	1244302	41610	1444
470 – 525	684	279	28	10	1000	1947329	43918	1596
525 – 615	806	147	40	7	1000	1438726	42540	1645
615 – 775	865	106	25	5	1000	1890425	50330	2039
775 – 950	869	116	14	1	1000	1183309	28874	1129
950 +	794	136	62	8	1000	1954718	35527	1473
all	772	172	42	14	1000	13771566	395324	14478
social -group								
SC	772	166	36	25	1000	573101	26793	1283
ST	736	202	50	12	1000	2440088	84121	2818
OBC	781	164	38	16	1000	5723570	164993	5901
others	778	168	42	12	1000	5027723	119326	4473
n.r.	1000	0	0	0	1000	7085	91	3
all	772	172	42	14	1000	13771566	395324	14478

For peer review only

For peer review only - http://bmjopen.bmj.com/site/about/guidelines.xhtml

Appendix A

Table (47): Per 1000 distribution of total household expenditure incurred during the last 15 days in connection with treatment (other than as inpatient of hospital) by source of finance for each mpce class and social group

all-India Urban

mpce class / social group	distn. of expenditure (Rs.) on treatment by source of finance					estd. total expen-diture (000) for treat-ment	persons medically treated during last 15 days	
	house-hold income/ savings	borro-wings	contri-bution from friends and relatives	other sources (incl.sale of ornaments and other physical assets, draught animals, etc.)	total		estd. no. (00)	sample
(1)	(2)	(3)	(4)	(5)	(6)	(7)	(8)	(9)
0 –300	717	244	39	0	1000	99464	3776	185
300 – 350	778	127	96	0	1000	117792	3596	165
350 – 425	820	147	31	2	1000	208926	8266	458
425 – 500	732	201	61	7	1000	501101	15836	876
500 – 575	851	116	28	5	1000	244017	7836	451
575 – 665	856	125	15	4	1000	383118	12402	710
665 – 775	843	99	26	32	1000	606923	18622	1017
775 – 915	932	38	14	15	1000	645158	16661	1011
915 – 1120	911	47	32	10	1000	736651	22234	1221
1120 – 1500	871	60	29	40	1000	1253445	24903	1366
1500 – 1925	964	12	21	3	1000	291118	7239	455
1925 +	862	11	123	4	1000	1186002	18822	883
all	865	71	48	16	1000	6273716	160193	8798
social -group								
SC	805	74	42	80	1000	71222	2742	325
ST	878	99	21	3	1000	764931	21958	1143
OBC	827	136	25	12	1000	1766430	53461	3031
others	881	35	65	19	1000	3670783	82022	4297
n.r.	1000	0	0	0	1000	350	10	2
all	865	71	48	16	1000	6273716	160193	8798

For peer review only

For peer review only - http://bmjopen.bmj.com/site/about/guidelines.xhtml

Appendix A

Table (47): Per 1000 distribution of total household expenditure incurred during the last 15 days in connection with treatment (other than as inpatient of hospital) by source of finance for each mpce class and social group

all-India Rural + Urban

mpce class / social group	distn. of expenditure (Rs.) on treatment by source of finance					estd. total expen-diture (000) for treat-ment	persons medically treated during last 15 days	
	house-hold income/ savings	borro-wings	contri-bution from friends and relatives	other sources (incl.sale of ornaments and other physical assets, draught animals, etc.)	total		estd. no. (00)	sample
(1)	(2)	(3)	(4)	(5)	(6)	(7)	(8)	(9)
0 – 235	771	157	46	26	1000	452992	19423	679
235 – 265	712	190	84	13	1000	427841	15089	538
265 – 320	793	170	25	13	1000	893713	37065	1376
320 – 365	706	237	41	16	1000	1224805	41725	1793
365 – 410	749	154	78	19	1000	1168445	42490	1616
410 – 460	759	177	37	27	1000	1499380	48444	1995
460 – 520	775	151	44	31	1000	1851225	60232	2461
520 – 605	751	213	24	11	1000	2592488	60579	2607
605 – 730	842	112	37	8	1000	2175377	64774	2866
730 – 980	867	87	27	19	1000	3143870	75233	3405
980 – 1285	888	95	15	2	1000	1474427	36113	1584
1285 +	821	87	86	6	1000	3140720	54349	2356
all	802	140	44	15	1000	20045282	555517	23276
social -group								
SC	776	156	37	32	1000	644323	29535	1608
ST	771	177	43	9	1000	3205019	106078	3961
OBC	792	157	35	15	1000	7490000	218454	8932
others	822	111	52	15	1000	8698506	201348	8770
n.r.	1000	0	0	0	1000	7434	101	5
all	802	140	44	15	1000	20045282	555517	23276

For peer review only - http://bmjopen.bmj.com/site/about/guidelines.xhtml

Appendix A

Table (48): Number per 1000 of treated persons getting reimbursement for treatment and per 1000 distribution of total amount of reimbursement by source of reimbursement for each sex, mpce class and social-group

all-India Rural

mpce class (Rs.) / social group	no. per 1000 of persons getting reim-burse-ment	reimbursement of total expenses (Rs.) incurred on treatment during last 15 days receivable from					estd. total amt. of reim-bur-sement (000)	treated persons getting reimbursement	
		employer		medical insurance companies	other agencies	total		estd. no. (00)	sample
		govern-ment	private						
(1)	(2)	(3)	(4)	(5)	(6)	(7)	(8)	(9)	(10)
**males**									
mpce class									
0 – 225	0	0	0	0	0	0	0	0	0
225 – 255	0	0	0	0	0	0	0	0	0
255 – 300	7	250	250	250	250	1000	13218	121	2
300 – 340	0	0	0	0	0	0	0	0	0
340 – 380	2	0	1000	0	0	1000	1961	34	2
380 – 420	1	638	0	0	362	1000	74	12	2
420 – 470	2	840	53	53	53	1000	178	52	2
470 – 525	0	1000	0	0	0	1000	832	6	1
525 – 615	0	0	0	0	0	0	0	0	0
615 – 775	3	846	41	0	112	1000	1553	100	4
775 – 950	3	1000	0	0	0	1000	6515	53	3
950 +	6	999	1	0	0	1000	4835	129	7
all	2	583	183	114	121	1000	29166	507	23
social - group									
SC	2	962	38	0	0	1000	2403	32	3
ST	0	1000	0	0	0	1000	120	8	1
OBC	2	498	220	137	145	1000	23269	245	12
others	3	880	37	37	45	1000	3374	223	7
all	2	583	183	114	121	1000	29166	507	23
**females**									
mpce class									
0 – 225	0	0	0	0	0	0	0	0	0
225 – 255	0	0	0	0	0	0	0	0	0
255 – 300	1	250	250	250	250	1000	736	18	1
300 – 340	0	0	0	0	0	0	0	0	0
340 – 380	1	0	500	0	500	1000	56	28	1
380 – 420	5	204	174	174	449	1000	2140	106	5
420 – 470	1	97	593	97	214	1000	261	27	4
470 – 525	0	45	955	0	0	1000	85	14	2
525 – 615	2	250	250	250	250	1000	10552	59	2
615 – 775	2	491	14	14	482	1000	1549	56	7
775 – 950	6	934	22	22	22	1000	2358	128	5
950 +	2	995	0	0	5	1000	3066	57	4
all	2	447	170	158	225	1000	20803	495	31

Continued

For peer review only - http://bmjopen.bmj.com/site/about/guidelines.xhtml

For peer review only

Appendix A

Table (48): Number per 1000 of treated persons getting reimbursement for treatment and per 1000 distribution of total amount of reimbursement by source of reimbursement for each sex, mpce class and social-group

all-India Rural

mpce class (Rs.) / social group	no. per 1000 of persons getting reim-burse-ment	reimbursement of total expenses (Rs.) incurred on treatment during last 15 days receivable from: employer – govern-ment	employer – private	medical insurance companies	other agencies	total	estd. total amt. of reim-bur-sement (000)	treated persons getting reimbursement: estd. no. (00)	sample
(1)	(2)	(3)	(4)	(5)	(6)	(7)	(8)	(9)	(10)
				females					
social - group									
SC	1	0	1000	0	0	1000	81	14	1
ST	2	423	188	188	200	1000	14659	127	9
OBC	2	451	121	121	307	1000	3459	164	9
others	2	591	104	43	262	1000	2604	191	12
all	2	447	170	158	225	1000	20803	495	31
				persons					
mpce class									
0 – 225	0	0	0	0	0	0	0	0	0
225 – 255	0	0	0	0	0	0	0	0	0
255 – 300	4	250	250	250	250	1000	13954	139	3
300 – 340	0	0	0	0	0	0	0	0	0
340 – 380	1	0	986	0	14	1000	2017	62	3
380 – 420	3	218	168	168	446	1000	2214	118	7
420 – 470	2	398	374	79	149	1000	439	79	6
470 – 525	0	911	89	0	0	1000	917	20	3
525 – 615	1	250	250	250	250	1000	10552	59	2
615 – 775	2	669	27	7	297	1000	3102	156	11
775 – 950	5	983	6	6	6	1000	8873	182	8
950 +	4	997	1	0	2	1000	7900	186	11
all	2	526	178	132	164	1000	49969	1002	54
social - group									
SC	1	931	69	0	0	1000	2485	45	4
ST	1	428	187	187	199	1000	14778	134	10
OBC	2	492	207	135	166	1000	26728	409	21
others	3	754	66	40	140	1000	5978	413	19
all	2	526	178	132	164	1000	49969	1002	54

For peer review only - http://bmjopen.bmj.com/site/about/guidelines.xhtml

Appendix A

Table (48): Number per 1000 of treated persons getting reimbursement for treatment and per 1000 distribution of total amount of reimbursement by source of reimbursement for each sex, mpce class and social-group

all-India Urban

mpce class (Rs.) / social group	no. per 1000 of persons getting reim-burse-ment	reimbursement of total expenses (Rs.) incurred on treatment during last 15 days receivable from: employer: govern-ment	employer: private	medical insurance companies	other agencies	total	estd. total amt. of reim-bur-sement (000)	treated persons getting reimbursement: estd. no. (00)	sample
(1)	(2)	(3)	(4)	(5)	(6)	(7)	(8)	(9)	(10)
**males**									
mpce class									
0 –300	0	0	0	0	0	0	0	0	0
300 – 350	0	0	0	0	0	0	0	0	0
350 – 425	0	0	0	0	0	0	0	0	0
425 – 500	10	0	1000	0	0	1000	1046	95	1
500 – 575	0	1000	0	0	0	1000	11	0	1
575 – 665	0	0	0	0	0	0	0	0	0
665 – 775	1	929	24	24	24	1000	183	9	3
775 – 915	21	967	30	1	1	1000	5176	207	10
915 – 1120	9	999	0	0	0	1000	3379	115	6
1120 – 1500	8	272	435	146	146	1000	5723	128	8
1500 – 1925	19	441	253	306	0	1000	4650	82	7
1925 +	21	391	116	6	487	1000	8752	223	17
all	9	539	204	81	177	1000	28920	860	53
social -group									
SC	12	990	3	3	3	1000	216	18	5
ST	3	248	0	0	752	1000	5669	42	8
OBC	9	500	223	257	19	1000	5985	296	12
others	10	644	267	46	43	1000	17050	504	28
all	9	539	204	81	177	1000	28920	860	53
**females**									
mpce class									
0 –300	0	0	0	0	0	0	0	0	0
300 – 350	0	0	0	0	0	0	0	0	0
350 – 425	0	0	0	0	0	0	0	0	0
425 – 500	1	1000	0	0	0	1000	6	6	1
500 – 575	3	0	0	0	1000	1000	923	15	1
575 – 665	1	0	0	0	1000	1000	44	7	1
665 – 775	1	566	0	0	434	1000	865	13	3
775 – 915	16	796	204	0	0	1000	23450	167	4
915 – 1120	2	385	573	0	42	1000	417	22	5
1120 – 1500	13	530	445	0	25	1000	11718	206	16
1500 – 1925	18	771	229	0	0	1000	3081	83	8
1925 +	19	896	102	1	1	1000	31952	235	12
all	7	781	196	0	23	1000	72456	755	51

Continued

For peer review only - http://bmjopen.bmj.com/site/about/guidelines.xhtml

Appendix A

Table (48): Number per 1000 of treated persons getting reimbursement for treatment and per 1000 distribution of total amount of reimbursement by source of reimbursement for each sex, mpce class and social-group

all-India Urban

mpce class (Rs.) / social group	no. per 1000 of persons getting reim-burse-ment	reimbursement of total expenses (Rs.) incurred on treatment during last 15 days receivable from					estd. total amt. of reim-bur-sement (000)	treated persons getting reimbursement	
		employer		medical insurance companies	other agencies	total		estd. no. (00)	sample
		govern-ment	private						
(1)	(2)	(3)	(4)	(5)	(6)	(7)	(8)	(9)	(10)
				females					
social -group									
SC	0	1000	0	0	0	1000	37	0	1
ST	2	1000	0	0	0	1000	2180	27	4
OBC	3	806	111	6	77	1000	5242	118	13
others	11	771	209	0	20	1000	64997	611	33
all	7	781	196	0	23	1000	72456	755	51
				persons					
mpce class									
0 –300	0	0	0	0	0	0	0	0	0
300 – 350	0	0	0	0	0	0	0	0	0
350 – 425	0	0	0	0	0	0	0	0	0
425 – 500	5	6	994	0	0	1000	1052	101	2
500 – 575	1	12	0	0	988	1000	934	15	2
575 – 665	0	0	0	0	1000	1000	44	7	1
665 – 775	1	629	4	4	362	1000	1048	22	6
775 – 915	18	827	172	0	0	1000	28626	375	14
915 – 1120	5	932	63	0	5	1000	3797	137	11
1120 – 1500	10	446	441	48	65	1000	17441	334	24
1500 – 1925	19	573	243	184	0	1000	7731	165	15
1925 +	20	788	105	2	105	1000	40704	458	29
all	8	712	198	23	67	1000	101375	1616	104
social -group									
SC	6	991	3	3	3	1000	253	18	6
ST	3	457	0	0	543	1000	7849	68	12
OBC	6	643	171	140	46	1000	11227	414	25
others	11	745	221	10	25	1000	82047	1115	61
all	8	712	198	23	67	1000	101375	1616	104

For peer review only - http://bmjopen.bmj.com/site/about/guidelines.xhtml

Appendix A

Table (48): Number per 1000 of treated persons getting reimbursement for treatment and per 1000 distribution of total amount of reimbursement by source of reimbursement for each sex, mpce class and social-group

all-India Rural + Urban

mpce class (Rs.) / social group	no. per 1000 of persons getting reim-burse-ment	reimbursement of total expenses (Rs.) incurred on treatment during last 15 days receivable from					estd. total amt. of reim-bur-sement (000)	treated persons getting reimbursement	
		employer		medical insurance companies	other agencies	total		estd. no. (00)	sample
		govern-ment	private						
(1)	(2)	(3)	(4)	(5)	(6)	(7)	(8)	(9)	(10)
**males**									
mpce class									
0 – 235	0	0	0	0	0	0	0	0	0
235 – 265	0	0	0	0	0	0	0	0	0
265 – 320	6	250	250	250	250	1000	13218	121	2
320 – 365	4	0	1000	0	0	1000	1046	95	1
365 – 410	1	6	994	0	0	1000	1972	34	3
410 – 460	0	638	0	0	362	1000	74	12	2
460 – 520	2	885	38	38	38	1000	361	62	5
520 – 605	6	971	26	1	1	1000	6008	213	11
605 – 730	3	999	0	0	0	1000	3379	115	6
730 – 980	5	395	351	115	139	1000	7276	228	12
980 – 1285	6	767	105	127	0	1000	11165	135	10
1285 +	11	607	75	4	314	1000	13586	352	24
all	4	561	193	97	149	1000	58085	1367	76
social -group									
SC	3	964	35	0	0	1000	2620	50	8
ST	1	264	0	0	736	1000	5789	50	9
OBC	4	498	221	162	119	1000	29254	541	24
others	6	683	229	45	43	1000	20423	727	35
all	4	561	193	97	149	1000	58085	1367	76
**females**									
mpce class									
0 – 235	0	0	0	0	0	0	0	0	0
235 – 265	0	0	0	0	0	0	0	0	0
265 – 320	1	250	250	250	250	1000	736	18	1
320 – 365	0	1000	0	0	0	1000	6	6	1
365 – 410	2	0	29	0	971	1000	978	43	2
410 – 460	4	199	170	170	460	1000	2183	114	6
460 – 520	1	457	138	22	383	1000	1126	40	7
520 – 605	5	794	206	0	0	1000	23535	181	6
605 – 730	2	255	262	240	242	1000	10969	81	7
730 – 980	5	526	394	2	78	1000	13267	262	23
980 – 1285	8	842	139	9	9	1000	5440	212	13
1285 +	7	905	93	1	1	1000	35018	293	16
all	3	706	190	36	68	1000	93259	1250	82

Continued

For peer review only - http://bmjopen.bmj.com/site/about/guidelines.xhtml

Table (48): Number per 1000 of treated persons getting reimbursement for treatment and per 1000 distribution of total amount of reimbursement by source of reimbursement for each sex, mpce class and social-group

all-India — Rural + Urban

mpce class (Rs.) / social group	no. per 1000 of persons getting reim-burse-ment	reimbursement of total expenses (Rs.) incurred on treatment during last 15 days receivable from: employer – govern-ment	employer – private	medical insurance companies	other agencies	total	estd. total amt. of reim-bur-sement (000)	treated persons getting reimbursement: estd. no. (00)	sample
(1)	(2)	(3)	(4)	(5)	(6)	(7)	(8)	(9)	(10)
social -group					females				
SC	1	310	690	0	0	1000	118	14	2
ST	2	498	164	164	174	1000	16838	153	13
OBC	2	665	115	51	169	1000	8701	282	22
others	6	764	205	2	29	1000	67601	802	45
all	3	706	190	36	68	1000	93259	1250	82
					persons				
mpce class									
0 – 235	0	0	0	0	0	0	0	0	0
235 – 265	0	0	0	0	0	0	0	0	0
265 – 320	3	250	250	250	250	1000	13954	139	3
320 – 365	2	6	994	0	0	1000	1052	101	2
365 – 410	1	4	674	0	322	1000	2950	76	5
410 – 460	2	214	165	165	456	1000	2258	126	8
460 – 520	1	561	113	26	299	1000	1487	102	12
520 – 605	5	830	170	0	0	1000	29543	394	17
605 – 730	2	430	201	184	185	1000	14349	196	13
730 – 980	5	479	379	42	100	1000	20542	491	35
980 – 1285	7	792	116	89	3	1000	16605	347	23
1285 +	9	822	88	2	89	1000	48604	645	40
all	4	650	191	59	99	1000	151344	2618	158
social -group									
SC	2	936	63	0	0	1000	2737	64	10
ST	2	438	122	122	318	1000	22627	203	22
OBC	3	537	197	136	130	1000	37955	823	46
others	6	745	211	12	32	1000	88025	1529	80
all	4	650	191	59	99	1000	151344	2618	158

For peer review only

For peer review only - http://bmjopen.bmj.com/site/about/guidelines.xhtml

Appendix A

Table (49): Number per 1000 of children (0 – 4 years) receiving any type of immunisation and average expenditure incurred by sex for each mpce class and social group

all-India Rural

mpce class (Rs.) / social group	number per 1000 of children (0 – 4 years) receiving any type of immunisation and average expenditure incurred						no. of children receiving any type of immunisation			
	boys		girls		children		estd. no. (00)		sample	
	no. per 1000 receiving any type of immunisation	average expenditure on immunisation (Rs. 0.0)	no. per 1000 receiving any type of immunisation	average expenditure on immunisation (Rs. 0.0)	no. per 1000 receiving any type of immunisation	average expenditure on immunisation (Rs. 0.0)	boys	girls	boys	girls
(1)	(2)	(3)	(4)	(5)	(6)	(7)	(8)	(9)	(10)	(11)
0 – 225	893	4.1	880	5.2	886	4.7	31070	32645	836	857
225 – 255	853	12.1	895	11.5	874	11.8	19830	22093	567	634
255 – 300	881	12	881	12.9	881	12.4	42862	44886	1260	1308
300 – 340	878	8.8	861	9.5	870	9.1	41333	38560	1251	1197
340 – 380	869	9.5	854	13.3	862	11.3	42150	37884	1270	1164
380 – 420	885	17.6	901	12.2	893	14.9	42999	42840	1358	1261
420 – 470	893	15	906	17.8	900	16.4	40916	40947	1364	1309
470 – 525	927	25.9	914	17.7	920	21.8	38570	38714	1358	1301
525 – 615	908	31	889	20.5	899	26	32882	29780	1341	1184
615 – 775	918	34	933	28.5	925	31.4	33323	29066	1341	1152
775 – 950	923	79.9	920	53.1	922	68.7	14262	10297	602	479
950 +	949	142	929	93.7	940	120.6	9774	7769	493	368
all	893	22.3	892	17.4	892	19.9	389970	375480	13041	12214
social-group										
ST	893	5.7	900	3.8	897	4.7	X	X	X	X
SC	896	11.8	888	10	892	10.9	X	X	X	X
OBC	886	28.5	881	19.5	883	24.1	X	X	X	X
others	905	28.9	909	27.2	907	28	X	X	X	X
all	893	22.3	892	17.4	892	19.9	X	X	X	X
estimated no. of children (00)	389970	X	375480	X	765450	X	X	X	X	X
sample children	13041	X	12214	X	25255	X	X	X	X	X

For peer review only - http://bmjopen.bmj.com/site/about/guidelines.xhtml

Appendix A

Table (49): Number per 1000 of children (0 – 4 years) receiving any type of immunisation and average expenditure incurred by sex for each mpce class and social group

all-India Urban

mpce class (Rs.) / social group	number per 1000 of children (0 – 4 years) receiving any type of immunisation and average expenditure incurred						no. of children receiving any type of immunisation			
	boys		girls		children		estd. no. (00)		sample	
	no. per 1000 receiving any type of immu-nisation	average expen-diture on immu-nisation (Rs. 0.0)	no. per 1000 receiving any type of immu-nisation	average expen-diture on immu-nisation (Rs. 0.0)	no. per 1000 receiving any type of immu-nisation	average expen-diture on immu-nisation (Rs. 0.0)	boys	girls	boys	girls
(1)	(2)	(3)	(4)	(5)	(6)	(7)	(8)	(9)	(10)	(11)
0 –300	895	36.9	919	6.2	907	21.4	4939	5004	255	223
300 – 350	905	18.2	886	19.5	897	18.8	3706	2906	182	163
350 – 425	887	26.8	900	12.4	893	20.2	9639	8183	477	440
425 – 500	919	18	945	19.5	932	18.8	15954	15127	874	816
500 – 575	940	42.6	935	26	938	34.5	7188	6903	435	381
575 – 665	951	41.1	953	65	952	52.2	11757	10180	673	588
665 – 775	939	67	932	65.5	936	66.3	12996	11392	818	682
775 – 915	952	85.3	942	104.5	948	94	11011	9142	747	651
915 – 1120	963	178.6	944	148.2	954	165.2	12600	9937	810	674
1120 – 1500	944	207.5	949	359.6	946	275.6	11023	8931	739	633
1500 – 1925	978	389.8	941	447.7	962	414.6	3044	2276	215	188
1925 +	928	615.8	980	796.2	948	686.6	4386	2828	281	222
all	934	109.2	937	118.3	936	113.4	108243	92810	6506	5661
social-group										
ST	952	30	966	71.7	959	51.4	X	X	X	X
SC	928	34.7	926	54.7	927	44.2	X	X	X	X
OBC	932	79.1	948	75.9	940	77.6	X	X	X	X
others	938	171.8	930	194.8	935	182	X	X	X	X
all	934	109.2	937	118.3	936	113.4	X	X	X	X
estimated no. of children (00)	108243	X	92810	X	201053	X	X	X	X	X
sample children	6506	X	5661	X	12167	X	X	X	X	X

For peer review only

For peer review only - http://bmjopen.bmj.com/site/about/guidelines.xhtml

Appendix A

Table (49): Number per 1000 of children (0 – 4 years) receiving any type of immunisation and average expenditure incurred by sex for each mpce class and social group

all-India Rural + Urban

mpce class (Rs.) / social group	number per 1000 of children (0 – 4 years) receiving any type of immunisation and average expenditure incurred: boys: no. per 1000 receiving any type of immunisation	boys: average expenditure on immunisation (Rs. 0.0)	girls: no. per 1000 receiving any type of immunisation	girls: average expenditure on immunisation (Rs. 0.0)	children: no. per 1000 receiving any type of immunisation	children: average expenditure on immunisation (Rs. 0.0)	no. of children receiving any type of immunisation: estd. no. (00): boys	estd. no. (00): girls	sample: boys	sample: girls
(1)	(2)	(3)	(4)	(5)	(6)	(7)	(8)	(9)	(10)	(11)
0 – 235	893	8.6	885	5.3	889	6.9	36009	37649	1091	1080
235 – 265	861	13.1	894	12.4	877	12.7	23536	24999	749	797
265 – 320	882	14.7	884	12.8	883	13.7	52501	53069	1737	1748
320 – 365	889	11.3	884	12.3	886	11.8	57288	53687	2125	2013
365 – 410	879	14.3	865	15.2	872	14.7	49338	44787	1705	1545
410 – 460	898	22.7	910	22.4	904	22.5	54756	53020	2031	1849
460 – 520	904	27.6	912	28.2	908	27.9	53912	52339	2182	1991
520 – 605	933	39.1	919	34.3	926	36.7	49580	47855	2105	1952
605 – 730	923	71.9	902	52.5	913	62.8	45482	39717	2151	1858
730 – 980	925	77.1	937	106.3	930	90.6	44345	37997	2080	1785
980 – 1285	932	134.5	924	124.5	929	130.3	17306	12573	817	667
1285 +	942	288.7	942	281.2	942	285.5	14160	10597	774	590
all	902	41.2	900	37.4	901	39.3	498213	468290	19547	17875
social-group										
ST	897	7.2	904	8	900	7.6	X	X	X	X
SC	901	15.8	894	17.4	898	16.6	X	X	X	X
OBC	895	38.5	893	30.2	894	34.5	X	X	X	X
others	915	75.3	915	74.7	915	75	X	X	X	X
all	902	41.2	900	37.4	901	39.3	X	X	X	X
estimated no. of children (00)	498213	X	468290	X	966503	X	X	X	X	X
sample children	19547	X	17875	X	37422	X	X	X	X	X

For peer review only - http://bmjopen.bmj.com/site/about/guidelines.xhtml

Appendix A

Table (50): Per 1000 number of ever married women aged 15 - 49 yrs. who were pregnant any time during last 365 days, their distribution by status of pregnancy and place of childbirth, and average expenditure incurred per case of childbirth, separately for each age-group, mpce class and social group

all-India Rural

age-group of ever married women mpce class (Rs.)/ social group	no. per 1000 of women who were pregnant at some time during last 365 days	status of pregnancy of ever married women on the date of survey (and place of childbirth)						average expenditure on childbirth (Rs.)				ever married women (15-49 yrs) who were pregnant at some time during last 365 days	
		child-birth not taken place	childbirth taken place			n.r.	total	govt. hos-pital	pri-vate hos-pital	at home	all	estd. no. (00)	sample
			in govt. hospital	in private hospital	at home								
(1)	(2)	(3)	(4)	(5)	(6)	(7)	(8)	(9)	(10)	(11)	(12)	(13)	(14)
15 - 19	250	366	137	109	378	10	1000	1029	3323	330	1007	18363	618
20 - 24	289	268	141	142	439	11	1000	1145	4310	429	1333	73625	2622
25 - 29	175	272	138	115	458	17	1000	1168	4146	440	1181	49172	1826
30 - 34	99	226	123	109	537	5	1000	953	4343	424	1066	26565	908
35 - 39	45	232	62	45	622	39	1000	2050	3123	334	652	10381	371
40 - 44	19	128	100	67	575	131	1000	3249	3902	372	1077	3359	123
45 - 49	14	161	46	67	649	77	1000	705	1989	399	557	2055	69
all (15-49)	127	267	131	119	467	16	1000	1165	4137	414	1169	183521	6537
mpce class													
0 – 225	154	171	77	51	664	37	1000	765	2232	329	494	12004	340
225 – 255	142	215	76	52	642	16	1000	652	2886	418	606	8738	261
255 – 300	146	163	115	81	605	37	1000	990	1951	352	605	19712	593
300 – 340	133	247	139	64	532	18	1000	609	2801	444	679	16826	583
340 – 380	145	235	182	51	516	17	1000	607	3003	351	593	20455	620
380 – 420	119	241	125	117	509	9	1000	1234	2790	461	952	17818	631
420 – 470	126	241	165	109	480	5	1000	1231	3630	383	1037	19057	664
470 – 525	129	323	118	147	404	9	1000	1506	2825	462	1165	20330	728
525 – 615	115	320	146	153	367	14	1000	1432	4163	550	1573	17061	718
615 – 775	114	350	139	197	307	8	1000	1479	5505	433	2216	16873	711

Continued

For peer review only - http://bmjopen.bmj.com/site/about/guidelines.xhtml

Appendix A

Table (50): Per 1000 number of ever married women aged 15 - 49 yrs. who were pregnant any time during last 365 days, their distribution by status of pregnancy and place of childbirth, and average expenditure incurred per case of childbirth, separately for each age-group, mpce class and social group

all-India Rural

age-group of ever married women mpce class (Rs.)/ social group	no. per 1000 of women who were pregnant at some time during last 365 days	status of pregnancy of ever married women on the date of survey (and place of childbirth)						average expenditure on childbirth (Rs.)				ever married women (15-49 yrs) who were pregnant at some time during last 365 days	
		child-birth not taken place	childbirth taken place			n.r.	total	govt. hos-pital	pri-vate hos-pital	at home	all	estd. no. (00)	sample
			in govt. hospital	in private hospital	at home								
(1)	(2)	(3)	(4)	(5)	(6)	(7)	(8)	(9)	(10)	(11)	(12)	(13)	(14)
775 – 950	118	373	119	237	257	13	1000	2343	7611	522	3616	8674	381
950 +	85	473	79	327	111	10	1000	2628	6087	570	4371	5975	307
all	127	267	131	119	467	16	1000	1165	4137	414	1169	183521	6537
social group:													
ST	131	234	113	46	599	8	1000	675	3082	264	496	18968	946
SC	141	239	151	84	518	8	1000	935	3212	408	825	43212	1303
OBC	125	273	124	134	456	13	1000	1236	3673	446	1189	75889	2605
others	119	297	130	158	382	32	1000	1485	5385	456	1819	45453	1683
all	127	267	131	119	467	16	1000	1165	4137	414	1169	183521	6537

For peer review only - http://bmjopen.bmj.com/site/about/guidelines.xhtml

Appendix A

Table (50): Per 1000 number of ever married women aged 15 - 49 yrs. who were pregnant any time during last 365 days, their distribution by status of pregnancy and place of childbirth, and average expenditure incurred per case of childbirth, separately for each age-group, mpce class and social group

all-India Urban

age-group of ever married women mpce class (Rs.)/ social group	no. per 1000 of women who were pregnant at some time during last 365 days	status of pregnancy of ever married women on the date of survey (and place of childbirth)						average expenditure on childbirth (Rs.)				ever married women (15-49 yrs) who were pregnant at some time during last 365 days	
		child-birth not taken place	childbirth taken place			n.r.	total	govt. hos-pital	pri-vate hos-pital	at home	all	estd. no. (00)	sample
			in govt. hospital	in private hospital	at home								
(1)	(2)	(3)	(4)	(5)	(6)	(7)	(8)	(9)	(10)	(11)	(12)	(13)	(14)
15 - 19	293	445	184	212	159	0	1000	706	7240	391	3106	3480	183
20 - 24	323	308	226	279	185	2	1000	880	4961	556	2441	22983	1289
25 - 29	178	283	218	344	150	5	1000	965	5291	545	2968	15966	1037
30 - 34	69	254	192	339	202	14	1000	1696	6245	470	3462	6281	410
35 - 39	23	201	185	236	287	92	1000	979	7892	889	3245	2075	152
40 - 44	6	182	52	114	528	125	1000	470	13768	517	2689	459	36
45 - 49	6	149	465	221	42	123	1000	1341	3226	299	1852	347	20
all (15-49)	107	296	215	298	181	10	1000	994	5480	552	2806	51591	3127
mpce class													
0 –300	157	170	309	53	429	39	1000	671	4845	466	841	2287	112
300 – 350	155	197	321	192	290	0	1000	628	1799	614	903	1736	95
350 – 425	115	200	268	233	297	2	1000	724	3633	447	1469	3396	206
425 – 500	137	294	228	201	270	6	1000	790	3790	512	1543	7819	428
500 – 575	145	291	186	235	279	10	1000	928	3058	591	1508	3911	202
575 – 665	110	239	343	190	224	4	1000	781	3837	488	1462	4722	306
665 – 775	112	298	265	247	188	1	1000	1021	7280	614	3119	6487	388
775 – 915	103	274	251	332	129	14	1000	949	4754	666	2673	5395	351
915 – 1120	99	357	130	413	75	25	1000	2653	4926	530	3913	6320	413
1120 – 1500	82	328	112	530	25	5	1000	989	6095	1566	5070	5365	362

Continued

For peer review only - http://bmjopen.bmj.com/site/about/guidelines.xhtml

For peer review only

Appendix A

Table (50): Per 1000 number of ever married women aged 15 - 49 yrs. who were pregnant any time during last 365 days, their distribution by status of pregnancy and place of childbirth, and average expenditure incurred per case of childbirth, separately for each age-group, mpce class and social group

all-India Urban

age-group of ever married women mpce class (Rs.)/ social group	no. per 1000 of women who were pregnant at some time during last 365 days	status of pregnancy of ever married women on the date of survey (and place of childbirth)						average expenditure on childbirth (Rs.)				ever married women (15-49 yrs) who were pregnant at some time during last 365 days	
		child-birth not taken place	childbirth taken place			n.r.	total	govt. hos-pital	pri-vate hos-pital	at home	all	estd. no. (00)	sample
			in govt. hospital	in private hospital	at home								
(1)	(2)	(3)	(4)	(5)	(6)	(7)	(8)	(9)	(10)	(11)	(12)	(13)	(14)
1500 – 1925	88	415	99	483	2	1	1000	1208	7976	1222	6807	1909	127
1925 +	57	486	51	439	16	8	1000	1470	10471	79	9239	2245	137
all	107	296	215	298	181	10	1000	994	5480	552	2806	51591	3127
social group:													
ST	143	218	357	162	252	11	1000	813	5458	388	1650	1663	215
SC	124	288	273	176	253	9	1000	737	5070	506	1741	9388	499
OBC	116	299	188	297	209	7	1000	852	5299	637	2691	20281	1136
others	92	305	204	367	113	12	1000	1312	5719	472	3539	20258	1277
all	107	296	215	298	181	10	1000	994	5480	552	2806	51591	3127

For peer review only - http://bmjopen.bmj.com/site/about/guidelines.xhtml

Appendix A

Table (50): Per 1000 number of ever married women aged 15 - 49 yrs. who were pregnant any time during last 365 days, their distribution by status of pregnancy and place of childbirth, and average expenditure incurred per case of childbirth, separately for each age-group, mpce class and social group

all-India Rural + Urban

age-group of ever married women mpce class (Rs.)/ social group	no. per 1000 of women who were pregnant at some time during last 365 days	status of pregnancy of ever married women on the date of survey (and place of childbirth)						average expenditure on childbirth (Rs.)				ever married women (15-49 yrs) who were pregnant at some time during last 365 days	
		child-birth not taken place	childbirth taken place			n.r.	total	govt. hos-pital	pri-vate hos-pital	at home	all	estd. no. (00)	sample
			in govt. hospital	in private hospital	at home								
(1)	(2)	(3)	(4)	(5)	(6)	(7)	(8)	(9)	(10)	(11)	(12)	(13)	(14)
15 - 19	256	379	145	125	343	8	1000	963	4376	334	1310	21843	801
20 - 24	297	277	161	174	378	8	1000	1056	4557	444	1587	96608	3911
25 - 29	175	274	157	171	383	14	1000	1099	4710	450	1619	65138	2863
30 - 34	91	231	136	153	473	7	1000	1154	5147	428	1507	32846	1318
35 - 39	39	227	82	77	566	48	1000	1650	5553	380	1073	12456	523
40 - 44	15	134	94	72	570	130	1000	3065	5767	388	1259	3818	159
45 - 49	12	159	106	89	562	84	1000	1107	2431	398	737	2402	89
all (15-49)	122	273	149	158	404	15	1000	1111	4692	428	1519	235113	9664
mpce class													
0 – 235	155	171	114	52	627	37	1000	724	2662	344	550	14291	452
235 – 265	144	212	117	75	583	13	1000	641	2424	434	657	10474	356
265 – 320	140	169	137	103	560	32	1000	914	2509	359	731	23108	799
320 – 365	134	262	167	107	449	15	1000	687	3388	457	944	24645	1011
365 – 410	145	244	182	80	478	15	1000	659	3029	373	732	24366	822
410 – 460	117	240	171	132	449	8	1000	1044	3106	464	1059	22540	937
460 – 520	122	255	190	144	406	4	1000	1157	5221	410	1537	25544	1052
520 – 605	123	313	146	186	346	10	1000	1304	3549	478	1497	25725	1079
605 – 730	110	330	142	223	288	17	1000	1734	4544	549	2172	23381	1131
730 – 980	104	344	132	278	239	7	1000	1379	5777	461	2924	22238	1073

Continued

For peer review only - http://bmjopen.bmj.com/site/about/guidelines.xhtml

Appendix A

Table (50): Per 1000 number of ever married women aged 15 - 49 yrs. who were pregnant any time during last 365 days, their distribution by status of pregnancy and place of childbirth, and average expenditure incurred per case of childbirth, separately for each age-group, mpce class and social group

all-India Rural + Urban

age-group of ever married women mpce class (Rs.)/ social group	no. per 1000 of women who were pregnant at some time during last 365 days	status of pregnancy of ever married women on the date of survey (and place of childbirth)						average expenditure on childbirth (Rs.)				ever married women (15-49 yrs) who were pregnant at some time during last 365 days	
		child-birth not taken place	childbirth taken place			n.r.	total	govt. hos-pital	pri-vate hos-pital	at home	all	estd. no. (00)	sample
			in govt. hospital	in private hospital	at home								
(1)	(2)	(3)	(4)	(5)	(6)	(7)	(8)	(9)	(10)	(11)	(12)	(13)	(14)
980 – 1285	111	381	116	282	211	11	1000	2167	7724	523	4168	10582	508
1285 +	75	477	71	357	85	9	1000	2403	7558	544	5680	8219	444
all	122	273	149	158	404	15	1000	1111	4692	428	1519	235113	9664
social group:													
ST	132	233	133	55	571	8	1000	705	3644	269	591	20631	1161
SC	138	248	173	100	471	9	1000	879	3796	418	979	52600	1802
OBC	123	278	138	169	404	11	1000	1125	4278	467	1498	96170	3741
others	109	300	153	223	299	26	1000	1414	5555	458	2357	65711	2960
all	122	273	149	158	404	15	1000	1111	4692	428	1519	235113	9664

For peer review only - http://bmjopen.bmj.com/site/about/guidelines.xhtml

Appendix A

Table (51): (i) Per 1000 distribution of ever married pregnant women (15 - 49 yrs.) during last 365 days, by source of receiving pre-natal care, (ii) per 1000 distribution of ever married pregnant women (15 - 49 yrs.) who gave child birth by source of receiving post-natal care, and (iii) average expenditure on pre-natal care and post-natal care by type of institution for each age-group

all-India Rural

age-group of ever married women	per 1000 distribution of pregnant women by source of receiving pre-natal care					average expenditure incurred on pre-natal care (Rs.)			per 1000 distribution of pregnant women by source of receiving post-natal care					average expenditure incurred on post-natal care (Rs.)			ever married pregnant women (15-49 yrs.)	
	not received	received		n.r.	all				not received	received		n.r.	all				estd. no. (00)	sample
		from govt. source	from private source			from govt. source	from private source	all		from govt. source	from private source			from govt. source	from private source	all		
(1)	(2)	(3)	(4)	(5)	(6)	(7)	(8)	(9)	(10)	(11)	(12)	(13)	(14)	(15)	(16)	(17)	(18)	(19)
15 - 19	235	504	255	6	1000	226	998	486	380	308	310	3	1000	222	571	397	18363	618
20 - 24	289	425	285	1	1000	247	950	529	361	286	344	10	1000	219	626	441	73625	2622
25 - 29	283	441	271	5	1000	203	901	469	372	290	326	12	1000	306	483	400	49172	1826
30 - 34	324	411	261	4	1000	234	941	509	373	248	371	7	1000	187	511	381	26565	908
35 - 39	434	332	234	0	1000	112	855	419	404	244	351	1	1000	132	319	242	10381	371
40 - 44	420	291	288	0	1000	678	356	518	496	206	294	5	1000	139	567	391	3359	123
45 - 49	641	109	249	1	1000	328	348	342	506	134	350	10	1000	67	211	171	2055	69
all	302	424	272	3	1000	230	918	499	374	277	340	9	1000	232	541	402	183521	6537

For peer review only

For peer review only - http://bmjopen.bmj.com/site/about/guidelines.xhtml

Appendix A

Table (51): (i) Per 1000 distribution of ever married pregnant women (15 - 49 yrs.) during last 365 days, by source of receiving pre-natal care, (ii) per 1000 distribution of ever married pregnant women (15 - 49 yrs.) who gave child birth by source of receiving post-natal care, and (iii) average expenditure on pre-natal care and post-natal care by type of institution for each age-group

all-India Urban

age-group of ever married women	per 1000 distribution of pregnant women by source of receiving pre-natal care					average expenditure incurred on pre-natal care (Rs.)			per 1000 distribution of pregnant women by source of receiving post-natal care					average expenditure incurred on post-natal care (Rs.)			ever married pregnant women (15-49 yrs.)	
	not recei-ved	received		n.r.	all	from govt. source	from private source	all	not rece-ived	received		n.r.	all	from govt. source	from private source	all	estd. no. (00)	sam-ple
		from govt. source	from private source							from govt. source	from private source							
(1)	(2)	(3)	(4)	(5)	(6)	(7)	(8)	(9)	(10)	(11)	(12)	(13)	(14)	(15)	(16)	(17)	(18)	(19)
15 - 19	188	403	408	0	1000	193	1637	920	372	256	372	0	1000	209	837	581	3480	183
20 - 24	149	400	445	5	1000	353	1345	875	253	322	411	14	1000	343	652	516	22983	1289
25 - 29	139	374	486	1	1000	330	1250	850	247	299	440	14	1000	341	778	602	15966	1037
30 - 34	199	352	449	0	1000	460	1835	1231	324	249	425	2	1000	666	1025	892	6281	410
35 - 39	275	411	314	0	1000	476	1028	715	337	357	287	19	1000	251	786	489	2075	152
40 - 44	512	259	230	0	1000	19	1100	527	363	153	336	149	1000	410	1215	963	459	36
45 - 49	302	292	406	0	1000	1545	745	1080	275	354	371	0	1000	368	911	646	347	20
all	164	385	448	3	1000	356	1377	905	271	302	414	13	1000	367	762	595	51591	3127

For peer review only

For peer review only - http://bmjopen.bmj.com/site/about/guidelines.xhtml

Appendix A

Table (51): (i) Per 1000 distribution of ever married pregnant women (15 - 49 yrs.) during last 365 days, by source of receiving pre-natal care, (ii) per 1000 distribution of ever married pregnant women (15 - 49 yrs.) who gave child birth by source of receiving post-natal care, and (iii) average expenditure on pre-natal care and post-natal care by type of institution for each age-group

all-India | Rural + Urban

age-group of ever married women	per 1000 distribution of pregnant women by source of receiving pre-natal care					average expenditure incurred on pre-natal care (Rs.)			per 1000 distribution of pregnant women by source of receiving post-natal care					average expenditure incurred on post-natal care (Rs.)			ever married pregnant women (15-49 yrs.)	
	not recei-ved	received		n.r.	all	from govt. source	from private source	all	not rece-ived	received		n.r.	all	from govt. source	from private source	all	estd. no. (00)	sam-ple
		from govt. source	from private source							from govt. source	from private source							
(1)	(2)	(3)	(4)	(5)	(6)	(7)	(8)	(9)	(10)	(11)	(12)	(13)	(14)	(15)	(16)	(17)	(18)	(19)
15 - 19	227	488	280	5	1000	222	1147	559	379	300	319	2	1000	220	616	424	21843	801
20 - 24	256	419	323	2	1000	271	1079	623	336	294	359	11	1000	250	632	460	96608	3911
25 - 29	248	425	323	4	1000	231	1030	576	341	292	354	13	1000	315	573	456	65138	2863
30 - 34	300	400	297	3	1000	272	1199	667	364	248	381	6	1000	275	616	482	32846	1318
35 - 39	407	345	247	0	1000	184	892	479	393	263	341	4	1000	158	382	285	12456	523
40 - 44	431	287	281	0	1000	607	429	519	481	200	299	21	1000	162	649	454	3818	159
45 - 49	592	135	272	1	1000	708	434	525	474	165	353	8	1000	157	314	264	2402	89
all	271	415	310	3	1000	256	1064	601	352	283	356	10	1000	263	596	449	235113	9664

For peer review only - http://bmjopen.bmj.com/site/about/guidelines.xhtml

Note on Sample Design and Estimation Procedure

1.0 Introduction

1.1 The National Sample Survey (NSS), set up by the Government of India in 1950 to collect socio-economic data employing scientific sampling methods, started its sixtieth round from 1st January 2004.

1.2 Sixtieth round of NSS was earmarked for survey on 'Household Consumer Expenditure', 'Employment and Unemployment' and 'Morbidity and Health care'. Survey on household consumer expenditure was conducted as a part of annual series of data on the subject. Two types of schedule on annual household consumer expenditure were canvassed in the current round. For information on employment-unemployment situation, a separate schedule was canvassed. Information relating to morbidity, problems of aged persons, utilisation of health care services and expenditure on medical treatment were collected through 'Morbidity and Health Care' schedule.

2.1 **Subject Coverage:** The 60th round (January-June 2004) of NSS covered annual survey of consumer expenditure, employment-unemployment situation and morbidity and health care. In this round a separate schedule was canvassed for employment and unemployment.

2.2 Geographical coverage: The survey covered the whole of the Indian Union *except* (i) Leh (Ladakh) and Kargil districts of Jammu & Kashmir, (ii) interior villages of Nagaland situated beyond five kilometres of the bus route and (iii) villages in Andaman and Nicobar Islands which remain inaccessible throughout the year.

2.3 Period of survey and work programme: The period of survey was of six months duration starting on 1st January 2004 and ending on 30th June 2004. The survey period of this round had been divided into two sub-rounds of three months duration each as follows:

sub-round 1	:	January - March 2004
sub-round 2	:	April - June 2004

In each of these two sub-rounds equal number of sample villages/blocks (FSUs) had been allotted for survey with a view to ensuring uniform spread of sample FSUs over the entire survey period. Attempt had been made to survey each of the FSUs during the sub-round to which it was allotted. *Because of the arduous field conditions, this restriction could not be strictly enforced in Andaman and Nicobar Islands, Lakshadweep, rural areas of Arunachal Pradesh and Nagaland.*

2.4 Schedules of enquiry: During this round, the following schedules of enquiry were canvassed:

Schedule 0.0	:	list of households
Schedule 1.0	:	consumer expenditure
Schedule 10	:	employment and unemployment
Schedule 25.0	:	morbidity and health care

For peer review only - http://bmjopen.bmj.com/site/about/guidelines.xhtml

It was decided that two types of Schedule 1.0 viz. Schedule Type 1 and Schedule Type 2 would be canvassed in this round. Schedule Type 1 was same as Schedule 1.0 of last few rounds including that of NSS 59th round. Schedule Type 2 had different reference period (7 days) for some items of food, pan, tobacco and intoxicants as compared to 30 days for block 6, Schedule Type 1.

2.5 Participation of States: In this round all the States and Union Territories except Andaman & Nicobar Islands, Dadra & Nagar Haveli and Lakshadweep participated at least on an equal matching basis. The following was the matching pattern of the participating States/UTs.

Nagaland (U)	: triple
J & K , Manipur & Delhi	: double
Goa, Maharashtra (U)	: one and half
Remaining States/UTs	: equal

3.0 Sample Design

3.1 Outline of sample design: A stratified multi-stage design had been adopted for the 60th round survey. The first stage units (FSU) were the 1991 census villages in the rural sector and Urban Frame Survey (UFS) blocks in the urban sector. The ultimate stage units (USU) were households in both the sectors. In case of large villages/blocks requiring hamlet-group (hg)/sub-block (sb) formation, one intermediate stage was the selection of two hgs/sbs from each FSU.

3.2 Sampling Frame for First Stage Units: *For the rural sector,* the list of Census 1991 villages (panchayat wards for Kerala and Census 1981 villages for J & K) constituted the sampling frame. *For the urban sector*, the list of latest available Urban Frame Survey (UFS) blocks had been considered as the sampling frame.

3.3 Stratification

3.3.1 **Rural sector**: Two *special strata* were formed at the State/ UT level**,** viz.

Stratum 1: all FSUs with population between 0 to 50 and
Stratum 2: FSUs with population more than 15,000 as per census 1991.

Special stratum 1 had been formed if at least 50 such FSUs were found in a State/UT. Similarly, special stratum 2 had been formed if at least 4 such FSUs were found in a State/UT. Otherwise, such FSUs were merged with the general strata.

From FSUs other than those covered under special strata 1 and 2, *general strata* were formed and its numbering started from 3. Each district of a State/UT was normally treated as a separate stratum. However, if the census rural population of the district was greater than or equal to 2.5 million as per population census 2001 or 2 million as per population census 1991, the district had been split into two or more strata, by grouping contiguous tehsils to form strata. However, in Gujarat, some districts were not wholly included in an NSS region. In such cases, the part of the district falling in an NSS region constituted a separate stratum.

For peer review only - http://bmjopen.bmj.com/site/about/guidelines.xhtml

3.3.2 **Urban sector**: In the urban sector, strata were formed within each NSS region on the basis of size class of towns as per Population Census 2001. The stratum numbers and their composition (within each region) are given below.

stratum 1 :	all towns with population less than 50,000
stratum 2 :	all towns with population 50,000 or more but less than 2 lakhs
stratum 3 :	all towns with population 2 lakhs or more but less than 10 lakhs
stratum 4, 5, 6,...:	each town with population 10 lakhs or more

The stratum numbers had been kept as above even if, in some regions, some of the strata were not formed.

3.4 Total sample size (FSUs): 7612 FSUs were allocated at all-India level on the basis of investigator strength in different States/UTs for central sample and 8188 for state sample.

3.5 Allocation of total sample to States and UTs: The total number of sample FSUs was allocated to the States and UTs in proportion to provisional population as per Census 2001 subject to the availability of investigators ensuring more or less uniform work-load.

3.6 Allocation of State/UT level sample to rural and urban sectors: State/UT level sample had been allocated between two sectors in proportion to provisional population as per *Census 2001* with 1.5 weightage to urban sector subject to the restriction that urban sample size for bigger states like Maharashtra, Tamil Nadu etc. would not exceed the rural sample size. Earlier practice of giving double weightage to urban sector was modified considering the fact that there had been considerable growth in urban population. A minimum of 8 FSUs was allocated to each state/UT separately for rural and urban areas.

The sample sizes by sector and State/UT are given in the Appendix Table 1.

3.7 Allocation to strata: Within each sector of a State/UT, the respective sample size had been allocated to the different strata in proportion to the stratum population as per census 2001. Allocations at stratum level were adjusted to a multiple of 4 with a minimum sample size of 4.

3.8 Selection of FSUs: FSUs were selected with Probability Proportional to Size With Replacement (PPSWR), size being the population as per Population Census 1991 in all the strata for rural sector except for stratum 1. In stratum 1 of rural sector and in all the strata of urban sector, selection was done using Simple Random Sampling Without Replacement (SRSWOR). Within each stratum, samples had been drawn in the form of two independent sub-samples in both the rural and urban sectors.

3.9 Selection of hamlet-groups/sub-blocks/households

3.9.1 Criterion for hamlet-group/sub-block formation: Large villages/blocks having approximate population of 1200 or more were divided into a suitable number (say, D) of 'hamlet-groups' in the rural sector and 'sub-blocks' in the urban sector as stated below.

For peer review only - http://bmjopen.bmj.com/site/about/guidelines.xhtml

approximate present population of the sample village/block		no. of hgs/sbs to be formed (D)
less than 1200	(no hamlet-groups/sub-blocks)	1
1200 to 1799		3
1800 to 2399		4
2400 to 2999		5
3000 to 3599		6
…………..and so on		

For rural areas of Himachal Pradesh, Sikkim and Poonch, Rajouri, Udhampur, Doda districts of Jammu and Kashmir and Idukki district of Kerala, the number of hamlet-groups were formed as follows.

approximate present population of the sample village		no. of hgs to be formed
less than 600	(no hamlet-groups)	1
600 to 899		3
900 to 1199		4
1200 to 1499		5
………..and so on		

Two hamlet-groups/sub-blocks were selected from a large village/UFS block, wherever hamlet-groups/sub-blocks were formed, by SRSWOR. Listing and selection of the households were done independently in the two selected hamlet-groups/sub-blocks.

4.0 Formation of Second Stage Strata and allocation of households:

4.1 Schedule 25.0: In each selected village/block/hamlet-group/sub-block, four second stage strata (SSS) were formed as given below.

composition of SSS		number of households to be surveyed for schedule 25.0	
		without hg/sb formation	with hg/sb formation (for each hg/sb)
SSS 1:	households with at least one member hospitalised during last 365 days	4	2
SSS 2:	from the remaining households, households having at least one child of age below 5 years	2	1
SSS 3:	from the remaining households, households with at least one member of age 60 years or above	2	1
SSS 4:	other households	2	1

For peer review only - http://bmjopen.bmj.com/site/about/guidelines.xhtml

4.2 **Selection of households:** From each SSS the sample households for all the schedules were selected by SRSWOR. If a household was selected for more than one schedule only one schedule was canvassed in that household in the priority order of Schedule 1.0, Schedule 10 and Schedule 25.0 and in that case the household was replaced for the other schedule. If a household had been selected for Schedule 1.0, it was not again selected for Schedule 10 or Schedule 25.0. Similarly, if a household had not been selected for Schedule 1.0 but selected for Schedule 10, it was not selected for Schedule 25.0. However, for the households selected from SSS1 of Schedule 25.0, the Schedule 25.0 was canvassed even if the household had been selected for other schedules.

5. **Estimation Procedure**

5.1 Notations:

s = subscript for s-th stratum

m = subscript for sub-sample (m =1, 2)

i = subscript for i-th FSU [village (panchayat ward) / block]

d = subscript for a hamlet-group/sub-block (d = 1, 2)

j = subscript for j-th second stage stratum in an FSU/ hg/sb

k = subscript for k-th sample household under a particular second stage stratum within an FSU/ hg/sb

D = total number of hg's/sb's formed in the sample village (panchayat ward) / block

D* = 1 if D = 1

= D / 2 for FSUs with D > 1

N = total number of FSUs in rural stratum 1 or in any urban stratum

Z = total size of a rural stratum other than stratum 1 (= sum of sizes for all the FSUs of a rural stratum other than stratum 1)

z = size of sample village used for selection.

n = number of sample village / block surveyed including zero cases but excluding casualty for a particular sub-sample and stratum.

H = total number of households listed in a second-stage stratum of a hamlet-group/sub-block of sample FSU

h = number of households surveyed in a second-stage stratum of a hamlet-group/sub-block of sample FSU

x, y = observed value of characteristics *x*, *y* under estimation

$\hat{X}$, $\hat{Y}$ = estimate of population total X, Y for the characteristics *x*, *y*

Under the above symbols,

y_{smidjk} = observed value of the characteristic y for the k-th household in the j-th second stage stratum of the d-th hg/sb (d = 1, 2) of the i-th FSU belonging to the m-th sub-sample for the s-th stratum;

However, for ease of understanding, a few symbols have been suppressed in following paragraphs where they are obvious.

For peer review only - http://bmjopen.bmj.com/site/about/guidelines.xhtml

Appendix B

5.2 Formulae for Estimation of Aggregates for a particular Sub-sample and Stratum in Rural / Urban sector:

Schedules 25.0:

Rural:

(a) Estimation formula for stratum 1:

(i) For households selected in j-th second stage stratum:

$$\hat{Y}_j = \frac{N}{n_j}\sum_{i=1}^{n_j} D_i^* \left[\frac{H_{i1j}}{h_{i1j}} \sum_{k=1}^{h_{i1j}} y_{i1jk} + \frac{H_{i2j}}{h_{i2j}} \sum_{k=1}^{h_{i2j}} y_{i2jk} \right]$$

(ii) For all selected households:

$$\hat{Y} = \sum_j \hat{Y}_j$$

(b) Estimation formula for other strata:

(i) For households selected in j-th second stage stratum:

$$\hat{Y}_j = \frac{Z}{n_j}\sum_{i=1}^{n_j} \frac{1}{z_i} D_i^* \left[\frac{H_{i1j}}{h_{i1j}} \sum_{k=1}^{h_{i1j}} y_{i1jk} + \frac{H_{i2j}}{h_{i2j}} \sum_{k=1}^{h_{i2j}} y_{i2jk} \right]$$

(ii) For all selected households:

$$\hat{Y} = \sum_j \hat{Y}_j$$

Urban:

(a) Estimation formula for a stratum:

(i) For households selected in j-th second stage stratum:

$$\hat{Y}_j = \frac{N}{n_j}\sum_{i=1}^{n_j} D_i^* \left[\frac{H_{i1j}}{h_{i1j}} \sum_{k=1}^{h_{i1j}} y_{i1jk} + \frac{H_{i2j}}{h_{i2j}} \sum_{k=1}^{h_{i2j}} y_{i2jk} \right]$$

(ii) For all selected households:

$$\hat{Y} = \sum_j \hat{Y}_j$$

For peer review only - http://bmjopen.bmj.com/site/about/guidelines.xhtml

Note: Values of j = 1, 2, 3 or 4

5.3 Overall Estimate for Aggregates:

Overall estimate for aggregates for a stratum ($\hat{Y}_s$) based on two sub-samples is obtained as:

$$\hat{Y}_s = \frac{1}{2}\sum_{m=1}^{2}\hat{Y}_{sm}$$

5.4 Overall Estimate of Aggregates at State/UT/all-India level:

The overall estimate $\hat{Y}$ at the State/ UT/ all-India level is obtained by summing the stratum estimates $\hat{Y}_s$ over all strata belonging to the State/ UT/ all-India.

5.5 Estimates of Ratios:

Let $\hat{Y}$ and $\hat{X}$ be the overall estimate of the aggregates Y and X for two characteristics *y* and *x* respectively at the State/ UT/ all-India level.

Then the combined ratio estimate ($\hat{R}$) of the ratio $(R = \frac{Y}{X})$ will be obtained as

$$\hat{R} = \frac{\hat{Y}}{\hat{X}}$$

5.6 **Estimates of Error**:

The estimated variances of the above estimates will be as follows:

A) For aggregate $\hat{Y}$:

$$V\hat{a}r(\hat{Y}) = \sum_{s} V\hat{a}r(\hat{Y}_s)$$

where $V\hat{a}r(\hat{Y}_s)$ are as given below.

a) For strata with PPSWR selection at first stage (i.e. for all rural strata except stratum 1):

$$V\hat{a}r_{ppswr}(\hat{Y}_s) = \sum_{j} V\hat{a}r_{ppswr}(\hat{Y}_{sj})$$

where $V\hat{a}r_{ppswr}(\hat{Y}_{sj}) = \frac{1}{n_{sj}(n_{sj}-1)}\left[\sum_{i=1}^{n_{sj}} \frac{Z_s{}^2\hat{Y}_{sij}{}^2}{z_{si}{}^2} - n_{sj}\hat{Y}_{sj}{}^2\right]$,

For peer review only - http://bmjopen.bmj.com/site/about/guidelines.xhtml

Appendix B

$$\hat{Y}_{sij} = D^*_{si}\left[\frac{H_{i1j}}{h_{i1j}}\sum_{k=1}^{h_{i1j}} y_{i1jk} + \frac{H_{i2j}}{h_{i2j}}\sum_{k=1}^{h_{i2j}} y_{i2jk}\right]$$

b) For strata with SRSWOR selection at first stage (i.e. for rural stratum 1 and all urban strata):

$$Va\hat{r}_{srswor}\left(\hat{Y}_s\right) = \frac{1}{4}\left(\hat{Y}_{s1} - \hat{Y}_{s2}\right)^2 \quad ,$$

where $\hat{Y}_{s1}$ and $\hat{Y}_{s2}$ are the estimates for sub-sample 1 and sub-sample 2 respectively for stratum ‘s’.

B) For ratio $\hat{R}$:

$$M\hat{S}E(\hat{R}) = \frac{1}{(\hat{X})^2}\left[\sum_s M\hat{S}E_s(\hat{R}) + \sum_{s'} M\hat{S}E_{s'}(\hat{R})\right]$$

where s, s' indicate respectively the strata with PPSWR and SRSWOR selection at first stage.

a) For strata with PPSWR selection at first stage (i.e. for all rural strata except stratum 1):

$$M\hat{S}E_s(\hat{R}) = \frac{1}{n_s(n_s-1)}\sum_{i=1}^{n_s}\left[\frac{Z_s}{z_{si}}(\hat{Y}_{si} - \hat{R}\hat{X}_{si}) - \frac{1}{n_s}\sum_{i=1}^{n_s}\frac{Z_s}{z_{si}}(\hat{Y}_{si} - \hat{R}\hat{X}_{si})\right]^2$$

where

$$\hat{Y}_{si} = \sum_j \hat{Y}_{sij} \ , \quad \hat{X}_{si} = \sum_j \hat{X}_{sij} \ ,$$

$$\hat{Y}_{sij} = D^*_{si}\left[\frac{H_{si1j}}{h_{si1j}}\sum_{k=1}^{h_{si1j}} y_{si1jk} + \frac{H_{si2j}}{h_{si2j}}\sum_{k=1}^{h_{si2j}} y_{si2jk}\right],$$

$$\hat{X}_{sij} = D^*_{si}\left[\frac{H_{si1j}}{h_{si1j}}\sum_{k=1}^{h_{si1j}} x_{si1jk} + \frac{H_{si2j}}{h_{si2j}}\sum_{k=1}^{h_{si2j}} x_{si2jk}\right]$$

b) For strata with SRSWOR selection at first stage (i.e. for rural stratum 1 and all urban strata):

For peer review only - http://bmjopen.bmj.com/site/about/guidelines.xhtml

$$M\hat{S}E_{s'}(\hat{R}) = \frac{1}{4}\left[\left(\hat{Y}_{s'1} - \hat{Y}_{s'2}\right)^2 + \hat{R}^2\left(\hat{X}_{s'1} - \hat{X}_{s'2}\right)^2 - 2\hat{R}\left(\hat{Y}_{s'1} - \hat{Y}_{s'2}\right)\left(\hat{X}_{s'1} - \hat{X}_{s'2}\right)\right]$$

C) Estimates of RSE:

$$R\hat{S}E\left(\hat{Y}\right) = \frac{\sqrt{V\hat{a}r\left(\hat{Y}\right)}}{\hat{Y}} \times 100$$

$$R\hat{S}E\left(\hat{R}\right) = \frac{\sqrt{M\hat{S}E\left(\hat{R}\right)}}{\hat{R}} \times 100$$

6.0 Multipliers:

The formulae for multipliers for a sub-sample and schedule 25.0 are given below:

stratum	formula for multipliers	
	hg / sb 1	hg / sb 2
rural stratum 1 and all urban strata	$\frac{N_s}{n_{smj}} \times D^*_{smi} \times \frac{H_{smi1j}}{h_{smi1j}}$, j = 1, 2, 3, 4	$\frac{N_s}{n_{smj}} \times D^*_{smi} \times \frac{H_{smi2j}}{h_{smi2j}}$, j = 1, 2, 3, 4
rural s ≠ 1	$\frac{Z_s}{n_{smj}} \times \frac{1}{z_{smi}} \times D^*_{smi} \times \frac{H_{smi1j}}{h_{smi1j}}$, j = 1, 2, 3, 4	$\frac{Z_s}{n_{smj}} \times \frac{1}{z_{smi}} \times D^*_{smi} \times \frac{H_{smi2j}}{h_{smi2j}}$, j = 1, 2, 3, 4

Note: (i) For estimating any characteristic for any domain not specifically considered in sample design, indicator variable may be used.

(ii) Multipliers have to be computed on the basis of information available in the listing schedule irrespective of any misclassification observed between the listing schedule and detailed enquiry schedule.

(iii) For estimating number of villages possessing a characteristics, D^*_{smi} = 1 in the relevant multipliers and there will be only one multiplier for the village.

7.0 Treatment for zero cases, casualty cases etc.:

7.1 While counting the number of FSUs surveyed (n_{sm}) in a stratum, all the FSUs with survey codes 1 to 6 in schedule 0.0 will be considered. In addition, for a particular schedule if no USU is available in the frame then also that FSU will be treated as surveyed in respect of that schedule. However, if the USUs of a particular schedule type are available in the frame of the FSU but none of these could be surveyed then that FSU has to be treated as casualty and it will not be treated as surveyed in respect of that schedule.

For peer review only - http://bmjopen.bmj.com/site/about/guidelines.xhtml

7.2 *Casualty cases*: FSUs with survey code 7 as per schedule 0.0 are treated as casualties. In addition to this, an FSU, although surveyed, may have to be treated as casualty for a particular schedule type and a particular *second stage stratum* as given in the following para:

7.2.1 FSUs with survey codes 1 and 4 as per schedule 0.0 having number of households in the frame of j-th second stage stratum greater than 0 but number of households surveyed according to data file, considering both hg/sb together, as nil (i.e. $H_{i1j} + H_{i2j} > 0$ but $h_{i1j} + h_{i2j} = 0$) will be taken as casualties for j-th second stage stratum.

All the FSUs with survey codes 1 to 6 as per schedule 0.0 minus the number of casualties as identified above will be taken as the number of surveyed FSUs (n_{mj}) for that stratum × second stage stratum.

When casualty for j-th second stage stratum occurs for a particular hg/sb but not for the other hg/sb, the FSU will not be treated as casualty but some adjustments in the value of H for the other hg/sb will be done as follows:

(i) Suppose for hg/sb 1, $H_{i1j} > 0$ but $h_{i1j} = 0$ while for hg/sb 2, $H_{i2j} > 0$ and $h_{i2j} > 0$. In that case $D_i^* \times H_{i2j}$ will be replaced by $D_i^* \times (H_{i1j} + H_{i2j})$ in the formula for multiplier of segment 2.

(ii) Suppose for hg/sb 1, $H_{i1j} > 0$ and $h_{i1j} > 0$ while for hg/sb 2, $H_{i2j} > 0$ but $h_{i2j} = 0$. In that case $D_i^* \times H_{i1j}$ will be replaced by $D_i^* \times (H_{i1j} + H_{i2j})$ in the formula for multiplier of hg/sb 1.

It may be noted that n_{smj} would be same for hg/sb 1 & 2 of an FSU.

8.0 Treatment in cases of void second-stage strata/sub-strata /strata/NSS region at FSU or household level

8.1 A stratum may be void because of the casualty of all the FSUs belonging to the stratum. This may occur in one sub-sample or in both the sub-samples. If it relates to only one sub-sample, then estimate for the void stratum may be replaced with the estimate as obtained from the other sub-sample for the same stratum.

8.2 When a stratum is void in both the sub-samples, the following procedure is recommended:

Case(I): Stratum void cases at FSU levels (i.e. all FSUs having survey code 7):

i) If a rural stratum, except stratum 1, is void then it may be merged with a similar stratum within the same NSS region. However, if rural stratum 1 is void for any State/UT, it will not be merged with other stratum and remarks to that effect may be added in appropriate places.

ii) If an urban stratum is void then it may be merged with another stratum within the same NSS region

iii) If all the strata within an NSS region is void, it may be excluded from the coverage of the survey. The state level estimates will be based on the estimates of NSS regions for which estimates are available and remarks to that effect may be added in appropriate places.

For peer review only - http://bmjopen.bmj.com/site/about/guidelines.xhtml

Case (II): Stratum void case at second stage stratum level (i.e. all the FSUs are casualties for a particular second stage stratum):

An FSU may be a casualty for a particular *second stage stratum* although survey code is not 7. If all the FSUs of a stratum become casualties in this manner for a particular *second stage stratum*, the stratum will become void. The adjustment for this type of stratum void case may be done according to the following guidelines.

The adjustment will be made involving other strata (within NSS region) of the State/U.T. Suppose A, B, C, and D are the four strata in the State/UT/Region and stratum C is void for j-th *second stage stratum*. If $\hat{Y}_{aj}$, $\hat{Y}_{bj}$ and $\hat{Y}_{dj}$ are the aggregate estimates for the strata A, B and D respectively, then the estimate $\hat{Y}_{cj}$ for stratum C may be obtained as $\left(\frac{\hat{Y}_{aj}+\hat{Y}_{bj}+\hat{Y}_{dj}}{Z_a+Z_b+Z_d}\times Z_c\right)$ where Z_a, Z_b, Z_c and Z_d are the sizes of strata A, B, C and D respectively.

******

For peer review only - http://bmjopen.bmj.com/site/about/guidelines.xhtml

Appendix C

RURAL		*
URBAN		

GOVERNMENT OF INDIA
NATIONAL SAMPLE SURVEY ORGANISATION
SOCIO-ECONOMIC SURVEY
SIXTIETH ROUND: JANUARY – JUNE, 2004

CENTRAL		*
STATE		

SCHEDULE 25.0: MORBIDITY AND HEALTH CARE

**[0] descriptive identification of sample household**	
1. state/u.t.:	5. hamlet name:
2. district:	6. ward/inv. unit/block:
3. tehsil/town:	7. name of head of household:
4. village name:	8. name of informant:

[1] identification of sample household

item no.	item	code	item no.	item	code
1.	srl. no. of sample village/ block		11.	sub-sample	
2.	round number	6 0	12.	FOD sub-region	
3.	schedule number	2 5 0	13.	sample hamlet-group/sub-block number	
4.	schedule type		14.	second stage stratum	
5.	sample (central-1, state-2)		15.	sample household number	
6.	sector (rural-1, urban-2)		16.	srl. no. of informant (as in col.1, block 4)	
7.	state-region		17.	response code	
8.	district		18.	survey code	
9.	stratum number		19.	reason for substitution of original household (code)	
10.	sub-round				

CODES FOR BLOCK 1

*item 17: **response code**: informant: co-operative and capable -1, co-operative but not capable - 2, busy -3, reluctant - 4, others - 9*

*item 18: **survey code**: original – 1, substitute – 2, casualty – 3*

*item 19: **reason for substitution of original household**: informant busy - 1, members away from home - 2, informant non-cooperative -3, others - 9*

** tick mark (✓) may be put in the appropriate place.*

For peer review only

For peer review only - http://bmjopen.bmj.com/site/about/guidelines.xhtml

Appendix C

[2] particulars of field operation										
srl.no.	item	investigator			assistant superintendent			superintendent		
(1)	(2)	(3)			(4)			(5)		
1.	(i) name (block letters)									
	(ii) code									
2.	**date(s) of**:	DD	MM	YY	DD	MM	YY	DD	MM	YY
	(i) survey/ inspection									
	(ii) receipt									
	(iii) scrutiny									
	(iv) despatch									
3.	number of additional sheets attached									
4.	total time taken to canvass Sch. 25.0 (in minutes)									
5.	signature									

**[12] remarks by investigator**

**[13] comments by supervisory officer(s)**

For peer review only

For peer review only - http://bmjopen.bmj.com/site/about/guidelines.xhtml

Appendix C

[3] household characteristics				
1. household size			11. major source of drinking water (code)	
2. principal industry (NIC-1998)	description:		12. is water treated before drinking? (yes - 1, no -2)	
	code (5-digit)		13. if 1 in item 12, type of water treatment (code)	
3. principal occupation (NCO-1968)	description:		14. primary source of energy for cooking (code)	
	code (3-digit)		15. is there any pet animal? (yes-1, no-2)	
4. household type (code)			**household consumer expenditure (Rs) during last 30 days out of:**	
5. religion (code)				
6. social group (code)			16. purchase	
7. land possessed as on the date of survey (code)			17. home-produced stock	
			18. receipts in exchange of goods and services	
8. type of structure (code)			19. gifts and loans	
9. type of latrine (code)			20. free collection	
10. type of drainage (code)			21. total (items 16 to 20)	

CODES FOR BLOCK 3

*item 4 - **household type**: **for rural areas**: self-employed in non-agriculture -1, agricultural labour-2, other labour -3, self-employed in agriculture - 4, others- 9*

***for urban areas**: self-employed -1, regular wage/salary earnings - 2, casual labour -3, others - 9*

*item 5 – **religion**: Hinduism -1, Islam -2, Christianity -3, Sikhism -4, Jainism -5, Buddhism -6, Zoroastrianism -7, others -9*

*item 6 - **social group**: scheduled tribe-1, scheduled caste -2, other backward class -3, others- 9*

*item 7 – **land possessed (class interval in hectares)**: less than 0.005 -01, 0.005 to 0.01 -02, 0.02 to 0.20 -03, 0.21 to 0.40 -04,0.41 to 1.00 -05, 1.01 to 2.00 -06, 2.01 to 3.00 -07, 3.01 to 4.00 -08, 4.01 to 6.00 -10, 6.01 to 8.00 -11, greater than 8.00 -12*

*item 8 - **type of structure**: structure: pucca - 1, semi-pucca -2, serviceable kutcha -3, unserviceable kutcha – 4; no structure - 5*

*item 9 – **type of latrine**: latrine: service - 1, pit - 2, septic tank/ flush system - 3, others - 9; no latrine - 4*

*item 10 – **type of drainage**; drainage: open kutcha - 1, open pucca - 2, covered pucca - 3, under ground – 4; no drainage - 5*

*item 11 - **major source of drinking water**: bottled water – 1, tap – 2, tube-well/hand pump-3, tankers - 4, pucca well – 5, tank/pond reserved for drinking – 6, river/canal - 7, others – 9*

*item 13 – **type of water treatment**: ultra-violet/resin/reverse osmosis – 1, boiling – 2, filter – 3, cloth screen – 4, any disinfectant – 5, others – 9*

*item 14 –**primary source of energy for cooking**: coke, coal – 01, firewood and chips – 02, LPG – 03, gobar gas – 04, dung cake – 05, charcoal – 06, kerosene – 07, electricity – 08, others – 99; no cooking arrangement – 10*

[5] particulars of household members who died during last 365 days

srl. no.	name of deceased member	sex (male -1, female-2)	age at death (years)	medical attention received before death (yes-1, no-2)	whether hospita-lised (yes-1, no-2)	if 1 in col. 6, no. of times hospita-lised	if 2 in col. 3,		annual amount paid (Rs) for	
							whether pregnant (yes-1, no-2)	if 1 in col. 8, time of death (code)	medical insurance premium	health schemes (CGHS, ESI, etc.)
(1)	(2)	(3)	(4)	(5)	(6)	(7)	(8)	(9)	(10)	(11)
91										
92										

CODES FOR BLOCK 5

*col. 9 - **time of death**: for deaths relating to pregnancy/delivery/abortion: during pregnancy – 1, during delivery – 2, during abortion – 3; within 6 weeks of delivery/abortion – 4; other deaths - 9*

1 Acre = 0.4047 Hectare; 1 Hectare = 10,000 sq metre

For peer review only - http://bmjopen.bmj.com/site/about/guidelines.xhtml

Appendix C

[4] demographic particulars of household members

srl. no.	name of member	relation to head (code)	sex (male -1, female -2)	age (years)	marital status (code)	general educational level (code)	usual activity status (code)	during last 365 days		whether ailing		report-ting of columns 11 & 12 (self - 1, proxy – 2)	annual amount paid (Rs) for	
								whether hospita-lised (yes-1, no-2)	if 1 in col. 9, no. of times hospit-alised	anytime during last 15 days (yes-1, no-2)	on the day before the date of survey (yes -1, no -2)		medical insurance premium	health schemes (CGHS, ESI, etc.)
(1)	(2)	(3)	(4)	(5)	(6)	(7)	(8)	(9)	(10)	(11)	(12)	(13)	(14)	(15)

CODES FOR BLOCK 4

*col. 3 - **relation to head**: self - 1, spouse of head - 2, married child - 3, spouse of married child - 4, unmarried chiId - 5, grand child - 6, father/mother/father-in-law/mother-in-law - 7, brother/sister/brother-in-law/sister-in-law/other relatives - 8, servant/employees/other non-relatives - 9*

*col. 6 - **marital status**: never married - 1, currently married - 2, widowed - 3, divorced/separated – 4*

*co1.7 - **general educational level**: not literate - 01; literate: without formal schooling - 02, below primary - 03, primary - 04, middle - 05, secondary - 06, higher secondary - 07, diploma/certificate course – 08, graduate – 10, post-graduate & above - 11*

*col. 8 - **usual activity status**: worked in hh enterprise (self-employed): own account worker – 11, employer – 12, worked as helper in hh enterprise (unpaid family worker) – 21; worked as regular salaried/wage employee – 31; worked as casual wage labour: in public works – 41, in other types of work – 51; did not work but was seeking and/or available for work – 81, attended educational institution – 91, attended domestic duties only – 92, attended domestic duties and was also engaged in free collection of goods (vegetables, roots, firewood, cattle feed, etc.), sewing, tailoring, weaving, etc. for household use – 93, rentiers, pensioners, remittance recipients, etc. – 94, not able to work due to disability – 95, beggars, prostitutes – 96, others – 97*

For peer review only
For peer review only - http://bmjopen.bmj.com/site/about/guidelines.xhtml

Appendix C

[6] particulars of economic independence and ailments on the date of survey for persons aged 60 years and above				
1. srl. no. of member (as in col. 1, block 4)				
2. age (years) (as in col. 5, block 4)				
3. number of sons living				
4. number of daughters living				
5. state of economic independence (code)				
6. for 1 in item 5, no. of dependants				
7. for 2 or 3 in item 5, person financially supporting aged person (code)				
8. for 1 in item 5, amount of loan(s) outstanding (Rs)				
9. living arrangement (code)				
10. if 1, 2 or 3 in item 9, whether child/ grandchild/sibling staying nearby (code)				
11. physical mobility (code)				
12. if 1 or 2 in item 11, person helping (household member - 1, other than household member - 2, none - 3)				
**ailments on the date of enquiry (code)**				
13. ailment 1 (code)				
14. ailment 2 (code)				
15. ailment 3 (code)				
16. ailment 4 (code)				
17. ailment 5 (code)				
**whether any treatment taken for the ailments reported in items 13to 17 (yes-1, no-2)**				
18. ailment 1				
19. ailment 2				
20. ailment 3				
21. ailment 4				
22. ailment 5				
23. own perception about current state of health (code)				
24. own perception about relative state of health (code)				

CODES FOR BLOCK 6

*item 5 - **state of economic independence**: not dependent on others - 1, partially dependent on others - 2, fully dependent on others - 3*

*item 7 - **person supporting aged person**: spouse - 1, own children - 2, grand children - 3, others - 9*

*item 9 - **living arrangement**: living alone:as an inmate of old age home - 1, not as an inmate of old age home - 2; living with spouse only - 3, with spouse and other members - 4, without spouse but with: children - 5,other relations - 6, non-relations - 9*

*item 10 - **whether child/grandchild/sibling staying nearby**: yes: within the same building -1, within the village/town - 2, outside the village/town - 3; not applicable - 4*

*item 11 - **physical mobility**: physically immobile: confined to bed - 1, confined to home - 2; physically mobile – 3*

*items 13 to 17- **ailment**: code list is given on page C-10*

*item 23 - **own perception about current state of health**: excellent/very good - 1, good/fair - 2, poor - 3*

*item 24 - **own perception about relative state of health:** compared to previous year: much better - 1, somewhat better - 2, nearly the same - 3, somewhat worse - 4, worse – 5*

Report No. 507: Morbidity, Health Care and the Condition of the Aged, Jan.-June,2004

For peer review only

Appendix C

[7] particulars of medical treatment received as inpatient of a hospital during the last 365 days						
1. sr1. no. of the hospitalisation case		1	2	3	4	5
2. srl. no. of member (as in col. 1, block 4/5) hospitalised						
3. age (years) (as in col. 5, block 4 / col. 4, block 5)						
4. type of hospital (code)						
5. nature of ailment (code)						
6. type of ward (free - 1, paying general - 2, paying special - 3)						
7. when admitted (code)						
8. when discharged (code)						
9. duration of stay in hospital (days)						
10. loss of household income, if any, due to hospitalisation (Rs)						
**details of medical services received** (not received - 1; received: free - 2, partly free - 3, on payment - 4)						
	11. surgery					
	12. medicine					
	13. X-ray/ECG/EEG/Scan					
	14. other diagnostic tests					
15. whether treatment availed before hospitalisation (yes - 1, no - 2)						
**if 1 in item 15**	16. source of treatment (code)					
	17. duration of treatment (days)					
18. whether treatment continued after discharge from hospital (yes - 1, no - 2)						
**if 1 in item 18**	19. source of treatment (code)					
	20. duration of treatment (days)					

CODES FOR BLOCK 7

*item 4 - **type of hospital:** public hospital (incl. PHC/ sub-centres/CHC) - 1, public dispensary (incl. CGHS/ESI) – 2, private hospital – 3*

*item 5 - **nature of ailment**: code list is given on page C-10*

*item 7 - **when admitted** : during last 15 days - 1, 16 days to 365 days ago - 2, more than 365 days ago – 3*

*item 8 - **when discharged**: not yet - 1, during last 15 days - 2, 16 days to 365 days ago – 3*

*items 16 & 19 - **source of treatment:** public hospital (incl. PHC/ sub-centres/CHC) - 1, public dispensary (incl. CGHS/ESI)– 2, private hospital – 3, private doctor – 4*

For peer review only - http://bmjopen.bmj.com/site/about/guidelines.xhtml

Appendix C

[8] expenses incurred for treatment of members treated as inpatient of hospital during the last 365 days and source of finance		1	2	3	4	5
1. srl. no. of the hospitalisation case (as in item 1, block 7)		1	2	3	4	5
2. srl. no. of member hospitalised (as in item 2, block 7)						
3. age (years) (as in item 3, block 7)						
4. whether any medical service provided free by employer (yes: Govt. - 1, pvt . - 2; no – 3, not applicable – 4)						
**medical expenditure for treatment during stay at hospital (Rs)**						
doctor's / surgeon's fee	5. hospital staff					
	6. other specialists					
medicines	7. from hospital					
	8. from outside					
9. diagnostic tests						
10. bed charges						
11. attendant charges						
12. physiotherapy						
13. personal medical appliances						
others	14. food and other materials					
	15. blood, oxygen cylinder, etc.					
	16. services (ambulance, etc.)					
17. expenditure not elsewhere reported						
18. total (items 5 to 17)						
**other expenses incurred by the household (Rs) (not included in item 18)**						
19. transport (other than ambulance)						
20. lodging charges of escort(s)						
21. others						
22. total (items 19 to 21)						
23. total expenditure incurred by the household (sum of items 18 & 22 for all cases of hospitalisation taken together)						
**expenses in item 23 by source of finance (Rs)**						
24. household income/savings						
25. borrowings						
26. contributions from friends and relatives						
27. other sources (incl. sale of ornaments and other physical assets, draught animals, etc.)						
28. total (items 24 to 27)						
29. amount of reimbursement (Rs)						
**if positive entry in item 29, amount reimbursed by (Rs)** — employer	30. Government					
	31. private					
	32. medical insurance companies					
	33. other agencies					

For peer review only

For peer review only - http://bmjopen.bmj.com/site/about/guidelines.xhtml

Appendix C

[9] particulars of spells of ailment of household members during the last 15 days (including hospitalisation)						
1. srl. no. of spell of ailment		1	2	3	4	5
2. srl. no. of member reporting ailment (as in col. 1 of block 4/5)						
3. age (years) (as in col. 5, block 4 / col. 4, block 5)						
**number of days within the reference period**	4. ill					
	5. on restricted activity					
	6. confined to bed					
7. nature of ailment (code)						
8. status of ailment (code)						
9. total duration of ailment (days)						
10. whether treatment taken on medical advice (yes - 1, no - 2)						
**if 1 in item 10**	11. whether any treatment received from govt. sources (yes - 1, no - 2)					
	12. **if 2 in item 11**, reason (code)					
**if 2 in item 10**	13. reason for no treatment (code)					
	14. whether any other measure taken for recovery/relief (yes - 1, no - 2)					
	**if 1 in item 14** — 15. whom consulted (code)					
	**if 1 in item 14** — 16. expenditure incurred (Rs)					
17. loss of household income, if any, due to ailment (Rs)						

CODES FOR BLOCK 9

*item 7 - **nature of ailment:** code list is given on page C-10*

*item 8 - **status of ailment**: started more than 15 days ago and is continuing -1, started more than 15 days ago and has ended -2, started within 15 days and is continuing -3, started within 15 days and has ended - 4*

*item 12 - **reason for not availing govt. sources**: Govt. doctor/facility too far-1, not satisfied with medical treatment by Govt. doctor/facility -2, long waiting -3,required specific services not available - 4, others - 9*

*item 13 - **reason for no treatment**: no medical facility available in the neighbourhood - 1, facilities available but no treatment sought owing to: lack of faith - 2, long waiting - 3, financial reasons - 4, ailment not considered serious - 5, others - 9*

*item 15 - **whom consulted**: self/other household member/ friend - 1, medicine shop - 2, others - 9*

For peer review only - http://bmjopen.bmj.com/site/about/guidelines.xhtml

Appendix C

[10] expenses incurred during the last 15 days for treatment of members (not as inpatient of hospital) and source of finance							
1. srl. no. of ailing member (as in item 2, block 9)							
2. age (years) (as in item 3, block 9)							
3. whether any medical service provided free by employer (yes: Govt. -1, pvt. - 2; no - 3, not applicable - 4)							
**details of medical services received** (not received - 1; received: free - 2, partly free - 3, on payment - 4)							
	4. surgery						
	5. medicine received						
	6. X-ray/ECG/EEG/Scan						
	7. other diagnostic tests						
**medical expenditure for treatment (Rs)**							
doctor's / surgeon's fee	8. hospital staff						
	9. other specialists						
medicines	10. from hospital						
	11. from outside						
	12. diagnostic tests						
	13. attendant charges						
	14. physiotherapy						
	15. personal medical appliances						
others	16. food and other materials						
	17. blood, oxygen cylinder, etc.						
	18. services (ambulance, etc.)						
	19. expenditure not elsewhere reported						
	20. total medical expenditure (items 8 to 19)						
**expenditure reported in item 20 from**	21. Govt. sources						
	22. other sources						
**other expenses incurred by the household (Rs) (not included in item 20)**							
	23. transport charges (other than ambulance)						
	24. lodging charges of ailing person and escort(s)						
	25. others						
	26. total (items 23 to 25)						
27. total expenditure incurred by the household (sum of items 20 & 26 for all persons taken together)							
**source of finance for meeting the expenses in item 27 (Rs)**							
	28. household income/savings						
	29. borrowings						
	30. contributions from friends and relatives						
	31. other sources (incl. sale of ornaments and other physical assets, draught animals, etc.)						
	32. total (items 28 to 31)						
33. total amount of reimbursement (Rs)							
**if positive entry in item 33, amount reimbursed by (Rs)**	employer	34. Government					
		35. private					
	36. medical insurance companies						
	37. other agencies						

For peer review only - http://bmjopen.bmj.com/site/about/guidelines.xhtml

Appendix C

[11] particulars of immunisation of children (0 – 4 yrs.), pre-natal care and post-natal care for ever married women of age below 50 years during the last 365 days

srl. no. (as in block 4/5)	age (years) (as in block 4/5)	for children of age 0-4 yrs.		for ever married women below 50 years, during the last 365 days						
		whether any immu-nisation received (yes - 1 no - 2)	if 1 in col. 3, expenditure incurred (Rs)	whether pregnant any time (yes - 1, no - 2)	if 1 in col. 5					
					whether any pre-natal care received (code)	if 1 or 2 in col. 6, expenditure incurred (Rs)	whether given birth to a child (yes: in govt. hospital-1, in private hospital - 2; at home - 3; no - 4)	if 1, 2 or 3 in col. 8, expenditure on childbirth (Rs)	if 1, 2 or 3 in col. 8, whether any post-natal care received (code)	if 1 or 2 in col. 10, expenditure incurred (Rs)
(1)	(2)	(3)	(4)	(5)	(6)	(7)	(8)	(9)	(10)	(11)

CODES FOR BLOCK 11

cols. 6 & 10 - ***pre-natal/post-natal care received****: yes: from Govt. sources – 1, from private sources – 2; no - 3*

CODES FOR: (i) AILMENT (items 13 to 17, block 6)

(ii) NATURE OF AILMENT (item 5, block 7 & item 7, block 9)

ailment	code	ailment	code
**Gastro-intestinal**		*Diabetes mellitus*	22
Diarrhoea/ dysentery	01	*Under-nutrition*	23
Gastritis/gastric or peptic ulcer	02	*Anaemia*	24
Worm infestation	03	*Sexually transmitted diseases*	25
Amoebiosis	04	**Febrile illnesses**	
Hepatitis/Jaundice	05	*Malaria*	26
**Cardiovascular Diseases**		*Eruptive*	27
Heart disease	06	*Mumps*	28
Hypertension	07	*Diphtheria*	29
		Whooping cough	30
Respiratory including ear/nose/throat ailments	08	*Fever of unknown origin*	31
Tuberculosis	09		
Bronchial asthma	10	*Tetanus*	32
Disorders of joints and bones	11	*Filariasis/Elephantiasis*	33
Diseases of kidney/urinary system	12		
Prostatic disorders	13	**Disabilities**	
Gynaecological disorders	14	*Locomotor*	34
Neurological disorders	15	*Visual including blindness (excluding cataract)*	35
Psychiatric disorders	16	*Speech*	36
		Hearing	37
**Eye ailments**		*Diseases of Mouth/Teeth/Gum*	38
Conjunctivitis	17	*Accidents/Injuries/Burns/ Fractures/Poisoning*	39
Glaucoma	18	*Cancer and other tumours*	40
Cataract	19		
Diseases of skin	20	*Other diagnosed ailments*	41
Goitre	21	*Other undiagnosed ailments*	99

For peer review only - http://bmjopen.bmj.com/site/about/guidelines.xhtml

List of NSS Reports available for sale

Sl. No.	Report No.	Title of the Report	Price (in Rs.) Hard Copy	Price (in Rs.) Floppy(s)/ CD(s)
(1)	(2)	(3)	(4)	(5)
		**Unorganised Trade, NSS 46th Round**		
1*	403	Small Trading Units in India	150	380
2	403/1	State Level results on small trading units in India: Vol.-I	250	710
3*	403/1	State Level results on small trading units in India: Vol.-II	250	710
		**Land & livestock holdings and Debt & Investment, NSS 48th Round**		
4*	407	Operational land holdings in India, 1991-92: Salient features	250	710
5	408	Live-stock and Agricultural implements in House-hold operational holdings, 1991-92	150	380
6	414	Seasonal variation in the operation of land holdings in India, 1991-92	250	710
7	419	Household Assets and Liabilities as on 30.6.91	250	1140
8	420	Indebtedness of Rural Households as on 30.6.1991	250	1370
9	421	Indebtedness of Urban Households as on 30.6.1991	250	1370
10	431(Part I)	Household Borrowings and Repayments during 1.7.91 to 30.6.92	250	1140
11	431(Part-II)	-do-	250	1140
12	432 (Part-I)	Households Assets and Indebtedness of Social Groups as on 30.6.91	250	1140
13	432 (Part-II)	-do-	250	710
14	437	Household capital expenditure during 1.7.91 to 30.6.92.	250	1370
		**Housing Conditions and Migration with special emphasis on slum dwellers, NSS 49th round**		
15	417	Slums in India	150	380
16	429	Housing Conditions in India	150	380
17	430	Migration in India	250	710
		**Employment & Unemployment, NSS 50th Round**		
18	406	Key Results on Employment & Unemployment	150	610
19	409	Employment & Unemployment in India, 1993-94	250	710
20	411	Employment & Unemployment situation in cities and Towns in India, 1993-94	150	380
21	412	Economic activities and school attendance by children in India, 1993-94	150	380
22*	416	Participation of Indian women in household work and other specified activities, 1993-94	150	380
23	418	Unemployed in India, 1993-94 : Salient Features	150	380
24	425	Employment & Unemployment situation among social groups in India, 1993-94	250	480
25	438	Employment & Unemployment situation among religious groups in India, 1993-94	150	610
		**Consumer Expenditure, NSS 50th Round**		
26	401	Key results on Household Consumer Expenditure, 1993-94	150	380
27	402	Level and Pattern of Consumer Expenditure	250	710
28*	404	Consumption of some important commodities in India	250	710
29	405	Nutritional intake in India	250	710
30	410/1	Dwellings in India	250	710
31	410/2	Energy used by Indian households	150	380
32	413	Sources of household income in India, 1993-94	150	380
33	415	Reported adequacy of food intake in India, 1993-94	150	380
34	422	Differences in level of consumption among socio-economic groups	150	380
35	423	IRDP assistance and participation in Public Works, 1993-94	150	380
36	426	Use of durable goods by Indian households, 1993-94	150	380
37	424	Ownership of Live-Stock, cultivation of selected crops and consumption levels, 1993-94	150	610
38	427	Consumption of tobacco in India, 1993-94	150	610
39	428	Wages in kind, Exchanges of Gifts and Expenditure on Ceremonies and Insurance in India, 1993-94	150	610

*** The hard copies are out of stock. However, soft copies are available.**

Continued

For peer review only

List of NSS Reports available for sale(contd.)

Sl. No.	Report No.	Title of the Report	Price (in Rs.) Hard Copy	Floppy(s)/ CD(s)
(1)	(2)	(3)	(4)	(5)
		**Consumer Expenditure and Unorganised Manufacture, NSS 51st Round**		
40	433	Unorganised Manufacturing Sector in India Its Size, Employment and Some Key Estimates.	250	710
41	434	Unorganised Manufacturing Enterprises in India: Salient Features	250	710
42	435	Assets and Borrowings of the Unorganised Manufacturing Enterprises in India	150	380
43	436	Household Consumer Expenditure and Employment Situation in India, 1994-95	150	610
		**Consumer Expenditure, NSS 52nd Round**		
44	440	Household Consumer Expenditure and Employment Situation in India, 1995-96	150	610
		**Education, NSS 52nd Round**		
45	439	Attending an Educational Institution in India: Its level, nature and cost	250	1140
		**Health, NSS 52nd Round**		
46*	441	Morbidity and Treatment of ailments	250	1140
47	445	Maternal and Child Health Care in India	150	1270
		**Aged in India, NSS 52nd Round**		
48	446	The Aged in India, A Socio-Economic Profile, 1995-96	150	380
		**Consumer Expenditure, NSS 53rd Round**		
49	442	Household Consumer Expenditure and Employment Situation in India, 1997	150	610
		**Unorganised Trade, NSS 53rd Round**		
50	443	Small Trading units in India and their Basic Characteristics: 1997, Vol. I	250	710
51	444	Small Trading units in India and their Basic Characteristics: 1997, Vol. II	250	710
		**Consumer Expenditure, Common Property Resources, Sanitation & Hygiene, Services, NSS 54th Round**		
52	448	Household Consumer Expenditure and Employment Situation in India	150	610
53	449	Drinking water, sanitation and hygiene in India	250	1140
54	450	Travel and Use of Mass Media and Financial Services by Indian Households	150	610
55	451	Cultivation Practices in India	250	1370
56	452	Common Property Resources	250	1370
		**Choice of Reference Period for Consumption Data, NSS 51st, 52nd, 53rd & 54th Round**		
57	447	Choice of Reference Period for Consumption Data	150	1700
		**Consumer Expenditure, NSS 55th Round (July'99 - June 2000)**		
58	453	Household Consumer Expenditure in India (July – December 1999) - Key Results	150	610
59	454	Household Consumer Expenditure in India, 1999–2000 - Key Results	150	610
60*	457	Level and Pattern of Consumer Expenditure in India, 1999 - 2000	250	1520
61	461	Consumption of some important Commodities in India, 1999-2000	250	1370
62	463	Sources of household income in India, 1999-2000	150	380
63	464	Energy Used by Indian Households, 1999-2000	150	610
64	466	Reported Adequacy of Food Intake in India, 1999 - 2000	150	610
65	467	IRDP Assistance and Participation in Public Works: 1999-2000	150	610
66	471	Nutritional Intake in India, 1999-2000	250	710
67	472	Differences in the level of consumption among socio economic groups, 1999-2000	250	480
68	473	Literacy and Levels of Education in India, 1999 - 2000	250	610
69	474	Sources of household consumption in India, 1999 - 2000	250	710

* **The hard copies are out of stock. However, soft copies are available.**

Continued

For peer review only

List of NSS Reports available for sale (contd.)

Sl. No.	Report No.	Title of the Report	Price (in Rs.)	
			Hard Copy	Floppy(s)/ CD(s)
(1)	(2)	(3)	(4)	(5)
		**Employment & Unemployment, NSS 55th Round (July'99 - June 2000)**		
70	455	Employment and Unemployment in India, 1999-2000 - Key Results	150	610
71	458 (Part-I)	Employment and Unemployment Situation in India, 1999 - 2000	250	750
72	458(Part-II)	Employment and Unemployment Situation in India, 1999 - 2000	250	1370
73	460	Non agricultural workers in Informal Sector based on Employment and Unemployment Survey, 1999-2000	150	610
74	462	Employment and Unemployment situation in Cities and Towns of India, 1999-2000	150	610
75	465	Participation of Indian Women in Household work and other specified activities, 1999-2000	150	610
76	468	Employment and Unemployment among religious groups in India, 1999-2000	150	610
77	469	Employment and Unemployment among social groups in India, 1999-2000	250	2950
78	470	Migration in India, 1999-2000	250	1140
		**Non-agricultural Enterprises in Informal Sector 1999-2000, NSS 55th Round (July'99 - June 2000)**		
79*	456	Non-agricultural Enterprises in the Informal Sector in India, 1999-2000 - Key Results	150	610
80	459	Informal Sector in India, 1999 - 2000 - Salient Features	250	1600
		**Unorganised Manufacturing, NSS 56th Round (July 2000 - June 2001)**		
81	477	Unorganised Manufacturing Sector in India 2000-2001 - Key Results	250	710
82	478	Unorganised Manufacturing Sector in India 2000-2001 - Characteristics of Enterprises	250	1370
83	479	Unorganised Manufacturing Sector in India, 2000 – 2001: Employment, Assets and Borrowings	250	1370
84	480	Unorganised Manufacturing Sector in India, 2000 – 2001: Input, Output and Value added	250	1370
		**Consumer Expenditure, NSS 56th Round (July 2000 - June 2001)**		
85	476	Household Consumer Expenditure and Employment - Unemployment Situation in India, 2000 - 2001	150	1040
		**Pilot Survey on Suitability of Reference Period for Measuring Household Consumption**		
86	475	Results of a Pilot Survey on Suitability of Different Reference Periods for Measuring Household Consumption	150	610
		**Unorganised Service Sector, NSS 57th Round (July 2001 - June 2002)**		
87	482	Unorganised Service Sector in India 2001 - 02 Salient Features	250	1925
88	483	Unorganised Service Sector in India 2001 - 02 Characteristics of Enterprises	250	1370
		**Consumer Expenditure, NSS 57th Round (July 2001 - June 2002)**		
89	481	Household Consumer Expenditure and Employment - Unemployment Situation in India, 2001 - 2002	250	2680
		**Disability, NSS 58th Round (July 2002 - December 2002)**		
90	485	Disabled Persons in India, July-December 2002	250	7080
		**Consumer Expenditure, NSS 58th Round (July 2002 - December 2002)**		
91	484	Household Consumer Expenditure and Employment - Unemployment Situation in India, 2002 - 2003	150	2380

* **The hard copies are out of stock. However, soft copies are available.**

Continued

For peer review only

List of NSS Reports available for sale

Sl. No.	Report No.	Title of the Report	Price (in Rs.) Hard Copy	Price (in Rs.) Floppy(s)/ CD(s)
(1)	(2)	(3)	(4)	(5)
		**Village facilities, NSS 58th Round (July 2002 - December 2002)**		
92	487	Report on village facilities, July-December 2002	150	980
		**Urban Slums, NSS 58th Round (July 2002 - December 2002)**		
93	486	Condition of Urban Slums, 2002: Salient Features	250	2080
		**Housing Condition, NSS 58th Round (July 2002 - December 2002)**		
94	488	Housing Condition in India, 2002: Housing stock and constructions	250	9280
95	489	Housing Condition in India, 2002: Household Amenities and Other Characteristics	250	9220
		**Consumer Expenditure, NSS 59th Round (January - December 2003)**		
96	490	Household Consumer Expenditure and Employment - Unemployment Situation in India	150	1580
		**Situation Assessment Survey of Farmers, NSS 59th Round (January - December 2003)**		
97	498	Indebtedness of Farmer Households	150	1380
98	499	Access to Modern Technology for Farming, 2003	250	1680
99	496	Some Aspects of Farming, 2003	250	2680
100	495	Consumption Expenditure of Farmer Households, 2003	250	2140
101	497	Income, Expenditure and Productive Assets of Farmer Households, 2003	250	3480
		**Land & livestock holdings and Debt & Investment, NSS 59th Round**		
102	493	Livestock Ownership Across Operational Land Holding Classes in India, 2002-03	150	1580
103	500	Household Assets and Liabilities in India as on 30.06.2002	250	4880
104	501	Household Indebtedness in India as on 30.06.2002	250	6000
105	502	Household Borrowings and Repayments in India during 1.7.2002 to 30.6.2003	250	4750
		**Employment & Unemployment, NSS 60th Round (January - June 2004)**		
106	506	Employment and Unemployment Situation in India, January - June 2004	250	3580
		**Consumer Expenditure, NSS 60th Round (January - June 2004)**		
107	505	Household Consumer Expenditure in India, January - June 2004	150	2580
		**Health, NSS 60th Round (January - June 2004)**		
108	507	Morbidity, Health Care and the Condition of the Aged	250	4480

* **The hard copies are out of stock. However, soft copies are available.**

Copies are available with the Deputy Director General, SDRD, NSSO, 164, Gopal Lal Tagore Road, Kolkata-700 108 on payment basis through Demand Draft drawn in favour of **"Pay & Accounts Officer, Ministry of Statistics & P.I., Kolkata". Postal Charges will be Rs. 85/- by Speed Post and Rs. 30/- by Regd. Parcel for single copy within India.**

For peer review only

STROBE Statement—Checklist of items that should be included in reports of ***cross-sectional studies***

	Item No	Recommendation
**Title and abstract**	1	(*a*) Indicate the study's design with a commonly used term in the title or the abstract Yes
		(*b*) Provide in the abstract an informative and balanced summary of what was done and what was found Yes
**Introduction**		
Background/rationale	2	Explain the scientific background and rationale for the investigation being reported Yes
Objectives	3	State specific objectives, including any prespecified hypotheses Yes
**Methods**		
Study design	4	Present key elements of study design early in the paper Yes
Setting	5	Describe the setting, locations, and relevant dates, including periods of recruitment, exposure, follow-up, and data collection Yes
Participants	6	(*a*) Give the eligibility criteria, and the sources and methods of selection of participants Yes
Variables	7	Clearly define all outcomes, exposures, predictors, potential confounders, and effect modifiers. Give diagnostic criteria, if applicable Yes
Data sources/ measurement	8*	For each variable of interest, give sources of data and details of methods of assessment (measurement). Describe comparability of assessment methods if there is more than one group Yes
Bias	9	Describe any efforts to address potential sources of bias Yes
Study size	10	Explain how the study size was arrived at Yes
Quantitative variables	11	Explain how quantitative variables were handled in the analyses. If applicable, describe which groupings were chosen and why Yes
Statistical methods	12	(*a*) Describe all statistical methods, including those used to control for confounding Yes
		(*b*) Describe any methods used to examine subgroups and interactions
		(*c*) Explain how missing data were addressed Method identical to baseline NSSO survey
		(*d*) If applicable, describe analytical methods taking account of sampling strategy
		(*e*) Describe any sensitivity analyses
**Results**		
Participants	13*	(a) Report numbers of individuals at each stage of study—eg numbers potentially eligible, examined for eligibility, confirmed eligible, included in the study, completing follow-up, and analysed Yes
		(b) Give reasons for non-participation at each stage
		(c) Consider use of a flow diagram
Descriptive data	14*	(a) Give characteristics of study participants (eg demographic, clinical, social) and information on exposures and potential confounders Yes
		(b) Indicate number of participants with missing data for each variable of interest
Outcome data	15*	Report numbers of outcome events or summary measures Yes
Main results	16	(*a*) Give unadjusted estimates and, if applicable, confounder-adjusted estimates and their precision (eg, 95% confidence interval). Make clear which confounders were adjusted for and why they were included Yes
		(*b*) Report category boundaries when continuous variables were categorized

For peer review only

		(*c*) If relevant, consider translating estimates of relative risk into absolute risk for a meaningful time period
Other analyses	17	Report other analyses done—eg analyses of subgroups and interactions, and sensitivity analyses Yes
**Discussion**		
Key results	18	Summarise key results with reference to study objectives Yes
Limitations	19	Discuss limitations of the study, taking into account sources of potential bias or imprecision. Discuss both direction and magnitude of any potential bias Yes
Interpretation	20	Give a cautious overall interpretation of results considering objectives, limitations, multiplicity of analyses, results from similar studies, and other relevant evidence Yes
Generalisability	21	Discuss the generalisability (external validity) of the study results Yes
**Other information**		
Funding	22	Give the source of funding and the role of the funders for the present study and, if applicable, for the original study on which the present article is based Yes

*Give information separately for exposed and unexposed groups.

Note: An Explanation and Elaboration article discusses each checklist item and gives methodological background and published examples of transparent reporting. The STROBE checklist is best used in conjunction with this article (freely available on the Web sites of PLoS Medicine at http://www.plosmedicine.org/, Annals of Internal Medicine at http://www.annals.org/, and Epidemiology at http://www.epidem.com/). Information on the STROBE Initiative is available at www.strobe-statement.org.

For peer review only

For peer review only - http://bmjopen.bmj.com/site/about/guidelines.xhtml

BMJ Open

Trends in addressing inequalities in access to hospital care in Andhra Pradesh and Maharashtra states of India

Journal:	*BMJ Open*
Manuscript ID:	bmjopen-2013-004471.R1
Article Type:	Research
Date Submitted by the Author:	18-Mar-2014
Complete List of Authors:	Rao, Mala; University of East London, Institute for Health and Human Development<br>Katyal, Anuradha; ACCESSHealth International,<br>Singh, Prabal; ACCESSHealth International,<br>Samarth, Amit; Sughavazhvu Healthcare,<br>Bergkvist, Sofi; ACCESSHealth International,<br>Kancharla, Manjusha; Indian School of Business,<br>Wagstaff, Adam; World Bank, Development Research Group<br>Netuveli, Gopalakrishnan; University of East London, Institute for Health and Human Development<br>Renton, Adrian; University of East London, Institute for Health and Human Development
**Primary Subject Heading**:	Public health
Secondary Subject Heading:	Health services research
Keywords:	Health economics < HEALTH SERVICES ADMINISTRATION & MANAGEMENT, Health policy < HEALTH SERVICES ADMINISTRATION & MANAGEMENT, PUBLIC HEALTH

SCHOLARONE™
Manuscripts

TITLE PAGE

TITLE:

Trends in addressing inequalities in access to hospital care in Andhra Pradesh and Maharashtra states of India

STUDY DESIGN: A cross-sectional difference in difference study with parallel control.

AUTHOR NAMES, ADDRESSES AND POSITIONS:

1.Mala Rao. Professor of International Health, Institute for Health and Human Development, University of East London, UK & Honorary Adviser, Administrative Staff College of India, Hyderabad

2. Anuradha Katyal, Consultant, ACCESS Health International, Hyderabad, India

3.Prabal V Singh, Director Research, ACCESS Health International, Hyderabad, India

4.Amit Samarth, Head, Strategy and Planning, SughaVazhvu Healthcare, Thanjavur, India

5.Sofi Bergkvist, Managing Director, ACCESS Health International, Hyderabad, India

6.Manjusha Kancharla, Research Associate, Indian School of Business, Hyderabad India

7.Adam Wagstaff, Research Manager, Human Development & Public Services team, Development Research Group (DECRG), The World Bank, Washington DC, USA

8.Gopalakrishnan Netuveli. Professor of Public Health, Institute for Health and Human Development, University of East London & ESRC International Centre for Life Course Studies in Society and Health, University College London, UK

9.Adrian Renton. Director, Institute for Health and Human Development, University of East London, UK

All authors had full access to all the data (including statistical reports and tables) and can take responsibility for the integrity of the data and the accuracy of the data analysis

Corresponding author and guarantor of the data and information: Mala Rao (best email address: m.rao@uel.ac.uk).

M Rao affirms that the manuscript is an honest, accurate and transparent account of the study being reported and no important aspects of the study have been omitted.

For peer review only - http://bmjopen.bmj.com/site/about/guidelines.xhtml

For peer review only

ABSTRACT. WORD COUNT: 298

OBJECTIVES

To compare the effects of the Rajiv Aarogyasri Health Insurance Scheme of Andhra Pradesh (AP) with health financing innovations including the RSBY in Maharashtra (MH) over time on access to and out of pocket expenditure (OOPE) on hospital inpatient care.

STUDY DESIGN

A cross-sectional difference in difference (DID) study with parallel control.

SETTING

National Sample Survey Organisation of India (NSSO) urban and rural 'first stratum units', 863 in AP and 1008 in MH.

METHODS

We used two cross-sectional surveys: as a baseline, the data from the NSSO 2004 survey collected before the AArogyasri and RSBY schemes were launched and as post-intervention, a survey using the same NSSO methodology conducted in 2012.

PARTICIPANTS

8623 households in AP and 10073 in MH

MAIN OUTCOME MEASURES

Average OOPE, large OOPE and large borrowing per household per year for inpatient care, hospitalisation rate per 1000 population per year.

RESULTS

Average expenditure, large expenditures and large borrowings on inpatient care had increased in both MH and AP, but the increase was smaller in AP across these 3 outcome measures. DIDs for average expenditure and large borrowings were significant and in favour of AP for the rural and the poorest households. Hospitalisation rates also increased in both states but more so in AP , although the DID was not significant and the sub group analysis presented a mixed picture.

CONCLUSIONS

Health innovations in AP had a greater beneficial effect on inpatient care-related expenditures than innovations in MH. The Aarogyasri scheme is likely to have contributed to these impacts in AP at least in part. However, out of pocket expenditure increased in both states over time. Schemes such as the Aarogyasri and RSBY may result in some positive outcomes but, additional interventions may be required, to improve access to care for the most vulnerable sections of the population.

For peer review only - http://bmjopen.bmj.com/site/about/guidelines.xhtml

ARTICLE SUMMARY

STRENGTHS AND LIMITATIONS OF THE STUDY

- This study uses a quasi-experimental design to compare changes between the Indian states of Andhra Pradesh and Maharashtra, in hospital inpatient care related expenditures and behaviours before and after the rollout of the Aarogyasri and RSBY schemes
- The study based on a survey of 18696 households has shown that health innovations in Andhra Pradesh had a greater beneficial effect on hospital inpatient care-related expenditures and access than innovations in Maharashtra. The Aarogyasri scheme is likely to have contributed to these impacts in Andhra Pradesh.
- The study also highlights the implications of the findings for policy and practice and additional interventions necessary to address gaps in the availability of and access to care.
- The study is only able to compare the effects of health innovations over time across the 2 states but does not allow the drawing of inferences on the impacts of individual initiatives.
- The study uses the difference in difference methodology and its findings may have been affected by unobservable differential changes between the 2 states.

For peer review only

For peer review only - http://bmjopen.bmj.com/site/about/guidelines.xhtml

INTRODUCTION

In 2005, member states of the World Health Organization (WHO) committed to develop their health financing systems to deliver universal coverage (UC); that is that all people have access to health services and do not suffer financial hardship paying for them[1] . WHO's 2010 World Health Report[2] recommended *inter alia* that countries reduce reliance on direct payments, and improve equity of access, including through the introduction of prepayment schemes. However, one recent systematic review of the impact of national health insurance schemes in low and middle income countries[3] found only weak evidence of increased use of healthcare and reduced out-of-pocket expenses, with the poorer benefitting less, whilst others[4,5] concluded that health insurance improved health care access and use, as well as financial protection in most cases but had no conclusive impact on health status. Both highlighted the need for more rigorous assessments of such schemes.

India has one of the highest levels of out-of-pocket health expenditure (more than 80% of private health expenditure)[6]. The aim of our study was to explore how recently introduced health financing initiatives have affected access to and out-of-pocket expenditure (OOPE) on inpatient hospital care in the Indian states of Andhra Pradesh (AP) and Maharashtra (MH).

BACKGROUND

In its *Eleventh Five Year Plan (2007-12)*[7], Government of India sought to increase Public Expenditure on Health and to strengthen investment in rural health infrastructure through the National Rural Health Mission. Its *Twelfth Five Year Plan (2012-17)*[8], reflected the recommendation of the Planning Commission's High Level Expert Group for general taxation to be the principal source of health care financing. It proposed the development of government-funded health insurance schemes, building on the evidence from experimental schemes being introduced across many States. In India, health is primarily a state rather than national responsibility[6]. Whilst it is recognised that political will and good governance are essential, both the political mobilization of funds for health schemes and the effectiveness and efficiency of funded schemes will be enhanced by robust evidence which documents as to whether schemes achieve objectives, what works well and the main challenges they face.

In India, evaluation is not routine, even of large costly public health care programmes. Nevertheless, some assessments have been carried out, for example of the Yeshasvini Co-operative Farmers Health Care Scheme of Karnataka, the longest running state-supported health insurance scheme for the informal sector in India[9], with promising results in terms of increased utilization of and reduced borrowing for health care services[10]. However, these early models covered small populations and offered limited benefits, so that the policy implications of conclusions drawn from even the best evaluations were unclear.

This scenario has changed during the past 5 years, with the launch of 2 schemes, the Rajiv Aarogyasri[11] Community Health Insurance Scheme (Aarogyasri) of Andhra Pradesh (AP) and Rashtriya Swasthya Bima Yojana (RSBY) currently offered in 30 states and union territories of India[12] including AP's neighbouring state of Maharashtra (MH). Their scale in terms of population coverage and range of treatments offered, significantly enhances their potential to inform India's road map towards universal health coverage. Both schemes belong to the new generation of publicly funded government-sponsored health insurance schemes, principally aimed at providing financial protection to the poor against catastrophic health shocks, which, for these schemes, the Government has defined as inpatient hospital care[9]. Both schemes have been subject to some assessment in their early phases, but a recent editorial[13] highlighted the necessity of further and repeated evaluations to enhance the credibility and accountability of existing schemes and to identify those which deserve scale-up. The Aarogyasri and RSBY schemes and the other recent health sector innovations in AP and

For peer review only - http://bmjopen.bmj.com/site/about/guidelines.xhtml

Maharashtra which form the major backdrop and complex health care architecture against which these have been launched, and which may have contributed to the changes in outcomes we have explored in our study, are described below.

The Rajiv Aarogyasri Health Insurance Scheme of AP

In 2007 AP launched a pioneering new state-wide fully state-funded health insurance scheme, the Rajiv Aarogyasri Community Health Insurance Scheme (Aarogyasri)[11] to provide treatment for serious and life-threatening illnesses. The specific objectives include: to improve access of poor families to quality medical care (meaning low-frequency, high cost specialist care) and treatment of identified diseases requiring hospitalisation through an identified network of health care providers, to provide financial cover for catastrophic illnesses which have the potential to wipe out life time savings of poor families and to provide 'universal coverage to the urban and the rural poor in the state[14] albeit for the conditions covered in the benefits package. All families with a 'below poverty line' (BPL) ration card, i.e. those on an annual income below USD 1384 (INR 75,000) in urban areas and USD 1107 (INR 60,000) in rural areas, and including individuals with pre-existing medical conditions are *automatically* enrolled and the scheme was estimated to cover approximately 20.4 million poor and lower middle class families, comprising about 85 percent of the state's population in 2009[9]. Enrollees make no contribution, the annual benefit is a maximum of USD 4,500 (INR 200,000) per family per year and there is no limit on the size of the family[14]. A total of 942 medical and surgical procedures across 31 clinical specialties [14] are provided and the benefits include all inpatient costs - associated investigations, food, transport and medicines for 10 days following discharge. One year follow-up packages including consultation, medicines, and diagnostics are also available for 125 procedures requiring longer periods of follow up[9] . Aarogyasri has unique features including *Aarogyamithras* (health system navigators), outreach *health camps* delivered by participating hospitals to educate, screen and case-find and a state-of-the-art information technology-based management system. At the time of this study, 353 public and private sector hospitals were 'empanelled' to provide services to Aarogyasri beneficiaries.

In 2009, a descriptive study of *Aarogyasri*, based on an analysis of claims data and a survey of beneficiaries[15], concluded that while the scheme was beginning to reach its intended beneficiaries uptake was lower among scheduled castes and tribes. This was confirmed by Fan and colleagues[16], who used variations in programme roll-out over time and districts to evaluate the scheme using National Sample Survey data collected before and after its launch. They reported reduced out-of-pocket expenditure in this initial phase but no major impact on catastrophic healthcare expenditure. Inspired by Aarogyasri and mindful of the political benefits of introducing popular health reforms, other states have launched health financing innovations similar to this model.

RSBY in Maharashtra

RSBY was launched across a number of states by the Ministry of Labour, Government of India (GOI) in 2008[17] and provides access to free inpatient hospital care up to USD 550 (INR 30,000) per family per year[18]. Households which meet the criteria based on the much more limiting definition of poverty and numbers of poor families provided for each State by the GOI Planning Commission are eligible to enrol, and pay a contribution of USD 0.55 (INR 30) at registration and at each annual renewal[9]. Up to 5 family members, including those with pre-existing conditions can be covered, and personal information including biometric data are collected prior to the issue of a smart card with encoded details of the family. 700 procedures covering 18 broad categories of interventions which would generally be included under the umbrella of 'secondary' care, are provided and the benefit packages include the intervention, public transport costs limited to 1.8 USD (INR 100) per visit and 18.2 USD (INR 1000) per year and post-hospitalization drugs for 5 days. Networked hospitals are required to provide free outpatient consultations (which have only recently been introduced. Personal communication, Kurian OC) but other costs such as ambulatory diagnostics and medicines

For peer review only - http://bmjopen.bmj.com/site/about/guidelines.xhtml

have to be borne by the beneficiaries, except if investigations lead to inpatient admissions within a day[9]. A pilot of the RSBY scheme was launched in 1 district of AP, but only after the start of our household survey.

In Maharashtra, enrolment began in Aug 2009, and by mid 2013, approximately 2 million of the eligible 4 million families were enrolled in the scheme[12] which is being implemented in 31 of 35 districts in the state. Enrolment had extended to 26 districts prior to June 2012, when our household survey began. Notably, only 15 out of 1215 hospitals contracted for RSBY funded services are from the public sector [12]. Early assessments of the scheme's impact nationally, suggest that although the rate of hospitalisations has increased, awareness of the scheme was poor and remains a barrier to uptake. Notable variations in enrolment and scheme awareness were also observed by a descriptive study of RSBY conducted in the Amravati district of Maharashtra which has a large tribal population[19]. In Maharashtra[17], utilisation rates have been reported to be lower than in other states and the male:female enrolment ratio is 6.5:3.5.

Other major health sector initiatives in AP and Maharashtra
Both states have a complex health care landscape with numerous programmes in place. There are several initiatives launched during the past decade, some of which are common to both states and driven by national strategies, and others owe their existence to state-level enterprise, innovation and political support. The most notable programmes with the potential to impact on in patient care are described below.

The National Rural Health Mission (NRHM) was launched in 2005 nationwide, with a key aim of reducing maternal and infant mortality[20]. Government reports suggest that its notable achievements include an increase in institutional deliveries; in AP from 1.25 million in 2005-06 to 1.46 million by 2011-12[21] and in MH from 1.1 million to 1.63 million[22], achieving an institutional delivery rate of approximately 92 percent in both states. Also common to both states is the '104 health information help line' launched in AP in 2008 and MH in 2011[23], to provide medical advice and information based on validated algorithms and disease summaries, direct callers to appropriate health facilities or to receive a complaint against a public sector health facility. In AP the help line and call centre were subsumed within the Aarogyasri infrastructure by 2011.

In MH, the RSBY was preceded by the Jeevandayee scheme launched in 1997 with the objective of reducing catastrophic OOPE on inpatient care in the BPL population[24]. Potential beneficiaries were required to apply for funding after a diagnosis was confirmed and the scheme covered serious illness such as cardiac and renal disease and cancer. However, the scheme uptake has been low, and while it has continued to run in parallel to the RSBY, only 66,853 procedures (4456 procedures per year in a state with 112.37 million people) have been approved during the scheme's lifetime[25]. Since 2006, MH has also had a scheme in place which mandated 20% of the beds in private hospitals to be made available for free or at subsidized rates to poor patients (personal communication, Kurian OC). It has been estimated that around 10,000 private beds are available for the poor across MH, equivalent to approximately 20% of the total bed capacity of the public sector. Although the implementation of the scheme is reported to be erratic (Kurian OC), it may have had some positive impact on access to hospital inpatient care for serious illness.

Launched in 1995-96, the Navasanjeevani Yojana scheme is exclusive to the 15 tribal districts of MH and was to improve maternal and infant mortality in these vulnerable populations[26]. It has focused on strengthening primary health and nutrition services and access to safe drinking water.

A service available in AP but not in MH is the '108' scheme, launched in 2005 to provide a state-of-the-art medical emergency response service[27]. At the time of our study, 802 ambulances catered to approximately 3,500 emergencies per day[28].

For peer review only - http://bmjopen.bmj.com/site/about/guidelines.xhtml

OBJECTIVES OF THE STUDY

Our objective was to compare the effects of health innovations over time on access to and OOPE on inpatient care in AP and MH and to assess whether the AP initiatives had larger or smaller beneficial effects than those found in MH. These differential effects are likely to be substantially due to the Aarogyasri scheme in AP and the RSBY in MH. In this paper, we report findings from a study which compared these trends. The findings do not allow us to draw inferences on the impacts of individual initiatives, but nevertheless contribute new knowledge on the impact and role of the innovations, provide lessons for other programmes, and strengthen the evidence base for policy on UC in India.

METHODS

Overview

None of the aforementioned initiatives – including the Aarogyasri and RSBY schemes in which we are especially interested – was piloted in a systematic way, let alone via a carefully designed randomized control trial. Following MRC Guidance[29,30] and best practice we therefore opted for a quasi experimental design in which we seek to minimise selection bias, to control for confounding variables and to reduce the effects of chance. Specifically, we compare changes in hospital inpatient care related expenditures and behaviours (HREB) in AP and MH before and after the rollout of the Aarogyasri scheme in AP and the RSBY scheme in MH. The difference in changes between AP and MH is not an estimate of a specific initiative. Rather it tells us whether, on balance, the AP initiatives have had larger (or smaller) beneficial effects than the MH initiatives, and if so how much more (or less) beneficial they have been. Since the NRHM was common to both states and the MH-specific initiatives were quite small in scale or unlikely to affect HREBs, any difference in change between the two states is quite likely to be mainly due to differential effects of the Aarogyasri and RSBY programmes.

AP and MH have a broadly similar development profile as shown by the data below. AP's other socio-economically similar neighbouring states of Karnataka and Tamil Nadu had already introduced Aarogyasri-like schemes, and Odisha and Chattisgarh the only other neighbours, had comparatively higher levels of socio-economic deprivation. HREBs were measured in both AP and MH by two waves of household survey before (2004) and after (2012) the introduction of Aarogyasri and RSBY. The study protocol and questionnaire for the 2012 survey were reviewed and agreed by the Research Ethics Committee of the Administrative Staff College of India, Hyderabad.

Maharashtra	Indicator	Andhra Pradesh
112.37	Population (2011 census, in millions)	84.66
10.20	% Schedule Caste (2001 Census)*	16.60
8.90	% Schedule tribe (2001 census)*	6.20
101,314	Per capita income 2011-12 (in INR)**	71,540
35	Number of districts	23
5,314	Households covered in NSSO 60th round (2004-05)	5,059
10,073	Households covered in the study 2012	8,623

*Note that 2011 census data for social groups are not yet published

**Source: Presentation on Annual Plan 2012-13 and Five Year Plan 2012-17[31]

Survey design

For peer review only - http://bmjopen.bmj.com/site/about/guidelines.xhtml

Baseline Survey : 2004

We used the original data from the National Sample Survey Organization (NSSO) 60th decennial round household survey undertaken in 2004[32] to estimate baseline HREB estimates for AP and MH (Table 1). This was the most recent round measuring morbidity profiles, use of health care services including hospitalised and non-hospitalised treatments and expenditures incurred. The household survey used a multi-stage stratified sampling methodology to identify a representative random population sample and an interviewer completed questionnaire to obtain measures of HREB along with socio-demographic, household expenditure and other information. (see Online Appendix for full technical details of sampling method and full questionnaire).

Follow up survey: 2012

We used the same household survey design and methods to collect post-intervention data in AP and MH as those used by NSSO. Briefly, the household survey used a multi-stage stratified sampling methodology with the 'First Stage Units' (FSUs) identical to those used by NSSO in their 66th round (2008-09)[33], the latest round for which FSUs had been mapped. However, the FSUs were not the same as those in NSSO 2004, our baseline survey, rapid urbanisation having changed substantially, the urban-rural landscape of both states and thus the geographical basis for sampling units.

Table 1: Urban and rural populations and households surveyed in 2004 and 2012 in Andhra Pradesh and Maharashtra

	**Andhra Pradesh**		**Maharashtra**	
	NSSO 60th round 2004-05	Our survey 2012	NSSO 60th round 2004-05	Our survey 2012
Population	76,210,007*	84,665,533**	96,878,627*	112,372,972**
Urban population	20,808,940*	28,353,745**	41,100,980*	50,827,531**
Rural population	55,401,067*	56,311,788 **	55,777,647*	61,545,441**
Total households (urban)	4,397,138*	6,778,225**	8,403,224*	10,813,928**
Total households (rural)	12,607,167*	14,246,309**	11,173,512*	13,016,652**
Total households	17,004,305*	21,024,534**	19,576,736*	23,830,580**
FSUs (urban)	183	372	267	504
FSU (rural)	325	491	265	504
Total households surveyed (urban)	1824	3715	2664	5038
Total households covered (rural)	3235	4908	2650	5035

*2001 census ** 2011 census

FSU - First Stratum Unit

The NSSO 66th round had 492 rural FSUs in AP, but 1 FSU was found to be uninhabited.

For peer review only

The interviewer completed household survey questionnaire was pre-tested and then piloted in both states prior to the survey. All respondents provided written informed consent for participation. Questions addressed the following: household composition and socio-demographic characteristics of members, household expenditure, and health expenditure (outpatient and inpatient) and means of its financing, healthcare seeking behaviour, factors affecting access to healthcare and awareness and perceptions of the quality of the *Aarogyasri* scheme (AP only). The survey questions in 2012 were identical to those from the NSSO 2004[34]. Additional questions specific to the Aarogyasri and other relevant schemes were also added.

A survey of 18696 households across 2 states and 1871 locations within the states is a challenging undertaking. The survey design had several features intended to assure the quality of data collected. Few academic institutions have internal capacity to carry out such large surveys, and consequently, the Social and Research Institute of IMRB International, a leading market research agency was selected to carry out the survey. The Institute has field survey teams based in every Indian state, conversant in local languages and dialects and trained to carry out surveys in the socio-economic development sector. Its clients include Government of India (for whom the national Family Health Survey data are collected), World Bank and other UN organisations. A group of NSSO consultants in AP and the Indian Socioeconomic Research Unit, Pune were recruited to support the training of the field survey teams and data verification.

We planned three levels of verification of the study data; the first to be undertaken by the survey agency, the second to be carried out by the study team and the third, by the agencies mentioned above. Survey teams for each district were accountable to a field supervisor who was responsible for checking both the household listing and data entry on a daily basis. The study team also accompanied the field staff to survey sites on a regular basis. Data collected from 250 households in each state (approximately 2.5% of the surveyed households) and 186 of the FSU listings (approximately 10%) were independently verified by the agencies in both the villages and urban blocks in order to ensure that the sampling method and administration of the questionnaire survey were being correctly applied. The data entry was carried out by the Institute using a double entry method and any questionnaires reported incorrect were sent back to the field for re-survey. The research team carried out a final validation and review of the data.

Outcome measures

Average inpatient (IP) expenditure per household per year. Average out-of-pocket expenditure for inpatient care during 1 year prior to the survey was estimated from questionnaire responses for AP and MH from both baseline and follow-up data. Reimbursements for inpatient expenditure were deducted from the total where households had received them.

Large out-of-pocket inpatient expenditure. Because of the limited data on household consumption in the 2004 NSSO health survey we did not estimate 'catastrophic health expenditure'. Instead, we constructed a measure of 'large' out-of-pocket expenditure. The Aarogyasri Health Care Trust data on expenditure incurred by the Government of AP per case in 2012 were examined[14] and the mean was estimated as USD 419 (INR 23,000). A household was deemed to have incurred 'large' expenditure if OOPE for inpatient care was equal to or greater than this threshold.

Large borrowing. We estimated the total amount borrowed by a household to meet the expenditure of all the inpatient episodes of that family during the previous year. A household was considered to have incurred 'large borrowing' if the borrowing was equal to or exceeded the BPL threshold set by Government of AP: INR 70000 for urban families and INR 65000 for rural households. These prices have been deflated to 2004 levels.

For peer review only - http://bmjopen.bmj.com/site/about/guidelines.xhtml

Hospitalisation rate. This was estimated as the number of individuals hospitalised during the previous year, per 1000 population.

Variations in outcomes were examined between male and female-headed households, and rural and urban populations, as well as across social groups and economic groups represented by asset quintiles.

Because of the limited data on household consumption in the 2004 NSSO health survey which made the estimation of wealth difficult, we have opted instead to measure household living standards using an asset or wealth index based on information on ownership of household durables, dwelling type, etc., using principal component analysis to estimate weights[35, 36,37] for each indicator. The indicators used were limited to those collected in the 2004 NSSO: type of structure of the dwelling unit, type of toilet, type of fuel used for cooking and source of drinking. Data from the 2004 and 2012 surveys were pooled so that the index captures changes in living standards between the two years; there are therefore more households in the top quintile in 2012 than in 2004. The statistical software Stata 11 was used to generate the index.

Deflation of follow-up expenditure estimates.
The 2012 expenditure data including the threshold for large expenditures were deflated using the consumer price index of the Government of India[38] to reflect 2004 prices.

Analysis
Our repeat cross-sectional surveys do not allow estimation of within-individual household changes in outcomes over time. Our analysis therefore focused on estimating outcomes averaged across states, and in comparing changes in these over time between AP and MH. If we assume that outcome determinants other than Aarogyasri and RSBY remained stable in the two states over time or followed a parallel trend, then a difference-in-difference (DID) analysis will uncover the net effect of Aarogyasri over and above RSBY.

The difference in differences of outcome (Y_{DD}) is

$$(Y_{2012}^{AP} - Y_{2004}^{AP}) - (Y_{2012}^{MH} - Y_{2004}^{MH})$$

where the subscripts and superscripts for Y refer to the respective states and the years when the surveys were done. Confidence intervals were calculated from the standard error Y_{DD} of and the p-value for the null-hypothesis ($Y_{DD} = 0$) was tested using the Wald test as $t = \frac{Y_{DD}}{SE_{Y_{DD}}}$ with one degree of freedom. Y_{DD} was estimated using ordinary least square regression:

$$y_{it} = \beta_0 + \beta_1 state_i + \beta_2 survey_t + \beta_3 (state * survey)_{it} + \sum_{k=1}^{m} \beta_{3+k} covariate_k + \varepsilon$$

The basic DID results are obtained using the above regression with covariates excluded. The adjusted DID results are obtained using the above regression with m=9 covariates, namely the gender of head of household, a dummy variable capturing whether the household lives in a rural or urban location, three dummy variables capturing the household's social group (the lowest is the excluded category), and four asset quintile dummies (the bottom is the excluded category). In the regression y_{it} is the outcome, *state* is a dummy variable with 0 for MH and 1 for AP, and *survey* is a dummy variable with 0 for 2004 survey and 1 for 2012 survey. The coefficient for the interaction term, β_3, gives the differences in differences estimate, Y_{DD}. Robust standard errors of Y_{DD} were calculated to account for clustering of households within FSUs using Stata survey commands. A positive value for Y_{DD}

For peer review only - http://bmjopen.bmj.com/site/about/guidelines.xhtml

suggested that the change in the outcome in AP was more than the change in MH and the negative value would suggest the reverse.

An advantage of regression based DID estimate is this ability to use co-variates which can account for differential trajectories in the two states. In addition to this we did sub-group analysis stratifying for different co-variates. This is particularly relevant in the case of scheduled tribes whose proportion increased in MH in the follow up survey.

Sub groups were not mutually adjusted for the analysis due to sample size restrictions in relation to some of them.

Role of the funding sources

The external funding sources had no role in study design, data collection, analysis, interpretation or reporting, or in submission decision.

RESULTS

A total of 5314 and 5059 households from Maharashtra and AP were surveyed by the NSSO in 2004 (Table 2). Our survey in 2012 included 10073 (MH) and 8623 (AP) households.

Table 2. Socio-demographic characteristics of baseline and follow-up samples

Subgroups	Number (%) of Households 2004		Number (%) of Households 2012	
	Maharashtra	Andhra Pradesh	Maharashtra	Andhra Pradesh
**All**	5314	5059	10073	8623
**Head of Household**				
Male	4785 (90.0)	4433(87.6)	8543(84.8)	7418(86.0)
Female	529 (10.0)	626 (12.4)	1530 (15.2)	1205 (14.0)
**Social Group**				
Scheduled Tribes	413 (7.8)	296 (5.9)	1364 (13.5)	883 (10.2)
Scheduled Castes	809 (15.2)	974 (19.3)	2235 (22.2)	1797 (20.8)
Other Excluded	1644 (30.9)	2317 (45.8)	1899 (18.9)	3419 (39.7)
All other Groups	2448 (46.1)	1472 (29.1)	4571 (45.4)	2524(29.3)
**Location**				
Rural	2,650 (49.9)	3235 (63.9)	5035(50.0)	4908 (57.0)
Urban	2664 (50.1)	1824 (36.1)	5038 (50.0)	3715(43.0)

For peer review only - http://bmjopen.bmj.com/site/about/guidelines.xhtml

Asset Quintile				
Lowest	1,260 (23.7)	1,594(31.5)	996(9.9)	826(9.6)
Second	1,016 (19.1)	1,237 (24.5)	1,841(18.2)	1,286(14.9)
Third	772 (14.5)	753(14.9)	2,228(22.1)	2,121(24.60)
Fourth	857 (16.1)	744(14.7)	2,373 (23.6)	3,072(35.6)
Fifth	1,408(26.5)	730(14.4)	2,633(26.1)	1,318(15.3)

For peer review only

Changes in average in-patient expenditure

Table 3 (top panel) shows average baseline levels of inpatient expenditure. The table also shows the real terms change (deflated to 2004 prices) in these outcomes at follow up and the difference in difference (DID) estimate comparing AP with MH. DIDs for overall results are shown unadjusted, as well as adjusted for the effects of the covariates. Breakdowns by sex of head of household, social group, urban/rural location and asset quintiles are also shown.

Overall, average inpatient expenditure increased in real terms in both the states between 2004 and 2012, but the increase was significantly greater in MH (unadjusted DID= -498.2 INR, 95% CI= -792.9: -203.5, p=0.0009). The trend of a greater increase in MH was evident across all sub groups of analysis except the richest asset quintile. However, the DIDs reached significance in male headed households (DID=-513.7INR, 95% CI= -843.9: -183.4, p=0.0023), scheduled castes (DID= -708.7INR , 95% CI=-1234.3: -183.2, p=0.0082), all 'other' social groups (DID=-1110.46INR, 95% CI=-1868 : -352.9, p=0.0041), rural households (DID= -504 INR, 95% CI= -801.9: -206.0, p=0.0009) and the poorest (DID= -1001.3 INR, 95% CI= -1751: -251.7, p= 0.0089) and middle asset quintiles (DID= -798.1 INR, 95% CI= -1362.9:-233.3, p= 0.0056).

Table 3: Change in average inpatient expenditure (in INR) in Maharashtra and Andhra Pradesh between 2004 and 2012

Subgroups	Baseline Mean (95% CI)		Change 2004:2012 Mean (95% CI)		DID	
	Maharashtra	Andhra Pradesh	Maharashtra	Andhra Pradesh	Mean (95%CI)	p
**Household inpatient expenditure**						
**All**	1091.6(978.4:1204.8)	723.5(527.5:919.5)	942.8(749.9:1135.6)	444.55(221.5: 667.6)	-498.2(-792.9:-203.5)	0.0009
					**DID (adjusted for covariates)**	
					-565.8(862.9: -268.6)	0.0002
**Head of Household**						
Male	1,132.9(1015:1,251)	758(419.7:1,096.3)	935(727:1,143.01)	1074.9(555.9:1593.8)	-513.7(-843.9: -183.4)	0.0023

For peer review only - http://bmjopen.bmj.com/site/about/guidelines.xhtml

Female	757.1(555.5:1014.6)	341.1(222.3:460.01)	421.3(164.7:678.0)	589.9(307.22:872.8)	-484.9(- 1075.6:105.9)	0.1076
**Social Group**						
Scheduled Tribes	376.6(231.7:521.6)	432.7(212.52:652.9)	1153.1(803.3:1502.9)	675.2(163.2:1187.2)	-477.9(- 1097.7:142)	0.1307
Scheduled Castes	696.7(500.2:893.2)	432.6(305.6:559.4)	1464.1(1039.9:1888.4)	755.4(444.9:1065.9)	-708.7(- 1234.3: - 183.2)	0.0082
Other Excluded	1,028.6(838.4:1218.8	562.4(463.7:662)	928.9(532.9:1324.9)	767.9(569.6:966.2)	-161(-603.7: 281.7)	0.4758
All other Groups	1,424.5(1,222.3:1626.7)	1306.2(627.8:1984.6)	734.9(427.9:1041.7)	-375.6(- 1,068.5:317.4)	-1110.46(- 1868 : -352.9)	0.0041
**Location**						
Rural	897.8(768.1:1027.5)	571.4(496.2:646.6)	1084.7(826.3:1343.1)	580.7(432.2:729.2)	-504(-801.9: -206.0)	0.0009
Urban	1343.5(1146.1:1540.9)	1113.5(466.2:1760.8)	753.6(458.7:1048.6)	92.3(-586.92:771.5)	-661.3(- 1401.5: 78.864)	0.0799
**Quintile**						
poorest	656.3(498.0:814.6)	391.5(319:464.1)	1692.5(1053.3:2331.7)	691.2(298.9:1083.5)	-1001.3(- 1751: -251.7)	0.0089
2nd	786.5(583.5:989.5)	443.3(356.5:530.2)	979.3(599.4:1359.2)	839.5(465.7:1213.3)	-139.8(- 672.5:393)	0.607
middle	1062.7(738.8:1386.1)	862.1(577.8:1146.5)	1011.8(550.2:1473.4)	213.7(-112.1:539.6)	-798.1(- 1362.9: - 233.3)	0.0056
4th	1241.7(894.4:1589.1)	1819(337.5:3302.5)	803.6(328.7:1278.5)	-644.3(-2128.3: 839.7)	-1447.9(- 3005.2:109.5)	0.0684
richest	1818.6(1505.5:2131.8)	908.3(682.1:1133.4)	252.3(-193.4:698.1)	362.1(15.3:708.9)	109.7(- 454.80:674.3)	0.7031

Large expenditures for inpatient care

Proportions of households incurring large expenditures showed an increase in both states (Table 4), but the increase was smaller in AP for the sample as a whole as well as for all the groups except for the second asset quintile. The DID was strongly significant for the households overall (adjusted DID=-1.8, 95% CI: -3:-0.7, p= 0.0009), but this was not observed for any of the sub groups of analysis.

Table 4: Change in the proportion (%) of households incurring large health expenditures for in-patient care in Maharashtra and AP between 2004 and 2012

Subgroups	Baseline Mean (95% CI)		Change 2004:2012 Mean (95% CI)		DID	
	Maharashtra	Andhra Pradesh	Maharashtra	Andhra Pradesh	Mean (95%CI)	p
Large IP expenditure (INR 23000 deflated to 2004 figures)						
**All**	6.7(6:7.3)	3.4(2.9:3.9)	3.1(2.1:4.1)	2.2(1.5:2.8)	-0.91(-2.1:0.27)	0.1302

For peer review only - http://bmjopen.bmj.com/site/about/guidelines.xhtml

					DID (adjusted for covariates)	
					-1.8(-3:-0.7)	0.0009
**Head of Household**						
Male	6.8(6.2:7.6)	3.5(3.1:4)	3.1(2.0:4.1)	2.1(1.5:2.9)	-0.8(-2.1:-0.4)	0.1928
Female	5.0(3.7:6.4)	2.8(1.9:3.7)	3.9(2:5.8)	2(6.6:3.4)	-1.8(-4.2:0.50)	0.1222
**Social Group**						
Scheduled Tribes	2.2(1.3:3.1)	1.4(0.5:2.2)	5.3(3.5:7)	3.5(1.9:5.1)	-1.7(-4.1:0.61)	0.1478
Scheduled Castes	6.1(4.6:7.7)	2.1(1.5:2.6)	4.3(2.3:6.3)	3(1.9:4.1)	-1.2(-3.5 :1.01)	0.2785
Other Excluded	5.9(4.7:7.0)	3.3(2.7:3.8)	2.9(1.1:4.6)	2.7(1.7:3.6)	-2.1(-2.2:1.8)	0.8389
All other Groups	8.3(7.5:7.9)	5.4(4.4:6.3)	2.2(0.9:3.6)	0.51(-0.7:1.7)	-1.7(-3.5:0.04)	0.0628
**Location**						
Rural	1.9(1.5:2.2)	0.9(0.79:1.1)	1.7(1.1:2.3)	1.3(0.09:1.6)	-0.45(-1.1:0.25)	0.2098
Urban	12.9(11.9:14)	9.7(8.9:10.7)	4.4(3.0:5.7)	3.9(2.6:5.3)	-0.7(-2.4: 1.5)	0.6350
**Quintile**						
poorest	1.8(1.3:2.4)	1.1(0.8:1.4)	3.7(2.2:5.2)	1.7(0.7:2.7)	-0.2(-3.8:-0.19)	0.0307
2nd	2.7(1.9:3.5)	1.2(0.9:1.5)	2.1(0.93:3.3)	2.2(1.4:3.1)	0.9(-1.4:1.6)	0.9079
middle	6.9(4.9:8.9)	4.2(3.1:5.3)	1.3(-1:3.6)	0.9(-1.2:1.4)	-1.2(-3.9:1.4)	0.3596
4th	10.7(8.7:12.6)	7.6(5.9: 9.2)	1.8(-0.57:4.3)	-0.036(-1.9:1.80)	-1.9(-4.9:1.2)	0.2268
richest	13.5(12:14.9)	9.6(8.1:11.2)	0.3(-1.6:2.2)	-0.6(-2.8:1.6)	-0.9(-3.7:2)	0.5601

Changes in large borrowing for inpatient care

In both states proportions of households incurring large borrowings to meet inpatient expenses increased from 2004 to 2012 (Table 5). However, there was a consistent pattern of smaller increases in AP for the overall population, as well as all subgroups (except the richest asset quintile) with DIDs strongly significant for the overall population (adjusted DID=-4, 95% CI: -6.6:-1.4, p= 0.0032), scheduled tribes (DID= -5.5, 95% CIs:-9.3:-1, p= 0.0048), rural households (DID= -4.7, 95% CIs: -7.3 : -2.1, p= 0.0007) and all asset quintiles except the richest (the poorest asset quintile DID= -9.0, 95% CI: -14.0: -4.4, p=0.0002).

Table 5: Change in the proportion (%) of households large borrowings for in-patient care in Maharashtra and AP between 2004 and 2012

Subgroups	Baseline Mean (95% CI)		Change 2004:2012 Mean (95% CI)		DID	
	Maharashtra	Andhra Pradesh	Maharashtra	Andhra Pradesh	Mean (95%CI)	p
	Proportion of households having large borrowings					
**All**	7.5(6.7:8.2)	3.8(3:4.5)	8.9(6.8:11)	5.3(3.4:7.2)	-3.7(-6.4:-0.908)	0.0100
					DID (adjusted for covariates)	
					-4(-6.6:-1.4)	0.0032
**Head of Household**						

For peer review only - http://bmjopen.bmj.com/site/about/guidelines.xhtml

Male	7.8(7.0:8.5)	3.9(3.1:4.7)	9.8(6.6:1.3)	5.3(3.3:7.3)	-3.6(-6.6: -0.62)	0.0187
Female	5(3:7)	2.9(1.7:4.1)	8.(6.6:11.2)	5(2.9:7.23)	-4.7(-8.3:-1)	0.0137
**Social Group**						
Scheduled Tribes	3.6(2.3:4.9)	2.4(0.93:3.9)	11(8.9:14)	5.8(2.8:8.8)	-5.5(-9.3: -1.8)	0.0048
Scheduled Castes	7.2(5.5:8.8)	3(1.7:4.2)	9.6(6.6:13)	5.8(3.4:8.3)	-3.8(-7.5:0.03)	0.0518
Other Excluded	8.0(6.8:9.2)	3.5(2.7:4.4)	8(5.8:10.3)	5.3(3.2:7.4)	-2.8(-5.7:0.19)	0.0661
All other Groups	8(7.15:8.8)	0.052(.040:0.064)	8.8(5.9:12)	4.7(2.2:7.4)	-4.1(-7.9:-.4.0)	0.0302
**Location**						
Rural	6.5(5.6:7.5)	0.03(0.024:0038)	10(8.5:12)	5·8(3.9:7.6)	-4.7(-7.3:-2.1)	0.0007
Urban	8.7(7.3:10)	0.056(0.048:0.064)	7.0(4.5:9.5)	4(1.1:6.9)	-3.0(-6.7:0.68)	0.1081
**Quintile**						
poorest	5.2(3.9:6.5)	0.025(0.016:0.033)	12.1(7.8:16)	3.1(1.3:0.049)	-9(-14:-4.4)	0.0002
2nd	0.064(0.048:0.08)	0.027(0.021:0.032)	0.095(0.070:0.12)	0.052(0.034:0.070)	-0.043(-.073: -.013)	0.0062
middle	0.074(0.050:0.098)	0.048(0.031:0.065)	0.10(.073:0.133)	0.044(0.013:0.076)	-0.059(-.100:-.017)	0.0069
4th	0 .087(0.063:0.110)	0.06(.041:0.078)	0.083(0.061:0.104)	0.039(0.014:0.064)	-0.044(-.075:-.012)	0.0076
richest	0.10(0.090:0.12)	0.064(0.049:0.079)	0.045(0.0035:0.086)	0.049(-.0068:0.105)	0.0045(-.062:0.071)	0.8937

Hospital utilisation for in-patient care

Overall, hospitalisation rates have increased in both AP and Maharashtra (Table 6) but more so in AP (5.6 per 1000 population vs 2.2), although the DID was not statistically significant. The sub group analysis presented a mixed picture. For both male and female headed households there was a greater increase in hospitalisation in AP, but this reached moderate statistical significance only for female headed households (DID=27.6, 95% CI =1.1:54.1, p-value = 0.0415). There is an increase in hospitalisations among scheduled tribes in Maharashtra and a reduction in AP (DID = -19.8, 95% CI = -37.3: -2.3, p-value = 0.0272) but the opposite trend was seen among 'other excluded' groups with an increase in AP and a reduction in MH (DID = 12.5, 95% CI= 1.2:23.9 p-value = 0.0309). In scheduled castes hospitalisations had increased in both states but more so in AP, while in the 'other' group there was a small increase in MH and a small reduction in AP. In the poorest quintile, the increase in hospitalisation was significantly greater in MH (DID= -14.4, 95%CI=-28:-0.31, p= 0.0451).

Table 6: Changes in hospitalisation (per 1000 population) in Maharashtra and AP between 2004 and 2012

Subgroups	Baseline Mean (95% CI)		Change 2004:2012 Mean (95% CI)		DID	
	Maharashtra	Andhra Pradesh	Maharashtra	Andhra Pradesh	Mean (95%CI)	p
Hospitalizations per 1000 population						
**All**	41.3(37.3:45.2)	31.5(27.8:35.3)	2.2 (-4.7:9.1)	5.6(-1.1:12.3)	3.4(-5.9 :12.7)	0.4636
					**DID (adjusted)**	
					0.7(-8.6:10.2)	0.8685

For peer review only - http://bmjopen.bmj.com/site/about/guidelines.xhtml

**Head of Household**						
Male	41.0(37.1:44.9)	31.7 (16.9:34.0)	1.9(-5.1:8.8)	4.4(-2.4:11.1)	2.5(-6.9:11.9)	0.5966
Female	51.6 (30.6:72.5)	25.5(27.7:35.7)	13.9(-7.53:35.4)	41.5 (24.6:58.4)	27.6(1.1:54.1)	0.0415
**Social Group**						
Scheduled Tribes	23.7(14.2 :33.1615)	35.5(21.9:49.1)	17.1(5.8:28.5)	-2.7(-16.95:11.5)	-19.8(-37.3: -2.3)	0.0272
Scheduled Castes	42.4(36.3:48.4)	29.5(22.6:36.5)	3.9(-7.9:15.7)	7.6(-2.6:17.7)	3.7(-11.4:18.7)	0.6268
Other Excluded	44.2(38.2:50.2)	29.9(25.5:34.3)	-1.9(-11.2 :7.3109)	10.6(3.4:17.8)	12.5(1.2:23.9)	0.0309
All other Groups	42.5(37.1:47.9)	34.9(29.0:40.9)	0.93(-7.3:9.1)	-1.1(-9.4: 7.4)	-2.0(-13.5 :9.4)	0.7235
**Location**						
Rural	36.6(32.2:41)	28.9(26.4:31.5)	9.5(3.5:15.4)	8.8(2.2:15.3)	-0.69(-9.3:7.9)	0.8725
Urban	48.2(43.7:53)	38.2(35.5:41.2)	-7.9(-14.5: -1.3)	-2.5(-12.1:7.1)	5.4(-5.8:16.6)	0.3358
**Quintile**						
poorest	31.4(25.9:36.9)	27.5(22.8:32.1)	20.7(9:32.8)	6.4(-1.7:14.4)	-14.4(-28:-0.31)	0.0451
2nd	36.5(28.3:44.7)	27.1(21.9:32.4)	8(0.5:15.4)	8.9(-.56:18.4)	0.9(-10 : 12.5)	0.8746
middle	47.8(33.6:62.1)	35.3(29.0:41.6)	-1.5(-17.9:14.8)	4.1(-.56:13.7)	5.6(-12.8:24.0)	0.5457
4th	46.9(39.9:53.9)	41.7(32.7:50.8)	-4.0(-12:3.4)	-5.1(-15.7:5.4)	-0.75(-13.3:11.9)	0.9056
richest	51.0(46:56.1)	36.5(25.8:47.2)	-13.1(-18.9: -7.1)	1.5(-19.5:22.6)	15.0(-5.8: 3.6)	0.1665

LIMITATIONS OF THE STUDY

Difference in difference estimations aimed at assessing the impacts of interventions, assume that both populations demonstrate similar characteristics prior to the start of the intervention, and that 'unobservables' follow a common trend; under such circumstances, any differences in changes observed over time between the 2 populations are attributable to the interventions[39, 40]. Despite AP and Maharashtra having broadly similar socio-economic profiles, as well as our DID analysis taking account of a number of covariates, there may have been other factors resulting in unobserved differential changes between the 2 populations and to which the results of the DID analysis may be at least partially attributable.

A second limitation could arise from the impact of other public health programmes implemented during the period 2004 to 2012. The most significant of these is the National Rural Health Mission launched in 2005, mainly to improve maternal and child health through the revitalization of rural primary care and child and maternal health services. A key assumption of our study is that the impacts of the NRHM in terms of healthcare expenditure for maternal and child health care would have been similar in both states, as this was a nationwide development. Despite the improvements in the public sector maternal and child health services sought by the NRHM, it is widely recognised that the public, including BPL families, continues to pay OOP for private health care. We have assumed that this behaviour is likely to be similar across the 2 states. Other health initiatives such as the Navsanjeevani Yojana and HMRI were unlikely to have had an impact on inpatient care or expenditure. The 108 scheme had the potential, in AP, to influence hospitalisation rates, by helping more households to visit hospitals when seriously ill. But the effect is likely to be small as the majority of even serious illnesses do not result in a 108 call for transport. The implementation of the RSBY and the scheme to make private hospital beds available for the poor in MH may have diluted the DID, although both schemes are known to have been only partially implemented.

For peer review only - http://bmjopen.bmj.com/site/about/guidelines.xhtml

Lastly, the 2004 NSSO survey which served as our baseline, was carried out between January and June 2004. Our end-line 2012 survey was carried out over a period of 3 months from June to September. The morbidity and mortality patterns recorded in different time periods may vary, and could have influenced the data.

DISCUSSION

We found that average expenditure, large expenditures and large borrowings on inpatient care had increased in both MH and AP, but the increase was consistently smaller in AP across these 3 outcome measures, and may be suggestive of Aarogyasri having a somewhat larger effect than RSBY. Similar increases in institutional deliveries across the 2 states, and low levels of utilisation of RSBY and Jeevandayee schemes in MH may further strengthen this explanation.

The increase in average OOPE on inpatient care in AP and MH reflects a trend observed nationwide[16, 41]. The Aarogyasri scheme may have contributed to the more favourable trajectory in AP both directly and indirectly, in that the scheme may have contributed to a reduction in the prices of interventions and an increase in competition among health care providers. The evaluation of the Yeshasvini scheme also found a significant reduction in the price of surgical interventions[10]. Our findings may suggest that the positive effects of Aarogyasri detected by other studies[15,16] at an early stage of the roll out of the scheme have been sustained. Automatic enrolment into the scheme, near universality of coverage and no requirement for enrollee contributions may have contributed to the significant DIDs in male headed households, scheduled castes, rural households and the poorest and middle asset quintiles. But these benefits were not demonstrated in some of the most vulnerable groups - female headed households and scheduled tribes. This is consistent with the findings of other studies[5,16] which reported that the slightly less vulnerable may benefit more from such schemes, with non-financial barriers undermining access to services for the most vulnerable socio-economic groups. The case summaries below illustrate the obstacles they face. A similar observation was reported by the evaluation of the Mexican Seguro Popular health scheme which showed that a third of the treatment-cluster households who were automatically affiliated were unaware of this fact[42].

The likely explanation of an Aarogyasri effect may be strengthened by the trend for large borrowings which has also increased over time in both states but less so in AP across all groups of analysis except the richest asset quintile. A multi-country analysis of household catastrophic health expenditure highlighted that increasing the availability of health services is critical to improving health in poor countries, but it could also raise the proportion of households facing catastrophic expenditure unless financial risk protection policies are given a high priority[43]. Borrowing of comparatively small amounts is less impactful and may be a result of improved access to financial markets and also supports consumption smoothening. During recent decades, AP has in particular witnessed a significant rise in microfinance institutions and debt due to high levels of interest levied by the more recent entrants to this market[44, 45]. Nevertheless our results suggest that increases in large borrowings associated with inpatient health care were smaller in AP. Strongly significant DIDs in scheduled tribes, rural households and the poorest and second asset quintiles and moderately significant DIDs in female headed households, scheduled castes, and other excluded social groups may also point to Aarogyasri beginning to offer greater access to health care when families are faced with serious illness.

In terms of large OOPE, a significant DID was found only for the total households, but the direction of all DIDs except for the second asset quintile was the same as for average expenditure on inpatient care and large borrowings, i.e., in favour of AP. It is perfectly possible that the non-significant differences for the majority of sub groups are due at least in part to the RSBY, the availability of private sector hospital beds for the poor and the Jeevandayee schemes reducing large OOPE in MH,

For peer review only - http://bmjopen.bmj.com/site/about/guidelines.xhtml

and that without these schemes the expenditure would have been greater. In AP, 70 percent of survey households reported that they were covered by the Aarogyasri scheme, but only 25 percent of these 'covered' households were aware that the benefit package was limited. It is also possible therefore that in AP some families seek hospital care assuming that the Aarogyasri scheme provides comprehensive cover, but are faced with large expenditures when their treatments fall outside the limits.

The mixed picture in relation to the increasing rate of hospitalisations in both states may suggest that the Aarogyasri and in particular, the RSBY scheme which covers common hospital procedures are addressing a large hitherto unmet need for inpatient care. The 108 scheme in AP may be an additional albeit smaller contributor. It may also be explained by a supplier-induced demand. An assessment of health provider behaviour and governance is an important strand of the evaluation of health financing schemes[46], but was outside the remit of our study. We would however strongly recommend an impact evaluation focusing on health care supply to complement our evidence. The greater increase in hospitalisation in AP, in female-headed households and 'other excluded groups', which are two of the most vulnerable population groups, is encouraging. But the reduction in hospitalisations among scheduled tribes and the poorest asset quintile in AP are of concern and suggest that if the poor are to secure the benefits appropriated by the near poor or more often by the rich[47], the provision of more comprehensive health schemes is essential, which combines the tertiary and secondary care focus of Aarogyasri and the secondary care benefits of RSBY with attention paid to minimise barriers such as the widespread influence of illiteracy and lack of awareness, which limit access to even schemes such as Aarogyasri that are apparently highly inclusive and non-discriminatory, as well as distance to facilities. Our case summaries illustrate this. Furthermore, health financing reforms such as the Jamkesmas[48] in Indonesia and the Seguro Popular in Mexico[42], both countries similar to India in terms of population and growing economies, include outpatient and inpatient care, and curative as well as preventive services, suggesting that a more comprehensive service is possible to implement and worthy of consideration.

In summary, health innovations in AP had a greater beneficial effect on hospital inpatient care-related expenditures than innovations in MH. The Aarogyasri scheme is likely to have contributed to these impacts in AP, at least in part. But in both states, OOPE increased over time. Hospitalisations also increased, and while it is generally assumed that in developing countries this is likely to address genuine need, its impact on health status is not known.

IMPLICATIONS FOR POLICY AND PRACTICE

Despite these uncertainties, Aarogyasri is perceived, with some justification across India as a successful scheme, and is being rapidly replicated across the states. Since July 2012, MH too has joined the list of states offering an Aarogyasri-like scheme, the Rajiv Gandhi Jeevandayee Arogya Yojana[49]. This study has highlighted that such schemes may result in some positive outcomes. Although the study was not designed to elicit the specific features of the scheme to which any comparatively greater benefits may be attributable, and which deserve to be replicated in other states, evidence from systematic reviews[3] points to the need for schemes to be more 'comprehensively' designed to maximise their positive impact. Aarogyasri's design in terms of its aims to address both financial and non-financial barriers - being fully state funded, the systematic administrative implementation of Aarogyasri across the state so that it is now almost universal, automatic enrolment, allowing access to the scheme via a ration card which most poor families own, the wide spectrum of treatments offered, the large number of health care providers empanelled - is perhaps responsible as a 'comprehensive' package, for the greater impact.

But the study also suggests that improved access to health care and the reduction of the overall burden of OOPE especially in the most vulnerable sections of the population[50] are likely to require

For peer review only - http://bmjopen.bmj.com/site/about/guidelines.xhtml

additional interventions that address gaps in the availability of care and provide patients appropriate pathways that support their journey from a strong and comprehensive primary care service where they may be informed of their entitlements and investigated for their initial symptoms to appropriate hospitals for the treatment of serious illness. Besides, inpatient care is only consumed by a small proportion of households in a year[51], and this is especially true of tertiary care, while many more will seek outpatient services and referral to inpatient care, should this be required. Others have strongly recommended the strengthening of the primary care base as an essential means to universal health coverage and this study confirms their view[41]. Key implications for AP are to explore how best the most advantageous features of Aarogyasri can be extended to include both secondary and primary care, while those for MH may be to build on and unify its menu of currently available schemes to create an evidence-based comprehensive health delivery system. These conclusions may be applicable to other states with similar health financing schemes.

The design of health financing systems as well as their evaluations is complex and challenging, as the mountain of available evidence suggests[41, 42, 51, 52]. Even the ground-breaking Seguro Popular health insurance programme of Mexico which used a cluster-randomised trial design with strong government support demonstrated some but not all anticipated outcomes, contrary to expectations[42], and a key recommendation was that continued assessment of the programme was needed. Furthermore, differences in data and methodology may result in even very well designed evaluations of the same programme producing contrasting findings[52]. Our evaluation is the first to use a quasi-experimental methodology - the best possible in the hierarchy of evaluation methodologies, when a randomised control trial is not achievable - to evaluate the health financing reforms in 2 large states of India. Despite that, the study has limitations which we have acknowledged. For example, the evaluation was not designed to assess provider behaviours. Many other pertinent questions such as the impact of the schemes on the overall economy of health care cannot be answered by a single evaluation. But these are recognised problems which can only be addressed through continuous assessments, as other evaluations have shown. Our study has nevertheless produced sufficient insights to enable policy leaders to improve programme effectiveness and, importantly, to undertake further assessment. Our household survey data provide a valuable baseline for future monitoring in both states.

This study needs to be followed up with further and repeated evaluations as AP's and MH's schemes evolve; to assess the impacts of re-design and to help health policy leaders achieve their aspiration of universal access to good quality health care.

Three faces of the Aarogyasri Scheme

The beneficiary:

Lakshmamma, a 65 year old widow, is from a tribal background. She is an unskilled labourer, supporting a family of 5, with a monthly income of INR 4000 (USD 74). She was referred to a municipal hospital with chest pain and underwent heart surgery. The hospital which is a part of the Aarogyasri network provided free care and her total out of pocket expenses amounted to INR 850 (16 USD) for initial transport. In addition to the surgery, she received free food, money for transport home and follow up medicines. She is very satisfied with the service she received.

The excluded:

For peer review only - http://bmjopen.bmj.com/site/about/guidelines.xhtml

Ramulamma, a mother has a BPL card, but her daughter Lakshmi does not, as she was abandoned by her husband who is a government employee and entitled to free health care. The mother was unable to secure free care using her BPL card for her seriously ill daughter who paid out-of-pocket at a private facility where she was offered a hysterectomy for the relief of her gynaecological symptoms. Lakshmi's health care costs were met by the family selling a number of household assets and Lakshmi's daughter discontinuing her education to take up paid work. Lakshmi is severely depressed and does not speak to anyone.

The uninformed:

Krishnamma 43 years old, had severe stomach pains one night. Although the family had a BPL card, Ramulu her husband and Srinivas her son rushed her to a private hospital nearby, which was not part of the Aarogyasri network. They were unaware of how to access the Aarogyasri scheme hospitals which were further away from home, and the local primary health services being inadequate, Krishnamma had not had her initial symptoms investigated. The treatment was funded through a loan from a private moneylender. On discharge, she has not attended follow up, as the family cannot afford transport or medicines.

For peer review only

ACKNOWLEDGEMENTS:

The study was funded by International Development and Research Centre, Canada, Wellcome Trust, the UK Department for International Development, and Rockefeller Foundation. The World Bank supported Adam Wagstaff's contribution to the study. We gratefully acknowledge the financial support of these organisations, as well as the enthusiastic encouragement we have received from them throughout the study. Our thanks also go to Bhimasankaram Pochiraju, Sundaresh Peri, C Ravi, and Rahul Ahluwalia for their advice and guidance at various stages of the study design and data analysis, and to colleagues at the University of East London and Administrative Staff College of India Hyderabad for their administrative support. The authors also thank P Suryanarayana, CM Reddy, D Chakrapani and K Pandu Ranga Reddy and AY Jadhav and his colleagues for their contribution to the training of the survey teams, verification of the survey and data collection and acknowledge the IMRB International Social Research Institute team's support in carrying out the survey.

We are grateful to Somil Nagpal and Joseph Kutzin for commenting on the report and helping us to improve it, to Sujatha Rao for her encouragement and valuable suggestions and ideas throughout the course of the study and to Jay Bagaria for her help during the early stage of the development of the proposal. Finally we thank PV Ramesh without whose constant support, guidance and encouragement, this study would not have been possible.

FUNDED BY : Wellcome Trust, IDRC, Rockefeller Foundation, DFID, World Bank.

CONTRIBUTORS' STATEMENT

MR conceived and designed the study, applied for funding, and was responsible for the supervision and management of the study as well as the dissemination of its results. She is also the guarantor of the study. SB shared responsibility for the conception of the study, applications for funding, study design and data collation and analysis, contributed to the questionnaire design and commented on drafts of the report. PS contributed to the conception of the study and study design, led the questionnaire design and survey implementation, including training of survey staff, monitoring survey progress and data collation and verification, commented on drafts of the report and helped prepare the references. AK undertook the data collation, verification and analysis, assisted with the survey and questionnaire design and survey implementation and prepared the tables for the report. AS led the literature review, assisted with the study and questionnaire design, survey implementation and preparation and analysis of baseline data, and commented on drafts of the report. MK helped with the data analysis. AW devised the methodology for the estimation of the programme impacts, advised during the data-collection and data-preparation stages, wrote and implemented the computer code for the model estimation, helped to oversee the production of the results, and contributed text to the report. GN provided technical advice on accounting for the complex survey structure in the analysis, developed a STATA equation, helped to compute an asset index, advised on the output tables, verified the analysis and commented on drafts of the report. AR helped develop a conceptual framework for the evaluation, advised on funding proposals, the study design, analytical methodology and presentation of results and contributed text to the report. MR wrote the first draft of the paper and its redrafts in accordance with the comments of all other authors and reviewers.

For peer review only

CONFLICT OF INTEREST

All authors have completed the ICMJE forms for disclosure of potential conflicts of interest (available on request from the corresponding author) and declare that 1) MR, AK, PS, AS, and SB have support from the Rockefeller Foundation, Wellcome Trust, International Development Research Centre, Canada and Department for International Development, UK, 2) the authors have no relationships with the funders that might have an interest in the submitted work in the previous 3 years, 3) the authors' spouses, partners, or children have no financial relationships that may be relevant to the submitted work, and 4) the authors have no non-financial interests that may be relevant to the submitted work.

DATA SHARING STATEMENT: The 2004 National Sample Survey Organisation of India household survey questionnaire and data are available to the public. The 2012 household survey questionnaire, and the full anonymised household survey dataset will be available with open access as soon as the data analyses and submissions for publication are completed.

ROLE OF THE FUNDING SOURCES: The external funding sources had no role in study design, data collection, analysis, interpretation or reporting, or in submission decision.

ROLE OF THE STUDY SPONSOR: The study was carried out in response to the formal invitation of the Government of Andhra Pradesh which supported our applications for funding (by providing formal letters of support). The Government did not provide any funding, and had no role in study design, data collection, analysis, interpretation or reporting, or in submission decision.

STATEMENT OF INDEPENDENCE OF RESEARCHERS: The authors can confirm that they worked independently from the Funders and Government of Andhra Pradesh who had no role in any aspect of the research

ETHICAL APPROVAL: given by the research ethics committee of the Administrative Staff College of India, Hyderabad, which hosted the study. Household survey questionnaires include signed consent by the head of the household or another adult representative of the household.

COPYRIGHT STATEMENT: Mala Rao, the Corresponding Author has the right to grant on behalf of all authors and does grant on behalf of all authors, an exclusive licence on a worldwide basis to the BMJ Publishing Group Ltd to permit this article (if accepted) to be published in BMJ editions and any other BMJPGL products and sublicences such use and exploit all subsidiary rights, as set out in your licence.

STROBE CHECKLIST FOR CROSS SECTIONAL STUDIES: We can confirm that all 22 items in the STROBE checklist are included in the paper.

For peer review only - http://bmjopen.bmj.com/site/about/guidelines.xhtml

References

[1] Resolution WHA58.33. Sustainable health financing, universal coverage and social health insurance. In: *Fifty-eighth World Health Assembly, Geneva, 16–25 May 2005.* Geneva, World Health Organization, 2005 (http://apps.who.int/gb/ebwha/pdf_files/WHA58/WHA58_33-en.pdf, accessed 26th March 2013).
[2] World Health Organization. Health Systems Financing Path to Universal Coverage. Geneva: World Health Organization. 2010. Available from: World Health Organization Library Information Services.
[3] Acharya A, Vellakkal S, Taylor F, et al. Impact of national health insurance for the poor and the informal sector in low and middle income countries : a systematic review. London: EPPICentre, Social Science Research Unit, Institute of Education, University of London. 2012. Available from: http://www.dfid.gov.uk/r4d/PDF/Outputs/SystematicReviews/Health-insurance-2012Acharya-report.pdf
[4] Giedion, U., B. Y. Diaz . A Review of the evidence. In: The Impact of Health Insurance in Low-and Middle-Income Countries. M. L. Escobar, C. Griffin and R. P. Shaw (ed). Washington DC, Brookings Inst Press 2011.

[5] Giedion, U, Alfonso, E , Diaz. *The impact of universal coverage schemes in the developing world : a review of the existing evidence*. Universal Health Coverage (UNICO) studies series ; no. 25. Washington D.C. : The World Bank. 2013
[6] National Commission on Macroeconomics and Health. Report of the National Commission on Macroeconomics and Health. New Delhi: Ministry of Health and Family Welfare of India. 2005. Available from: Ministry of Health and Family Welfare
[7] Eleventh Five Year Plan (2007–2012). Vol 2: Social Sector. Government of India Planning Commission. October, 2008. http://planningcommission.nic.in/plans/planrel/fiveyr/11th/11_v2/11th_vol2.pdf, (accessed Mar 26 2013).
[8] Faster, Sustainable and More Inclusive Growth. An Approach to the Twelfth Five Year Plan (2012-17). Government of India Planning Commission. October, 2011. http://planningcommission.nic.in/plans/planrel/12appdrft/appraoch_12plan.pdf, accessed 26th March 2013
[9] La Forgia G, Nagpal S. Government-Sponsored Health Insurance in India. *Are You Covered?* World Bank Washington DC 2012. doi:10.1596/978-0-8213-9618-6.
[10] Agarwal A. "Impact evaluation of India's 'Yeshasvini' community based health insurance programme. *Health Economics* 2010; 19: 5-35
[11] See The scheme website at https://www.aarogyasri.org/ASRI/index.jsp, accessed 26th March 2013.
[12] Scheme status. http://www.rsby.gov.in/statewise.aspx?state=35 (accessed Apr 30 2013)
[13] Mahal A. Learning and getting better: Rigorous evaluation of health policy in India. National Medical Journal of India. 2011. 325-27.
[14] Aarogyasri Health Care Trust Annual Report 2011-2012. https://www.aarogyasri.org/ASRI/EXT_IMAGES/documents/Annual_Report_201011.pdf (accessed Mar 26 2013)
[15] Rao M, Ramachandra S, Bandyopadhyay S, et al. Addressing healthcare needs of people living below the poverty line: a rapid assessment of the Andhra Pradesh Health Insurance Scheme. National Medical Journal of India. 2011. 24(6):335-41.
[16] Fan V, Karan A, Mahal A. "State Health Insurance and Out of-Pocket Health Expenditures in Andhra Pradesh, India." CGD Working Paper 298.Washington, D.C.: Center for Global Development. 2012 http://www.cgdev.org/content/publications/detail/1426275 (accessed Mar 15 2013)
[17] Palacios R, Das J, Sun C eds. India's health insurance scheme for the poor: Evidence from the early experience of Rashtriya Swasthya Bima Yojana. New Delhi: Center for Policy Research; 2011
[18] Rashtriya Swasthya Bima Yojana overview and scheme details. http://www.rsby.gov.in (accessed Apr 30 2013)
[19] Rathi P. http://www.priorities2012.com/documents/3d-4-patreek.pdf
[20] NRHM Mission Document.http://www.nird.org.in/brgf/doc/Rural%20HealthMission_Document.pdf (accessed Apr 30 2013)
[21] State HMIS data analysis, April'10-March'11 Andhra Pradesh, prepared by NHSRC.http://cfw.ap.nic.in/nrhm/pdf/AP_Analysis_Apr2010-Mar2011.pdf (accessed May 2 2013)
[22] NRHM achievements in Maharashtra. http://www.nrhm.maharashtra.gov.in/achievements.htm (accessed May 2 2013)
[23] Health information helpline.http://www.hmri.in/oursolutions-healthinformation.html (accessed Apr 30 2013)

For peer review only - http://bmjopen.bmj.com/site/about/guidelines.xhtml

[24] About the project/scheme. http://www.maha-arogya.gov.in/projectandschemes/Jeevandaiaarogya/default.htm (accessed Apr 30 2013)

[25] Jeevandayee scheme performance: Information received from Rajiv Gandhi Jeevandayee Arogya Yojana Society Govt of Maharashtra on May 17 2013

[26] Projects and schemes. http://maha-arogya.gov.in/projectandschemes/itdpnavsanjivani%5Cdefault.htm (accessed Apr 30 2013)

[27] Mallepeddi R , Pernefeldt H, Bergkvist S . Andhra Pradesh health sector reform, A narrative case study, Technical paper No.7 for The Rockefeller Foundation–Sponsored Initiative on the Role of the Private Sector in Health Systems in Developing Countries. 2009 ; pp 42-48

[28]EMRI. http://www.emri.in/states.html (accessed Apr 30 2013)

[29] Developing and evaluating complex interventions: new guidance. Medical Research Council. London 2008.

[30] Cesar G V, Jean-Pierre H, Jennifer B. Evidence-Based Public Health: Moving Beyond Randomized Trials. Am J Public Health. 2004 March; 94(3): 400–405.

[31] Presentation on Annual Plan 2012-13 and Five Year Plan 2012-17. http://planningcommission.nic.in/plans/stateplan/Presentations12_13/maharashtra1213.pdf (accessed on mar 25 2013)

[32] National Sample Survey Organization. Morbidity, Healthcare and condition of the Aged. New Delhi: Ministry of Statistics and Programme Implementation. 2004.

[33] National Sample Survey Organization. Household Consumer Expenditure 66th round . New Delhi: Ministry of Statistics and Programme Implementation; 2009-10

[34] National Sample Survey Organization. Household Consumer Expenditure in India. New Delhi: Ministry of Statistics and Programme Implementation; 2005.

[35] Booysen F, Van der Berg S & Burger R. Using an Asset Index to Assess Trends in Poverty in Seven Sub-Saharan African Countries. World Development. 2008; 36(6): 1113–1130

36 Vyas S. & Kumarnayake L. Constructing socio-economic status indices: How to use principal components analysis. Health Policy and planning. 2006; 459-468

[37] O'Donnell O, Van Doorslaer E, Wagstaff, et al. Analyzing Health Equity Using Household Survey Data: A Guide to Techniques and Their Implementation. Washington DC: World Bank Institute. Chapter 6, Measurement of Living Standards. http://siteresources.worldbank.org/INTPAH/Resources/Publications/459843-1195594469249/HealthEquityFINAL.pdf (accessed May 9 2013)

[38] Ministry of Finance. Economic Survey 2011-12. New Delhi: Government of India. 2012. Available from: http://indiabudget.nic.in/

[39] Poverty Reduction and Social Development Department. A User's Guide to Poverty and Social Impact Analysis. Washington: World Bank;2003

[40] Gertler P, Martinez S, Premand P, et al. Impact Evaluation in Practice. Washington: World Bank; 2011.

[41] Selvaraj S, Karan A. Why Publicly-Financed Health Insurance Schemes Are Ineffective in Providing Financial Risk Protection. Economic and Political Weekly 2012 March 17; XLVII(11):60-68

[42] King G, Gakidou E, Imai K, et al. Public policy for the poor? A randomised assessment of the Mexican universal health programme. Lancet. 2009 Apr 8; DOI:10.1016/S0140-6736(09)60239-7.

[43] Xu K, Evans DB, Kawabata K,et al. Household catastrophic health expenditure: a multicountry analysis. Lancet. 2003 Jul 12;362(9378):111-7.

[44] Naga Sridhar G. Microfinance institutions: Moving beyond AP crisis. The Business Line [Newspaper Online]. 2012 Dec 29. Available from: http://www.thehindubusinessline.com/companies/microfinance-institutions-moving-beyond-ap-crisis/article4253239.ece

[45] Naga Sridhar G. Life after microfinance in AP. The Business Line [Newspaper Online]. 2013 Jan 4. Available from: http://www.thehindubusinessline.com/opinion/life-after-microfinance-in-ap/article4273183.ece

[46] Prasad N P, Raghavendra P. Healthcare models in the era of medical neoliberalism. A study of Aarogyasri in Andhra Pradesh. Economic and Political Weekly 2012 Oct 27;XLVII(43):118-126

[47] Health Systems 20-20. An Evaluation of the Effects of the National Health Insurance Scheme in Ghana. Bethesda: Health Systems 20-20. 2009.

[48] http://www.jointlearningnetwork.org/programs/compare/benefits/142%2C16

[49] Rajiv Jeevandayi scheme details. http://www.jeevandayee.gov.in/RGJAY/FrontServlet?requestType=CommonRH&actionVal=RightFrame&page=undefined%3E%3E%3Cb%3ERGJAY%3C/b%3E&pageName=RGJAY&mainMenu=About&subMenu=RGJAY (accessed on Apr 30 2013)

For peer review only - http://bmjopen.bmj.com/site/about/guidelines.xhtml

[50] Tangcharoensathien V, Swasdiworn W, Jongudomsuk P et al . Universal Coverage Scheme in Thailand: Equity Outcomes and Future Agendas to Meet Challenges. Geneva: World Health Organization. 2010.
[51] Dilip TR. On Publicy-Financed Health Insurance Schemes. Is the analysis premature? Economic and Political Weekly. 2012 May 5; XLVII(1)8:79-80.
[52] Vallakal S, Ebrahim S. Publicly-Financed Health Insurance Schemes. Concerns about Impact Assessment. Economic and Political Weekly. 2013 Jan 5; XLVIII(1);24-27.

For peer review only

For peer review only - http://bmjopen.bmj.com/site/about/guidelines.xhtml

TITLE PAGE

TITLE:

Trends in addressing inequalities in access to hospital care in Andhra Pradesh and Maharashtra states of India: A tale of two Indian states

STUDY DESIGN: A cross-sectional difference in difference study with parallel control.

AUTHOR NAMES, ADDRESSES AND POSITIONS:

1.Mala Rao. Professor of International Health, Institute for Health and Human Development, University of East London, UK & Honorary Adviser, Administrative Staff College of India, Hyderabad

2. Anuradha Katyal, Consultant, ACCESS Health International, Hyderabad, India

3.Prabal V Singh, Director Research, ACCESS Health International, Hyderabad, India

4.Amit Samarth, Head, Strategy and Planning, SughaVazhvu Healthcare, Thanjavur, India

5.Sofi Bergkvist, Managing Director, ACCESS Health International, Hyderabad, India

6.Manjusha Kancharla, Research Associate, Indian School of Business, Hyderabad India

7.Adam Wagstaff, Research Manager, Human Development & Public Services team, Development Research Group (DECRG), The World Bank, Washington DC, USA

8.Gopalakrishnan Netuveli. Professor of Public Health, Institute for Health and Human Development, University of East London & ESRC International Centre for Life Course Studies in Society and Health, University College London, UK

9.Adrian Renton. Director, Institute for Health and Human Development, University of East London, UK

All authors had full access to all the data (including statistical reports and tables) and can take responsibility for the integrity of the data and the accuracy of the data analysis

Corresponding author and guarantor of the data and information: Mala Rao (best email address: m.rao@uel.ac.uk).

M Rao affirms that the manuscript is an honest, accurate and transparent account of the study being reported and no important aspects of the study have been omitted.

FUNDED BY : Wellcome Trust, IDRC, Rockefeller Foundation, DFID, World Bank.

ROLE OF THE FUNDING SOURCES: The external funding sources had no role in study design, data collection, analysis, interpretation or reporting, or in submission decision.

ROLE OF THE STUDY SPONSOR: The study was carried out in response to the formal invitation of the Government of Andhra Pradesh which supported our applications for funding (by providing formal

For peer review only - http://bmjopen.bmj.com/site/about/guidelines.xhtml

letters of support). The Government did not provide any funding, and had no role in study design, data collection, analysis, interpretation or reporting, or in submission decision.

STATEMENT OF INDEPENDENCE OF RESEARCHERS: The authors can confirm that they worked independently from the Funders and Government of Andhra Pradesh who had no role in any aspect of the research

CONFLICT OF INTEREST

All authors have completed the ICMJE forms for disclosure of potential conflicts of interest (available on request from the corresponding author) and declare that 1) MR, AK, PS, AS, and SB have support from the Rockefeller Foundation, Wellcome Trust, International Development Research Centre, Canada and Department for International Development, UK, 2) the authors have no relationships with the funders that might have an interest in the submitted work in the previous 3 years, 3) the authors' spouses, partners, or children have no financial relationships that may be relevant to the submitted work, and 4) the authors have no non-financial interests that may be relevant to the submitted work.

ETHICAL APPROVAL: given by the research ethics committee of the Administrative Staff College of India, Hyderabad, which hosted the study. Household survey questionnaires include signed consent by the head of the household or another adult representative of the household.

DATA SHARING STATEMENT: The 2004 National Sample Survey Organisation of India household survey questionnaire and data are available to the public. The 2012 household survey questionnaire, and the full anonymised household survey dataset will be available with open access as soon as the data analyses and submissions for publication are completed.

COPYRIGHT STATEMENT: Mala Rao, the Corresponding Author has the right to grant on behalf of all authors and does grant on behalf of all authors, an exclusive licence on a worldwide basis to the BMJ Publishing Group Ltd to permit this article (if accepted) to be published in BMJ editions and any other BMJPGL products and sublicences such use and exploit all subsidiary rights, as set out in your licence.

STROBE CHECKLIST FOR CROSS SECTIONAL STUDIES: We can confirm that all 22 items in the STROBE checklist are included in the paper.

CONTRIBUTORS' STATEMENT

MR conceived and designed the study, applied for funding, and was responsible for the supervision and management of the study as well as the dissemination of its results. She is also the guarantor of the study. SB shared responsibility for the conception of the study, applications for funding, study design and data collation and analysis, contributed to the questionnaire design and commented on drafts of the report. PS contributed to the conception of the study and study design, led the questionnaire design and survey implementation, including training of survey staff, monitoring survey progress and data collation and verification, commented on drafts of the report and helped prepare the references. AK undertook the data collation, verification and analysis, assisted with the survey and questionnaire design and survey implementation and prepared the tables for the report.

For peer review only

AS led the literature review, assisted with the study and questionnaire design, survey implementation and preparation and analysis of baseline data, and commented on drafts of the report. MK helped with the data analysis. AW devised the methodology for the estimation of the programme impacts, advised during the data-collection and data-preparation stages, wrote and implemented the computer code for the model estimation, helped to oversee the production of the results, and contributed text to the report. GN provided technical advice on accounting for the complex survey structure in the analysis, developed a STATA equation, helped to compute an asset index, advised on the output tables, verified the analysis and commented on drafts of the report. AR helped develop a conceptual framework for the evaluation, advised on funding proposals, the study design, analytical methodology and presentation of results and contributed text to the report. MR wrote the first draft of the paper and its redrafts in accordance with the comments of all other authors and reviewers.

For peer review only

For peer review only - http://bmjopen.bmj.com/site/about/guidelines.xhtml

ABSTRACT. WORD COUNT: ~~294~~298

OBJECTIVES

To compare the effects of ~~health innovations~~the Rajiv Aarogyasri Health Insurance Scheme of Andhra Pradesh (AP) with health financing innovations including the RSBY in Maharashtra (MH) over time on access to and out of pocket expenditure (OOPE) on hospital inpatient care. ~~in Andhra Pradesh (AP) and Maharashtra (MH) states of India~~

STUDY DESIGN

A cross-sectional difference in difference (DID) study with parallel control.

SETTING

National Sample Survey Organisation of India (NSSO) urban and rural 'first stratum units', 863 in AP and 1008 in MH.

METHODS

We used two cross-sectional surveys: as a baseline, the data ~~for Maharashtra and Andhra Pradesh~~ ~~Two data sets were used. The~~ from the NSSO 2004 survey ~~data~~ collected before the AArogyasri and RSBY schemes were launched and as post-intervention, ~~served as the baseline and~~ a ~~cross-sectional~~ survey ~~in the two states~~ using the same NSSO methodology ~~as NSSO survey~~ ~~survey we~~ conducted in 2012.~~, using NSSO methodology provided post-intervention information.~~

PARTICIPANTS

8623 households in AP and 10073 in MH

MAIN OUTCOME MEASURES

Average OOPE, large OOPE and large borrowing per household per year for inpatient care, hospitalisation rate per 1000 population per year.

RESULTS

Average expenditure, large expenditures and large borrowings on inpatient care had increased in both MH and AP, but the increase was smaller in AP across these 3 outcome measures. DIDs for average expenditure and large borrowings were significant and in favour of AP for the rural and the poorest households. Hospitalisation rates also increased in both states but more so in AP ~~(5.6 per 1000 population vs 2.2)~~, although the DID was not significant and the sub group analysis presented a mixed picture.

CONCLUSIONS

Health innovations in A~~ndhra~~ P~~radesh~~ had a greater beneficial effect on ~~hospital~~ inpatient care-related expenditures than innovations in MH~~aharashtra~~. The ~~Rajiv~~ Aarogyasri ~~Community Health Insurance~~ scheme is likely to have contributed to these impacts in AP~~ndhra Pradesh~~ at least in part. However, out of pocket expenditure increased in both states over time. Schemes such as the Aarogyasri and RSBY may result in some positive outcomes but, ~~equity of access to health care and the reduction of the overall burden of out of pocket expenditure especially in the most vulnerable sections of the population are likely to require~~ additional interventions may be required, to improve access to care for the most vulnerable sections of the population ~~that address gaps in the availability of and access to care~~.

For peer review only

For peer review only - http://bmjopen.bmj.com/site/about/guidelines.xhtml

ARTICLE SUMMARY

STRENGTHS AND LIMITATIONS OF THE STUDY

- This study uses a quasi-experimental design to compare changes between the Indian states of Andhra Pradesh and Maharashtra, in hospital inpatient care related expenditures and behaviours before and after the rollout of the Aarogyasri and RSBY schemes
- The study based on a survey of 18696 households has shown that health innovations in Andhra Pradesh had a greater beneficial effect on hospital inpatient care-related expenditures and access than innovations in Maharashtra. The Aarogyasri scheme is likely to have contributed to these impacts in Andhra Pradesh.
- The study also highlights the implications of the findings for policy and practice and additional interventions necessary to address gaps in the availability of and access to care.
- The study is only able to compare the effects of health innovations over time across the 2 states but does not allow the drawing of inferences on the impacts of individual initiatives.
- The study uses the difference in difference methodology and its findings may have been affected by unobservable differential changes between the 2 states.

For peer review only - http://bmjopen.bmj.com/site/about/guidelines.xhtml

ACKNOWLEDGEMENTS:

The study was funded by International Development and Research Centre, Canada, Wellcome Trust, the UK Department for International Development, and Rockefeller Foundation. The World Bank supported Adam Wagstaff's contribution to the study. We gratefully acknowledge the financial support of these organisations, as well as the enthusiastic encouragement we have received from them throughout the study. Our thanks also go to Bhimasankaram Pochiraju, Sundaresh Peri, C Ravi, and Rahul Ahluwalia for their advice and guidance at various stages of the study design and data analysis, and to colleagues at the University of East London and Administrative Staff College of India Hyderabad for their administrative support. The authors also thank P Suryanarayana, CM Reddy, D Chakrapani and K Pandu Ranga Reddy and AY Jadhav and his colleagues for their contribution to the training of the survey teams, verification of the survey and data collection and acknowledge the IMRB International Social Research Institute team's support in carrying out the survey.

We are grateful to Somil Nagpal and Joseph Kutzin for commenting on the report and helping us to improve it, to Sujatha Rao for her encouragement and valuable suggestions and ideas throughout the course of the study and to Jay Bagaria for her help during the early stage of the development of the proposal. Finally we thank PV Ramesh without whose constant support, guidance and encouragement, this study would not have been possible.

For peer review only

INTRODUCTION

In 2005, member states of the World Health Organization (WHO) committed to develop their health financing systems to deliver universal coverage (UC); that is that all people have access to health services and do not suffer financial hardship paying for them[1]. WHO's 2010 World Health Report[2] recommended *inter alia* that countries reduce reliance on direct payments, and improve equity of access, including through the introduction of prepayment schemes. However, one recent systematic review of the impact of national health insurance schemes in low and middle income countries[3] found only weak evidence of increased use of healthcare and reduced out-of-pocket expenses, with the poorer benefitting less, whilst others[4,5] concluded that health insurance improved health care access and use, as well as financial protection in most cases but had no conclusive impact on health status. Both highlighted the need for more rigorous assessments of such schemes.

India has one of the highest levels of out-of-pocket health expenditure (more than 80% of private health expenditure)[6]. The aim of our study was to explore how recently introduced health financing initiatives have affected access to and out-of-pocket expenditure (OOPE) on inpatient hospital care in the Indian states of Andhra Pradesh (AP) and Maharashtra (MH).

BACKGROUND

In its *Eleventh Five Year Plan (2007-12)*[7], Government of India sought to increase Public Expenditure on Health and to strengthen investment in rural health infrastructure through the National Rural Health Mission. Its *Twelfth Five Year Plan (2012-17)*[8]*,* reflected the recommendation of the Planning Commission's High Level Expert Group for general taxation to be the principal source of health care financing. It proposed the development of government-funded health insurance schemes, building on the evidence from experimental schemes being introduced across many States. In India, health is primarily a state rather than national responsibility[6]. Whilst it is recognised that political will and good governance are essential, both the political mobilization of funds for health schemes and the effectiveness and efficiency of funded schemes will be enhanced by robust evidence which documents as to whether schemes achieve objectives, what works well and the main challenges they face.

In India, evaluation is not routine, even of large costly public health care programmes. Nevertheless, some assessments have been carried out, for example of the Yeshasvini Co-operative Farmers Health Care Scheme of Karnataka, the longest running state-supported health insurance scheme for the informal sector in India[9], with promising results in terms of increased utilization of and reduced borrowing for health care services[10]. However, these early models covered small populations and offered limited benefits, so that the policy implications of conclusions drawn from even the best evaluations were unclear.

This scenario has changed during the past 5 years, with the launch of 2 schemes, the Rajiv Aarogyasri[11] Community Health Insurance Scheme (Aarogyasri) of Andhra Pradesh (AP) and Rashtriya Swasthya Bima Yojana (RSBY) currently offered in 30 states and union territories of India[12] including AP's neighbouring state of Maharashtra (MH). Their scale in terms of population coverage and range of treatments offered, significantly enhances their potential to inform India's road map towards universal health coverage. Both schemes belong to the new generation of publicly funded government-sponsored health insurance schemes, principally aimed at providing financial protection to the poor against catastrophic health shocks, which, for these schemes, the Government has defined as inpatient hospital care[9]. Both schemes have been subject to some assessment in their early phases, but a recent editorial[13] highlighted the necessity of further and repeated evaluations to enhance the credibility and accountability of existing schemes and to identify those which deserve scale-up. The Aarogyasri and RSBY schemes and the other recent health sector innovations in AP and

For peer review only - http://bmjopen.bmj.com/site/about/guidelines.xhtml

Maharashtra which form the major backdrop and complex health care architecture against which these have been launched, and which may have contributed to the changes in outcomes we have explored in our study, are described below.

The Rajiv Aarogyasri Health Insurance Scheme of AP

In 2007 AP launched a pioneering new state-wide fully state-funded health insurance scheme, the Rajiv Aarogyasri Community Health Insurance Scheme (Aarogyasri)[11] to provide treatment for serious and life-threatening illnesses. The specific objectives include: to improve access of poor families to quality ~~'tertiary'~~ medical care (meaning low-frequency, high cost specialist care) and treatment of identified diseases requiring hospitalisation through an identified network of health care providers, to provide financial cover for catastrophic illnesses which have the potential to wipe out life time savings of poor families and to provide 'universal coverage to the urban and the rural poor in the state[14] albeit for the conditions covered in the benefits package. All families with a 'below poverty line' (BPL) ration card, i.e. those on an annual income below USD 1384 (INR 75,000) in urban areas and USD 1107 (INR 60,000) in rural areas, and including individuals with pre-existing medical conditions are *automatically* enrolled and the scheme was estimated to cover approximately 20.4 million poor and lower middle class families, comprising about 85 percent of the state's population in 2009[9]. Enrollees make no contribution, the annual benefit is a maximum of USD 4,500 (INR 200,000) per family per year and there is no limit on the size of the family[14]. A total of 942 medical and surgical procedures across 31 clinical specialties [14] are provided and the benefits include all inpatient costs - associated investigations, food, transport and medicines for 10 days following discharge. One year follow-up packages including consultation, medicines, and diagnostics are also available for 125 procedures requiring longer periods of follow up[9] . Aarogyasri has unique features including *Aarogyamithras* (health system navigators), outreach *health camps* delivered by participating hospitals to educate, screen and case-find and a state-of-the-art information technology-based management system. At the time of this study, 353 public and private sector hospitals were 'empanelled' to provide services to Aarogyasri beneficiaries.

In 2009, a descriptive study of *Aarogyasri*, based on an analysis of claims data and a survey of beneficiaries[15], concluded that while the scheme was beginning to reach its intended beneficiaries uptake was lower among scheduled castes and tribes. This was confirmed by Fan and colleagues[16], who used variations in programme roll-out over time and districts to evaluate the scheme using National Sample Survey data collected before and after its launch. They reported reduced out-of-pocket expenditure in this initial phase but no major impact on catastrophic healthcare expenditure. Inspired by Aarogyasri and mindful of the political benefits of introducing popular health reforms, other states have launched health financing innovations similar to this model.

RSBY in Maharashtra

RSBY was launched across a number of states by the Ministry of Labour, Government of India (GOI) in 2008[17] and provides access to free inpatient hospital care up to USD 550 (INR 30,000) per family per year[18]. Households which meet the criteria based on the much more limiting definition of poverty and numbers of poor families provided for each State by the GOI Planning Commission are eligible to enrol, and pay a contribution of USD 0.55 (INR 30) at registration and at each annual renewal[9]. Up to 5 family members, including those with pre-existing conditions can be covered, and personal information including biometric data are collected prior to the issue of a smart card with encoded details of the family. 700 procedures covering 18 broad categories of interventions which would generally be included under the umbrella of 'secondary' care, are provided and the benefit packages include the intervention, public transport costs limited to 1.8 USD (INR 100) per visit and 18.2 USD (INR 1000) per year and post-hospitalization drugs for 5 days. Networked hospitals are required to provide free outpatient consultations (which have only recently been introduced. Personal communication, Kurian OC) but other costs such as ambulatory diagnostics and medicines

For peer review only

have to be borne by the beneficiaries, except if investigations lead to inpatient admissions within a day[9]. A pilot of the RSBY scheme was launched in 1 district of AP, but only after the start of our household survey.

In Maharashtra, enrolment began in Aug 2009, and by mid 2013, approximately 2 million of the eligible 4 million families were enrolled in the scheme[12] which is being implemented in 31 of 35 districts in the state. Enrolment had extended to 26 districts prior to June 2012, when our household survey began. Notably, only 15 out of 1215 hospitals contracted for RSBY funded services are from the public sector [12]. Early assessments of the scheme's impact nationally, suggest that although the rate of hospitalisations has increased, awareness of the scheme was poor and remains a barrier to uptake. Notable variations in enrolment and scheme awareness were also observed by a descriptive study of RSBY conducted in the Amravati district of Maharashtra which has a large tribal population[19]. In Maharashtra[17], utilisation rates have been reported to be lower than in other states and the male:female enrolment ratio is 6.5:3.5.

Other major health sector initiatives in AP and Maharashtra
Both states have a complex health care landscape with numerous programmes in place. There are several initiatives launched during the past decade, some of which are common to both states and driven by national strategies, and others owe their existence to state-level enterprise, innovation and political support. The most notable programmes with the potential to impact on in patient care are described below.

The National Rural Health Mission (NRHM) was launched in 2005 nationwide, with a key aim of reducing maternal and infant mortality[20]. Government reports suggest that its notable achievements include an increase in institutional deliveries; in AP from 1.25 million in 2005-06 to 1.46 million by 2011-12[21] and in MH from 1.1 million to 1.63 million[22], achieving an institutional delivery rate of approximately 92 percent in both states. Also common to both states is the '104 health information help line' launched in AP in 2008 and MH in 2011[23], to provide medical advice and information based on validated algorithms and disease summaries, direct callers to appropriate health facilities or to receive a complaint against a public sector health facility. In AP the help line and call centre were subsumed within the Aarogyasri infrastructure by 2011.

In MH, the RSBY was preceded by the Jeevandayee scheme launched in 1997 with the objective of reducing catastrophic OOPE on inpatient care in the BPL population[24]. Potential beneficiaries were required to apply for funding after a diagnosis was confirmed and the scheme covered serious illness such as cardiac and renal disease and cancer. However, the scheme uptake has been low, and while it has continued to run in parallel to the RSBY, only 66,853 procedures (4456 procedures per year in a state with 112.37 million people) have been approved during the scheme's lifetime[25]. Since 2006, MH has also had a scheme in place which mandated 20% of the beds in private hospitals to be made available for free or at subsidized rates to poor patients (personal communication, Kurian OC). It has been estimated that around 10,000 private beds are available for the poor across MH, equivalent to approximately 20% of the total bed capacity of the public sector. Although the implementation of the scheme is reported to be erratic (Kurian OC), it may have had some positive impact on access to hospital inpatient care for serious illness.

Launched in 1995-96, the Navasanjeevani Yojana scheme is exclusive to the 15 tribal districts of MH and was to improve maternal and infant mortality in these vulnerable populations[26]. It has focused on strengthening primary health and nutrition services and access to safe drinking water.

A service available in AP but not in MH is the '108' scheme, launched in 2005 to provide a state-of-the-art medical emergency response service[27]. At the time of our study, 802 ambulances catered to approximately 3,500 emergencies per day[28].

For peer review only - http://bmjopen.bmj.com/site/about/guidelines.xhtml

OBJECTIVES OF THE STUDY

Our objective was to compare the effects of health innovations over time on access to and OOPE on inpatient care in AP and MH and to assess whether the AP initiatives had larger or smaller beneficial effects than those found in MH. These differential effects are likely to be substantially due to the Aarogyasri scheme in AP and the RSBY in MH. In this paper, we report findings from a study which compared these trends. The findings do not allow us to draw inferences on the impacts of individual initiatives, but nevertheless contribute new knowledge on the impact and role of the innovations, provide lessons for other programmes, and strengthen the evidence base for policy on UC in India.

METHODS

Overview

None of the aforementioned initiatives – including the Aarogyasri and RSBY schemes in which we are especially interested – was piloted in a systematic way, let alone via a carefully designed randomized control trial. Following MRC Guidance[29,30] and best practice we therefore opted for a quasi experimental design in which we seek to minimise selection bias, to control for confounding variables and to reduce the effects of chance. Specifically, we compare changes in hospital inpatient care related expenditures and behaviours (HREB) in AP and MH before and after the rollout of the Aarogyasri scheme in AP and the RSBY scheme in MH. The difference in changes between AP and MH is not an estimate of a specific initiative. Rather it tells us whether, on balance, the AP initiatives have had larger (or smaller) beneficial effects than the MH initiatives, and if so how much more (or less) beneficial they have been. Since the NRHM was common to both states and the MH-specific initiatives were quite small in scale or unlikely to affect HREBs, any difference in change between the two states is quite likely to be mainly due to differential effects of the Aarogyasri and RSBY programmes.

AP and MH have a broadly similar development profile as shown by the data below~~(Figure 1)~~. AP's other socio-economically similar neighbouring states of Karnataka and Tamil Nadu had already introduced Aarogyasri-like schemes, and Odisha and Chattisgarh the only other neighbours, had comparatively higher levels of socio-economic deprivation. HREBs were measured in both AP and MH by two waves of household survey before (2004) and after (2012) the introduction of Aarogyasri and RSBY. The study protocol and questionnaire for the 2012 survey were reviewed and agreed by the Research Ethics Committee of the Administrative Staff College of India, Hyderabad.

~~**Figure 1. Location of and selected indicators for Andhra Pradesh and Maharashtra**~~

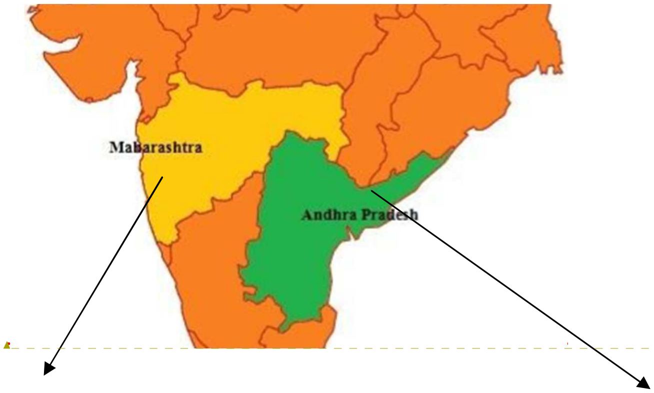

Formatted: Font: Bold

For peer review only - http://bmjopen.bmj.com/site/about/guidelines.xhtml

Maharashtra	Indicator	Andhra Pradesh
112.37	Population (2011 census, in millions)	84.66
10.20	% Schedule Caste (2001 Census)*	16.60
8.90	% Schedule tribe (2001 census)*	6.20
101,314	Per capita income 2011-12 (in INR)**	71,540
35	Number of districts	23
5,314	Households covered in NSSO 60th round (2004-05)	5,059
10,073	Households covered in the study 2012	8,623

*Note that 2011 census data for social groups are not yet published

**Source: Presentation on Annual Plan 2012-13 and Five Year Plan 2012-17[31]

Survey design

Baseline Survey : 2004

We used the original data from the National Sample Survey Organization (NSSO) 60th decennial round household survey undertaken in 2004[32] to estimate baseline HREB estimates for AP and MH (Table 1). This was the most recent round measuring morbidity profiles, use of health care services including hospitalised and non-hospitalised treatments and expenditures incurred. The household survey used a multi-stage stratified sampling methodology to identify a representative random population sample and an interviewer completed questionnaire to obtain measures of HREB along with socio-demographic, household expenditure and other information. (see Online Appendix for full technical details of sampling method and full questionnaire).

Follow up survey: 2012

We used the same household survey design and methods to collect post-intervention data in AP and MH as those used by NSSO. Briefly, the household survey used a multi-stage stratified sampling methodology with the 'First Stage Units' (FSUs) identical to those used by NSSO in their 66th round (2008-09)[33], the latest round for which FSUs had been mapped. However, the FSUs were not the same as those in NSSO 2004, our baseline survey, rapid urbanisation having changed substantially, the urban-rural landscape of both states and thus the geographical basis for sampling units.

Table 1: Urban and rural populations and households surveyed in 2004 and 2012 in Andhra Pradesh and Maharashtra

	Andhra Pradesh		Maharashtra	
	NSSO 60th round 2004-05	Our survey 2012	NSSO 60th round 2004-05	Our survey 2012
Population	76,210,007*	84,665,533**	96,878,627*	112,372,972**
Urban population	20,808,940*	28,353,745**	41,100,980*	50,827,531**
Rural population	55,401,067*	56,311,788 **	55,777,647*	61,545,441**
Total households (urban)	4,397,138*	6,778,225**	8,403,224*	10,813,928**
Total households (rural)	12,607,167*	14,246,309**	11,173,512*	13,016,652**
Total households	17,004,305*	21,024,534**	19,576,736*	23,830,580**
FSUs (urban)	183	372	267	504
FSU (rural)	325	491	265	504
Total households	1824	3715	2664	5038

For peer review only - http://bmjopen.bmj.com/site/about/guidelines.xhtml

surveyed (urban)				
Total households covered (rural)	3235	4908	2650	5035

*2001 census ** 2011 census

FSU - First Stratum Unit

The NSSO 66th round had 492 rural FSUs in AP, but 1 FSU was found to be uninhabited.

The interviewer completed household survey questionnaire was pre-tested and then piloted in both states prior to the survey. All respondents provided written informed consent for participation. Questions addressed the following: household composition and socio-demographic characteristics of members, household expenditure, and health expenditure (outpatient and inpatient) and means of its financing, healthcare seeking behaviour, factors affecting access to healthcare and awareness and perceptions of the quality of the *Aarogyasri* scheme (AP only). The survey questions in 2012 were identical to those from the NSSO 2004[34]. Additional questions specific to the Aarogyasri and other relevant schemes were also added.

A survey of 18696 households across 2 states and 1871 locations within the states is a challenging undertaking. The survey design had several features intended to assure the quality of data collected. Few academic institutions have internal capacity to carry out such large surveys, and consequently, the Social and Research Institute of IMRB International, a leading market research agency was selected to carry out the survey. The Institute has field survey teams based in every Indian state, conversant in local languages and dialects and trained to carry out surveys in the socio-economic development sector. Its clients include Government of India (for whom the national Family Health Survey data are collected), World Bank and other UN organisations. A group of NSSO consultants in AP and the Indian Socioeconomic Research Unit, Pune were recruited to support the training of the field survey teams and data verification.

We planned three levels of verification of the study data; the first to be undertaken by the survey agency, the second to be carried out by the study team and the third, by the agencies mentioned above. Survey teams for each district were accountable to a field supervisor who was responsible for checking both the household listing and data entry on a daily basis. The study team also accompanied the field staff to survey sites on a regular basis. Data collected from 250 households in each state (approximately 2.5% of the surveyed households) and 186 of the FSU listings (approximately 10%) were independently verified by the agencies in both the villages and urban blocks in order to ensure that the sampling method and administration of the questionnaire survey were being correctly applied. The data entry was carried out by the Institute using a double entry method and any questionnaires reported incorrect were sent back to the field for re-survey. The research team carried out a final validation and review of the data.

Outcome measures

Average inpatient (IP) expenditure per household per year. Average out-of-pocket expenditure for inpatient care during 1 year prior to the survey was estimated from questionnaire responses for AP

For peer review only - http://bmjopen.bmj.com/site/about/guidelines.xhtml

and MH from both baseline and follow-up data. Reimbursements for inpatient expenditure were deducted from the total where households had received them.

Large out-of-pocket inpatient expenditure. Because of the limited data on household consumption in the 2004 NSSO health survey we did not estimate 'catastrophic health expenditure'. Instead, we constructed a measure of 'large' out-of-pocket expenditure. The Aarogyasri Health Care Trust data on expenditure incurred by the Government of AP per case in 2012 were examined[14] and the mean was estimated as USD 419 (INR 23,000). A household was deemed to have incurred 'large' expenditure if OOPE for inpatient care was equal to or greater than this threshold.

Large borrowing. We estimated the total amount borrowed by a household to meet the expenditure of all the inpatient episodes of that family during the previous year. A household was considered to have incurred 'large borrowing' if the borrowing was equal to or exceeded the BPL threshold set by Government of AP: INR 70000 for urban families and INR 65000 for rural households. These prices have been deflated to 2004 levels.

Hospitalisation rate. This was estimated as the number of individuals hospitalised during the previous year, per 1000 population.

Variations in outcomes were examined between male and female-headed households, and rural and urban populations, as well as across social groups and economic groups represented by asset quintiles.

Because of the limited data on household consumption in the 2004 NSSO health survey which made the estimation of wealth difficult, we have opted instead to measure household living standards using an asset or wealth index based on information on ownership of household durables, dwelling type, etc., using principal component analysis to estimate weights[35, 36,37] for each indicator. The indicators used were limited to those collected in the 2004 NSSO: type of structure of the dwelling unit, type of toilet, type of fuel used for cooking and source of drinking. Data from the 2004 and 2012 surveys were pooled so that the index captures changes in living standards between the two years; there are therefore more households in the top quintile in 2012 than in 2004. The statistical software Stata 11 was used to generate the index.

Deflation of follow-up expenditure estimates.
The 2012 expenditure data including the threshold for large expenditures were deflated using the consumer price index of the Government of India[38] to reflect 2004 prices.

Analysis
Our repeat cross-sectional surveys do not allow estimation of within-individual household changes in outcomes over time. Our analysis therefore focused on estimating outcomes averaged across states, and in comparing changes in these over time between AP and MH. If we assume that outcome determinants other than Aarogyasri and RSBY remained stable in the two states over time or followed a parallel trend, then a difference-in-difference (DID) analysis will uncover the net effect of Aarogyasri over and above RSBY.

The difference in differences of outcome (Y_{DD}) is

$$(Y_{2012}^{AP} - Y_{2004}^{AP}) - (Y_{2012}^{MH} - Y_{2004}^{MH})$$

where the subscripts and superscripts for Y refer to the respective states and the years when the surveys were done. Confidence intervals were calculated from the standard error Y_{DD} of and the p-

For peer review only - http://bmjopen.bmj.com/site/about/guidelines.xhtml

value for the null-hypothesis ($Y_{DD} = 0$) was tested using the Wald test as $t = \frac{Y_{DD}}{SE_{Y_{DD}}}$ with one degree of freedom. Y_{DD} was estimated using ordinary least square regression:

$$y_{it} = \beta_0 + \beta_1 state_i + \beta_2 survey_t + \beta_3 (state * survey)_{it} + \sum_{k=1}^{m} \beta_{3+k} covariate_k + \varepsilon$$

The basic DID results are obtained using the above regression with covariates excluded. The adjusted DID results are obtained using the above regression with m=9 covariates, namely the gender of head of household, a dummy variable capturing whether the household lives in a rural or urban location, three dummy variables capturing the household's social group (the lowest is the excluded category), and four asset quintile dummies (the bottom is the excluded category). In the regression y_{it} is the outcome, *state* is a dummy variable with 0 for MH and 1 for AP, and *survey* is a dummy variable with 0 for 2004 survey and 1 for 2012 survey. The coefficient for the interaction term, β_3, gives the differences in differences estimate, Y_{DD}. Robust standard errors of Y_{DD} were calculated to account for clustering of households within FSUs using Stata survey commands. A positive value for Y_{DD} suggested that the change in the outcome in AP was more than the change in MH and the negative value would suggest the reverse.

An advantage of regression based DID estimate is this ability to use co-variates which can account for differential trajectories in the two states. In addition to this we did sub-group analysis stratifying for different co-variates. This is particularly relevant in the case of ~~S~~scheduled tribes whose proportion increased in MH in the follow up survey.

Sub groups were not mutually adjusted for the analysis due to sample size restrictions in relation to some of them.

Role of the funding sources

The external funding sources had no role in study design, data collection, analysis, interpretation or reporting, or in submission decision.

RESULTS

A total of 5314 and 5059 households from Maharashtra and AP were surveyed by the NSSO in 2004 (Table 2). Our survey in 2012 included 10073 (MH) and 8623 (AP) households.

Table 2. Socio-demographic characteristics of baseline and follow-up samples

Subgroups	Number (%) of Households 2004	Number (%) of Households 2012

For peer review only - http://bmjopen.bmj.com/site/about/guidelines.xhtml

	Maharashtra	Andhra Pradesh	Maharashtra	Andhra Pradesh
**All**	5314	5059	10073	8623
**Head of Household**				
Male	4785 (90.0)	4433(87.6)	8543(84.8)	7418(86.0)
Female	529 (10.0)	626 (12.4)	1530 (15.2)	1205 (14.0)
**Social Group**				
Scheduled Tribes	413 (7.8)	296 (5.9)	1364 (13.5)	883 (10.2)
Scheduled Castes	809 (15.2)	974 (19.3)	2235 (22.2)	1797 (20.8)
Other Excluded	1644 (30.9)	2317 (45.8)	1899 (18.9)	3419 (39.7)
All other Groups	2448 (46.1)	1472 (29.1)	4571 (45.4)	2524(29.3)
**Location**				
Rural	2,650 (49.9)	3235 (63.9)	5035(50.0)	4908 (57.0)
Urban	2664 (50.1)	1824 (36.1)	5038 (50.0)	3715(43.0)
**Asset Quintile**				
Lowest	1,260 (23.7)	1,594(31.5)	996(9.9)	826(9.6)
Second	1,016 (19.1)	1,237 (24.5)	1,841(18.2)	1,286(14.9)
Third	772 (14.5)	753(14.9)	2,228(22.1)	2,121(24.60)
Fourth	857 (16.1)	744(14.7)	2,373 (23.6)	3,072(35.6)
Fifth	1,408(26.5)	730(14.4)	2,633(26.1)	1,318(15.3)

Changes in average in-patient expenditure

Table 3 (top panel) shows average baseline levels of inpatient expenditure. The table also shows the real terms change (deflated to 2004 prices) in these outcomes at follow up and the difference in difference (DID) estimate comparing AP with MH. DIDs for overall results are shown unadjusted, as well as adjusted for the effects of the covariates. Breakdowns by sex of head of household, social group, urban/rural location and asset quintiles are also shown.

Overall, average inpatient expenditure increased in real terms in both the states between 2004 and 2012, but the increase was significantly greater in MH (unadjusted DID= -498.2 INR, 95% CI= -792.9: -203.5, p=0.0009). The trend of a greater increase in MH was evident across all sub groups of analysis except the richest asset quintile. However, the DIDs reached significance in male headed households (DID=-513.7INR, 95% CI= -843.9: -183.4, p=0.0023), scheduled castes (DID= -708.7INR , 95% CI=-1234.3: -183.2, p=0.0082), all 'other' social groups (DID=-1110.46INR, 95% CI=-1868 : -352.9, p=0.0041), rural households (DID= -504 INR, 95% CI= -801.9: -206.0, p=0.0009) and the poorest (DID= -1001.3 INR, 95% CI= -1751: -251.7, p= 0.0089) and middle asset quintiles (DID= -798.1 INR, 95% CI= -1362.9:-233.3, p= 0.0056).

Table 3: Change in average inpatient expenditure (in INR) in Maharashtra and Andhra Pradesh between 2004 and 2012

For peer review only - http://bmjopen.bmj.com/site/about/guidelines.xhtml

Subgroups	Baseline Mean (95% CI)		Change 2004:2012 Mean (95% CI)		DID	
	Maharashtra	Andhra Pradesh	Maharashtra	Andhra Pradesh	Mean (95%CI)	p
Household inpatient expenditure						
**All**	1091.6(978.4:1204.8)	723.5(527.5:919.5)	942.8(749.9:1135.6)	444.55(221.5: 667.6)	-498.2(-792.9:-203.5)	0.0009
					DID (adjusted for covariates)	
					-565.8(862.9: -268.6)	0.0002
**Head of Household**						
Male	1,132.9(1015:1,251)	758(419.7:1,096.3)	935(727:1,143.01)	1074.9(555.9:1593.8)	-513.7(-843.9: -183.4)	0.0023
Female	757.1(555.5:1014.6)	341.1(222.3:460.01)	421.3(164.7:678.0)	589.9(307.22:872.8)	-484.9(-1075.6:105.9)	0.1076
**Social Group**						
Scheduled Tribes	376.6(231.7:521.6)	432.7(212.52:652.9)	1153.1(803.3:1502.9)	675.2(163.2:1187.2)	-477.9(-1097.7:142)	0.1307
Scheduled Castes	696.7(500.2:893.2)	432.6(305.6:559.4)	1464.1(1039.9:1888.4)	755.4(444.9:1065.9)	-708.7(-1234.3: -183.2)	0.0082
Other excluded	1,028.6(838.4:1218.8	562.4(463.7:662)	928.9(532.9:1324.9)	767.9(569.6:966.2)	-161(-603.7: 281.7)	0.4758
All other Groups	1,424.5(1,222.3:1626.7)	1306.2(627.8:1984.6)	734.9(427.9:1041.7)	-375.6(-1,068.5:317.4)	-1110.46(-1868 : -352.9)	0.0041
**Location**						
Rural	897.8(768.1:1027.5)	571.4(496.2:646.6)	1084.7(826.3:1343.1)	580.7(432.2:729.2)	-504(-801.9: -206.0)	0.0009
Urban	1343.5(1146.1:1540.9)	1113.5(466.2:1760.8)	753.6(458.7:1048.6)	92.3(-586.92:771.5)	-661.3(-1401.5: 78.864)	0.0799
**Quintile**						
poorest	656.3(498.0:814.6)	391.5(319:464.1)	1692.5(1053.3:2331.7)	691.2(298.9:1083.5)	-1001.3(-1751: -251.7)	0.0089
2nd	786.5(583.5:989.5)	443.3(356.5:530.2)	979.3(599.4:1359.2)	839.5(465.7:1213.3)	-139.8(-672.5:393)	0.607
middle	1062.7(738.8:1386.1)	862.1(577.8:1146.5)	1011.8(550.2:1473.4)	213.7(-112.1:539.6)	-798.1(-1362.9: -233.3)	0.0056
4th	1241.7(894.4:1589.1)	1819(337.5:3302.5)	803.6(328.7:1278.5)	-644.3(-2128.3: 839.7)	-1447.9(-3005.2:109.5)	0.0684
richest	1818.6(1505.5:2131.8)	908.3(682.1:1133.4)	252.3(-193.4:698.1)	362.1(15.3:708.9)	109.7(-454.80:674.3)	0.7031

Large expenditures for inpatient care

For peer review only - http://bmjopen.bmj.com/site/about/guidelines.xhtml

Proportions of households incurring large expenditures showed an increase in both states (Table 4), but the increase was smaller in AP for the sample as a whole as well as for all the groups except for the second asset quintile. The DID was strongly significant for the households overall (adjusted DID=-1.8, 95% CI: -3:-0.7, p= 0.0009), but this was not observed for any of the sub groups of analysis.

Table 4: Change in the proportion (%) of households incurring large health expenditures for in-patient care in Maharashtra and AP between 2004 and 2012

Subgroups	Baseline Mean (95% CI)		Change 2004:2012 Mean (95% CI)		DID	
	Maharashtra	Andhra Pradesh	Maharashtra	Andhra Pradesh	Mean (95%CI)	p
Large IP expenditure (INR 23000 deflated to 2004 figures)						
**All**	6.7(6:7.3)	3.4(2.9:3.9)	3.1(2.1:4.1)	2.2(1.5:2.8)	-0.91(-2.1:0.27)	0.1302
					DID (adjusted for covariates)	
					-1.8(-3:-0.7)	0.0009
**Head of Household**						
Male	6.8(6.2:7.6)	3.5(3.1:4)	3.1(2.0:4.1)	2.1(1.5:2.9)	-0.8(-2.1:-0.4)	0.1928
Female	5.0(3.7:6.4)	2.8(1.9:3.7)	3.9(2:5.8)	2(6.6:3.4)	-1.8(-4.2:0.50)	0.1222
**Social Group**						
Scheduled Tribes	2.2(1.3:3.1)	1.4(0.5:2.2)	5.3(3.5:7)	3.5(1.9:5.1)	-1.7(-4.1:0.61)	0.1478
Scheduled Castes	6.1(4.6:7.7)	2.1(1.5:2.6)	4.3(2.3:6.3)	3(1.9:4.1)	-1.2(-3.5 :1.01)	0.2785
Other Excluded	5.9(4.7:7.0)	3.3(2.7:3.8)	2.9(1.1:4.6)	2.7(1.7:3.6)	-2.1(-2.2:1.8)	0.8389
All other Groups	8.3(7.5:7.9)	5.4(4.4:6.3)	2.2(0.9:3.6)	0.51(-0.7:1.7)	-1.7(-3.5:0.04)	0.0628
**Location**						
Rural	1.9(1.5:2.2)	0.9(0.79:1.1)	1.7(1.1:2.3)	1.3(0.09:1.6)	-0.45(-1.1:0.25)	0.2098
Urban	12.9(11.9:14)	9.7(8.9:10.7)	4.4(3.0:5.7)	3.9(2.6:5.3)	-0.7(-2.4: 1.5)	0.6350
**Quintile**						
poorest	1.8(1.3:2.4)	1.1(0.8:1.4)	3.7(2.2:5.2)	1.7(0.7:2.7)	-0.2(-3.8:-0.19)	0.0307
2nd	2.7(1.9:3.5)	1.2(0.9:1.5)	2.1(0.93:3.3)	2.2(1.4:3.1)	0.9(-1.4:1.6)	0.9079
middle	6.9(4.9:8.9)	4.2(3.1:5.3)	1.3(-1:3.6)	0.9(-1.2:1.4)	-1.2(-3.9:1.4)	0.3596
4th	10.7(8.7:12.6)	7.6(5.9: 9.2)	1.8(-0.57:4.3)	-0.036(-1.9:1.80)	-1.9(-4.9:1.2)	0.2268
richest	13.5(12:14.9)	9.6(8.1:11.2)	0.3(-1.6:2.2)	-0.6(-2.8:1.6)	-0.9(-3.7:2)	0.5601

Changes in large borrowing for inpatient care

In both states proportions of households incurring large borrowings to meet inpatient expenses increased from 2004 to 2012 (Table 5). However, there was a consistent pattern of smaller increases in AP for the overall population, as well as all subgroups (except the richest asset quintile) with DIDs strongly significant for the overall population (adjusted DID=-4, 95% CI: -6.6:-1.4, p= 0.0032), scheduled tribes (DID= -5.5, 95% CIs:-9.3:-1, p= 0.0048), rural households (DID= -4.7, 95% CIs: -7.3 : -2.1, p= 0.0007) and all asset quintiles except the richest (the poorest asset quintile DID= -9.0, 95% CI: -14.0: -4.4, p=0.0002).

For peer review only - http://bmjopen.bmj.com/site/about/guidelines.xhtml

Table 5: Change in the proportion (%) of households large borrowings for in-patient care in Maharashtra and AP between 2004 and 2012

Subgroups	Baseline Mean (95% CI)		Change 2004:2012 Mean (95% CI)		DID	
	Maharashtra	Andhra Pradesh	Maharashtra	Andhra Pradesh	Mean (95%CI)	p
Proportion of households having large borrowings						
**All**	7.5(6.7:8.2)	3.8(3:4.5)	8.9(6.8:11)	5.3(3.4:7.2)	-3.7(-6.4:-0.908)	0.0100
					DID (adjusted for covariates)	
					-4(-6.6:-1.4)	0.0032
**Head of Household**						
Male	7.8(7.0:8.5)	3.9(3.1:4.7)	9.8(6.6:1.3)	5.3(3.3:7.3)	-3.6(-6.6: -0.62)	0.0187
Female	5(3:7)	2.9(1.7:4.1)	8.(6.6:11.2)	5(2.9:7.23)	-4.7(-8.3:-1)	0.0137
**Social Group**						
Scheduled Tribes	3.6(2.3:4.9)	2.4(0.93:3.9)	11(8.9:14)	5.8(2.8:8.8)	-5.5(-9.3: -1.8)	0.0048
Scheduled Castes	7.2(5.5:8.8)	3(1.7:4.2)	9.6(6.6:13)	5.8(3.4:8.3)	-3.8(-7.5:0.03)	0.0518
Other Excluded	8.0(6.8:9.2)	3.5(2.7:4.4)	8(5.8:10.3)	5.3(3.2:7.4)	-2.8(-5.7:0.19)	0.0661
All other Groups	8(7.15:8.8)	0.052(.040:0.064)	8.8(5.9:12)	4.7(2.2:7.4)	-4.1(-7.9:-.4.0)	0.0302
**Location**						
Rural	6.5(5.6:7.5)	0.03(0.024:0038)	10(8.5:12)	5·8(3.9:7.6)	-4.7(-7.3:-2.1)	0.0007
Urban	8.7(7.3:10)	0.056(0.048:0.064)	7.0(4.5:9.5)	4(1.1:6.9)	-3.0(-6.7:0.68)	0.1081
**Quintile**						
poorest	5.2(3.9:6.5)	0.025(0.016:0.033)	12.1(7.8:16)	3.1(1.3:0.049)	-9(-14:-4.4)	0.0002
2nd	0.064(0.048:0.08)	0.027(0.021:0.032)	0.095(0.070:0.12)	0.052(0.034:0.070)	-0.043(-.073: -.013)	0.0062
middle	0.074(0.050:0.098)	0.048(0.031:0.065)	0.10(.073:0.133)	0.044(0.013:0.076)	-0.059(-.100:-.017)	0.0069
4th	0 .087(0.063:0.110)	0.06(.041:0.078)	0.083(0.061:0.104)	0.039(0.014:0.064)	-0.044(-.075:-.012)	0.0076
richest	0.10(0.090:0.12)	0.064(0.049:0.079)	0.045(0.0035:0.086)	0.049(-.0068:0.105)	0.0045(-.062:0.071)	0.8937

Hospital utilisation for in-patient care

Overall, hospitalisation rates have increased in both AP and Maharashtra (Table 6) but more so in AP (5.6 per 1000 population vs 2.2), although the DID was not statistically significant. The sub group analysis presented a mixed picture. For both male and female headed households there was a greater increase in hospitalisation in AP, but this reached moderate statistical significance only for female headed households (DID=27.6, 95% CI =1.1:54.1, p-value = 0.0415). There is an increase in hospitalisations among scheduled tribes in Maharashtra and a reduction in AP (DID = -19.8, 95% CI = -37.3: -2.3, p-value = 0.0272) but the opposite trend was seen among 'other excluded' groups with an increase in AP and a reduction in MH (DID = 12.5, 95% CI= 1.2:23.9 p-value = 0.0309). In scheduled castes hospitalisations had increased in both states but more so in AP, while in the 'other' group there was a small increase in MH and a small reduction in AP. In the poorest quintile, the increase in hospitalisation was significantly greater in MH (DID= -14.4, 95%CI=-28:-0.31, p= 0.0451).

For peer review only

For peer review only - http://bmjopen.bmj.com/site/about/guidelines.xhtml

Table 6: Changes in hospitalisation (per 1000 population) in Maharashtra and AP between 2004 and 2012

Subgroups	Baseline Mean (95% CI)		Change 2004:2012 Mean (95% CI)		DID	
	Maharashtra	Andhra Pradesh	Maharashtra	Andhra Pradesh	Mean (95%CI)	p
Hospitalizations per 1000 population						
**All**	41.3(37.3:45.2)	31.5(27.8:35.3)	2.2 (-4.7:9.1)	5.6(-1.1:12.3)	3.4(-5.9 :12.7)	0.4636
					**DID (adjusted)**	
					0.7(-8.6:10.2)	0.8685
**Head of Household**						
Male	41.0(37.1:44.9)	31.7 (16.9:34.0)	1.9(-5.1:8.8)	4.4(-2.4:11.1)	2.5(-6.9:11.9)	0.5966
Female	51.6 (30.6:72.5)	25.5(27.7:35.7)	13.9(-7.53:35.4)	41.5 (24.6:58.4)	27.6(1.1:54.1)	0.0415
**Social Group**						
Scheduled Tribes	23.7(14.2 :33.1615)	35.5(21.9:49.1)	17.1(5.8:28.5)	-2.7(-16.95:11.5)	-19.8(-37.3: -2.3)	0.0272
Scheduled Castes	42.4(36.3:48.4)	29.5(22.6:36.5)	3.9(-7.9:15.7)	7.6(-2.6:17.7)	3.7(-11.4:18.7)	0.6268
Other Excluded	44.2(38.2:50.2)	29.9(25.5:34.3)	-1.9(-11.2 :7.3109)	10.6(3.4:17.8)	12.5(1.2:23.9)	0.0309
All other Groups	42.5(37.1:47.9)	34.9(29.0:40.9)	0.93(-7.3:9.1)	-1.1(-9.4: 7.4)	-2.0(-13.5 :9.4)	0.7235
**Location**						
Rural	36.6(32.2:41)	28.9(26.4:31.5)	9.5(3.5:15.4)	8.8(2.2:15.3)	-0.69(-9.3:7.9)	0.8725
Urban	48.2(43.7:53)	38.2(35.5:41.2)	-7.9(-14.5: -1.3)	-2.5(-12.1:7.1)	5.4(-5.8:16.6)	0.3358
**Quintile**						
poorest	31.4(25.9:36.9)	27.5(22.8:32.1)	20.7(9:32.8)	6.4(-1.7:14.4)	-14.4(-28:-0.31)	0.0451
2nd	36.5(28.3:44.7)	27.1(21.9:32.4)	8(0.5:15.4)	8.9(-.56:18.4)	0.9(-10 : 12.5)	0.8746
middle	47.8(33.6:62.1)	35.3(29.0:41.6)	-1.5(-17.9:14.8)	4.1(-.56:13.7)	5.6(-12.8:24.0)	0.5457
4th	46.9(39.9:53.9)	41.7(32.7:50.8)	-4.0(-12:3.4)	-5.1(-15.7:5.4)	-0.75(-13.3:11.9)	0.9056
richest	51.0(46:56.1)	36.5(25.8:47.2)	-13.1(-18.9: -7.1)	1.5(-19.5:22.6)	15.0(-5.8: 3.6)	0.1665

LIMITATIONS OF THE STUDY

Difference in difference estimations aimed at assessing the impacts of interventions, assume that both populations demonstrate similar characteristics prior to the start of the intervention, and that 'unobservables' follow a common trend; under such circumstances, any differences in changes observed over time between the 2 populations are attributable to the interventions[39, 40]. Despite AP and Maharashtra having broadly similar socio-economic profiles, as well as our DID analysis taking account of a number of covariates, there may have been other factors resulting in unobserved differential changes between the 2 populations and to which the results of the DID analysis may be at least partially attributable.

A second limitation could arise from the impact of other public health programmes implemented during the period 2004 to 2012. The most significant of these is the National Rural Health Mission

For peer review only

For peer review only - http://bmjopen.bmj.com/site/about/guidelines.xhtml

launched in 2005, mainly to improve maternal and child health through the revitalization of rural primary care and child and maternal health services. A key assumption of our study is that the impacts of the NRHM in terms of healthcare expenditure for maternal and child health care would have been similar in both states, as this was a nationwide development. Despite the improvements in the public sector maternal and child health services sought by the NRHM, it is widely recognised that the public, including BPL families, continues to pay OOP for private health care. We have assumed that this behaviour is likely to be similar across the 2 states. Other health initiatives such as the Navsanjeevani Yojana and HMRI were unlikely to have had an impact on inpatient care or expenditure. The 108 scheme had the potential, in AP, to influence hospitalisation rates, by helping more households to visit hospitals when seriously ill. But the effect is likely to be small as the majority of even serious illnesses do not result in a 108 call for transport. The implementation of the RSBY and the scheme to make private hospital beds available for the poor in MH may have diluted the DID, although both schemes are known to have been only partially implemented.

Lastly, the 2004 NSSO survey which served as our baseline, was carried out between January and June 2004. Our end-line 2012 survey was carried out over a period of 3 months from June to September. The morbidity and mortality patterns recorded in different time periods may vary, and could have influenced the data.

DISCUSSION

We found that average expenditure, large expenditures and large borrowings on inpatient care had increased in both MH and AP, but the increase was consistently smaller in AP across these 3 outcome measures, and may be suggestive of Aarogyasri having a somewhat larger effect than RSBY. Similar increases in institutional deliveries across the 2 states, and low levels of utilisation of RSBY and Jeevandayee schemes in MH may further strengthen this explanation.

The increase in average OOPE on inpatient care in AP and MH reflects a trend observed nationwide[16, 41]. The Aarogyasri scheme may have contributed to the more favourable trajectory in AP both directly and indirectly, in that the scheme may have contributed to a reduction in the prices of interventions and an increase in competition among health care providers. The evaluation of the Yeshasvini scheme also found a significant reduction in the price of surgical interventions[10]. Our findings may suggest that the positive effects of Aarogyasri detected by other studies[15,16] at an early stage of the roll out of the scheme have been sustained. Automatic enrolment into the scheme, near universality of coverage and no requirement for enrollee contributions may have contributed to the significant DIDs in male headed households, scheduled castes, rural households and the poorest and middle asset quintiles. But these benefits were not demonstrated in some of the most vulnerable groups - female headed households and scheduled tribes. This is consistent with the findings of other studies[5,16] which reported that the slightly less vulnerable may benefit more from such schemes, with non-financial barriers undermining access to services for the most vulnerable socio-economic groups. The case summaries below illustrate the obstacles they face. A similar observation was reported by the evaluation of the Mexican Seguro Popular health scheme which showed that a third of the treatment-cluster households who were automatically affiliated were unaware of this fact[42].

The likely explanation of an Aarogyasri effect may be strengthened by the trend for large borrowings which has also increased over time in both states but less so in AP across all groups of analysis except the richest asset quintile. A multi-country analysis of household catastrophic health expenditure highlighted that increasing the availability of health services is critical to improving health in poor countries, but it could also raise the proportion of households facing catastrophic expenditure unless financial risk protection policies are given a high priority[43]. Borrowing of comparatively small amounts is less impactful and may be a result of improved access to financial

For peer review only

For peer review only - http://bmjopen.bmj.com/site/about/guidelines.xhtml

markets and also supports consumption smoothening. During recent decades, AP has in particular witnessed a significant rise in microfinance institutions and debt due to high levels of interest levied by the more recent entrants to this market[44, 45]. Nevertheless our results suggest that increases in large borrowings associated with inpatient health care were smaller in AP. Strongly significant DIDs in scheduled tribes, rural households and the poorest and second asset quintiles and moderately significant DIDs in female headed households, scheduled castes, and other excluded social groups may also point to Aarogyasri beginning to offer greater ~~equity of~~ access to health care when families are faced with serious illness.

In terms of large OOPE, a significant DID was found only for the total households, but the direction of all DIDs except for the second asset quintile was the same as for average expenditure on inpatient care and large borrowings, i.e., in favour of AP. It is perfectly possible that the non-significant differences for the majority of sub groups are due at least in part to the RSBY, the availability of private sector hospital beds for the poor and the Jeevandayee schemes reducing large OOPE in MH, and that without these schemes the expenditure would have been greater. In AP, 70 percent of survey households reported that they were covered by the Aarogyasri scheme, but only 25 percent of these 'covered' households were aware that the benefit package was limited. It is also possible therefore that in AP some families seek hospital care assuming that the Aarogyasri scheme provides comprehensive cover, but are faced with large expenditures when their treatments fall outside the limits.

The mixed picture in relation to the increasing rate of hospitalisations in both states may suggest that the Aarogyasri and in particular, the RSBY scheme which covers common hospital procedures are addressing a large hitherto unmet need for inpatient care. The 108 scheme in AP may be an additional albeit smaller contributor. It may also be explained by a supplier-induced demand. An assessment of health provider behaviour and governance is an important strand of the evaluation of health financing schemes[46], but was outside the remit of our study. We would however strongly recommend an impact evaluation focusing on health care supply to complement our evidence. The greater increase in hospitalisation in AP, in female-headed households and 'other excluded groups', which are two of the most vulnerable population groups, is encouraging. But ~~T~~the reduction in hospitalisations among scheduled tribes and the poorest asset quintile in AP are of concern and suggest that if the poor are to secure the benefits appropriated by the near poor or more often by the rich[47], the provision of more comprehensive health schemes is essential, which combines the tertiary and secondary care focus of Aarogyasri and the secondary care benefits of RSBY with attention paid to minimise barriers such as the widespread influence of illiteracy and lack of awareness, which limit access to even schemes such as Aarogyasri that are apparently highly inclusive and non-discriminatory, as well as distance to facilities. Our case summaries illustrate this. Furthermore, health financing reforms such as the Jamkesmas[48] in Indonesia and the Seguro Popular in Mexico[42], both countries similar to India in terms of population and growing economies, include outpatient and inpatient care, and curative as well as preventive services, suggesting that a more comprehensive service is possible to implement and worthy of consideration.

In summary, health innovations in AP had a greater beneficial effect on hospital inpatient care-related expenditures than innovations in MH. The Aarogyasri scheme is likely to have contributed to these impacts in AP, at least in part. But in both states, OOPE increased over time. Hospitalisations also increased, and while it is generally assumed that in developing countries this is likely to address genuine need, its impact on health status is not known.

IMPLICATIONS FOR POLICY AND PRACTICE

Despite these uncertainties, Aarogyasri is perceived, with some justification across India as a successful scheme, and is being rapidly replicated across the states. Since July 2012, MH too has

For peer review only - http://bmjopen.bmj.com/site/about/guidelines.xhtml

joined the list of states offering an Aarogyasri-like scheme, the Rajiv Gandhi Jeevandayee Arogya Yojana[49]. This study has highlighted that such schemes may result in some positive outcomes. Although the study was not designed to elicit the specific features of the scheme to which any comparatively greater benefits may be attributable, and which deserve to be replicated in other states, evidence from systematic reviews[3] points to the need for schemes to be more 'comprehensively' designed to maximise their positive impact. Aarogyasri's design in terms of its aims to address both financial and non-financial barriers - being fully state funded, the systematic administrative implementation of Aarogyasri across the state so that it is now almost universal, automatic enrolment, allowing access to the scheme via a ration card which most poor families own, the wide spectrum of treatments offered, the large number of health care providers empanelled - is perhaps responsible as a 'comprehensive' package, for the greater impact.

But the study also suggests that ~~equity of~~improved access to health care and the reduction of the overall burden of OOPE especially in the most vulnerable sections of the population[50] are likely to require additional interventions that address gaps in the availability of care and provide patients appropriate pathways that support their journey from a strong and comprehensive primary care service where they may be informed of their entitlements and investigated for their initial symptoms to appropriate hospitals for the treatment of serious illness. Besides, inpatient care is only consumed by a small proportion of households in a year[51], and this is especially true of tertiary care, while many more will seek outpatient services and referral to inpatient care, should this be required. Others have strongly recommended the strengthening of the primary care base as an essential means to universal health coverage and this study confirms their view[41]. Key implications for AP are to explore how best the most advantageous features of Aarogyasri can be extended to include both secondary and primary care, while those for MH may be to build on and unify its menu of currently available schemes to create an evidence-based comprehensive health delivery system. These conclusions may be applicable to other states with similar health financing schemes.

The design of health financing systems as well as their evaluations is complex and challenging, as the mountain of available evidence suggests[41, 42, 51, 52]. Even the ground-breaking Seguro Popular health insurance programme of Mexico which used a cluster-randomised trial design with strong government support demonstrated some but not all anticipated outcomes, contrary to expectations[42], and a key recommendation was that continued assessment of the programme was needed. Furthermore, differences in data and methodology may result in even very well designed evaluations of the same programme producing contrasting findings[52]. Our evaluation is the first to use a quasi-experimental methodology - the best possible in the hierarchy of evaluation methodologies, when a randomised control trial is not achievable - to evaluate the health financing reforms in 2 large states of India. Despite that, the study has limitations which we have acknowledged. For example, the evaluation was not designed to assess provider behaviours. Many other pertinent questions such as the impact of the schemes on the overall economy of health care cannot be answered by a single evaluation. But these are recognised problems which can only be addressed through continuous assessments, as other evaluations have shown. Our study has nevertheless produced sufficient insights to enable policy leaders to improve programme effectiveness and, importantly, to undertake further assessment. Our household survey data provide a valuable baseline for future monitoring in both states.

This study needs to be followed up with further and repeated evaluations as AP's and MH's schemes evolve; to assess the impacts of re-design and to help health policy leaders achieve their aspiration of universal access to good quality health care.

For peer review only

Three faces of the Aarogyasri Scheme

The beneficiary:

Lakshmamma, a 65 year old widow, is from a tribal background. She is an unskilled labourer, supporting a family of 5, with a monthly income of INR 4000 (USD 74). She was referred to a municipal hospital with chest pain and underwent heart surgery. The hospital which is a part of the Aarogyasri network provided free care and her total out of pocket expenses amounted to INR 850 (16 USD) for initial transport. In addition to the surgery, she received free food, money for transport home and follow up medicines. She is very satisfied with the service she received.

The excluded:

Ramulamma, a mother has a BPL card, but her daughter Lakshmi does not, as she was abandoned by her husband who is a government employee and entitled to free health care. The mother was unable to secure free care using her BPL card for her seriously ill daughter who paid out-of-pocket at a private facility where she was offered a hysterectomy for the relief of her gynaecological symptoms. Lakshmi's health care costs were met by the family selling a number of household assets and Lakshmi's daughter discontinuing her education to take up paid work. Lakshmi is severely depressed and does not speak to anyone.

The uninformed:

Krishnamma 43 years old, had severe stomach pains one night. Although the family had a BPL card, Ramulu her husband and Srinivas her son rushed her to a private hospital nearby, which was not part of the Aarogyasri network. They were unaware of how to access the Aarogyasri scheme hospitals which were further away from home, and the local primary health services being inadequate, Krishnamma had not had her initial symptoms investigated. The treatment was funded through a loan from a private moneylender. On discharge, she has not attended follow up, as the family cannot afford transport or medicines.

References

[1] Resolution WHA58.33. Sustainable health financing, universal coverage and social health insurance. In: *Fifty-eighth World Health Assembly, Geneva, 16–25 May 2005.* Geneva, World Health Organization, 2005 (http://apps.who.int/gb/ebwha/pdf_files/WHA58/WHA58_33-en.pdf, accessed 26th March 2013).

For peer review only - http://bmjopen.bmj.com/site/about/guidelines.xhtml

[2] World Health Organization. Health Systems Financing Path to Universal Coverage. Geneva: World Health Organization. 2010. Available from: World Health Organization Library Information Services.
[3] Acharya A, Vellakkal S, Taylor F, Masset E, Satija A, Burke M, Ebrahim S. Impact of national health insurance for the poor and the informal sector in low and middle income countries : a systematic review. London: EPPICentre, Social Science Research Unit, Institute of Education, University of London. 2012. Available from: http://www.dfid.gov.uk/r4d/PDF/Outputs/SystematicReviews/Health-insurance-2012Acharya-report.pdf
[4] Giedion, U., B. Y. Diaz . A Review of the evidence. In: The Impact of Health Insurance in Low-and Middle-Income Countries. M. L. Escobar, C. Griffin and R. P. Shaw (ed). Washington DC, Brookings Inst Press 2011.

[5] Giedion, U, Alfonso, E , Diaz. *The impact of universal coverage schemes in the developing world : a review of the existing evidence*. Universal Health Coverage (UNICO) studies series ; no. 25. Washington D.C. : The World Bank. 2013
[6] National Commission on Macroeconomics and Health. Report of the National Commission on Macroeconomics and Health. New Delhi: Ministry of Health and Family Welfare of India. 2005. Available from: Ministry of Health and Family Welfare
[7] Eleventh Five Year Plan (2007–2012). Vol 2: Social Sector. Government of India Planning Commission. October, 2008. http://planningcommission.nic.in/plans/planrel/fiveyr/11th/11_v2/11th_vol2.pdf, (accessed Mar 26 2013).
[8] Faster, Sustainable and More Inclusive Growth. An Approach to the Twelfth Five Year Plan (2012-17). Government of India Planning Commission. October, 2011. http://planningcommission.nic.in/plans/planrel/12appdrft/appraoch_12plan.pdf, accessed 26th March 2013
[9] La Forgia G, Nagpal S. Government-Sponsored Health Insurance in India. *Are You Covered?* World Bank Washington DC 2012. doi:10.1596/978-0-8213-9618-6.
[10] Agarwal A. "Impact evaluation of India's 'Yeshasvini' community based health insurance programme. *Health Economics* 2010; 19: 5-35
[11] See The scheme website at https://www.aarogyasri.org/ASRI/index.jsp, accessed 26th March 2013.
[12] Scheme status. http://www.rsby.gov.in/statewise.aspx?state=35 (accessed Apr 30 2013)
[13] Mahal A. Learning and getting better: Rigorous evaluation of health policy in India. National Medical Journal of India. 2011. 325-27.
[14] Aarogyasri Health Care Trust Annual Report 2011-2012. https://www.aarogyasri.org/ASRI/EXT_IMAGES/documents/Annual_Report_201011.pdf (accessed Mar 26 2013)
[15] Rao M, Ramachandra S, Bandyopadhyay S, et al. Addressing healthcare needs of people living below the poverty line: a rapid assessment of the Andhra Pradesh Health Insurance Scheme. National Medical Journal of India. 2011. 24(6):335-41.
[16] Fan V, Karan A, Mahal A. "State Health Insurance and Out of-Pocket Health Expenditures in Andhra Pradesh, India." CGD Working Paper 298.Washington, D.C.: Center for Global Development. 2012 http://www.cgdev.org/content/publications/detail/1426275 (accessed Mar 15 2013)
[17] Palacios R, Das J, Sun C eds. India's health insurance scheme for the poor: Evidence from the early experience of Rashtriya Swasthya Bima Yojana. New Delhi: Center for Policy Research; 2011
[18] Rashtriya Swasthya Bima Yojana overview and scheme details. http://www.rsby.gov.in (accessed Apr 30 2013)
[19] Rathi P. http://www.priorities2012.com/documents/3d-4-patreek.pdf
[20] NRHM Mission Document.http://www.nird.org.in/brgf/doc/Rural%20HealthMission_Document.pdf (accessed Apr 30 2013)
[21] State HMIS data analysis, April'10-March'11 Andhra Pradesh, prepared by NHSRC.http://cfw.ap.nic.in/nrhm/pdf/AP_Analysis_Apr2010-Mar2011.pdf (accessed May 2 2013)
[22] NRHM achievements in Maharashtra. http://www.nrhm.maharashtra.gov.in/achievements.htm (accessed May 2 2013)
[23] Health information helpline.http://www.hmri.in/oursolutions-healthinformation.html (accessed Apr 30 2013)
[24] About the project/scheme. http://www.maha-arogya.gov.in/projectandschemes/Jeevandaiaarogya/default.htm (accessed Apr 30 2013)
[25] Jeevandayee scheme performance: Information received from Rajiv Gandhi Jeevandayee Arogya Yojana Society Govt of Maharashtra on May 17 2013
[26] Projects and schemes. http://maha-arogya.gov.in/projectandschemes/itdpnavsanjivani%5Cdefault.htm (accessed Apr 30 2013)

For peer review only

For peer review only - http://bmjopen.bmj.com/site/about/guidelines.xhtml

[27] Mallepeddi R , Pernefeldt H, Bergkvist S . Andhra Pradesh health sector reform, A narrative case study, Technical paper No.7 for The Rockefeller Foundation–Sponsored Initiative on the Role of the Private Sector in Health Systems in Developing Countries. 2009 ; pp 42-48
[28]EMRI. http://www.emri.in/states.html (accessed Apr 30 2013)
[29] Developing and evaluating complex interventions: new guidance. Medical Research Council. London 2008.
[30] Cesar G V, Jean-Pierre H, Jennifer B. Evidence-Based Public Health: Moving Beyond Randomized Trials. Am J Public Health. 2004 March; 94(3): 400–405.
[31] Presentation on Annual Plan 2012-13 and Five Year Plan 2012-17. http://planningcommission.nic.in/plans/stateplan/Presentations12_13/maharashtra1213.pdf (accessed on mar 25 2013)
[32] National Sample Survey Organization. Morbidity, Healthcare and condition of the Aged. New Delhi: Ministry of Statistics and Programme Implementation. 2004.
[33] National Sample Survey Organization. Household Consumer Expenditure 66th round . New Delhi: Ministry of Statistics and Programme Implementation; 2009-10
[34] National Sample Survey Organization. Household Consumer Expenditure in India. New Delhi: Ministry of Statistics and Programme Implementation; 2005.
[35] Booysen F, Van der Berg S & Burger R. Using an Asset Index to Assess Trends in Poverty in Seven Sub-Saharan African Countries. World Development. 2008; 36(6): 1113–1130
36 Vyas S. & Kumarnayake L. Constructing socio-economic status indices: How to use principal components analysis. Health Policy and planning. 2006; 459-468
[37] O'Donnell O, Van Doorslaer E, Wagstaff A & Lindelow M, 2008. Analyzing Health Equity Using Household Survey Data: A Guide to Techniques and Their Implementation. Washington DC: World Bank Institute. Chapter 6, Measurement of Living Standards. http://siteresources.worldbank.org/INTPAH/Resources/Publications/459843-1195594469249/HealthEquityFINAL.pdf (accessed May 9 2013)
[38] Ministry of Finance. Economic Survey 2011-12. New Delhi: Government of India. 2012. Available from: http://indiabudget.nic.in/
[39] Poverty Reduction and Social Development Department. A User's Guide to Poverty and Social Impact Analysis. Washington: World Bank;2003
[40] Gertler P, Martinez S, Premand P, Rawlings L, Vermeersch C. Impact Evaluation in Practice. Washington: World Bank; 2011.
[41] Selvaraj S, Karan A. Why Publicly-Financed Health Insurance Schemes Are Ineffective in Providing Financial Risk Protection. Economic and Political Weekly 2012 March 17; XLVII(11):60-68
[42] King G, Gakidou E, Imai K, Lakin J, Moore RT, Nall C. Public policy for the poor? A randomised assessment of the Mexican universal health programme. Lancet. 2009 Apr 8; DOI:10.1016/S0140-6736(09)60239-7.
[43] Xu K, Evans DB, Kawabata K, Zeramdini R, Klavus J, Murray CJ. Household catastrophic health expenditure: a multicountry analysis. Lancet. 2003 Jul 12;362(9378):111-7.
[44] Naga Sridhar G. Microfinance institutions: Moving beyond AP crisis. The Business Line [Newspaper Online]. 2012 Dec 29. Available from: http://www.thehindubusinessline.com/companies/microfinance-institutions-moving-beyond-ap-crisis/article4253239.ece
[45] Naga Sridhar G. Life after microfinance in AP. The Business Line [Newspaper Online]. 2013 Jan 4. Available from: http://www.thehindubusinessline.com/opinion/life-after-microfinance-in-ap/article4273183.ece
[46] Prasad N P, Raghavendra P. Healthcare models in the era of medical neoliberalism. A study of Aarogyasri in Andhra Pradesh. Economic and Political Weekly 2012 Oct 27;XLVII(43):118-126
[47] Health Systems 20-20. An Evaluation of the Effects of the National Health Insurance Scheme in Ghana. Bethesda: Health Systems 20-20. 2009.
[48] http://www.jointlearningnetwork.org/programs/compare/benefits/142%2C16
[49] Rajiv Jeevandayi scheme details. http://www.jeevandayee.gov.in/RGJAY/FrontServlet?requestType=CommonRH&actionVal=RightFrame&page=undefined%3E%3E%3Cb%3ERGJAY%3C/b%3E&pageName=RGJAY&mainMenu=About&subMenu=RGJAY (accessed on Apr 30 2013)
[50] Tangcharoensathien V, Swasdiworn W, Jongudomsuk P et al . Universal Coverage Scheme in Thailand: Equity Outcomes and Future Agendas to Meet Challenges. Geneva: World Health Organization. 2010.
[51] Dilip TR. On Publicy-Financed Health Insurance Schemes. Is the analysis premature? Economic and Political Weekly. 2012 May 5; XLVII(1)8:79-80.

For peer review only - http://bmjopen.bmj.com/site/about/guidelines.xhtml

[52] Vallakal S, Ebrahim S. Publicly-Financed Health Insurance Schemes. Concerns about Impact Assessment. Economic and Political Weekly. 2013 Jan 5; XLVIII(1);24-27.

For peer review only

For peer review only - http://bmjopen.bmj.com/site/about/guidelines.xhtml

STROBE Statement—Checklist of items that should be included in reports of ***cross-sectional studies***

	Item No	Recommendation
**Title and abstract**	1	(*a*) Indicate the study's design with a commonly used term in the title or the abstract Yes
		(*b*) Provide in the abstract an informative and balanced summary of what was done and what was found Yes
**Introduction**		
Background/rationale	2	Explain the scientific background and rationale for the investigation being reported Yes
Objectives	3	State specific objectives, including any prespecified hypotheses Yes
**Methods**		
Study design	4	Present key elements of study design early in the paper Yes
Setting	5	Describe the setting, locations, and relevant dates, including periods of recruitment, exposure, follow-up, and data collection Yes
Participants	6	(*a*) Give the eligibility criteria, and the sources and methods of selection of participants Yes
Variables	7	Clearly define all outcomes, exposures, predictors, potential confounders, and effect modifiers. Give diagnostic criteria, if applicable Yes
Data sources/ measurement	8*	For each variable of interest, give sources of data and details of methods of assessment (measurement). Describe comparability of assessment methods if there is more than one group Yes
Bias	9	Describe any efforts to address potential sources of bias Yes
Study size	10	Explain how the study size was arrived at Yes
Quantitative variables	11	Explain how quantitative variables were handled in the analyses. If applicable, describe which groupings were chosen and why Yes
Statistical methods	12	(*a*) Describe all statistical methods, including those used to control for confounding Yes
		(*b*) Describe any methods used to examine subgroups and interactions
		(*c*) Explain how missing data were addressed Method identical to baseline NSSO survey
		(*d*) If applicable, describe analytical methods taking account of sampling strategy
		(*e*) Describe any sensitivity analyses
**Results**		
Participants	13*	(a) Report numbers of individuals at each stage of study—eg numbers potentially eligible, examined for eligibility, confirmed eligible, included in the study, completing follow-up, and analysed Yes
		(b) Give reasons for non-participation at each stage
		(c) Consider use of a flow diagram
Descriptive data	14*	(a) Give characteristics of study participants (eg demographic, clinical, social) and information on exposures and potential confounders Yes
		(b) Indicate number of participants with missing data for each variable of interest
Outcome data	15*	Report numbers of outcome events or summary measures Yes
Main results	16	(*a*) Give unadjusted estimates and, if applicable, confounder-adjusted estimates and their precision (eg, 95% confidence interval). Make clear which confounders were adjusted for and why they were included Yes
		(*b*) Report category boundaries when continuous variables were categorized

For peer review only - http://bmjopen.bmj.com/site/about/guidelines.xhtml

		(*c*) If relevant, consider translating estimates of relative risk into absolute risk for a meaningful time period
Other analyses	17	Report other analyses done—eg analyses of subgroups and interactions, and sensitivity analyses Yes
**Discussion**		
Key results	18	Summarise key results with reference to study objectives Yes
Limitations	19	Discuss limitations of the study, taking into account sources of potential bias or imprecision. Discuss both direction and magnitude of any potential bias Yes
Interpretation	20	Give a cautious overall interpretation of results considering objectives, limitations, multiplicity of analyses, results from similar studies, and other relevant evidence Yes
Generalisability	21	Discuss the generalisability (external validity) of the study results Yes
**Other information**		
Funding	22	Give the source of funding and the role of the funders for the present study and, if applicable, for the original study on which the present article is based Yes

*Give information separately for exposed and unexposed groups.

Note: An Explanation and Elaboration article discusses each checklist item and gives methodological background and published examples of transparent reporting. The STROBE checklist is best used in conjunction with this article (freely available on the Web sites of PLoS Medicine at http://www.plosmedicine.org/, Annals of Internal Medicine at http://www.annals.org/, and Epidemiology at http://www.epidem.com/). Information on the STROBE Initiative is available at www.strobe-statement.org.

For peer review only

For peer review only - http://bmjopen.bmj.com/site/about/guidelines.xhtml

BMJ Open

Changes in addressing inequalities in access to hospital care in Andhra Pradesh and Maharashtra states of India. A cross sectional difference-in-differences study

Journal:	*BMJ Open*
Manuscript ID:	bmjopen-2013-004471.R2
Article Type:	Research
Date Submitted by the Author:	06-May-2014
Complete List of Authors:	Rao, Mala; University of East London, Institute for Health and Human Development<br>Katyal, Anuradha; ACCESSHealth International,<br>Singh, Prabal; ACCESSHealth International,<br>Samarth, Amit; Sughavazhvu Healthcare,<br>Bergkvist, Sofi; ACCESSHealth International,<br>Kancharla, Manjusha; Indian School of Business,<br>Wagstaff, Adam; World Bank, Development Research Group<br>Netuveli, Gopalakrishnan; University of East London, Institute for Health and Human Development<br>Renton, Adrian; University of East London, Institute for Health and Human Development
**Primary Subject Heading**:	Public health
Secondary Subject Heading:	Health services research
Keywords:	Health economics < HEALTH SERVICES ADMINISTRATION & MANAGEMENT, Health policy < HEALTH SERVICES ADMINISTRATION & MANAGEMENT, PUBLIC HEALTH

SCHOLARONE™ Manuscripts

TITLE PAGE

TITLE:

Changes in addressing inequalities in access to hospital care in Andhra Pradesh and Maharashtra states of India. *A cross sectional difference-in-differences study*

STUDY DESIGN: A cross-sectional difference in differences study with parallel control.

AUTHOR NAMES, ADDRESSES AND POSITIONS:

1.Mala Rao. Professor of International Health, Institute for Health and Human Development, University of East London, UK & Honorary Adviser, Administrative Staff College of India, Hyderabad

2. Anuradha Katyal, Consultant, ACCESS Health International, Hyderabad, India

3.Prabal V Singh, Director Research, ACCESS Health International, Hyderabad, India

4.Amit Samarth, Head, Strategy and Planning, SughaVazhvu Healthcare, Thanjavur, India

5.Sofi Bergkvist, Managing Director, ACCESS Health International, Hyderabad, India

6.Manjusha Kancharla, Research Associate, Indian School of Business, Hyderabad India

7.Adam Wagstaff, Research Manager, Human Development & Public Services team, Development Research Group (DECRG), The World Bank, Washington DC, USA

8.Gopalakrishnan Netuveli. Professor of Public Health, Institute for Health and Human Development, University of East London & ESRC International Centre for Life Course Studies in Society and Health, University College London, UK

9.Adrian Renton. Director, Institute for Health and Human Development, University of East London, UK

All authors had full access to all the data (including statistical reports and tables) and can take responsibility for the integrity of the data and the accuracy of the data analysis

Corresponding author and guarantor of the data and information: Mala Rao (best email address: m.rao@uel.ac.uk).

M Rao affirms that the manuscript is an honest, accurate and transparent account of the study being reported and no important aspects of the study have been omitted.

FUNDED BY : Wellcome Trust, IDRC, Rockefeller Foundation, DFID, World Bank.

ROLE OF THE FUNDING SOURCES: The external funding sources had no role in study design, data collection, analysis, interpretation or reporting, or in submission decision.

ROLE OF THE STUDY SPONSOR: The study was carried out in response to the formal invitation of the Government of Andhra Pradesh which supported our applications for funding (by providing formal

For peer review only - http://bmjopen.bmj.com/site/about/guidelines.xhtml

For peer review only

letters of support). The Government did not provide any funding, and had no role in study design, data collection, analysis, interpretation or reporting, or in submission decision.

STATEMENT OF INDEPENDENCE OF RESEARCHERS: The authors can confirm that they worked independently from the Funders and Government of Andhra Pradesh who had no role in any aspect of the research

CONFLICT OF INTEREST

All authors have completed the ICMJE forms for disclosure of potential conflicts of interest (available on request from the corresponding author) and declare that 1) MR, AK, PS, AS, and SB have support from the Rockefeller Foundation, Wellcome Trust, International Development Research Centre, Canada and Department for International Development, UK, 2) the authors have no relationships with the funders that might have an interest in the submitted work in the previous 3 years, 3) the authors' spouses, partners, or children have no financial relationships that may be relevant to the submitted work, and 4) the authors have no non-financial interests that may be relevant to the submitted work.

ETHICAL APPROVAL: given by the research ethics committee of the Administrative Staff College of India, Hyderabad, which hosted the study. Household survey questionnaires include signed consent by the head of the household or another adult representative of the household.

DATA SHARING STATEMENT: The 2004 National Sample Survey Organisation of India household survey questionnaire and data are available to the public. The 2012 household survey questionnaire, and the full anonymised household survey dataset will be available with open access as soon as the data analyses and submissions for publication are completed.

COPYRIGHT STATEMENT: Mala Rao, the Corresponding Author has the right to grant on behalf of all authors and does grant on behalf of all authors, an exclusive licence on a worldwide basis to the BMJ Publishing Group Ltd to permit this article (if accepted) to be published in BMJ editions and any other BMJPGL products and sublicences such use and exploit all subsidiary rights, as set out in your licence.

STROBE CHECKLIST FOR CROSS SECTIONAL STUDIES: We can confirm that all 22 items in the STROBE checklist are included in the paper.

CONTRIBUTORS' STATEMENT

MR conceived and designed the study, applied for funding, and was responsible for the supervision and management of all aspects of the study as well as the dissemination of its results. She is also the guarantor of the study. SB shared responsibility for the conception of the study, applications for funding, study design and data collation and analysis, contributed to the questionnaire design and commented on drafts of the report. PS contributed to the conception of the study and study design, led the questionnaire design and survey implementation, including training of survey staff, monitoring survey progress and data collation and verification, commented on drafts of the report and helped prepare the references. AK undertook the data collation, verification and analysis, assisted with the survey and questionnaire design and survey implementation and prepared the

For peer review only - http://bmjopen.bmj.com/site/about/guidelines.xhtml

For peer review only

tables for the report. AS led the literature review, assisted with the study and questionnaire design, survey implementation and preparation and analysis of baseline data, and commented on drafts of the report. MK helped with the data analysis. AW devised the methodology for the estimation of the programme impacts, advised during the data-collection and data-preparation stages, wrote and implemented the computer code for the model estimation, helped to oversee the production of the results, and contributed text to the report. GN provided technical advice on accounting for the complex survey structure in the analysis, developed a STATA equation, helped to compute an asset index, advised on the output tables, verified the analysis and commented on drafts of the report. AR helped develop a conceptual framework for the evaluation, advised on funding proposals, the study design, analytical methodology and presentation of results and contributed text to the report. MR wrote the first draft of the paper and its redrafts in accordance with the comments of all other authors and reviewers.

For peer review only

For peer review only - http://bmjopen.bmj.com/site/about/guidelines.xhtml

ABSTRACT. WORD COUNT: 300

OBJECTIVES

To compare the effects of the Rajiv Aarogyasri Health Insurance Scheme of Andhra Pradesh (AP) with health financing innovations including the RSBY in Maharashtra (MH) over time on access to and out of pocket expenditure (OOPE) on hospital inpatient care.

STUDY DESIGN

A cross-sectional difference in differences (DID) study with parallel control.

SETTING

National Sample Survey Organisation of India (NSSO) urban and rural 'first stratum units', 863 in AP and 1008 in MH.

METHODS

We used two cross-sectional surveys: as a baseline, the data from the NSSO 2004 survey collected before the Aarogyasri and RSBY schemes were launched and as post-intervention, a survey using the same methodology conducted in 2012.

PARTICIPANTS

8623 households in AP and 10073 in MH

MAIN OUTCOME MEASURES

Average OOPE, large OOPE and large borrowing per household per year for inpatient care, hospitalisation rate per 1000 population per year.

RESULTS

Average expenditure, large expenditures and large borrowings on inpatient care had increased in both MH and AP, but the increase was smaller in AP across these 3 measures. DIDs for average expenditure and large borrowings were significant and in favour of AP for the rural and the poorest households. Hospitalisation rates also increased in both states but more so in AP, although the DID was not significant and the sub group analysis presented a mixed picture.

CONCLUSIONS

Health innovations in AP had a greater beneficial effect on inpatient care-related expenditures than innovations in MH. The Aarogyasri scheme is likely to have contributed to these impacts in AP at least in part. However, out of pocket expenditure increased in both states over time. Schemes such as the Aarogyasri and RSBY may result in some positive outcomes but, additional interventions may be required, to improve access to care for the most vulnerable sections of the population.

For peer review only - http://bmjopen.bmj.com/site/about/guidelines.xhtml

For peer review only

ARTICLE SUMMARY

STRENGTHS AND LIMITATIONS OF THE STUDY

- This study uses 2 cross-sectional surveys to compare changes between the Indian states of Andhra Pradesh and Maharashtra, in hospital inpatient care related expenditures and behaviours before and after the rollout of the Aarogyasri and RSBY schemes
- The study based on a survey of 18696 households has shown that health innovations in Andhra Pradesh had a greater beneficial effect on hospital inpatient care-related expenditures and access than innovations in Maharashtra. The Aarogyasri scheme is likely to have contributed to these impacts in Andhra Pradesh.
- The study also highlights the implications of the findings for policy and practice and additional interventions necessary to address gaps in the availability of and access to care.
- The study is only able to compare the effects of health innovations over time across the 2 states but does not allow the drawing of inferences on the impacts of individual initiatives.
- The study uses the difference in differences methodology and its findings may have been affected by unobservable differential changes between the 2 states.

For peer review only

ACKNOWLEDGEMENTS:

The study was funded by the International Development Research Centre, Canada, the Wellcome Trust, the UK Department for International Development, and Rockefeller Foundation. The World Bank supported Adam Wagstaff's contribution to the study. We gratefully acknowledge the financial support of these organisations, as well as the enthusiastic encouragement we have received from them throughout the study. Our thanks also go to Bhimasankaram Pochiraju, Sundaresh Peri, C Ravi, and Rahul Ahluwalia for their advice and guidance at various stages of the study design and data analysis, and to colleagues at the University of East London and Administrative Staff College of India Hyderabad for their administrative support. The authors also thank P Suryanarayana, CM Reddy, D Chakrapani and K Pandu Ranga Reddy and AY Jadhav and his colleagues for their contribution to the training of the survey teams, verification of the survey and data collection and acknowledge the IMRB International Social Research Institute team's support in carrying out the survey.

We are grateful to Somil Nagpal and Joseph Kutzin for commenting on the report and helping us to improve it, to Sujatha Rao for her encouragement and valuable suggestions and ideas throughout the course of the study and to Jay Bagaria for her help during the early stage of the development of the proposal. Finally we thank PV Ramesh without whose constant support, guidance and encouragement, this study would not have been possible.

For peer review only

INTRODUCTION

In 2005, member states of the World Health Organization (WHO) committed to develop their health financing systems to deliver universal coverage (UC); that is that all people have access to health services and do not suffer financial hardship paying for them[1] . WHO's 2010 World Health Report[2] recommended *inter alia* that countries reduce reliance on direct payments, and improve equity of access, including through the introduction of prepayment schemes. However, one recent systematic review of the impact of national health insurance schemes in low and middle income countries[3] found only weak evidence of increased use of healthcare and reduced out-of-pocket expenses, with the poorer benefitting less, whilst others[4,5] concluded that health insurance improved health care access and use, as well as financial protection in most cases but had no conclusive impact on health status. Both highlighted the need for more rigorous assessments of such schemes.

India has one of the highest levels of out-of-pocket health expenditure (more than 80% of private health expenditure)[6]. The aim of our study was to explore how recently introduced health financing initiatives have affected access to and out-of-pocket expenditure (OOPE) on inpatient hospital care in the Indian states of Andhra Pradesh (AP) and Maharashtra (MH).

BACKGROUND

In its *Eleventh Five Year Plan (2007-12)*[7], Government of India sought to increase Public Expenditure on Health and to strengthen investment in rural health infrastructure through the National Rural Health Mission. Its *Twelfth Five Year Plan (2012-17)*[8], reflected the recommendation of the Planning Commission's High Level Expert Group for general taxation to be the principal source of health care financing. It proposed the development of government-funded health insurance schemes, building on the evidence from experimental schemes being introduced across many States. In India, health is primarily a state rather than national responsibility[6]. Whilst it is recognised that political will and good governance are essential, both the political mobilization of funds for health schemes and the effectiveness and efficiency of funded schemes will be enhanced by robust evidence which documents as to whether schemes achieve objectives, what works well and the main challenges they face.

In India, evaluation is not routine, even of large costly public health care programmes. Nevertheless, some assessments have been carried out, for example of the Yeshasvini Co-operative Farmers Health Care Scheme of Karnataka, the longest running state-supported health insurance scheme for the informal sector in India[9], with promising results in terms of increased utilization of and reduced borrowing for health care services[10]. However, these early models covered small populations and offered limited benefits, so that the policy implications of conclusions drawn from even the best evaluations were unclear.

This scenario has changed during the past 5 years, with the launch of 2 schemes, the Rajiv Aarogyasri[11] Community Health Insurance Scheme (Aarogyasri) of Andhra Pradesh (AP) and Rashtriya Swasthya Bima Yojana (RSBY) currently offered in 30 states and union territories of India[12] including AP's neighbouring state of Maharashtra (MH). Their scale in terms of population coverage and range of treatments offered, significantly enhances their potential to inform India's road map towards universal health coverage. Both schemes belong to the new generation of publicly funded government-sponsored health insurance schemes, principally aimed at providing financial protection to the poor against catastrophic health shocks, which, for these schemes, the Government has defined as inpatient hospital care[9]. Both schemes have been subject to some assessment in their early phases, but a recent editorial[13] highlighted the necessity of further and repeated evaluations to enhance the credibility and accountability of existing schemes and to identify those which deserve scale-up. The Aarogyasri and RSBY schemes and the other recent health sector innovations in AP and

For peer review only - http://bmjopen.bmj.com/site/about/guidelines.xhtml

Maharashtra which form the major backdrop and complex health care architecture against which these have been launched, and which may have contributed to the changes in outcomes we have explored in our study, are described below.

The Rajiv Aarogyasri Health Insurance Scheme of AP

In 2007 AP launched a pioneering new state-wide fully state-funded health insurance scheme, the Rajiv Aarogyasri Community Health Insurance Scheme (Aarogyasri)[11] to provide treatment for serious and life-threatening illnesses. The specific objectives include: to improve access of poor families to quality medical care (meaning low-frequency, high cost specialist care) and treatment of identified diseases requiring hospitalisation through an identified network of health care providers, to provide financial cover for catastrophic illnesses which have the potential to wipe out life time savings of poor families and to provide 'universal coverage to the urban and the rural poor in the state[14] albeit for the conditions covered in the benefits package. All families with a 'below poverty line' (BPL) ration card, i.e. those on an annual income below USD 1384 (INR 75,000) in urban areas and USD 1107 (INR 60,000) in rural areas, and including individuals with pre-existing medical conditions are *automatically* enrolled and the scheme was estimated to cover approximately 20.4 million poor and lower middle class families, comprising about 85 percent of the state's population in 2009[9]. Enrollees make no contribution, the annual benefit is a maximum of USD 4,500 (INR 200,000) per family per year and there is no limit on the size of the family[14]. A total of 942 medical and surgical procedures across 31 clinical specialties[14] are provided and the benefits include all inpatient costs - associated investigations, food, transport and medicines for 10 days following discharge. One year follow-up packages including consultation, medicines, and diagnostics are also available for 125 procedures requiring longer periods of follow up[9] . Aarogyasri has unique features including *Aarogyamithras* (health system navigators), outreach *health camps* delivered by participating hospitals to educate, screen and case-find and a state-of-the-art information technology-based management system. At the time of this study, 353 public and private sector hospitals were 'empanelled' to provide services to Aarogyasri beneficiaries.

In 2009, a descriptive study of *Aarogyasri*, based on an analysis of claims data and a survey of beneficiaries[15], concluded that while the scheme was beginning to reach its intended beneficiaries uptake was lower among scheduled castes and tribes. This was confirmed by Fan and colleagues[16], who used variations in programme roll-out over time and districts to evaluate the scheme using National Sample Survey data collected before and after its launch. They reported reduced out-of-pocket expenditure in this initial phase but no major impact on catastrophic healthcare expenditure. Inspired by Aarogyasri and mindful of the political benefits of introducing popular health reforms, other states have launched health financing innovations similar to this model.

RSBY in Maharashtra

RSBY was launched across a number of states by the Ministry of Labour, Government of India (GOI) in 2008[17] and provides access to free inpatient hospital care up to USD 550 (INR 30,000) per family per year[18]. Households which meet the criteria based on the much more limiting definition of poverty and numbers of poor families provided for each State by the GOI Planning Commission are eligible to enrol, and pay a contribution of USD 0.55 (INR 30) at registration and at each annual renewal[9]. Up to 5 family members, including those with pre-existing conditions can be covered, and personal information including biometric data are collected prior to the issue of a smart card with encoded details of the family. 700 procedures covering 18 broad categories of interventions which would generally be included under the umbrella of 'secondary' care, are provided and the benefit packages include the intervention, public transport costs limited to 1.8 USD (INR 100) per visit and 18.2 USD (INR 1000) per year and post-hospitalization drugs for 5 days. Networked hospitals are required to provide free outpatient consultations (which have only recently been introduced. Personal communication, Kurian OC) but other costs such as ambulatory diagnostics and medicines

For peer review only - http://bmjopen.bmj.com/site/about/guidelines.xhtml

have to be borne by the beneficiaries, except if investigations lead to inpatient admissions within a day[9]. A pilot of the RSBY scheme was launched in 1 district of AP, but only after the start of our household survey.

In Maharashtra, enrolment began in Aug 2009, and by mid 2013, approximately 2 million of the eligible 4 million families were enrolled in the scheme[12] which is being implemented in 31 of 35 districts in the state. Enrolment had extended to 26 districts prior to June 2012, when our household survey began. Notably, only 15 out of 1215 hospitals contracted for RSBY funded services are from the public sector[12]. Early assessments of the scheme's impact nationally, suggest that although the rate of hospitalisations has increased, awareness of the scheme was poor and remains a barrier to uptake. Notable variations in enrolment and scheme awareness were also observed by a descriptive study of RSBY conducted in the Amravati district of Maharashtra which has a large tribal population[19]. In Maharashtra[17], utilisation rates have been reported to be lower than in other states and the male:female enrolment ratio is 6.5:3.5.

Other major health sector initiatives in AP and Maharashtra

Both states have a complex health care landscape with numerous programmes in place. There are several initiatives launched during the past decade, some of which are common to both states and driven by national strategies, and others owe their existence to state-level enterprise, innovation and political support. The most notable programmes with the potential to impact on in patient care are described below.

The National Rural Health Mission (NRHM) was launched in 2005 nationwide, with a key aim of reducing maternal and infant mortality[20]. Government reports suggest that its notable achievements include an increase in institutional deliveries; in AP from 1.25 million in 2005-06 to 1.46 million by 2011-12[21] and in MH from 1.1 million to 1.63 million[22], achieving an institutional delivery rate of approximately 92 percent in both states. Also common to both states is the '104 health information help line' launched in AP in 2008 and MH in 2011[23], to provide medical advice and information based on validated algorithms and disease summaries, direct callers to appropriate health facilities or to receive a complaint against a public sector health facility. In AP the help line and call centre were subsumed within the Aarogyasri infrastructure by 2011.

In MH, the RSBY was preceded by the Jeevandayee scheme launched in 1997 with the objective of reducing catastrophic OOPE on inpatient care in the BPL population[24]. Potential beneficiaries were required to apply for funding after a diagnosis was confirmed and the scheme covered serious illness such as cardiac and renal disease and cancer. However, the scheme uptake has been low, and while it has continued to run in parallel to the RSBY, only 66,853 procedures (4456 procedures per year in a state with 112.37 million people) have been approved during the scheme's lifetime[25]. Since 2006, MH has also had a scheme in place which mandated 20% of the beds in private hospitals to be made available for free or at subsidized rates to poor patients (personal communication, Kurian OC). It has been estimated that around 10,000 private beds are available for the poor across MH, equivalent to approximately 20% of the total bed capacity of the public sector. Although the implementation of the scheme is reported to be erratic (Kurian OC), it may have had some positive impact on access to hospital inpatient care for serious illness.

Launched in 1995-96, the Navasanjeevani Yojana scheme is exclusive to the 15 tribal districts of MH and was to improve maternal and infant mortality in these vulnerable populations[26]. It has focused on strengthening primary health and nutrition services and access to safe drinking water.

A service available in AP but not in MH is the '108' scheme, launched in 2005 to provide a state-of-the-art medical emergency response service[27]. At the time of our study, 802 ambulances catered to approximately 3,500 emergencies per day[28].

For peer review only - http://bmjopen.bmj.com/site/about/guidelines.xhtml

OBJECTIVES OF THE STUDY

Our objective was to compare the effects of health innovations over time on access to and OOPE on inpatient care in AP and MH and to assess whether the AP initiatives had larger or smaller beneficial effects than those found in MH. These differential effects are likely to be substantially due to the Aarogyasri scheme in AP and the RSBY in MH. In this paper, we report findings from a study which compared these changes. The findings do not allow us to draw inferences on the impacts of individual initiatives, but nevertheless contribute new knowledge on the impact and role of the innovations, provide lessons for other programmes, and strengthen the evidence base for policy on UC in India.

METHODS

Overview

None of the aforementioned initiatives – including the Aarogyasri and RSBY schemes in which we are especially interested – was piloted in a systematic way, let alone via a carefully designed randomized control trial. Following MRC Guidance[29,30] and best practice we therefore opted for a cross-sectional survey design in which we seek to minimise selection bias, to control for confounding variables and to reduce the effects of chance. Specifically, we compare changes in hospital inpatient care related expenditures and behaviours (HREB) in AP and MH before and after the rollout of the Aarogyasri scheme in AP and the RSBY scheme in MH. The difference in changes between AP and MH is not an estimate of a specific initiative. Rather it tells us whether, on balance, the AP initiatives have had larger (or smaller) beneficial effects than the MH initiatives, and if so how much more (or less) beneficial they have been. Since the NRHM was common to both states and the MH-specific initiatives were quite small in scale or unlikely to affect HREBs, any difference in change between the two states is quite likely to be mainly due to differential effects of the Aarogyasri and RSBY programmes.

AP and MH have a broadly similar development profile as shown by the data below. AP's other socio-economically similar neighbouring states of Karnataka and Tamil Nadu had already introduced Aarogyasri-like schemes, and Odisha and Chattisgarh the only other neighbours, had comparatively higher levels of socio-economic deprivation. HREBs were measured in both AP and MH by two waves of household survey before (2004) and after (2012) the introduction of Aarogyasri and RSBY. The study protocol and questionnaire for the 2012 survey were reviewed and agreed by the Research Ethics Committee of the Administrative Staff College of India, Hyderabad.

Maharashtra	Indicator	Andhra Pradesh
112.37	Population (2011 census, in millions)	84.66
10.20	% Schedule Caste (2001 Census)*	16.60
8.90	% Schedule tribe (2001 census)*	6.20
101,314	Per capita income 2011-12 (in INR)**	71,540
35	Number of districts	23
5,314	Households covered in NSSO 60th round (2004-05)	5,059
10,073	Households covered in the study 2012	8,623

For peer review only - http://bmjopen.bmj.com/site/about/guidelines.xhtml

*Note that 2011 census data for social groups are not yet published

**Source: Presentation on Annual Plan 2012-13 and Five Year Plan 2012-17[31]

Survey design

Baseline Survey : 2004

We used the original data from the National Sample Survey Organization (NSSO) 60th decennial round household survey undertaken in 2004[32] to estimate baseline HREB estimates for AP and MH (Table 1). This was the most recent round measuring morbidity profiles, use of health care services including hospitalised and non-hospitalised treatments and expenditures incurred. The household survey used a multi-stage stratified sampling methodology to identify a representative random population sample and an interviewer completed questionnaire to obtain measures of HREB along with socio-demographic, household expenditure and other information.

Follow up survey: 2012

We used the same household survey design and methods to collect post-intervention data in AP and MH as those used by NSSO. Briefly, the household survey used a multi-stage stratified sampling methodology with the 'First Stage Units' (FSUs) identical to those used by NSSO in their 66th round (2008-09)[33], the latest round for which FSUs had been mapped. However, the FSUs were not the same as those in NSSO 2004, our baseline survey, rapid urbanisation having changed substantially, the urban-rural landscape of both states and thus the geographical basis for sampling units.

Table 1: Urban and rural populations and households surveyed in 2004 and 2012 in Andhra Pradesh and Maharashtra

	Andhra Pradesh		Maharashtra	
	NSSO 60th round 2004-05	Our survey 2012	NSSO 60th round 2004-05	Our survey 2012
Population	76,210,007*	84,665,533**	96,878,627*	112,372,972**
Urban population	20,808,940*	28,353,745**	41,100,980*	50,827,531**
Rural population	55,401,067*	56,311,788 **	55,777,647*	61,545,441**
Total households (urban)	4,397,138*	6,778,225**	8,403,224*	10,813,928**
Total households (rural)	12,607,167*	14,246,309**	11,173,512*	13,016,652**
Total households	17,004,305*	21,024,534**	19,576,736*	23,830,580**
FSUs (urban)	183	372	267	504
FSU (rural)	325	491	265	504
Total households surveyed (urban)	1824	3715	2664	5038
Total households covered (rural)	3235	4908	2650	5035

*2001 census ** 2011 census

FSU - First Stratum Unit

The NSSO 66th round had 492 rural FSUs in AP, but 1 FSU was found to be uninhabited.

For peer review only - http://bmjopen.bmj.com/site/about/guidelines.xhtml

The interviewer completed household survey questionnaire was pre-tested and then piloted in both states prior to the survey. All respondents provided written informed consent for participation. Questions addressed the following: household composition and socio-demographic characteristics of members, household expenditure, and health expenditure (outpatient and inpatient) and means of its financing, healthcare seeking behaviour, factors affecting access to healthcare and awareness and perceptions of the quality of the *Aarogyasri* scheme (AP only). The survey questions in 2012 were identical to those from the NSSO 2004[34]. Additional questions specific to the Aarogyasri and other relevant schemes were also added.

A survey of 18696 households across 2 states and 1871 locations within the states is a challenging undertaking. The survey design had several features intended to assure the quality of data collected. Few academic institutions have internal capacity to carry out such large surveys, and consequently, the Social and Research Institute of IMRB International, a leading market research agency was selected to carry out the survey. The Institute has field survey teams based in every Indian state, conversant in local languages and dialects and trained to carry out surveys in the socio-economic development sector. Its clients include Government of India (for whom the national Family Health Survey data are collected), World Bank and other UN organisations. A group of NSSO consultants in AP and the Indian Socioeconomic Research Unit, Pune were recruited to support the training of the field survey teams and data verification.

We planned three levels of verification of the study data; the first to be undertaken by the survey agency, the second to be carried out by the study team and the third, by the agencies mentioned above. Survey teams for each district were accountable to a field supervisor who was responsible for checking both the household listing and data entry on a daily basis. The study team also accompanied the field staff to survey sites on a regular basis. Data collected from 250 households in each state (approximately 2.5% of the surveyed households) and 186 of the FSU listings (approximately 10%) were independently verified by the agencies in both the villages and urban blocks in order to ensure that the sampling method and administration of the questionnaire survey were being correctly applied. The data entry was carried out by the Institute using a double entry method and any questionnaires reported incorrect were sent back to the field for re-survey. The research team carried out a final validation and review of the data.

Outcome measures

Average inpatient (IP) expenditure per household per year. Average out-of-pocket expenditure for inpatient care during 1 year prior to the survey was estimated from questionnaire responses for AP and MH from both baseline and follow-up data. Reimbursements for inpatient expenditure were deducted from the total where households had received them.

Large out-of-pocket inpatient expenditure. Because of the limited data on household consumption in the 2004 NSSO health survey we did not estimate 'catastrophic health expenditure'. Instead, we constructed a measure of 'large' out-of-pocket expenditure. The Aarogyasri Health Care Trust data on expenditure incurred by the Government of AP per case in 2012 were examined[14] and the mean was estimated as USD 419 (INR 23,000). A household was deemed to have incurred 'large' expenditure if OOPE for inpatient care was equal to or greater than this threshold.

For peer review only - http://bmjopen.bmj.com/site/about/guidelines.xhtml

Large borrowing. We estimated the total amount borrowed by a household to meet the expenditure of all the inpatient episodes of that family during the previous year. A household was considered to have incurred 'large borrowing' if the borrowing was equal to or exceeded the BPL threshold set by Government of AP: INR 70000 for urban families and INR 65000 for rural households. These prices have been deflated to 2004 levels.

Hospitalisation rate. This was estimated as the number of individuals hospitalised during the previous year, per 1000 population.

Variations in outcomes were examined between male and female-headed households, and rural and urban populations, as well as across social groups and economic groups represented by asset quintiles.

Because of the limited data on household consumption in the 2004 NSSO health survey which made the estimation of wealth difficult, we have opted instead to measure household living standards using an asset or wealth index based on information on ownership of household durables, dwelling type, etc., using principal component analysis to estimate weights[35, 36,37] for each indicator. The indicators used were limited to those collected in the 2004 NSSO: type of structure of the dwelling unit, type of toilet, type of fuel used for cooking and source of drinking. Data from the 2004 and 2012 surveys were pooled so that the index captures changes in living standards between the two years; there are therefore more households in the top quintile in 2012 than in 2004. The statistical software Stata 11 was used to generate the index.

Deflation of follow-up expenditure estimates.
The 2012 expenditure data including the threshold for large expenditures were deflated using the consumer price index of the Government of India[38] to reflect 2004 prices.

Analysis
Our repeat cross-sectional surveys do not allow estimation of within-individual household changes in outcomes over time. Our analysis therefore focused on estimating outcomes averaged across states, and in comparing changes in these over time between AP and MH. If we assume that outcome determinants other than Aarogyasri and RSBY remained stable in the two states over time or followed a parallel change, then a difference-in-differences (DID) analysis will uncover the net effect of Aarogyasri over and above RSBY.

The difference in differences of outcome (Y_{DD}) is

$$(Y^{AP}_{2012} - Y^{AP}_{2004}) - (Y^{MH}_{2012} - Y^{MH}_{2004})$$

where the subscripts and superscripts for Y refer to the respective states and the years when the surveys were done. Confidence intervals were calculated from the standard error Y_{DD} of and the p-value for the null-hypothesis $(Y_{DD} = 0)$ was tested using the Wald test as $t = \frac{Y_{DD}}{SE_{Y_{DD}}}$ with one degree of freedom. Y_{DD} was estimated using ordinary least square regression:

$$y_{it} = \beta_0 + \beta_1 state_i + \beta_2 survey_t + \beta_3 (state * survey)_{it} + \sum_{k=1}^{m} \beta_{3+k} covariate_k + \varepsilon$$

The basic DID results are obtained using the above regression with covariates excluded. The adjusted DID results are obtained using the above regression with m=9 covariates, namely the gender of head of household, a dummy variable capturing whether the household lives in a rural or urban location, three dummy variables capturing the household's social group (the lowest is the excluded category),

For peer review only - http://bmjopen.bmj.com/site/about/guidelines.xhtml

and four asset quintile dummies (the bottom is the excluded category). In the regression y_{it} is the outcome, *state* is a dummy variable with 0 for MH and 1 for AP, and *survey* is a dummy variable with 0 for 2004 survey and 1 for 2012 survey. The coefficient for the interaction term, β_3, gives the differences in differences estimate, Y_{DD}. Robust standard errors of Y_{DD} were calculated to account for clustering of households within FSUs using Stata survey commands. A positive value for Y_{DD} suggested that the change in the outcome in AP was more than the change in MH and the negative value would suggest the reverse.

An advantage of regression based DID estimate is this ability to use co-variates which can account for differential trajectories in the two states. In addition to this we did sub-group analysis stratifying for different co-variates. This is particularly relevant in the case of scheduled tribes whose proportion increased in MH in the follow up survey.

Sub groups were not mutually adjusted for the analysis due to sample size restrictions in relation to some of them.

Role of the funding sources
The external funding sources had no role in study design, data collection, analysis, interpretation or reporting, or in submission decision.

RESULTS

A total of 5314 and 5059 households from Maharashtra and AP were surveyed by the NSSO in 2004 (Table 2). Our survey in 2012 included 10073 (MH) and 8623 (AP) households.

Table 2. Socio-demographic characteristics of baseline and follow-up samples

Subgroups	Number (%) of Households 2004		Number (%) of Households 2012	
	Maharashtra	Andhra Pradesh	Maharashtra	Andhra Pradesh
**All**	5314	5059	10073	8623
**Head of Household**				
Male	4785 (90.0)	4433(87.6)	8543(84.8)	7418(86.0)
Female	529 (10.0)	626 (12.4)	1530 (15.2)	1205 (14.0)
**Social Group**				
Scheduled Tribes	413 (7.8)	296 (5.9)	1364 (13.5)	883 (10.2)
Scheduled Castes	809 (15.2)	974 (19.3)	2235 (22.2)	1797 (20.8)
Other Excluded	1644 (30.9)	2317 (45.8)	1899 (18.9)	3419 (39.7)

For peer review only - http://bmjopen.bmj.com/site/about/guidelines.xhtml

All other Groups	2448 (46.1)	1472 (29.1)	4571 (45.4)	2524(29.3)
**Location**				
Rural	2,650 (49.9)	3235 (63.9)	5035(50.0)	4908 (57.0)
Urban	2664 (50.1)	1824 (36.1)	5038 (50.0)	3715(43.0)
**Asset Quintile**				
Lowest	1,260 (23.7)	1,594(31.5)	996(9.9)	826(9.6)
Second	1,016 (19.1)	1,237 (24.5)	1,841(18.2)	1,286(14.9)
Third	772 (14.5)	753(14.9)	2,228(22.1)	2,121(24.60)
Fourth	857 (16.1)	744(14.7)	2,373 (23.6)	3,072(35.6)
Fifth	1,408(26.5)	730(14.4)	2,633(26.1)	1,318(15.3)

Changes in average in-patient expenditure

Table 3 (top panel) shows average baseline levels of inpatient expenditure. The table also shows the real terms change (deflated to 2004 prices) in these outcomes at follow up and the difference in differences (DID) estimate comparing AP with MH. DIDs for overall results are shown unadjusted, as well as adjusted for the effects of the covariates. Breakdowns by sex of head of household, social group, urban/rural location and asset quintiles are also shown.

Overall, average inpatient expenditure increased in real terms in both the states between 2004 and 2012, but the increase was significantly greater in MH (unadjusted DID= -498.2 INR, 95% CI= -792.9: -203.5, p=0.0009). The direction in terms of a greater increase in MH was evident across all sub groups of analysis except the richest asset quintile. However, the DIDs reached significance in male headed households (DID=-513.7INR, 95% CI= -843.9: -183.4, p=0.0023), scheduled castes (DID= -708.7INR , 95% CI=-1234.3: -183.2, p=0.0082), all 'other' social groups (DID=-1110.46INR, 95% CI=-1868 : -352.9, p=0.0041), rural households (DID= -504 INR, 95% CI= -801.9: -206.0, p=0.0009) and the poorest (DID= -1001.3 INR, 95% CI= -1751: -251.7, p= 0.0089) and middle asset quintiles (DID= -798.1 INR, 95% CI= -1362.9:-233.3, p= 0.0056).

Table 3: Change in average inpatient expenditure (in INR) in Maharashtra and Andhra Pradesh between 2004 and 2012

**Subgroups**	**Baseline Mean (95% CI)**		**Change 2004:2012 Mean (95% CI)**		**DID**	
	**Maharashtra**	**Andhra Pradesh**	**Maharashtra**	**Andhra Pradesh**	**Mean (95%CI)**	**p**
**Household inpatient expenditure**						
**All**	1091.6(978.4:1204.8)	723.5(527.5:919.5)	942.8(749.9:1135.6)	444.55(221.5: 667.6)	-498.2(-792.9:-203.5)	0.0009
					**DID (adjusted for covariates)**	
					-565.8(862.9: -268.6)	0.0002

For peer review only

**Head of Household**						
Male	1,132.9(1015:1,251)	758(419.7:1,096.3)	935(727:1,143.01)	1074.9(555.9:1593.8)	-513.7(-843.9: -183.4)	0.0023
Female	757.1(555.5:1014.6)	341.1(222.3:460.01)	421.3(164.7:678.0)	589.9(307.22:872.8)	-484.9(-1075.6:105.9)	0.1076
**Social Group**						
Scheduled Tribes	376.6(231.7:521.6)	432.7(212.52:652.9)	1153.1(803.3:1502.9)	675.2(163.2:1187.2)	-477.9(-1097.7:142)	0.1307
Scheduled Castes	696.7(500.2:893.2)	432.6(305.6:559.4)	1464.1(1039.9:1888.4)	755.4(444.9:1065.9)	-708.7(-1234.3: -183.2)	0.0082
Other Excluded	1,028.6(838.4:1218.8	562.4(463.7:662)	928.9(532.9:1324.9)	767.9(569.6:966.2)	-161(-603.7: 281.7)	0.4758
All other Groups	1,424.5(1,222.3:1626.7)	1306.2(627.8:1984.6)	734.9(427.9:1041.7)	-375.6(-1,068.5:317.4)	-1110.46(-1868 : -352.9)	0.0041
**Location**						
Rural	897.8(768.1:1027.5)	571.4(496.2:646.6)	1084.7(826.3:1343.1)	580.7(432.2:729.2)	-504(-801.9: -206.0)	0.0009
Urban	1343.5(1146.1:1540.9)	1113.5(466.2:1760.8)	753.6(458.7:1048.6)	92.3(-586.92:771.5)	-661.3(-1401.5: 78.864)	0.0799
**Quintile**						
poorest	656.3(498.0:814.6)	391.5(319:464.1)	1692.5(1053.3:2331.7)	691.2(298.9:1083.5)	-1001.3(-1751: -251.7)	0.0089
2nd	786.5(583.5:989.5)	443.3(356.5:530.2)	979.3(599.4:1359.2)	839.5(465.7:1213.3)	-139.8(-672.5:393)	0.607
middle	1062.7(738.8:1386.1)	862.1(577.8:1146.5)	1011.8(550.2:1473.4)	213.7(-112.1:539.6)	-798.1(-1362.9: -233.3)	0.0056
4th	1241.7(894.4:1589.1)	1819(337.5:3302.5)	803.6(328.7:1278.5)	-644.3(-2128.3: 839.7)	-1447.9(-3005.2:109.5)	0.0684
richest	1818.6(1505.5:2131.8)	908.3(682.1:1133.4)	252.3(-193.4:698.1)	362.1(15.3:708.9)	109.7(-454.80:674.3)	0.7031

Large expenditures for inpatient care

Proportions of households incurring large expenditures showed an increase in both states (Table 4), but the increase was smaller in AP for the sample as a whole as well as for all the groups except for the second asset quintile. The DID was strongly significant for the households overall (adjusted DID=-1.8, 95% CI: -3:-0.7, p= 0.0009), but this was not observed for any of the sub groups of analysis.

Table 4: Change in the proportion (%) of households incurring large health expenditures for in-patient care in Maharashtra and AP between 2004 and 2012

Subgroups	Baseline Mean (95% CI)	Change 2004:2012 Mean (95% CI)	DID

For peer review only - http://bmjopen.bmj.com/site/about/guidelines.xhtml

	Maharashtra	Andhra Pradesh	Maharashtra	Andhra Pradesh	Mean (95%CI)	p
Large IP expenditure (INR 23000 deflated to 2004 figures)						
**All**	6.7(6:7.3)	3.4(2.9:3.9)	3.1(2.1:4.1)	2.2(1.5:2.8)	-0.91(-2.1:0.27)	0.1302
					DID (adjusted for covariates)	
					-1.8(-3:-0.7)	0.0009
**Head of Household**						
Male	6.8(6.2:7.6)	3.5(3.1:4)	3.1(2.0:4.1)	2.1(1.5:2.9)	-0.8(-2.1:-0.4)	0.1928
Female	5.0(3.7:6.4)	2.8(1.9:3.7)	3.9(2:5.8)	2(6.6:3.4)	-1.8(-4.2:0.50)	0.1222
**Social Group**						
Scheduled Tribes	2.2(1.3:3.1)	1.4(0.5:2.2)	5.3(3.5:7)	3.5(1.9:5.1)	-1.7(-4.1:0.61)	0.1478
Scheduled Castes	6.1(4.6:7.7)	2.1(1.5:2.6)	4.3(2.3:6.3)	3(1.9:4.1)	-1.2(-3.5 :1.01)	0.2785
Other Excluded	5.9(4.7:7.0)	3.3(2.7:3.8)	2.9(1.1:4.6)	2.7(1.7:3.6)	-2.1(-2.2:1.8)	0.8389
All other Groups	8.3(7.5:7.9)	5.4(4.4:6.3)	2.2(0.9:3.6)	0.51(-0.7:1.7)	-1.7(-3.5:0.04)	0.0628
**Location**						
Rural	1.9(1.5:2.2)	0.9(0.79:1.1)	1.7(1.1:2.3)	1.3(0.09:1.6)	-0.45(-1.1:0.25)	0.2098
Urban	12.9(11.9:14)	9.7(8.9:10.7)	4.4(3.0:5.7)	3.9(2.6:5.3)	-0.7(-2.4: 1.5)	0.6350
**Quintile**						
poorest	1.8(1.3:2.4)	1.1(0.8:1.4)	3.7(2.2:5.2)	1.7(0.7:2.7)	-0.2(-3.8:-0.19)	0.0307
2nd	2.7(1.9:3.5)	1.2(0.9:1.5)	2.1(0.93:3.3)	2.2(1.4:3.1)	0.9(-1.4:1.6)	0.9079
middle	6.9(4.9:8.9)	4.2(3.1:5.3)	1.3(-1:3.6)	0.9(-1.2:1.4)	-1.2(-3.9:1.4)	0.3596
4th	10.7(8.7:12.6)	7.6(5.9: 9.2)	1.8(-0.57:4.3)	-0.036(-1.9:1.80)	-1.9(-4.9:1.2)	0.2268
richest	13.5(12:14.9)	9.6(8.1:11.2)	0.3(-1.6:2.2)	-0.6(-2.8:1.6)	-0.9(-3.7:2)	0.5601

Changes in large borrowing for inpatient care

In both states proportions of households incurring large borrowings to meet inpatient expenses increased from 2004 to 2012 (Table 5). However, there was a consistent pattern of smaller increases in AP for the overall population, as well as all subgroups (except the richest asset quintile) with DIDs strongly significant for the overall population (adjusted DID=-4, 95% CI: -6.6:-1.4, p= 0.0032), scheduled tribes (DID= -5.5, 95% CIs:-9.3:-1, p= 0.0048), rural households (DID= -4.7, 95% CIs: -7.3 : -2.1, p= 0.0007) and all asset quintiles except the richest (the poorest asset quintile DID= -9.0, 95% CI: -14.0: -4.4, p=0.0002).

Table 5: Change in the proportion (%) of households large borrowings for in-patient care in Maharashtra and AP between 2004 and 2012

Subgroups	Baseline Mean (95% CI)		Change 2004:2012 Mean (95% CI)		DID	
	Maharashtra	Andhra Pradesh	Maharashtra	Andhra Pradesh	Mean (95%CI)	p
Proportion of households having large borrowings						
**All**	7.5(6.7:8.2)	3.8(3:4.5)	8.9(6.8:11)	5.3(3.4:7.2)	-3.7(-6.4:-0.908)	0.0100

For peer review only - http://bmjopen.bmj.com/site/about/guidelines.xhtml

					DID (adjusted for covariates)	
					-4(-6.6:-1.4)	0.0032
**Head of Household**						
Male	7.8(7.0:8.5)	3.9(3.1:4.7)	9.8(6.6:1.3)	5.3(3.3:7.3)	-3.6(-6.6: -0.62)	0.0187
Female	5(3:7)	2.9(1.7:4.1)	8.(6.6:11.2)	5(2.9:7.23)	-4.7(-8.3:-1)	0.0137
**Social Group**						
Scheduled Tribes	3.6(2.3:4.9)	2.4(0.93:3.9)	11(8.9:14)	5.8(2.8:8.8)	-5.5(-9.3: -1.8)	0.0048
Scheduled Castes	7.2(5.5:8.8)	3(1.7:4.2)	9.6(6.6:13)	5.8(3.4:8.3)	-3.8(-7.5:0.03)	0.0518
Other Excluded	8.0(6.8:9.2)	3.5(2.7:4.4)	8(5.8:10.3)	5.3(3.2:7.4)	-2.8(-5.7:0.19)	0.0661
All other Groups	8(7.15:8.8)	0.052(.040:0.064)	8.8(5.9:12)	4.7(2.2:7.4)	-4.1(-7.9:-.4.0)	0.0302
**Location**						
Rural	6.5(5.6:7.5)	0.03(0.024:0038)	10(8.5:12)	5·8(3.9:7.6)	-4.7(-7.3:-2.1)	0.0007
Urban	8.7(7.3:10)	0.056(0.048:0.064)	7.0(4.5:9.5)	4(1.1:6.9)	-3.0(-6.7:0.68)	0.1081
**Quintile**						
poorest	5.2(3.9:6.5)	0.025(0.016:0.033)	12.1(7.8:16)	3.1(1.3:0.049)	-9(-14:-4.4)	0.0002
2nd	0.064(0.048:0.08)	0.027(0.021:0.032)	0.095(0.070:0.12)	0.052(0.034:0.070)	-0.043(-.073: -.013)	0.0062
middle	0.074(0.050:0.098)	0.048(0.031:0.065)	0.10(.073:0.133)	0.044(0.013:0.076)	-0.059(-.100:-.017)	0.0069
4th	0 .087(0.063:0.110)	0.06(.041:0.078)	0.083(0.061:0.104)	0.039(0.014:0.064)	-0.044(-.075:-.012)	0.0076
richest	0.10(0.090:0.12)	0.064(0.049:0.079)	0.045(0.0035:0.086)	0.049(-.0068:0.105)	0.0045(-.062:0.071)	0.8937

Hospital utilisation for in-patient care

Overall, hospitalisation rates have increased in both AP and Maharashtra (Table 6) but more so in AP (5.6 per 1000 population vs 2.2), although the DID was not statistically significant. The sub group analysis presented a mixed picture. For both male and female headed households there was a greater increase in hospitalisation in AP, but this reached moderate statistical significance only for female headed households (DID=27.6, 95% CI =1.1:54.1, p-value = 0.0415). There is an increase in hospitalisations among scheduled tribes in Maharashtra and a reduction in AP (DID = -19.8, 95% CI = -37.3: -2.3, p-value = 0.0272) but the opposite picture was seen among 'other excluded' groups with an increase in AP and a reduction in MH (DID = 12.5, 95% CI= 1.2:23.9 p-value = 0.0309). In scheduled castes hospitalisations had increased in both states but more so in AP, while in the 'other' group there was a small increase in MH and a small reduction in AP. In the poorest quintile, the increase in hospitalisation was significantly greater in MH (DID= -14.4, 95%CI=-28:-0.31, p= 0.0451).

Table 6: Changes in hospitalisation (per 1000 population) in Maharashtra and AP between 2004 and 2012

Subgroups	Baseline Mean (95% CI)		Change 2004:2012 Mean (95% CI)		DID	
	Maharashtra	Andhra Pradesh	Maharashtra	Andhra Pradesh	Mean (95%CI)	p
**Hospitalizations per 1000 population**						

For peer review only - http://bmjopen.bmj.com/site/about/guidelines.xhtml

For peer review only

**All**	41.3(37.3:45.2)	31.5(27.8:35.3)	2.2 (-4.7:9.1)	5.6(-1.1:12.3)	3.4(-5.9 :12.7)	0.4636
					**DID (adjusted)**	
					0.7(-8.6:10.2)	0.8685
**Head of Household**						
Male	41.0(37.1:44.9)	31.7 (16.9:34.0)	1.9(-5.1:8.8)	4.4(-2.4:11.1)	2.5(-6.9:11.9)	0.5966
Female	51.6 (30.6:72.5)	25.5(27.7:35.7)	13.9(-7.53:35.4)	41.5 (24.6:58.4)	27.6(1.1:54.1)	0.0415
**Social Group**						
Scheduled Tribes	23.7(14.2 :33.1615)	35.5(21.9:49.1)	17.1(5.8:28.5)	-2.7(-16.95:11.5)	-19.8(-37.3: -2.3)	0.0272
Scheduled Castes	42.4(36.3:48.4)	29.5(22.6:36.5)	3.9(-7.9:15.7)	7.6(-2.6:17.7)	3.7(-11.4:18.7)	0.6268
Other Excluded	44.2(38.2:50.2)	29.9(25.5:34.3)	-1.9(-11.2 :7.3109)	10.6(3.4:17.8)	12.5(1.2:23.9)	0.0309
All other Groups	42.5(37.1:47.9)	34.9(29.0:40.9)	0.93(-7.3:9.1)	-1.1(-9.4: 7.4)	-2.0(-13.5 :9.4)	0.7235
**Location**						
Rural	36.6(32.2:41)	28.9(26.4:31.5)	9.5(3.5:15.4)	8.8(2.2:15.3)	-0.69(-9.3:7.9)	0.8725
Urban	48.2(43.7:53)	38.2(35.5:41.2)	-7.9(-14.5: -1.3)	-2.5(-12.1:7.1)	5.4(-5.8:16.6)	0.3358
**Quintile**						
poorest	31.4(25.9:36.9)	27.5(22.8:32.1)	20.7(9:32.8)	6.4(-1.7:14.4)	-14.4(-28:-0.31)	0.0451
2nd	36.5(28.3:44.7)	27.1(21.9:32.4)	8(0.5:15.4)	8.9(-.56:18.4)	0.9(-10 : 12.5)	0.8746
middle	47.8(33.6:62.1)	35.3(29.0:41.6)	-1.5(-17.9:14.8)	4.1(-.56:13.7)	5.6(-12.8:24.0)	0.5457
4th	46.9(39.9:53.9)	41.7(32.7:50.8)	-4.0(-12:3.4)	-5.1(-15.7:5.4)	-0.75(-13.3:11.9)	0.9056
richest	51.0(46:56.1)	36.5(25.8:47.2)	-13.1(-18.9: -7.1)	1.5(-19.5:22.6)	15.0(-5.8: 3.6)	0.1665

LIMITATIONS OF THE STUDY

Difference in differences estimations aimed at assessing the impacts of interventions, assume that both populations demonstrate similar characteristics prior to the start of the intervention, and that 'unobservables' follow a common trend; under such circumstances, any differences in changes observed over time between the 2 populations are attributable to the interventions[39, 40]. Despite AP and Maharashtra having broadly similar socio-economic profiles, as well as our DID analysis taking account of a number of covariates, there may have been other factors resulting in unobserved differential changes between the 2 populations and to which the results of the DID analysis may be at least partially attributable.

A second limitation could arise from the impact of other public health programmes implemented during the period 2004 to 2012. The most significant of these is the National Rural Health Mission launched in 2005, mainly to improve maternal and child health through the revitalization of rural primary care and child and maternal health services. A key assumption of our study is that the impacts of the NRHM in terms of healthcare expenditure for maternal and child health care would have been similar in both states, as this was a nationwide development. Despite the improvements in the public sector maternal and child health services sought by the NRHM, it is widely recognised that the public, including BPL families, continues to pay OOP for private health care. We have assumed that this behaviour is likely to be similar across the 2 states. Other health initiatives such as the Navsanjeevani Yojana and HMRI were unlikely to have had an impact on inpatient care or

For peer review only

expenditure. The 108 scheme had the potential, in AP, to influence hospitalisation rates, by helping more households to visit hospitals when seriously ill. But the effect is likely to be small as the majority of even serious illnesses do not result in a 108 call for transport. The implementation of the RSBY and the scheme to make private hospital beds available for the poor in MH may have diluted the DID, although both schemes are known to have been only partially implemented.

Lastly, the 2004 NSSO survey which served as our baseline, was carried out between January and June 2004. Our end-line 2012 survey was carried out over a period of 3 months from June to September. The morbidity and mortality patterns recorded in different time periods may vary, and could have influenced the data.

DISCUSSION

We found that average expenditure, large expenditures and large borrowings on inpatient care had increased in both MH and AP, but the increase was consistently smaller in AP across these 3 outcome measures, and may be suggestive of Aarogyasri having a somewhat larger effect than RSBY. Similar increases in institutional deliveries across the 2 states, and low levels of utilisation of RSBY and Jeevandayee schemes in MH may further strengthen this explanation.

The increase in average OOPE on inpatient care in AP and MH reflects a pattern observed nationwide[16, 41]. The Aarogyasri scheme may have contributed to the more favourable trajectory in AP both directly and indirectly, in that the scheme may have contributed to a reduction in the prices of interventions and an increase in competition among health care providers. The evaluation of the Yeshasvini scheme also found a significant reduction in the price of surgical interventions[10]. Our findings may suggest that the positive effects of Aarogyasri detected by other studies[15,16] at an early stage of the roll out of the scheme have been sustained. Automatic enrolment into the scheme, near universality of coverage and no requirement for enrollee contributions may have contributed to the significant DIDs in male headed households, scheduled castes, rural households and the poorest and middle asset quintiles. But these benefits were not demonstrated in some of the most vulnerable groups - female headed households and scheduled tribes. This is consistent with the findings of other studies[5,16] which reported that the slightly less vulnerable may benefit more from such schemes, with non-financial barriers undermining access to services for the most vulnerable socio-economic groups. The case summaries below illustrate the obstacles they face. A similar observation was reported by the evaluation of the Mexican Seguro Popular health scheme which showed that a third of the treatment-cluster households who were automatically affiliated were unaware of this fact[42].

The likely explanation of an Aarogyasri effect may be strengthened by the changes in terms of large borrowings which has also increased over time in both states but less so in AP across all groups of analysis except the richest asset quintile. A multi-country analysis of household catastrophic health expenditure highlighted that increasing the availability of health services is critical to improving health in poor countries, but it could also raise the proportion of households facing catastrophic expenditure unless financial risk protection policies are given a high priority[43]. Borrowing of comparatively small amounts is less impactful and may be a result of improved access to financial markets and also supports consumption smoothening. During recent decades, AP has in particular witnessed a significant rise in microfinance institutions and debt due to high levels of interest levied by the more recent entrants to this market[44, 45]. Nevertheless our results suggest that increases in large borrowings associated with inpatient health care were smaller in AP. Strongly significant DIDs in scheduled tribes, rural households and the poorest and second asset quintiles and moderately significant DIDs in female headed households, scheduled castes, and other excluded social groups may also point to Aarogyasri beginning to offer greater access to health care when families are faced with serious illness.

For peer review only - http://bmjopen.bmj.com/site/about/guidelines.xhtml

For peer review only

In terms of large OOPE, a significant DID was found only for the total households, but the direction of all DIDs except for the second asset quintile was the same as for average expenditure on inpatient care and large borrowings, i.e., in favour of AP. It is perfectly possible that the non-significant differences for the majority of sub groups are due at least in part to the RSBY, the availability of private sector hospital beds for the poor and the Jeevandayee schemes reducing large OOPE in MH, and that without these schemes the expenditure would have been greater. In AP, 70 percent of survey households reported that they were covered by the Aarogyasri scheme, but only 25 percent of these 'covered' households were aware that the benefit package was limited. It is also possible therefore that in AP some families seek hospital care assuming that the Aarogyasri scheme provides comprehensive cover, but are faced with large expenditures when their treatments fall outside the limits.

The mixed picture in relation to the increasing rate of hospitalisations in both states may suggest that the Aarogyasri and in particular, the RSBY scheme which covers common hospital procedures are addressing a large hitherto unmet need for inpatient care. The 108 scheme in AP may be an additional albeit smaller contributor. It may also be explained by a supplier-induced demand. An assessment of health provider behaviour and governance is an important strand of the evaluation of health financing schemes[46], but was outside the remit of our study. We would however strongly recommend an impact evaluation focusing on health care supply to complement our evidence. The greater increase in hospitalisation in AP, in female-headed households and 'other excluded groups', which are two of the most vulnerable population groups, is encouraging. But the reduction in hospitalisations among scheduled tribes and the poorest asset quintile in AP are of concern and suggest that if the poor are to secure the benefits appropriated by the near poor or more often by the rich[47], the provision of more comprehensive health schemes is essential, which combines the tertiary and secondary care focus of Aarogyasri and the secondary care benefits of RSBY with attention paid to minimise barriers such as the widespread influence of illiteracy and lack of awareness, which limit access to even schemes such as Aarogyasri that are apparently highly inclusive and non-discriminatory, as well as distance to facilities. Our case summaries illustrate this. Furthermore, health financing reforms such as the Jamkesmas[48] in Indonesia and the Seguro Popular in Mexico[42], both countries similar to India in terms of population and growing economies, include outpatient and inpatient care, and curative as well as preventive services, suggesting that a more comprehensive service is possible to implement and worthy of consideration.

In summary, health innovations in AP had a greater beneficial effect on hospital inpatient care-related expenditures than innovations in MH. The Aarogyasri scheme is likely to have contributed to these impacts in AP, at least in part. But in both states, OOPE increased over time, in keeping with the picture reported nationwide[49]. Likely explanations are that the poor spend the largest proportion of OOPE on drugs[50], an expenditure not adequately addressed by the health financing schemes including the RAS which provide only 'follow up' medicines for limited periods, after discharge from hospital. Yet, chronic disease such as diabetes may result in a one-off cardio-vascular intervention funded by the RAS, but also life-long medication required to be paid for out-of-pocket. Other possible explanations are medical inflation and rising costs of initial consultation and diagnostic investigation of symptoms, often in the rapidly growing private sector out-patient services, prior to hospitalisation under the cover of the RAS or the other health financing schemes. Hospitalisations also increased, and while it is generally assumed that in developing countries this is likely to address genuine need, its impact on health status is not known.

IMPLICATIONS FOR POLICY AND PRACTICE

Despite these uncertainties, Aarogyasri is perceived, with some justification across India as a successful scheme, and is being rapidly replicated across the states. Since July 2012, MH too has joined the list of states offering an Aarogyasri-like scheme, the Rajiv Gandhi Jeevandayee Arogya

For peer review only - http://bmjopen.bmj.com/site/about/guidelines.xhtml

Yojana[51]. This study has highlighted that such schemes may result in some positive outcomes. Although the study was not designed to elicit the specific features of the scheme to which any comparatively greater benefits may be attributable, and which deserve to be replicated in other states, evidence from systematic reviews[3] points to the need for schemes to be more 'comprehensively' designed to maximise their positive impact. Aarogyasri's design in terms of its aims to address both financial and non-financial barriers - being fully state funded, the systematic administrative implementation of Aarogyasri across the state so that it is now almost universal, automatic enrolment, allowing access to the scheme via a ration card which most poor families own, the wide spectrum of treatments offered, the large number of health care providers empanelled - is perhaps responsible as a 'comprehensive' package, for the greater impact.

But the study also suggests that improved access to health care and the reduction of the overall burden of OOPE especially in the most vulnerable sections of the population[52] are likely to require additional interventions that address gaps in the availability of care and provide patients appropriate pathways that support their journey from a strong and comprehensive primary care service where they may be informed of their entitlements and investigated for their initial symptoms to appropriate hospitals for the treatment of serious illness. Besides, inpatient care is only consumed by a small proportion of households in a year[53], and this is especially true of tertiary care, while many more will seek outpatient services and referral to inpatient care, should this be required. Others have strongly recommended the strengthening of the primary care base as an essential means to universal health coverage and this study confirms their view[41]. Key implications for AP are to explore how best the most advantageous features of Aarogyasri can be extended to include both secondary and primary care, while those for MH may be to build on and unify its menu of currently available schemes to create an evidence-based comprehensive health delivery system. These conclusions may be applicable to other states with similar health financing schemes.

The design of health financing systems as well as their evaluations is complex and challenging, as the mountain of available evidence suggests[41, 42, 52, 54]. Even the ground-breaking Seguro Popular health insurance programme of Mexico which used a cluster-randomised trial design with strong government support demonstrated some but not all anticipated outcomes, contrary to expectations[42], and a key recommendation was that continued assessment of the programme was needed. Furthermore, differences in data and methodology may result in even very well designed evaluations of the same programme producing contrasting findings[52]. Our evaluation is the first to use a survey methodology - the best possible in the hierarchy of evaluation methodologies, when a randomised control trial is not achievable - to evaluate the health financing reforms in 2 large states of India. Despite that, the study has limitations which we have acknowledged. For example, this study has examined health related expenditure and behaviours at only two points in time. To establish trends, it is suggested that more than two time points are needed. However, it needs to be recognised that schemes such as the Aarogyasri themselves may not have longevity in their original form and may change or be replaced in response to changing needs and policy imperatives. King et al acknowledged that their assessment of the Mexican Seguro Popular programme (at 10 months)[42] was undertaken at an early stage, but it was nevertheless recognised as having provided important evidence of impacts, albeit early ones. Therefore a realistic aim of an evaluation in this rapidly changing health delivery landscape in India would be to study change over time, even if that is limited initially to two time points, but to continue to evaluate the system repeatedly, and use the evidence to reshape health delivery to be responsive to future socio-economic and epidemiological trends. In addition, the evaluation was not designed to assess provider behaviours. Many other pertinent questions such as the impact of the schemes on the overall economy of health care cannot be answered by a single evaluation. But these are recognised problems which can only be addressed through continuous assessments, as other evaluations have shown. Our study has nevertheless produced sufficient insights to enable policy leaders to improve programme effectiveness and,

For peer review only - http://bmjopen.bmj.com/site/about/guidelines.xhtml

importantly, to undertake further assessment. Our household survey data provide a valuable baseline for future monitoring and analyses of trends in both states.

This study needs to be followed up with further and repeated evaluations as AP's and MH's schemes evolve; to assess the impacts of re-design and to help health policy leaders achieve their aspiration of universal access to good quality health care.

Three faces of the Aarogyasri Scheme

The beneficiary:

Lakshmamma, a 65 year old widow, is from a tribal background. She is an unskilled labourer, supporting a family of 5, with a monthly income of INR 4000 (USD 74). She was referred to a municipal hospital with chest pain and underwent heart surgery. The hospital which is a part of the Aarogyasri network provided free care and her total out of pocket expenses amounted to INR 850 (16 USD) for initial transport. In addition to the surgery, she received free food, money for transport home and follow up medicines. She is very satisfied with the service she received.

The excluded:

Ramulamma, a mother has a BPL card, but her daughter Lakshmi does not, as she was abandoned by her husband who is a government employee and entitled to free health care. The mother was unable to secure free care using her BPL card for her seriously ill daughter who paid out-of-pocket at a private facility where she was offered a hysterectomy for the relief of her gynaecological symptoms. Lakshmi's health care costs were met by the family selling a number of household assets and Lakshmi's daughter discontinuing her education to take up paid work. Lakshmi is severely depressed and does not speak to anyone.

The uninformed:

Krishnamma 43 years old, had severe stomach pains one night. Although the family had a BPL card, Ramulu her husband and Srinivas her son rushed her to a private hospital nearby, which was not part of the Aarogyasri network. They were unaware of how to access the Aarogyasri scheme hospitals which were further away from home, and the local primary health services being inadequate, Krishnamma had not had her initial symptoms investigated. The treatment was funded through a loan from a private moneylender. On discharge, she has not attended follow up, as the family cannot afford transport or medicines.

For peer review only - http://bmjopen.bmj.com/site/about/guidelines.xhtml

References

[1] Resolution WHA58.33. Sustainable health financing, universal coverage and social health insurance. In: *Fifty-eighth World Health Assembly, Geneva, 16–25 May 2005.* Geneva, World Health Organization, 2005 (http://apps.who.int/gb/ebwha/pdf_files/WHA58/WHA58_33-en.pdf, accessed 26th March 2013).

[2] World Health Organization. Health Systems Financing. Path to Universal Coverage. Geneva: World Health Organization. 2010. Available from: World Health Organization Library Information Services.

[3] Acharya A, Vellakkal S, Taylor F, Masset E, Satija A, Burke M, Ebrahim S. Impact of national health insurance for the poor and the informal sector in low and middle income countries : a systematic review. London: EPPICentre, Social Science Research Unit, Institute of Education, University of London. 2012. Available from: http://www.dfid.gov.uk/r4d/PDF/Outputs/SystematicReviews/Health-insurance-2012Acharya-report.pdf

[4] Giedion, U., B. Y. Diaz . A Review of the evidence. In: The Impact of Health Insurance in Low-and Middle-Income Countries. M. L. Escobar, C. Griffin and R. P. Shaw (ed). Washington DC, Brookings Inst Press 2011.

[5] Giedion, U, Alfonso, E , Diaz. *The impact of universal coverage schemes in the developing world : a review of the existing evidence*. Universal Health Coverage (UNICO) studies series ; no. 25. Washington D.C. : The World Bank. 2013

[6] National Commission on Macroeconomics and Health. Report of the National Commission on Macroeconomics and Health. New Delhi: Ministry of Health and Family Welfare of India. 2005. Available from: Ministry of Health and Family Welfare

[7] Eleventh Five Year Plan (2007–2012). Vol 2: Social Sector. Government of India Planning Commission. October, 2008. http://planningcommission.nic.in/plans/planrel/fiveyr/11th/11_v2/11th_vol2.pdf, (accessed Mar 26 2013).

[8] Faster, Sustainable and More Inclusive Growth. An Approach to the Twelfth Five Year Plan (2012-17). Government of India Planning Commission. October, 2011. http://planningcommission.nic.in/plans/planrel/12appdrft/appraoch_12plan.pdf, accessed 26th March 2013

[9] La Forgia G, Nagpal S. Government-Sponsored Health Insurance in India. *Are You Covered?* World Bank Washington DC 2012. doi:10.1596/978-0-8213-9618-6.

[10] Agarwal A. "Impact evaluation of India's 'Yeshasvini' community based health insurance programme. *Health Economics* 2010; 19: 5-35

[11] The Rajiv Aarogyasri scheme website at https://www.aarogyasri.org/ASRI/index.jsp, accessed 26th March 2013.

[12] RSBY scheme status. http://www.rsby.gov.in/statewise.aspx?state=35 (accessed Apr 30 2013)

[13] Mahal A. Learning and getting better: Rigorous evaluation of health policy in India. National Medical Journal of India. 2011. 325-27.

[14] Aarogyasri Health Care Trust Annual Report 2011-2012. https://www.aarogyasri.org/ASRI/EXT_IMAGES/documents/Annual_Report_201011.pdf (accessed Mar 26 2013)

[15] Rao M, Ramachandra S, Bandyopadhyay S, et al. Addressing healthcare needs of people living below the poverty line: a rapid assessment of the Andhra Pradesh Health Insurance Scheme. National Medical Journal of India. 2011. 24(6):335-41.

[16] Fan V, Karan A, Mahal A. "State Health Insurance and Out of-Pocket Health Expenditures in Andhra Pradesh, India." CGD Working Paper 298.Washington, D.C.: Center for Global Development. 2012 http://www.cgdev.org/content/publications/detail/1426275 (accessed Mar 15 2013)

[17] Palacios R, Das J, Sun C eds. India's health insurance scheme for the poor: Evidence from the early experience of Rashtriya Swasthya Bima Yojana. New Delhi: Center for Policy Research; 2011

[18] Rashtriya Swasthya Bima Yojana overview and scheme details. http://www.rsby.gov.in (accessed Apr 30 2013)

[19] Rathi P. http://www.priorities2012.com/documents/3d-4-patreek.pdf

[20] NRHM Mission Document.http://www.nird.org.in/brgf/doc/Rural%20HealthMission_Document.pdf (accessed Apr 30 2013)

[21] State HMIS data analysis, April'10-March'11 Andhra Pradesh, prepared by NHSRC.http://cfw.ap.nic.in/nrhm/pdf/AP_Analysis_Apr2010-Mar2011.pdf (accessed May 2 2013)

For peer review only - http://bmjopen.bmj.com/site/about/guidelines.xhtml

[22] NRHM achievements in Maharashtra. http://www.nrhm.maharashtra.gov.in/achievements.htm (accessed May 2 2013)
[23] Health information helpline.http://www.hmri.in/oursolutions-healthinformation.html (accessed Apr 30 2013)
[24] About the project/scheme. http://www.maha-arogya.gov.in/projectandschemes/Jeevandaiaarogya/default.htm (accessed Apr 30 2013)
[25] Jeevandayee scheme performance: Information received from Rajiv Gandhi Jeevandayee Arogya Yojana Society Govt of Maharashtra on May 17 2013
[26] Projects and schemes. http://maha-arogya.gov.in/projectandschemes/itdpnavsanjivani%5Cdefault.htm (accessed Apr 30 2013)
[27] Mallepeddi R , Pernefeldt H, Bergkvist S . Andhra Pradesh health sector reform, A narrative case study, Technical paper No.7 for The Rockefeller Foundation–Sponsored Initiative on the Role of the Private Sector in Health Systems in Developing Countries. 2009 ; pp 42-48
[28]EMRI. http://www.emri.in/states.html (accessed Apr 30 2013)
[29] Developing and evaluating complex interventions: new guidance. Medical Research Council. London 2008.
[30] Cesar G V, Jean-Pierre H, Jennifer B. Evidence-Based Public Health: Moving Beyond Randomized Trials. Am J Public Health. 2004 March; 94(3): 400–405.
[31] Presentation on Annual Plan 2012-13 and Five Year Plan 2012-17. http://planningcommission.nic.in/plans/stateplan/Presentations12_13/maharashtra1213.pdf (accessed on Mar 25 2013)
[32] National Sample Survey Organization. Morbidity, Healthcare and condition of the Aged. New Delhi: Ministry of Statistics and Programme Implementation. 2004.
[33] National Sample Survey Organization. Household Consumer Expenditure 66th round . New Delhi: Ministry of Statistics and Programme Implementation; 2009-10
[34] National Sample Survey Organization. Household Consumer Expenditure in India. New Delhi: Ministry of Statistics and Programme Implementation; 2005.
[35] Booysen F, Van der Berg S & Burger R. Using an Asset Index to Assess Trends in Poverty in Seven Sub-Saharan African Countries. World Development. 2008; 36(6): 1113–1130
36 Vyas S. & Kumarnayake L. Constructing socio-economic status indices: How to use principal components analysis. Health Policy and planning. 2006; 459-468
[37] O'Donnell O, Van Doorslaer E, Wagstaff A & Lindelow M, 2008. Analyzing Health Equity Using Household Survey Data: A Guide to Techniques and Their Implementation. Washington DC: World Bank Institute. Chapter 6, Measurement of Living Standards. http://siteresources.worldbank.org/INTPAH/Resources/Publications/459843-1195594469249/HealthEquityFINAL.pdf (accessed May 9 2013)
[38] Ministry of Finance. Economic Survey 2011-12. New Delhi: Government of India. 2012. Available from: http://indiabudget.nic.in/
[39] Poverty Reduction and Social Development Department. A User's Guide to Poverty and Social Impact Analysis. Washington: World Bank;2003
[40] Gertler P, Martinez S, Premand P, Rawlings L, Vermeersch C. Impact Evaluation in Practice. Washington: World Bank; 2011.
[41] Selvaraj S, Karan A. Why Publicly-Financed Health Insurance Schemes Are Ineffective in Providing Financial Risk Protection. Economic and Political Weekly 2012 March 17; XLVII(11):60-68
[42] King G, Gakidou E, Imai K, Lakin J, Moore RT, Nall C. Public policy for the poor? A randomised assessment of the Mexican universal health programme. Lancet. 2009 Apr 8; DOI:10.1016/S0140-6736(09)60239-7.
[43] Xu K, Evans DB, Kawabata K, Zeramdini R, Klavus J, Murray CJ. Household catastrophic health expenditure: a multicountry analysis. Lancet. 2003 Jul 12;362(9378):111-7.
[44] Naga Sridhar G. Microfinance institutions: Moving beyond AP crisis. The Business Line [Newspaper Online]. 2012 Dec 29. Available from: http://www.thehindubusinessline.com/companies/microfinance-institutions-moving-beyond-ap-crisis/article4253239.ece
[45] Naga Sridhar G. Life after microfinance in AP. The Business Line [Newspaper Online]. 2013 Jan 4. Available from: http://www.thehindubusinessline.com/opinion/life-after-microfinance-in-ap/article4273183.ece
[46] Prasad N P, Raghavendra P. Healthcare models in the era of medical neoliberalism. A study of Aarogyasri in Andhra Pradesh. Economic and Political Weekly 2012 Oct 27;XLVII(43):118-126

For peer review only - http://bmjopen.bmj.com/site/about/guidelines.xhtml

[47] Health Systems 20-20. An Evaluation of the Effects of the National Health Insurance Scheme in Ghana. Bethesda: Health Systems 20-20. 2009.

[48] http://www.jointlearningnetwork.org/programs/compare/benefits/142%2C16

[49] Ghosh S. Catastrophic Payments and Impoverishment due to Out-of-Pocket Health Spending. Economic and Political Weekly, 2011 Nov 19; XLVI(47): 63-70.

[50]Garg CC, Karan AK. Reducing out-of-pocket expenditures to reduce poverty: a disaggregated analysis at rural-urban and state level in India. Health Policy Plan. 2009; 24 (2):116-128.

[51] Rajiv Jeevandayi scheme details. http://www.jeevandayee.gov.in/RGJAY/FrontServlet?requestType=CommonRH&actionVal=RightFrame&page=undefined%3E%3E%3Cb%3ERGJAY%3C/b%3E&pageName=RGJAY&mainMenu=About&subMenu=RGJAY (accessed on Apr 30 2013)

[52] Tangcharoensathien V, Swasdiworn W, Jongudomsuk P et al . Universal Coverage Scheme in Thailand: Equity Outcomes and Future Agendas to Meet Challenges. Geneva: World Health Organization. 2010.

[53] Dilip TR. On Publicy-Financed Health Insurance Schemes. Is the analysis premature? Economic and Political Weekly. 2012 May 5; XLVII(1)8:79-80.

[54] Vallakal S, Ebrahim S. Publicly-Financed Health Insurance Schemes. Concerns about Impact Assessment. Economic and Political Weekly. 2013 Jan 5; XLVIII(1);24-27.

For peer review only

For peer review only - http://bmjopen.bmj.com/site/about/guidelines.xhtml

TITLE PAGE

TITLE:

~~Trends~~Changes in addressing inequalities in access to hospital care in Andhra Pradesh and Maharashtra states of India. *A cross sectional difference-in-differences study*

STUDY DESIGN: A cross-sectional difference in differences study with parallel control.

AUTHOR NAMES, ADDRESSES AND POSITIONS:

1.Mala Rao. Professor of International Health, Institute for Health and Human Development, University of East London, UK & Honorary Adviser, Administrative Staff College of India, Hyderabad

2. Anuradha Katyal, Consultant, ACCESS Health International, Hyderabad, India

3.Prabal V Singh, Director Research, ACCESS Health International, Hyderabad, India

4.Amit Samarth, Head, Strategy and Planning, SughaVazhvu Healthcare, Thanjavur, India

5.Sofi Bergkvist, Managing Director, ACCESS Health International, Hyderabad, India

6.Manjusha Kancharla, Research Associate, Indian School of Business, Hyderabad India

7.Adam Wagstaff, Research Manager, Human Development & Public Services team, Development Research Group (DECRG), The World Bank, Washington DC, USA

8.Gopalakrishnan Netuveli. Professor of Public Health, Institute for Health and Human Development, University of East London & ESRC International Centre for Life Course Studies in Society and Health, University College London, UK

9.Adrian Renton. Director, Institute for Health and Human Development, University of East London, UK

All authors had full access to all the data (including statistical reports and tables) and can take responsibility for the integrity of the data and the accuracy of the data analysis

Corresponding author and guarantor of the data and information: Mala Rao (best email address: m.rao@uel.ac.uk).

M Rao affirms that the manuscript is an honest, accurate and transparent account of the study being reported and no important aspects of the study have been omitted.

FUNDED BY : Wellcome Trust, IDRC, Rockefeller Foundation, DFID, World Bank.

ROLE OF THE FUNDING SOURCES: The external funding sources had no role in study design, data collection, analysis, interpretation or reporting, or in submission decision.

ROLE OF THE STUDY SPONSOR: The study was carried out in response to the formal invitation of the Government of Andhra Pradesh which supported our applications for funding (by providing formal

For peer review only - http://bmjopen.bmj.com/site/about/guidelines.xhtml

letters of support). The Government did not provide any funding, and had no role in study design, data collection, analysis, interpretation or reporting, or in submission decision.

STATEMENT OF INDEPENDENCE OF RESEARCHERS: The authors can confirm that they worked independently from the Funders and Government of Andhra Pradesh who had no role in any aspect of the research

CONFLICT OF INTEREST

All authors have completed the ICMJE forms for disclosure of potential conflicts of interest (available on request from the corresponding author) and declare that 1) MR, AK, PS, AS, and SB have support from the Rockefeller Foundation, Wellcome Trust, International Development Research Centre, Canada and Department for International Development, UK, 2) the authors have no relationships with the funders that might have an interest in the submitted work in the previous 3 years, 3) the authors' spouses, partners, or children have no financial relationships that may be relevant to the submitted work, and 4) the authors have no non-financial interests that may be relevant to the submitted work.

ETHICAL APPROVAL: given by the research ethics committee of the Administrative Staff College of India, Hyderabad, which hosted the study. Household survey questionnaires include signed consent by the head of the household or another adult representative of the household.

DATA SHARING STATEMENT: The 2004 National Sample Survey Organisation of India household survey questionnaire and data are available to the public. The 2012 household survey questionnaire, and the full anonymised household survey dataset will be available with open access as soon as the data analyses and submissions for publication are completed.

COPYRIGHT STATEMENT: Mala Rao, the Corresponding Author has the right to grant on behalf of all authors and does grant on behalf of all authors, an exclusive licence on a worldwide basis to the BMJ Publishing Group Ltd to permit this article (if accepted) to be published in BMJ editions and any other BMJPGL products and sublicences such use and exploit all subsidiary rights, as set out in your licence.

STROBE CHECKLIST FOR CROSS SECTIONAL STUDIES: We can confirm that all 22 items in the STROBE checklist are included in the paper.

CONTRIBUTORS' STATEMENT

MR conceived and designed the study, applied for funding, and was responsible for the supervision and management of all aspects of the study as well as the dissemination of its results. She is also the guarantor of the study. SB shared responsibility for the conception of the study, applications for funding, study design and data collation and analysis, contributed to the questionnaire design and commented on drafts of the report. PS contributed to the conception of the study and study design, led the questionnaire design and survey implementation, including training of survey staff, monitoring survey progress and data collation and verification, commented on drafts of the report and helped prepare the references. AK undertook the data collation, verification and analysis, assisted with the survey and questionnaire design and survey implementation and prepared the

For peer review only - http://bmjopen.bmj.com/site/about/guidelines.xhtml

For peer review only

tables for the report. AS led the literature review, assisted with the study and questionnaire design, survey implementation and preparation and analysis of baseline data, and commented on drafts of the report. MK helped with the data analysis. AW devised the methodology for the estimation of the programme impacts, advised during the data-collection and data-preparation stages, wrote and implemented the computer code for the model estimation, helped to oversee the production of the results, and contributed text to the report. GN provided technical advice on accounting for the complex survey structure in the analysis, developed a STATA equation, helped to compute an asset index, advised on the output tables, verified the analysis and commented on drafts of the report. AR helped develop a conceptual framework for the evaluation, advised on funding proposals, the study design, analytical methodology and presentation of results and contributed text to the report. MR wrote the first draft of the paper and its redrafts in accordance with the comments of all other authors and reviewers.

For peer review only

For peer review only - http://bmjopen.bmj.com/site/about/guidelines.xhtml

ABSTRACT. WORD COUNT: 300

OBJECTIVES

To compare the effects of the Rajiv Aarogyasri Health Insurance Scheme of Andhra Pradesh (AP) with health financing innovations including the RSBY in Maharashtra (MH) over time on access to and out of pocket expenditure (OOPE) on hospital inpatient care.

STUDY DESIGN

A cross-sectional difference in differences (DID) study with parallel control.

SETTING

National Sample Survey Organisation of India (NSSO) urban and rural 'first stratum units', 863 in AP and 1008 in MH.

METHODS

We used two cross-sectional surveys: as a baseline, the data from the NSSO 2004 survey collected before the Aarogyasri and RSBY schemes were launched and as post-intervention, a survey using the same methodology conducted in 2012.

PARTICIPANTS

8623 households in AP and 10073 in MH

MAIN OUTCOME MEASURES

Average OOPE, large OOPE and large borrowing per household per year for inpatient care, hospitalisation rate per 1000 population per year.

RESULTS

Average expenditure, large expenditures and large borrowings on inpatient care had increased in both MH and AP, but the increase was smaller in AP across these 3 measures. DIDs for average expenditure and large borrowings were significant and in favour of AP for the rural and the poorest households. Hospitalisation rates also increased in both states but more so in AP, although the DID was not significant and the sub group analysis presented a mixed picture.

CONCLUSIONS

Health innovations in AP had a greater beneficial effect on inpatient care-related expenditures than innovations in MH. The Aarogyasri scheme is likely to have contributed to these impacts in AP at least in part. However, out of pocket expenditure increased in both states over time. Schemes such as the Aarogyasri and RSBY may result in some positive outcomes but, additional interventions may be required, to improve access to care for the most vulnerable sections of the population.

For peer review only

ARTICLE SUMMARY

STRENGTHS AND LIMITATIONS OF THE STUDY

- This study uses a quasi-experimental design2 cross-sectional surveys to compare changes between the Indian states of Andhra Pradesh and Maharashtra, in hospital inpatient care related expenditures and behaviours before and after the rollout of the Aarogyasri and RSBY schemes
- The study based on a survey of 18696 households has shown that health innovations in Andhra Pradesh had a greater beneficial effect on hospital inpatient care-related expenditures and access than innovations in Maharashtra. The Aarogyasri scheme is likely to have contributed to these impacts in Andhra Pradesh.
- The study also highlights the implications of the findings for policy and practice and additional interventions necessary to address gaps in the availability of and access to care.
- The study is only able to compare the effects of health innovations over time across the 2 states but does not allow the drawing of inferences on the impacts of individual initiatives.
- The study uses the difference in differences methodology and its findings may have been affected by unobservable differential changes between the 2 states.

For peer review only

For peer review only - http://bmjopen.bmj.com/site/about/guidelines.xhtml

ACKNOWLEDGEMENTS:

The study was funded by the International Development Research Centre, Canada, the Wellcome Trust, the UK Department for International Development, and Rockefeller Foundation. The World Bank supported Adam Wagstaff's contribution to the study. We gratefully acknowledge the financial support of these organisations, as well as the enthusiastic encouragement we have received from them throughout the study. Our thanks also go to Bhimasankaram Pochiraju, Sundaresh Peri, C Ravi, and Rahul Ahluwalia for their advice and guidance at various stages of the study design and data analysis, and to colleagues at the University of East London and Administrative Staff College of India Hyderabad for their administrative support. The authors also thank P Suryanarayana, CM Reddy, D Chakrapani and K Pandu Ranga Reddy and AY Jadhav and his colleagues for their contribution to the training of the survey teams, verification of the survey and data collection and acknowledge the IMRB International Social Research Institute team's support in carrying out the survey.

We are grateful to Somil Nagpal and Joseph Kutzin for commenting on the report and helping us to improve it, to Sujatha Rao for her encouragement and valuable suggestions and ideas throughout the course of the study and to Jay Bagaria for her help during the early stage of the development of the proposal. Finally we thank PV Ramesh without whose constant support, guidance and encouragement, this study would not have been possible.

For peer review only

INTRODUCTION

In 2005, member states of the World Health Organization (WHO) committed to develop their health financing systems to deliver universal coverage (UC); that is that all people have access to health services and do not suffer financial hardship paying for them[1] . WHO's 2010 World Health Report[2] recommended *inter alia* that countries reduce reliance on direct payments, and improve equity of access, including through the introduction of prepayment schemes. However, one recent systematic review of the impact of national health insurance schemes in low and middle income countries[3] found only weak evidence of increased use of healthcare and reduced out-of-pocket expenses, with the poorer benefitting less, whilst others[4,5] concluded that health insurance improved health care access and use, as well as financial protection in most cases but had no conclusive impact on health status. Both highlighted the need for more rigorous assessments of such schemes.

India has one of the highest levels of out-of-pocket health expenditure (more than 80% of private health expenditure)[6]. The aim of our study was to explore how recently introduced health financing initiatives have affected access to and out-of-pocket expenditure (OOPE) on inpatient hospital care in the Indian states of Andhra Pradesh (AP) and Maharashtra (MH).

BACKGROUND

In its *Eleventh Five Year Plan (2007-12)*[7], Government of India sought to increase Public Expenditure on Health and to strengthen investment in rural health infrastructure through the National Rural Health Mission. Its *Twelfth Five Year Plan (2012-17)*[8], reflected the recommendation of the Planning Commission's High Level Expert Group for general taxation to be the principal source of health care financing. It proposed the development of government-funded health insurance schemes, building on the evidence from experimental schemes being introduced across many States. In India, health is primarily a state rather than national responsibility[6]. Whilst it is recognised that political will and good governance are essential, both the political mobilization of funds for health schemes and the effectiveness and efficiency of funded schemes will be enhanced by robust evidence which documents as to whether schemes achieve objectives, what works well and the main challenges they face.

In India, evaluation is not routine, even of large costly public health care programmes. Nevertheless, some assessments have been carried out, for example of the Yeshasvini Co-operative Farmers Health Care Scheme of Karnataka, the longest running state-supported health insurance scheme for the informal sector in India[9], with promising results in terms of increased utilization of and reduced borrowing for health care services[10]. However, these early models covered small populations and offered limited benefits, so that the policy implications of conclusions drawn from even the best evaluations were unclear.

This scenario has changed during the past 5 years, with the launch of 2 schemes, the Rajiv Aarogyasri[11] Community Health Insurance Scheme (Aarogyasri) of Andhra Pradesh (AP) and Rashtriya Swasthya Bima Yojana (RSBY) currently offered in 30 states and union territories of India[12] including AP's neighbouring state of Maharashtra (MH). Their scale in terms of population coverage and range of treatments offered, significantly enhances their potential to inform India's road map towards universal health coverage. Both schemes belong to the new generation of publicly funded government-sponsored health insurance schemes, principally aimed at providing financial protection to the poor against catastrophic health shocks, which, for these schemes, the Government has defined as inpatient hospital care[9]. Both schemes have been subject to some assessment in their early phases, but a recent editorial[13] highlighted the necessity of further and repeated evaluations to enhance the credibility and accountability of existing schemes and to identify those which deserve scale-up. The Aarogyasri and RSBY schemes and the other recent health sector innovations in AP and

For peer review only - http://bmjopen.bmj.com/site/about/guidelines.xhtml

Maharashtra which form the major backdrop and complex health care architecture against which these have been launched, and which may have contributed to the changes in outcomes we have explored in our study, are described below.

The Rajiv Aarogyasri Health Insurance Scheme of AP

In 2007 AP launched a pioneering new state-wide fully state-funded health insurance scheme, the Rajiv Aarogyasri Community Health Insurance Scheme (Aarogyasri)[11] to provide treatment for serious and life-threatening illnesses. The specific objectives include: to improve access of poor families to quality medical care (meaning low-frequency, high cost specialist care) and treatment of identified diseases requiring hospitalisation through an identified network of health care providers, to provide financial cover for catastrophic illnesses which have the potential to wipe out life time savings of poor families and to provide 'universal coverage to the urban and the rural poor in the state[14] albeit for the conditions covered in the benefits package. All families with a 'below poverty line' (BPL) ration card, i.e. those on an annual income below USD 1384 (INR 75,000) in urban areas and USD 1107 (INR 60,000) in rural areas, and including individuals with pre-existing medical conditions are *automatically* enrolled and the scheme was estimated to cover approximately 20.4 million poor and lower middle class families, comprising about 85 percent of the state's population in 2009[9]. Enrollees make no contribution, the annual benefit is a maximum of USD 4,500 (INR 200,000) per family per year and there is no limit on the size of the family[14]. A total of 942 medical and surgical procedures across 31 clinical specialties[14] are provided and the benefits include all inpatient costs - associated investigations, food, transport and medicines for 10 days following discharge. One year follow-up packages including consultation, medicines, and diagnostics are also available for 125 procedures requiring longer periods of follow up[9] . Aarogyasri has unique features including *Aarogyamithras* (health system navigators), outreach *health camps* delivered by participating hospitals to educate, screen and case-find and a state-of-the-art information technology-based management system. At the time of this study, 353 public and private sector hospitals were 'empanelled' to provide services to Aarogyasri beneficiaries.

In 2009, a descriptive study of *Aarogyasri*, based on an analysis of claims data and a survey of beneficiaries[15], concluded that while the scheme was beginning to reach its intended beneficiaries uptake was lower among scheduled castes and tribes. This was confirmed by Fan and colleagues[16], who used variations in programme roll-out over time and districts to evaluate the scheme using National Sample Survey data collected before and after its launch. They reported reduced out-of-pocket expenditure in this initial phase but no major impact on catastrophic healthcare expenditure. Inspired by Aarogyasri and mindful of the political benefits of introducing popular health reforms, other states have launched health financing innovations similar to this model.

RSBY in Maharashtra

RSBY was launched across a number of states by the Ministry of Labour, Government of India (GOI) in 2008[17] and provides access to free inpatient hospital care up to USD 550 (INR 30,000) per family per year[18]. Households which meet the criteria based on the much more limiting definition of poverty and numbers of poor families provided for each State by the GOI Planning Commission are eligible to enrol, and pay a contribution of USD 0.55 (INR 30) at registration and at each annual renewal[9]. Up to 5 family members, including those with pre-existing conditions can be covered, and personal information including biometric data are collected prior to the issue of a smart card with encoded details of the family. 700 procedures covering 18 broad categories of interventions which would generally be included under the umbrella of 'secondary' care, are provided and the benefit packages include the intervention, public transport costs limited to 1.8 USD (INR 100) per visit and 18.2 USD (INR 1000) per year and post-hospitalization drugs for 5 days. Networked hospitals are required to provide free outpatient consultations (which have only recently been introduced. Personal communication, Kurian OC) but other costs such as ambulatory diagnostics and medicines

For peer review only - http://bmjopen.bmj.com/site/about/guidelines.xhtml

have to be borne by the beneficiaries, except if investigations lead to inpatient admissions within a day[9]. A pilot of the RSBY scheme was launched in 1 district of AP, but only after the start of our household survey.

In Maharashtra, enrolment began in Aug 2009, and by mid 2013, approximately 2 million of the eligible 4 million families were enrolled in the scheme[12] which is being implemented in 31 of 35 districts in the state. Enrolment had extended to 26 districts prior to June 2012, when our household survey began. Notably, only 15 out of 1215 hospitals contracted for RSBY funded services are from the public sector[12]. Early assessments of the scheme's impact nationally, suggest that although the rate of hospitalisations has increased, awareness of the scheme was poor and remains a barrier to uptake. Notable variations in enrolment and scheme awareness were also observed by a descriptive study of RSBY conducted in the Amravati district of Maharashtra which has a large tribal population[19]. In Maharashtra[17], utilisation rates have been reported to be lower than in other states and the male:female enrolment ratio is 6.5:3.5.

Other major health sector initiatives in AP and Maharashtra

Both states have a complex health care landscape with numerous programmes in place. There are several initiatives launched during the past decade, some of which are common to both states and driven by national strategies, and others owe their existence to state-level enterprise, innovation and political support. The most notable programmes with the potential to impact on in patient care are described below.

The National Rural Health Mission (NRHM) was launched in 2005 nationwide, with a key aim of reducing maternal and infant mortality[20]. Government reports suggest that its notable achievements include an increase in institutional deliveries; in AP from 1.25 million in 2005-06 to 1.46 million by 2011-12[21] and in MH from 1.1 million to 1.63 million[22], achieving an institutional delivery rate of approximately 92 percent in both states. Also common to both states is the '104 health information help line' launched in AP in 2008 and MH in 2011[23], to provide medical advice and information based on validated algorithms and disease summaries, direct callers to appropriate health facilities or to receive a complaint against a public sector health facility. In AP the help line and call centre were subsumed within the Aarogyasri infrastructure by 2011.

In MH, the RSBY was preceded by the Jeevandayee scheme launched in 1997 with the objective of reducing catastrophic OOPE on inpatient care in the BPL population[24]. Potential beneficiaries were required to apply for funding after a diagnosis was confirmed and the scheme covered serious illness such as cardiac and renal disease and cancer. However, the scheme uptake has been low, and while it has continued to run in parallel to the RSBY, only 66,853 procedures (4456 procedures per year in a state with 112.37 million people) have been approved during the scheme's lifetime[25]. Since 2006, MH has also had a scheme in place which mandated 20% of the beds in private hospitals to be made available for free or at subsidized rates to poor patients (personal communication, Kurian OC). It has been estimated that around 10,000 private beds are available for the poor across MH, equivalent to approximately 20% of the total bed capacity of the public sector. Although the implementation of the scheme is reported to be erratic (Kurian OC), it may have had some positive impact on access to hospital inpatient care for serious illness.

Launched in 1995-96, the Navasanjeevani Yojana scheme is exclusive to the 15 tribal districts of MH and was to improve maternal and infant mortality in these vulnerable populations[26]. It has focused on strengthening primary health and nutrition services and access to safe drinking water.

A service available in AP but not in MH is the '108' scheme, launched in 2005 to provide a state-of-the-art medical emergency response service[27]. At the time of our study, 802 ambulances catered to approximately 3,500 emergencies per day[28].

For peer review only - http://bmjopen.bmj.com/site/about/guidelines.xhtml

OBJECTIVES OF THE STUDY

Our objective was to compare the effects of health innovations over time on access to and OOPE on inpatient care in AP and MH and to assess whether the AP initiatives had larger or smaller beneficial effects than those found in MH. These differential effects are likely to be substantially due to the Aarogyasri scheme in AP and the RSBY in MH. In this paper, we report findings from a study which compared these ~~trends~~changes. The findings do not allow us to draw inferences on the impacts of individual initiatives, but nevertheless contribute new knowledge on the impact and role of the innovations, provide lessons for other programmes, and strengthen the evidence base for policy on UC in India.

METHODS

Overview

None of the aforementioned initiatives – including the Aarogyasri and RSBY schemes in which we are especially interested – was piloted in a systematic way, let alone via a carefully designed randomized control trial. Following MRC Guidance[29,30] and best practice we therefore opted for a ~~quasi experimental~~cross-sectional survey design in which we seek to minimise selection bias, to control for confounding variables and to reduce the effects of chance. Specifically, we compare changes in hospital inpatient care related expenditures and behaviours (HREB) in AP and MH before and after the rollout of the Aarogyasri scheme in AP and the RSBY scheme in MH. The difference in changes between AP and MH is not an estimate of a specific initiative. Rather it tells us whether, on balance, the AP initiatives have had larger (or smaller) beneficial effects than the MH initiatives, and if so how much more (or less) beneficial they have been. Since the NRHM was common to both states and the MH-specific initiatives were quite small in scale or unlikely to affect HREBs, any difference in change between the two states is quite likely to be mainly due to differential effects of the Aarogyasri and RSBY programmes.

AP and MH have a broadly similar development profile as shown by the data below. AP's other socio-economically similar neighbouring states of Karnataka and Tamil Nadu had already introduced Aarogyasri-like schemes, and Odisha and Chattisgarh the only other neighbours, had comparatively higher levels of socio-economic deprivation. HREBs were measured in both AP and MH by two waves of household survey before (2004) and after (2012) the introduction of Aarogyasri and RSBY. The study protocol and questionnaire for the 2012 survey were reviewed and agreed by the Research Ethics Committee of the Administrative Staff College of India, Hyderabad.

Maharashtra	Indicator	Andhra Pradesh
112.37	Population (2011 census, in millions)	84.66
10.20	% Schedule Caste (2001 Census)*	16.60
8.90	% Schedule tribe (2001 census)*	6.20
101,314	Per capita income 2011-12 (in INR)**	71,540
35	Number of districts	23
5,314	Households covered in NSSO 60th round (2004-05)	5,059
10,073	Households covered in the study 2012	8,623

For peer review only - http://bmjopen.bmj.com/site/about/guidelines.xhtml

*Note that 2011 census data for social groups are not yet published

**Source: Presentation on Annual Plan 2012-13 and Five Year Plan 2012-17[31]

Survey design

Baseline Survey : 2004

We used the original data from the National Sample Survey Organization (NSSO) 60th decennial round household survey undertaken in 2004[32] to estimate baseline HREB estimates for AP and MH (Table 1). This was the most recent round measuring morbidity profiles, use of health care services including hospitalised and non-hospitalised treatments and expenditures incurred. The household survey used a multi-stage stratified sampling methodology to identify a representative random population sample and an interviewer completed questionnaire to obtain measures of HREB along with socio-demographic, household expenditure and other information.

Follow up survey: 2012

We used the same household survey design and methods to collect post-intervention data in AP and MH as those used by NSSO. Briefly, the household survey used a multi-stage stratified sampling methodology with the 'First Stage Units' (FSUs) identical to those used by NSSO in their 66th round (2008-09)[33], the latest round for which FSUs had been mapped. However, the FSUs were not the same as those in NSSO 2004, our baseline survey, rapid urbanisation having changed substantially, the urban-rural landscape of both states and thus the geographical basis for sampling units.

Table 1: Urban and rural populations and households surveyed in 2004 and 2012 in Andhra Pradesh and Maharashtra

	Andhra Pradesh		Maharashtra	
	NSSO 60th round 2004-05	Our survey 2012	NSSO 60th round 2004-05	Our survey 2012
Population	76,210,007*	84,665,533**	96,878,627*	112,372,972**
Urban population	20,808,940*	28,353,745**	41,100,980*	50,827,531**
Rural population	55,401,067*	56,311,788 **	55,777,647*	61,545,441**
Total households (urban)	4,397,138*	6,778,225**	8,403,224*	10,813,928**
Total households (rural)	12,607,167*	14,246,309**	11,173,512*	13,016,652**
Total households	17,004,305*	21,024,534**	19,576,736*	23,830,580**
FSUs (urban)	183	372	267	504
FSU (rural)	325	491	265	504
Total households surveyed (urban)	1824	3715	2664	5038
Total households covered (rural)	3235	4908	2650	5035

*2001 census ** 2011 census

FSU - First Stratum Unit

The NSSO 66th round had 492 rural FSUs in AP, but 1 FSU was found to be uninhabited.

For peer review only - http://bmjopen.bmj.com/site/about/guidelines.xhtml

The interviewer completed household survey questionnaire was pre-tested and then piloted in both states prior to the survey. All respondents provided written informed consent for participation. Questions addressed the following: household composition and socio-demographic characteristics of members, household expenditure, and health expenditure (outpatient and inpatient) and means of its financing, healthcare seeking behaviour, factors affecting access to healthcare and awareness and perceptions of the quality of the *Aarogyasri* scheme (AP only). The survey questions in 2012 were identical to those from the NSSO 2004[34]. Additional questions specific to the Aarogyasri and other relevant schemes were also added.

A survey of 18696 households across 2 states and 1871 locations within the states is a challenging undertaking. The survey design had several features intended to assure the quality of data collected. Few academic institutions have internal capacity to carry out such large surveys, and consequently, the Social and Research Institute of IMRB International, a leading market research agency was selected to carry out the survey. The Institute has field survey teams based in every Indian state, conversant in local languages and dialects and trained to carry out surveys in the socio-economic development sector. Its clients include Government of India (for whom the national Family Health Survey data are collected), World Bank and other UN organisations. A group of NSSO consultants in AP and the Indian Socioeconomic Research Unit, Pune were recruited to support the training of the field survey teams and data verification.

We planned three levels of verification of the study data; the first to be undertaken by the survey agency, the second to be carried out by the study team and the third, by the agencies mentioned above. Survey teams for each district were accountable to a field supervisor who was responsible for checking both the household listing and data entry on a daily basis. The study team also accompanied the field staff to survey sites on a regular basis. Data collected from 250 households in each state (approximately 2.5% of the surveyed households) and 186 of the FSU listings (approximately 10%) were independently verified by the agencies in both the villages and urban blocks in order to ensure that the sampling method and administration of the questionnaire survey were being correctly applied. The data entry was carried out by the Institute using a double entry method and any questionnaires reported incorrect were sent back to the field for re-survey. The research team carried out a final validation and review of the data.

Outcome measures

Average inpatient (IP) expenditure per household per year. Average out-of-pocket expenditure for inpatient care during 1 year prior to the survey was estimated from questionnaire responses for AP and MH from both baseline and follow-up data. Reimbursements for inpatient expenditure were deducted from the total where households had received them.

Large out-of-pocket inpatient expenditure. Because of the limited data on household consumption in the 2004 NSSO health survey we did not estimate 'catastrophic health expenditure'. Instead, we constructed a measure of 'large' out-of-pocket expenditure. The Aarogyasri Health Care Trust data on expenditure incurred by the Government of AP per case in 2012 were examined[14] and the mean was estimated as USD 419 (INR 23,000). A household was deemed to have incurred 'large' expenditure if OOPE for inpatient care was equal to or greater than this threshold.

For peer review only - http://bmjopen.bmj.com/site/about/guidelines.xhtml

Large borrowing. We estimated the total amount borrowed by a household to meet the expenditure of all the inpatient episodes of that family during the previous year. A household was considered to have incurred 'large borrowing' if the borrowing was equal to or exceeded the BPL threshold set by Government of AP: INR 70000 for urban families and INR 65000 for rural households. These prices have been deflated to 2004 levels.

Hospitalisation rate. This was estimated as the number of individuals hospitalised during the previous year, per 1000 population.

Variations in outcomes were examined between male and female-headed households, and rural and urban populations, as well as across social groups and economic groups represented by asset quintiles.

Because of the limited data on household consumption in the 2004 NSSO health survey which made the estimation of wealth difficult, we have opted instead to measure household living standards using an asset or wealth index based on information on ownership of household durables, dwelling type, etc., using principal component analysis to estimate weights[35, 36,37] for each indicator. The indicators used were limited to those collected in the 2004 NSSO: type of structure of the dwelling unit, type of toilet, type of fuel used for cooking and source of drinking. Data from the 2004 and 2012 surveys were pooled so that the index captures changes in living standards between the two years; there are therefore more households in the top quintile in 2012 than in 2004. The statistical software Stata 11 was used to generate the index.

Deflation of follow-up expenditure estimates.
The 2012 expenditure data including the threshold for large expenditures were deflated using the consumer price index of the Government of India[38] to reflect 2004 prices.

Analysis
Our repeat cross-sectional surveys do not allow estimation of within-individual household changes in outcomes over time. Our analysis therefore focused on estimating outcomes averaged across states, and in comparing changes in these over time between AP and MH. If we assume that outcome determinants other than Aarogyasri and RSBY remained stable in the two states over time or followed a parallel ~~trend~~change, then a difference-in-differences (DID) analysis will uncover the net effect of Aarogyasri over and above RSBY.

The difference in differences of outcome (Y_{DD}) is

$$(Y_{2012}^{AP} - Y_{2004}^{AP}) - (Y_{2012}^{MH} - Y_{2004}^{MH})$$

where the subscripts and superscripts for Y refer to the respective states and the years when the surveys were done. Confidence intervals were calculated from the standard error Y_{DD} of and the p-value for the null-hypothesis ($Y_{DD} = 0$) was tested using the Wald test as $t = \frac{Y_{DD}}{SE_{Y_{DD}}}$ with one degree of freedom. Y_{DD} was estimated using ordinary least square regression:

$$y_{it} = \beta_0 + \beta_1 state_i + \beta_2 survey_t + \beta_3 (state * survey)_{it} + \sum_{k=1}^{m} \beta_{3+k} covariate_k + \varepsilon$$

The basic DID results are obtained using the above regression with covariates excluded. The adjusted DID results are obtained using the above regression with m=9 covariates, namely the gender of head of household, a dummy variable capturing whether the household lives in a rural or urban location, three dummy variables capturing the household's social group (the lowest is the excluded category),

For peer review only - http://bmjopen.bmj.com/site/about/guidelines.xhtml

and four asset quintile dummies (the bottom is the excluded category). In the regression y_{it} is the outcome, *state* is a dummy variable with 0 for MH and 1 for AP, and *survey* is a dummy variable with 0 for 2004 survey and 1 for 2012 survey. The coefficient for the interaction term, β_3, gives the differences in differences estimate, Y_{DD}. Robust standard errors of Y_{DD} were calculated to account for clustering of households within FSUs using Stata survey commands. A positive value for Y_{DD} suggested that the change in the outcome in AP was more than the change in MH and the negative value would suggest the reverse.

An advantage of regression based DID estimate is this ability to use co-variates which can account for differential trajectories in the two states. In addition to this we did sub-group analysis stratifying for different co-variates. This is particularly relevant in the case of scheduled tribes whose proportion increased in MH in the follow up survey.

Sub groups were not mutually adjusted for the analysis due to sample size restrictions in relation to some of them.

Role of the funding sources
The external funding sources had no role in study design, data collection, analysis, interpretation or reporting, or in submission decision.

RESULTS

A total of 5314 and 5059 households from Maharashtra and AP were surveyed by the NSSO in 2004 (Table 2). Our survey in 2012 included 10073 (MH) and 8623 (AP) households.

Table 2. Socio-demographic characteristics of baseline and follow-up samples

Subgroups	Number (%) of Households 2004		Number (%) of Households 2012	
	Maharashtra	Andhra Pradesh	Maharashtra	Andhra Pradesh
**All**	5314	5059	10073	8623
**Head of Household**				
Male	4785 (90.0)	4433(87.6)	8543(84.8)	7418(86.0)
Female	529 (10.0)	626 (12.4)	1530 (15.2)	1205 (14.0)
**Social Group**				
Scheduled Tribes	413 (7.8)	296 (5.9)	1364 (13.5)	883 (10.2)
Scheduled Castes	809 (15.2)	974 (19.3)	2235 (22.2)	1797 (20.8)
Other Excluded	1644 (30.9)	2317 (45.8)	1899 (18.9)	3419 (39.7)

For peer review only - http://bmjopen.bmj.com/site/about/guidelines.xhtml

All other Groups	2448 (46.1)	1472 (29.1)	4571 (45.4)	2524(29.3)
**Location**				
Rural	2,650 (49.9)	3235 (63.9)	5035(50.0)	4908 (57.0)
Urban	2664 (50.1)	1824 (36.1)	5038 (50.0)	3715(43.0)
**Asset Quintile**				
Lowest	1,260 (23.7)	1,594(31.5)	996(9.9)	826(9.6)
Second	1,016 (19.1)	1,237 (24.5)	1,841(18.2)	1,286(14.9)
Third	772 (14.5)	753(14.9)	2,228(22.1)	2,121(24.60)
Fourth	857 (16.1)	744(14.7)	2,373 (23.6)	3,072(35.6)
Fifth	1,408(26.5)	730(14.4)	2,633(26.1)	1,318(15.3)

For peer review only

Changes in average in-patient expenditure

Table 3 (top panel) shows average baseline levels of inpatient expenditure. The table also shows the real terms change (deflated to 2004 prices) in these outcomes at follow up and the difference in differences (DID) estimate comparing AP with MH. DIDs for overall results are shown unadjusted, as well as adjusted for the effects of the covariates. Breakdowns by sex of head of household, social group, urban/rural location and asset quintiles are also shown.

Overall, average inpatient expenditure increased in real terms in both the states between 2004 and 2012, but the increase was significantly greater in MH (unadjusted DID= -498.2 INR, 95% CI= -792.9: -203.5, p=0.0009). The ~~trend~~direction in terms of a greater increase in MH was evident across all sub groups of analysis except the richest asset quintile. However, the DIDs reached significance in male headed households (DID=-513.7INR, 95% CI= -843.9: -183.4, p=0.0023), scheduled castes (DID= -708.7INR , 95% CI=-1234.3: -183.2, p=0.0082), all 'other' social groups (DID=-1110.46INR, 95% CI=-1868 : -352.9, p=0.0041), rural households (DID= -504 INR, 95% CI= -801.9: -206.0, p=0.0009) and the poorest (DID= -1001.3 INR, 95% CI= -1751: -251.7, p= 0.0089) and middle asset quintiles (DID= -798.1 INR, 95% CI= -1362.9:-233.3, p= 0.0056).

Table 3: Change in average inpatient expenditure (in INR) in Maharashtra and Andhra Pradesh between 2004 and 2012

Subgroups	Baseline Mean (95% CI)		Change 2004:2012 Mean (95% CI)		DID	
	Maharashtra	Andhra Pradesh	Maharashtra	Andhra Pradesh	Mean (95%CI)	p
**Household inpatient expenditure**						
All	1091.6(978.4:1204.8)	723.5(527.5:919.5)	942.8(749.9:1135.6)	444.55(221.5: 667.6)	-498.2(-792.9:-203.5)	0.0009
					**DID (adjusted for covariates)**	
					-565.8(862.9: -268.6)	0.0002

**Head of Household**						
Male	1,132.9(1015:1,251)	758(419.7:1,096.3)	935(727:1,143.01)	1074.9(555.9:1593.8)	-513.7(- 843.9: - 183.4)	0.0023
Female	757.1(555.5:1014.6)	341.1(222.3:460.01)	421.3(164.7:678.0)	589.9(307.22:872.8)	-484.9(- 1075.6:105.9)	0.1076
**Social Group**						
Scheduled Tribes	376.6(231.7:521.6)	432.7(212.52:652.9)	1153.1(803.3:1502.9)	675.2(163.2:1187.2)	-477.9(- 1097.7:142)	0.1307
Scheduled Castes	696.7(500.2:893.2)	432.6(305.6:559.4)	1464.1(1039.9:1888.4)	755.4(444.9:1065.9)	-708.7(- 1234.3: - 183.2)	0.0082
Other Excluded	1,028.6(838.4:1218.8	562.4(463.7:662)	928.9(532.9:1324.9)	767.9(569.6:966.2)	-161(-603.7: 281.7)	0.4758
All other Groups	1,424.5(1,222.3:1626.7)	1306.2(627.8:1984.6)	734.9(427.9:1041.7)	-375.6(- 1,068.5:317.4)	-1110.46(- 1868 : -352.9)	0.0041
**Location**						
Rural	897.8(768.1:1027.5)	571.4(496.2:646.6)	1084.7(826.3:1343.1)	580.7(432.2:729.2)	-504(-801.9: -206.0)	0.0009
Urban	1343.5(1146.1:1540.9)	1113.5(466.2:1760.8)	753.6(458.7:1048.6)	92.3(-586.92:771.5)	-661.3(- 1401.5: 78.864)	0.0799
**Quintile**						
poorest	656.3(498.0:814.6)	391.5(319:464.1)	1692.5(1053.3:2331.7)	691.2(298.9:1083.5)	-1001.3(- 1751: -251.7)	0.0089
2nd	786.5(583.5:989.5)	443.3(356.5:530.2)	979.3(599.4:1359.2)	839.5(465.7:1213.3)	-139.8(- 672.5:393)	0.607
middle	1062.7(738.8:1386.1)	862.1(577.8:1146.5)	1011.8(550.2:1473.4)	213.7(-112.1:539.6)	-798.1(- 1362.9: - 233.3)	0.0056
4th	1241.7(894.4:1589.1)	1819(337.5:3302.5)	803.6(328.7:1278.5)	-644.3(-2128.3: 839.7)	-1447.9(- 3005.2:109.5)	0.0684
richest	1818.6(1505.5:2131.8)	908.3(682.1:1133.4)	252.3(-193.4:698.1)	362.1(15.3:708.9)	109.7(- 454.80:674.3)	0.7031

For peer review only

Large expenditures for inpatient care

Proportions of households incurring large expenditures showed an increase in both states (Table 4), but the increase was smaller in AP for the sample as a whole as well as for all the groups except for the second asset quintile. The DID was strongly significant for the households overall (adjusted DID=-1.8, 95% CI: -3:-0.7, p= 0.0009), but this was not observed for any of the sub groups of analysis.

Table 4: Change in the proportion (%) of households incurring large health expenditures for in-patient care in Maharashtra and AP between 2004 and 2012

Subgroups	Baseline Mean (95% CI)	Change 2004:2012 Mean (95% CI)	DID

For peer review only - http://bmjopen.bmj.com/site/about/guidelines.xhtml

	Maharashtra	Andhra Pradesh	Maharashtra	Andhra Pradesh	Mean (95%CI)	p
**Large IP expenditure (INR 23000 deflated to 2004 figures)**						
**All**	6.7(6:7.3)	3.4(2.9:3.9)	3.1(2.1:4.1)	2.2(1.5:2.8)	-0.91(-2.1:0.27)	0.1302
					**DID (adjusted for covariates)**	
					-1.8(-3:-0.7)	0.0009
**Head of Household**						
Male	6.8(6.2:7.6)	3.5(3.1:4)	3.1(2.0:4.1)	2.1(1.5:2.9)	-0.8(-2.1:-0.4)	0.1928
Female	5.0(3.7:6.4)	2.8(1.9:3.7)	3.9(2:5.8)	2(6.6:3.4)	-1.8(-4.2:0.50)	0.1222
**Social Group**						
Scheduled Tribes	2.2(1.3:3.1)	1.4(0.5:2.2)	5.3(3.5:7)	3.5(1.9:5.1)	-1.7(-4.1:0.61)	0.1478
Scheduled Castes	6.1(4.6:7.7)	2.1(1.5:2.6)	4.3(2.3:6.3)	3(1.9:4.1)	-1.2(-3.5 :1.01)	0.2785
Other Excluded	5.9(4.7:7.0)	3.3(2.7:3.8)	2.9(1.1:4.6)	2.7(1.7:3.6)	-2.1(-2.2:1.8)	0.8389
All other Groups	8.3(7.5:7.9)	5.4(4.4:6.3)	2.2(0.9:3.6)	0.51(-0.7:1.7)	-1.7(-3.5:0.04)	0.0628
**Location**						
Rural	1.9(1.5:2.2)	0.9(0.79:1.1)	1.7(1.1:2.3)	1.3(0.09:1.6)	-0.45(-1.1:0.25)	0.2098
Urban	12.9(11.9:14)	9.7(8.9:10.7)	4.4(3.0:5.7)	3.9(2.6:5.3)	-0.7(-2.4: 1.5)	0.6350
**Quintile**						
poorest	1.8(1.3:2.4)	1.1(0.8:1.4)	3.7(2.2:5.2)	1.7(0.7:2.7)	-0.2(-3.8:-0.19)	0.0307
2nd	2.7(1.9:3.5)	1.2(0.9:1.5)	2.1(0.93:3.3)	2.2(1.4:3.1)	0.9(-1.4:1.6)	0.9079
middle	6.9(4.9:8.9)	4.2(3.1:5.3)	1.3(-1:3.6)	0.9(-1.2:1.4)	-1.2(-3.9:1.4)	0.3596
4th	10.7(8.7:12.6)	7.6(5.9: 9.2)	1.8(-0.57:4.3)	-0.036(-1.9:1.80)	-1.9(-4.9:1.2)	0.2268
richest	13.5(12:14.9)	9.6(8.1:11.2)	0.3(-1.6:2.2)	-0.6(-2.8:1.6)	-0.9(-3.7:2)	0.5601

Changes in large borrowing for inpatient care

In both states proportions of households incurring large borrowings to meet inpatient expenses increased from 2004 to 2012 (Table 5). However, there was a consistent pattern of smaller increases in AP for the overall population, as well as all subgroups (except the richest asset quintile) with DIDs strongly significant for the overall population (adjusted DID=-4, 95% CI: -6.6:-1.4, p= 0.0032), scheduled tribes (DID= -5.5, 95% CIs:-9.3:-1, p= 0.0048), rural households (DID= -4.7, 95% CIs: -7.3 : -2.1, p= 0.0007) and all asset quintiles except the richest (the poorest asset quintile DID= -9.0, 95% CI: -14.0: -4.4, p=0.0002).

Table 5: Change in the proportion (%) of households large borrowings for in-patient care in Maharashtra and AP between 2004 and 2012

Subgroups	Baseline Mean (95% CI)		Change 2004:2012 Mean (95% CI)		DID	
	Maharashtra	Andhra Pradesh	Maharashtra	Andhra Pradesh	Mean (95%CI)	p
**Proportion of households having large borrowings**						
**All**	7.5(6.7:8.2)	3.8(3:4.5)	8.9(6.8:11)	5.3(3.4:7.2)	-3.7(-6.4:-0.908)	0.0100

For peer review only - http://bmjopen.bmj.com/site/about/guidelines.xhtml

					DID (adjusted for covariates)	
					-4(-6.6:-1.4)	0.0032
**Head of Household**						
Male	7.8(7.0:8.5)	3.9(3.1:4.7)	9.8(6.6:1.3)	5.3(3.3:7.3)	-3.6(-6.6: -0.62)	0.0187
Female	5(3:7)	2.9(1.7:4.1)	8.(6.6:11.2)	5(2.9:7.23)	-4.7(-8.3:-1)	0.0137
**Social Group**						
Scheduled Tribes	3.6(2.3:4.9)	2.4(0.93:3.9)	11(8.9:14)	5.8(2.8:8.8)	-5.5(-9.3: -1.8)	0.0048
Scheduled Castes	7.2(5.5:8.8)	3(1.7:4.2)	9.6(6.6:13)	5.8(3.4:8.3)	-3.8(-7.5:0.03)	0.0518
Other Excluded	8.0(6.8:9.2)	3.5(2.7:4.4)	8(5.8:10.3)	5.3(3.2:7.4)	-2.8(-5.7:0.19)	0.0661
All other Groups	8(7.15:8.8)	0.052(.040:0.064)	8.8(5.9:12)	4.7(2.2:7.4)	-4.1(-7.9:-.4.0)	0.0302
**Location**						
Rural	6.5(5.6:7.5)	0.03(0.024:0038)	10(8.5:12)	5·8(3.9:7.6)	-4.7(-7.3:-2.1)	0.0007
Urban	8.7(7.3:10)	0.056(0.048:0.064)	7.0(4.5:9.5)	4(1.1:6.9)	-3.0(-6.7:0.68)	0.1081
**Quintile**						
poorest	5.2(3.9:6.5)	0.025(0.016:0.033)	12.1(7.8:16)	3.1(1.3:0.049)	-9(-14:-4.4)	0.0002
2nd	0.064(0.048:0.08)	0.027(0.021:0.032)	0.095(0.070:0.12)	0.052(0.034:0.070)	-0.043(-.073: -.013)	0.0062
middle	0.074(0.050:0.098)	0.048(0.031:0.065)	0.10(.073:0.133)	0.044(0.013:0.076)	-0.059(-.100:-.017)	0.0069
4th	0 .087(0.063:0.110)	0.06(.041:0.078)	0.083(0.061:0.104)	0.039(0.014:0.064)	-0.044(-.075:-.012)	0.0076
richest	0.10(0.090:0.12)	0.064(0.049:0.079)	0.045(0.0035:0.086)	0.049(-.0068:0.105)	0.0045(-.062:0.071)	0.8937

Hospital utilisation for in-patient care

Overall, hospitalisation rates have increased in both AP and Maharashtra (Table 6) but more so in AP (5.6 per 1000 population vs 2.2), although the DID was not statistically significant. The sub group analysis presented a mixed picture. For both male and female headed households there was a greater increase in hospitalisation in AP, but this reached moderate statistical significance only for female headed households (DID=27.6, 95% CI =1.1:54.1, p-value = 0.0415). There is an increase in hospitalisations among scheduled tribes in Maharashtra and a reduction in AP (DID = -19.8, 95% CI = -37.3: -2.3, p-value = 0.0272) but the opposite ~~trend~~picture was seen among 'other excluded' groups with an increase in AP and a reduction in MH (DID = 12.5, 95% CI= 1.2:23.9 p-value = 0.0309). In scheduled castes hospitalisations had increased in both states but more so in AP, while in the 'other' group there was a small increase in MH and a small reduction in AP. In the poorest quintile, the increase in hospitalisation was significantly greater in MH (DID= -14.4, 95%CI=-28:-0.31, p= 0.0451).

Table 6: Changes in hospitalisation (per 1000 population) in Maharashtra and AP between 2004 and 2012

Subgroups	Baseline Mean (95% CI)		Change 2004:2012 Mean (95% CI)		DID	
	Maharashtra	Andhra Pradesh	Maharashtra	Andhra Pradesh	Mean (95%CI)	p
**Hospitalizations per 1000 population**						

For peer review only

For peer review only - http://bmjopen.bmj.com/site/about/guidelines.xhtml

**All**	41.3(37.3:45.2)	31.5(27.8:35.3)	2.2 (-4.7:9.1)	5.6(-1.1:12.3)	3.4(-5.9 :12.7)	0.4636
					**DID (adjusted)**	
					0.7(-8.6:10.2)	0.8685
**Head of Household**						
Male	41.0(37.1:44.9)	31.7 (16.9:34.0)	1.9(-5.1:8.8)	4.4(-2.4:11.1)	2.5(-6.9:11.9)	0.5966
Female	51.6 (30.6:72.5)	25.5(27.7:35.7)	13.9(-7.53:35.4)	41.5 (24.6:58.4)	27.6(1.1:54.1)	0.0415
**Social Group**						
Scheduled Tribes	23.7(14.2 :33.1615)	35.5(21.9:49.1)	17.1(5.8:28.5)	-2.7(-16.95:11.5)	-19.8(-37.3: -2.3)	0.0272
Scheduled Castes	42.4(36.3:48.4)	29.5(22.6:36.5)	3.9(-7.9:15.7)	7.6(-2.6:17.7)	3.7(-11.4:18.7)	0.6268
Other Excluded	44.2(38.2:50.2)	29.9(25.5:34.3)	-1.9(-11.2 :7.3109)	10.6(3.4:17.8)	12.5(1.2:23.9)	0.0309
All other Groups	42.5(37.1:47.9)	34.9(29.0:40.9)	0.93(-7.3:9.1)	-1.1(-9.4: 7.4)	-2.0(-13.5 :9.4)	0.7235
**Location**						
Rural	36.6(32.2:41)	28.9(26.4:31.5)	9.5(3.5:15.4)	8.8(2.2:15.3)	-0.69(-9.3:7.9)	0.8725
Urban	48.2(43.7:53)	38.2(35.5:41.2)	-7.9(-14.5: -1.3)	-2.5(-12.1:7.1)	5.4(-5.8:16.6)	0.3358
**Quintile**						
poorest	31.4(25.9:36.9)	27.5(22.8:32.1)	20.7(9:32.8)	6.4(-1.7:14.4)	-14.4(-28:-0.31)	0.0451
2nd	36.5(28.3:44.7)	27.1(21.9:32.4)	8(0.5:15.4)	8.9(-.56:18.4)	0.9(-10 : 12.5)	0.8746
middle	47.8(33.6:62.1)	35.3(29.0:41.6)	-1.5(-17.9:14.8)	4.1(-.56:13.7)	5.6(-12.8:24.0)	0.5457
4th	46.9(39.9:53.9)	41.7(32.7:50.8)	-4.0(-12:3.4)	-5.1(-15.7:5.4)	-0.75(-13.3:11.9)	0.9056
richest	51.0(46:56.1)	36.5(25.8:47.2)	-13.1(-18.9: -7.1)	1.5(-19.5:22.6)	15.0(-5.8: 3.6)	0.1665

LIMITATIONS OF THE STUDY

Difference in differences estimations aimed at assessing the impacts of interventions, assume that both populations demonstrate similar characteristics prior to the start of the intervention, and that 'unobservables' follow a common trend; under such circumstances, any differences in changes observed over time between the 2 populations are attributable to the interventions[39, 40]. Despite AP and Maharashtra having broadly similar socio-economic profiles, as well as our DID analysis taking account of a number of covariates, there may have been other factors resulting in unobserved differential changes between the 2 populations and to which the results of the DID analysis may be at least partially attributable.

A second limitation could arise from the impact of other public health programmes implemented during the period 2004 to 2012. The most significant of these is the National Rural Health Mission launched in 2005, mainly to improve maternal and child health through the revitalization of rural primary care and child and maternal health services. A key assumption of our study is that the impacts of the NRHM in terms of healthcare expenditure for maternal and child health care would have been similar in both states, as this was a nationwide development. Despite the improvements in the public sector maternal and child health services sought by the NRHM, it is widely recognised that the public, including BPL families, continues to pay OOP for private health care. We have assumed that this behaviour is likely to be similar across the 2 states. Other health initiatives such as the Navsanjeevani Yojana and HMRI were unlikely to have had an impact on inpatient care or

For peer review only

For peer review only - http://bmjopen.bmj.com/site/about/guidelines.xhtml

expenditure. The 108 scheme had the potential, in AP, to influence hospitalisation rates, by helping more households to visit hospitals when seriously ill. But the effect is likely to be small as the majority of even serious illnesses do not result in a 108 call for transport. The implementation of the RSBY and the scheme to make private hospital beds available for the poor in MH may have diluted the DID, although both schemes are known to have been only partially implemented.

Lastly, the 2004 NSSO survey which served as our baseline, was carried out between January and June 2004. Our end-line 2012 survey was carried out over a period of 3 months from June to September. The morbidity and mortality patterns recorded in different time periods may vary, and could have influenced the data.

DISCUSSION

We found that average expenditure, large expenditures and large borrowings on inpatient care had increased in both MH and AP, but the increase was consistently smaller in AP across these 3 outcome measures, and may be suggestive of Aarogyasri having a somewhat larger effect than RSBY. Similar increases in institutional deliveries across the 2 states, and low levels of utilisation of RSBY and Jeevandayee schemes in MH may further strengthen this explanation.

The increase in average OOPE on inpatient care in AP and MH reflects a ~~trend~~pattern observed nationwide[16, 41]. The Aarogyasri scheme may have contributed to the more favourable trajectory in AP both directly and indirectly, in that the scheme may have contributed to a reduction in the prices of interventions and an increase in competition among health care providers. The evaluation of the Yeshasvini scheme also found a significant reduction in the price of surgical interventions[10]. Our findings may suggest that the positive effects of Aarogyasri detected by other studies[15,16] at an early stage of the roll out of the scheme have been sustained. Automatic enrolment into the scheme, near universality of coverage and no requirement for enrollee contributions may have contributed to the significant DIDs in male headed households, scheduled castes, rural households and the poorest and middle asset quintiles. But these benefits were not demonstrated in some of the most vulnerable groups - female headed households and scheduled tribes. This is consistent with the findings of other studies[5,16] which reported that the slightly less vulnerable may benefit more from such schemes, with non-financial barriers undermining access to services for the most vulnerable socio-economic groups. The case summaries below illustrate the obstacles they face. A similar observation was reported by the evaluation of the Mexican Seguro Popular health scheme which showed that a third of the treatment-cluster households who were automatically affiliated were unaware of this fact[42].

The likely explanation of an Aarogyasri effect may be strengthened by the ~~trend~~changes in terms of ~~for~~ large borrowings which has also increased over time in both states but less so in AP across all groups of analysis except the richest asset quintile. A multi-country analysis of household catastrophic health expenditure highlighted that increasing the availability of health services is critical to improving health in poor countries, but it could also raise the proportion of households facing catastrophic expenditure unless financial risk protection policies are given a high priority[43]. Borrowing of comparatively small amounts is less impactful and may be a result of improved access to financial markets and also supports consumption smoothening. During recent decades, AP has in particular witnessed a significant rise in microfinance institutions and debt due to high levels of interest levied by the more recent entrants to this market[44, 45]. Nevertheless our results suggest that increases in large borrowings associated with inpatient health care were smaller in AP. Strongly significant DIDs in scheduled tribes, rural households and the poorest and second asset quintiles and moderately significant DIDs in female headed households, scheduled castes, and other excluded social groups may also point to Aarogyasri beginning to offer greater access to health care when families are faced with serious illness.

For peer review only - http://bmjopen.bmj.com/site/about/guidelines.xhtml

In terms of large OOPE, a significant DID was found only for the total households, but the direction of all DIDs except for the second asset quintile was the same as for average expenditure on inpatient care and large borrowings, i.e., in favour of AP. It is perfectly possible that the non-significant differences for the majority of sub groups are due at least in part to the RSBY, the availability of private sector hospital beds for the poor and the Jeevandayee schemes reducing large OOPE in MH, and that without these schemes the expenditure would have been greater. In AP, 70 percent of survey households reported that they were covered by the Aarogyasri scheme, but only 25 percent of these 'covered' households were aware that the benefit package was limited. It is also possible therefore that in AP some families seek hospital care assuming that the Aarogyasri scheme provides comprehensive cover, but are faced with large expenditures when their treatments fall outside the limits.

The mixed picture in relation to the increasing rate of hospitalisations in both states may suggest that the Aarogyasri and in particular, the RSBY scheme which covers common hospital procedures are addressing a large hitherto unmet need for inpatient care. The 108 scheme in AP may be an additional albeit smaller contributor. It may also be explained by a supplier-induced demand. An assessment of health provider behaviour and governance is an important strand of the evaluation of health financing schemes[46], but was outside the remit of our study. We would however strongly recommend an impact evaluation focusing on health care supply to complement our evidence. The greater increase in hospitalisation in AP, in female-headed households and 'other excluded groups', which are two of the most vulnerable population groups, is encouraging. But the reduction in hospitalisations among scheduled tribes and the poorest asset quintile in AP are of concern and suggest that if the poor are to secure the benefits appropriated by the near poor or more often by the rich[47], the provision of more comprehensive health schemes is essential, which combines the tertiary and secondary care focus of Aarogyasri and the secondary care benefits of RSBY with attention paid to minimise barriers such as the widespread influence of illiteracy and lack of awareness, which limit access to even schemes such as Aarogyasri that are apparently highly inclusive and non-discriminatory, as well as distance to facilities. Our case summaries illustrate this. Furthermore, health financing reforms such as the Jamkesmas[48] in Indonesia and the Seguro Popular in Mexico[42], both countries similar to India in terms of population and growing economies, include outpatient and inpatient care, and curative as well as preventive services, suggesting that a more comprehensive service is possible to implement and worthy of consideration.

In summary, health innovations in AP had a greater beneficial effect on hospital inpatient care-related expenditures than innovations in MH. The Aarogyasri scheme is likely to have contributed to these impacts in AP, at least in part. But in both states, OOPE increased over time, ~~a trend~~ in keeping with the picture reported nationwide[49]. Likely explanations are that the poor spend the largest proportion of OOPE on drugs[50], an expenditure not adequately addressed by the health financing schemes including the RAS which provide only 'follow up' medicines for limited periods, after discharge from hospital. Yet, chronic disease such as diabetes may result in a one-off cardio-vascular intervention funded by the RAS, but also life-long medication required to be paid for out-of-pocket. Other possible explanations are medical inflation and rising costs of initial consultation and diagnostic investigation of symptoms, often in the rapidly growing private sector out-patient services, prior to hospitalisation under the cover of the RAS or the other health financing schemes. Hospitalisations also increased, and while it is generally assumed that in developing countries this is likely to address genuine need, its impact on health status is not known.

IMPLICATIONS FOR POLICY AND PRACTICE

Despite these uncertainties, Aarogyasri is perceived, with some justification across India as a successful scheme, and is being rapidly replicated across the states. Since July 2012, MH too has joined the list of states offering an Aarogyasri-like scheme, the Rajiv Gandhi Jeevandayee Arogya

For peer review only - http://bmjopen.bmj.com/site/about/guidelines.xhtml

Yojana[51]. This study has highlighted that such schemes may result in some positive outcomes. Although the study was not designed to elicit the specific features of the scheme to which any comparatively greater benefits may be attributable, and which deserve to be replicated in other states, evidence from systematic reviews[3] points to the need for schemes to be more 'comprehensively' designed to maximise their positive impact. Aarogyasri's design in terms of its aims to address both financial and non-financial barriers - being fully state funded, the systematic administrative implementation of Aarogyasri across the state so that it is now almost universal, automatic enrolment, allowing access to the scheme via a ration card which most poor families own, the wide spectrum of treatments offered, the large number of health care providers empanelled - is perhaps responsible as a 'comprehensive' package, for the greater impact.

But the study also suggests that improved access to health care and the reduction of the overall burden of OOPE especially in the most vulnerable sections of the population[52] are likely to require additional interventions that address gaps in the availability of care and provide patients appropriate pathways that support their journey from a strong and comprehensive primary care service where they may be informed of their entitlements and investigated for their initial symptoms to appropriate hospitals for the treatment of serious illness. Besides, inpatient care is only consumed by a small proportion of households in a year[53], and this is especially true of tertiary care, while many more will seek outpatient services and referral to inpatient care, should this be required. Others have strongly recommended the strengthening of the primary care base as an essential means to universal health coverage and this study confirms their view[41]. Key implications for AP are to explore how best the most advantageous features of Aarogyasri can be extended to include both secondary and primary care, while those for MH may be to build on and unify its menu of currently available schemes to create an evidence-based comprehensive health delivery system. These conclusions may be applicable to other states with similar health financing schemes.

The design of health financing systems as well as their evaluations is complex and challenging, as the mountain of available evidence suggests[41, 42, 52, 54]. Even the ground-breaking Seguro Popular health insurance programme of Mexico which used a cluster-randomised trial design with strong government support demonstrated some but not all anticipated outcomes, contrary to expectations[42], and a key recommendation was that continued assessment of the programme was needed. Furthermore, differences in data and methodology may result in even very well designed evaluations of the same programme producing contrasting findings[52]. Our evaluation is the first to use a ~~quasi-experimental~~ survey methodology - the best possible in the hierarchy of evaluation methodologies, when a randomised control trial is not achievable - to evaluate the health financing reforms in 2 large states of India. Despite that, the study has limitations which we have acknowledged. For example, this study has examined health related expenditure and behaviours at only two points in time. To establish trends, it is suggested that more than two time points are needed. However, it needs to be recognised that schemes such as the Aarogyasri themselves may not have longevity in their original form and may change or be replaced in response to changing needs and policy imperatives. King et al acknowledged that their assessment of the Mexican Seguro Popular programme (at 10 months)[42] was undertaken at an early stage, but it was nevertheless recognised as having provided important evidence of impacts, albeit early ones. Therefore a realistic aim of an evaluation in this rapidly changing health delivery landscape in India would be to study change over time, even if that is limited initially to two time points, but to continue to evaluate the system repeatedly, and use the evidence to reshape health delivery to be responsive to future socio-economic and epidemiological trends. In addition, the evaluation was not designed to assess provider behaviours. Many other pertinent questions such as the impact of the schemes on the overall economy of health care cannot be answered by a single evaluation. But these are recognised problems which can only be addressed through continuous assessments, as other evaluations have shown. Our study has nevertheless produced sufficient insights to enable policy leaders to improve

For peer review only - http://bmjopen.bmj.com/site/about/guidelines.xhtml

programme effectiveness and, importantly, to undertake further assessment. Our household survey data provide a valuable baseline for future monitoring and analyses of trends in both states.

This study needs to be followed up with further and repeated evaluations as AP's and MH's schemes evolve; to assess the impacts of re-design and to help health policy leaders achieve their aspiration of universal access to good quality health care.

Three faces of the Aarogyasri Scheme

The beneficiary:

Lakshmamma, a 65 year old widow, is from a tribal background. She is an unskilled labourer, supporting a family of 5, with a monthly income of INR 4000 (USD 74). She was referred to a municipal hospital with chest pain and underwent heart surgery. The hospital which is a part of the Aarogyasri network provided free care and her total out of pocket expenses amounted to INR 850 (16 USD) for initial transport. In addition to the surgery, she received free food, money for transport home and follow up medicines. She is very satisfied with the service she received.

The excluded:

Ramulamma, a mother has a BPL card, but her daughter Lakshmi does not, as she was abandoned by her husband who is a government employee and entitled to free health care. The mother was unable to secure free care using her BPL card for her seriously ill daughter who paid out-of-pocket at a private facility where she was offered a hysterectomy for the relief of her gynaecological symptoms. Lakshmi's health care costs were met by the family selling a number of household assets and Lakshmi's daughter discontinuing her education to take up paid work. Lakshmi is severely depressed and does not speak to anyone.

The uninformed:

Krishnamma 43 years old, had severe stomach pains one night. Although the family had a BPL card, Ramulu her husband and Srinivas her son rushed her to a private hospital nearby, which was not part of the Aarogyasri network. They were unaware of how to access the Aarogyasri scheme hospitals which were further away from home, and the local primary health services being inadequate, Krishnamma had not had her initial symptoms investigated. The treatment was funded through a loan from a private moneylender. On discharge, she has not attended follow up, as the family cannot afford transport or medicines.

For peer review only - http://bmjopen.bmj.com/site/about/guidelines.xhtml

References

[1] Resolution WHA58.33. Sustainable health financing, universal coverage and social health insurance. In: *Fifty-eighth World Health Assembly, Geneva, 16–25 May 2005.* Geneva, World Health Organization, 2005 (http://apps.who.int/gb/ebwha/pdf_files/WHA58/WHA58_33-en.pdf, accessed 26th March 2013).

[2] World Health Organization. Health Systems Financing. Path to Universal Coverage. Geneva: World Health Organization. 2010. Available from: World Health Organization Library Information Services.

[3] Acharya A, Vellakkal S, Taylor F, Masset E, Satija A, Burke M, Ebrahim S. Impact of national health insurance for the poor and the informal sector in low and middle income countries : a systematic review. London: EPPICentre, Social Science Research Unit, Institute of Education, University of London. 2012. Available from: http://www.dfid.gov.uk/r4d/PDF/Outputs/SystematicReviews/Health-insurance-2012Acharya-report.pdf

[4] Giedion, U., B. Y. Diaz . A Review of the evidence. In: The Impact of Health Insurance in Low-and Middle-Income Countries. M. L. Escobar, C. Griffin and R. P. Shaw (ed). Washington DC, Brookings Inst Press 2011.

[5] Giedion, U, Alfonso, E , Diaz. *The impact of universal coverage schemes in the developing world : a review of the existing evidence*. Universal Health Coverage (UNICO) studies series ; no. 25. Washington D.C. : The World Bank. 2013

[6] National Commission on Macroeconomics and Health. Report of the National Commission on Macroeconomics and Health. New Delhi: Ministry of Health and Family Welfare of India. 2005. Available from: Ministry of Health and Family Welfare

[7] Eleventh Five Year Plan (2007–2012). Vol 2: Social Sector. Government of India Planning Commission. October, 2008. http://planningcommission.nic.in/plans/planrel/fiveyr/11th/11_v2/11th_vol2.pdf, (accessed Mar 26 2013).

[8] Faster, Sustainable and More Inclusive Growth. An Approach to the Twelfth Five Year Plan (2012-17). Government of India Planning Commission. October, 2011. http://planningcommission.nic.in/plans/planrel/12appdrft/appraoch_12plan.pdf, accessed 26th March 2013

[9] La Forgia G, Nagpal S. Government-Sponsored Health Insurance in India. *Are You Covered?* World Bank Washington DC 2012. doi:10.1596/978-0-8213-9618-6.

[10] Agarwal A. "Impact evaluation of India's 'Yeshasvini' community based health insurance programme. *Health Economics* 2010; 19: 5-35

[11] The Rajiv Aarogyasri scheme website at https://www.aarogyasri.org/ASRI/index.jsp, accessed 26th March 2013.

[12] RSBY scheme status. http://www.rsby.gov.in/statewise.aspx?state=35 (accessed Apr 30 2013)

[13] Mahal A. Learning and getting better: Rigorous evaluation of health policy in India. National Medical Journal of India. 2011. 325-27.

[14] Aarogyasri Health Care Trust Annual Report 2011-2012. https://www.aarogyasri.org/ASRI/EXT_IMAGES/documents/Annual_Report_201011.pdf (accessed Mar 26 2013)

[15] Rao M, Ramachandra S, Bandyopadhyay S, et al. Addressing healthcare needs of people living below the poverty line: a rapid assessment of the Andhra Pradesh Health Insurance Scheme. National Medical Journal of India. 2011. 24(6):335-41.

[16] Fan V, Karan A, Mahal A. "State Health Insurance and Out of-Pocket Health Expenditures in Andhra Pradesh, India." CGD Working Paper 298.Washington, D.C.: Center for Global Development. 2012 http://www.cgdev.org/content/publications/detail/1426275 (accessed Mar 15 2013)

[17] Palacios R, Das J, Sun C eds. India's health insurance scheme for the poor: Evidence from the early experience of Rashtriya Swasthya Bima Yojana. New Delhi: Center for Policy Research; 2011

[18] Rashtriya Swasthya Bima Yojana overview and scheme details. http://www.rsby.gov.in (accessed Apr 30 2013)

[19] Rathi P. http://www.priorities2012.com/documents/3d-4-patreek.pdf

[20] NRHM Mission Document.http://www.nird.org.in/brgf/doc/Rural%20HealthMission_Document.pdf (accessed Apr 30 2013)

[21] State HMIS data analysis, April'10-March'11 Andhra Pradesh, prepared by NHSRC.http://cfw.ap.nic.in/nrhm/pdf/AP_Analysis_Apr2010-Mar2011.pdf (accessed May 2 2013)

For peer review only - http://bmjopen.bmj.com/site/about/guidelines.xhtml

[22] NRHM achievements in Maharashtra. http://www.nrhm.maharashtra.gov.in/achievements.htm (accessed May 2 2013)
[23] Health information helpline.http://www.hmri.in/oursolutions-healthinformation.html (accessed Apr 30 2013)
[24] About the project/scheme. http://www.maha-arogya.gov.in/projectandschemes/Jeevandaiaarogya/default.htm (accessed Apr 30 2013)
[25] Jeevandayee scheme performance: Information received from Rajiv Gandhi Jeevandayee Arogya Yojana Society Govt of Maharashtra on May 17 2013
[26] Projects and schemes. http://maha-arogya.gov.in/projectandschemes/itdpnavsanjivani%5Cdefault.htm (accessed Apr 30 2013)
[27] Mallepeddi R , Pernefeldt H, Bergkvist S . Andhra Pradesh health sector reform, A narrative case study, Technical paper No.7 for The Rockefeller Foundation–Sponsored Initiative on the Role of the Private Sector in Health Systems in Developing Countries. 2009 ; pp 42-48
[28]EMRI. http://www.emri.in/states.html (accessed Apr 30 2013)
[29] Developing and evaluating complex interventions: new guidance. Medical Research Council. London 2008.
[30] Cesar G V, Jean-Pierre H, Jennifer B. Evidence-Based Public Health: Moving Beyond Randomized Trials. Am J Public Health. 2004 March; 94(3): 400–405.
[31] Presentation on Annual Plan 2012-13 and Five Year Plan 2012-17. http://planningcommission.nic.in/plans/stateplan/Presentations12_13/maharashtra1213.pdf (accessed on Mar 25 2013)
[32] National Sample Survey Organization. Morbidity, Healthcare and condition of the Aged. New Delhi: Ministry of Statistics and Programme Implementation. 2004.
[33] National Sample Survey Organization. Household Consumer Expenditure 66th round . New Delhi: Ministry of Statistics and Programme Implementation; 2009-10
[34] National Sample Survey Organization. Household Consumer Expenditure in India. New Delhi: Ministry of Statistics and Programme Implementation; 2005.
[35] Booysen F, Van der Berg S & Burger R. Using an Asset Index to Assess Trends in Poverty in Seven Sub-Saharan African Countries. World Development. 2008; 36(6): 1113–1130
36 Vyas S. & Kumarnayake L. Constructing socio-economic status indices: How to use principal components analysis. Health Policy and planning. 2006; 459-468
[37] O'Donnell O, Van Doorslaer E, Wagstaff A & Lindelow M, 2008. Analyzing Health Equity Using Household Survey Data: A Guide to Techniques and Their Implementation. Washington DC: World Bank Institute. Chapter 6, Measurement of Living Standards. http://siteresources.worldbank.org/INTPAH/Resources/Publications/459843-1195594469249/HealthEquityFINAL.pdf (accessed May 9 2013)
[38] Ministry of Finance. Economic Survey 2011-12. New Delhi: Government of India. 2012. Available from: http://indiabudget.nic.in/
[39] Poverty Reduction and Social Development Department. A User's Guide to Poverty and Social Impact Analysis. Washington: World Bank;2003
[40] Gertler P, Martinez S, Premand P, Rawlings L, Vermeersch C. Impact Evaluation in Practice. Washington: World Bank; 2011.
[41] Selvaraj S, Karan A. Why Publicly-Financed Health Insurance Schemes Are Ineffective in Providing Financial Risk Protection. Economic and Political Weekly 2012 March 17; XLVII(11):60-68
[42] King G, Gakidou E, Imai K, Lakin J, Moore RT, Nall C. Public policy for the poor? A randomised assessment of the Mexican universal health programme. Lancet. 2009 Apr 8; DOI:10.1016/S0140-6736(09)60239-7.
[43] Xu K, Evans DB, Kawabata K, Zeramdini R, Klavus J, Murray CJ. Household catastrophic health expenditure: a multicountry analysis. Lancet. 2003 Jul 12;362(9378):111-7.
[44] Naga Sridhar G. Microfinance institutions: Moving beyond AP crisis. The Business Line [Newspaper Online]. 2012 Dec 29. Available from: http://www.thehindubusinessline.com/companies/microfinance-institutions-moving-beyond-ap-crisis/article4253239.ece
[45] Naga Sridhar G. Life after microfinance in AP. The Business Line [Newspaper Online]. 2013 Jan 4. Available from: http://www.thehindubusinessline.com/opinion/life-after-microfinance-in-ap/article4273183.ece
[46] Prasad N P, Raghavendra P. Healthcare models in the era of medical neoliberalism. A study of Aarogyasri in Andhra Pradesh. Economic and Political Weekly 2012 Oct 27;XLVII(43):118-126

For peer review only - http://bmjopen.bmj.com/site/about/guidelines.xhtml

[47] Health Systems 20-20. An Evaluation of the Effects of the National Health Insurance Scheme in Ghana. Bethesda: Health Systems 20-20. 2009.
[48] http://www.jointlearningnetwork.org/programs/compare/benefits/142%2C16
[49] Ghosh S. Catastrophic Payments and Impoverishment due to Out-of-Pocket Health Spending. Economic and Political Weekly, 2011 Nov 19; XLVI(47): 63-70.

[50]Garg CC, Karan AK. Reducing out-of-pocket expenditures to reduce poverty: a disaggregated analysis at rural-urban and state level in India. Health Policy Plan. 2009; 24 (2):116-128.
[51] Rajiv Jeevandayi scheme details.
http://www.jeevandayee.gov.in/RGJAY/FrontServlet?requestType=CommonRH&actionVal=RightFrame&page=undefined%3E%3E%3Cb%3ERGJAY%3C/b%3E&pageName=RGJAY&mainMenu=About&subMenu=RGJAY (accessed on Apr 30 2013)
[52] Tangcharoensathien V, Swasdiworn W, Jongudomsuk P et al . Universal Coverage Scheme in Thailand: Equity Outcomes and Future Agendas to Meet Challenges. Geneva: World Health Organization. 2010.
[53] Dilip TR. On Publicy-Financed Health Insurance Schemes. Is the analysis premature? Economic and Political Weekly. 2012 May 5; XLVII(1)8:79-80.
[54] Vallakal S, Ebrahim S. Publicly-Financed Health Insurance Schemes. Concerns about Impact Assessment. Economic and Political Weekly. 2013 Jan 5; XLVIII(1);24-27.

For peer review only - http://bmjopen.bmj.com/site/about/guidelines.xhtml

STROBE Statement—Checklist of items that should be included in reports of ***cross-sectional studies***

	Item No	Recommendation
**Title and abstract**	1	(*a*) Indicate the study's design with a commonly used term in the title or the abstract Yes
		(*b*) Provide in the abstract an informative and balanced summary of what was done and what was found Yes
**Introduction**		
Background/rationale	2	Explain the scientific background and rationale for the investigation being reported Yes
Objectives	3	State specific objectives, including any prespecified hypotheses Yes
**Methods**		
Study design	4	Present key elements of study design early in the paper Yes
Setting	5	Describe the setting, locations, and relevant dates, including periods of recruitment, exposure, follow-up, and data collection Yes
Participants	6	(*a*) Give the eligibility criteria, and the sources and methods of selection of participants Yes
Variables	7	Clearly define all outcomes, exposures, predictors, potential confounders, and effect modifiers. Give diagnostic criteria, if applicable Yes
Data sources/ measurement	8*	For each variable of interest, give sources of data and details of methods of assessment (measurement). Describe comparability of assessment methods if there is more than one group Yes
Bias	9	Describe any efforts to address potential sources of bias Yes
Study size	10	Explain how the study size was arrived at Yes
Quantitative variables	11	Explain how quantitative variables were handled in the analyses. If applicable, describe which groupings were chosen and why Yes
Statistical methods	12	(*a*) Describe all statistical methods, including those used to control for confounding Yes
		(*b*) Describe any methods used to examine subgroups and interactions
		(*c*) Explain how missing data were addressed Method identical to baseline NSSO survey
		(*d*) If applicable, describe analytical methods taking account of sampling strategy
		(*e*) Describe any sensitivity analyses
**Results**		
Participants	13*	(a) Report numbers of individuals at each stage of study—eg numbers potentially eligible, examined for eligibility, confirmed eligible, included in the study, completing follow-up, and analysed Yes
		(b) Give reasons for non-participation at each stage
		(c) Consider use of a flow diagram
Descriptive data	14*	(a) Give characteristics of study participants (eg demographic, clinical, social) and information on exposures and potential confounders Yes
		(b) Indicate number of participants with missing data for each variable of interest
Outcome data	15*	Report numbers of outcome events or summary measures Yes
Main results	16	(*a*) Give unadjusted estimates and, if applicable, confounder-adjusted estimates and their precision (eg, 95% confidence interval). Make clear which confounders were adjusted for and why they were included Yes
		(*b*) Report category boundaries when continuous variables were categorized

For peer review only - http://bmjopen.bmj.com/site/about/guidelines.xhtml

		(*c*) If relevant, consider translating estimates of relative risk into absolute risk for a meaningful time period
Other analyses	17	Report other analyses done—eg analyses of subgroups and interactions, and sensitivity analyses Yes
**Discussion**		
Key results	18	Summarise key results with reference to study objectives Yes
Limitations	19	Discuss limitations of the study, taking into account sources of potential bias or imprecision. Discuss both direction and magnitude of any potential bias Yes
Interpretation	20	Give a cautious overall interpretation of results considering objectives, limitations, multiplicity of analyses, results from similar studies, and other relevant evidence Yes
Generalisability	21	Discuss the generalisability (external validity) of the study results Yes
**Other information**		
Funding	22	Give the source of funding and the role of the funders for the present study and, if applicable, for the original study on which the present article is based Yes

*Give information separately for exposed and unexposed groups.

Note: An Explanation and Elaboration article discusses each checklist item and gives methodological background and published examples of transparent reporting. The STROBE checklist is best used in conjunction with this article (freely available on the Web sites of PLoS Medicine at http://www.plosmedicine.org/, Annals of Internal Medicine at http://www.annals.org/, and Epidemiology at http://www.epidem.com/). Information on the STROBE Initiative is available at www.strobe-statement.org.

For peer review only

For peer review only - http://bmjopen.bmj.com/site/about/guidelines.xhtml
